# Genome-wide association study of brain amyloid deposition as measured by Pittsburgh Compound-B (PiB)-PET imaging

**DOI:** 10.1038/s41380-018-0246-7

**Published:** 2018-10-25

**Authors:** Qi Yan, Kwangsik Nho, Jorge L. Del-Aguila, Xingbin Wang, Shannon L. Risacher, Kang-Hsien Fan, Beth E. Snitz, Howard J. Aizenstein, Chester A. Mathis, Oscar L. Lopez, F. Yesim Demirci, Eleanor Feingold, William E. Klunk, Andrew J. Saykin, for the Alzheimer’s Disease Neuroimaging Initiative (ADNI), Carlos Cruchaga, M. Ilyas Kamboh

**Affiliations:** 1 Department of Human Genetics, University of Pittsburgh, Pittsburgh, PA USA; 2 Department of Pediatrics, Children’s Hospital of Pittsburgh of UPMC, University of Pittsburgh, Pittsburgh, PA USA; 3 Department of Radiology and Imaging Sciences, Indiana University School of Medicine, Indianapolis, IN USA; 4 Indiana Alzheimer Disease Center, Indiana University School of Medicine, Indianapolis, IN USA; 5 Department of Psychiatry, Washington University School of Medicine, St. Louis, MO USA; 6 Department of Neurology, University of Pittsburgh, Pittsburgh, PA USA; 7 Alzheimer Disease Research Center, University of Pittsburgh, Pittsburgh, PA USA; 8 Department of Psychiatry, University of Pittsburgh, Pittsburgh, PA USA; 9 Department of Radiology, University of Pittsburgh, Pittsburgh, PA USA

**Keywords:** Genetics, Psychology

## Abstract

Deposition of amyloid plaques in the brain is one of the two main pathological hallmarks of Alzheimer’s disease (AD). Amyloid positron emission tomography (PET) is a neuroimaging tool that selectively detects in vivo amyloid deposition in the brain and is a reliable endophenotype for AD that complements cerebrospinal fluid biomarkers with regional information. We measured in vivo amyloid deposition in the brains of ~1000 subjects from three collaborative AD centers and ADNI using ^11^C-labeled Pittsburgh Compound-B (PiB)-PET imaging followed by meta-analysis of genome-wide association studies, first to our knowledge for PiB-PET, to identify novel genetic loci for this endophenotype. The *APOE* region showed the most significant association where several SNPs surpassed the genome-wide significant threshold, with APOE*4 being most significant (*P*-meta = 9.09E-30; *β* = 0.18). Interestingly, after conditioning on APOE*4, 14 SNPs remained significant at *P* < 0.05 in the *APOE* region that were not in linkage disequilibrium with APOE*4. Outside the *APOE* region, the meta-analysis revealed 15 non-*APOE* loci with *P* < 1E-05 on nine chromosomes, with two most significant SNPs on chromosomes 8 (*P-*meta = 4.87E-07) and 3 (*P-*meta = 9.69E-07). Functional analyses of these SNPs indicate their potential relevance with AD pathogenesis. Top 15 non-*APOE* SNPs along with APOE*4 explained 25–35% of the amyloid variance in different datasets, of which 14–17% was explained by APOE*4 alone. In conclusion, we have identified novel signals in *APOE* and non-*APOE* regions that affect amyloid deposition in the brain. Our data also highlights the presence of yet to be discovered variants that may be responsible for the unexplained genetic variance of amyloid deposition.

## Introduction

Genomic efforts mainly through large-scale genome-wide association studies (GWAS), as part of the Alzheimer’s Disease Genetics Consortium (ADGC) [1] and the International Genomics of Alzheimer’s Project (IGAP) [2], have identified over 20 genes/loci for late-onset Alzheimer’s disease (AD). However, known common AD variants account for only ~30% of the AD genetic variance [3], and they also do not provide definitive information about the underlying disease mechanisms. Genetic studies focusing on AD-related quantitative phenotypes/endophenotypes may help to identify additional AD-related genes. One such AD-related phenotype is deposition of amyloid-beta (Aβ) in the brain, which is one of the two main pathologic hallmarks of AD; the other being the formation of tau deposits in the form of neurofibrillary tangles, neuropil threads, and dystrophic neurites (tau pathology) in the brain [4]. According to the current model for sporadic AD, Aβ pathology occurs independently of tau pathology, is detectable earlier, and is believed to accelerate neocortical tau pathology and neurodegeneration [5]. Recent longitudinal studies on cognitively normal subjects also confirm that amyloidosis is an early process in AD [6, 7]. The in vivo detection of Aβ deposition in the brain, as measured by positron emission tomography (PET) scanning with ^11^C-labeled Pittsburgh Compound-B (PiB) and the increased retention of PiB observed in the brains of AD patients compared with cognitively normal controls, was first reported by Klunk and colleagues [8, 9] and since has been confirmed in many studies [10]. There is a high correlation between amyloid PET imaging and neuritic plaque frequency as confirmed by autopsy studies [11–13]. Multiple studies have shown that amyloid PET has a high value for the clinical diagnosis of AD and in clinical trials aiming to reduce brain Aβ burden [14].

There is a well-established association of *APOE* variants with risk [1, 2] and age-at-onset [15, 16] of AD. Likewise, *APOE* genetic variation is also strongly associated with Aβ deposition in the brain as measured by PiB retention [17–19], indicating a genetic basis of Aβ deposition in the brain. Here, we used PiB-PET as an endophenotype to identify novel genetic loci for AD pathology using meta-analysis of three GWAS, the first to our knowledge, using the largest sample with the PiB-PET imaging from three different centers and the Alzheimer’s Disease Neuroimaging Initiative (ADNI).

## Materials and methods

### Sample description

All subjects with PiB-PET data were European-Americans and derived from three sites: University of Pittsburgh (PITT), Washington University (WU), and Indiana University (IU) combined with the initial phase of the multicenter ADNI PiB-PET add-on study (here they are referred to as ADNI/IU). All subjects provided informed consent, and all studies were approved by their local Institutional Review Boards. The summary statistics of these samples are included in Supplementary Table S1 and their description is given Supplementary Text.

### Amyloid-PET data

Detailed methods for acquisition and processing of PiB-PET scans are described in previous reports for the PITT [17, 18], WU [19], ADNI [20–22], and IU [23] studies. PiB retention was measured in four cortical regions of the brain, including medial frontal cortex (MFC; anterior cingulate/gyrus rectus), lateral frontal cortex (LFC), precuneus cortex (PRC), and parietal cortex (PAR) and expressed as a ratio to the cerebellum. In the GWAS meta-analysis, the PiB retention values from these four cortical regions were averaged in each subject to calculate a mean global score (GBL4) as the quantitative endophenotype. PiB retention was expressed as standardized uptake volume ratio (SUVR) in the PITT and ADNI/IU data [23, 24] and as binding potential (BP) in the WU data [25]. BP is approximately equal to SUVR-1. Because of this inconsistency in the PiB measurement methods, the GWAS data were analyzed via *P*-value-based meta-analysis as described below.

### Genotyping, imputation, and quality control

The genotyping platforms used for each study sample are listed in Supplementary Table S1. Imputation of non-genotyped single-nucleotide polymorphisms (SNPs) was performed with IMPUTE2 [26] using the 1000 Genomes Project [27] Phase III (May 2013 release) data as the reference panel for PITT and Phase I (November 2010 release) data for WU and ADNI/IU datasets. Full description of these procedures is given in Supplementary Text.

### Meta-analysis

METAL [28] software was used to perform meta-analysis on three GWAS, using the mean PiB-PET GBL4 value. METAL performs a *P*-value-based meta-analysis, which is appropriate when the effects being estimated are different in different cohorts. It does, however, account for differences in sample size between cohorts and for the direction of effects. The summary effect size was calculated by averaging the study-specific effect sizes, with weights reflecting the standard errors from the study-specific effect sizes.

### Functional analyses

To evaluate the biological significance of PiB-associated signals, we conducted five different analyses: differential gene expression in AD versus non-AD in relevant tissues, brain gene expression, expression quantitative trait loci (eQTL) analyses, and summary-data-based Mendelian randomization (SMR) analyses to test for pleiotropic association between gene expression and PiB, and pathway analyses. Detailed description of these analyses is given in Supplementary Text.

## Results

### Amyloid PET data characteristics

**Fig. 1:**
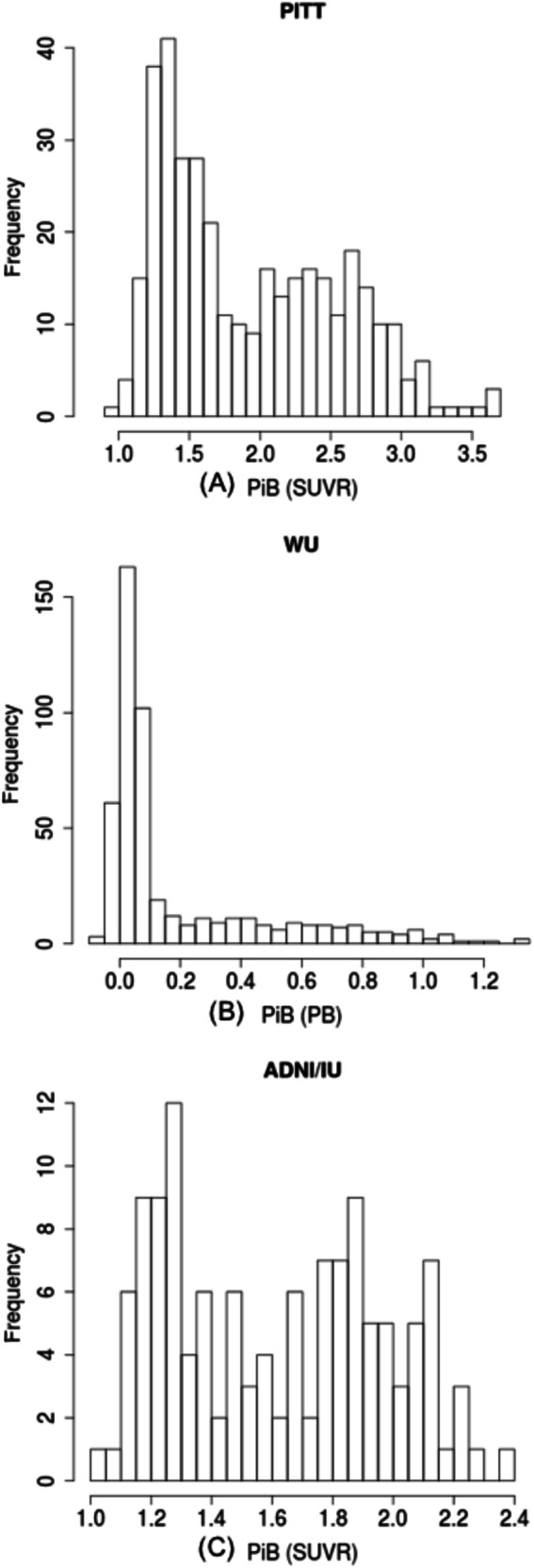
Distribution of PiB retention in the University of Pittsburgh (PITT) (**a**), Washington University (WU) (**b**), and the Alzheimer’s disease Neuroimaging Initiative (ADNI) and the Indiana Memory and Aging Study (ADNI/IU) (**c**) samples. SUVR standardized uptake volume ratio, BP binding potential

The characteristics of participants in each of the three datasets included in the meta-analysis are shown in Supplementary Table S1. The WU sample was younger with less male participants. The distribution of mean global PiB retention is shown in Fig. 1.

### GWAS analysis

**Fig. 2:**
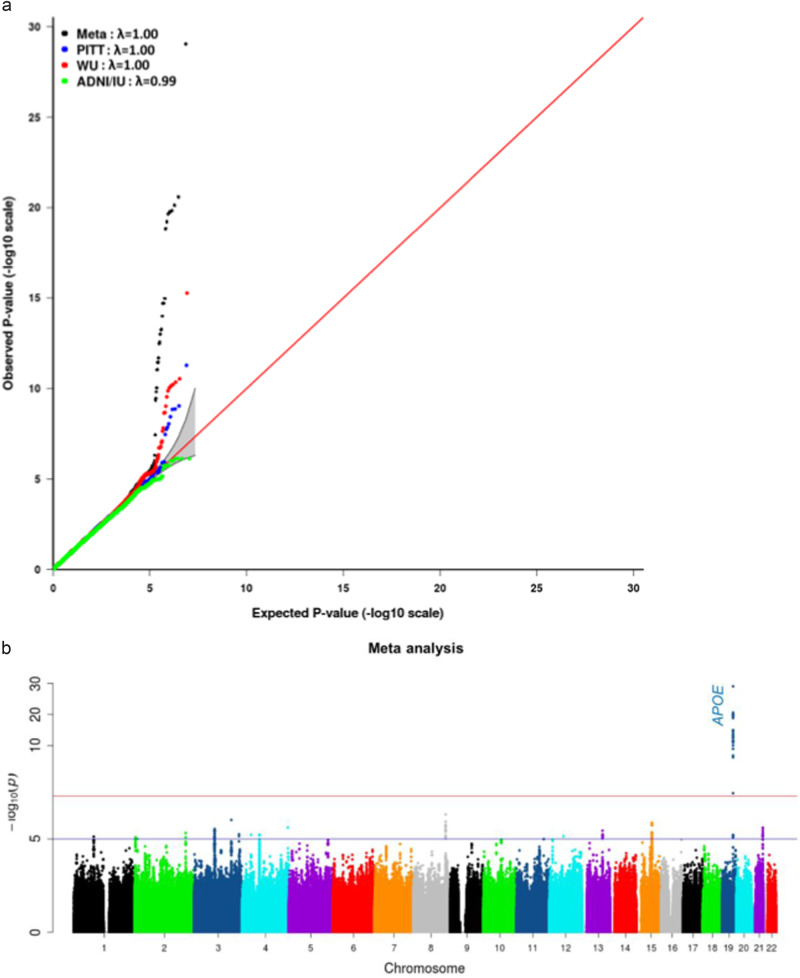
**a** Quantile–quantile plot for the individual GWAS results in the University of Pittsburgh (PITT), Washington University (WU), and the Alzheimer’s disease Neuroimaging Initiative (ADNI) and the Indiana Memory and Aging Study (ADNI/IU) datasets and in the meta-analysis. λ is the genomic control value. **b** Manhattan plot showing the *P*-values in the meta-analysis. The blue line represents the suggestive significance line (*P* < E-05). The red line represents the significance threshold (*P* < 5E-08)

**Fig. 3:**
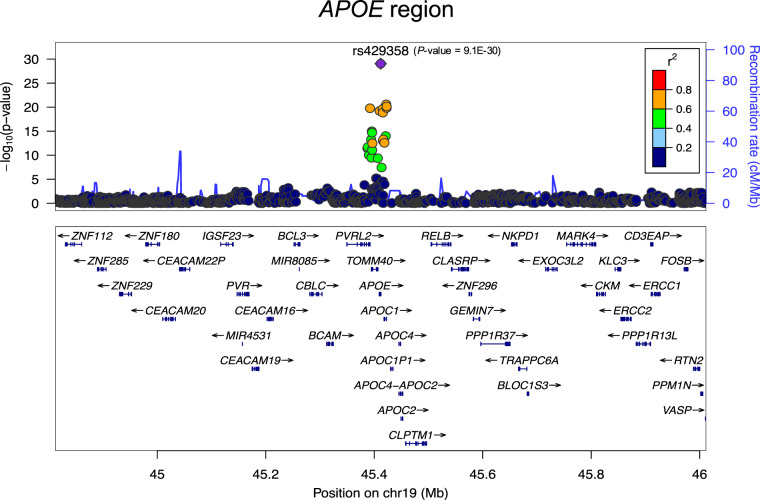
Regional plot of the *APOE* region on chromosome 19 in the meta-analysis. The relative location of genes and the direction of transcription are shown in the lower portion of the figure, and the chromosomal position is shown on the *x* -axis. The light blue line shows the recombination rate across the region (right *y* -axis) and the left *y*-axis shows the significance of the associations. The purple diamond shows the *P*-value for rs429358 that is the most significant SNP in the meta-analysis. The circles show the *P*-values for all other SNPs and are color coded according to the level of LD with rs429358 in the 1000 Genome Project EUR population

Quantile–quantile (QQ) plots and lambda values for the meta-analysis showed that neither the results from each of the three component studies nor the combined results from meta-analysis were inflated in their test statistics (Fig. 2a). Meta-analysis revealed 27 genome-wide significant SNPs (*P* < 5E-08) in a four-gene region on chromosome 19: *PVRL2-TOMM40-APOE-APOC1*(Fig. 2b, and Supplementary Table S2). As expected, APOE*4/rs429358 showed the most significant association with the average global PiB retention (*P*-meta = 9.09E-30; *β* = 0.18; Fig. 3, Supplementary Figure S1).

Outside of the *APOE* region, no genome-wide significant signal was observed. However, the meta-analysis revealed 15 non-*APOE* loci with *P* < 1E-05 on chromosomes 8, 3, 15, 4, 21, 13, 2, 12, and 1 (Table 1). Most of these loci show quite consistent results across all datasets. The regional plots of these 15 non-*APOE* loci are shown in Supplementary Figures S2.1–S2.15. The most significant SNP outside the *APOE* region is intergenically located between *ADCY8* and *EFR3A* on chromosome 8 (rs13260032; *P* = 4.87E-07, Supplementary Figure S2.1). The next most significant SNP is also intergenically located between *RAP2B* and *C3orf79* on chromosome 3 (rs4680057; *P* = 9.69E-07, Supplementary Figure S2.2). Chromosome 3 also harbors two additional signals: one in ncRNA (*LINC00971*/rs9831119; *P* = 2.98E-06, Supplementary Figure S2.6) and another near *MAGEF1*/ rs11923588 (*P* = 5.66E-06, Figure S2.9). The third most significant SNP is located in the *DAPK2* gene on chromosome 15 (rs12908891; *P* = 1.39E-06, Supplementary Figure S2.3). We also analyzed the data after adjusting for the effect of APOE*4/rs429358 in these non-*APOE* regions, which showed a slight attenuation of the association strengths (Table 1).

**Table 1:** Genetic loci associated with PiB-PET with *P* < 1E-05 in the meta-analysis

SNP	Chr	Position	A1	A2	Gene	Region	PITT			WU			ADNI/IU			Meta		Meta adjusted for APOE*4	
							MAF	Beta	*P*-value	MAF	Beta	*P*-value	MAF	Beta	*P*-value	Beta	*P*-value	Beta	*P*-value
rs429358	19	45411941	C	T	*APOE*	Exonic	0.20	0.35	5.20E-12	0.21	0.16	5.36E-16	0.28	0.19	7.88E-05	0.18	9.09E-30	NA	NA
rs13260032	8	132451455	C	A	*ADCY8,EFR3A*	Intergenic	0.43	−0.17	4.90E-05	0.44	−0.06	1.34E-02	0.43	−0.07	6.76E-02	−0.08	4.87E-07	−0.07	1.15E-05
rs4680057	3	153096985	A	G	*RAP2B,C3orf79*	Intergenic	0.45	0.16	2.60E-04	0.42	0.04	1.93E-02	0.46	0.11	8.07E-03	0.06	9.69E-07	0.05	2.61E-05
rs12908891	15	64236441	G	A	*DAPK2*	Intronic	0.46	0.12	6.38E-03	0.49	0.06	5.77E-06	0.50	0.01	9.01E-01	0.06	1.39E-06	0.04	5.30E-04
rs7377304	4	187129780	G	T	*CYP4V2*	Intronic	0.42	0.08	8.84E-02	0.46	0.06	5.98E-05	0.47	0.09	2.83E-02	0.06	2.46E-06	0.06	1.59E-05
rs55708341	21	45627581	T	A	*C21orf33,ICOSLG*	Intergenic	0.23	0.18	1.88E-04	0.18	0.05	4.65E-03	0.19	0.07	2.26E-01	0.07	2.51E-06	0.06	6.74E-05
rs9831119	3	84712077	C	T	*LINC00971*	ncRNA_intronic	0.12	−0.13	3.66E-02	0.14	−0.07	5.15E-04	0.12	−0.16	8.89E-03	−0.08	2.98E-06	−0.07	1.80E-05
rs9531483	13	84244873	A	C	*SLITRK1*	Intergenic	0.30	−0.12	7.81E-03	0.31	−0.06	5.62E-04	0.33	−0.07	1.12E-01	−0.06	3.65E-06	−0.05	8.45E-05
rs6722000	2	209075957	G	A	*C2orf80,IDH1*	Intergenic	0.21	0.14	7.27E-03	0.20	0.07	3.92E-04	0.22	0.06	2.37E-01	0.07	4.96E-06	0.07	1.71E-05
rs11923588	3	184459667	T	C	*MAGEF1,LOC101928992*	Intergenic	0.07	−0.20	2.19E-02	0.06	−0.19	6.50E-05	0.06	−0.14	1.53E-01	−0.18	5.66E-06	−0.13	1.56E-03
rs66837203	4	36897136	T	C	*DTHD1,MIR4801*	Intergenic	0.06	0.24	9.85E-03	0.08	0.10	3.53E-04	0.04	0.13	2.36E-01	0.11	6.03E-06	0.09	4.66E-04
rs200028958	4	70923661	A	G	*HTN1*	Intronic	0.10	0.29	2.99E-05	0.11	0.09	1.29E-03	0.09	−0.05	5.54E-01	0.10	6.25E-06	0.09	8.02E-05
rs4526799	12	57280586	T	C	*HSD17B6,SDR9C7*	Intergenic	0.34	−0.19	3.58E-05	0.34	−0.04	3.09E-02	0.36	−0.04	4.18E-01	−0.05	7.26E-06	−0.06	1.16E-06
rs17105538	1	81315043	G	A	*ELTD1,LPHN2*	Intergenic	0.13	0.13	3.65E-02	0.15	0.08	2.08E-04	0.17	0.09	1.10E-01	0.08	7.66E-06	0.07	1.58E-05
rs62121100	2	3093952	G	T	*LINC01250*	ncRNA_intronic	0.19	−0.20	1.09E-04	0.16	−0.05	1.03E-02	0.21	−0.04	4.79E-01	−0.07	8.44E-06	−0.06	2.90E-05
rs1809136	2	11152180	G	C	*KCNF1,FLJ33534*	Intergenic	0.07	0.28	5.91E-04	0.07	0.17	5.45E-04	0.08	0.00	9.95E-01	0.16	9.99E-06	0.15	6.36E-05

*A1* minor allele, *A2* major allele

### Conditional analysis in the *APOE* region

In order to check if there were independent SNPs associated with the PiB retention in the *APOE* region, we performed conditional analysis by adjusting for the top SNP (APOE*4/rs429358). A total of 14 SNPs remained significant at *P* < 0.05 (Table 2), including three SNPs that showed genome-wide significance before adjusting for APOE*4 (rs75627662, rs483082, and rs438811; Supplementary Table S2). Supplementary Figure S3 shows LD structure of these 14 SNPs along with APOE*4/rs429358 and APOE*2/rs7412 SNPs. APOE*4 and APOE*2 have essentially no LD with nine of the 14 SNPs that are located in the *PVRL2* gene (SNPs 1–9 in Supplementary Figure S3). One SNP located in the *APOE*/*APOC1* intergenic region (rs59325138) has only very weak correlation with APOE*4 (*R*^2^ = 0.15) and APOE*2 (*R*^2^ = 0.03), while three SNPs located downstream of *APOE* and *APOE/APOC1* intergenic region have weak to moderate LD with APOE*4 (*R*^2^ = 0.42, 0.64, 0.65 for rs75627662, rs483082, and rs438811, respectively).

**Table 2:** Conditional analysis on SNPs reaching *P* < 0.05 in the *APOE* region with additional adjustment for APOE*4 (rs429358) in the meta-analysis

SNP	Chr	Position	A1	A2	Gene	Region	PITT			WU			ADNI/IU			Meta		LD (*R*^2^) with	
							MAF	Beta	*P*-value	MAF	Beta	*P*-value	MAF	Beta	*P*-value	Beta	*P*-value	E*4	E*2
rs7412	19	45412079	T	C	*APOE*	Exonic	0.07	−0.21	7.63E-03	0.07	−0.04	1.45E-01	0.04	−0.06	5.49E-01	−0.06	3.69E-03	0.01	NA
rs3852859	19	45379309	C	T	*PVRL2*	Intronic	0.20	0.08	1.14E-01	0.20	0.05	1.10E-01	0.24	0.07	1.52E-01	0.06	8.81E-03	0.004	0.01
rs2075642	19	45377467	A	G	*PVRL2*	Intronic	0.20	0.07	1.78E-01	0.20	0.06	8.44E-02	0.22	0.07	1.33E-01	0.06	1.11E-02	0.004	0.01
rs3852856	19	45361574	A	G	*PVRL2*	Intronic	0.21	0.06	2.43E-01	0.20	0.07	3.16E-02	0.22	0.06	2.03E-01	0.06	1.15E-02	0.006	0.01
rs75627662	19	45413576	T	C	*APOE*	Downstream	0.23	−0.15	1.57E-02	0.23	−0.02	2.92E-01	0.26	−0.03	6.19E-01	−0.03	1.50E-02	0.42	0.21
rs4803767	19	45372959	T	C	*PVRL2*	Intronic	0.26	0.05	2.63E-01	0.28	0.04	1.56E-01	0.31	0.07	8.34E-02	0.05	2.06E-02	0.002	0.01
rs60389450	19	45372184	C	A	*PVRL2*	Intronic	0.26	0.04	3.20E-01	0.26	0.02	1.72E-01	0.31	0.07	8.34E-02	0.03	2.72E-02	0.003	0.01
rs59325138	19	45416291	T	C	*APOE,APOC1*	Intergenic	0.38	0.06	2.16E-01	0.36	0.02	2.50E-01	0.36	0.07	1.17E-01	0.03	3.10E-02	0.15	0.03
rs483082	19	45416178	T	G	*APOE,APOC1*	Intergenic	0.26	−0.10	1.60E-01	0.29	−0.04	1.59E-01	0.32	−0.06	4.51E-01	−0.05	3.35E-02	0.64	0.16
rs8104483	19	45372354	G	T	*PVRL2*	Intronic	0.27	0.03	4.65E-01	0.27	0.02	2.04E-01	0.32	0.09	4.20E-02	0.03	3.67E-02	0.003	0.01
rs3729640	19	45381917	T	C	*PVRL2*	UTR3	0.20	0.08	9.33E-02	0.19	0.03	3.35E-01	0.23	0.04	4.33E-01	0.05	3.79E-02	0.004	0.01
rs8104292	19	45372707	A	G	*PVRL2*	Intronic	0.27	0.04	4.14E-01	0.28	0.03	2.62E-01	0.32	0.09	4.20E-02	0.04	3.80E-02	0.003	0.01
rs438811	19	45416741	T	C	*APOE,APOC1*	Intergenic	0.26	−0.10	1.60E-01	0.30	−0.04	1.84E-01	0.32	−0.06	4.51E-01	−0.04	3.84E-02	0.65	0.16
rs58521715	19	45372129	T	A	*PVRL2*	Intronic	0.27	0.03	4.65E-01	0.27	0.02	2.53E-01	0.32	0.09	4.20E-02	0.03	4.50E-02	0.004	0.01

*A1* minor allele, *A2* major allele

The most significant SNP in meta-conditional analysis was APOE*2/rs7412 (*P*-meta = 3.69E-03; *β* = −0.06; Table 2), though it was not genome-wide significant before adjusting for APOE*4 (*P*-meta = 6.57E-05; *β* = −0.09). A similar strength of association was seen with an intronic *PVRL2*/rs3852859 SNP after adjusting for APOE*4 (*P*-meta = 8.8E-03; *β* = 0.06; Table 2) that was in LD with three additional SNPs (SNPs 1, 7, and 9 in Supplementary Figure S3). Three additional apparently independent associations were seen with rs4803767 (*P*-meta = 2.06E-02; *β* = 0.05 Table 2) that was in LD with four additional SNPs (SNPs 2–5 in Supplementary Figure S3), rs75627662 (*P*-meta = 1.50E-02; *β* = −0.03; Table 2) that was in LD with two additional SNPs (SNPs 13, 15 in Supplementary Figure S3), and rs59325138 (*P*-meta = 3.10E-02; *β* = 0.03; Table 2) that has very weak correlation with all other SNPs (*R*^2^ = 0.01–0.24).

### Association of known AD risk loci with amyloid burden and association of amyloid loci with AD risk

We examined the top IGAP genome-wide significant SNPs (Supplementary Table S3.1) and the associated gene regions (Supplementary Table S3.2) in relation to amyloid burden and found only some nominally significant SNPs. Likewise, we examined the suggestive non-*APOE* amyloid loci in our PITT-ADRC case–control sample of >2200 subjects [29] and found association of two top amyloid-associated SNPs with AD risk (Supplementary Table S4.1). When we examined additional Aβ-associated SNPs in each region with AD risk, we found multiple associations with *P* < 0.05 (Supplementary Table S4.2), indicating that our suggestive Aβ-associated loci are also associated with AD risk (see Supplementary Text for more details).

### Estimation of amyloid-PET variance by *APOE* and non-*APOE* loci

The genetic variance was estimated based on the R-square calculated from a linear regression model regressing global PiB retention on six independent *APOE* SNPs (rs429358, rs7412, rs3852859, rs4803767, rs75627662, and rs59325138), as described above, and 15 non-*APOE* SNPs given in Table 1. The contribution of six *APOE* SNPs to the variance of global PiB retention was 28.0, 17.3, and 17.12% in the PITT, WU, and ADNI/IU datasets, respectively; APOE*4/rs429358 alone explained 17.5, 16.5, and 13.9%, respectively. The top 15 non-*APOE* SNPs explained 22.6, 21.6, and 21.7% of the amyloid variance in the PITT, WU, and ADNI/IU datasets, respectively. The consistency of these estimates across the different datasets gives confidence that the difference in measurement of PiB across the datasets does not affect the bottom-line results.

### Functional analyses

We performed five analyses (Methods section) to evaluate the biological significance of PiB-implicated signals/genes. We considered all genes within ±500 kb of the top variant in each locus from Table 1 plus any eQTL-controlled genes outside the ±500 kb boundary as target genes (Fig. 3, Supplementary Figures S2.1-S2.15) and selected a total of 257 genes.

**Fig. 4:**
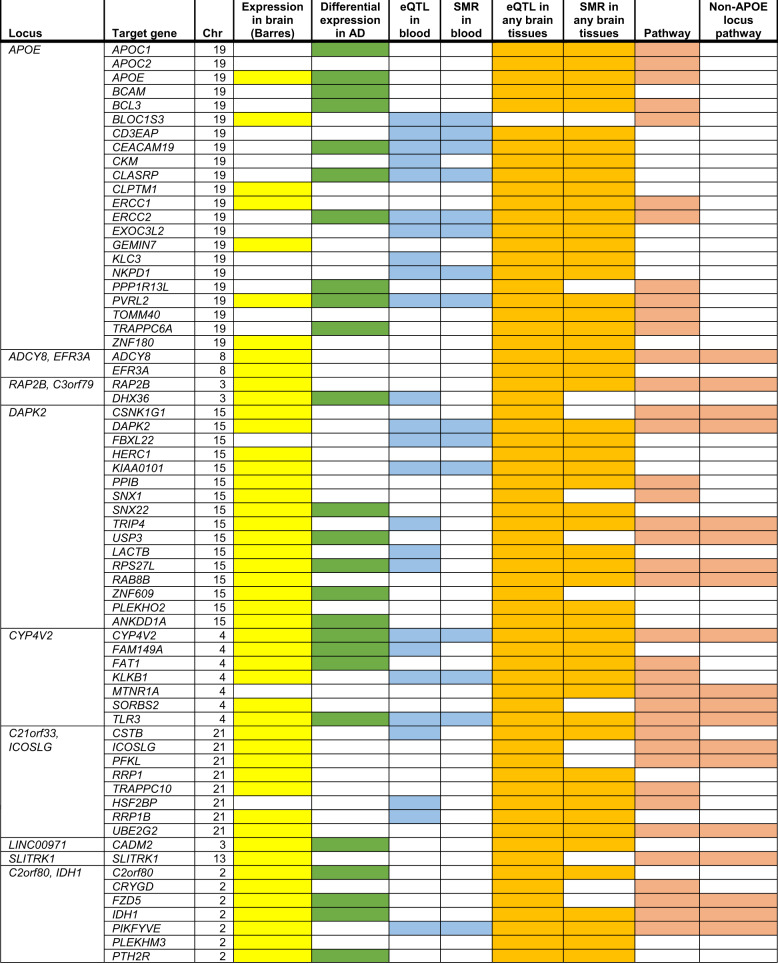

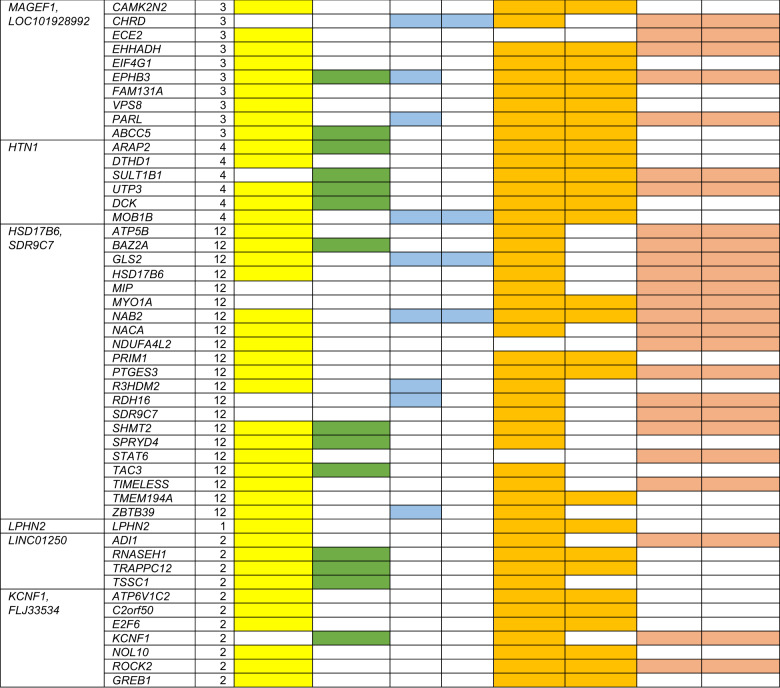
The functional analysis results for target genes. Out of 257 target genes, only genes meeting at least three functional criteria are listed. The criteria include: (1) differential expression in at least two Alzheimer disease studies that up- or downregulated consistently in different studies; (2) expression in the brain cells (Barres website); (3) having *cis*-eQTL effects in any brain tissues using GTEx database (*P* < 0.05); 4) mediating genetic effects on PiB (SMR analysis with *P* < 0.05) in any brain tissues; (5) having *cis*-eQTL effects in whole blood (*P* < 0.05); (6) mediating genetic effects on PiB (SMR analysis with *P* < 0.05) in whole blood; and (7) included in nominally significant pathways. The detailed results are summarized in Supplementary Table S6

Of 257 target genes, we found 20 upregulated and 25 downregulated genes that were differentially expressed in the same direction in two or more AD studies and no opposite directions were reported (Fig. 4 and Supplementary Table S6 marked in green color). Brain RNA-seq data reveals that many of these differentially expressed candidate genes are expressed in AD-relevant cell types (Fig. 4 and Supplementary Table S6 marked in yellow color).

For eQTL analyses, we identified SNPs in LD (*R*^2^ ≥ 0.5) with the top SNP for each locus in Table 1. For these SNPs, there were *cis*-acting eQTLs (eQTL *P* < 0.05) for 151 of the 257 target genes in various brain tissues and 36 genes in whole blood available in GTEx. Supplementary Table S5 gives the eQTL results for each top SNP in 15 non-*APOE* loci, and the detailed results of LD SNPs (*R*^2^ ≥ 0.80) with top SNPs are given in Supplementary Table S7. With the exception of *SLITRK1*/rs9831119, the other 14 top SNPs were eQTLs in different brain regions; 11 of them were eQTL in anterior cingulate cortex/frontal cortex/cortex where PiB intake is highest [30], indicating their role in affecting amyloid deposition in the brain.

For SMR analyses, only the gene/variant pairs identified in the *cis*-eQTL analyses were considered. For these gene/variant pairs, 99 genes in any brain tissue and 19 in whole blood were shown to mediate genetic effects on PiB by cis-regulating gene expression (SMR *P* < 0.05; Fig. 4, Supplementary Table S6).

We conducted pathway analyses (MAGMA [31]) using four gene set resources, including and excluding target genes in the *APOE* region, and detected nine genome-wide significant pathways: NDK dynamin pathway, *FDR* = 4.6E-04; synaptic vesicle recycling, *FDR* = 3.5E-07; synaptic vesicle endocytosis, *FDR* = 3.1E-04; protein depolymerization, *FDR* = 3.1E-04; inositol tetrakisphosphate phosphatase activity, *FDR* = 5.7E-03; positive regulation of vacuole organization, *FD* *=* 5.7E-03; inositol trisphosphate phosphatase activity, *FDR* = 0.033; regulation of clathrin-mediated endocytosis, *FDR* = 0.038; and clathrin-mediated endocytosis, *FDR* *=* 0.043. Although none of the 257 target genes, including *APOE*, are included in these nine genome-wide significant pathways, 71 target genes are included in the nominally significant pathways, and 46 target genes are included in the non-*APOE* region-related nominally significant pathways (*P* < 0.05. Fig. 4 and Supplementary Table S6 marked in pink color).

## Discussion

In this investigation, we have used the largest PiB-PET imaging data (*n* = ~1000), available from multiple collaborative centers, as an endophenotype to identify novel genetic loci for AD pathology using the GWAS meta-analysis approach, the first to our knowledge for PiB-PET.

The *APOE* region showed the most significant association where several SNPs surpassed the genome-wide significant threshold *(P* < 5E-08), with APOE*4 as the top hit that was associated with higher PiB retention in the brain (*P*-meta = 9.09E-30; *β* = 0.18). APOE*2, a protective genetic factor against AD, was associated with lower PiB retention, albeit, not genome-wide significant (*P*-meta = 6.57E-05; *β* = −0.09). This observation is consistent with earlier reports of the association of the APOE* 2/3/4 polymorphism with Aβ deposition in the brain as measured by PiB-PET [17–19] or florbetapir-PET [32]. Likewise, a GWAS of cerebrospinal fluid (CSF) Aβ has identified a genome-wide significant SNP that was a proxy for APOE*4 [33]. Numerous prior studies have investigated the role of the APOE* 2/3/4 polymorphism on Aβ production, aggregation, and clearance in the brain [34], but recent studies provide solid mechanistic clues into the role of *APOE* genetic variation in affecting APP transcription and Aβ production [35], and seeding of amyloid pathology [36]. In addition to the APOE*2/3/4 association, conditional analysis on APOE*4 identified 14 independent signals in the *APOE* region that also affect brain amyloidosis. Nine of 14 SNPs had essentially no LD with APOE*4 and APOE*2, and the remaining five showed moderate to weak LD with APOE*4. Thus, our meta-analysis indicates the presence of additional signals in the *APOE* region, beyond the APOE*4/rs429358 and APOE*2/rs7412 SNPs, that affect Aβ deposition in the brain.

Outside the *APOE* region, the meta-analysis revealed 15 suggestive non-*APOE* loci with *P* < 1E-05 on nine chromosomes. Although they do not meet the established genome-wide significance criteria, their consistent and directional associations in three independent datasets (Table 1) suggest that at least some of them are likely candidate loci for brain amyloidosis process and/or AD risk and variants in these loci may have achieved the genome-wide significance threshold in larger datasets. Credence to this idea was provided by our observation that most of these suggestive loci were also associated with AD risk when we examined the Aβ-associated SNPs in a published AD GWAS [29] (Supplementary Tables S4.1-S4.2). The most significant non-*APOE* SNP (rs13260032; *P* = 4.87E-07) on chromosomes 8 is intergenic, and this was an eQTL for a nearby *ADCY8* gene in frontal cortex, which is one of the highest PiB uptake cortical regions [30]. *ADCY8* is essential to long-term potentiation and synaptic plasticity and is implicated in memory and learning [37]. Genetic variation in or around *ADCY8* has shown to be associated with dissociation symptoms in subjects with post-traumatic stress disorder [37], abdominal visceral [38] and alcohol-dependent depression [39]. The second top SNP (rs4680057; *P* = 9.69E-07) resides near *C3orf79* and was an eQTL for a nearby long noncoding RNA (lncRNA) gene in anterior cingulate cortex and hippocampus in the brain and for *ARHGEF26* in blood. lncRNAs play a critical role in gene regulatory networks and may affect diverse biological processes and diseases [40], including AD where several IncRNAs have been shown to regulate Aβ production/generation [41, 42]. A recent GWAS has identified *ARHGEF26* as a new genetic factor for coronary artery disease risk that influences the transendothelial migration of leukocytes [43]. The third top SNP (rs12908891; *P* = 1.39E-06) is located *DAPK2* on chromosome 15 that belongs to a family of related serine/threonine kinases shown to be involved in multiple functions, including apoptosis, autophagy, tumor suppression, and inflammation [44]. Although the role of *DAPK2* in amyloidosis in unknown, another family member, *DAPK1*, promotes the phosphorylation and amyloidogenic processing of APP [45]. The *DAPK2* region contains other candidate genes, such as *GSNK1G1* and *TRIP4*. While *TRIP4* is a known gene for AD [46], *GSNK1G1* has been implicated in the formation of Aβ [47]. The top SNP was the most significant eQTL for *HERC1* gene expression in anterior cingulate cortex (*P*_eQTL= 7.02E-05; *P*_SMR = 1.94E-03). HERC1 belongs to the ubiquitin–proteasome system that plays a key role in the protein degradation pathway essential for neuronal homeostasis, synaptic development and maintenance. Mutations in *HERC1* have been associated with intellectual disability [48] and autism spectrum disorders [49].

To identify additional PiB-relevant candidate genes, we combined results from the brain expression, differential brain expression in AD, eQTL/SMR in the brain, and pathway analyses. Four genes meeting all these functional criteria were identified: *RPS27L* in the *DAPK2* region, *CYP4V2* and *TLR3* in the *CYP4V2* region, and *IDH1* in the *IDH1/C2orf80* region (Fig. 4, Supplementary Table S6). RPS27L is an evolutionarily conserved ribosomal protein and a physiological regulator of transcription factor p53 that is involved in genomic stability and tumor suppression [50]. p53 has also been implicated in AD progression, in part, due to its interaction with Aβ in AD progression [51]. p53 also interact with IDH1 in glioblastoma [52]. It seems that the involvement of RPS27L and IDH1 in the amyloidogenic process is through their effect on or interaction with p53. Although the role of CYP4V2 in amyloidosis is currently unclear, activated TLR3, along with some members of the toll-like receptors family, can induce Aβ uptake or inflammatory response during the AD progression [53]. Further functional characterization of these candidate genes may help to elucidate their roles in brain amyloidosis.

A recent GWAS using CSFAβ42 as an endophenotype has identified two novel loci in addition to the *APOE* locus [33]. One locus is near *GLIS1* on chromosome 1 and the other in *SERPINB1* on chromosome 6. The reported *GLIS1*/185031519 SNP was neither present in our genotyping array nor was it imputed. This SNP was also not in high LD with other SNPs. On the other hand, the reported *SERPINB1*/rs316341 SNP was present in our data, but it was not significant (*P* = 0.148). We also examined four additional reported *SERPINB1* SNPs with *P* < 1E-05 (rs316339, rs316337, rs392120, rsrs2293772) [33] and found one of them to be nominally significant in our data (rs392120; *P* = 0.033).

We estimated the genetic variance of global PiB retention explained by the *APOE* and top 15 non-*APOE* SNPs with *P* < 1E-05 using a linear regression model. The non-*APOE* SNPs along with APOE*4 explained 25–35% of the amyloid variance; of which 14–17% was explained by APOE*4 alone. A previous study using a different amyloid tracer (florbetapir-PET) [32] found a similar contribution of APOE*4 (11%) to amyloid variance. However, a GWAS on CSF Aβ42 found a smaller contribution of APOE*4 (4%) to amyloid variance [33]. This may be due to the use of different methods to estimate the amyloid variance. While the CSF study used the Genome-wide Complex Trait Analysis (GCTA) that requires >3000 sample size [54], the two amyloid tracer studies with smaller sample sizes used linear regression. Our data, in conjunction with previous studies, highlight the presence of yet to be discovered variants that may be responsible for the unexplained genetic variance of amyloid deposition.

As with any genome-wide study, this study has limitations. Although the present study used the largest combined sample of PiB-PET imaging data reported to-date (from three different centers and ADNI), the sample size was relatively small to achieve genome-wide significance for loci with small effect sizes. We predict that at least some of our suggestive loci with *P* < 1E-05 might have achieved genome-wide significance with a larger sample size, as the direction of allelic effects for all suggestive loci were consistent in all datasets. Unlike some other phenotypes where data could be obtained readily on large numbers of subjects at a relatively low-cost, this is not the case with amyloid PET. Thus, the lack of a very large PiB-PET imaging database for a genome-wide study was a significant constraint. As more PiB-PET imaging data are obtained by different centers, future collaborative studies, as done here, on larger samples may allow the identification of additional genes for brain amyloidosis.

In conclusion, this is the first GWAS on PiB-PET that has confirmed the established association of the *APOE* locus with in vivo brain amyloidosis. In addition to the known association, we have identified novel variants in the *APOE* region that affect amyloidosis. A combination of genetic and functional approaches has also led to the identification of additional putative candidate genes that warrant follow-up genetic and functional studies to confirm their role in brain amyloidosis.

## Electronic supplementary material

Supplementary Material

Tables S6

Table S7
