# Supplementary material for: Genome-wide association study of brain amyloid deposition as measured by Pittsburgh Compound-B (PiB)-PET imaging
Source: Mol Psychiatry. 2018 Oct 25;26(1):309–21. doi: 10.1038/s41380-018-0246-7 (PMC6219464; doi:10.1038/s41380-018-0246-7)
Supplement: Supplementary file 1 — Supplementary Material [file 41380_2018_246_MOESM1_ESM.pdf]

Supplementary Material

Genome-wide association study of brain amyloid deposition as measured by Pittsburgh Compound-B (PiB)-PET imaging

Qi Yan PhD[1,2], Kwangsik Nho PhD[3,4], Jorge L. Del-Aguila PhD[5], Xingbin Wang PhD[1], Shannon L. Risacher PhD[3,4], Kang-Hsien Fan PhD[1], Beth E. Snitz PhD[6,7], Howard J. Aizenstein PhD[8], Chester A. Mathis PhD[7,9], Oscar L. Lopez MD[6,7,8], F. Yesim Demirci MD[1], Eleanor Feingold PhD[1], William E. Klunk MD; PhD[6,7,8], Andrew J. Saykin PsyD[3,4] for the Alzheimer's Disease Neuroimaging Initiative (ADNI), Carlos Cruchaga PhD[5], M. Ilyas Kamboh PhD[1,7,8]

[1]Department of Human Genetics, University of Pittsburgh, Pittsburgh, PA USA

[2]Department of Pediatrics, Children's Hospital of Pittsburgh of UPMC, University of Pittsburgh, Pittsburgh, PA USA

[3]Department of Radiology and Imaging Sciences, Indiana University School of Medicine, Indianapolis, IN USA

[4]Indiana Alzheimer Disease Center, Indiana University School of Medicine, Indianapolis, IN USA

[5]Department of Psychiatry, Washington University School of Medicine, St. Louis, MO USA

[6]Department of Neurology, University of Pittsburgh, Pittsburgh, PA USA

[7]Alzheimer Disease Research Center, University of Pittsburgh, Pittsburgh, PA USA

[8]Department of Psychiatry, University of Pittsburgh, Pittsburgh, PA USA

[9]Department of Radiology, University of Pittsburgh, Pittsburgh, PA USA

Running title: GWAS of brain amyloidosis as measured by PiB-PET

Keywords: Brain amyloid, amyloid-PET, GWAS, meta-analysis

Address for correspondence:

M. Ilyas Kamboh, PhD

Department of Human Genetics, Graduate School of Public Health

University of Pittsburgh, PA 15261, USA

E-mail: kamboh@pitt.edu

Supplementary Text

Sample description

All subjects with PiB-PET data were European-Americans and derived from three sites: University of Pittsburgh (PITT), Washington University (WU) and Indiana University (IU) combined with the initial phase of the multicenter ADNI PiB-PET add-on study (here they are referred to as ADNI/IU). All subjects provided informed consent and all studies were approved by their local Institutional Review Boards. The summary statistics of these samples are included in Supplementary Table S1.

The PITT sample comprised a total of 361 subjects with age 36 to 97 years (mean age=78.3±10.1) and they were derived from the Ginkgo Evaluation of Memory Study (GEMS) imaging sub-study[1], healthy aging study[2] or the PITT-Alzheimer's Disease Research Center (ADRC). The WU sample consisted of 495 Subjects with age 37 to 88 years (mean age=65.8±9.8) and they were recruited as part of the WU-Knight ADRC in St. Louis.[3] A total of 127 subjects with age 55 to 88 years (mean age=73.8±7.6) were collected as part of the ADNI and the Indiana Memory and Aging Study.[4-7]

Genotyping, imputation and quality control

The genotyping platforms used for each study sample are listed in Supplementary Table S1. Imputation of non-genotyped single-nucleotide polymorphisms (SNPs) was performed with IMPUTE2[8] using the 1000 Genomes Project[9] Phase III (May 2013 release) data as the reference panel for PITT and Phase I (November 2010 release) data for WU and ADNI/IU datasets. In the PITT sample, SNPs with the imputation info score <0.5, the minor allele frequency (MAF) <0.01, P<1E-06 in the Hardy Weinberg equilibrium test and the missing rate >5% were removed as well as the insertions and deletions were excluded. After quality control measures, 361 subjects and ~7 million genotyped and imputed SNPs were included in the GWAS analysis. Genetic association analysis was conducted using linear regression under an additive genetic model,

adjusting for age, gender, diagnosis, and first four principal components (PCs) calculated using smartPCA.[10]

As part of quality control steps in the WU sample, SNPs with minor allele frequency <1%, call rates <98%, Hardy–Weinberg equilibrium *P*>1E-06 and individuals with >2% missing genotypes were removed from the analysis. The quality control SNP post imputation were as follows: MAF<1%, call rates <95%, Hardy–Weinberg equilibrium *P*>1E-06 and $r^2 \leq 0.3$. Pairwise genome-wide estimates of proportion identity-by-descent were used to find duplicate and related individuals which were eliminated from the analysis. 495 subjects and ~14 million SNPs were included in the association test. Association testing was conducted using linear regression under an additive genetic model, adjusting for age, gender, diagnosis, and first two PCs calculated using PLINK v1.9 (http://www.cog-genomics.org/plink2) to confirm ethnicity of each sample.

In the ADNI/IU sample, SNPs with the imputation info score ≤0.5, MAF<0.01, and *P*<1E-06 in the Hardy Weinberg equilibrium test were removed as well as the insertions and deletions. After performing the quality control, 127 subjects and ~8 million SNPs were used in the statistical analysis. Association analysis was performed using linear regression adjusted for age, gender, diagnosis and first three PCs.

Functional analyses

To evaluate the biological significance of PiB-associated signals, we conducted five different analyses: differential gene expression in AD versus non-AD in relevant tissues, brain gene expression, expression quantitative trait loci (eQTL) analyses, summary-data-based Mendelian randomization (SMR) analyses to test for pleiotropic association between gene expression and PiB, and pathway analyses.

Differentially expressed genes: We searched for differentially expressed genes from the target gene list using gene expression data from AlzBase.[11] AlzBase (http://alz.big.ac.cn/alzBase/)

includes transcription data from brain and blood from aging, non-dementia, mild cognitive impairment, early stage AD and late stage AD.

Human brain gene expression: We evaluated the expression level of all target genes in human brain tissues from the Barres Human and Mouse Brain RNA-Seq Resource[12, 13] (http://www.brainrnaseq.org/), and listed the corresponding cell types with expressed genes.

Expression quantitative trait loci (eQTL) analysis: To identify potential functional risk genes at each associated locus, we first identified variants in LD ($R^2 \geq 0.5$) with the top SNPs for the 16 associated loci in **Table 2**. The SNiPA website (https://snipa.helmholtz-muenchen.de/snipa3/) was used to search for variants in LD, using the 1000 Genomes, Phase 3v5 variant set for the European population. The list of variants was searched for genes functionally linked via eQTLs to our expanded list of variants. The GTEx[14] database was searched for eQTL associations in various brain tissues and whole blood.

Summary-data-based Mendelian randomization (SMR) analysis: To test if the genetic effect on PiB is mediated by gene expression in specific tissues, we performed SMR analyses to test for pleiotropic association between the expression level of a gene and PiB using summary-level data from GWAS and eQTL studies.[15]

Pathway analyses: Pathway analyses were performed with MAGMA,[16] which performs SNP-wise gene analysis of summary statistics with correction for LD between variants and genes to test whether sets of genes are jointly associated with a phenotype (i.e. PiB), compared to other genes across the genome. Adaptive permutation was used to produce an empirical p-value and FDR. Gene-sets used in the analyses were from GO,[17, 18] KEGG,[19, 20] REACTOME,[21, 22] and BIOCARTA pathways.

Data Availability

Upon publication, data will be submitted to the National Institute on Aging Genetics of Alzheimer's Disease (NIAGADS) data repository.

Association analysis of known non-*APOE* AD risk loci with amyloid burden

Although none of the known AD susceptibility loci, other than *APOE*, showed significant association at *P*<1E-05, we examined the top IGAP genome-wide significant SNPs (Supplementary Table S3.1) and the associated gene regions (Supplementary Table S3.2) in relation to amyloid burden. Only one IGAP variant, *HLA-DRB1,HLA-DQA1*/rs2760980; *P*=0.0093, was associated with global PiB retention at *P*<0.05. This is a proxy for the top *HLA-DRB1,HLA-DQA1*/rs9271192 SNP that was not present in our dataset. The regional analysis of these loci found nominally significance SNPs at *P*<0.05 in all but 4 loci (*CR1, PTK2B, PICALM, SORL1*) and 8 regions had top SNPs with *P*<0.01 (Supplementary Table S3.2).

Association of amyloid loci with AD risk

To test if the suggestive non-*APOE* amyloid loci listed in **Table 2** are also associated with AD risk, we examined their associations in our PITT-ADRC case-control sample of >2,200 subjects.[23] Two top amyloid-associated SNPs showed association with AD risk at *P*<0.05 (Supplementary Table S4.1**)**: one is located on chromosome 4 in *CYP4V2*/rs7377304 (*P*=0.017) and the other on chromosome 21 near *ICOSLG*/rs55708341 (*P*=0.0136). When we examined additional Aβ-associated SNPs having *P*<0.05 in each region with AD risk, multiple SNPs with *P*<0.05 were found in 13 of 15 loci and 10 of them showed odds ratios and beta-values in the same directions (Supplementary Table S4.2), indicating that our suggestive Aβ-associated loci are also associated with AD risk.

Supplementary Tables

Table S1. Summary of characteristics of participants from the University of Pittsburgh (PITT), Washington University (WU), and the Alzheimer's disease Neuroimaging Initiative (ADNI) and the Indiana Memory and Aging Study (ADNI/IU) included in the meta-analysis

	PITT (n=361)	WU (n=495)	ADNI/IU (n=127)
Age in years (mean ± standard deviation)	78.3±10.1	65.6±9.8	73.8±7.6
Male gender (n, %)	197(54.6)	205(41.4)	74(58.3)
PiB (mean ± standard deviation)	1.9±0.6*	0.2±0.3**	1.6±0.4*
Clinical diagnosis of probable Alzheimer disease (n, %)	43(11.9)	76(15.4)	32(25.2)
Genotyping platform	Illumina Omni Quad Chip; Omni2.5 Chip	Illumina 610; Omniexpress chip	Human610-Quad BeadChip; HumanOmni Express BeadChip

*Standardized Uptake Volume Ratio (SUVR)
**Binding Potential (BP)

Table S2. SNPs achieving genome-wide significance (*P*< 5E-08) in the meta-analysis

SNP	Chr	Position	A1	A2	Gene	Region	PITT			WU			ADNI/IU			Meta	
							MAF	Beta	*P*-value	MAF	Beta	*P*-value	MAF	Beta	*P*-value	Beta	*P*-value
rs429358	19	45411941	C	T	*APOE*	exonic	0.20	0.35	5.20E-12	0.21	0.16	5.36E-16	0.28	0.19	7.88E-05	0.18	**9.09E-30**
rs12721051	19	45422160	G	C	*APOC1*	intronic	0.22	0.31	9.04E-10	0.24	0.12	7.13E-11	0.30	0.14	1.55E-03	0.14	**2.60E-21**
rs4420638	19	45422946	G	A	*APOC1*	downstream	0.22	0.31	1.41E-09	0.25	0.11	1.34E-10	0.30	0.14	1.55E-03	0.14	**7.31E-21**
rs56131196	19	45422846	A	G	*APOC1*	downstream	0.22	0.31	1.34E-09	0.25	0.11	2.84E-10	0.30	0.14	1.55E-03	0.14	**1.45E-20**
rs6857	19	45392254	T	C	*PVRL2*	UTR3	0.23	0.29	8.77E-09	0.20	0.13	4.42E-11	0.27	0.16	1.71E-03	0.15	**1.70E-20**
rs10414043	19	45415713	A	G	*APOE,APOC1*	intergenic	0.16	0.33	3.57E-09	0.17	0.13	9.15E-11	0.22	0.16	2.67E-03	0.15	**2.24E-20**
rs769449	19	45410002	A	G	*APOE*	intronic	0.16	0.32	1.36E-08	0.15	0.13	5.90E-11	0.22	0.16	3.27E-03	0.15	**6.04E-20**
rs7256200	19	45415935	T	G	*APOE,APOC1*	intergenic	0.16	0.32	1.77E-08	0.17	0.13	2.88E-11	0.21	0.15	8.82E-03	0.15	**1.43E-19**
rs2075650	19	45395619	G	A	*TOMM40*	intronic	0.20	0.24	2.81E-06	0.17	0.12	9.31E-10	0.22	0.13	1.98E-02	0.13	**1.06E-15**
rs34404554	19	45395909	G	C	*TOMM40*	intronic	0.20	0.24	2.37E-06	0.16	0.11	2.10E-09	0.22	0.13	1.98E-02	0.13	**1.92E-15**
rs11556505	19	45396144	T	C	*TOMM40*	exonic	0.20	0.24	2.37E-06	0.17	0.11	2.23E-09	0.22	0.13	1.98E-02	0.13	**2.03E-15**
rs12721046	19	45421254	A	G	*APOC1*	intronic	0.19	0.26	1.24E-06	0.19	0.11	1.50E-08	0.24	0.11	2.57E-02	0.13	**1.01E-14**
rs438811	19	45416741	T	C	*APOE,APOC1*	intergenic	0.26	0.21	1.45E-05	0.30	0.09	7.89E-08	0.32	0.13	2.31E-03	0.11	**5.30E-14**
rs71352238	19	45394336	C	T	*TOMM40*	upstream	0.20	0.23	7.82E-06	0.16	0.11	2.22E-08	0.22	0.13	1.98E-02	0.12	**5.70E-14**
rs483082	19	45416178	T	G	*APOE,APOC1*	intergenic	0.26	0.21	1.45E-05	0.29	0.09	1.58E-07	0.32	0.13	2.31E-03	0.11	**1.02E-13**
rs5117	19	45418790	C	T	*APOC1*	intronic	0.26	0.22	7.03E-06	0.28	0.08	1.17E-06	0.32	0.14	1.77E-03	0.10	**2.65E-13**
rs59007384	19	45396665	T	G	*TOMM40*	intronic	0.26	0.21	1.20E-05	0.25	0.09	1.71E-07	0.30	0.12	9.68E-03	0.11	**3.31E-13**
rs34342646	19	45388130	A	G	*PVRL2*	intronic	0.21	0.23	8.92E-06	0.13	0.17	1.86E-07	0.23	0.11	3.47E-02	0.17	**2.05E-12**
rs12972156	19	45387459	G	C	*PVRL2*	intronic	0.19	0.23	1.51E-05	0.13	0.17	1.86E-07	0.23	0.11	3.47E-02	0.17	**3.52E-12**
rs12972970	19	45387596	A	G	*PVRL2*	intronic	0.20	0.23	1.58E-05	0.13	0.17	1.86E-07	0.23	0.11	3.47E-02	0.17	**3.71E-12**
rs157582	19	45396219	T	C	*TOMM40*	intronic	0.27	0.20	2.91E-05	0.28	0.09	9.19E-07	0.31	0.11	2.29E-02	0.10	**8.92E-12**
rs157581	19	45395714	C	T	*TOMM40*	exonic	0.27	0.20	3.60E-05	0.28	0.09	7.42E-07	0.31	0.11	2.29E-02	0.10	**9.19E-12**
rs283815	19	45390333	G	A	*PVRL2*	intronic	0.27	0.21	1.41E-05	0.23	0.15	8.86E-06	0.31	0.11	2.29E-02	0.15	**9.15E-11**
rs34095326	19	45395844	A	G	*TOMM40*	intronic	0.17	0.19	5.59E-04	0.11	0.12	9.67E-08	0.17	0.09	1.36E-01	0.12	**1.50E-10**
rs184017	19	45394969	G	T	*TOMM40*	intronic	0.27	0.20	2.55E-05	0.24	0.15	2.34E-05	0.31	0.11	2.29E-02	0.15	**3.43E-10**
rs10119	19	45406673	A	G	*TOMM40*	UTR3	0.32	0.14	3.39E-03	0.31	0.16	4.50E-07	0.37	0.14	9.84E-04	0.15	**4.31E-10**
rs75627662	19	45413576	T	C	*APOE*	downstream	0.23	0.14	3.49E-03	0.23	0.07	3.28E-05	0.26	0.10	2.34E-02	0.08	**3.58E-08**

A1: minor allele; A2: major allele.

Table S3.1. Association of top IGAP SNPs with PiB-PET

IGAP Locus/SNP	Chr	IGAP *P*-value	Meta PiB *P*-value
CR1/rs6656401	1	5.70E-24	0.7552
BIN1/rs10200967*	2	2.24E-35	0.5425
INPP5D/rs35349669	2	3.20E-08	0.05966
CASS4/rs7274581	5	2.50E-08	0.2891
MEF2C/rs190982	5	3.20E-08	0.9975
CD2AP/rs10948363	6	5.20E-11	0.7107
HLA-DRB5,HLA-DRB1/rs2760980*	6	4.03E-51	0.009257
EPHA1/rs11771145	7	1.10E-13	0.1628
NME8/rs2718058	7	4.80E-09	0.2263
SORL1/rs11218343	7	9.70E-15	0.1851
ZCWPW1/rs1476679	7	5.60E-10	0.5949
CLU/rs17057043*	8	2.13E-20	0.5606
PTK2B/rs28834970	8	7.40E-14	0.4002
CELF1/rs10838725	11	1.10E-08	0.07309
MS4A6A/rs983392	11	6.10E-16	0.8271
PICALM/rs10792832	11	9.30E-26	0.1104
FERMT2/rs17125944	14	7.90E-09	0.2323
SLC24A4,RIN3/rs10498633	14	5.50E-09	0.2475
ABCA7/rs4147929	19	1.10E-15	0.3858

*Proxy SNP for the top IGAP SNP

Table S3.2. Regional association of IGAP loci with PiB-PET

IGAP Locus	Start BP	End BP	Total SNPs in the region	# of SNPs <0.05	Top SNP in the region	Chr	Position	A1	A2	Beta	*P*-value
CR1	207669924	207814835	142	0	*CR1*/rs7542494	1	207809744	A	G	-0.04	0.0684
BIN1	127806605	127864546	189	5	*BIN1*/rs17014818	2	127810697	T	C	-0.03	0.0325
INPP5D	233743532	234115739	824	30	*INPP5D*/rs12052961	2	234076434	A	G	-0.04	0.0043
MEF2C	87963600	88199223	238	1	*MEF2C-AS1*/rs13159808	5	88194669	A	T	0.03	0.0290
HLA-DRB5/HLA-DRB1	32261252	32826450	3780	128	*HLA-DQB1*/rs28746853	6	32634646	C	T	0.04	0.0022
CD2AP	47445789	47688606	646	9	*GPR111*/rs7764134	6	47655330	A	T	0.03	0.0104
NME8	37780799	37939840	439	52	*NME8*/rs1823519	7	37915816	A	C	0.06	0.0065
ZCWPW1	99488543	100200674	794	10	*AZGP1P1*/rs10953293	7	99579032	G	A	-0.06	0.0073
EPHA1	143014244	143218545	408	4	*CLCN1,FAM131B*/rs7806322	7	143049965	T	C	-0.06	0.0188
PTK2B	27094615	27402132	652	0	*PTK2B*/rs35575787	8	27187520	T	G	-0.04	0.0547
CLU	27183710	27695123	1289	1	*EPHX2,CLU*/rs1532275	8	27424988	A	G	0.06	0.0496
CELF1	47186424	47868853	907	98	*DDB2*/rs2291120	11	47237680	C	T	0.12	0.0010
MS4A6A	59807796	60108278	631	13	*MS4A6A,MS4A4A*/rs11230208	11	60008547	A	C	0.06	0.0107
PICALM	85566157	85779310	486	0	*PICALM*/rs7951357	11	85762557	G	A	0.04	0.0552
SORL1	121328954	121502894	190	0	*SORL1*/rs17245976	11	121365720	T	G	-0.04	0.0944
FERMT2	53109716	53417538	427	78	*STYX*/rs10483618	14	53203590	C	T	0.08	0.0016
SLC24A4	92791068	93153606	1005	60	*SLC24A4*/rs72695119	14	92838693	A	G	0.09	0.0060
ABCA7	841398	1275987	680	44	*PRTN3*/rs629631	19	846041	T	C	0.11	0.0012
CASS4	54968038	55111371	241	2	*CASS4*/rs6127744	20	54986274	T	G	0.06	0.0278

Table S4.1. Association of top non-*APOE* PiB-PET variants with AD risk in a case-control maple of >2,200 subjects (Kamboh et al. *Mol Psychiatry* 2012; 2:e117)

Top PiB-PET Locus/SNP	Odds Ratio	*P*-value
ADCY8,EFR3A/rs13260032	1.050	0.4419
RAP2B,C3orf79/rs4680057	0.995	0.9258
DAPK2/rs12908891	1.118	0.07431
CYP4V2/rs7377304	0.862	0.01727
C21orf33,ICOSLG/rs55708341	1.206	0.01365
LINC00971/rs9831119	0.918	0.3492
SLITRK1/rs9531483	0.985	0.8269
C2orf80,IDH1/rs6722000	1.001	0.8912
MAGEF1,LOC101928992/rs11923588	0.924	0.5286
DTHD,MIR4801/rs66837203	1.204	0.1694
HTN1/rs200028958	0.907	0.3367
HSD17B6,SDR9C7/rs4526799	1.002	0.9728
ELTD1,LPHN2/rs17105538	1.176	0.06483
LINC01250/rs62121100	1.053	0.5476
KCNF,FLJ33534/rs1809136	0.865	0.2300

Table S4.2. Regional association of top non-*APOE* PiB-PET loci with AD risk in a case-control maple of >2,200 subjects (Kamboh et al. *Mol Psychiatry* 2012; 2:e117). For comparison PiB *P* and beta values are also given for the top corresponding AD risk SNP

Association of PiB-PET gene regions with AD risk													
PiB-PET Locus	Chr	Region Start	Region End	Total PiB SNPs	PiB SNPs <0.05	AD SNPs <0.05	Top AD Risk SNP in the region	A1 from PiB	AD risk Odds Ratio	*P* for AD Risk	Beta for PiB	*P* for PiB
ADCY8,EFR3A	8	131951455	132951455	2290	303	110	*ADCY8,EFR3A*/rs9693785	C	0.7272	0.0004175	-0.056907	0.007005
RAP2B,C3orf79	3	152596985	153596985	2377	359	11	*C3orf79,ARHGEF26-AS1*/rs7649472	G	1.156	0.02487	0.024879	0.04522
DAPK2	15	63736441	64736441	1626	165	11	*DAPK2*/rs1380843	A	0.8621	0.02232	-0.053325	0.005655
CYP4V2	4	186629780	187629780	2445	177	13	*CYP4V2*/rs1053094	A	1.1763	0.009745	0.058916	0.002189
C21orf33,ICOSLG	21	45127581	46127581	2199	227	73	*C21orf33,ICOSLG*/rs2070554	T	1.237	0.00879	0.037261	0.002774
LINC00971	3	84212077	85212077	2480	657	1	*CADM2*/rs13075478	T	1.152	0.02369	0.028393	0.02801
SLITRK1	13	83744873	84744873	1677	312	6	*SLITRK1*/rs9602135	G	0.7734	0.01895	0.042266	0.0297
C2orf80,IDH1	2	208575957	209575957	1562	188	0	*C2orf80,IDH1*/rs71418664	A	0.8554	0.06475	0.058783	0.00121
MAGEF1,LOC101928992	3	183959667	184959667	1774	72	1	*EIF4G1* /rs9846954	T	1.164	0.03531	-0.034909	0.04058
DTHD,MIR4801	4	36397136	37397136	3117	91	4	*DTHD1,MIR4801*/rs1319729	G	1.321	0.008938	0.039352	0.002278
HTN1	4	70423661	71423661	3151	465	42	*HTN1*/rs6854359	A	1.176	0.01454	-0.038333	0.00506
HSD17B6,SDR9C7	12	56780586	57780586	1132	95	0	*HSD17B6,SDR9C7*/rs1846400	A	0.9274	0.2208	-0.028111	0.0117
ELTD1,LPHN2	1	80815043	81815043	2130	159	11	*ELTD1,LPHN2*/rs10874177	C	1.2	0.008051	0.041764	0.002923
LINC01250	2	2593952	3593952	2434	267	7	*MYT1L,LINC01250*/rs12617695	T	1.447	0.002762	0.062219	0.04794
KCNF,FLJ33534	2	10652180	11652180	2394	209	10	*KCNF1,FLJ33534*/rs7556946	C	1.507	0.004408	-0.102457	0.04779

Table S5. The eQTL and SMR *P*-values for the top non-*APOE* region SNPs from Table 1

Tissue	Chr	Gene	Probe_bp	SNP	SNP_bp	A1	A2	Freq	p_GWAS	p_eQTL	p_SMR
Brain_Amygdala	8	*ADCY8*	131923609	rs13260032	132451455	C	A	0.43	4.87E-07	1.54E-02	3.27E-02
Brain_Frontal_Cortex_BA9	8	*ADCY8*	131923609	rs13260032	132451455	C	A	0.43	4.87E-07	4.75E-02	6.96E-02
Brain_Putamen_basal_ganglia	8	*ADCY8*	131923609	rs13260032	132451455	C	A	0.43	4.87E-07	2.86E-02	4.88E-02
Brain_Spinal_cord_cervical_c-1	8	*ADCY8*	131923609	rs13260032	132451455	C	A	0.43	4.87E-07	1.77E-02	3.57E-02
Brain_Anterior_cingulate_cortex_BA24	3	*RP11-38P22.2*	152558203	rs4680057	153096985	A	G	0.44	9.69E-07	2.43E-02	4.72E-02
Brain_Hippocampus	3	*RP11-38P22.2*	152558203	rs4680057	153096985	A	G	0.44	9.69E-07	1.58E-02	3.64E-02
Brain_Nucleus_accumbens_basal_ganglia	3	*RAP2B*	152883147	rs4680057	153096985	A	G	0.44	9.69E-07	2.00E-02	4.19E-02
Brain_Spinal_cord_cervical_c-1	3	*RP11-23D24.2*	153400349	rs4680057	153096985	A	G	0.44	9.69E-07	1.51E-02	3.54E-02
Whole_Blood	3	*ARHGEF26*	153907204	rs4680057	153096985	A	G	0.44	9.69E-07	1.31E-03	1.07E-02
Brain_Amygdala	15	*RAB8B*	63520824	rs12908891	64236441	A	G	0.52	1.39E-06	1.98E-02	3.51E-02
Brain_Anterior_cingulate_cortex_BA24	15	*HERC1*	64013479	rs12908891	64236441	A	G	0.52	1.39E-06	7.02E-05	1.94E-03
Brain_Anterior_cingulate_cortex_BA24	15	*DAPK2*	64281733	rs12908891	64236441	A	G	0.52	1.39E-06	1.40E-02	2.77E-02
Brain_Cerebellar_Hemisphere	15	*ANKDD1A*	65227571	rs12908891	64236441	A	G	0.52	1.39E-06	3.20E-02	4.90E-02
Brain_Cerebellum	15	*RP11-244F12.3*	63339909	rs12908891	64236441	A	G	0.52	1.39E-06	4.94E-02	6.77E-02
Brain_Cerebellum	15	*LACTB*	63424129	rs12908891	64236441	A	G	0.52	1.39E-06	2.10E-02	3.64E-02
Brain_Cerebellum	15	*CA12*	63643968	rs12908891	64236441	A	G	0.52	1.39E-06	2.44E-02	4.04E-02
Brain_Cerebellum	15	*USP3-AS1*	63864970	rs12908891	64236441	A	G	0.52	1.39E-06	4.85E-02	6.69E-02
Brain_Cerebellum	15	*DAPK2*	64281733	rs12908891	64236441	A	G	0.52	1.39E-06	1.95E-03	8.63E-03
Brain_Frontal_Cortex_BA9	15	*AC100830.3*	65002624	rs12908891	64236441	A	G	0.52	1.39E-06	1.67E-02	3.12E-02
Brain_Frontal_Cortex_BA9	15	*PLEKHO2*	65147147	rs12908891	64236441	A	G	0.52	1.39E-06	4.73E-02	6.56E-02
Brain_Hippocampus	15	*FBXL22*	63892089	rs12908891	64236441	A	G	0.52	1.39E-06	4.97E-02	6.81E-02
Brain_Hippocampus	15	*AC100830.4*	64987516	rs12908891	64236441	A	G	0.52	1.39E-06	4.44E-02	6.25E-02
Brain_Nucleus_accumbens_basal_ganglia	15	*RP11-244F12.3*	63339909	rs12908891	64236441	A	G	0.52	1.39E-06	3.96E-02	5.74E-02
Brain_Nucleus_accumbens_basal_ganglia	15	*USP3-AS1*	63864970	rs12908891	64236441	A	G	0.52	1.39E-06	7.63E-03	1.88E-02
Brain_Nucleus_accumbens_basal_ganglia	15	*SNX22*	64446798	rs12908891	64236441	A	G	0.52	1.39E-06	3.18E-02	4.89E-02
Brain_Putamen_basal_ganglia	15	*TPM1*	63349472	rs12908891	64236441	A	G	0.52	1.39E-06	3.55E-02	5.29E-02
Brain_Putamen_basal_ganglia	15	*SNX22*	64446798	rs12908891	64236441	A	G	0.52	1.39E-06	4.62E-03	1.39E-02
Brain_Putamen_basal_ganglia	15	*TRIP4*	64713724	rs12908891	64236441	A	G	0.52	1.39E-06	2.45E-02	4.06E-02
Brain_Putamen_basal_ganglia	15	*RBPMS2*	65049938	rs12908891	64236441	A	G	0.52	1.39E-06	4.15E-02	5.94E-02
Brain_Spinal_cord_cervical_c-1	15	*RAB8B*	63520824	rs12908891	64236441	A	G	0.52	1.39E-06	4.81E-02	6.64E-02
Brain_Spinal_cord_cervical_c-1	15	*FBXL22*	63892089	rs12908891	64236441	A	G	0.52	1.39E-06	1.29E-02	2.63E-02
Brain_Spinal_cord_cervical_c-1	15	*TRIP4*	64713724	rs12908891	64236441	A	G	0.52	1.39E-06	4.60E-02	6.42E-02
Brain_Substantia_nigra	15	*FBXL22*	63892089	rs12908891	64236441	A	G	0.52	1.39E-06	2.05E-02	3.58E-02
Whole_Blood	15	*TPM1*	63349472	rs12908891	64236441	A	G	0.52	1.39E-06	2.66E-02	4.30E-02
Whole_Blood	15	*KIAA0101*	64668539	rs12908891	64236441	A	G	0.52	1.39E-06	1.86E-02	3.35E-02
Brain_Amygdala	4	*KIAA1430*	186105738	rs7377304	187129780	G	T	0.45	2.46E-06	2.21E-02	3.88E-02
Brain_Anterior_cingulate_cortex_BA24	4	*SORBS2*	186692202	rs7377304	187129780	G	T	0.45	2.46E-06	3.88E-02	5.77E-02
Brain_Anterior_cingulate_cortex_BA24	4	*FAM149A*	187059697	rs7377304	187129780	G	T	0.45	2.46E-06	1.35E-02	2.81E-02
Brain_Anterior_cingulate_cortex_BA24	4	*F11*	187198967	rs7377304	187129780	G	T	0.45	2.46E-06	8.33E-03	2.08E-02
Brain_Anterior_cingulate_cortex_BA24	4	*MTNR1A*	187465765	rs7377304	187129780	G	T	0.45	2.46E-06	1.51E-02	3.02E-02
Brain_Caudate_basal_ganglia	4	*CYP4V2*	187123642	rs7377304	187129780	G	T	0.45	2.46E-06	1.48E-02	2.97E-02
Brain_Caudate_basal_ganglia	4	*F11*	187198967	rs7377304	187129780	G	T	0.45	2.46E-06	2.71E-02	4.47E-02
Brain_Cerebellar_Hemisphere	4	*C4orf47*	186359187	rs7377304	187129780	G	T	0.45	2.46E-06	2.02E-02	3.66E-02
Brain_Cerebellum	4	*UFSP2*	186333916	rs7377304	187129780	G	T	0.45	2.46E-06	4.55E-02	6.48E-02
Brain_Cerebellum	4	*PDLIM3*	186439808	rs7377304	187129780	G	T	0.45	2.46E-06	7.07E-03	1.88E-02
Brain_Cerebellum	4	*ORAOV1P1*	187092214	rs7377304	187129780	G	T	0.45	2.46E-06	2.97E-02	4.77E-02
Brain_Cortex	4	*F11*	187198967	rs7377304	187129780	G	T	0.45	2.46E-06	1.10E-05	1.19E-03
Brain_Frontal_Cortex_BA9	4	*RP11-714G18.1*	186302025	rs7377304	187129780	G	T	0.45	2.46E-06	2.27E-02	3.96E-02
Brain_Frontal_Cortex_BA9	4	*F11*	187198967	rs7377304	187129780	G	T	0.45	2.46E-06	4.91E-02	6.87E-02
Brain_Hippocampus	4	*F11*	187198967	rs7377304	187129780	G	T	0.45	2.46E-06	2.85E-04	3.80E-03
Brain_Nucleus_accumbens_basal_ganglia	4	*CYP4V2*	187123642	rs7377304	187129780	G	T	0.45	2.46E-06	2.81E-02	4.58E-02
Brain_Putamen_basal_ganglia	4	*F11*	187198967	rs7377304	187129780	G	T	0.45	2.46E-06	5.04E-03	1.55E-02
Brain_Putamen_basal_ganglia	4	*FAT1*	187578406	rs7377304	187129780	G	T	0.45	2.46E-06	1.06E-02	2.40E-02
Brain_Spinal_cord_cervical_c-1	4	*TLR3*	186999764	rs7377304	187129780	G	T	0.45	2.46E-06	4.35E-02	6.27E-02
Brain_Substantia_nigra	4	*F11*	187198967	rs7377304	187129780	G	T	0.45	2.46E-06	1.19E-02	2.59E-02
Whole_Blood	4	*UFSP2*	186333916	rs7377304	187129780	G	T	0.45	2.46E-06	3.65E-04	4.22E-03
Brain_Amygdala	21	*AP001053.11*	45229043	rs55708341	45627581	T	A	0.2	2.51E-06	4.54E-02	7.10E-02
Brain_Anterior_cingulate_cortex_BA24	21	*CSTB*	45194359	rs55708341	45627581	T	A	0.2	2.51E-06	3.97E-02	6.49E-02
Brain_Anterior_cingulate_cortex_BA24	21	*RRP1*	45217284	rs55708341	45627581	T	A	0.2	2.51E-06	8.64E-03	2.61E-02
Brain_Caudate_basal_ganglia	21	*TRAPPC10*	45479316	rs55708341	45627581	T	A	0.2	2.51E-06	2.90E-02	5.29E-02
Brain_Caudate_basal_ganglia	21	*UBE2G2*	46205444	rs55708341	45627581	T	A	0.2	2.51E-06	3.66E-02	6.15E-02
Brain_Cerebellar_Hemisphere	21	*RRP1B*	45097693	rs55708341	45627581	T	A	0.2	2.51E-06	2.72E-02	5.08E-02
Brain_Cerebellar_Hemisphere	21	*CSTB*	45194359	rs55708341	45627581	T	A	0.2	2.51E-06	4.25E-02	6.79E-02
Brain_Cerebellar_Hemisphere	21	*AP001055.6*	45579291	rs55708341	45627581	T	A	0.2	2.51E-06	1.11E-02	2.99E-02
Brain_Cerebellar_Hemisphere	21	*AP001058.3*	45626845	rs55708341	45627581	T	A	0.2	2.51E-06	3.48E-02	5.95E-02
Brain_Cerebellum	21	*C21orf67*	46356278	rs55708341	45627581	T	A	0.2	2.51E-06	2.07E-02	4.29E-02
Brain_Cortex	21	*LL21NC02-1C16.1*	46349810	rs55708341	45627581	T	A	0.2	2.51E-06	7.94E-03	2.50E-02
Brain_Frontal_Cortex_BA9	21	*LINC00313*	44890694	rs55708341	45627581	T	A	0.2	2.51E-06	4.70E-02	7.27E-02
Brain_Frontal_Cortex_BA9	21	*HSF2BP*	45014223	rs55708341	45627581	T	A	0.2	2.51E-06	4.84E-02	7.42E-02

Brain_Frontal_Cortex_BA9	21	*CSTB*	45194359	rs55708341	45627581	T	A	0.2	2.51E-06	5.21E-03	2.02E-02
Brain_Frontal_Cortex_BA9	21	*AP001058.3*	45626845	rs55708341	45627581	T	A	0.2	2.51E-06	6.86E-04	8.38E-03
Brain_Hippocampus	21	*LL21NC02-1C16.2*	46356543	rs55708341	45627581	T	A	0.2	2.51E-06	2.38E-02	4.67E-02
Brain_Hypothalamus	21	*HSF2BP*	45014223	rs55708341	45627581	T	A	0.2	2.51E-06	3.71E-02	6.20E-02
Brain_Hypothalamus	21	*CSTB*	45194359	rs55708341	45627581	T	A	0.2	2.51E-06	1.83E-02	3.98E-02
Brain_Hypothalamus	21	*AP001065.7*	45891105	rs55708341	45627581	T	A	0.2	2.51E-06	3.73E-02	6.23E-02
Brain_Nucleus_accumbens_basal_ganglia	21	*AP001058.3*	45626845	rs55708341	45627581	T	A	0.2	2.51E-06	4.65E-02	7.22E-02
Brain_Nucleus_accumbens_basal_ganglia	21	*ICOSLG*	45651861	rs55708341	45627581	T	A	0.2	2.51E-06	4.79E-02	7.37E-02
Brain_Nucleus_accumbens_basal_ganglia	21	*LRRC3*	45877054	rs55708341	45627581	T	A	0.2	2.51E-06	3.77E-02	6.27E-02
Brain_Nucleus_accumbens_basal_ganglia	21	*C21orf90*	45941467	rs55708341	45627581	T	A	0.2	2.51E-06	1.11E-02	2.99E-02
Brain_Putamen_basal_ganglia	21	*SUMO3*	46232113	rs55708341	45627581	T	A	0.2	2.51E-06	3.26E-02	5.71E-02
Brain_Substantia_nigra	21	*AP001056.1*	45595854	rs55708341	45627581	T	A	0.2	2.51E-06	4.07E-02	6.60E-02
Whole_Blood	21	*RRP1B*	45097693	rs55708341	45627581	T	A	0.2	2.51E-06	4.30E-02	6.84E-02
Brain_Amygdala	3	*CADM2*	85565855	rs9831119	84712077	C	T	0.13	2.98E-06	1.50E-03	9.29E-03
Brain_Cortex	3	*CADM2*	85565855	rs9831119	84712077	C	T	0.13	2.98E-06	2.43E-02	4.37E-02
Brain_Anterior_cingulate_cortex_BA24	2	*AC096772.6*	208686753	rs6722000	209075957	G	A	0.21	4.96E-06	7.00E-03	2.15E-02
Brain_Caudate_basal_ganglia	2	*CREB1*	208431337	rs6722000	209075957	G	A	0.21	4.96E-06	2.49E-02	4.57E-02
Brain_Cerebellum	2	*C2orf80*	209042432	rs6722000	209075957	G	A	0.21	4.96E-06	6.70E-03	2.10E-02
Brain_Cerebellum	2	*PIKFYVE*	209177233	rs6722000	209075957	G	A	0.21	4.96E-06	2.27E-02	4.31E-02
Brain_Cortex	2	*IDH1-AS1*	209120438	rs6722000	209075957	G	A	0.21	4.96E-06	4.81E-03	1.76E-02
Brain_Hippocampus	2	*IDH1-AS1*	209120438	rs6722000	209075957	G	A	0.21	4.96E-06	1.65E-02	3.54E-02
Brain_Hypothalamus	2	*PTH2R*	209471832	rs6722000	209075957	G	A	0.21	4.96E-06	3.53E-02	5.77E-02
Brain_Substantia_nigra	2	*CCNYL1*	208601413	rs6722000	209075957	G	A	0.21	4.96E-06	4.92E-02	7.26E-02
Brain_Substantia_nigra	2	*CRYGEP*	208975096	rs6722000	209075957	G	A	0.21	4.96E-06	4.90E-02	7.24E-02
Whole_Blood	2	*AC007879.2*	208076115	rs6722000	209075957	G	A	0.21	4.96E-06	4.39E-02	6.70E-02
Whole_Blood	2	*PIKFYVE*	209177233	rs6722000	209075957	G	A	0.21	4.96E-06	1.08E-02	2.75E-02
Brain_Amygdala	3	*YEATS2-AS1*	183526253	rs11923588	184459667	T	C	0.06	5.66E-06	7.92E-03	2.02E-02
Brain_Amygdala	3	*PARL*	183574934	rs11923588	184459667	T	C	0.06	5.66E-06	1.56E-02	3.08E-02
Brain_Amygdala	3	*ABCC5*	183686762	rs11923588	184459667	T	C	0.06	5.66E-06	3.63E-03	1.29E-02
Brain_Amygdala	3	*CAMK2N2*	183978312	rs11923588	184459667	T	C	0.06	5.66E-06	2.62E-02	4.37E-02
Brain_Amygdala	3	*VPS8*	184650166	rs11923588	184459667	T	C	0.06	5.66E-06	2.94E-02	4.74E-02
Brain_Anterior_cingulate_cortex_BA24	3	*YEATS2-AS1*	183526253	rs11923588	184459667	T	C	0.06	5.66E-06	1.68E-02	3.23E-02
Brain_Anterior_cingulate_cortex_BA24	3	*ABCC5*	183686762	rs11923588	184459667	T	C	0.06	5.66E-06	8.10E-03	2.05E-02
Brain_Anterior_cingulate_cortex_BA24	3	*VPS8*	184650166	rs11923588	184459667	T	C	0.06	5.66E-06	1.96E-02	3.59E-02
Brain_Caudate_basal_ganglia	3	*YEATS2-AS1*	183526253	rs11923588	184459667	T	C	0.06	5.66E-06	4.74E-02	6.69E-02
Brain_Caudate_basal_ganglia	3	*FAM131A*	184058843	rs11923588	184459667	T	C	0.06	5.66E-06	3.06E-02	4.87E-02
Brain_Caudate_basal_ganglia	3	*SENP2*	185325811	rs11923588	184459667	T	C	0.06	5.66E-06	1.91E-02	3.53E-02
Brain_Cerebellar_Hemisphere	3	*HSP90AA5P*	183834413	rs11923588	184459667	T	C	0.06	5.66E-06	2.43E-02	4.14E-02
Brain_Cerebellar_Hemisphere	3	*RP11-329B9.5*	184460839	rs11923588	184459667	T	C	0.06	5.66E-06	1.48E-02	2.98E-02
Brain_Cerebellum	3	*CAMK2N2*	183978312	rs11923588	184459667	T	C	0.06	5.66E-06	1.98E-02	3.61E-02
Brain_Hippocampus	3	*RP11-329B9.4*	184454300	rs11923588	184459667	T	C	0.06	5.66E-06	3.38E-02	5.23E-02
Brain_Nucleus_accumbens_basal_ganglia	3	*AP2M1*	183897178	rs11923588	184459667	T	C	0.06	5.66E-06	3.05E-02	4.86E-02
Brain_Putamen_basal_ganglia	3	*RP11-778D9.12*	183852416	rs11923588	184459667	T	C	0.06	5.66E-06	4.80E-02	6.75E-02
Brain_Putamen_basal_ganglia	3	*EPHB3*	184289884	rs11923588	184459667	T	C	0.06	5.66E-06	4.75E-02	6.70E-02
Brain_Spinal_cord_cervical_c-1	3	*CAMK2N2*	183978312	rs11923588	184459667	T	C	0.06	5.66E-06	4.73E-02	6.68E-02
Brain_Substantia_nigra	3	*CAMK2N2*	183978312	rs11923588	184459667	T	C	0.06	5.66E-06	4.15E-02	6.07E-02
Brain_Substantia_nigra	3	*LIPH*	185247225	rs11923588	184459667	T	C	0.06	5.66E-06	3.57E-02	5.43E-02
Whole_Blood	3	*CHRD*	184102739	rs11923588	184459667	T	C	0.06	5.66E-06	3.51E-02	5.37E-02
Brain_Cerebellum	4	*DTHD1*	36315311	rs66837203	36897136	T	C	0.07	6.03E-06	1.20E-02	2.98E-02
Brain_Hypothalamus	4	*ARAP2*	36097987	rs66837203	36897136	T	C	0.07	6.03E-06	2.68E-03	1.36E-02
Brain_Putamen_basal_ganglia	4	*ARAP2*	36097987	rs66837203	36897136	T	C	0.07	6.03E-06	4.88E-02	7.29E-02
Brain_Putamen_basal_ganglia	4	*RP11-431M7.3*	36260790	rs66837203	36897136	T	C	0.07	6.03E-06	1.57E-02	3.49E-02
Brain_Substantia_nigra	4	*RP11-431M7.3*	36260790	rs66837203	36897136	T	C	0.07	6.03E-06	2.53E-02	4.69E-02
Brain_Cerebellar_Hemisphere	4	*RP11-46J23.1*	71569836	rs200028958	70923661	A	G	0.1	6.25E-06	2.90E-02	5.35E-02
Brain_Hypothalamus	4	*UTP3*	71555231	rs200028958	70923661	A	G	0.1	6.25E-06	1.41E-02	3.49E-02
Brain_Hypothalamus	4	*RUFY3*	71621775	rs200028958	70923661	A	G	0.1	6.25E-06	2.78E-02	5.21E-02
Brain_Hypothalamus	4	*DCK*	71877443	rs200028958	70923661	A	G	0.1	6.25E-06	1.25E-02	3.26E-02
Whole_Blood	4	*GRSF1*	71693580	rs200028958	70923661	A	G	0.1	6.25E-06	6.40E-03	2.29E-02
Brain_Amygdala	12	*ERBB3*	56485465	rs4526799	57280586	T	C	0.34	7.26E-06	3.43E-02	6.91E-02
Brain_Amygdala	12	*ATP5B*	57035878	rs4526799	57280586	T	C	0.34	7.26E-06	1.95E-02	5.10E-02
Brain_Amygdala	12	*PRIM1*	57135785	rs4526799	57280586	T	C	0.34	7.26E-06	4.71E-03	2.70E-02
Brain_Amygdala	12	*AC025165.8*	58008874	rs4526799	57280586	T	C	0.34	7.26E-06	2.87E-02	6.25E-02
Brain_Amygdala	12	*TSPAN31*	58138756	rs4526799	57280586	T	C	0.34	7.26E-06	4.57E-02	8.16E-02
Brain_Amygdala	12	*AVIL*	58201929	rs4526799	57280586	T	C	0.34	7.26E-06	2.48E-03	2.13E-02
Brain_Anterior_cingulate_cortex_BA24	12	*MYL6*	56554355	rs4526799	57280586	T	C	0.34	7.26E-06	2.10E-02	5.30E-02
Brain_Anterior_cingulate_cortex_BA24	12	*CNPY2*	56706816	rs4526799	57280586	T	C	0.34	7.26E-06	4.40E-02	7.98E-02
Brain_Anterior_cingulate_cortex_BA24	12	*BAZ2A*	57009990	rs4526799	57280586	T	C	0.34	7.26E-06	2.80E-02	6.17E-02
Brain_Anterior_cingulate_cortex_BA24	12	*TMEM194A*	57465636	rs4526799	57280586	T	C	0.34	7.26E-06	9.25E-04	1.54E-02
Brain_Anterior_cingulate_cortex_BA24	12	*INHBE*	57849584	rs4526799	57280586	T	C	0.34	7.26E-06	1.93E-03	1.95E-02
Brain_Anterior_cingulate_cortex_BA24	12	*RP11-571M6.17*	58197909	rs4526799	57280586	T	C	0.34	7.26E-06	4.87E-02	8.48E-02
Brain_Anterior_cingulate_cortex_BA24	12	*AVIL*	58201929	rs4526799	57280586	T	C	0.34	7.26E-06	1.68E-02	4.73E-02
Brain_Caudate_basal_ganglia	12	*PA2G4*	56502897	rs4526799	57280586	T	C	0.34	7.26E-06	1.56E-02	4.56E-02

Brain_Caudate_basal_ganglia	12	*COQ10A*	56662696	rs4526799	57280586	T	C	0.34	7.26E-06	2.82E-02	6.19E-02
Brain_Caudate_basal_ganglia	12	*IL23A*	56733428	rs4526799	57280586	T	C	0.34	7.26E-06	1.34E-02	4.24E-02
Brain_Caudate_basal_ganglia	12	*RP11-74M13.4*	57242410	rs4526799	57280586	T	C	0.34	7.26E-06	1.97E-02	5.12E-02
Brain_Caudate_basal_ganglia	12	*MYO1A*	57433641	rs4526799	57280586	T	C	0.34	7.26E-06	1.86E-02	4.97E-02
Brain_Cerebellar_Hemisphere	12	*PMEL*	56357495	rs4526799	57280586	T	C	0.34	7.26E-06	9.90E-03	3.69E-02
Brain_Cerebellar_Hemisphere	12	*RP11-603J24.7*	56374517	rs4526799	57280586	T	C	0.34	7.26E-06	9.33E-03	3.59E-02
Brain_Cerebellar_Hemisphere	12	*MYL6*	56554355	rs4526799	57280586	T	C	0.34	7.26E-06	1.40E-02	4.33E-02
Brain_Cerebellar_Hemisphere	12	*METTL21B*	58170856	rs4526799	57280586	T	C	0.34	7.26E-06	4.24E-02	7.81E-02
Brain_Cerebellum	12	*RPS26*	56436876	rs4526799	57280586	T	C	0.34	7.26E-06	2.88E-02	6.27E-02
Brain_Cerebellum	12	*CS*	56679829	rs4526799	57280586	T	C	0.34	7.26E-06	3.16E-02	6.59E-02
Brain_Cerebellum	12	*MIP*	56853054	rs4526799	57280586	T	C	0.34	7.26E-06	4.42E-02	8.00E-02
Brain_Cerebellum	12	*PTGES3*	57069643	rs4526799	57280586	T	C	0.34	7.26E-06	3.11E-02	6.53E-02
Brain_Cerebellum	12	*RDH16*	57349188	rs4526799	57280586	T	C	0.34	7.26E-06	2.13E-02	5.34E-02
Brain_Cortex	12	*WIBG*	56310770	rs4526799	57280586	T	C	0.34	7.26E-06	3.72E-02	7.24E-02
Brain_Cortex	12	*PMEL*	56357495	rs4526799	57280586	T	C	0.34	7.26E-06	2.36E-03	2.09E-02
Brain_Cortex	12	*STAC3*	57641106	rs4526799	57280586	T	C	0.34	7.26E-06	4.54E-02	8.12E-02
Brain_Frontal_Cortex_BA9	12	*IKZF4*	56416831	rs4526799	57280586	T	C	0.34	7.26E-06	2.60E-02	5.92E-02
Brain_Frontal_Cortex_BA9	12	*COQ10A*	56662696	rs4526799	57280586	T	C	0.34	7.26E-06	4.08E-02	7.63E-02
Brain_Frontal_Cortex_BA9	12	*RP11-977G19.11*	56701259	rs4526799	57280586	T	C	0.34	7.26E-06	1.96E-02	5.11E-02
Brain_Hippocampus	12	*RAB5B*	56378093	rs4526799	57280586	T	C	0.34	7.26E-06	2.48E-02	5.78E-02
Brain_Hippocampus	12	*RNF41*	56607001	rs4526799	57280586	T	C	0.34	7.26E-06	2.32E-02	5.58E-02
Brain_Hippocampus	12	*ANKRD52*	56641903	rs4526799	57280586	T	C	0.34	7.26E-06	3.06E-02	6.47E-02
Brain_Hippocampus	12	*ZBTB39*	57396424	rs4526799	57280586	T	C	0.34	7.26E-06	4.04E-02	7.58E-02
Brain_Hippocampus	12	*METTL1*	58164318	rs4526799	57280586	T	C	0.34	7.26E-06	8.61E-03	3.47E-02
Brain_Hypothalamus	12	*PTGES3*	57069643	rs4526799	57280586	T	C	0.34	7.26E-06	1.05E-02	3.79E-02
Brain_Nucleus_accumbens_basal_ganglia	12	*RNF41*	56607001	rs4526799	57280586	T	C	0.34	7.26E-06	4.91E-02	8.53E-02
Brain_Nucleus_accumbens_basal_ganglia	12	*BAZ2A*	57009990	rs4526799	57280586	T	C	0.34	7.26E-06	3.20E-02	6.64E-02
Brain_Putamen_basal_ganglia	12	*WIBG*	56310770	rs4526799	57280586	T	C	0.34	7.26E-06	3.45E-02	6.93E-02
Brain_Putamen_basal_ganglia	12	*CDK2*	56363560	rs4526799	57280586	T	C	0.34	7.26E-06	1.90E-02	5.03E-02
Brain_Putamen_basal_ganglia	12	*RPS26*	56436876	rs4526799	57280586	T	C	0.34	7.26E-06	1.38E-02	4.30E-02
Brain_Putamen_basal_ganglia	12	*MYL6B*	56548905	rs4526799	57280586	T	C	0.34	7.26E-06	1.59E-02	4.60E-02
Brain_Putamen_basal_ganglia	12	*NABP2*	56619718	rs4526799	57280586	T	C	0.34	7.26E-06	3.66E-02	7.16E-02
Brain_Putamen_basal_ganglia	12	*CS*	56679829	rs4526799	57280586	T	C	0.34	7.26E-06	3.12E-02	6.55E-02
Brain_Putamen_basal_ganglia	12	*RP11-977G19.11*	56701259	rs4526799	57280586	T	C	0.34	7.26E-06	3.55E-02	7.04E-02
Brain_Putamen_basal_ganglia	12	*IL23A*	56733428	rs4526799	57280586	T	C	0.34	7.26E-06	1.97E-02	5.13E-02
Brain_Putamen_basal_ganglia	12	*NAB2*	57485933	rs4526799	57280586	T	C	0.34	7.26E-06	7.23E-03	3.22E-02
Brain_Putamen_basal_ganglia	12	*INHBE*	57849584	rs4526799	57280586	T	C	0.34	7.26E-06	3.79E-02	7.31E-02
Brain_Putamen_basal_ganglia	12	*AVIL*	58201929	rs4526799	57280586	T	C	0.34	7.26E-06	2.01E-02	5.18E-02
Brain_Spinal_cord_cervical_c-1	12	*ESYT1*	56530147	rs4526799	57280586	T	C	0.34	7.26E-06	4.85E-02	8.46E-02
Brain_Spinal_cord_cervical_c-1	12	*TAC3*	57413225	rs4526799	57280586	T	C	0.34	7.26E-06	3.04E-02	6.46E-02
Brain_Substantia_nigra	12	*RP11-603J24.7*	56374517	rs4526799	57280586	T	C	0.34	7.26E-06	3.56E-02	7.06E-02
Brain_Substantia_nigra	12	*MYL6*	56554355	rs4526799	57280586	T	C	0.34	7.26E-06	3.69E-02	7.20E-02
Brain_Substantia_nigra	12	*RP11-153M3.1*	56905636	rs4526799	57280586	T	C	0.34	7.26E-06	4.31E-02	7.88E-02
Brain_Substantia_nigra	12	*CYP27B1*	58158578	rs4526799	57280586	T	C	0.34	7.26E-06	4.56E-02	8.15E-02
Whole_Blood	12	*RP11-977G19.11*	56701259	rs4526799	57280586	T	C	0.34	7.26E-06	1.21E-02	4.04E-02
Whole_Blood	12	*STAT2*	56744645	rs4526799	57280586	T	C	0.34	7.26E-06	3.25E-02	6.69E-02
Whole_Blood	12	*GLS2*	56873481	rs4526799	57280586	T	C	0.34	7.26E-06	1.06E-02	3.80E-02
Whole_Blood	12	*NAB2*	57485933	rs4526799	57280586	T	C	0.34	7.26E-06	7.08E-03	3.19E-02
Whole_Blood	12	*KIF5A*	57962098	rs4526799	57280586	T	C	0.34	7.26E-06	5.09E-03	2.79E-02
Whole_Blood	12	*RP11-571M6.8*	58118261	rs4526799	57280586	T	C	0.34	7.26E-06	4.56E-02	8.15E-02
Whole_Blood	12	*AGAP2*	58127694	rs4526799	57280586	T	C	0.34	7.26E-06	1.48E-02	4.45E-02
Brain_Amygdala	1	*MED28P8*	82023161	rs17105538	81315043	G	A	0.15	7.66E-06	2.23E-02	4.19E-02
Brain_Hippocampus	1	*LPHN2*	82114982	rs17105538	81315043	G	A	0.15	7.66E-06	1.48E-02	3.23E-02
Brain_Substantia_nigra	1	*LPHN2*	82114982	rs17105538	81315043	G	A	0.15	7.66E-06	3.79E-02	5.98E-02
Brain_Amygdala	2	*RNASEH1*	3599232	rs62121100	3093952	G	T	0.18	8.44E-06	1.76E-02	4.34E-02
Brain_Cerebellar_Hemisphere	2	*RNASEH1*	3599232	rs62121100	3093952	G	T	0.18	8.44E-06	4.98E-02	8.07E-02
Brain_Cerebellar_Hemisphere	2	*ALLC*	3727979	rs62121100	3093952	G	T	0.18	8.44E-06	4.90E-02	7.98E-02
Brain_Cerebellum	2	*RP11-1293J14.1*	3500929	rs62121100	3093952	G	T	0.18	8.44E-06	8.66E-03	3.03E-02
Brain_Cortex	2	*TSSC1*	3287174	rs62121100	3093952	G	T	0.18	8.44E-06	3.99E-02	7.00E-02
Brain_Cortex	2	*RPS7*	3625652	rs62121100	3093952	G	T	0.18	8.44E-06	7.25E-03	2.78E-02
Brain_Frontal_Cortex_BA9	2	*TRAPPC12*	3436155	rs62121100	3093952	G	T	0.18	8.44E-06	2.53E-02	5.32E-02
Brain_Hippocampus	2	*MYT1L*	2063958	rs62121100	3093952	G	T	0.18	8.44E-06	3.86E-02	6.86E-02
Brain_Hippocampus	2	*RP11-1293J14.1*	3500929	rs62121100	3093952	G	T	0.18	8.44E-06	1.11E-02	3.42E-02
Brain_Nucleus_accumbens_basal_ganglia	2	*TSSC1-IT1*	3303674	rs62121100	3093952	G	T	0.18	8.44E-06	1.98E-02	4.64E-02
Brain_Nucleus_accumbens_basal_ganglia	2	*RP11-1293J14.1*	3500929	rs62121100	3093952	G	T	0.18	8.44E-06	4.22E-02	7.26E-02
Brain_Nucleus_accumbens_basal_ganglia	2	*AC142528.1*	3525007	rs62121100	3093952	G	T	0.18	8.44E-06	3.56E-02	6.53E-02
Brain_Spinal_cord_cervical_c-1	2	*AC142528.1*	3525007	rs62121100	3093952	G	T	0.18	8.44E-06	3.75E-02	6.74E-02
Brain_Substantia_nigra	2	*TMSB4XP2*	3665242	rs62121100	3093952	G	T	0.18	8.44E-06	2.51E-02	5.30E-02

Brain_Amygdala	2	*NOL10*	10770496	rs1809136	11152180	C	G	0.93	9.99E-06	1.71E-02	3.85E-02
Brain_Amygdala	2	*RP11-791G15.2*	10909092	rs1809136	11152180	C	G	0.93	9.99E-06	2.43E-02	4.75E-02
Brain_Amygdala	2	*PDIA6*	10952755	rs1809136	11152180	C	G	0.93	9.99E-06	4.29E-02	6.86E-02
Brain_Amygdala	2	*E2F6*	11595399	rs1809136	11152180	C	G	0.93	9.99E-06	6.29E-04	8.21E-03
Brain_Amygdala	2	*GREB1*	11728578	rs1809136	11152180	C	G	0.93	9.99E-06	1.31E-02	3.31E-02
Brain_Caudate_basal_ganglia	2	*RN7SL832P*	10831347	rs1809136	11152180	C	G	0.93	9.99E-06	1.66E-02	3.78E-02
Brain_Cerebellar_Hemisphere	2	*RP11-254F7.2*	10180004	rs1809136	11152180	C	G	0.93	9.99E-06	3.36E-03	1.65E-02
Brain_Cerebellar_Hemisphere	2	*C2orf50*	11280047	rs1809136	11152180	C	G	0.93	9.99E-06	1.71E-02	3.85E-02
Brain_Cortex	2	*AC007249.3*	10594061	rs1809136	11152180	C	G	0.93	9.99E-06	3.75E-02	6.27E-02
Brain_Cortex	2	*ROCK2*	11404171	rs1809136	11152180	C	G	0.93	9.99E-06	8.31E-03	2.58E-02
Brain_Frontal_Cortex_BA9	2	*ATP6V1C2*	10893505	rs1809136	11152180	C	G	0.93	9.99E-06	1.64E-02	3.75E-02
Brain_Hippocampus	2	*CYS1*	10208989	rs1809136	11152180	C	G	0.93	9.99E-06	2.82E-02	5.21E-02
Brain_Hippocampus	2	*ROCK2*	11404171	rs1809136	11152180	C	G	0.93	9.99E-06	3.30E-02	5.77E-02
Brain_Hippocampus	2	*NTSR2*	11804297	rs1809136	11152180	C	G	0.93	9.99E-06	2.92E-03	1.54E-02
Brain_Hypothalamus	2	*ATP6V1C2*	10893505	rs1809136	11152180	C	G	0.93	9.99E-06	4.93E-02	7.54E-02
Brain_Putamen_basal_ganglia	2	*KCNF1*	11053206	rs1809136	11152180	C	G	0.93	9.99E-06	2.97E-02	5.39E-02
Brain_Putamen_basal_ganglia	2	*NTSR2*	11804297	rs1809136	11152180	C	G	0.93	9.99E-06	1.50E-02	3.56E-02
Brain_Spinal_cord_cervical_c-1	2	*RRM2*	10267000	rs1809136	11152180	C	G	0.93	9.99E-06	2.32E-02	4.62E-02
Brain_Substantia_nigra	2	*ATP6V1C2*	10893505	rs1809136	11152180	C	G	0.93	9.99E-06	2.13E-02	4.38E-02
Whole_Blood	2	*RP11-245G13.2*	11021819	rs1809136	11152180	C	G	0.93	9.99E-06	5.78E-03	2.14E-02

Supplementary Figures

Figure S1. Forest plots of betas (i.e., effect sizes) and 95% confidence interval for the association of the *APOE*/rs429358 SNP with PiB-PET

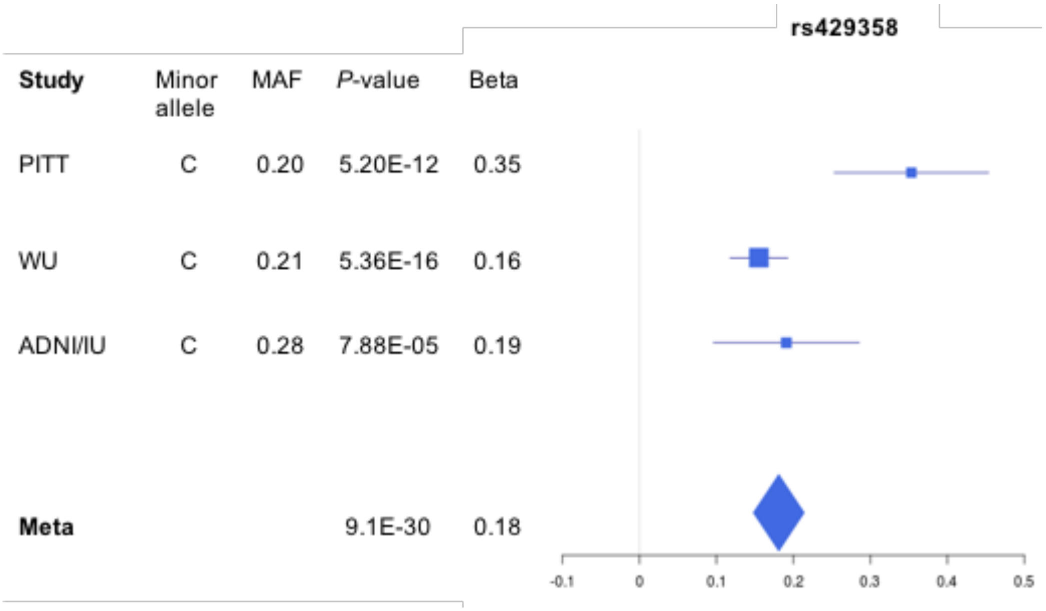

Figure S2. Regional plots of 15 non-*APOE* loci listed as S2a to S2o and they correspond to loci listed in Table 2.

Figure S2.1

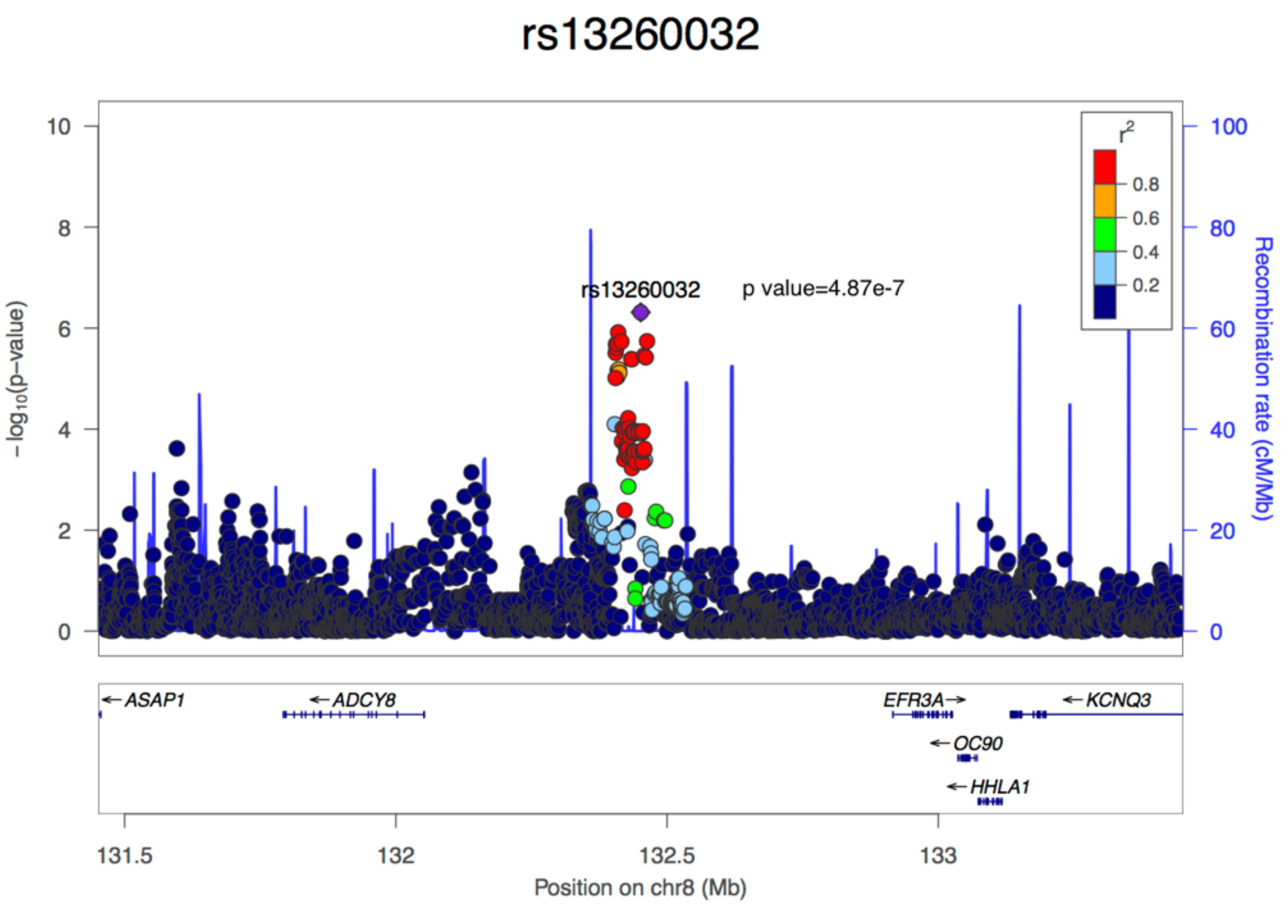

Figure S2.2

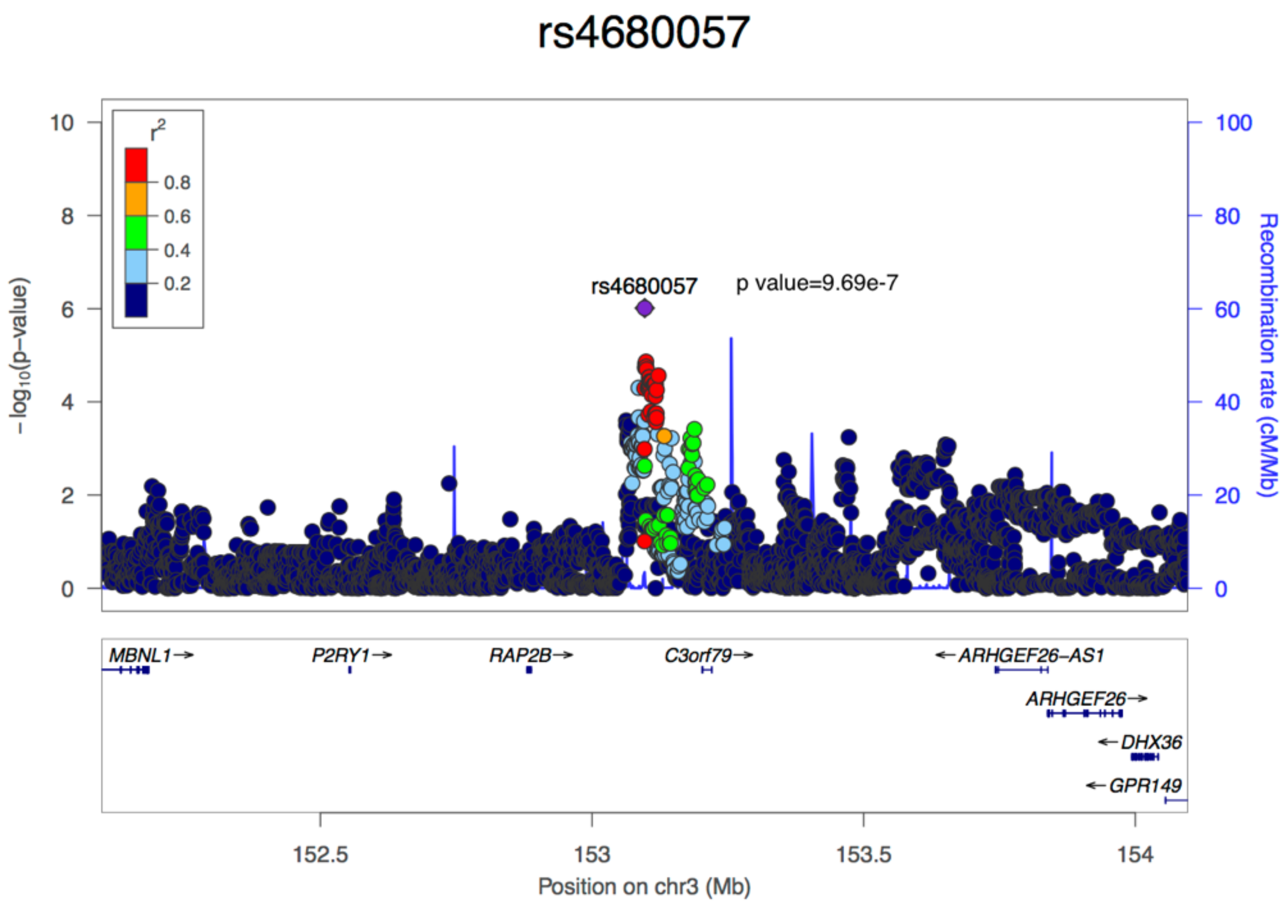

rs4680057
r²
0.8
0.6
0.4
0.2
rs4680057
p value=9.69e-7
−log10(p−value)
Recombination rate (cM/Mb)
10
8
6
4
2
0
100
80
60
40
20
0
MBNL1→
P2RY1→
RAP2B→
C3orf79→
←ARHGEF26−AS1
ARHGEF26→
←DHX36
←GPR149
152.5
153
153.5
154
Position on chr3 (Mb)

Figure S2.3

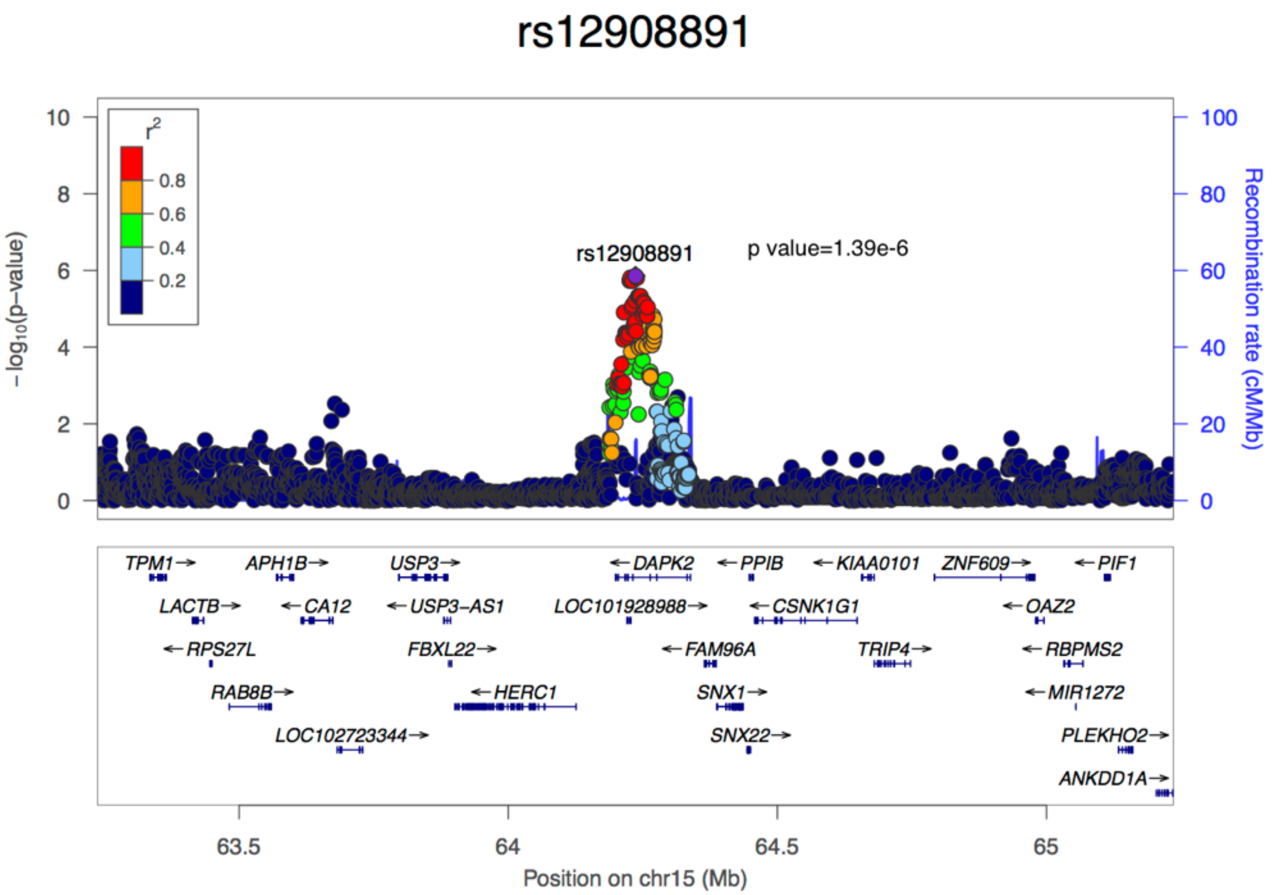

rs12908891
r2
0.8
0.6
0.4
0.2
rs12908891
p value=1.39e-6
−log10(p−value)
Recombination rate (cM/Mb)
Position on chr15 (Mb)
63.5
64
64.5
65
TPM1
APH1B
USP3
DAPK2
PPIB
KIAA0101
ZNF609
PIF1
LACTB
CA12
USP3−AS1
LOC101928988
CSNK1G1
OAZ2
RPS27L
FBXL22
FAM96A
TRIP4
RBPMS2
RAB8B
HERC1
SNX1
MIR1272
LOC102723344
SNX22
PLEKHO2
ANKDD1A

Figure S2.4

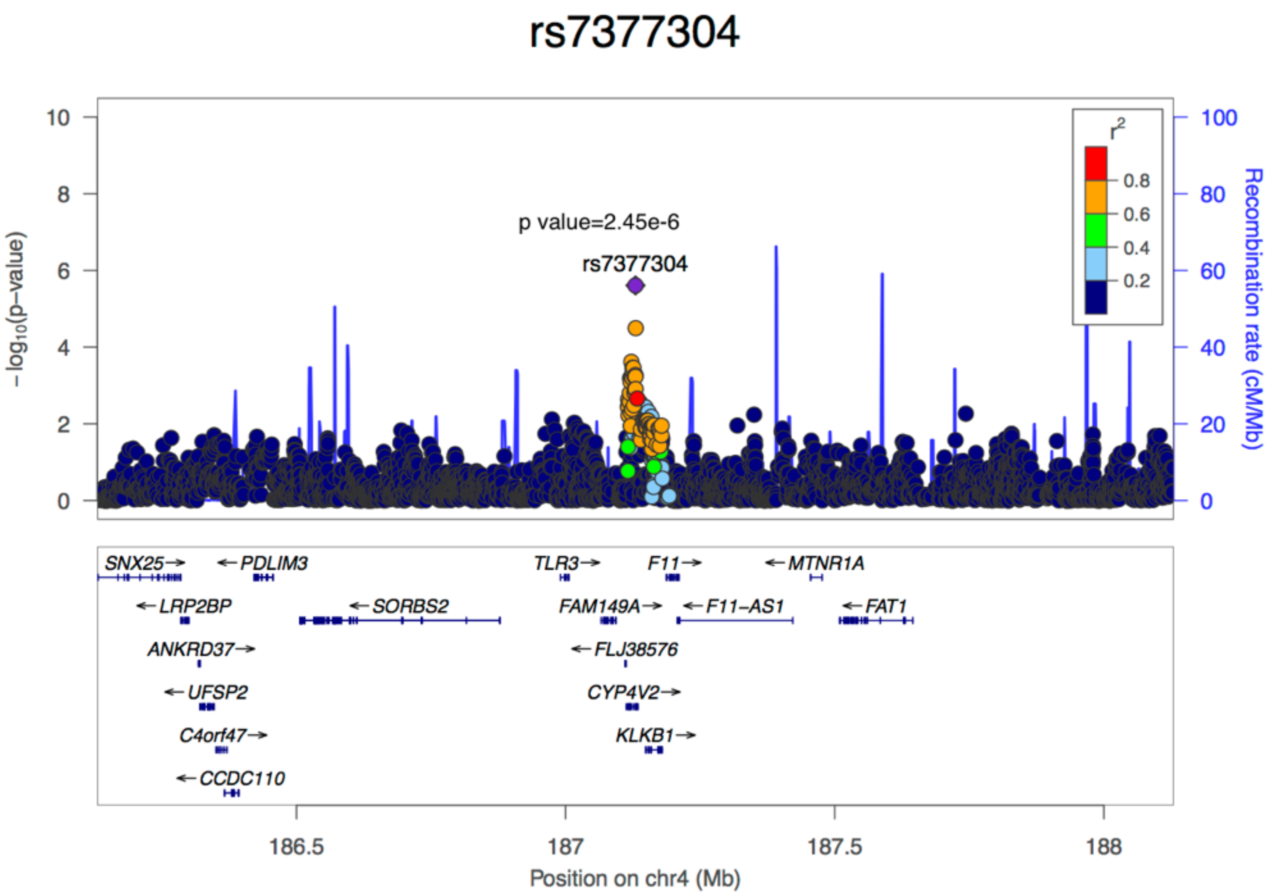

rs7377304
p value=2.45e-6
rs7377304
−log10(p−value)
Recombination rate (cM/Mb)
r2
0.8
0.6
0.4
0.2
SNX25
PDLIM3
LRP2BP
SORBS2
ANKRD37
UFSP2
C4orf47
CCDC110
TLR3
F11
MTNR1A
FAM149A
F11−AS1
FAT1
FLJ38576
CYP4V2
KLKB1
186.5
187
187.5
188
Position on chr4 (Mb)

Figure S2.5

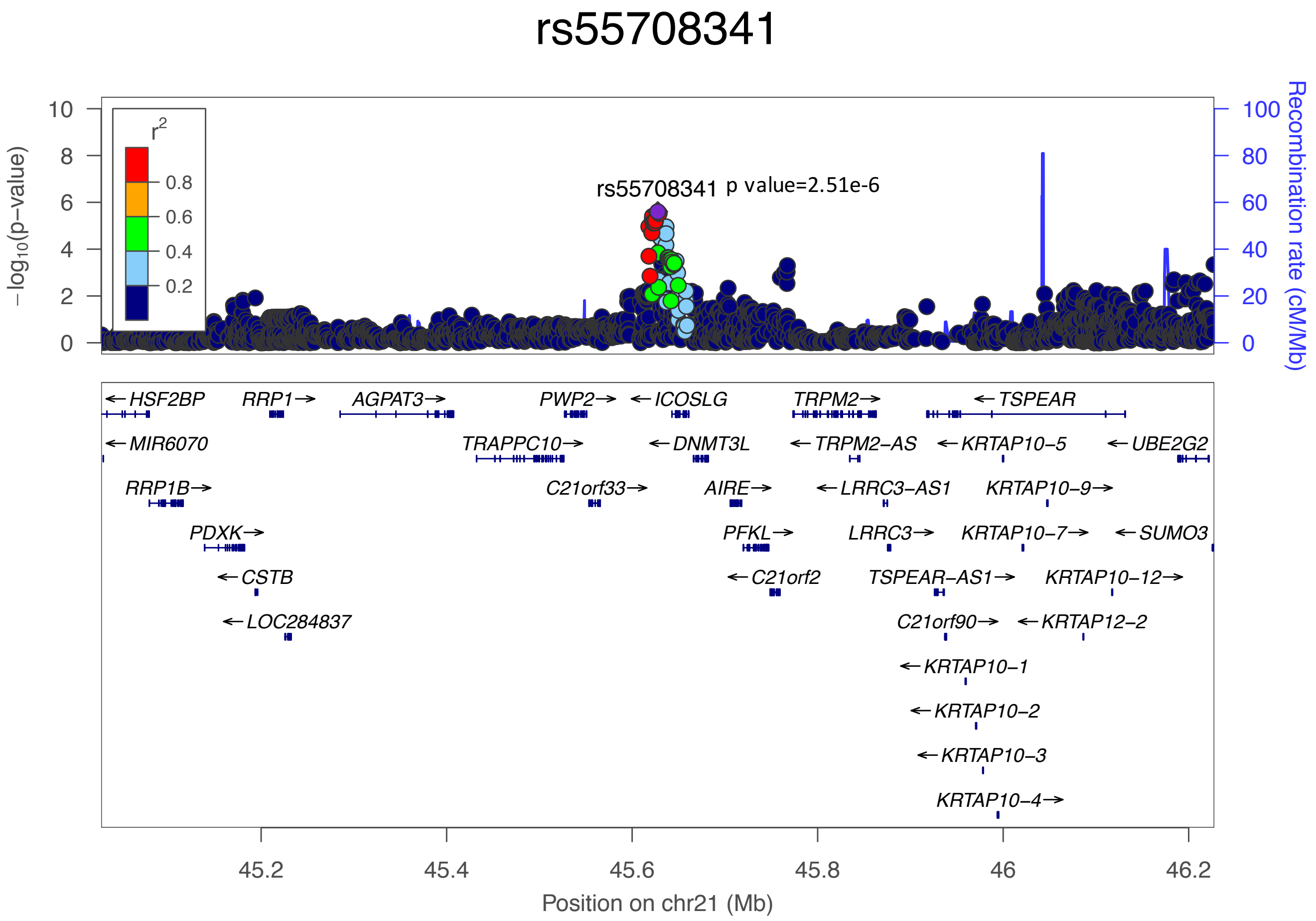

rs55708341
rs55708341 p value=2.51e-6
r2
0.8
0.6
0.4
0.2
−log10(p−value)
Recombination rate (cM/Mb)
HSF2BP
RRP1
AGPAT3
PWP2
ICOSLG
TRPM2
TSPEAR
MIR6070
TRAPPC10
DNMT3L
TRPM2−AS
KRTAP10−5
UBE2G2
RRP1B
C21orf33
AIRE
LRRC3−AS1
KRTAP10−9
PDXK
PFKL
LRRC3
KRTAP10−7
SUMO3
CSTB
C21orf2
TSPEAR−AS1
KRTAP10−12
LOC284837
C21orf90
KRTAP12−2
KRTAP10−1
KRTAP10−2
KRTAP10−3
KRTAP10−4
Position on chr21 (Mb)

Figure S2.6

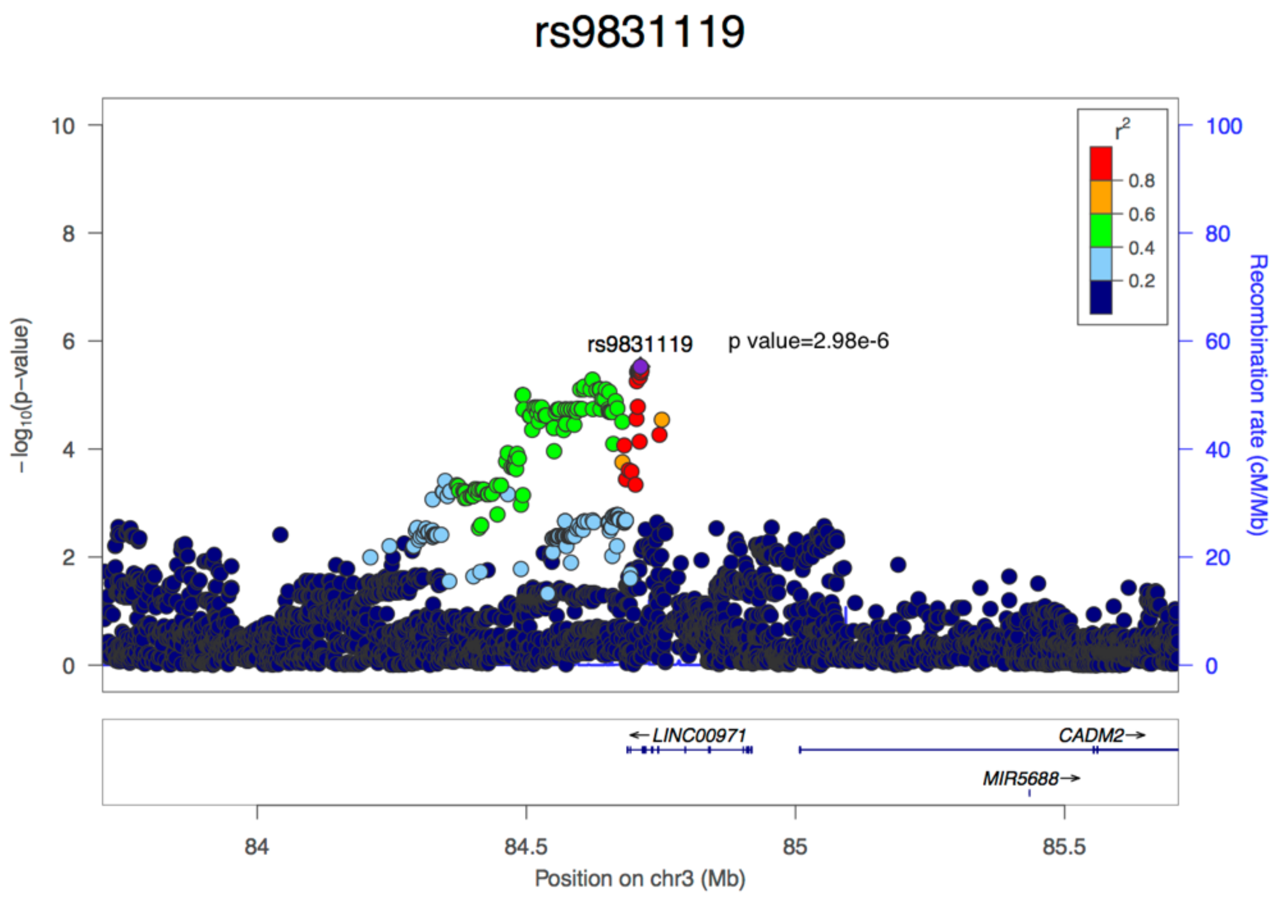
rs9831119
r2
0.8
0.6
0.4
0.2
rs9831119
p value=2.98e-6
10
8
6
4
2
0
−log10(p−value)
100
80
60
40
20
0
Recombination rate (cM/Mb)
LINC00971
CADM2
MIR5688
84
84.5
85
85.5
Position on chr3 (Mb)

Figure S2.7

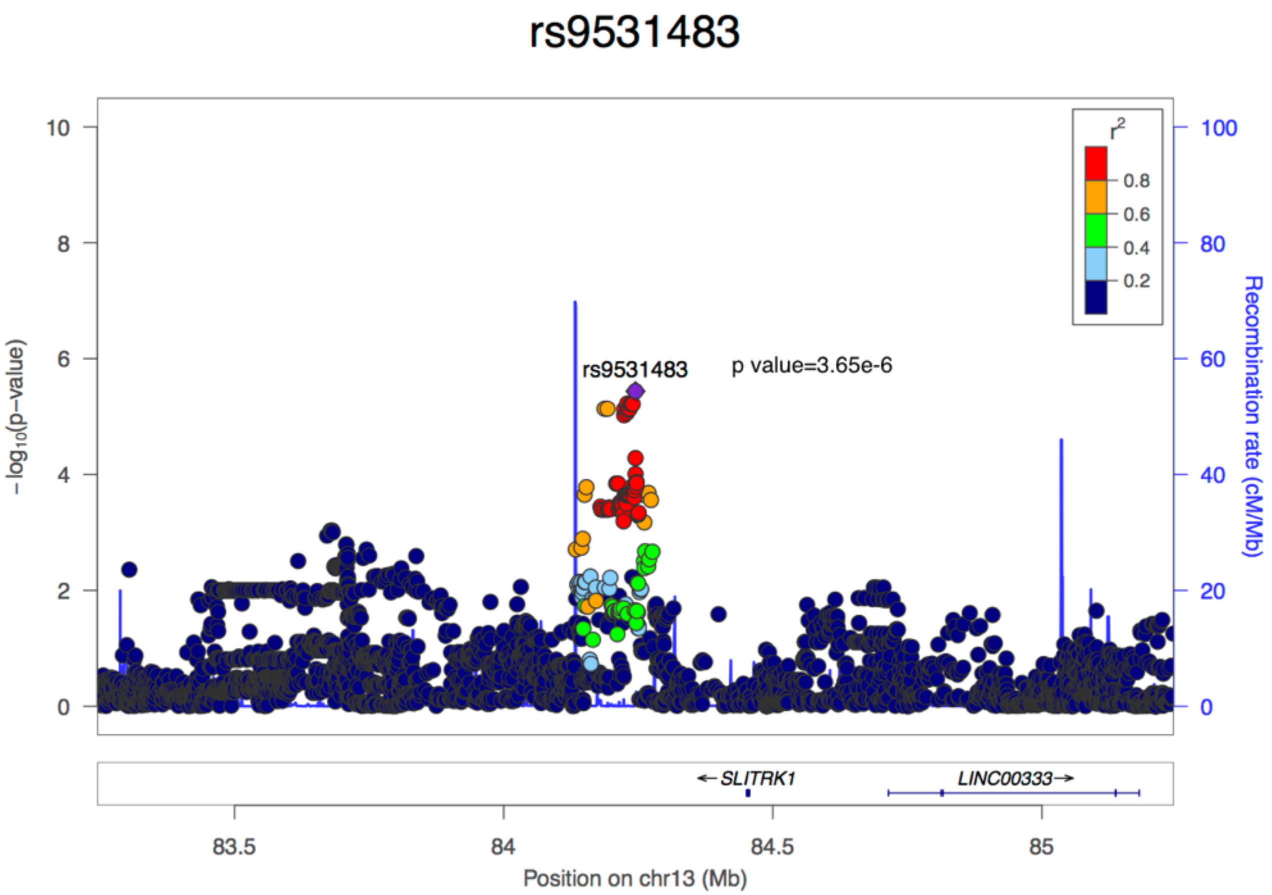

rs9531483
10
8
6
4
2
0
−log10(p−value)
100
80
60
40
20
0
Recombination rate (cM/Mb)
r2
0.8
0.6
0.4
0.2
rs9531483
p value=3.65e-6
← SLITRK1
LINC00333→
83.5
84
84.5
85
Position on chr13 (Mb)

Figure S2.8

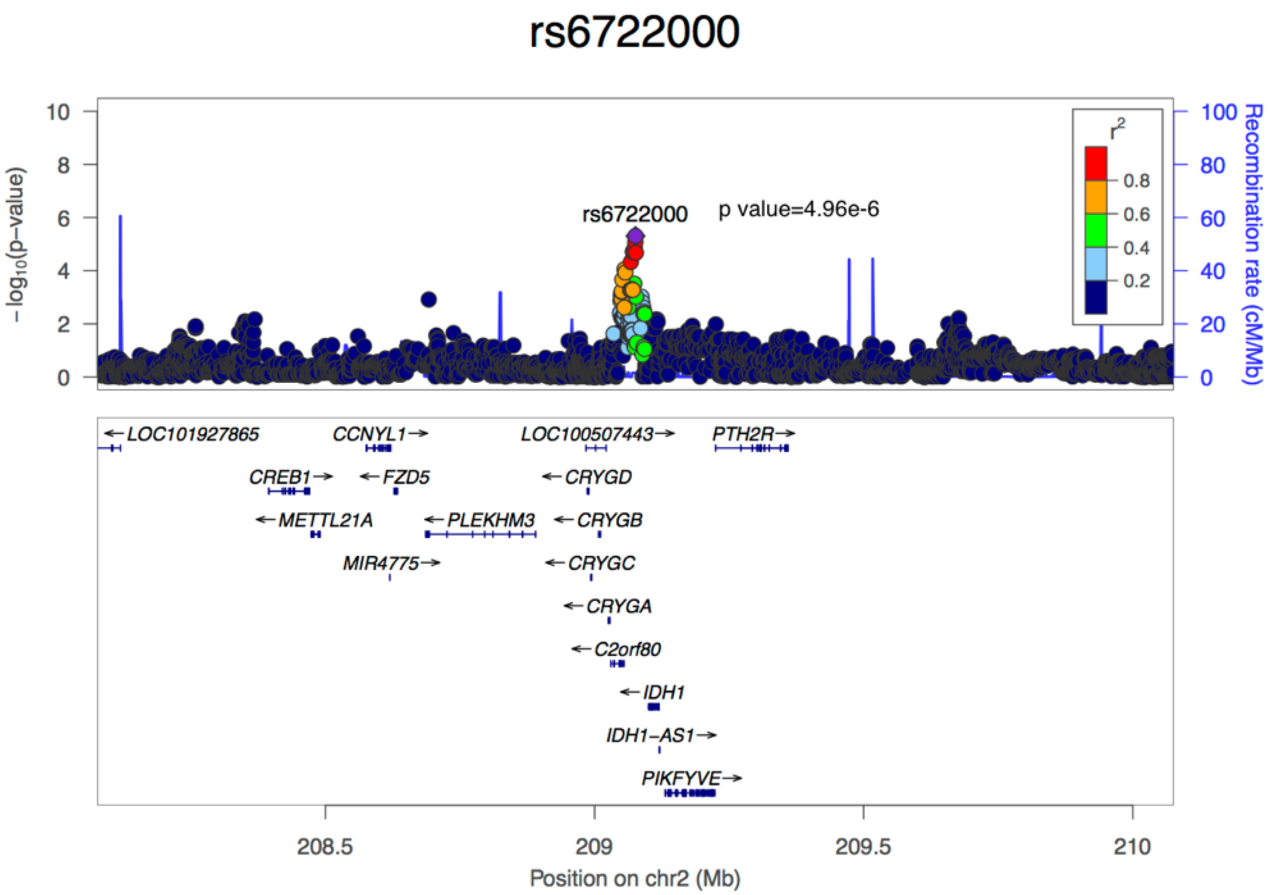
rs6722000
rs6722000
p value=4.96e-6
− log10(p−value)
Recombination rate (cM/Mb)
r2
0.8
0.6
0.4
0.2
LOC101927865
CCNYL1
LOC100507443
PTH2R
CREB1
FZD5
CRYGD
METTL21A
PLEKHM3
CRYGB
MIR4775
CRYGC
CRYGA
C2orf80
IDH1
IDH1−AS1
PIKFYVE
208.5
209
209.5
210
Position on chr2 (Mb)

Figure S2.9

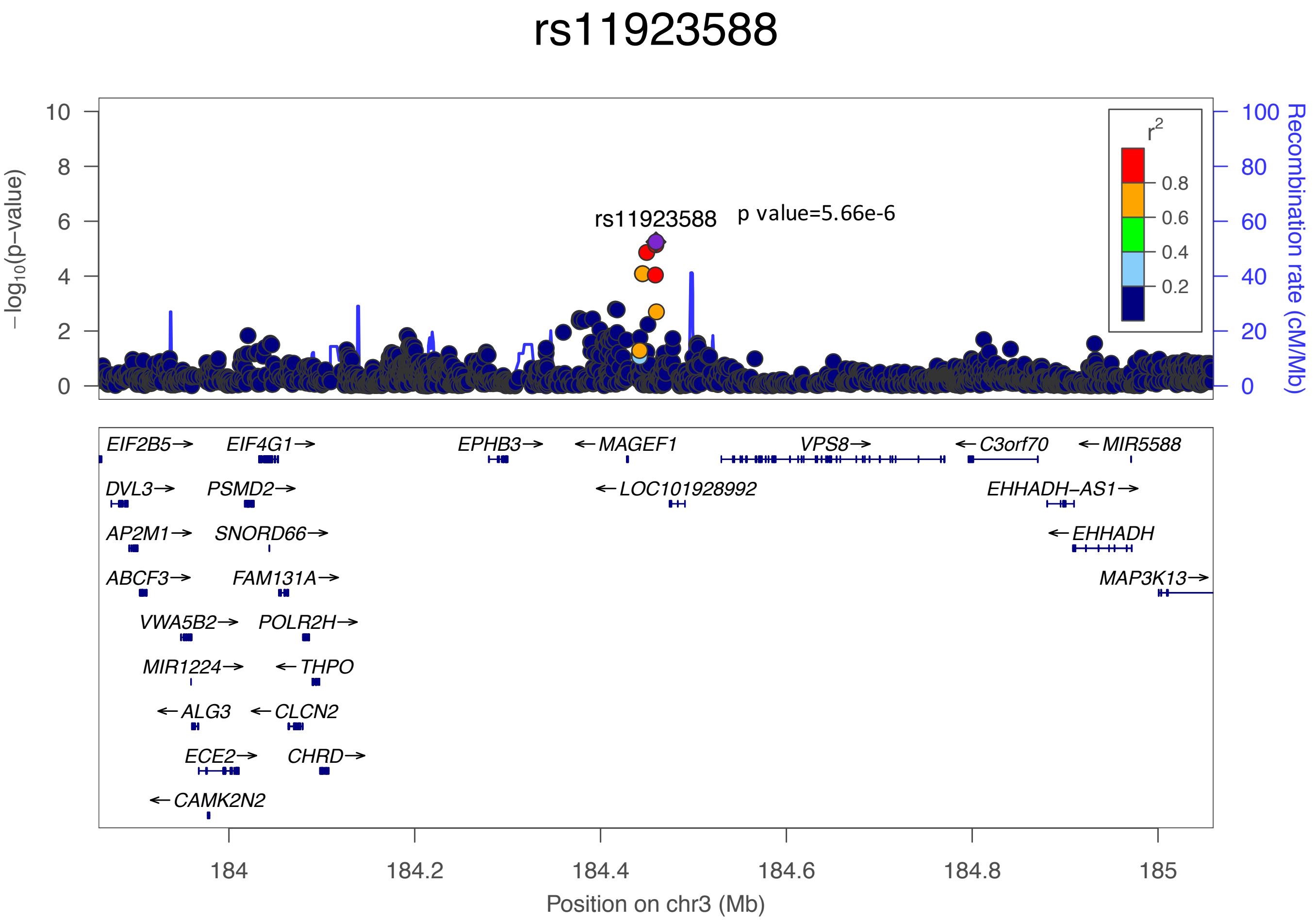
rs11923588
rs11923588
p value=5.66e-6
−log10(p−value)
Recombination rate (cM/Mb)
r2
0.8
0.6
0.4
0.2
EIF2B5→
EIF4G1→
EPHB3→
←MAGEF1
VPS8→
←C3orf70
←MIR5588
DVL3→
PSMD2→
←LOC101928992
EHHADH−AS1→
AP2M1→
SNORD66→
←EHHADH
ABCF3→
FAM131A→
MAP3K13→
VWA5B2→
POLR2H→
MIR1224→
←THPO
←ALG3
←CLCN2
ECE2→
CHRD→
←CAMK2N2
184
184.2
184.4
184.6
184.8
185
Position on chr3 (Mb)

Figure S2.10

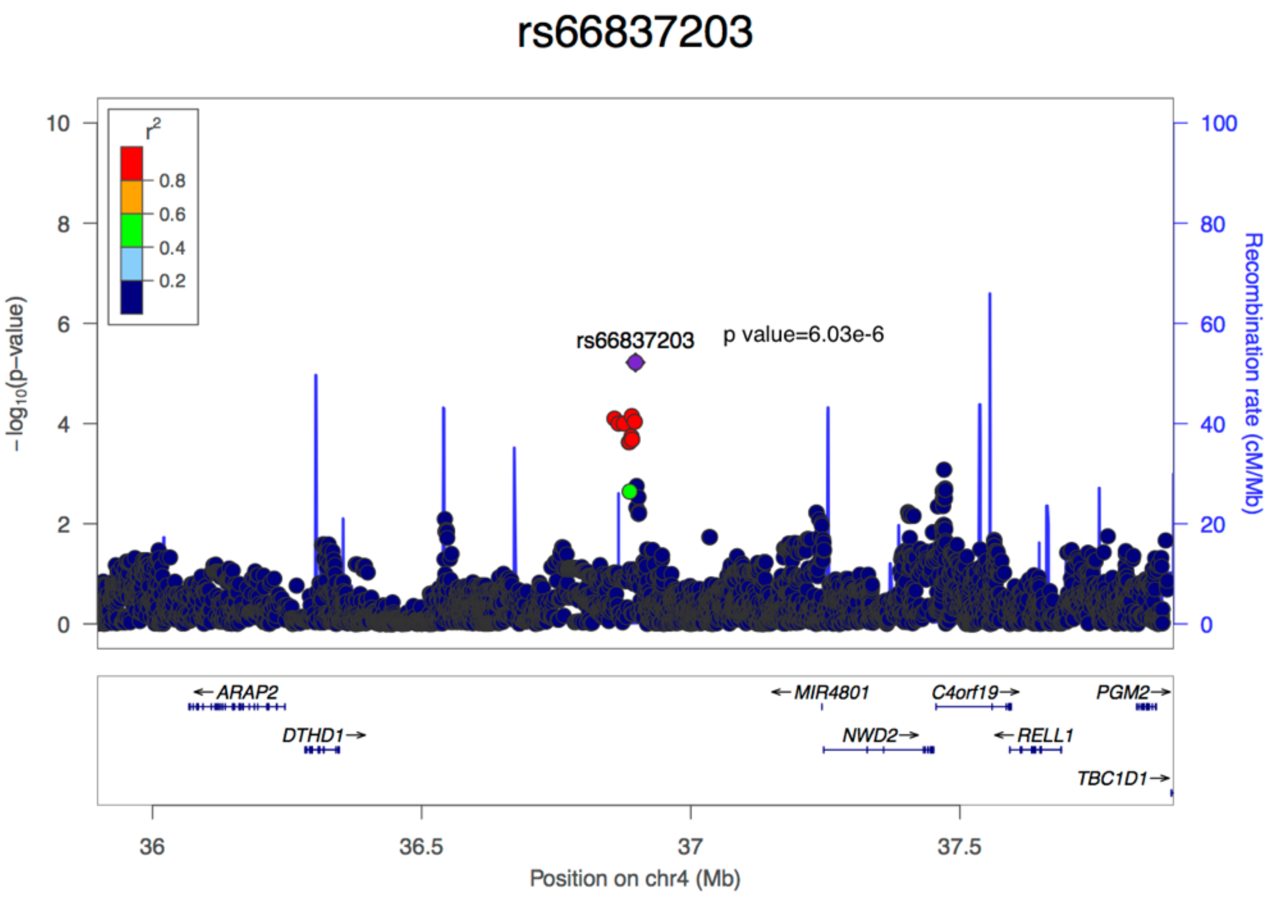
rs66837203
r2
0.8
0.6
0.4
0.2
rs66837203
p value=6.03e-6
−log10(p−value)
Recombination rate (cM/Mb)
ARAP2
DTHD1
MIR4801
NWD2
C4orf19
RELL1
PGM2
TBC1D1
36
36.5
37
37.5
Position on chr4 (Mb)

Figure S2.11

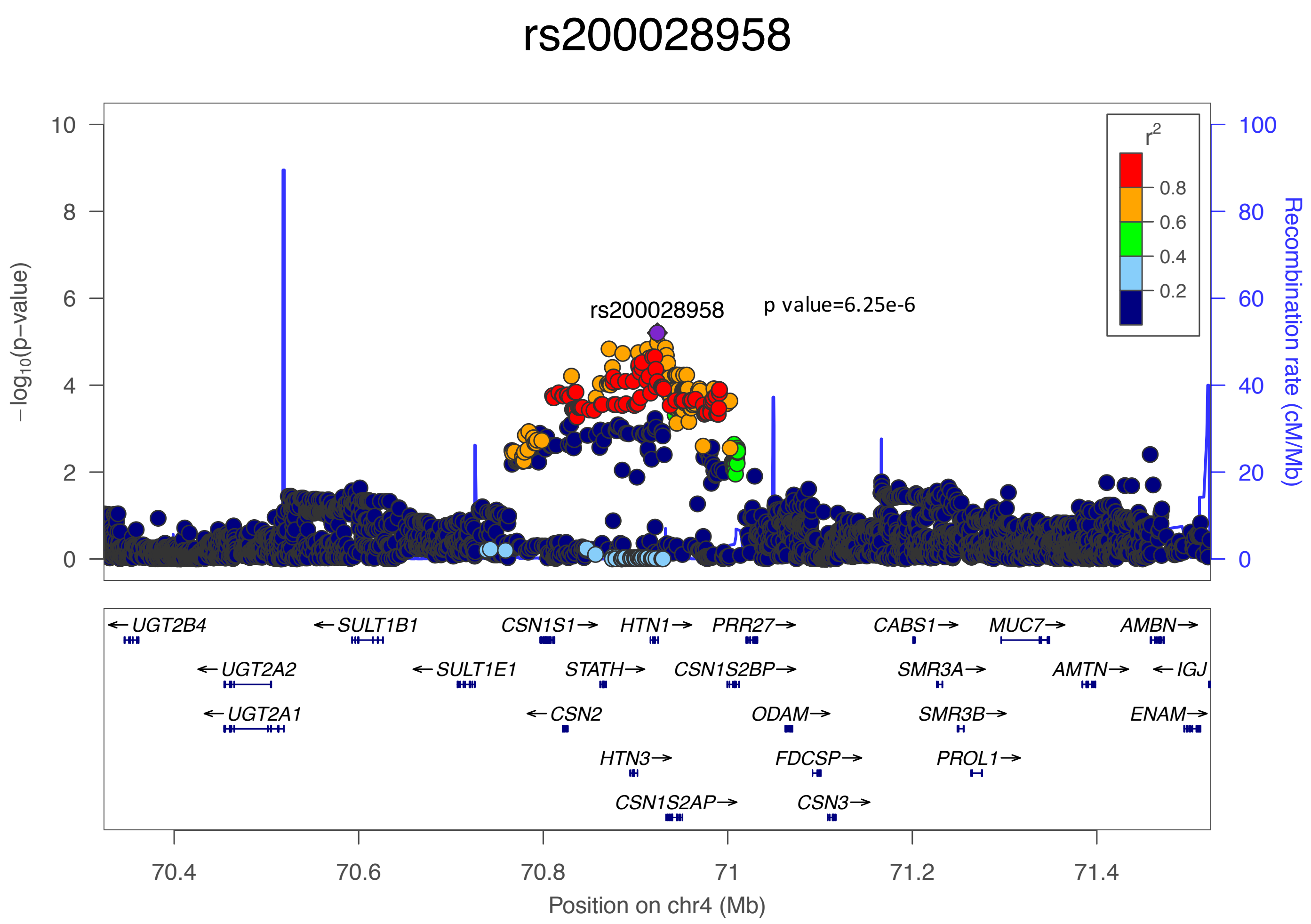
rs200028958
10
8
6
4
2
0
−log10(p−value)
100
80
60
40
20
0
Recombination rate (cM/Mb)
r2
0.8
0.6
0.4
0.2
rs200028958
p value=6.25e-6
←UGT2B4
←SULT1B1
CSN1S1→
HTN1→
PRR27→
CABS1→
MUC7→
AMBN→
←UGT2A2
←SULT1E1
STATH→
CSN1S2BP→
SMR3A→
AMTN→
←IGJ
←UGT2A1
←CSN2
ODAM→
SMR3B→
ENAM→
HTN3→
FDCSP→
PROL1→
CSN1S2AP→
CSN3→
70.4
70.6
70.8
71
71.2
71.4
Position on chr4 (Mb)

Figure S2.12

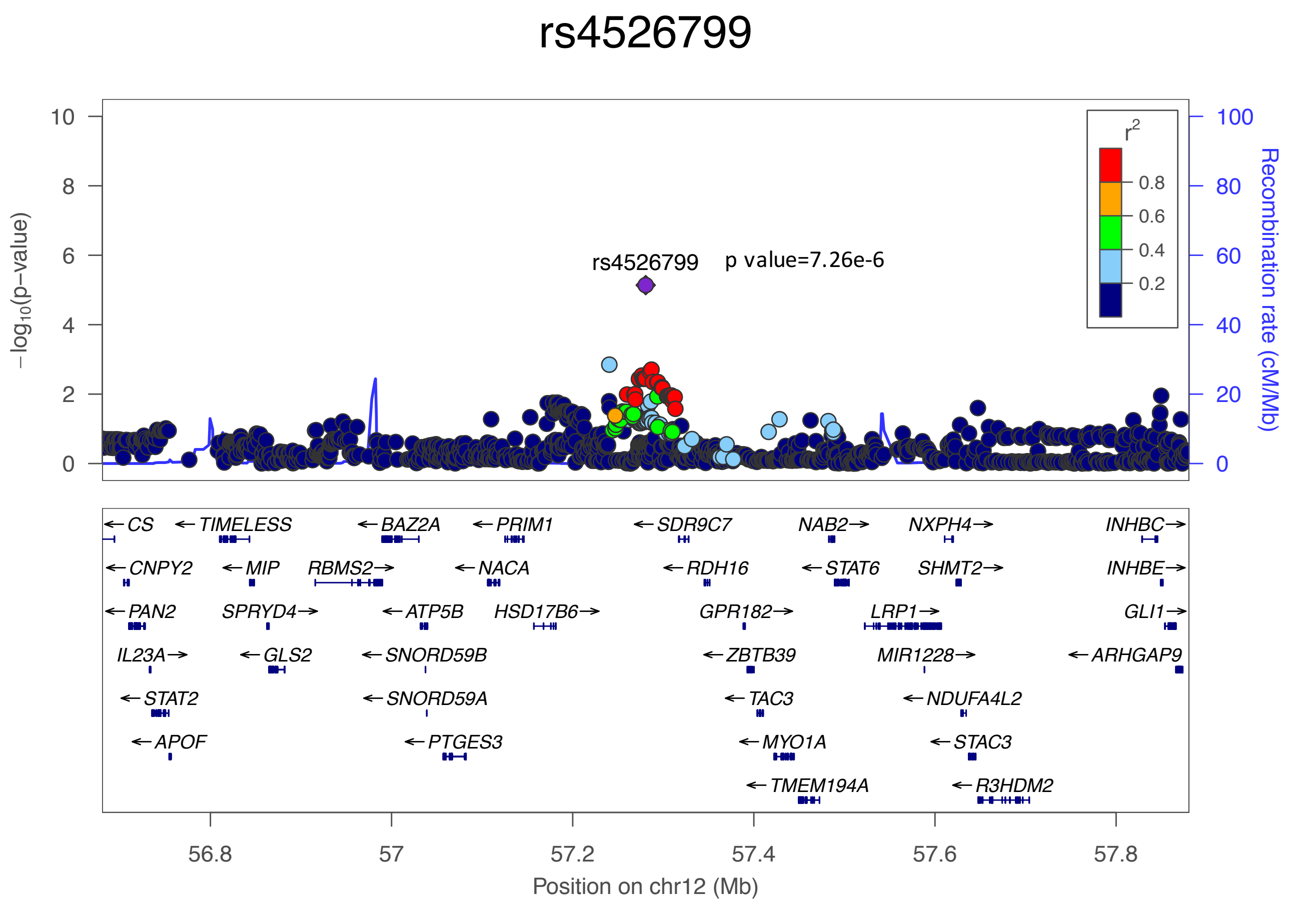

rs4526799
rs4526799
p value=7.26e-6
r2
0.8
0.6
0.4
0.2
−log10(p−value)
Recombination rate (cM/Mb)
10
8
6
4
2
0
100
80
60
40
20
0
CS
TIMELESS
BAZ2A
PRIM1
SDR9C7
NAB2
NXPH4
INHBC
CNPY2
MIP
RBMS2
NACA
RDH16
STAT6
SHMT2
INHBE
PAN2
SPRYD4
ATP5B
HSD17B6
GPR182
LRP1
GLI1
IL23A
GLS2
SNORD59B
ZBTB39
MIR1228
ARHGAP9
STAT2
SNORD59A
TAC3
NDUFA4L2
APOF
PTGES3
MYO1A
STAC3
TMEM194A
R3HDM2
56.8
57
57.2
57.4
57.6
57.8
Position on chr12 (Mb)

Figure S2.13

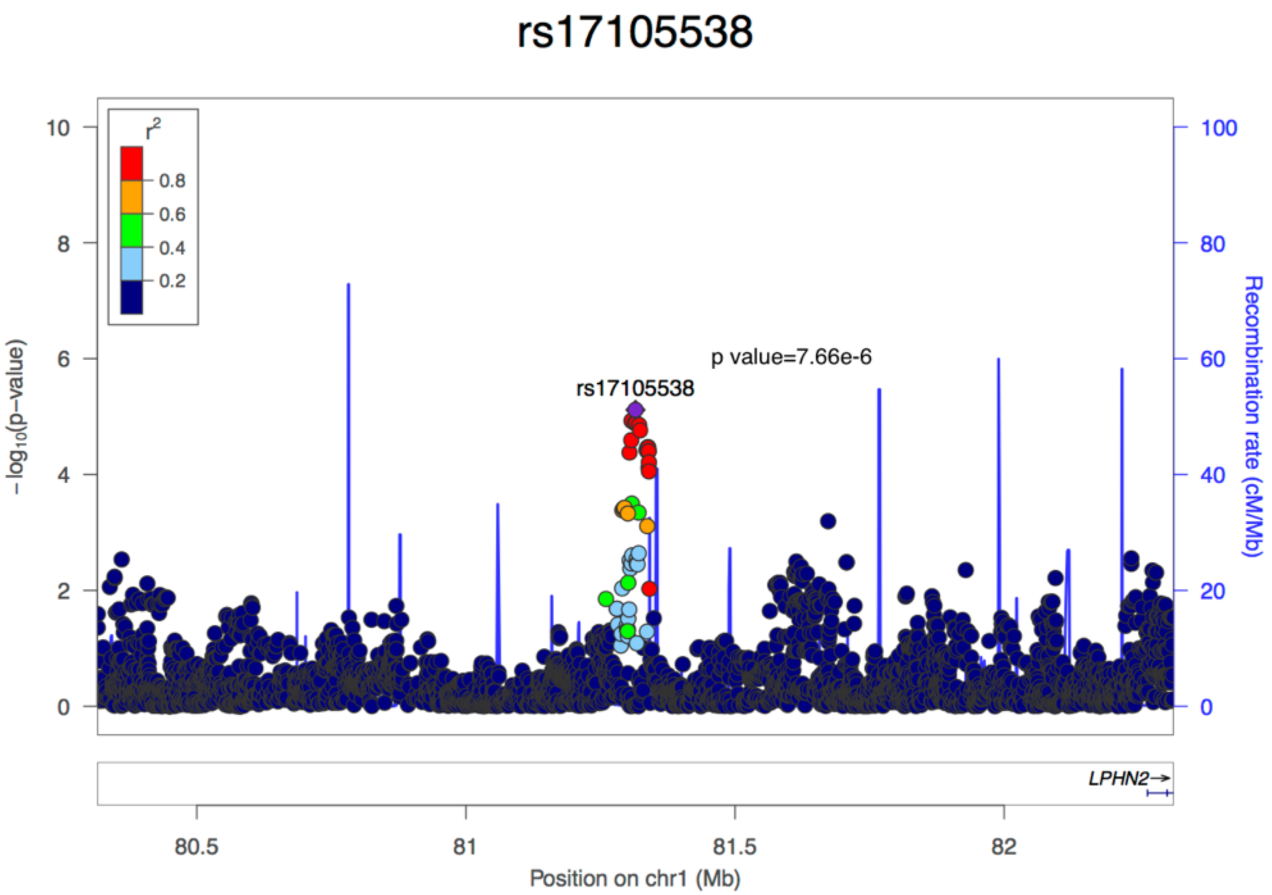
rs17105538
r²
0.8
0.6
0.4
0.2
p value=7.66e-6
rs17105538
−log₁₀(p−value)
Recombination rate (cM/Mb)
LPHN2
80.5
81
81.5
82
Position on chr1 (Mb)

Figure S2.14

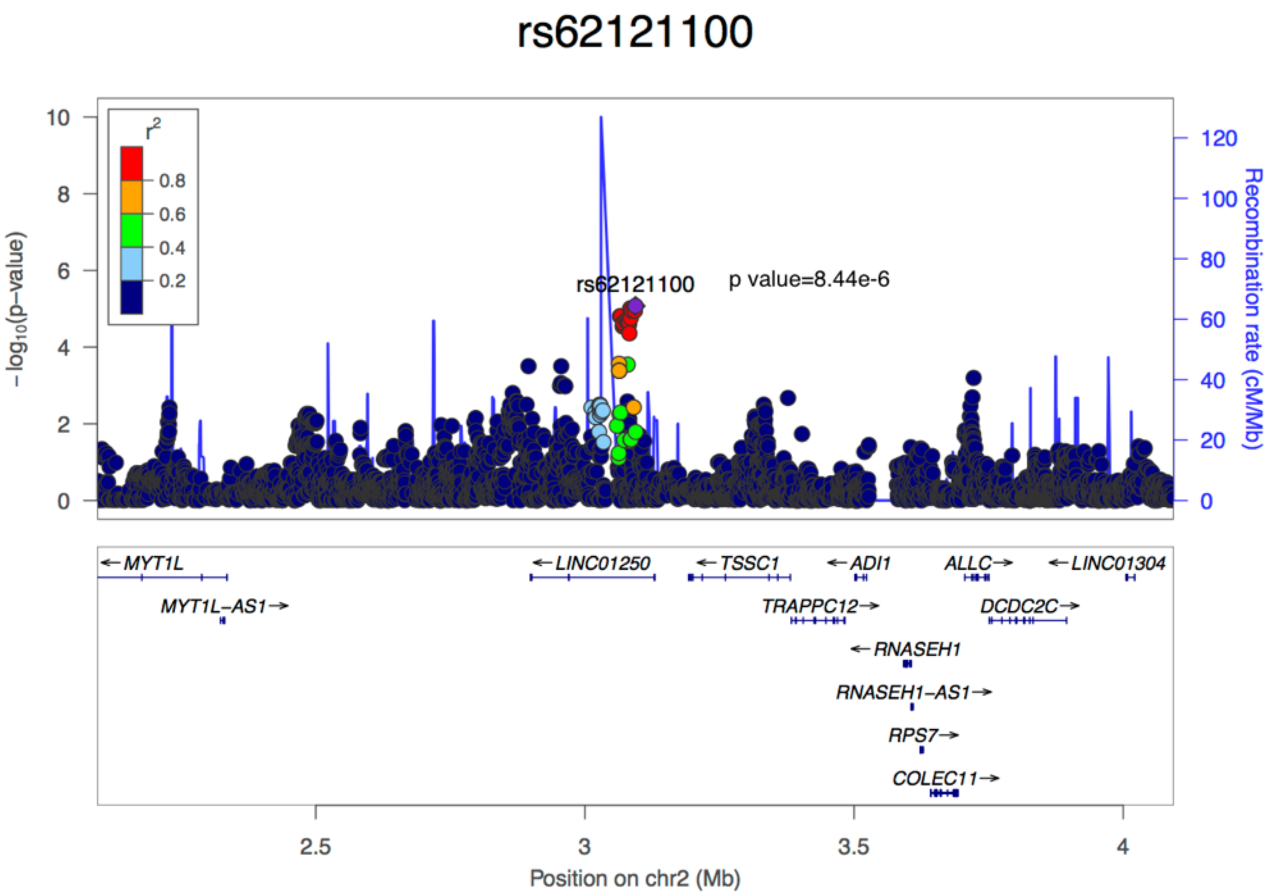
rs62121100
r2
0.8
0.6
0.4
0.2
rs62121100
p value=8.44e-6
−log10(p−value)
Recombination rate (cM/Mb)
MYT1L
MYT1L−AS1
LINC01250
TSSC1
ADI1
ALLC
LINC01304
TRAPPC12
DCDC2C
RNASEH1
RNASEH1−AS1
RPS7
COLEC11
Position on chr2 (Mb)

Figure S2.15

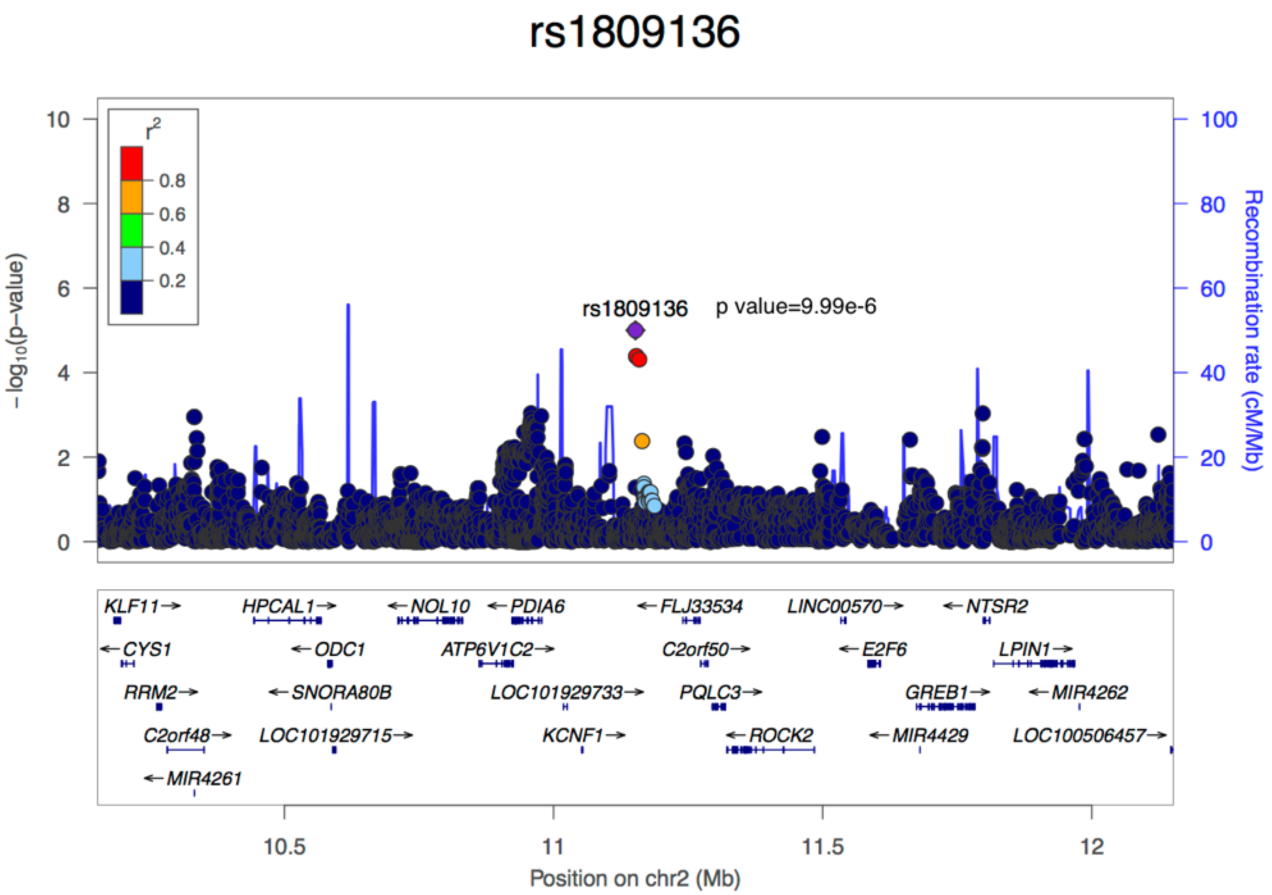
rs1809136
r2
0.8
0.6
0.4
0.2
rs1809136 p value=9.99e-6
−log10(p−value)
Recombination rate (cM/Mb)
KLF11→
HPCAL1→
←NOL10
←PDIA6
←FLJ33534
LINC00570→
←NTSR2
←CYS1
←ODC1
ATP6V1C2→
C2orf50→
←E2F6
LPIN1→
RRM2→
←SNORA80B
LOC101929733→
PQLC3→
GREB1→
←MIR4262
C2orf48→
LOC101929715→
KCNF1→
←ROCK2
←MIR4429
LOC100506457→
←MIR4261
Position on chr2 (Mb)

Figure S3. LD structure of SNPs listed in Table 2, including *APOE*4* (rs429358) in three datasets (PITT, WU, and ADNI/IU). R-squared values are shown in boxes.

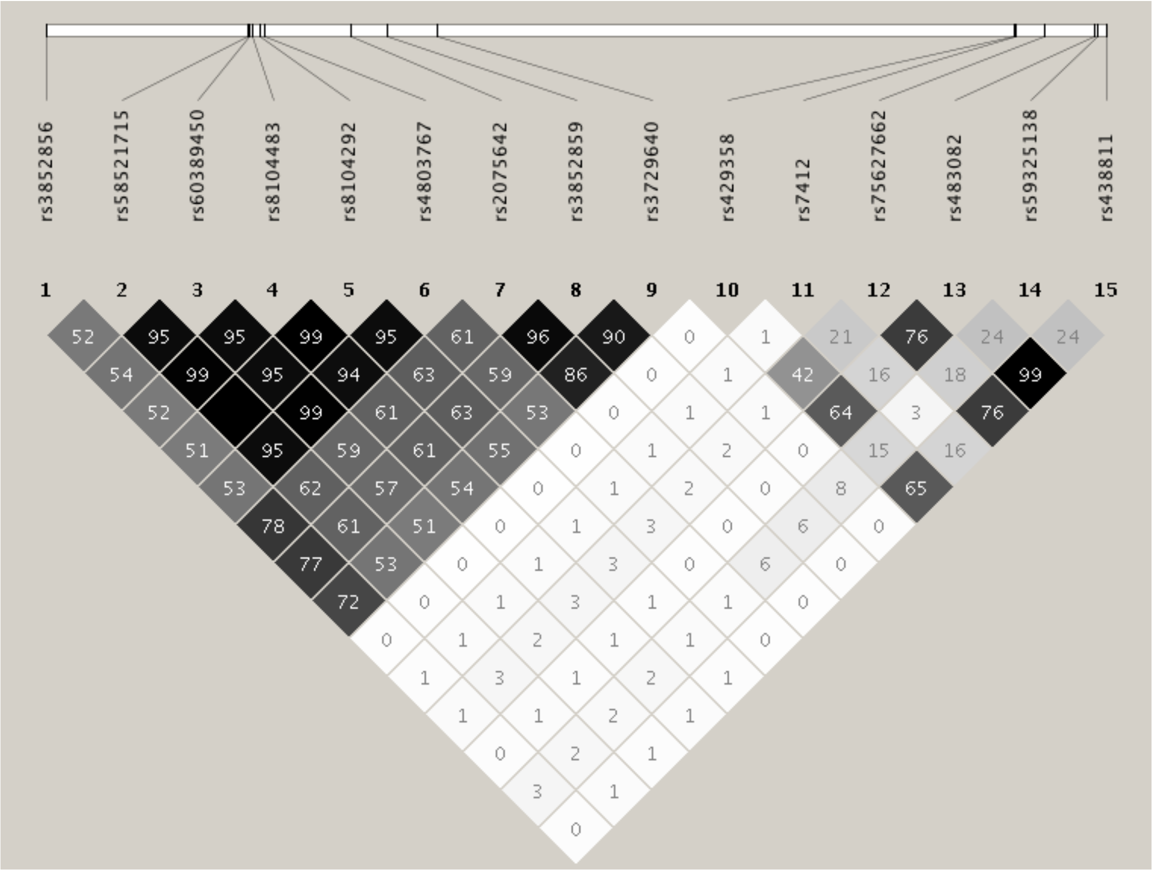

ACKNOWKEDGMENTS

This study was supported by the US National Institutes of Health (NIH) grants AG030653, AG041718, AG005133, AG025516, AG025204, AG044546, AG003991, AG053303, AG054936, and LM012535. The recruitment and clinical characterization of research participants at Washington University were supported by AG05681, AG003991, and AG026276. Data collection and sharing for the Alzheimer's Disease Neuroimaging Initiative (ADNI) was funded by NIH grant U01 AG024904 and DOD ADNI (Department of Defense award number W81XWH-12-2-0012). ADNI is funded by the National Institute on Aging, the National Institute of Biomedical Imaging and Bioengineering, and through generous contributions from the following: Alzheimer's Association; Alzheimer's Drug Discovery Foundation; BioClinica, Inc.; Biogen Idec Inc.; Bristol-Myers Squibb Company; Eisai Inc.; Elan Pharmaceuticals, Inc.; Eli Lilly and Company; F. Hoffmann-La Roche Ltd and its affiliated company Genentech, Inc.; GE Healthcare; Innogenetics, N.V.; IXICO Ltd.; Janssen Alzheimer Immunotherapy Research & Development, LLC.; Johnson & Johnson Pharmaceutical Research & Development LLC.; Medpace, Inc.; Merck & Co., Inc.; Meso Scale Diagnostics, LLC.; NeuroRx Research; Novartis Pharmaceuticals Corporation; Pfizer Inc.; Piramal Imaging; Servier; Synarc Inc.; and Takeda Pharmaceutical Company. The Canadian Institutes of Health Research is providing funds to support ADNI clinical sites in Canada. Private sector contributions are facilitated by the Foundation for the National Institutes of Health (www.fnih.org). The grantee organization is the Northern California Institute for Research and Education, and the study is coordinated by the Alzheimer's Disease Cooperative Study at the University of California, San Diego. ADNI data are disseminated by the Laboratory for Neuro Imaging at the University of Southern California. Samples from the National Cell Repository for AD (NCRAD), which receives government support under a cooperative agreement grant (U24 AG21886) awarded by the National Institute on Aging, were used in this study. Data used in the preparation of this article were obtained from the Alzheimer's Disease

Neuroimaging Initiative (ADNI) database (adni.loni.usc.edu). As such, the investigators within the ADNI contributed to the design and implementation of ADNI and/or provided data but did not participate in analysis or writing of this report. A complete listing of ADNI investigators can be found at http://adni.loni.usc.edu/wp-content/uploads/how_to_apply/ADNI_Acknowledgement_List.pdf

Supplementary References

1. Mathis CA, Kuller LH, Klunk WE, Snitz BE, Price JC, Weissfeld LA *et al.* In vivo assessment of amyloid-beta deposition in nondemented very elderly subjects. *Ann Neurol* 2013; **73**(6)**:** 751-761.

2. Nebes RD, Snitz BE, Cohen AD, Aizenstein HJ, Saxton JA, Halligan EM *et al.* Cognitive aging in persons with minimal amyloid-beta and white matter hyperintensities. *Neuropsychologia* 2013; **51**(11)**:** 2202-2209.

3. Morris JC, Roe CM, Xiong C, Fagan AM, Goate AM, Holtzman DM *et al.* APOE predicts amyloid-beta but not tau Alzheimer pathology in cognitively normal aging. *Ann Neurol* 2010; **67**(1)**:** 122-131.

4. Swaminathan S, Shen L, Risacher SL, Yoder KK, West JD, Kim S *et al.* Amyloid pathway-based candidate gene analysis of [(11)C]PiB-PET in the Alzheimer's Disease Neuroimaging Initiative (ADNI) cohort. *Brain Imaging Behav* 2012; **6**(1)**:** 1-15.

5. Saykin AJ, Shen L, Yao X, Kim S, Nho K, Risacher SL *et al.* Genetic studies of quantitative MCI and AD phenotypes in ADNI: Progress, opportunities, and plans. *Alzheimers Dement* 2015; **11**(7)**:** 792-814.

6. Jagust WJ, Bandy D, Chen K, Foster NL, Landau SM, Mathis CA *et al.* The Alzheimer's Disease Neuroimaging Initiative positron emission tomography core. *Alzheimers Dement* 2010; **6**(3)**:** 221-229.

7. Deters KD, Risacher SL, Yoder KK, Oblak AL, Unverzagt FW, Murrell JR *et al.* [(11)C]PiB PET in Gerstmann-Straussler-Scheinker disease. *Am J Nucl Med Mol Imaging* 2016; **6**(1)**:** 84-93.

8. Howie BN, Donnelly P, Marchini J. A flexible and accurate genotype imputation method for the next generation of genome-wide association studies. *PLoS Genet* 2009; **5**(6)**:** e1000529.

9. Genomes Project C, Abecasis GR, Altshuler D, Auton A, Brooks LD, Durbin RM *et al.* A map of human genome variation from population-scale sequencing. *Nature* 2010; **467**(7319)**:** 1061-1073.

10. Price AL, Patterson NJ, Plenge RM, Weinblatt ME, Shadick NA, Reich D. Principal components analysis corrects for stratification in genome-wide association studies. *Nat Genet* 2006; **38**(8)**:** 904-909.

11. Bai Z, Han G, Xie B, Wang J, Song F, Peng X *et al.* AlzBase: an Integrative Database for Gene Dysregulation in Alzheimer's Disease. *Mol Neurobiol* 2016; **53**(1)**:** 310-319.

12. Zhang Y, Chen K, Sloan SA, Bennett ML, Scholze AR, O'Keeffe S *et al.* An RNA-sequencing transcriptome and splicing database of glia, neurons, and vascular cells of the cerebral cortex. *J Neurosci* 2014; **34**(36)**:** 11929-11947.

13. Zhang Y, Sloan SA, Clarke LE, Caneda C, Plaza CA, Blumenthal PD *et al.* Purification and Characterization of Progenitor and Mature Human Astrocytes Reveals Transcriptional and Functional Differences with Mouse. *Neuron* 2016; **89**(1)**:** 37-53.

14. Consortium GT. The Genotype-Tissue Expression (GTEx) project. *Nat Genet* 2013; **45**(6)**:** 580-585.

15. Zhu Z, Zhang F, Hu H, Bakshi A, Robinson MR, Powell JE *et al.* Integration of summary data from GWAS and eQTL studies predicts complex trait gene targets. *Nat Genet* 2016; **48**(5)**:** 481-487.

16. de Leeuw CA, Mooij JM, Heskes T, Posthuma D. MAGMA: generalized gene-set analysis of GWAS data. *PLoS Comput Biol* 2015; **11**(4)**:** e1004219.

17. Ashburner M, Ball CA, Blake JA, Botstein D, Butler H, Cherry JM *et al.* Gene ontology: tool for the unification of biology. The Gene Ontology Consortium. *Nat Genet* 2000; **25**(1)**:** 25-29.

18. Gene Ontology C. Gene Ontology Consortium: going forward. *Nucleic Acids Res* 2015; **43**(Database issue)**:** D1049-1056.

19. Kanehisa M, Sato Y, Kawashima M, Furumichi M, Tanabe M. KEGG as a reference resource for gene and protein annotation. *Nucleic Acids Res* 2016; **44**(D1)**:** D457-462.

20. Ogata H, Goto S, Sato K, Fujibuchi W, Bono H, Kanehisa M. KEGG: Kyoto Encyclopedia of Genes and Genomes. *Nucleic Acids Res* 1999; **27**(1)**:** 29-34.

21. Fabregat A, Sidiropoulos K, Garapati P, Gillespie M, Hausmann K, Haw R *et al.* The Reactome pathway Knowledgebase. *Nucleic Acids Res* 2016; **44**(D1)**:** D481-487.

22. Croft D, O'Kelly G, Wu G, Haw R, Gillespie M, Matthews L *et al.* Reactome: a database of reactions, pathways and biological processes. *Nucleic Acids Res* 2011; **39**(Database issue)**:** D691-697.

23. Kamboh MI, Demirci FY, Wang X, Minster RL, Carrasquillo MM, Pankratz VS *et al.* Genome-wide association study of Alzheimer's disease. *Transl Psychiatry* 2012; **2:** e117.
