# Supplementary material for: Genome-wide association study of brain amyloid deposition as measured by Pittsburgh Compound-B (PiB)-PET imaging
Source: Mol Psychiatry. 2018 Oct 25;26(1):309–21. doi: 10.1038/s41380-018-0246-7 (PMC6219464; doi:10.1038/s41380-018-0246-7)
Supplement: Supplementary file 2 — Tables S6 [file 41380_2018_246_MOESM2_ESM.pdf]

Table S6. The detailed functional analysis results for 257 target genes

#Gene: genes included in each locus.
#Expression in brain (Barres): Barres Human Brain RNA-Seq (only FPKM > 1 was shown; FPKM > 10 signified by *)
#AD sum: Total number of differential expression from all transcriptome studies of Alzheimer's disease (AD).
#AD Up: Total number of up regulation from all transcriptome studies of AD.
#AD Dn: Total number of down regulation from all transcriptome studies of AD.
#AD Pe: Total number of dys-regulation with unknown direction from all transcriptome studies of AD.
#The highlighted AD_sum, AD_Up, AD_Dn and AD_Pe indicate Genes up- or downregulated exclusively in two or more studies
#Blood.lowest.p_eQTL: The lowest eQTL p-value between any SNP and the corresponding gene in whole blood
#Blood.lowest.p_SMR: The lowest SMR p-value between any SNP and the corresponding gene in whole blood
#Brain.lowest.p_eQTL: The lowest eQTL p-value between any SNP and the corresponding gene in any brain tissues
#Brain.lowest.p_SMR: The lowest SMR p-value between any SNP and the corresponding gene in any brain tissues
#Pathway: Nominally significant pathways (p-value<0.05) including the corresponding gene
#Non-APOE locus pathway: Nominally significant pathways (p-value<0.05) including the corresponding gene and not including APOE-locus genes

Gene	Locus	Chr	Expression in brain (Barres)	AD_sum	AD_Up	AD_Dn	AD_Pe	Blood.lowest.p_eQTL	Blood.lowest.p_SMR	Brain.lowest.p_eQTL	Brain.lowest.p_SMR
APOC1	*APOE*	19	NA	11	11	0	0	NA	NA	0.0130436	0.01714493
APOC1P1	*APOE*	19	NA	NA	NA	NA	NA	NA	NA	0.0169751	0.0214768
APOC2	*APOE*	19	NA	3	2	1	0	NA	NA	0.0271157	0.03207496
APOC4	*APOE*	19	NA	2	1	1	0	NA	NA	0.00309672	0.006630909
APOC4-APOC2	*APOE*	19	NA	NA	NA	NA	NA	NA	NA	NA	NA
APOE	*APOE*	19	MatureAstrocytes,FetalAstrocytes	4	4	0	0	NA	NA	0.006062548	0.008965919
BCAM	*APOE*	19	NA	3	3	0	0	NA	NA	0.0114098	0.01718973
BCL3	*APOE*	19	NA	5	5	0	0	NA	NA	0.03741179	0.04308533
BLOC1S3	*APOE*	19	Endothelial	NA	NA	NA	NA	0.02308549	0.02813479	NA	NA
CBLC	*APOE*	19	NA	0	0	0	0	NA	NA	NA	NA
CD3EAP	*APOE*	19	NA	NA	NA	NA	NA	0.00216876	0.003192043	0.000291562	0.000849297
CEACAM16	*APOE*	19	NA	NA	NA	NA	NA	NA	NA	NA	NA
CEACAM19	*APOE*	19	NA	2	0	2	0	0.02056821	0.02533825	0.0272381	0.03581018
CEACAM20	*APOE*	19	NA	NA	NA	NA	NA	1	1	NA	NA
CEACAM22P	*APOE*	19	NA	NA	NA	NA	NA	1	1	NA	NA
CKM	*APOE*	19	NA	NA	NA	NA	NA	0.0457541	0.05437545	0.0106896	0.01660494
CLASRP	*APOE*	19	NA	6	6	0	0	0.008379739	0.01403407	0.00332479	0.005458997
CLPTM1	*APOE*	19	MatureAstrocytes*,FetalAstrocytes,Neurons,Endothelial,Oligodendrocytes,Microglia/Macrophage	7	2	5	0	NA	NA	0.008161097	0.01311337
ERCC1	*APOE*	19	Microglia/Macrophage*,FetalAstrocytes,MatureAstrocytes,Neurons,Oligodendrocytes,Endothelial	1	0	1	0	1	1	0.003903099	0.006265742
ERCC2	*APOE*	19	NA	4	0	4	0	0.0285481	0.03387421	0.0131752	0.01725854
EXOC3L2	*APOE*	19	NA	0	0	0	0	0.00431701	0.008978838	0.0277449	0.03299044
GEMIN7	*APOE*	19	FetalAstrocytes,Neurons	1	0	1	0	NA	NA	0.0124582	0.01635656
IGSF23	*APOE*	19	NA	NA	NA	NA	NA	0.0445799	0.05416729	NA	NA
KLC3	*APOE*	19	NA	NA	NA	NA	NA	0.041254	0.05070623	0.0167149	0.02370563
MARK4	*APOE*	19	FetalAstrocytes	6	1	5	0	NA	NA	0.0455546	0.05146517
MIR4531	*APOE*	19	NA	NA	NA	NA	NA	NA	NA	NA	NA
MIR8085	*APOE*	19	NA	NA	NA	NA	NA	NA	NA	NA	NA
NKPD1	*APOE*	19	NA	1	1	0	0	0.0253115	0.03445331	0.000293253	0.00156271
PPP1R13L	*APOE*	19	NA	6	6	0	0	NA	NA	0.04719221	0.05615469
PPP1R37	*APOE*	19	NA	NA	NA	NA	NA	NA	NA	0.000341028	0.001896521
PVR	*APOE*	19	NA	NA	NA	NA	NA	NA	NA	0.0129687	0.02099118
PVRL2	*APOE*	19	Endothelial,FetalAstrocytes,MatureAstrocytes	3	3	0	0	0.0284957	0.03383601	0.009117321	0.01466462
RELB	*APOE*	19	NA	1	1	0	0	NA	NA	0.0162579	0.02354847
TOMM40	*APOE*	19	NA	1	0	1	0	NA	NA	0.0172514	0.02153904
TRAPPC6A	*APOE*	19	NA	6	0	6	0	NA	NA	0.0117093	0.01889504
ZNF180	*APOE*	19	FetalAstrocytes,Neurons	1	1	0	0	NA	NA	0.000224494	0.001364411
ZNF229	*APOE*	19	NA	NA	NA	NA	NA	NA	NA	0.009165888	0.01263481
ZNF296	*APOE*	19	NA	2	1	1	0	NA	NA	0.00121543	0.003835691
ADCY8	*ADCY8,EFR3A*	8	MatureAstrocytes,Neurons	2	1	1	0	NA	NA	0.003887251	0.01794854
EFR3A	*ADCY8,EFR3A*	8	Neurons*,FetalAstrocytes,MatureAstrocytes,Oligodendrocytes,Microglia/Macrophage,Endothelial	1	1	0	0	NA	NA	0.02375819	0.04567933
7SK	*ADCY8,EFR3A*	8	NA	NA	NA	NA	NA	NA	NA	NA	NA
RAP2B	*RAP2B,C3orf79*	3	Microglia/Macrophage,Neurons,FetalAstrocytes,Oligodendrocytes,MatureAstrocytes	1	1	0	0	NA	NA	0.003429461	0.02490314
C3orf79	*RAP2B,C3orf79*	3	NA	NA	NA	NA	NA	NA	NA	NA	NA
AK092619ARHGEF26	*RAP2B,C3orf79*	3	NA	NA	NA	NA	NA	NA	NA	NA	NA
ARHGEF26-AS1	*RAP2B,C3orf79*	3	MatureAstrocytes,Neurons	NA	NA	NA	NA	NA	NA	0.009571812	0.05225144

DHX36	*RAP2B,C3orf79*	3	Microglia/Macrophage*,FetalAstrocytes,MatureAstrocytes,Neurons,Oligodendrocytes,Endothelial	6	0	6	0	0.0202715	0.07707963	0.008093867	0.06693651
AK091624	*DAPK2*	15	NA	NA	NA	NA	NA	NA	NA	NA	NA
AL137522	*DAPK2*	15	NA	NA	NA	NA	NA	NA	NA	NA	NA
CSNK1G1	*DAPK2*	15	FetalAstrocytes,Neurons,Microglia/Macrophage,Oligodendrocytes,MatureAstrocytes	1	1	0	0	NA	NA	0.0251826	0.05621508
DAPK2	*DAPK2*	15	Oligodendrocytes	NA	NA	NA	NA	0.001214999	0.02672872	4.8018E-05	0.005423494
FAM96A	*DAPK2*	15	Microglia/Macrophage*,Oligodendrocytes*,FetalAstrocytes*,MatureAstrocytes,Neurons,Endothelial	NA	NA	NA	NA	NA	NA	0.0115372	0.06143011
FBXL22	*DAPK2*	15	NA	5	2	3	0	0.0194601	0.03453912	0.000680118	0.008416777
HERC1	*DAPK2*	15	Neurons*,FetalAstrocytes,Microglia/Macrophage,MatureAstrocytes,Oligodendrocytes,Endothelial	5	1	4	0	NA	NA	5.28909E-06	0.001165938
KIAA0101	*DAPK2*	15	FetalAstrocytes	NA	NA	NA	NA	0.002086639	0.01284171	0.01481601	0.04558375
LOC101928988	*DAPK2*	15	NA	NA	NA	NA	NA	NA	NA	NA	NA
Mir_422	*DAPK2*	15	NA	NA	NA	NA	NA	NA	NA	NA	NA
PPIB	*DAPK2*	15	FetalAstrocytes*,MatureAstrocytes*,Microglia/Macrophage*,Oligodendrocytes*,Endothelial*,Neurons	5	1	4	0	NA	NA	0.013666	0.03928803
SNX1	*DAPK2*	15	Oligodendrocytes,MatureAstrocytes,Microglia/Macrophage,FetalAstrocytes,Neurons,Endothelial	NA	NA	NA	NA	NA	NA	0.0235128	0.0528879
SNX22	*DAPK2*	15	Oligodendrocytes,FetalAstrocytes	3	3	0	0	NA	NA	0.001059241	0.007676698
TRIP4	*DAPK2*	15	FetalAstrocytes,MatureAstrocytes,Neurons,Oligodendrocytes,Microglia/Macrophage,Endothelial	1	0	1	0	0.0394381	0.0773594	0.008576522	0.02616356
USP3	*DAPK2*	15	FetalAstrocytes,MatureAstrocytes,Neurons,Oligodendrocytes,Microglia/Macrophage,Endothelial	5	5	0	0	NA	NA	0.015184	0.05930517
USP3-AS1	*DAPK2*	15	NA	NA	NA	NA	NA	NA	NA	0.004499268	0.01660221
LACTB	*DAPK2*	15	Microglia/Macrophage*,MatureAstrocytes,Neurons,Oligodendrocytes,Endothelial	NA	NA	NA	NA	0.029037	0.05474122	0.00278342	0.01652845
RPS27L	*DAPK2*	15	Neurons*,Oligodendrocytes*,Microglia/Macrophage*,FetalAstrocytes,MatureAstrocytes,Endothelial	3	0	3	0	0.04597681	0.07377635	0.02669451	0.04958412
RAB8B	*DAPK2*	15	Microglia/Macrophage*,FetalAstrocytes,MatureAstrocytes,Neurons,Oligodendrocytes,Endothelial	1	1	0	0	NA	NA	0.00354762	0.01683508
ZNF609	*DAPK2*	15	FetalAstrocytesi*,MatureAstrocytes,Neurons,Oligodendrocytes,Microglia/Macrophage,Endothelial	10	10	0	0	NA	NA	0.02761069	0.05413036
PLEKHO2	*DAPK2*	15	MatureAstrocytes,Microglia/Macrophage	6	5	1	0	NA	NA	0.009212592	0.04522358
ANKDD1A	*DAPK2*	15	FetalAstrocytes,MatureAstrocytes,Neurons,Microglia/Macrophage	4	4	0	0	NA	NA	0.008213609	0.03241788
CYP4V2	*CYP4V2*	4	MatureAstrocytes*,Oligodendrocytes,Microglia/Macrophage,Endothelial,Neurons,FetalAstrocytes	3	0	3	0	1.51E-05	0.005551456	2.48706E-07	0.001659108
F11	*CYP4V2*	4	NA	NA	NA	NA	NA	NA	NA	3.32919E-09	0.001186214
F11-AS1	*CYP4V2*	4	NA	NA	NA	NA	NA	NA	NA	0.00253563	0.03945836
FAM149A	*CYP4V2*	4	Microglia/Macrophage*,MatureAstrocytes,FetalAstrocytes,Neurons,Oligodendrocytes	6	0	6	0	0.03135371	0.08962952	0.007552137	0.02812527
FAT1	*CYP4V2*	4	FetalAstrocytes*,MatureAstrocytes*,Neurons,Oligodendrocytes,Endothelial	9	9	0	0	NA	NA	0.005185649	0.02403027
FLJ38576	*CYP4V2*	4	Microglia/Macrophage	NA	NA	NA	NA	NA	NA	NA	NA
KLKB1	*CYP4V2*	4	MatureAstrocytes	8	7	1	0	0.00218066	0.01944021	4.69044E-05	0.007435745
LOC285441	*CYP4V2*	4	MatureAstrocytes	NA	NA	NA	NA	NA	NA	NA	NA
MTNR1A	*CYP4V2*	4	NA	1	1	0	0	NA	NA	0.000952446	0.01123799
SORBS2	*CYP4V2*	4	FetalAstrocytes,Endothelial,Neurons	9	2	7	0	NA	NA	0.0106827	0.05082933
TLR3	*CYP4V2*	4	Microglia/Macrophage*,Endothelial,Oligodendrocytes,MatureAstrocytes	3	3	0	0	0.00109993	0.01977908	0.002002931	0.03023616
U4	*CYP4V2*	4	NA	NA	NA	NA	NA	NA	NA	NA	NA
U6	*CYP4V2*	4	NA	NA	NA	NA	NA	NA	NA	NA	NA
UNQ2560	*CYP4V2*	4	NA	NA	NA	NA	NA	NA	NA	NA	NA
C4orf47	*CYP4V2*	4	NA	NA	NA	NA	NA	NA	NA	0.004989443	0.03284341
AGPAT3	*C21orf33,ICOSLG*	21	MatureAstrocytes,Neurons,Oligodendrocytes,FetalAstrocytes	3	1	2	0	NA	NA	0.03575969	0.06491277
AIRE	*C21orf33,ICOSLG*	21	NA	3	3	0	0	NA	NA	0.04376239	0.07574331
C21orf2	*C21orf33,ICOSLG*	21	FetalAstrocytes	NA	NA	NA	NA	NA	NA	NA	NA
C21orf33	*C21orf33,ICOSLG*	21	MatureAstrocytes*,Oligodendrocytes*,Neurons*,FetalAstrocytes,Microglia/Macrophage,Endothelial	NA	NA	NA	NA	NA	NA	NA	NA
C21orf90	*C21orf33,ICOSLG*	21	NA	NA	NA	NA	NA	NA	NA	0.0111082	0.02994254
CSTB	*C21orf33,ICOSLG*	21	MatureAstrocytes*,Neurons*,Oligodendrocytes*,Microglia/Macrophage*,FetalAstrocytes,Endothelial	1	0	1	0	0.0221216	0.05506822	0.005214101	0.02015999
DNMT3L	*C21orf33,ICOSLG*	21	NA	NA	NA	NA	NA	NA	NA	NA	NA
DQ588725	*C21orf33,ICOSLG*	21	NA	NA	NA	NA	NA	NA	NA	NA	NA
DQ590668	*C21orf33,ICOSLG*	21	NA	NA	NA	NA	NA	NA	NA	NA	NA
DQ598454	*C21orf33,ICOSLG*	21	NA	NA	NA	NA	NA	NA	NA	NA	NA
DQ599834	*C21orf33,ICOSLG*	21	NA	NA	NA	NA	NA	NA	NA	NA	NA
DQ601137	*C21orf33,ICOSLG*	21	NA	NA	NA	NA	NA	NA	NA	NA	NA
ICOSLG	*C21orf33,ICOSLG*	21	Microglia/Macrophage	1	1	0	0	NA	NA	0.0479285	0.07373075
KRTAP10-1	*C21orf33,ICOSLG*	21	NA	NA	NA	NA	NA	NA	NA	NA	NA
KRTAP10-10	*C21orf33,ICOSLG*	21	NA	NA	NA	NA	NA	NA	NA	NA	NA
KRTAP10-11	*C21orf33,ICOSLG*	21	NA	NA	NA	NA	NA	NA	NA	NA	NA
KRTAP10-12	*C21orf33,ICOSLG*	21	NA	NA	NA	NA	NA	NA	NA	NA	NA
KRTAP10-2	*C21orf33,ICOSLG*	21	NA	NA	NA	NA	NA	NA	NA	NA	NA
KRTAP10-3	*C21orf33,ICOSLG*	21	NA	NA	NA	NA	NA	NA	NA	NA	NA
KRTAP10-4	*C21orf33,ICOSLG*	21	NA	NA	NA	NA	NA	NA	NA	NA	NA
KRTAP10-5	*C21orf33,ICOSLG*	21	NA	NA	NA	NA	NA	NA	NA	NA	NA
KRTAP10-6	*C21orf33,ICOSLG*	21	NA	NA	NA	NA	NA	NA	NA	NA	NA
KRTAP10-7	*C21orf33,ICOSLG*	21	NA	NA	NA	NA	NA	NA	NA	NA	NA
KRTAP10-8	*C21orf33,ICOSLG*	21	NA	NA	NA	NA	NA	NA	NA	NA	NA

KRTAP10-9	*C21orf33,ICOSLG*	21	NA	NA	NA	NA	NA	NA	NA	NA	NA
KRTAP12-1	*C21orf33,ICOSLG*	21	NA	NA	NA	NA	NA	NA	NA	NA	NA
KRTAP12-2	*C21orf33,ICOSLG*	21	NA	NA	NA	NA	NA	NA	NA	NA	NA
KRTAP12-3	*C21orf33,ICOSLG*	21	NA	NA	NA	NA	NA	NA	NA	NA	NA
KRTAP12-4	*C21orf33,ICOSLG*	21	NA	NA	NA	NA	NA	NA	NA	NA	NA
LOC284837	*C21orf33,ICOSLG*	21	NA	NA	NA	NA	NA	NA	NA	NA	NA
LRRC3	*C21orf33,ICOSLG*	21	NA	NA	NA	NA	NA	NA	NA	0.00278988	0.01670277
LRRC3-AS1	*C21orf33,ICOSLG*	21	NA	NA	NA	NA	NA	NA	NA	0.01305419	0.0425205
PDXK	*C21orf33,ICOSLG*	21	MatureAstrocytes,Neurons,Endothelial	5	3	2	0	NA	NA	NA	NA
PFKL	*C21orf33,ICOSLG*	21	FetalAstrocytes	NA	NA	NA	NA	NA	NA	0.03995329	0.07600761
PWP2	*C21orf33,ICOSLG*	21	NA	1	1	0	0	NA	NA	NA	NA
RRP1	*C21orf33,ICOSLG*	21	FetalAstrocytes	NA	NA	NA	NA	NA	NA	0.005592472	0.02326094
TRAPPC10	*C21orf33,ICOSLG*	21	Microglia/Macrophage,Neurons,Oligodendrocytes,FetalAstrocytes,MatureAstrocytes	NA	NA	NA	NA	NA	NA	0.0186256	0.04433238
TRPM2	*C21orf33,ICOSLG*	21	NA	2	0	2	0	NA	NA	NA	NA
TRPM2-AS	*C21orf33,ICOSLG*	21	NA	NA	NA	NA	NA	NA	NA	NA	NA
TSPEAR	*C21orf33,ICOSLG*	21	NA	NA	NA	NA	NA	0.0276463	0.1173633	NA	NA
TSPEAR-AS1	*C21orf33,ICOSLG*	21	NA	NA	NA	NA	NA	NA	NA	0.03589631	0.06506584
HSF2BP	*C21orf33,ICOSLG*	21	NA	1	1	0	0	0.03786589	0.1188581	0.0138905	0.03622745
RRP1B	*C21orf33,ICOSLG*	21	FetalAstrocytes,Neurons,Endothelial	1	1	0	0	0.0429562	0.06843161	0.006256578	0.02551044
UBE2G2	*C21orf33,ICOSLG*	21	FetalAstrocytes,MatureAstrocytes,Neurons,Oligodendrocytes,Microglia/Macrophage,Endothelial	NA	NA	NA	NA	NA	NA	0.0101961	0.03228033
C21orf67	*C21orf33,ICOSLG*	21	NA	NA	NA	NA	NA	NA	NA	0.009335018	0.02971858
LINC00971	*LINC00971*	3	NA	NA	NA	NA	NA	NA	NA	NA	NA
CADM2	*LINC00971*	3	MatureAstrocytes*,Neurons*,Oligodendrocytes*,FetalAstrocytes,Endothelial	5	0	5	0	NA	NA	0.001500799	0.009292013
SLITRK1	*SLITRK1*	13	Neurons,Oligodendrocytes	5	1	4	0	NA	NA	0.0212201	0.06841025
LINC00333	*SLITRK1*	13	NA	NA	NA	NA	NA	NA	NA	NA	NA
C2orf80	*C2orf80,IDH1*	2	Neurons*,Oligodendrocytes	12	0	12	0	NA	NA	0.00241572	0.02103459
CCNYL1	*C2orf80,IDH1*	2	FetalAstrocytes,MatureAstrocytes,Neurons,Oligodendrocytes,Microglia/Macrophage,Endothelial	1	1	0	0	NA	NA	0.0181751	0.05618972
CRYGA	*C2orf80,IDH1*	2	NA	1	1	0	0	NA	NA	NA	NA
CRYGB	*C2orf80,IDH1*	2	NA	1	0	1	0	NA	NA	NA	NA
CRYGC	*C2orf80,IDH1*	2	NA	NA	NA	NA	NA	NA	NA	NA	NA
CRYGD	*C2orf80,IDH1*	2	FetalAstrocytes	1	1	0	0	NA	NA	0.0161334	0.05750588
FZD5	*C2orf80,IDH1*	2	FetalAstrocytes	3	3	0	0	NA	NA	0.0309839	0.06655298
IDH1	*C2orf80,IDH1*	2	FetalAstrocytes,Microglia/Macrophage,MatureAstrocytes,Oligodendrocytes,Neurons	2	0	2	0	NA	NA	0.007434959	0.03330903
IDH1-AS1	*C2orf80,IDH1*	2	NA	NA	NA	NA	NA	NA	NA	6.48868E-05	0.008377588
JA429503	*C2orf80,IDH1*	2	NA	NA	NA	NA	NA	NA	NA	NA	NA
LOC100507443	*C2orf80,IDH1*	2	NA	NA	NA	NA	NA	NA	NA	NA	NA
MIR4775	*C2orf80,IDH1*	2	NA	NA	NA	NA	NA	NA	NA	NA	NA
Mir_548	*C2orf80,IDH1*	2	NA	NA	NA	NA	NA	NA	NA	NA	NA
PIKFYVE	*C2orf80,IDH1*	2	MatureAstrocytes,Neurons,FetalAstrocytes,Microglia/Macrophage,Oligodendrocytes,Endothelial	2	1	1	0	0.00232473	0.02237377	0.002468939	0.02265926
PLEKHM3	*C2orf80,IDH1*	2	Neurons,Oligodendrocytes,MatureAstrocytes,Microglia/Macrophage,Endothelial,FetalAstrocytes	1	0	1	0	NA	NA	0.0103876	0.02991419
PTH2R	*C2orf80,IDH1*	2	Neurons	4	0	4	0	NA	NA	0.008929127	0.03633748
ALG3	*MAGEF1,LOC101928992*	3	FetalAstrocytes,MatureAstrocytes	NA	NA	NA	NA	NA	NA	0.02722891	0.06037791
C3orf70	*MAGEF1,LOC101928992*	3	FetalAstrocytes,MatureAstrocytes,Neurons,Oligodendrocytes,Microglia/Macrophage	0	0	0	0	NA	NA	0.04598209	0.06679984
CAMK2N2	*MAGEF1,LOC101928992*	3	Neurons,Endothelial	NA	NA	NA	NA	NA	NA	0.01059	0.03614694
CHRD	*MAGEF1,LOC101928992*	3	NA	1	0	1	0	0.0115377	0.02654106	0.0425987	0.09908303
CLCN2	*MAGEF1,LOC101928992*	3	NA	2	1	1	0	NA	NA	NA	NA
ECE2	*MAGEF1,LOC101928992*	3	Neurons	3	2	1	0	NA	NA	NA	NA
EHHADH	*MAGEF1,LOC101928992*	3	MatureAstrocytes	NA	NA	NA	NA	NA	NA	0.009567481	0.03414038
EHHADH-AS1	*MAGEF1,LOC101928992*	3	NA	NA	NA	NA	NA	NA	NA	NA	NA
EIF4G1	*MAGEF1,LOC101928992*	3	FetalAstrocytes*,MatureAstrocytes,Neurons,Oligodendrocytes,Microglia/Macrophage,Endothelial	NA	NA	NA	NA	NA	NA	0.0101147	0.03504143
EPHB3	*MAGEF1,LOC101928992*	3	FetalAstrocytes	2	0	2	0	0.04061578	0.0756772	0.003739471	0.02437574
FAM131A	*MAGEF1,LOC101928992*	3	FetalAstrocytes,Neurons	9	1	7	1	NA	NA	0.02383299	0.04874397
LOC101928992	*MAGEF1,LOC101928992*	3	NA	NA	NA	NA	NA	NA	NA	NA	NA
MAGEF1	*MAGEF1,LOC101928992*	3	Neurons*,FetalAstrocytes,MatureAstrocytes,Oligodendrocytes,Endothelial,Microglia/Macrophage	6	0	6	0	NA	NA	NA	NA
POLR2H	*MAGEF1,LOC101928992*	3	FetalAstrocytes*,MatureAstrocytes*,Neurons*,Microglia/Macrophage*,Endothelial*,Oligodendrocytes	1	1	0	0	NA	NA	0.0381033	0.09415976
PSMD2	*MAGEF1,LOC101928992*	3	FetalAstrocytes,MatureAstrocytes,Neurons,Oligodendrocytes,Microglia/Macrophage,Endothelial	1	1	0	0	NA	NA	NA	NA
SNORD66	*MAGEF1,LOC101928992*	3	NA	NA	NA	NA	NA	NA	NA	NA	NA
THPO	*MAGEF1,LOC101928992*	3	NA	NA	NA	NA	NA	NA	NA	0.04540571	0.1020861
TRNA_Asp	*MAGEF1,LOC101928992*	3	NA	NA	NA	NA	NA	NA	NA	NA	NA
VPS8	*MAGEF1,LOC101928992*	3	Neurons,MatureAstrocytes,Oligodendrocytes,Microglia/Macrophage,FetalAstrocytes,Endothelial	NA	NA	NA	NA	NA	NA	0.00692571	0.03586173
VWA5B2	*MAGEF1,LOC101928992*	3	NA	4	0	4	0	NA	NA	NA	NA
MIR1224	*MAGEF1,LOC101928992*	3	NA	NA	NA	NA	NA	NA	NA	0.04368899	0.06436506

PARL	*MAGEF1,LOC101928992*	3	FetalAstrocytes,MatureAstrocytes,Neurons,Oligodendrocytes,Microglia/Macrophage,Endothelial	4	3	1	0	0.02424359	0.05466969	0.0155895	0.03082056
ABCC5	*MAGEF1,LOC101928992*	3	Neurons*,FetalAstrocytes,MatureAstrocytes,Oligodendrocytes,Microglia/Macrophage	3	0	3	0	NA	NA	0.003625709	0.01287403
MIR4801	*HTN1*	4	NA	NA	NA	NA	NA	NA	NA	NA	NA
NWD2	*HTN1*	4	Neurons	NA	NA	NA	NA	NA	NA	NA	NA
ARAP2	*HTN1*	4	MatureAstrocytes*,Oligodendrocytes*,FetalAstrocytes,Neurons,Microglia/Macrophage	3	0	3	0	NA	NA	0.002682461	0.01363795
DTHD1	*HTN1*	4	FetalAstrocytes	NA	NA	NA	NA	NA	NA	0.011436	0.0297504
AMTN	*HTN1*	4	Microglia/Macrophage	NA	NA	NA	NA	NA	NA	NA	NA
C4orf40	*HTN1*	4	NA	NA	NA	NA	NA	NA	NA	NA	NA
CABS1	*HTN1*	4	NA	NA	NA	NA	NA	NA	NA	NA	NA
CSN1S1	*HTN1*	4	NA	2	0	1	1	NA	NA	0.02970931	0.09818479
CSN1S2AP	*HTN1*	4	NA	NA	NA	NA	NA	NA	NA	NA	NA
CSN1S2BP	*HTN1*	4	NA	NA	NA	NA	NA	NA	NA	NA	NA
CSN2	*HTN1*	4	NA	NA	NA	NA	NA	NA	NA	NA	NA
CSN3	*HTN1*	4	NA	1	0	1	0	NA	NA	NA	NA
FDCSP	*HTN1*	4	NA	NA	NA	NA	NA	NA	NA	NA	NA
HTN1	*HTN1*	4	NA	NA	NA	NA	NA	NA	NA	NA	NA
HTN3	*HTN1*	4	NA	NA	NA	NA	NA	NA	NA	NA	NA
MUC7	*HTN1*	4	NA	NA	NA	NA	NA	NA	NA	0.006008013	0.05867084
ODAM	*HTN1*	4	NA	NA	NA	NA	NA	NA	NA	NA	NA
PROL1	*HTN1*	4	NA	NA	NA	NA	NA	NA	NA	NA	NA
PRR27	*HTN1*	4	NA	NA	NA	NA	NA	NA	NA	NA	NA
SMR3A	*HTN1*	4	NA	NA	NA	NA	NA	NA	NA	NA	NA
SMR3B	*HTN1*	4	NA	NA	NA	NA	NA	NA	NA	NA	NA
STATH	*HTN1*	4	NA	NA	NA	NA	NA	NA	NA	NA	NA
SULT1B1	*HTN1*	4	NA	3	0	3	0	NA	NA	0.000464812	0.0194774
SULT1E1	*HTN1*	4	NA	3	0	3	0	NA	NA	NA	NA
UGT2A1	*HTN1*	4	NA	NA	NA	NA	NA	NA	NA	NA	NA
UGT2A2	*HTN1*	4	NA	NA	NA	NA	NA	NA	NA	NA	NA
CRSF1	*HTN1*	4	NA	NA	NA	NA	NA	NA	NA	NA	NA
UTP3	*HTN1*	4	FetalAstrocytes*,Neurons*,Microglia/Macrophage*,MatureAstrocytes,Oligodendrocytes,Endothelial	6	0	6	0	NA	NA	0.002628832	0.03170488
DCK	*HTN1*	4	FetalAstrocytes,MatureAstrocytes,Neurons,Oligodendrocytes,Microglia/Macrophage	6	0	6	0	NA	NA	0.00151667	0.01583722
MOB1B	*HTN1*	4	Microglia/Macrophage*,FetalAstrocytes,MatureAstrocytes,Neurons,Oligodendrocytes,Endothelial	1	1	0	0	0.01038139	0.04639694	0.0113066	0.04960198
ATP5B	*HSD17B6,SDR9C7*	12	Neurons*,MatureAstrocytes*,Microglia/Macrophage*,Oligodendrocytes*,FetalAstrocytes*,Endothelial*	15	1	14	0	NA	NA	0.005917163	0.05103623
BAZ2A	*HSD17B6,SDR9C7*	12	FetalAstrocytes,MatureAstrocytes,Neurons,Oligodendrocytes,Microglia/Macrophage,Endothelial	4	0	4	0	NA	NA	0.0177892	0.06174366
BC059370	*HSD17B6,SDR9C7*	12	NA	NA	NA	NA	NA	NA	NA	NA	NA
DQ590166	*HSD17B6,SDR9C7*	12	NA	NA	NA	NA	NA	NA	NA	NA	NA
GLS2	*HSD17B6,SDR9C7*	12	Neurons	9	0	8	1	0.003593591	0.03804135	0.02022129	0.155425
GPR182	*HSD17B6,SDR9C7*	12	NA	NA	NA	NA	NA	NA	NA	0.03018812	0.4587216
HSD17B6	*HSD17B6,SDR9C7*	12	MatureAstrocytes*,Neurons,Oligodendrocytes	5	2	3	0	NA	NA	0.00167252	0.1325787
KIAA1002	*HSD17B6,SDR9C7*	12	NA	NA	NA	NA	NA	NA	NA	NA	NA
LRP1	*HSD17B6,SDR9C7*	12	MatureAstrocytes,FetalAstrocytes,Microglia/Macrophage,Endothelial	0	0	0	0	NA	NA	0.03310618	0.1454531
MIP	*HSD17B6,SDR9C7*	12	NA	NA	NA	NA	NA	NA	NA	0.00222475	0.07998036
MIR1228	*HSD17B6,SDR9C7*	12	NA	NA	NA	NA	NA	NA	NA	NA	NA
MYO1A	*HSD17B6,SDR9C7*	12	NA	NA	NA	NA	NA	NA	NA	0.01856491	0.04974075
NAB2	*HSD17B6,SDR9C7*	12	Microglia/Macrophage,FetalAstrocytes	2	2	0	0	0.005663729	0.03188447	0.0072254	0.03216068
NACA	*HSD17B6,SDR9C7*	12	Oligodendrocytes*,Microglia/Macrophage*,Neurons*,FetalAstrocytes*,MatureAstrocytes*,Endothelial*	4	1	3	0	NA	NA	0.0373797	0.4372618
NDUFA4L2	*HSD17B6,SDR9C7*	12	Endothelial	5	4	1	0	NA	NA	NA	NA
NXPH4	*HSD17B6,SDR9C7*	12	NA	3	3	0	0	NA	NA	0.0131798	0.186454
PRIM1	*HSD17B6,SDR9C7*	12	FetalAstrocytes,Neurons,Oligodendrocytes,Microglia/Macrophage,MatureAstrocytes	NA	NA	NA	NA	NA	NA	0.000173519	0.02701317
PTGES3	*HSD17B6,SDR9C7*	12	Neurons*,Oligodendrocytes*,Microglia/Macrophage*,FetalAstrocytes*,MatureAstrocytes,Endothelial	4	1	3	0	NA	NA	0.0105087	0.03789839
R3HDM2	*HSD17B6,SDR9C7*	12	FetalAstrocytes*,MatureAstrocytes,Neurons,Oligodendrocytes,Microglia/Macrophage,Endothelial	2	2	0	0	0.03188409	0.4578529	0.02750749	0.2142083
RBMS2	*HSD17B6,SDR9C7*	12	FetalAstrocytes,MatureAstrocytes,Microglia/Macrophage,Endothelial	1	1	0	0	NA	NA	0.003147581	0.2007427
RDH16	*HSD17B6,SDR9C7*	12	NA	NA	NA	NA	NA	0.0377466	0.1503	0.003579018	0.05337248
SDR9C7	*HSD17B6,SDR9C7*	12	NA	NA	NA	NA	NA	NA	NA	0.0143727	0.179096
SHMT2	*HSD17B6,SDR9C7*	12	FetalAstrocytes,MatureAstrocytes,Neurons,Oligodendrocytes,Endothelial	4	4	0	0	NA	NA	0.03435319	0.1935344
SNORD59A	*HSD17B6,SDR9C7*	12	NA	NA	NA	NA	NA	NA	NA	NA	NA
SNORD59B	*HSD17B6,SDR9C7*	12	NA	NA	NA	NA	NA	NA	NA	NA	NA
SPRYD4	*HSD17B6,SDR9C7*	12	FetalAstrocytes,MatureAstrocytes,Oligodendrocytes,Microglia/Macrophage	3	3	0	0	NA	NA	0.0150504	0.1424193
STAC3	*HSD17B6,SDR9C7*	12	NA	NA	NA	NA	NA	NA	NA	0.008508203	0.08124034
STAT6	*HSD17B6,SDR9C7*	12	Microglia/Macrophage,Endothelial,MatureAstrocytes	6	2	4	0	NA	NA	NA	NA
TAC3	*HSD17B6,SDR9C7*	12	Neurons*,Endothelial	4	0	4	0	NA	NA	0.002849189	0.06458654
TIMELESS	*HSD17B6,SDR9C7*	12	FetalAstrocytes	2	0	2	0	NA	NA	0.0136564	0.1715554

TMEM194A	*HSD17B6,SDR9C7*	12	Microglia/Macrophage,FetalAstrocytes,MatureAstrocytes,Neurons,Oligodendrocytes	2	1	1	0	NA	NA	0.000149217	0.01542551
ZBTB39	*HSD17B6,SDR9C7*	12	FetalAstrocytes	NA	NA	NA	NA	0.0422855	0.3672971	0.0119194	0.07581507
LPHN2	*LPHN2*	1	Neurons*,FetalAstrocytes,Endothelial	3	2	1	0	NA	NA	0.006424668	0.02142006
ADI1	*LINC01250*	2	MatureAstrocytes*,FetalAstrocytes,Neurons,Oligodendrocytes,Microglia/Macrophage,Endothelial	2	1	1	0	NA	NA	0.0177489	0.05635992
LINC01250	*LINC01250*	2	NA	NA	NA	NA	NA	NA	NA	NA	NA
RNASEH1	*LINC01250*	2	FetalAstrocytes,MatureAstrocytes,Neurons,Oligodendrocytes,Microglia/Macrophage	4	4	0	0	NA	NA	0.005677189	0.04091087
TRAPPC12	*LINC01250*	2	FetalAstrocytes,MatureAstrocytes	3	0	3	0	NA	NA	0.011023	0.03879543
TSSC1	*LINC01250*	2	Neurons,FetalAstrocytes,MatureAstrocytes	3	3	0	0	NA	NA	0.03781211	0.07000966
ALLC	*LINC01250*	2	NA	2	0	2	0	NA	NA	0.00472295	0.03261152
RPS7	*LINC01250*	2	FetalAstrocytes*,MatureAstrocytes,Oligodendrocytes,Microglia/Macrophage,Endothelial	NA	NA	NA	NA	NA	NA	0.002261259	0.02001135
ATP6V1C2	*KCNF1,FLJ33534*	2	FetalAstrocytes	NA	NA	NA	NA	NA	NA	0.006346192	0.02715962
AX746649	*KCNF1,FLJ33534*	2	NA	NA	NA	NA	NA	NA	NA	NA	NA
C2orf50	*KCNF1,FLJ33534*	2	FetalAstrocytes	2	1	1	0	NA	NA	0.0170999	0.03847193
E2F6	*KCNF1,FLJ33534*	2	MatureAstrocytes*,FetalAstrocytes,Neurons,Oligodendrocytes,Microglia/Macrophage	1	1	0	0	NA	NA	0.000629197	0.008209331
FLJ33534	*KCNF1,FLJ33534*	2	NA	3	2	0	1	NA	NA	NA	NA
KCNF1	*KCNF1,FLJ33534*	2	NA	10	0	10	0	NA	NA	0.008151703	0.05394396
LINC00570	*KCNF1,FLJ33534*	2	NA	NA	NA	NA	NA	NA	NA	NA	NA
LOC101929733	*KCNF1,FLJ33534*	2	NA	NA	NA	NA	NA	NA	NA	NA	NA
NOL10	*KCNF1,FLJ33534*	2	FetalAstrocytes,MatureAstrocytes,Neurons,Oligodendrocytes,Microglia/Macrophage,Endothelial	NA	NA	NA	NA	NA	NA	0.003228419	0.0203919
PDIA6	*KCNF1,FLJ33534*	2	Neurons*,MatureAstrocytes*,FetalAstrocytes*,Oligodendrocytes*,Microglia/Macrophage*,Endothelial	7	5	2	0	NA	NA	0.03240921	0.06292947
PQLC3	*KCNF1,FLJ33534*	2	Microglia/Macrophage,Endothelial,Oligodendrocytes,MatureAstrocytes,FetalAstrocytes,Neurons	0	0	0	0	NA	NA	0.0234169	0.05759389
ROCK2	*KCNF1,FLJ33534*	2	Neurons*,FetalAstrocytes*,Microglia/Macrophage,MatureAstrocytes,Endothelial,Oligodendrocytes	7	2	5	0	NA	NA	0.00831231	0.02576358
CYS1	*KCNF1,FLJ33534*	2	NA	3	0	3	0	NA	NA	0.02816689	0.05212099
PRM2	*KCNF1,FLJ33534*	2	NA	NA	NA	NA	NA	NA	NA	NA	NA
NTS2R	*KCNF1,FLJ33534*	2	NA	NA	NA	NA	NA	NA	NA	NA	NA
GREB1	*KCNF1,FLJ33534*	2	MatureAstrocytes,Neurons	4	3	1	0	NA	NA	0.013131	0.03305501

Gene	Locus	Chr	Pathway	Non-APOE locus pathway
APOC1	*APOE*	19	GO_NEGATIVE_REGULATION_OF_LIPID_METABOLIC_PROCESS	NA
			GO_ORGANOPHOSPHATE_ESTER_TRANSPORT	
			GO_REGULATION_OF_RECEPTOR_MEDIATED_ENDOCYTOSIS	
			GO_NEGATIVE_REGULATION_OF_LIPID_TRANSPORT	
			GO_PHOSPHOLIPID_TRANSPORT	
			GO_NEGATIVE_REGULATION_OF_STEROL_TRANSPORT	
			GO_PHOSPHOLIPID_EFFLUX	
APOC1P1	*APOE*	19	NA	NA
APOC2	*APOE*	19	GO_NEGATIVE_REGULATION_OF_LIPID_METABOLIC_PROCESS	NA
			GO_ORGANOPHOSPHATE_ESTER_TRANSPORT	
			GO_REGULATION_OF_RECEPTOR_MEDIATED_ENDOCYTOSIS	
			GO_NEGATIVE_REGULATION_OF_LIPID_TRANSPORT	
			GO_PHOSPHOLIPID_TRANSPORT	
			GO_NEGATIVE_REGULATION_OF_STEROL_TRANSPORT	
			GO_PHOSPHOLIPID_EFFLUX	
APOC4	*APOE*	19	NA	NA
APOC4-APOC2	*APOE*	19	NA	NA
APOE	*APOE*	19	GO_NEGATIVE_REGULATION_OF_LIPID_METABOLIC_PROCESS	NA
			GO_REGULATION_OF_DEVELOPMENTAL_GROWTH	
			GO_REGULATION_OF_CELL_MORPHOGENESIS_INVOLVED_IN_DIFFERENTIATION	
			GO_MODULATION_BY_HOST_OF_VIRAL_PROCESS	
			GO_REGULATION_OF_GROWTH	
			GO_ORGANOPHOSPHATE_ESTER_TRANSPORT	
			GO_ORGANIC_HYDROXY_COMPOUND_CATABOLIC_PROCESS	
			GO_MAINTENANCE_OF_LOCATION_IN_CELL	
			GO_REGULATION_OF_CELL_GROWTH	
			GO_NEGATIVE_REGULATION_OF_LIPID_TRANSPORT	
			GO_PHOSPHOLIPID_TRANSPORT	
			GO_POSITIVE_REGULATION_OF_PROTEOLYSIS	
			GO_NEGATIVE_REGULATION_OF_STEROL_TRANSPORT	
			GO_REGULATION_OF_EXTENT_OF_CELL_GROWTH	
			GO_REGULATION_OF_PROTEOLYSIS	
			GO_PHOSPHOLIPID_EFFLUX	
			GO_ALCOHOL_CATABOLIC_PROCESS	
			GO_RECEPTOR_CLUSTERING	
			GO_BETA_AMYLOID_BINDING	
BCAM	*APOE*	19	NA	NA
BCL3	*APOE*	19	GO_MAINTENANCE_OF_LOCATION_IN_CELL	NA
			GO_SIGNAL_TRANSDUCTION_IN_RESPONSE_TO_DNA_DAMAGE	
			GO_RESPONSE_TO_PROTOZOAN	
			GO_REGULATION_OF_INTRACELLULAR_PROTEIN_TRANSPORT	
			GO_REGULATION_OF_PROTEIN_TARGETING	
BLOC1S3	*APOE*	19	GO_SYNAPTIC_VESICLE_LOCALIZATION	NA
			GO_VESICLE_LOCALIZATION	
			GO_CYTOSOLIC_TRANSPORT	
CBLC	*APOE*	19	GO_REGULATION_OF_PROTEIN_TYROSINE_KINASE_ACTIVITY	NA
CD3EAP	*APOE*	19	NA	NA
CEACAM16	*APOE*	19	GO_ACTIN_BASED_CELL_PROJECTION	NA
CEACAM19	*APOE*	19	NA	NA
CEACAM20	*APOE*	19	NA	NA
CEACAM22P	*APOE*	19	NA	NA
CKM	*APOE*	19	NA	NA
CLASRP	*APOE*	19	NA	NA
CLPTM1	*APOE*	19	NA	NA
ERCC1	*APOE*	19	GO_GROWTH	NA
			GO_CELL_AGING	
			GO_DEVELOPMENTAL_GROWTH	
ERCC2	*APOE*	19	GO_EPITHELIUM_DEVELOPMENT	NA
			GO_GROWTH	
			GO_DEVELOPMENTAL_GROWTH	
			GO_METAL_CLUSTER_BINDING	
			GO_4_IRON_4_SULFUR_CLUSTER_BINDING	
EXOC3L2	*APOE*	19	NA	NA
GEMIN7	*APOE*	19	NA	NA
IGSF23	*APOE*	19	NA	NA
KLC3	*APOE*	19	NA	NA
MARK4	*APOE*	19	NA	NA
MIR4531	*APOE*	19	NA	NA

MIR8085	*APOE*	19	NA	NA
NKPD1	*APOE*	19	NA	NA
PPP1R13L	*APOE*	19	GO_HEART_MORPHOGENESIS<br>GO_GROWTH<br>GO_DEVELOPMENTAL_GROWTH	NA
PPP1R37	*APOE*	19	NA	NA
PVR	*APOE*	19	NA	NA
PVRL2	*APOE*	19	GO_MITOCHONDRION_ORGANIZATION<br>GO_MITOCHONDRION_LOCALIZATION	NA
RELB	*APOE*	19	NA	NA
TOMM40	*APOE*	19	GO_MITOCHONDRION_ORGANIZATION<br>GO_INTRINSIC_COMPONENT_OF_ORGANELLE_MEMBRANE<br>GO_PORE_COMPLEX<br>GO_MITOCHONDRIAL_MEMBRANE_PART	NA
TRAPPC6A	*APOE*	19	GO_VESICLE_LOCALIZATION	NA
ZNF180	*APOE*	19	NA	NA
ZNF229	*APOE*	19	NA	NA
ZNF296	*APOE*	19	NA	NA
ADCY8	*ADCY8,EFR3A*	8	GO_ADENYLATE_CYCLASE_ACTIVATING_G_PROTEIN_COUPLED_RECEPTOR_SIGNALING_PATHWAY	GO_ADENYLATE_CYCLASE_ACTIVATING_G_PROTEIN_COUPLED_RECEPTOR_SIGNALING_PATHWAY
EFR3A	*ADCY8,EFR3A*	8	NA	NA
7SK	*ADCY8,EFR3A*	8	NA	NA
RAP2B	*RAP2B,C3orf79*	3	GO_REGULATION_OF_PROTEIN_TYROSINE_KINASE_ACTIVITY<br>GO_EPITHELIUM_DEVELOPMENT<br>GO_SMALL_GTPASE_MEDIATED_SIGNAL_TRANSDUCTION<br>GO_GDP_BINDING	GO_SMALL_GTPASE_MEDIATED_SIGNAL_TRANSDUCTION
C3orf79	*RAP2B,C3orf79*	3	NA	NA
AK092619ARHGEF26	*RAP2B,C3orf79*	3	NA	NA
ARHGEF26-AS1	*RAP2B,C3orf79*	3	NA	NA
DHX36	*RAP2B,C3orf79*	3	NA	NA
AK091624	*DAPK2*	15	NA	NA
AL137522	*DAPK2*	15	NA	NA
CSNK1G1	*DAPK2*	15	GO_PROTEIN_AUTOPHOSPHORYLATION	GO_PROTEIN_AUTOPHOSPHORYLATION
DAPK2	*DAPK2*	15	GO_PROTEIN_AUTOPHOSPHORYLATION<br>GO_REGULATION_OF_INTRINSIC_APOPTOTIC_SIGNALING_PATHWAY<br>GO_REGULATION_OF_APOPTOTIC_SIGNALING_PATHWAY	GO_PROTEIN_AUTOPHOSPHORYLATION
FAM96A	*DAPK2*	15	NA	NA
FBXL22	*DAPK2*	15	NA	NA
HERC1	*DAPK2*	15	NA	NA
KIAA0101	*DAPK2*	15	NA	NA
LOC101928988	*DAPK2*	15	NA	NA
Mir_422	*DAPK2*	15	NA	NA
PPIB	*DAPK2*	15	GO_REGULATION_OF_DEVELOPMENTAL_GROWTH<br>GO_MODULATION_BY_HOST_OF_VIRAL_PROCESS<br>GO_REGULATION_OF_GROWTH	NA
SNX1	*DAPK2*	15	GO_PROTEIN_LOCALIZATION_TO_CYTOSKELETON<br>GO_PROTEIN_LOCALIZATION_TO_CENTROSOME<br>GO_ORGANIC_HYDROXY_COMPOUND_CATABOLIC_PROCESS<br>GO_NEGATIVE_REGULATION_OF_ESTABLISHMENT_OF_PROTEIN_LOCALIZATION<br>GO_REGULATION_OF_PROTEOLYSIS<br>GO_ALCOHOL_CATABOLIC_PROCESS<br>GO_CYTOSOLIC_TRANSPORT<br>GO_EXTRINSIC_COMPONENT_OF_PLASMA_MEMBRANE	NA
SNX22	*DAPK2*	15	NA	NA
TRIP4	*DAPK2*	15	GO_TRANSCRIPTION_COACTIVATOR_ACTIVITY	GO_TRANSCRIPTION_COACTIVATOR_ACTIVITY
USP3	*DAPK2*	15	GO_CHROMATIN_MODIFICATION	GO_CHROMATIN_MODIFICATION
USP3-AS1	*DAPK2*	15	NA	NA
LACTB	*DAPK2*	15	NA	NA
RPS27L	*DAPK2*	15	GO_G1_DNA_DAMAGE_CHECKPOINT<br>GO_POSITIVE_REGULATION_OF_PEPTIDASE_ACTIVITY<br>GO_MITOTIC_DNA_INTEGRITY_CHECKPOINT<br>GO_MITOTIC_CELL_CYCLE_CHECKPOINT<br>GO_NEGATIVE_REGULATION_OF_CELL_CYCLE_PHASE_TRANSITION<br>GO_NEGATIVE_REGULATION_OF_CELL_CYCLE_G1_S_PHASE_TRANSITION<br>GO_REGULATION_OF_CYSTEINE_TYPE_ENDOPEPTIDASE_ACTIVITY<br>GO_POSITIVE_REGULATION_OF_PROTEOLYSIS<br>GO_DNA_INTEGRITY_CHECKPOINT<br>GO_CELL_CYCLE_CHECKPOINT<br>GO_REGULATION_OF_PROTEOLYSIS<br>GO_PEPTIDASE_ACTIVATOR_ACTIVITY	GO_G1_DNA_DAMAGE_CHECKPOINT<br>GO_POSITIVE_REGULATION_OF_PEPTIDASE_ACTIVITY<br>GO_MITOTIC_DNA_INTEGRITY_CHECKPOINT<br>GO_MITOTIC_CELL_CYCLE_CHECKPOINT<br>GO_NEGATIVE_REGULATION_OF_CELL_CYCLE_PHASE_TRANSITION<br>GO_NEGATIVE_REGULATION_OF_CELL_CYCLE_G1_S_PHASE_TRANSITION<br>GO_REGULATION_OF_CYSTEINE_TYPE_ENDOPEPTIDASE_ACTIVITY<br>GO_DNA_INTEGRITY_CHECKPOINT<br>GO_CELL_CYCLE_CHECKPOINT<br>GO_PEPTIDASE_ACTIVATOR_ACTIVITY

RAB8B	*DAPK2*	15	GO_SMALL_GTPASE_MEDIATED_SIGNAL_TRANSDUCTION	GO_SMALL_GTPASE_MEDIATED_SIGNAL_TRANSDUCTION
			GO_GTPASE_ACTIVITY	GO_GTPASE_ACTIVITY
			GO_GDP_BINDING	GO_GDP_BINDING
ZNF609	*DAPK2*	15	NA	NA
PLEKHO2	*DAPK2*	15	NA	NA
ANKDD1A	*DAPK2*	15	NA	NA
CYP4V2	*CYP4V2*	4	GO_OXIDOREDUCTASE_ACTIVITY	GO_OXIDOREDUCTASE_ACTIVITY
F11	*CYP4V2*	4	NA	NA
F11-AS1	*CYP4V2*	4	NA	NA
FAM149A	*CYP4V2*	4	NA	NA
FAT1	*CYP4V2*	4	GO_ACTIN_BASED_CELL_PROJECTION	NA
FLJ38576	*CYP4V2*	4	NA	NA
KLKB1	*CYP4V2*	4	GO_POSITIVE_REGULATION_OF_PROTEOLYSIS	NA
			GO_REGULATION_OF_PROTEOLYSIS	
LOC285441	*CYP4V2*	4	NA	NA
MTNR1A	*CYP4V2*	4	GO_NEGATIVE_REGULATION_OF_ESTABLISHMENT_OF_PROTEIN_LOCALIZATION	GO_NEGATIVE_REGULATION_OF_ESTABLISHMENT_OF_PROTEIN_LOCALIZATION
SORBS2	*CYP4V2*	4	GO_CARDIAC_MUSCLE_CELL_DIFFERENTIATION	GO_CARDIAC_MUSCLE_CELL_DIFFERENTIATION
			GO_GROWTH	
			GO_CARDIOCYTE_DIFFERENTIATION	
			GO_DEVELOPMENTAL_CELL_GROWTH	
			GO_DEVELOPMENTAL_GROWTH	
TLR3	*CYP4V2*	4	GO_EXTRINSIC_APOPTOTIC_SIGNALING_PATHWAY	GO_EXTRINSIC_APOPTOTIC_SIGNALING_PATHWAY
			GO_RESPONSE_TO_INTERFERON_BETA	
			GO_POSITIVE_REGULATION_OF_NF_KAPPAB_TRANSCRIPTION_FACTOR_ACTIVITY	
			GO_REGULATION_OF_INTRACELLULAR_PROTEIN_TRANSPORT	
			GO_POSITIVE_REGULATION_OF_SEQUENCE_SPECIFIC_DNA_BINDING_TRANSCRIPTION_FACTOR_ACTIVITY	
			GO_CELLULAR_RESPONSE_TO_DSRNA	
			GO_REGULATION_OF_SEQUENCE_SPECIFIC_DNA_BINDING_TRANSCRIPTION_FACTOR_ACTIVITY	
			GO_CELLULAR_RESPONSE_TO_INTERFERON_BETA	
			GO_REGULATION_OF_PROTEIN_TARGETING	
U4	*CYP4V2*	4	NA	NA
U6	*CYP4V2*	4	NA	NA
UNQ2560	*CYP4V2*	4	NA	NA
C4orf47	*CYP4V2*	4	NA	NA
AGPAT3	*C21orf33,ICOSLG*	21	NA	NA
AIRE	*C21orf33,ICOSLG*	21	NA	NA
C21orf2	*C21orf33,ICOSLG*	21	NA	NA
C21orf33	*C21orf33,ICOSLG*	21	NA	NA
C21orf90	*C21orf33,ICOSLG*	21	NA	NA
CSTB	*C21orf33,ICOSLG*	21	GO_REGULATION_OF_PROTEOLYSIS	NA
DNMT3L	*C21orf33,ICOSLG*	21	NA	NA
DQ588725	*C21orf33,ICOSLG*	21	NA	NA
DQ590668	*C21orf33,ICOSLG*	21	NA	NA
DQ598454	*C21orf33,ICOSLG*	21	NA	NA
DQ599834	*C21orf33,ICOSLG*	21	NA	NA
DQ601137	*C21orf33,ICOSLG*	21	NA	NA
ICOSLG	*C21orf33,ICOSLG*	21	KEGG_INTESTINAL_IMMUNE_NETWORK_FOR_IGA_PRODUCTION	KEGG_INTESTINAL_IMMUNE_NETWORK_FOR_IGA_PRODUCTION
KRTAP10-1	*C21orf33,ICOSLG*	21	NA	NA
KRTAP10-10	*C21orf33,ICOSLG*	21	NA	NA
KRTAP10-11	*C21orf33,ICOSLG*	21	NA	NA
KRTAP10-12	*C21orf33,ICOSLG*	21	NA	NA
KRTAP10-2	*C21orf33,ICOSLG*	21	NA	NA
KRTAP10-3	*C21orf33,ICOSLG*	21	NA	NA
KRTAP10-4	*C21orf33,ICOSLG*	21	NA	NA
KRTAP10-5	*C21orf33,ICOSLG*	21	NA	NA
KRTAP10-6	*C21orf33,ICOSLG*	21	NA	NA
KRTAP10-7	*C21orf33,ICOSLG*	21	NA	NA
KRTAP10-8	*C21orf33,ICOSLG*	21	NA	NA
KRTAP10-9	*C21orf33,ICOSLG*	21	NA	NA
KRTAP12-1	*C21orf33,ICOSLG*	21	NA	NA
KRTAP12-2	*C21orf33,ICOSLG*	21	NA	NA
KRTAP12-3	*C21orf33,ICOSLG*	21	NA	NA
KRTAP12-4	*C21orf33,ICOSLG*	21	NA	NA
LOC284837	*C21orf33,ICOSLG*	21	NA	NA
LRRC3	*C21orf33,ICOSLG*	21	NA	NA
LRRC3-AS1	*C21orf33,ICOSLG*	21	NA	NA
PDXK	*C21orf33,ICOSLG*	21	NA	NA
PFKL	*C21orf33,ICOSLG*	21	GO_PROTEIN_HOMOTETRAMERIZATION	GO_PROTEIN_HOMOTETRAMERIZATION
			GO_NAD_METABOLIC_PROCESS	

			GO_NEGATIVE_REGULATION_OF_ESTABLISHMENT_OF_PROTEIN_LOCALIZATION	
			GO_NUCLEOSIDE_TRIPHOSPHATE_METABOLIC_PROCESS	
			GO_NADH_METABOLIC_PROCESS	
			GO_PROTEIN_HOMOOLIGOMERIZATION	
			GO_GLUCOSE_CATABOLIC_PROCESS	
			KEGG_GLYCOLYSIS_GLUCONEOGENESIS	
			BIOCARTA_GLYCOLYSIS_PATHWAY	
PWP2	*C21orf33,ICOSLG*	21	NA	NA
RRP1	*C21orf33,ICOSLG*	21	NA	NA
TRAPPC10	*C21orf33,ICOSLG*	21	GO_VESICLE_LOCALIZATION	NA
			GO_CYTOSOLIC_TRANSPORT	
TRPM2	*C21orf33,ICOSLG*	21	NA	NA
TRPM2-AS	*C21orf33,ICOSLG*	21	NA	NA
TSPEAR	*C21orf33,ICOSLG*	21	GO_ACTIN_BASED_CELL_PROJECTION	NA
TSPEAR-AS1	*C21orf33,ICOSLG*	21	NA	NA
HSF2BP	*C21orf33,ICOSLG*	21	GO_MITOCHONDRION_ORGANIZATION	NA
RRP1B	*C21orf33,ICOSLG*	21	NA	NA
UBE2G2	*C21orf33,ICOSLG*	21	GO_RESPONSE_TO_INTERFERON_BETA	GO_RESPONSE_TO_INTERFERON_BETA
			GO_NEGATIVE_REGULATION_OF_INTRACELLULAR_PROTEIN_TRANSPORT	GO_NEGATIVE_REGULATION_OF_INTRACELLULAR_PROTEIN_TRANSPORT
			GO_NEGATIVE_REGULATION_OF_ESTABLISHMENT_OF_PROTEIN_LOCALIZATION	GO_NEGATIVE_REGULATION_OF_ESTABLISHMENT_OF_PROTEIN_LOCALIZATION
			GO_REGULATION_OF_INTRACELLULAR_PROTEIN_TRANSPORT	GO_CELLULAR_RESPONSE_TO_INTERFERON_BETA
			GO_CELLULAR_RESPONSE_TO_INTERFERON_BETA	KEGG_PARKINSONS_DISEASE
C21orf67	*C21orf33,ICOSLG*	21	NA	NA
LINC00971	*LINC00971*	3	NA	NA
CADM2	*LINC00971*	3	NA	NA
SLITRK1	*SLITRK1*	13	GO_NEURON_PROJECTION_MORPHOGENESIS	GO_NEURON_PROJECTION_MORPHOGENESIS
			GO_GROWTH	
			GO_DEVELOPMENTAL_GROWTH	
LINC00333	*SLITRK1*	13	NA	NA
C2orf80	*C2orf80,IDH1*	2	NA	NA
CCNYL1	*C2orf80,IDH1*	2	NA	NA
CRYGA	*C2orf80,IDH1*	2	NA	NA
CRYGB	*C2orf80,IDH1*	2	GO_EPITHELIUM_DEVELOPMENT	NA
CRYGC	*C2orf80,IDH1*	2	NA	NA
CRYGD	*C2orf80,IDH1*	2	GO_EPITHELIUM_DEVELOPMENT	NA
FZD5	*C2orf80,IDH1*	2	GO_EPITHELIUM_DEVELOPMENT	GO_MACROMOLECULAR_COMPLEX_DISASSEMBLY
			GO_MACROMOLECULAR_COMPLEX_DISASSEMBLY	GO_CANONICAL_WNT_SIGNALING_PATHWAY
			GO_CANONICAL_WNT_SIGNALING_PATHWAY	GO_ANTERIOR_POSTERIOR_PATTERN_SPECIFICATION
			GO_ANTERIOR_POSTERIOR_PATTERN_SPECIFICATION	GO_PATTERN_SPECIFICATION_PROCESS
			GO_REGULATION_OF_INTRACELLULAR_PROTEIN_TRANSPORT	GO_REGIONALIZATION
			GO_PATTERN_SPECIFICATION_PROCESS	KEGG_BASAL_CELL_CARCINOMA
			GO_REGIONALIZATION	
			GO_REGULATION_OF_PROTEIN_TARGETING	
			GO_BETA_AMYLOID_BINDING	
			KEGG_BASAL_CELL_CARCINOMA	
IDH1	*C2orf80,IDH1*	2	GO_OXIDOREDUCTASE_ACTIVITY	GO_OXIDOREDUCTASE_ACTIVITY
IDH1-AS1	*C2orf80,IDH1*	2	NA	NA
JA429503	*C2orf80,IDH1*	2	NA	NA
LOC100507443	*C2orf80,IDH1*	2	NA	NA
MIR4775	*C2orf80,IDH1*	2	NA	NA
Mir_548	*C2orf80,IDH1*	2	NA	NA
PIKFYVE	*C2orf80,IDH1*	2	GO_REGULATION_OF_VACUOLE_ORGANIZATION	GO_REGULATION_OF_VACUOLE_ORGANIZATION
			GO_CYTOSOLIC_TRANSPORT	
			GO_PHOSPHATIDYLINOSITOL_PHOSPHATE_PHOSPHATASE_ACTIVITY	
			GO_PHOSPHATIDYLINOSITOL_PHOSPHATE_5_PHOSPHATASE_ACTIVITY	
			KEGG_INOSITOL_PHOSPHATE_METABOLISM	
			REACTOME_PI_METABOLISM	
PLEKHM3	*C2orf80,IDH1*	2	NA	NA
PTH2R	*C2orf80,IDH1*	2	NA	NA
ALG3	*MAGEF1,LOC10192899.*	3	NA	NA
C3orf70	*MAGEF1,LOC10192899.*	3	NA	NA
CAMK2N2	*MAGEF1,LOC10192899.*	3	NA	NA
CHRD	*MAGEF1,LOC10192899.*	3	GO_EPITHELIUM_DEVELOPMENT	GO_PATTERN_SPECIFICATION_PROCESS
			GO_PATTERN_SPECIFICATION_PROCESS	GO_REGIONALIZATION
			GO_REGIONALIZATION	
CLCN2	*MAGEF1,LOC10192899.*	3	NA	NA
ECE2	*MAGEF1,LOC10192899.*	3	GO_CARDIOBLAST_DIFFERENTIATION	GO_CARDIOBLAST_DIFFERENTIATION
			GO_CARDIOCYTE_DIFFERENTIATION	
EHHADH	*MAGEF1,LOC10192899.*	3	GO_OXIDOREDUCTASE_ACTIVITY	GO_OXIDOREDUCTASE_ACTIVITY

EHHADH-AS1	*MAGEF1,LOC10192899.*	3	NA	NA
EIF4G1	*MAGEF1,LOC10192899.*	3	NA	NA
EPHB3	*MAGEF1,LOC10192899.*	3	GO_REGULATION_OF_CELL_MORPHOGENESIS_INVOLVED_IN_DIFFERENTIATION	GO_NEURON_PROJECTION_MORPHOGENESIS
			GO_NEURON_PROJECTION_MORPHOGENESIS	
			GO_TUBE_MORPHOGENESIS	
			GO_NEURON_PROJECTION_GUIDANCE	
			GO_PROTEIN_AUTOPHOSPHORYLATION	
			GO_TRANSMEMBRANE_RECEPTOR_PROTEIN_KINASE_ACTIVITY	
			GO_TRANSMEMBRANE_RECEPTOR_PROTEIN_TYROSINE_KINASE_ACTIVITY	
			GO_EPHRIN_RECEPTOR_ACTIVITY	
FAM131A	*MAGEF1,LOC10192899.*	3	NA	NA
LOC101928992	*MAGEF1,LOC10192899.*	3	NA	NA
MAGEF1	*MAGEF1,LOC10192899.*	3	NA	NA
POLR2H	*MAGEF1,LOC10192899.*	3	NA	NA
PSMD2	*MAGEF1,LOC10192899.*	3	GO_POSITIVE_REGULATION_OF_PROTEOLYSIS	NA
			GO_REGULATION_OF_PROTEOLYSIS	
SNORD66	*MAGEF1,LOC10192899.*	3	NA	NA
THPO	*MAGEF1,LOC10192899.*	3	NA	NA
TRNA_Asp	*MAGEF1,LOC10192899.*	3	NA	NA
VPS8	*MAGEF1,LOC10192899.*	3	NA	NA
VWA5B2	*MAGEF1,LOC10192899.*	3	NA	NA
MIR1224	*MAGEF1,LOC10192899.*	3	NA	NA
PARL	*MAGEF1,LOC10192899.*	3	GO_NEGATIVE_REGULATION_OF_INTRINSIC_APOPTOTIC_SIGNALING_PATHWAY	GO_NEGATIVE_REGULATION_OF_INTRINSIC_APOPTOTIC_SIGNALING_PATHWAY
			GO_REGULATION_OF_INTRACELLULAR_PROTEIN_TRANSPORT	GO_REGULATION_OF_INTRINSIC_APOPTOTIC_SIGNALING_PATHWAY
			GO_REGULATION_OF_PROTEOLYSIS	GO_REGULATION_OF_APOPTOTIC_SIGNALING_PATHWAY
			GO_REGULATION_OF_INTRINSIC_APOPTOTIC_SIGNALING_PATHWAY	
			GO_REGULATION_OF_PROTEIN_TARGETING	
			GO_REGULATION_OF_APOPTOTIC_SIGNALING_PATHWAY	
ABCC5	*MAGEF1,LOC10192899.*	3	NA	NA
MIR4801	*HTN1*	4	NA	NA
NWD2	*HTN1*	4	NA	NA
ARAP2	*HTN1*	4	NA	NA
DTHD1	*HTN1*	4	NA	NA
AMTN	*HTN1*	4	NA	NA
C4orf40	*HTN1*	4	NA	NA
CABS1	*HTN1*	4	NA	NA
CSN1S1	*HTN1*	4	NA	NA
CSN1S2AP	*HTN1*	4	NA	NA
CSN1S2BP	*HTN1*	4	NA	NA
CSN2	*HTN1*	4	NA	NA
CSN3	*HTN1*	4	NA	NA
FDCSP	*HTN1*	4	NA	NA
HTN1	*HTN1*	4	NA	NA
HTN3	*HTN1*	4	NA	NA
MUC7	*HTN1*	4	NA	NA
ODAM	*HTN1*	4	NA	NA
PROL1	*HTN1*	4	GO_REGULATION_OF_PROTEOLYSIS	NA
PRR27	*HTN1*	4	NA	NA
SMR3A	*HTN1*	4	NA	NA
SMR3B	*HTN1*	4	NA	NA
STATH	*HTN1*	4	NA	NA
SULT1B1	*HTN1*	4	GO_EPITHELIUM_DEVELOPMENT	GO_TRANSFERASE_ACTIVITY_TRANSFERRING_SULFUR_CONTAINING_GROUPS
			GO_TRANSFERASE_ACTIVITY_TRANSFERRING_SULFUR_CONTAINING_GROUPS	
SULT1E1	*HTN1*	4	GO_TRANSFERASE_ACTIVITY_TRANSFERRING_SULFUR_CONTAINING_GROUPS	GO_TRANSFERASE_ACTIVITY_TRANSFERRING_SULFUR_CONTAINING_GROUPS
UGT2A1	*HTN1*	4	NA	NA
UGT2A2	*HTN1*	4	NA	NA
CRSF1	*HTN1*	4	NA	NA
UTP3	*HTN1*	4	GO_CHROMATIN_MODIFICATION	GO_CHROMATIN_MODIFICATION
DCK	*HTN1*	4	NA	NA
MOB1B	*HTN1*	4	NA	NA
ATP5B	*HSD17B6,SDR9C7*	12	GO_NUCLEOSIDE_TRIPHOSPHATE_METABOLIC_PROCESS	GO_NUCLEOSIDE_TRIPHOSPHATE_METABOLIC_PROCESS
			GO_MITOCHONDRION_ORGANIZATION	
			GO_MITOCHONDRIAL_MATRIX	
			GO_MITOCHONDRIAL_MEMBRANE_PART	
			GO_PROTON_TRANSPORTING_ATP_SYNTHASE_COMPLEX	
			KEGG_PARKINSONS_DISEASE	
BAZ2A	*HSD17B6,SDR9C7*	12	GO_MACROMOLECULE_DEACYLATION	GO_MACROMOLECULE_DEACYLATION
			GO_CHROMATIN_MODIFICATION	
BC059370	*HSD17B6,SDR9C7*	12	NA	NA

DQ590166	*HSD17B6,SDR9C7*	12	NA	NA
GLS2	*HSD17B6,SDR9C7*	12	GO_REGULATION_OF_INTRACELLULAR_PROTEIN_TRANSPORT<br>GO_REGULATION_OF_PROTEIN_TARGETING<br>GO_MITOCHONDRIAL_MATRIX<br>GO_HYDROLASE_ACTIVITY_ACTING_ON_CARBON_NITROGEN_BUT_NOT_PEPTIDE_BONDS_IN_LINEAR_AMIDES	GO_MITOCHONDRIAL_MATRIX<br>GO_HYDROLASE_ACTIVITY_ACTING_ON_CARBON_NITROGEN_BUT_NOT_PEPTIDE_BONDS_IN_LINEAR_AMIDES
GPR182	*HSD17B6,SDR9C7*	12	NA	NA
HSD17B6	*HSD17B6,SDR9C7*	12	GO_HORMONE_BIOSYNTHETIC_PROCESS<br>GO_ESTRADIOL_17_BETA_DEHYDROGENASE_ACTIVITY<br>GO_ELECTRON_CARRIER_ACTIVITY<br>GO_OXIDOREDUCTASE_ACTIVITY	GO_HORMONE_BIOSYNTHETIC_PROCESS
KIAA1002	*HSD17B6,SDR9C7*	12	NA	NA
LRP1	*HSD17B6,SDR9C7*	12	NA	NA
MIP	*HSD17B6,SDR9C7*	12	GO_PROTEIN_HOMOTETRAMERIZATION<br>GO_PROTEIN_HOMOOLIGOMERIZATION	GO_PROTEIN_HOMOTETRAMERIZATION<br>GO_PROTEIN_HOMOOLIGOMERIZATION
MIR1228	*HSD17B6,SDR9C7*	12	NA	NA
MYO1A	*HSD17B6,SDR9C7*	12	GO_VESICLE_LOCALIZATION<br>GO_MICROVILLUS<br>GO_ACTIN_BASED_CELL_PROJECTION	GO_MICROVILLUS
NAB2	*HSD17B6,SDR9C7*	12	GO_PERIPHERAL_NERVOUS_SYSTEM_DEVELOPMENT<br>GO_EXTRINSIC_COMPONENT_OF_PLASMA_MEMBRANE<br>GO_OXIDOREDUCTASE_ACTIVITY	GO_PERIPHERAL_NERVOUS_SYSTEM_DEVELOPMENT
NACA	*HSD17B6,SDR9C7*	12	GO_REGULATION_OF_DEVELOPMENTAL_GROWTH<br>GO_REGULATION_OF_GROWTH<br>GO_REGULATION_OF_HEART_MORPHOGENESIS<br>GO_GROWTH<br>GO_DEVELOPMENTAL_GROWTH<br>GO_TRANSCRIPTION_COACTIVATOR_ACTIVITY	GO_REGULATION_OF_HEART_MORPHOGENESIS
NDUFA4L2	*HSD17B6,SDR9C7*	12	KEGG_PARKINSONS_DISEASE	KEGG_PARKINSONS_DISEASE
NXPH4	*HSD17B6,SDR9C7*	12	NA	NA
PRIM1	*HSD17B6,SDR9C7*	12	NA	NA
PTGES3	*HSD17B6,SDR9C7*	12	GO_PROTEIN_REFOLDING<br>GO_DNA_POLYMERASE_ACTIVITY	GO_PROTEIN_REFOLDING
R3HDM2	*HSD17B6,SDR9C7*	12	NA	NA
RBMS2	*HSD17B6,SDR9C7*	12	NA	NA
RDH16	*HSD17B6,SDR9C7*	12	GO_ELECTRON_CARRIER_ACTIVITY<br>GO_OXIDOREDUCTASE_ACTIVITY	GO_ELECTRON_CARRIER_ACTIVITY
SDR9C7	*HSD17B6,SDR9C7*	12	GO_OXIDOREDUCTASE_ACTIVITY	GO_OXIDOREDUCTASE_ACTIVITY
SHMT2	*HSD17B6,SDR9C7*	12	GO_PROTEIN_HOMOTETRAMERIZATION<br>GO_ONE_CARBON_METABOLIC_PROCESS<br>GO_PROTEIN_HOMOOLIGOMERIZATION<br>GO_MITOCHONDRIAL_MATRIX	GO_PROTEIN_HOMOTETRAMERIZATION<br>GO_ONE_CARBON_METABOLIC_PROCESS<br>GO_PROTEIN_HOMOOLIGOMERIZATION<br>GO_MITOCHONDRIAL_MATRIX
SNORD59A	*HSD17B6,SDR9C7*	12	NA	NA
SNORD59B	*HSD17B6,SDR9C7*	12	NA	NA
SPRYD4	*HSD17B6,SDR9C7*	12	NA	NA
STAC3	*HSD17B6,SDR9C7*	12	NA	NA
STAT6	*HSD17B6,SDR9C7*	12	GO_EPITHELIUM_DEVELOPMENT<br>GO_CELL_FATE_COMMITMENT	GO_CELL_FATE_COMMITMENT
TAC3	*HSD17B6,SDR9C7*	12	NA	NA
TIMELESS	*HSD17B6,SDR9C7*	12	GO_EPITHELIUM_DEVELOPMENT<br>GO_TUBE_MORPHOGENESIS	GO_TUBE_MORPHOGENESIS
TMEM194A	*HSD17B6,SDR9C7*	12	NA	NA
ZBTB39	*HSD17B6,SDR9C7*	12	NA	NA
LPHN2	*LPHN2*	1	NA	NA
ADI1	*LINC01250*	2	GO_OXIDOREDUCTASE_ACTIVITY	GO_OXIDOREDUCTASE_ACTIVITY
LINC01250	*LINC01250*	2	NA	NA
RNASEH1	*LINC01250*	2	NA	NA
TRAPPC12	*LINC01250*	2	NA	NA
TSSC1	*LINC01250*	2	NA	NA
ALLC	*LINC01250*	2	NA	NA
RPS7	*LINC01250*	2	GO_EPITHELIUM_DEVELOPMENT<br>GO_TUBE_MORPHOGENESIS	GO_TUBE_MORPHOGENESIS
ATP6V1C2	*KCNF1,FLJ33534*	2	NA	NA
AX746649	*KCNF1,FLJ33534*	2	NA	NA
C2orf50	*KCNF1,FLJ33534*	2	NA	NA
E2F6	*KCNF1,FLJ33534*	2	NA	NA
FLJ33534	*KCNF1,FLJ33534*	2	NA	NA
KCNF1	*KCNF1,FLJ33534*	2	GO_PROTEIN_HOMOOLIGOMERIZATION<br>GO_DELAYED_RECTIFIER_POTASSIUM_CHANNEL_ACTIVITY	GO_PROTEIN_HOMOOLIGOMERIZATION
LINC00570	*KCNF1,FLJ33534*	2	NA	NA

LOC101929733	*KCNF1,FLJ33534*	2	NA	NA
NOL10	*KCNF1,FLJ33534*	2	NA	NA
PDIA6	*KCNF1,FLJ33534*	2	NA	NA
PQLC3	*KCNF1,FLJ33534*	2	NA	NA
ROCK2	*KCNF1,FLJ33534*	2	GO_SMALL_GTPASE_MEDIATED_SIGNAL_TRANSDUCTION	GO_SMALL_GTPASE_MEDIATED_SIGNAL_TRANSDUCTION
CYS1	*KCNF1,FLJ33534*	2	NA	NA
PRM2	*KCNF1,FLJ33534*	2	NA	NA
NTS2R	*KCNF1,FLJ33534*	2	NA	NA
GREB1	*KCNF1,FLJ33534*	2	NA	NA
