# Supplementary material for: Genome-wide association study of brain amyloid deposition as measured by Pittsburgh Compound-B (PiB)-PET imaging
Source: Mol Psychiatry. 2018 Oct 25;26(1):309–21. doi: 10.1038/s41380-018-0246-7 (PMC6219464; doi:10.1038/s41380-018-0246-7)
Supplement: Supplementary file 3 — Table S7 [file 41380_2018_246_MOESM3_ESM.pdf]

Table S7. The detailed eQTL and SMR results of LD SNPs R2≥0.8 with the 16 top SNPs including both *APOE* and non-*APOE* loci

#sentinelSNP: top SNPs of each locus from Table 2
#Gene: *cis*-eQTL genes with the test SNP
#Probe_bp: gene location
#testSNP: the test SNP that is in LD with the sentinel SNP
#R2: LD R-square
#testSNP_bp: the test SNP location
#A1: allele1
#A2: allele2
#Freq: frequency of allele1
#p_GWAS: p-value from the meta-analysis of GWAS
#p_eQTL: the *cis*-eQTL p-value between the test SNP and the gene in the corresponding tissue
#p_SMR: the SMR p-value between the test SNP and the gene in the corresponding tissue

sentinelSNP	Tissue	Chr	Gene	Probe_bp	testSNP	R2	testSNP_bp	A1	A2	Freq	p_GWAS	p_eQTL	p_SMR
rs429358	Brain_Amygdala	19	*CTB-171A8.1*	45178765	rs429358	1.0000	45411941	C	T	0.22	9.09E-30	1.96E-02	2.25E-02
rs429358	Brain_Amygdala	19	*PPP1R37*	45622796	rs429358	1.0000	45411941	C	T	0.22	9.09E-30	2.14E-02	2.44E-02
rs429358	Brain_Amygdala	19	*GIPR*	46179242	rs429358	1.0000	45411941	C	T	0.22	9.09E-30	4.89E-02	5.27E-02
rs429358	Brain_Anterior_cingulate_cortex_BA24	19	*ZNF229*	44937225	rs429358	1.0000	45411941	C	T	0.22	9.09E-30	3.82E-02	4.18E-02
rs429358	Brain_Anterior_cingulate_cortex_BA24	19	*ZNF180*	44992215	rs429358	1.0000	45411941	C	T	0.22	9.09E-30	2.55E-02	2.87E-02
rs429358	Brain_Anterior_cingulate_cortex_BA24	19	*BCAM*	45318499	rs429358	1.0000	45411941	C	T	0.22	9.09E-30	4.94E-02	5.32E-02
rs429358	Brain_Anterior_cingulate_cortex_BA24	19	*CTB-129P6.11*	45455282	rs429358	1.0000	45411941	C	T	0.22	9.09E-30	3.43E-02	3.78E-02
rs429358	Brain_Anterior_cingulate_cortex_BA24	19	*CLASRP*	45558256	rs429358	1.0000	45411941	C	T	0.22	9.09E-30	1.55E-02	1.82E-02
rs429358	Brain_Anterior_cingulate_cortex_BA24	19	*SYMPK*	46342608	rs429358	1.0000	45411941	C	T	0.22	9.09E-30	1.33E-02	1.59E-02
rs429358	Brain_Caudate_basal_ganglia	19	*AC084219.3*	44601274	rs429358	1.0000	45411941	C	T	0.22	9.09E-30	4.21E-02	4.58E-02
rs429358	Brain_Caudate_basal_ganglia	19	*ZNF226*	44675880	rs429358	1.0000	45411941	C	T	0.22	9.09E-30	2.62E-02	2.95E-02
rs429358	Brain_Cerebellar_Hemisphere	19	*ZNF227*	44726560	rs429358	1.0000	45411941	C	T	0.22	9.09E-30	3.49E-03	4.82E-03
rs429358	Brain_Cerebellar_Hemisphere	19	*ZNF285B*	44974651	rs429358	1.0000	45411941	C	T	0.22	9.09E-30	2.01E-02	2.31E-02
rs429358	Brain_Cerebellar_Hemisphere	19	*AC006132.1*	46145425	rs429358	1.0000	45411941	C	T	0.22	9.09E-30	3.90E-02	4.26E-02
rs429358	Brain_Cerebellum	19	*AC084219.4*	44613457	rs429358	1.0000	45411941	C	T	0.22	9.09E-30	2.02E-02	2.32E-02
rs429358	Brain_Cerebellum	19	*ZNF225*	44626710	rs429358	1.0000	45411941	C	T	0.22	9.09E-30	4.48E-04	8.47E-04
rs429358	Brain_Cerebellum	19	*ZNF226*	44675880	rs429358	1.0000	45411941	C	T	0.22	9.09E-30	1.57E-02	1.85E-02
rs429358	Brain_Cerebellum	19	*ZNF227*	44726560	rs429358	1.0000	45411941	C	T	0.22	9.09E-30	6.97E-04	1.22E-03
rs429358	Brain_Cerebellum	19	*ZNF235*	44771040	rs429358	1.0000	45411941	C	T	0.22	9.09E-30	2.66E-02	2.99E-02
rs429358	Brain_Cerebellum	19	*CTC-512J12.7*	44794649	rs429358	1.0000	45411941	C	T	0.22	9.09E-30	3.43E-02	3.78E-02
rs429358	Brain_Cerebellum	19	*ZNF112*	44851042	rs429358	1.0000	45411941	C	T	0.22	9.09E-30	7.76E-03	9.76E-03
rs429358	Brain_Cerebellum	19	*TOMM40*	45400386	rs429358	1.0000	45411941	C	T	0.22	9.09E-30	1.91E-02	2.21E-02
rs429358	Brain_Cerebellum	19	*ZNF296*	45577262	rs429358	1.0000	45411941	C	T	0.22	9.09E-30	2.19E-02	2.49E-02
rs429358	Brain_Cortex	19	*ZNF284*	44585031	rs429358	1.0000	45411941	C	T	0.22	9.09E-30	3.88E-02	4.24E-02

rs429358	Brain_Cortex	19	*APOE*	45410830	rs429358	1.0000	45411941	C	T	0.22	9.09E-30	2.15E-02	2.46E-02
rs429358	Brain_Cortex	19	*CLASRP*	45558256	rs429358	1.0000	45411941	C	T	0.22	9.09E-30	4.21E-02	4.58E-02
rs429358	Brain_Cortex	19	*FOSB*	45974845	rs429358	1.0000	45411941	C	T	0.22	9.09E-30	4.17E-02	4.54E-02
rs429358	Brain_Frontal_Cortex_BA9	19	*ZNF235*	44771040	rs429358	1.0000	45411941	C	T	0.22	9.09E-30	1.04E-02	1.26E-02
rs429358	Brain_Frontal_Cortex_BA9	19	*FOSB*	45974845	rs429358	1.0000	45411941	C	T	0.22	9.09E-30	4.05E-02	4.42E-02
rs429358	Brain_Hippocampus	19	*ZNF284*	44585031	rs429358	1.0000	45411941	C	T	0.22	9.09E-30	2.89E-03	4.09E-03
rs429358	Brain_Hippocampus	19	*SNRPD2*	46193179	rs429358	1.0000	45411941	C	T	0.22	9.09E-30	4.26E-02	4.64E-02
rs429358	Brain_Hippocampus	19	*DMPK*	46279437	rs429358	1.0000	45411941	C	T	0.22	9.09E-30	3.61E-02	3.97E-02
rs429358	Brain_Hypothalamus	19	*ZNF224*	44601935	rs429358	1.0000	45411941	C	T	0.22	9.09E-30	3.56E-02	3.92E-02
rs429358	Brain_Hypothalamus	19	*ZNF180*	44992215	rs429358	1.0000	45411941	C	T	0.22	9.09E-30	4.69E-02	5.07E-02
rs429358	Brain_Hypothalamus	19	*OPA3*	46068077	rs429358	1.0000	45411941	C	T	0.22	9.09E-30	1.85E-02	2.14E-02
rs429358	Brain_Nucleus_accumbens_basal_ganglia	19	*ZNF155*	44486530	rs429358	1.0000	45411941	C	T	0.22	9.09E-30	6.44E-03	8.26E-03
rs429358	Brain_Nucleus_accumbens_basal_ganglia	19	*ZNF225*	44626710	rs429358	1.0000	45411941	C	T	0.22	9.09E-30	3.44E-03	4.75E-03
rs429358	Brain_Nucleus_accumbens_basal_ganglia	19	*ZNF296*	45577262	rs429358	1.0000	45411941	C	T	0.22	9.09E-30	5.07E-03	6.68E-03
rs429358	Brain_Nucleus_accumbens_basal_ganglia	19	*ERCC1*	45946338	rs429358	1.0000	45411941	C	T	0.22	9.09E-30	3.64E-02	4.00E-02
rs429358	Brain_Nucleus_accumbens_basal_ganglia	19	*FOSB*	45974845	rs429358	1.0000	45411941	C	T	0.22	9.09E-30	1.19E-02	1.44E-02
rs429358	Brain_Putamen_basal_ganglia	19	*ZNF222*	44533379	rs429358	1.0000	45411941	C	T	0.22	9.09E-30	9.77E-04	1.62E-03
rs429358	Brain_Spinal_cord_cervical_c-1	19	*SYMPK*	46342608	rs429358	1.0000	45411941	C	T	0.22	9.09E-30	2.28E-02	2.59E-02
rs429358	Brain_Spinal_cord_cervical_c-1	19	*MYPOP*	46399570	rs429358	1.0000	45411941	C	T	0.22	9.09E-30	4.86E-02	5.24E-02
rs429358	Brain_Substantia_nigra	19	*ZNF285B*	44974651	rs429358	1.0000	45411941	C	T	0.22	9.09E-30	3.06E-02	3.41E-02
rs429358	Brain_Substantia_nigra	19	*TOMM40*	45400386	rs429358	1.0000	45411941	C	T	0.22	9.09E-30	3.74E-02	4.10E-02
rs429358	Brain_Substantia_nigra	19	*CD3EAP*	45911745	rs429358	1.0000	45411941	C	T	0.22	9.09E-30	3.55E-03	4.89E-03
rs429358	Brain_Substantia_nigra	19	*RTN2*	45994433	rs429358	1.0000	45411941	C	T	0.22	9.09E-30	3.97E-02	4.34E-02
rs429358	Brain_Substantia_nigra	19	*OPA3*	46068077	rs429358	1.0000	45411941	C	T	0.22	9.09E-30	2.95E-02	3.28E-02
rs429358	Brain_Substantia_nigra	19	*AC006132.1*	46145425	rs429358	1.0000	45411941	C	T	0.22	9.09E-30	9.01E-04	1.51E-03
rs429358	Brain_Substantia_nigra	19	*GIPR*	46179242	rs429358	1.0000	45411941	C	T	0.22	9.09E-30	8.16E-03	1.02E-02
rs429358	Whole_Blood	19	*CD3EAP*	45911745	rs429358	1.0000	45411941	C	T	0.22	9.09E-30	2.17E-03	3.19E-03
rs429358	Whole_Blood	19	*GPR4*	46098016	rs429358	1.0000	45411941	C	T	0.22	9.09E-30	6.11E-03	7.89E-03
rs13260032	Brain_Amygdala	8	*ADCY8*	131923609	rs12546678	0.8954	132405080	A	C	0.42	9.70E-06	3.89E-03	1.86E-02
rs13260032	Brain_Amygdala	8	*ADCY8*	131923609	rs28544439	0.8954	132405308	A	C	0.42	3.11E-06	5.09E-03	1.98E-02
rs13260032	Brain_Amygdala	8	*ADCY8*	131923609	rs1900932	0.8954	132405894	A	G	0.43	2.08E-06	4.47E-03	1.79E-02
rs13260032	Brain_Amygdala	8	*ADCY8*	131923609	rs891429	0.8014	132406960	C	T	0.46	2.44E-06	8.99E-03	2.78E-02
rs13260032	Brain_Amygdala	8	*ADCY8*	131923609	rs10956599	0.8954	132408083	T	C	0.42	2.04E-06	5.96E-03	2.10E-02
rs13260032	Brain_Amygdala	8	*ADCY8*	131923609	rs11774542	0.8269	132416893	A	G	0.45	1.75E-04	2.39E-02	9.19E-02
rs13260032	Brain_Amygdala	8	*ADCY8*	131923609	rs10956600	0.8576	132418520	C	A	0.47	9.59E-05	1.51E-02	6.75E-02
rs13260032	Brain_Amygdala	8	*ADCY8*	131923609	rs1430324	0.8497	132419109	T	C	0.47	9.59E-05	1.51E-02	6.75E-02
rs13260032	Brain_Amygdala	8	*ADCY8*	131923609	rs1430325	0.8303	132421240	T	A	0.45	3.99E-04	2.37E-02	1.04E-01
rs13260032	Brain_Amygdala	8	*ADCY8*	131923609	rs1375602	0.8497	132422143	T	C	0.45	1.18E-04	3.19E-02	9.27E-02
rs13260032	Brain_Amygdala	8	*ADCY8*	131923609	rs715985	0.8576	132423889	G	A	0.47	9.61E-05	1.51E-02	6.77E-02
rs13260032	Brain_Amygdala	8	*ADCY8*	131923609	rs6470972	0.8303	132424471	T	C	0.47	2.22E-04	1.05E-02	7.17E-02

rs13260032	Brain_Amygdala	8	*ADCY8*	131923609	rs1449471	0.8303	132424978	A	G	0.47	2.51E-04	1.05E-02	7.23E-02
rs13260032	Brain_Amygdala	8	*ADCY8*	131923609	rs1449472	0.8226	132425379	T	C	0.45	3.13E-04	2.39E-02	9.65E-02
rs13260032	Brain_Amygdala	8	*ADCY8*	131923609	rs9918761	0.8303	132425906	A	T	0.47	2.58E-04	1.05E-02	7.26E-02
rs13260032	Brain_Amygdala	8	*ADCY8*	131923609	rs9297822	0.8621	132428061	G	T	0.47	8.54E-05	1.56E-02	6.77E-02
rs13260032	Brain_Amygdala	8	*ADCY8*	131923609	rs1430323	0.8541	132428314	T	C	0.47	6.09E-05	1.19E-02	5.86E-02
rs13260032	Brain_Amygdala	8	*ADCY8*	131923609	rs1430322	0.8346	132429002	A	G	0.45	2.42E-04	2.39E-02	9.53E-02
rs13260032	Brain_Amygdala	8	*ADCY8*	131923609	rs1346816	0.8626	132430495	C	T	0.47	9.47E-05	1.51E-02	6.95E-02
rs13260032	Brain_Amygdala	8	*ADCY8*	131923609	rs6997462	0.8626	132431123	G	A	0.46	3.64E-04	1.53E-02	9.73E-02
rs13260032	Brain_Amygdala	8	*ADCY8*	131923609	rs1562702	0.8626	132431713	C	T	0.47	1.38E-04	1.51E-02	7.52E-02
rs13260032	Brain_Amygdala	8	*ADCY8*	131923609	rs7013917	0.8546	132434320	C	A	0.46	4.10E-06	1.53E-02	4.01E-02
rs13260032	Brain_Amygdala	8	*ADCY8*	131923609	rs1346815	0.8546	132434697	A	G	0.46	4.15E-06	1.53E-02	4.01E-02
rs13260032	Brain_Amygdala	8	*ADCY8*	131923609	rs1367279	0.8626	132435197	G	T	0.46	5.92E-04	2.16E-02	1.19E-01
rs13260032	Brain_Amygdala	8	*ADCY8*	131923609	rs1430320	0.8546	132437383	C	G	0.47	1.19E-04	1.51E-02	7.24E-02
rs13260032	Brain_Amygdala	8	*ADCY8*	131923609	rs1430319	0.8626	132437577	C	T	0.46	2.78E-04	1.43E-02	8.74E-02
rs13260032	Brain_Amygdala	8	*ADCY8*	131923609	rs1367278	0.8592	132437635	G	A	0.47	1.10E-04	1.51E-02	7.12E-02
rs13260032	Brain_Amygdala	8	*ADCY8*	131923609	rs2195626	0.8626	132437929	A	G	0.47	1.09E-04	1.51E-02	7.09E-02
rs13260032	Brain_Amygdala	8	*ADCY8*	131923609	rs10956601	0.8660	132439003	G	T	0.46	2.77E-04	1.47E-02	8.76E-02
rs13260032	Brain_Amygdala	8	*ADCY8*	131923609	rs6470975	0.8626	132439128	C	T	0.46	3.99E-04	1.47E-02	9.65E-02
rs13260032	Brain_Amygdala	8	*ADCY8*	131923609	rs10429315	0.8660	132439631	G	T	0.47	1.14E-04	1.66E-02	7.42E-02
rs13260032	Brain_Amygdala	8	*ADCY8*	131923609	rs1430317	0.8581	132441048	C	T	0.46	2.81E-04	1.47E-02	8.81E-02
rs13260032	Brain_Amygdala	8	*ADCY8*	131923609	rs1430316	0.8660	132443583	C	T	0.46	4.56E-04	1.53E-02	1.02E-01
rs13260032	Brain_Amygdala	8	*ADCY8*	131923609	rs7016765	0.8581	132446857	A	C	0.47	1.10E-04	9.18E-03	6.11E-02
rs13260032	Brain_Amygdala	8	*ADCY8*	131923609	rs7016957	0.8581	132447031	T	C	0.47	1.20E-04	2.28E-02	8.33E-02
rs13260032	Brain_Amygdala	8	*ADCY8*	131923609	rs1119003	0.8660	132447848	A	T	0.46	2.80E-04	1.45E-02	8.78E-02
rs13260032	Brain_Amygdala	8	*ADCY8*	131923609	rs1119002	0.8660	132447890	G	A	0.46	2.80E-04	1.60E-02	9.01E-02
rs13260032	Brain_Amygdala	8	*ADCY8*	131923609	rs13260032	1.0000	132451455	C	A	0.43	4.87E-07	1.54E-02	3.27E-02
rs13260032	Brain_Amygdala	8	*ADCY8*	131923609	rs1430315	0.8695	132452764	A	G	0.47	1.20E-04	1.66E-02	7.47E-02
rs13260032	Brain_Amygdala	8	*ADCY8*	131923609	rs1449473	0.8615	132454864	A	C	0.46	4.54E-04	1.47E-02	1.00E-01
rs13260032	Brain_Amygdala	8	*ADCY8*	131923609	rs1449474	0.8615	132454897	A	G	0.46	2.74E-04	1.47E-02	8.76E-02
rs13260032	Brain_Amygdala	8	*ADCY8*	131923609	rs1346812	0.8615	132455103	C	G	0.47	1.10E-04	1.66E-02	7.35E-02
rs13260032	Brain_Amygdala	8	*ADCY8*	131923609	rs1346811	0.8615	132455575	T	A	0.46	2.75E-04	1.48E-02	8.79E-02
rs13260032	Brain_Amygdala	8	*ADCY8*	131923609	rs17489	0.8576	132456603	A	G	0.46	2.65E-04	1.47E-02	8.69E-02
rs13260032	Brain_Amygdala	8	*ADCY8*	131923609	rs13266903	0.8576	132457471	C	A	0.47	3.50E-06	1.66E-02	4.13E-02
rs13260032	Brain_Amygdala	8	*ADCY8*	131923609	rs1430314	0.8576	132458270	G	A	0.46	2.45E-04	1.47E-02	8.56E-02
rs13260032	Brain_Amygdala	8	*ADCY8*	131923609	rs1594331	0.8576	132460787	A	G	0.47	3.79E-06	1.66E-02	4.11E-02
rs13260032	Brain_Amygdala	8	*ADCY8*	131923609	rs10109839	0.8541	132463052	G	A	0.47	1.82E-06	1.66E-02	3.81E-02
rs13260032	Brain_Frontal_Cortex_BA9	8	*ADCY8*	131923609	rs28544439	0.8954	132405308	A	C	0.42	3.11E-06	4.00E-02	6.51E-02
rs13260032	Brain_Frontal_Cortex_BA9	8	*ADCY8*	131923609	rs1900932	0.8954	132405894	A	G	0.43	2.08E-06	4.42E-02	6.87E-02
rs13260032	Brain_Frontal_Cortex_BA9	8	*ADCY8*	131923609	rs11774542	0.8269	132416893	A	G	0.45	1.75E-04	4.63E-02	1.17E-01
rs13260032	Brain_Frontal_Cortex_BA9	8	*ADCY8*	131923609	rs10956600	0.8576	132418520	C	A	0.47	9.59E-05	4.34E-02	1.02E-01

rs13260032	Brain_Frontal_Cortex_BA9	8	*ADCY8*	131923609	rs1430324	0.8497	132419109	T	C	0.47	9.59E-05	3.80E-02	9.66E-02
rs13260032	Brain_Frontal_Cortex_BA9	8	*ADCY8*	131923609	rs1430325	0.8303	132421240	T	A	0.45	3.99E-04	4.63E-02	1.29E-01
rs13260032	Brain_Frontal_Cortex_BA9	8	*ADCY8*	131923609	rs1375602	0.8497	132422143	T	C	0.45	1.18E-04	4.02E-02	1.02E-01
rs13260032	Brain_Frontal_Cortex_BA9	8	*ADCY8*	131923609	rs715985	0.8576	132423889	G	A	0.47	9.61E-05	4.34E-02	1.03E-01
rs13260032	Brain_Frontal_Cortex_BA9	8	*ADCY8*	131923609	rs6470972	0.8303	132424471	T	C	0.47	2.22E-04	4.52E-02	1.16E-01
rs13260032	Brain_Frontal_Cortex_BA9	8	*ADCY8*	131923609	rs1449471	0.8303	132424978	A	G	0.47	2.51E-04	4.52E-02	1.17E-01
rs13260032	Brain_Frontal_Cortex_BA9	8	*ADCY8*	131923609	rs1449472	0.8226	132425379	T	C	0.45	3.13E-04	4.14E-02	1.17E-01
rs13260032	Brain_Frontal_Cortex_BA9	8	*ADCY8*	131923609	rs9918761	0.8303	132425906	A	T	0.47	2.58E-04	4.52E-02	1.17E-01
rs13260032	Brain_Frontal_Cortex_BA9	8	*ADCY8*	131923609	rs1430323	0.8541	132428314	T	C	0.47	6.09E-05	1.71E-02	6.66E-02
rs13260032	Brain_Frontal_Cortex_BA9	8	*ADCY8*	131923609	rs7016957	0.8581	132447031	T	C	0.47	1.20E-04	2.67E-02	8.83E-02
rs13260032	Brain_Frontal_Cortex_BA9	8	*ADCY8*	131923609	rs13260032	1.0000	132451455	C	A	0.43	4.87E-07	4.75E-02	6.96E-02
rs13260032	Brain_Hippocampus	8	*EFR3A*	132971112	rs12546678	0.8954	132405080	A	C	0.42	9.70E-06	4.39E-02	7.10E-02
rs13260032	Brain_Hippocampus	8	*EFR3A*	132971112	rs28544439	0.8954	132405308	A	C	0.42	3.11E-06	3.20E-02	5.62E-02
rs13260032	Brain_Hippocampus	8	*EFR3A*	132971112	rs1900932	0.8954	132405894	A	G	0.43	2.08E-06	2.38E-02	4.57E-02
rs13260032	Brain_Hippocampus	8	*EFR3A*	132971112	rs10956599	0.8954	132408083	T	C	0.42	2.04E-06	2.42E-02	4.65E-02
rs13260032	Brain_Nucleus_accumbens_basal_ganglia	8	*CTD-2501M5.1*	132325335	rs12546678	0.8954	132405080	A	C	0.42	9.70E-06	3.80E-02	6.46E-02
rs13260032	Brain_Nucleus_accumbens_basal_ganglia	8	*CTD-2501M5.1*	132325335	rs28544439	0.8954	132405308	A	C	0.42	3.11E-06	1.48E-02	3.50E-02
rs13260032	Brain_Nucleus_accumbens_basal_ganglia	8	*CTD-2501M5.1*	132325335	rs1900932	0.8954	132405894	A	G	0.43	2.08E-06	4.23E-02	6.66E-02
rs13260032	Brain_Nucleus_accumbens_basal_ganglia	8	*CTD-2501M5.1*	132325335	rs891429	0.8014	132406960	C	T	0.46	2.44E-06	2.46E-02	4.89E-02
rs13260032	Brain_Nucleus_accumbens_basal_ganglia	8	*CTD-2501M5.1*	132325335	rs10956599	0.8954	132408083	T	C	0.42	2.04E-06	3.71E-02	6.14E-02
rs13260032	Brain_Nucleus_accumbens_basal_ganglia	8	*CTD-2501M5.1*	132325335	rs11774542	0.8269	132416893	A	G	0.45	1.75E-04	1.19E-02	7.44E-02
rs13260032	Brain_Nucleus_accumbens_basal_ganglia	8	*CTD-2501M5.1*	132325335	rs10956600	0.8576	132418520	C	A	0.47	9.59E-05	3.72E-02	9.57E-02
rs13260032	Brain_Nucleus_accumbens_basal_ganglia	8	*CTD-2501M5.1*	132325335	rs1430324	0.8497	132419109	T	C	0.47	9.59E-05	3.48E-02	9.29E-02
rs13260032	Brain_Nucleus_accumbens_basal_ganglia	8	*CTD-2501M5.1*	132325335	rs1430325	0.8303	132421240	T	A	0.45	3.99E-04	1.20E-02	8.69E-02
rs13260032	Brain_Nucleus_accumbens_basal_ganglia	8	*CTD-2501M5.1*	132325335	rs1375602	0.8497	132422143	T	C	0.45	1.18E-04	9.40E-03	6.09E-02
rs13260032	Brain_Nucleus_accumbens_basal_ganglia	8	*CTD-2501M5.1*	132325335	rs715985	0.8576	132423889	G	A	0.47	9.61E-05	3.72E-02	9.58E-02
rs13260032	Brain_Nucleus_accumbens_basal_ganglia	8	*CTD-2501M5.1*	132325335	rs6470972	0.8303	132424471	T	C	0.47	2.22E-04	4.40E-02	1.15E-01
rs13260032	Brain_Nucleus_accumbens_basal_ganglia	8	*CTD-2501M5.1*	132325335	rs1449471	0.8303	132424978	A	G	0.47	2.51E-04	4.40E-02	1.15E-01
rs13260032	Brain_Nucleus_accumbens_basal_ganglia	8	*CTD-2501M5.1*	132325335	rs1449472	0.8226	132425379	T	C	0.45	3.13E-04	1.15E-02	7.83E-02
rs13260032	Brain_Nucleus_accumbens_basal_ganglia	8	*CTD-2501M5.1*	132325335	rs9918761	0.8303	132425906	A	T	0.47	2.58E-04	4.40E-02	1.16E-01
rs13260032	Brain_Nucleus_accumbens_basal_ganglia	8	*CTD-2501M5.1*	132325335	rs9297822	0.8621	132428061	G	T	0.47	8.54E-05	4.55E-02	1.04E-01
rs13260032	Brain_Nucleus_accumbens_basal_ganglia	8	*CTD-2501M5.1*	132325335	rs1430323	0.8541	132428314	T	C	0.47	6.09E-05	1.95E-02	7.01E-02
rs13260032	Brain_Nucleus_accumbens_basal_ganglia	8	*CTD-2501M5.1*	132325335	rs1430322	0.8346	132429002	A	G	0.45	2.42E-04	1.54E-02	8.35E-02
rs13260032	Brain_Nucleus_accumbens_basal_ganglia	8	*CTD-2501M5.1*	132325335	rs1346816	0.8626	132430495	C	T	0.47	9.47E-05	4.65E-02	1.08E-01
rs13260032	Brain_Nucleus_accumbens_basal_ganglia	8	*CTD-2501M5.1*	132325335	rs6997462	0.8626	132431123	G	A	0.46	3.64E-04	2.81E-02	1.14E-01
rs13260032	Brain_Nucleus_accumbens_basal_ganglia	8	*CTD-2501M5.1*	132325335	rs1562702	0.8626	132431713	C	T	0.47	1.38E-04	4.65E-02	1.13E-01
rs13260032	Brain_Nucleus_accumbens_basal_ganglia	8	*CTD-2501M5.1*	132325335	rs7013917	0.8546	132434320	C	A	0.46	4.10E-06	2.76E-02	5.58E-02
rs13260032	Brain_Nucleus_accumbens_basal_ganglia	8	*CTD-2501M5.1*	132325335	rs1346815	0.8546	132434697	A	G	0.46	4.15E-06	2.76E-02	5.58E-02
rs13260032	Brain_Nucleus_accumbens_basal_ganglia	8	*CTD-2501M5.1*	132325335	rs1367279	0.8626	132435197	G	T	0.46	5.92E-04	3.69E-02	1.37E-01
rs13260032	Brain_Nucleus_accumbens_basal_ganglia	8	*CTD-2501M5.1*	132325335	rs1430320	0.8546	132437383	C	G	0.47	1.19E-04	4.38E-02	1.08E-01

rs13260032	Brain_Nucleus_accumbens_basal_ganglia	8	*CTD-2501M5.1*	132325335	rs1430319	0.8626	132437577	C	T	0.46	2.78E-04	1.77E-02	9.26E-02
rs13260032	Brain_Nucleus_accumbens_basal_ganglia	8	*CTD-2501M5.1*	132325335	rs1367278	0.8592	132437635	G	A	0.47	1.10E-04	4.65E-02	1.09E-01
rs13260032	Brain_Nucleus_accumbens_basal_ganglia	8	*CTD-2501M5.1*	132325335	rs2195626	0.8626	132437929	A	G	0.47	1.09E-04	4.62E-02	1.09E-01
rs13260032	Brain_Nucleus_accumbens_basal_ganglia	8	*CTD-2501M5.1*	132325335	rs10956601	0.8660	132439003	G	T	0.46	2.77E-04	2.06E-02	9.62E-02
rs13260032	Brain_Nucleus_accumbens_basal_ganglia	8	*CTD-2501M5.1*	132325335	rs6470975	0.8626	132439128	C	T	0.46	3.99E-04	2.25E-02	1.08E-01
rs13260032	Brain_Nucleus_accumbens_basal_ganglia	8	*CTD-2501M5.1*	132325335	rs1430317	0.8581	132441048	C	T	0.46	2.81E-04	2.03E-02	9.62E-02
rs13260032	Brain_Nucleus_accumbens_basal_ganglia	8	*CTD-2501M5.1*	132325335	rs1430316	0.8660	132443583	C	T	0.46	4.56E-04	4.79E-02	1.40E-01
rs13260032	Brain_Nucleus_accumbens_basal_ganglia	8	*CTD-2501M5.1*	132325335	rs7016765	0.8581	132446857	A	C	0.47	1.10E-04	2.42E-02	8.39E-02
rs13260032	Brain_Nucleus_accumbens_basal_ganglia	8	*CTD-2501M5.1*	132325335	rs7016957	0.8581	132447031	T	C	0.47	1.20E-04	4.50E-02	1.09E-01
rs13260032	Brain_Nucleus_accumbens_basal_ganglia	8	*CTD-2501M5.1*	132325335	rs1119003	0.8660	132447848	A	T	0.46	2.80E-04	2.63E-02	1.04E-01
rs13260032	Brain_Nucleus_accumbens_basal_ganglia	8	*CTD-2501M5.1*	132325335	rs1119002	0.8660	132447890	G	A	0.46	2.80E-04	1.25E-02	8.45E-02
rs13260032	Brain_Nucleus_accumbens_basal_ganglia	8	*CTD-2501M5.1*	132325335	rs1449473	0.8615	132454864	A	C	0.46	4.54E-04	2.03E-02	1.09E-01
rs13260032	Brain_Nucleus_accumbens_basal_ganglia	8	*CTD-2501M5.1*	132325335	rs1449474	0.8615	132454897	A	G	0.46	2.74E-04	2.03E-02	9.58E-02
rs13260032	Brain_Nucleus_accumbens_basal_ganglia	8	*CTD-2501M5.1*	132325335	rs1346811	0.8615	132455575	T	A	0.46	2.75E-04	2.03E-02	9.60E-02
rs13260032	Brain_Nucleus_accumbens_basal_ganglia	8	*CTD-2501M5.1*	132325335	rs17489	0.8576	132456603	A	G	0.46	2.65E-04	2.03E-02	9.51E-02
rs13260032	Brain_Nucleus_accumbens_basal_ganglia	8	*CTD-2501M5.1*	132325335	rs1430314	0.8576	132458270	G	A	0.46	2.45E-04	2.03E-02	9.39E-02
rs13260032	Brain_Putamen_basal_ganglia	8	*ADCY8*	131923609	rs13260032	1.0000	132451455	C	A	0.43	4.87E-07	2.86E-02	4.88E-02
rs13260032	Brain_Spinal_cord_cervical_c-1	8	*ADCY8*	131923609	rs10956601	0.8660	132439003	G	T	0.46	2.77E-04	4.79E-02	1.27E-01
rs13260032	Brain_Spinal_cord_cervical_c-1	8	*ADCY8*	131923609	rs6470975	0.8626	132439128	C	T	0.46	3.99E-04	3.38E-02	1.21E-01
rs13260032	Brain_Spinal_cord_cervical_c-1	8	*ADCY8*	131923609	rs1430317	0.8581	132441048	C	T	0.46	2.81E-04	4.79E-02	1.28E-01
rs13260032	Brain_Spinal_cord_cervical_c-1	8	*ADCY8*	131923609	rs1430316	0.8660	132443583	C	T	0.46	4.56E-04	4.70E-02	1.39E-01
rs13260032	Brain_Spinal_cord_cervical_c-1	8	*ADCY8*	131923609	rs13260032	1.0000	132451455	C	A	0.43	4.87E-07	1.77E-02	3.57E-02
rs13260032	Brain_Spinal_cord_cervical_c-1	8	*ADCY8*	131923609	rs1449473	0.8615	132454864	A	C	0.46	4.54E-04	4.79E-02	1.40E-01
rs13260032	Brain_Spinal_cord_cervical_c-1	8	*ADCY8*	131923609	rs1449474	0.8615	132454897	A	G	0.46	2.74E-04	4.79E-02	1.27E-01
rs13260032	Brain_Spinal_cord_cervical_c-1	8	*ADCY8*	131923609	rs17489	0.8576	132456603	A	G	0.46	2.65E-04	4.79E-02	1.27E-01
rs13260032	Brain_Spinal_cord_cervical_c-1	8	*ADCY8*	131923609	rs1430314	0.8576	132458270	G	A	0.46	2.45E-04	4.79E-02	1.26E-01
rs4680057	Brain_Anterior_cingulate_cortex_BA24	3	*RP11-38P22.2*	152558203	rs4680056	0.9523	153096430	G	A	0.43	5.13E-05	4.30E-02	6.91E-02
rs4680057	Brain_Anterior_cingulate_cortex_BA24	3	*RP11-38P22.2*	152558203	rs4680057	1.0000	153096985	A	G	0.44	9.69E-07	2.43E-02	4.72E-02
rs4680057	Brain_Anterior_cingulate_cortex_BA24	3	*RP11-38P22.2*	152558203	rs7627262	0.9489	153097522	T	C	0.43	1.84E-05	2.79E-02	6.43E-02
rs4680057	Brain_Anterior_cingulate_cortex_BA24	3	*RP11-38P22.2*	152558203	rs7639345	0.9489	153097961	C	T	0.43	1.54E-05	2.79E-02	6.36E-02
rs4680057	Brain_Anterior_cingulate_cortex_BA24	3	*RP11-38P22.2*	152558203	rs2135773	0.9489	153098130	T	C	0.43	1.79E-05	2.79E-02	6.39E-02
rs4680057	Brain_Anterior_cingulate_cortex_BA24	3	*RP11-38P22.2*	152558203	rs1018179	0.9489	153098264	C	T	0.43	1.66E-05	2.79E-02	6.35E-02
rs4680057	Brain_Anterior_cingulate_cortex_BA24	3	*RP11-38P22.2*	152558203	rs9852688	0.9489	153098558	C	T	0.43	1.66E-05	2.79E-02	6.35E-02
rs4680057	Brain_Anterior_cingulate_cortex_BA24	3	*RP11-38P22.2*	152558203	rs955014	0.9451	153099380	C	T	0.43	1.37E-05	4.10E-02	7.61E-02
rs4680057	Brain_Anterior_cingulate_cortex_BA24	3	*RP11-38P22.2*	152558203	rs13091512	0.9451	153099518	T	A	0.43	1.76E-05	2.79E-02	6.26E-02
rs4680057	Brain_Anterior_cingulate_cortex_BA24	3	*RP11-38P22.2*	152558203	rs9843491	0.9412	153100477	A	G	0.43	2.01E-05	1.29E-02	4.33E-02
rs4680057	Brain_Anterior_cingulate_cortex_BA24	3	*RP11-38P22.2*	152558203	rs4679708	0.9372	153102867	G	C	0.43	4.96E-05	1.29E-02	4.64E-02
rs4680057	Brain_Anterior_cingulate_cortex_BA24	3	*RP11-38P22.2*	152558203	rs4679709	0.9332	153103018	G	A	0.43	5.10E-05	1.29E-02	4.66E-02
rs4680057	Brain_Anterior_cingulate_cortex_BA24	3	*RP11-38P22.2*	152558203	rs1018176	0.9332	153103636	A	G	0.43	4.96E-05	1.29E-02	4.64E-02
rs4680057	Brain_Anterior_cingulate_cortex_BA24	3	*ARHGEF26-AS1*	153790655	rs1018177	0.8535	153103714	G	A	0.48	1.88E-04	2.08E-02	7.08E-02

rs4680057	Brain_Anterior_cingulate_cortex_BA24	3	*RP11-38P22.2*	152558203	rs9864621	0.9294	153104577	T	C	0.43	2.99E-05	1.29E-02	4.48E-02
rs4680057	Brain_Anterior_cingulate_cortex_BA24	3	*RP11-38P22.2*	152558203	rs9815053	0.9332	153105128	A	G	0.43	4.62E-05	1.29E-02	4.60E-02
rs4680057	Brain_Anterior_cingulate_cortex_BA24	3	*RP11-38P22.2*	152558203	rs9815215	0.9332	153105380	T	C	0.43	2.97E-05	1.29E-02	4.37E-02
rs4680057	Brain_Anterior_cingulate_cortex_BA24	3	*RP11-38P22.2*	152558203	rs9835730	0.9332	153105773	C	T	0.43	4.71E-05	1.29E-02	4.61E-02
rs4680057	Brain_Anterior_cingulate_cortex_BA24	3	*RP11-38P22.2*	152558203	rs9819928	0.9332	153105912	A	G	0.43	4.07E-05	1.29E-02	4.57E-02
rs4680057	Brain_Anterior_cingulate_cortex_BA24	3	*RP11-38P22.2*	152558203	rs9858222	0.9332	153106464	G	A	0.43	4.24E-05	1.29E-02	4.58E-02
rs4680057	Brain_Anterior_cingulate_cortex_BA24	3	*RP11-38P22.2*	152558203	rs6777504	0.9294	153106837	T	C	0.43	3.60E-05	1.29E-02	4.53E-02
rs4680057	Brain_Anterior_cingulate_cortex_BA24	3	*ARHGEF26-AS1*	153790655	rs9831094	0.8535	153108239	A	G	0.48	1.61E-04	2.67E-02	7.72E-02
rs4680057	Brain_Anterior_cingulate_cortex_BA24	3	*RP11-38P22.2*	152558203	rs9831443	0.9332	153108551	G	C	0.43	3.66E-05	1.29E-02	4.54E-02
rs4680057	Brain_Anterior_cingulate_cortex_BA24	3	*P2RY1*	152554288	rs1827993	0.9294	153110407	T	G	0.43	7.17E-05	4.99E-02	9.15E-02
rs4680057	Brain_Anterior_cingulate_cortex_BA24	3	*RP11-38P22.2*	152558203	rs1827993	0.9294	153110407	T	G	0.43	7.17E-05	4.97E-03	3.21E-02
rs4680057	Brain_Anterior_cingulate_cortex_BA24	3	*RP11-38P22.2*	152558203	rs9862101	0.9332	153110559	A	T	0.43	3.66E-05	1.25E-02	4.48E-02
rs4680057	Brain_Anterior_cingulate_cortex_BA24	3	*RP11-38P22.2*	152558203	rs2135774	0.9332	153113202	T	C	0.43	4.33E-05	1.25E-02	4.52E-02
rs4680057	Brain_Anterior_cingulate_cortex_BA24	3	*ARHGEF26-AS1*	153790655	rs1580988	0.8573	153114027	C	A	0.48	2.19E-04	4.94E-02	1.04E-01
rs4680057	Brain_Anterior_cingulate_cortex_BA24	3	*RP11-38P22.2*	152558203	rs6790236	0.9331	153115590	G	C	0.43	4.20E-05	1.29E-02	4.48E-02
rs4680057	Brain_Anterior_cingulate_cortex_BA24	3	*RP11-38P22.2*	152558203	rs6790408	0.9331	153115593	A	G	0.43	4.20E-05	1.29E-02	4.48E-02
rs4680057	Brain_Anterior_cingulate_cortex_BA24	3	*ARHGEF26-AS1*	153790655	rs3903613	0.8498	153116369	C	T	0.48	1.75E-04	9.57E-03	5.23E-02
rs4680057	Brain_Anterior_cingulate_cortex_BA24	3	*P2RY1*	152554288	rs9289909	0.9255	153116825	C	G	0.43	7.61E-05	4.99E-02	9.15E-02
rs4680057	Brain_Anterior_cingulate_cortex_BA24	3	*RP11-38P22.2*	152558203	rs9289909	0.9255	153116825	C	G	0.43	7.61E-05	4.97E-03	3.21E-02
rs4680057	Brain_Anterior_cingulate_cortex_BA24	3	*ARHGEF26-AS1*	153790655	rs6794116	0.8498	153116888	A	G	0.48	1.83E-04	2.17E-02	7.12E-02
rs4680057	Brain_Anterior_cingulate_cortex_BA24	3	*RP11-38P22.2*	152558203	rs9850272	0.9293	153116954	A	C	0.43	4.46E-05	1.14E-02	4.35E-02
rs4680057	Brain_Anterior_cingulate_cortex_BA24	3	*RP11-38P22.2*	152558203	rs9289910	0.9293	153117193	G	A	0.43	4.04E-05	1.29E-02	4.52E-02
rs4680057	Brain_Anterior_cingulate_cortex_BA24	3	*ARHGEF26-AS1*	153790655	rs1507160	0.8462	153117704	G	A	0.47	2.69E-04	2.08E-02	7.54E-02
rs4680057	Brain_Anterior_cingulate_cortex_BA24	3	*ARHGEF26-AS1*	153790655	rs10049279	0.8498	153118601	A	G	0.48	1.77E-04	2.08E-02	6.92E-02
rs4680057	Brain_Anterior_cingulate_cortex_BA24	3	*RP11-38P22.2*	152558203	rs4680059	0.8498	153118849	A	G	0.48	2.20E-04	4.90E-02	1.03E-01
rs4680057	Brain_Anterior_cingulate_cortex_BA24	3	*ARHGEF26-AS1*	153790655	rs4680059	0.8498	153118849	A	G	0.48	2.20E-04	1.04E-02	5.43E-02
rs4680057	Brain_Anterior_cingulate_cortex_BA24	3	*RP11-38P22.2*	152558203	rs12695988	0.9293	153122300	A	C	0.43	2.73E-05	1.12E-02	4.00E-02
rs4680057	Brain_Caudate_basal_ganglia	3	*DDX50P2*	154071256	rs1018177	0.8535	153103714	G	A	0.48	1.88E-04	2.79E-02	7.99E-02
rs4680057	Brain_Caudate_basal_ganglia	3	*ARHGEF26-AS1*	153790655	rs9831094	0.8535	153108239	A	G	0.48	1.61E-04	2.23E-02	7.17E-02
rs4680057	Brain_Caudate_basal_ganglia	3	*DDX50P2*	154071256	rs9831094	0.8535	153108239	A	G	0.48	1.61E-04	1.27E-02	5.77E-02
rs4680057	Brain_Caudate_basal_ganglia	3	*DDX50P2*	154071256	rs1580988	0.8573	153114027	C	A	0.48	2.19E-04	2.73E-02	7.95E-02
rs4680057	Brain_Caudate_basal_ganglia	3	*DDX50P2*	154071256	rs3903613	0.8498	153116369	C	T	0.48	1.75E-04	2.88E-02	7.98E-02
rs4680057	Brain_Caudate_basal_ganglia	3	*ARHGEF26-AS1*	153790655	rs6794116	0.8498	153116888	A	G	0.48	1.83E-04	4.64E-02	1.00E-01
rs4680057	Brain_Caudate_basal_ganglia	3	*DDX50P2*	154071256	rs6794116	0.8498	153116888	A	G	0.48	1.83E-04	2.97E-02	8.13E-02
rs4680057	Brain_Caudate_basal_ganglia	3	*ARHGEF26-AS1*	153790655	rs1507160	0.8462	153117704	G	A	0.47	2.69E-04	4.58E-02	1.05E-01
rs4680057	Brain_Caudate_basal_ganglia	3	*DDX50P2*	154071256	rs1507160	0.8462	153117704	G	A	0.47	2.69E-04	3.17E-02	8.90E-02
rs4680057	Brain_Caudate_basal_ganglia	3	*ARHGEF26-AS1*	153790655	rs10049279	0.8498	153118601	A	G	0.48	1.77E-04	4.51E-02	9.77E-02
rs4680057	Brain_Caudate_basal_ganglia	3	*DDX50P2*	154071256	rs10049279	0.8498	153118601	A	G	0.48	1.77E-04	3.25E-02	8.37E-02
rs4680057	Brain_Caudate_basal_ganglia	3	*DDX50P2*	154071256	rs4680059	0.8498	153118849	A	G	0.48	2.20E-04	1.69E-02	6.47E-02
rs4680057	Brain_Cerebellar_Hemisphere	3	*RAP2B*	152883147	rs4680056	0.9523	153096430	G	A	0.43	5.13E-05	2.94E-02	5.39E-02

rs4680057	Brain_Cerebellum	3	*RP11-362A9.3*	152195075	rs13073679	0.9523	153096642	T	C	0.44	1.03E-03	4.55E-02	1.10E-01
rs4680057	Brain_Cerebellum	3	*RP11-362A9.3*	152195075	rs9289909	0.9255	153116825	C	G	0.43	7.61E-05	4.79E-02	8.94E-02
rs4680057	Brain_Frontal_Cortex_BA9	3	*ARHGEF26-AS1*	153790655	rs4680059	0.8498	153118849	A	G	0.48	2.20E-04	4.49E-02	9.85E-02
rs4680057	Brain_Hippocampus	3	*RP11-38P22.2*	152558203	rs13073679	0.9523	153096642	T	C	0.44	1.03E-03	1.56E-02	7.39E-02
rs4680057	Brain_Hippocampus	3	*RP11-38P22.2*	152558203	rs9822507	0.9293	153096674	C	G	0.48	9.79E-02	1.88E-02	4.92E-02
rs4680057	Brain_Hippocampus	3	*RP11-38P22.2*	152558203	rs4680057	1.0000	153096985	A	G	0.44	9.69E-07	1.58E-02	3.64E-02
rs4680057	Brain_Hippocampus	3	*RP11-38P22.2*	152558203	rs7627262	0.9489	153097522	T	C	0.43	1.84E-05	1.88E-02	5.29E-02
rs4680057	Brain_Hippocampus	3	*RP11-38P22.2*	152558203	rs7639345	0.9489	153097961	C	T	0.43	1.54E-05	1.88E-02	5.21E-02
rs4680057	Brain_Hippocampus	3	*RP11-38P22.2*	152558203	rs2135773	0.9489	153098130	T	C	0.43	1.79E-05	1.88E-02	5.24E-02
rs4680057	Brain_Hippocampus	3	*RP11-38P22.2*	152558203	rs1018179	0.9489	153098264	C	T	0.43	1.66E-05	1.88E-02	5.20E-02
rs4680057	Brain_Hippocampus	3	*RP11-38P22.2*	152558203	rs9852688	0.9489	153098558	C	T	0.43	1.66E-05	1.88E-02	5.20E-02
rs4680057	Brain_Hippocampus	3	*RP11-38P22.2*	152558203	rs955014	0.9451	153099380	C	T	0.43	1.37E-05	2.32E-03	2.05E-02
rs4680057	Brain_Hippocampus	3	*RP11-38P22.2*	152558203	rs13091512	0.9451	153099518	T	A	0.43	1.76E-05	4.58E-03	2.76E-02
rs4680057	Brain_Hippocampus	3	*RP11-38P22.2*	152558203	rs9843491	0.9412	153100477	A	G	0.43	2.01E-05	1.38E-02	4.47E-02
rs4680057	Brain_Hippocampus	3	*RP11-38P22.2*	152558203	rs4679708	0.9372	153102867	G	C	0.43	4.96E-05	1.14E-02	4.40E-02
rs4680057	Brain_Hippocampus	3	*RP11-38P22.2*	152558203	rs4679709	0.9332	153103018	G	A	0.43	5.10E-05	1.14E-02	4.42E-02
rs4680057	Brain_Hippocampus	3	*RP11-38P22.2*	152558203	rs1018176	0.9332	153103636	A	G	0.43	4.96E-05	1.14E-02	4.40E-02
rs4680057	Brain_Hippocampus	3	*RP11-38P22.2*	152558203	rs1018177	0.8535	153103714	G	A	0.48	1.88E-04	3.70E-02	9.06E-02
rs4680057	Brain_Hippocampus	3	*RP11-38P22.2*	152558203	rs9864621	0.9294	153104577	T	C	0.43	2.99E-05	1.14E-02	4.25E-02
rs4680057	Brain_Hippocampus	3	*RP11-38P22.2*	152558203	rs9815053	0.9332	153105128	A	G	0.43	4.62E-05	1.14E-02	4.36E-02
rs4680057	Brain_Hippocampus	3	*RP11-38P22.2*	152558203	rs9815215	0.9332	153105380	T	C	0.43	2.97E-05	1.14E-02	4.14E-02
rs4680057	Brain_Hippocampus	3	*RP11-38P22.2*	152558203	rs9835730	0.9332	153105773	C	T	0.43	4.71E-05	1.14E-02	4.37E-02
rs4680057	Brain_Hippocampus	3	*RP11-38P22.2*	152558203	rs9819928	0.9332	153105912	A	G	0.43	4.07E-05	1.14E-02	4.33E-02
rs4680057	Brain_Hippocampus	3	*RP11-38P22.2*	152558203	rs9858222	0.9332	153106464	G	A	0.43	4.24E-05	1.14E-02	4.34E-02
rs4680057	Brain_Hippocampus	3	*RP11-38P22.2*	152558203	rs6777504	0.9294	153106837	T	C	0.43	3.60E-05	1.14E-02	4.29E-02
rs4680057	Brain_Hippocampus	3	*RP11-38P22.2*	152558203	rs9831094	0.8535	153108239	A	G	0.48	1.61E-04	2.18E-02	7.10E-02
rs4680057	Brain_Hippocampus	3	*RP11-38P22.2*	152558203	rs9831443	0.9332	153108551	G	C	0.43	3.66E-05	1.14E-02	4.30E-02
rs4680057	Brain_Hippocampus	3	*RP11-38P22.2*	152558203	rs1827993	0.9294	153110407	T	G	0.43	7.17E-05	1.19E-02	4.51E-02
rs4680057	Brain_Hippocampus	3	*RP11-38P22.2*	152558203	rs9862101	0.9332	153110559	A	T	0.43	3.66E-05	1.15E-02	4.33E-02
rs4680057	Brain_Hippocampus	3	*RP11-38P22.2*	152558203	rs2135774	0.9332	153113202	T	C	0.43	4.33E-05	1.09E-02	4.26E-02
rs4680057	Brain_Hippocampus	3	*RP11-38P22.2*	152558203	rs1580988	0.8573	153114027	C	A	0.48	2.19E-04	3.72E-02	9.11E-02
rs4680057	Brain_Hippocampus	3	*RP11-38P22.2*	152558203	rs6790236	0.9331	153115590	G	C	0.43	4.20E-05	1.14E-02	4.24E-02
rs4680057	Brain_Hippocampus	3	*RP11-38P22.2*	152558203	rs6790408	0.9331	153115593	A	G	0.43	4.20E-05	1.14E-02	4.24E-02
rs4680057	Brain_Hippocampus	3	*RP11-38P22.2*	152558203	rs3903613	0.8498	153116369	C	T	0.48	1.75E-04	3.94E-02	9.20E-02
rs4680057	Brain_Hippocampus	3	*ARHGEF26-AS1*	153790655	rs3903613	0.8498	153116369	C	T	0.48	1.75E-04	4.40E-02	9.70E-02
rs4680057	Brain_Hippocampus	3	*RP11-38P22.2*	152558203	rs9289909	0.9255	153116825	C	G	0.43	7.61E-05	1.19E-02	4.51E-02
rs4680057	Brain_Hippocampus	3	*RP11-38P22.2*	152558203	rs6794116	0.8498	153116888	A	G	0.48	1.83E-04	3.70E-02	8.98E-02
rs4680057	Brain_Hippocampus	3	*RP11-38P22.2*	152558203	rs9850272	0.9293	153116954	A	C	0.43	4.46E-05	9.79E-03	4.08E-02
rs4680057	Brain_Hippocampus	3	*RP11-38P22.2*	152558203	rs9289910	0.9293	153117193	G	A	0.43	4.04E-05	1.14E-02	4.28E-02
rs4680057	Brain_Hippocampus	3	*RP11-38P22.2*	152558203	rs1507160	0.8462	153117704	G	A	0.47	2.69E-04	3.70E-02	9.51E-02

rs4680057	Brain_Hippocampus	3	*RP11-38P22.2*	152558203	rs10049279	0.8498	153118601	A	G	0.48	1.77E-04	3.70E-02	8.90E-02
rs4680057	Brain_Hippocampus	3	*RP11-38P22.2*	152558203	rs4680059	0.8498	153118849	A	G	0.48	2.20E-04	1.53E-02	6.22E-02
rs4680057	Brain_Hippocampus	3	*RP11-38P22.2*	152558203	rs12695988	0.9293	153122300	A	C	0.43	2.73E-05	9.64E-03	3.75E-02
rs4680057	Brain_Hypothalamus	3	*DHX36*	154016310	rs1018177	0.8535	153103714	G	A	0.48	1.88E-04	3.54E-02	8.88E-02
rs4680057	Brain_Hypothalamus	3	*DHX36*	154016310	rs9831094	0.8535	153108239	A	G	0.48	1.61E-04	3.61E-02	8.83E-02
rs4680057	Brain_Hypothalamus	3	*ARHGEF26*	153907204	rs3903613	0.8498	153116369	C	T	0.48	1.75E-04	2.25E-02	7.18E-02
rs4680057	Brain_Hypothalamus	3	*DHX36*	154016310	rs6794116	0.8498	153116888	A	G	0.48	1.83E-04	3.72E-02	8.99E-02
rs4680057	Brain_Hypothalamus	3	*DHX36*	154016310	rs1507160	0.8462	153117704	G	A	0.47	2.69E-04	3.61E-02	9.41E-02
rs4680057	Brain_Hypothalamus	3	*DHX36*	154016310	rs10049279	0.8498	153118601	A	G	0.48	1.77E-04	3.61E-02	8.79E-02
rs4680057	Brain_Nucleus_accumbens_basal_ganglia	3	*RAP2B*	152883147	rs4680056	0.9523	153096430	G	A	0.43	5.13E-05	7.60E-03	2.49E-02
rs4680057	Brain_Nucleus_accumbens_basal_ganglia	3	*RAP2B*	152883147	rs13073679	0.9523	153096642	T	C	0.44	1.03E-03	1.99E-02	8.00E-02
rs4680057	Brain_Nucleus_accumbens_basal_ganglia	3	*RAP2B*	152883147	rs9822507	0.9293	153096674	C	G	0.48	9.79E-02	1.18E-02	3.91E-02
rs4680057	Brain_Nucleus_accumbens_basal_ganglia	3	*RAP2B*	152883147	rs4680057	1.0000	153096985	A	G	0.44	9.69E-07	2.00E-02	4.19E-02
rs4680057	Brain_Nucleus_accumbens_basal_ganglia	3	*RAP2B*	152883147	rs7627262	0.9489	153097522	T	C	0.43	1.84E-05	1.21E-02	4.31E-02
rs4680057	Brain_Nucleus_accumbens_basal_ganglia	3	*RAP2B*	152883147	rs7639345	0.9489	153097961	C	T	0.43	1.54E-05	1.21E-02	4.24E-02
rs4680057	Brain_Nucleus_accumbens_basal_ganglia	3	*RAP2B*	152883147	rs2135773	0.9489	153098130	T	C	0.43	1.79E-05	1.21E-02	4.27E-02
rs4680057	Brain_Nucleus_accumbens_basal_ganglia	3	*RAP2B*	152883147	rs1018179	0.9489	153098264	C	T	0.43	1.66E-05	1.21E-02	4.23E-02
rs4680057	Brain_Nucleus_accumbens_basal_ganglia	3	*RAP2B*	152883147	rs9852688	0.9489	153098558	C	T	0.43	1.66E-05	1.21E-02	4.23E-02
rs4680057	Brain_Nucleus_accumbens_basal_ganglia	3	*RAP2B*	152883147	rs955014	0.9451	153099380	C	T	0.43	1.37E-05	4.12E-03	2.53E-02
rs4680057	Brain_Nucleus_accumbens_basal_ganglia	3	*RAP2B*	152883147	rs13091512	0.9451	153099518	T	A	0.43	1.76E-05	5.64E-03	3.00E-02
rs4680057	Brain_Nucleus_accumbens_basal_ganglia	3	*RAP2B*	152883147	rs9843491	0.9412	153100477	A	G	0.43	2.01E-05	3.07E-02	6.68E-02
rs4680057	Brain_Nucleus_accumbens_basal_ganglia	3	*RAP2B*	152883147	rs4679708	0.9372	153102867	G	C	0.43	4.96E-05	3.22E-02	7.18E-02
rs4680057	Brain_Nucleus_accumbens_basal_ganglia	3	*RAP2B*	152883147	rs4679709	0.9332	153103018	G	A	0.43	5.10E-05	2.91E-02	6.83E-02
rs4680057	Brain_Nucleus_accumbens_basal_ganglia	3	*RAP2B*	152883147	rs1018176	0.9332	153103636	A	G	0.43	4.96E-05	2.91E-02	6.81E-02
rs4680057	Brain_Nucleus_accumbens_basal_ganglia	3	*RAP2B*	152883147	rs1018177	0.8535	153103714	G	A	0.48	1.88E-04	9.26E-03	5.29E-02
rs4680057	Brain_Nucleus_accumbens_basal_ganglia	3	*RAP2B*	152883147	rs9864621	0.9294	153104577	T	C	0.43	2.99E-05	2.91E-02	6.64E-02
rs4680057	Brain_Nucleus_accumbens_basal_ganglia	3	*RAP2B*	152883147	rs9815053	0.9332	153105128	A	G	0.43	4.62E-05	2.91E-02	6.76E-02
rs4680057	Brain_Nucleus_accumbens_basal_ganglia	3	*RAP2B*	152883147	rs9815215	0.9332	153105380	T	C	0.43	2.97E-05	2.91E-02	6.52E-02
rs4680057	Brain_Nucleus_accumbens_basal_ganglia	3	*RAP2B*	152883147	rs9835730	0.9332	153105773	C	T	0.43	4.71E-05	2.91E-02	6.77E-02
rs4680057	Brain_Nucleus_accumbens_basal_ganglia	3	*RAP2B*	152883147	rs9819928	0.9332	153105912	A	G	0.43	4.07E-05	2.91E-02	6.73E-02
rs4680057	Brain_Nucleus_accumbens_basal_ganglia	3	*RAP2B*	152883147	rs9858222	0.9332	153106464	G	A	0.43	4.24E-05	2.91E-02	6.74E-02
rs4680057	Brain_Nucleus_accumbens_basal_ganglia	3	*RAP2B*	152883147	rs6777504	0.9294	153106837	T	C	0.43	3.60E-05	2.91E-02	6.69E-02
rs4680057	Brain_Nucleus_accumbens_basal_ganglia	3	*RAP2B*	152883147	rs9831094	0.8535	153108239	A	G	0.48	1.61E-04	1.02E-02	5.34E-02
rs4680057	Brain_Nucleus_accumbens_basal_ganglia	3	*RAP2B*	152883147	rs9831443	0.9332	153108551	G	C	0.43	3.66E-05	2.91E-02	6.70E-02
rs4680057	Brain_Nucleus_accumbens_basal_ganglia	3	*RP11-38P22.2*	152558203	rs1827993	0.9294	153110407	T	G	0.43	7.17E-05	4.85E-02	9.00E-02
rs4680057	Brain_Nucleus_accumbens_basal_ganglia	3	*RAP2B*	152883147	rs1827993	0.9294	153110407	T	G	0.43	7.17E-05	3.42E-02	7.43E-02
rs4680057	Brain_Nucleus_accumbens_basal_ganglia	3	*RAP2B*	152883147	rs9862101	0.9332	153110559	A	T	0.43	3.66E-05	2.91E-02	6.70E-02
rs4680057	Brain_Nucleus_accumbens_basal_ganglia	3	*RAP2B*	152883147	rs2135774	0.9332	153113202	T	C	0.43	4.33E-05	3.80E-02	7.77E-02
rs4680057	Brain_Nucleus_accumbens_basal_ganglia	3	*RAP2B*	152883147	rs1580988	0.8573	153114027	C	A	0.48	2.19E-04	3.43E-03	3.99E-02
rs4680057	Brain_Nucleus_accumbens_basal_ganglia	3	*RAP2B*	152883147	rs6790236	0.9331	153115590	G	C	0.43	4.20E-05	2.55E-02	6.20E-02

rs4680057	Brain_Nucleus_accumbens_basal_ganglia	3	*RAP2B*	152883147	rs6790408	0.9331	153115593	A	G	0.43	4.20E-05	2.55E-02	6.20E-02
rs4680057	Brain_Nucleus_accumbens_basal_ganglia	3	*RAP2B*	152883147	rs3903613	0.8498	153116369	C	T	0.48	1.75E-04	5.85E-03	4.47E-02
rs4680057	Brain_Nucleus_accumbens_basal_ganglia	3	*RP11-38P22.2*	152558203	rs9289909	0.9255	153116825	C	G	0.43	7.61E-05	4.85E-02	9.00E-02
rs4680057	Brain_Nucleus_accumbens_basal_ganglia	3	*RAP2B*	152883147	rs9289909	0.9255	153116825	C	G	0.43	7.61E-05	3.42E-02	7.43E-02
rs4680057	Brain_Nucleus_accumbens_basal_ganglia	3	*RAP2B*	152883147	rs6794116	0.8498	153116888	A	G	0.48	1.83E-04	9.49E-03	5.26E-02
rs4680057	Brain_Nucleus_accumbens_basal_ganglia	3	*RAP2B*	152883147	rs9850272	0.9293	153116954	A	C	0.43	4.46E-05	2.04E-02	5.66E-02
rs4680057	Brain_Nucleus_accumbens_basal_ganglia	3	*RAP2B*	152883147	rs9289910	0.9293	153117193	G	A	0.43	4.04E-05	2.55E-02	6.24E-02
rs4680057	Brain_Nucleus_accumbens_basal_ganglia	3	*RAP2B*	152883147	rs1507160	0.8462	153117704	G	A	0.47	2.69E-04	8.07E-03	5.50E-02
rs4680057	Brain_Nucleus_accumbens_basal_ganglia	3	*RAP2B*	152883147	rs10049279	0.8498	153118601	A	G	0.48	1.77E-04	8.80E-03	5.05E-02
rs4680057	Brain_Nucleus_accumbens_basal_ganglia	3	*RAP2B*	152883147	rs4680059	0.8498	153118849	A	G	0.48	2.20E-04	3.72E-03	3.98E-02
rs4680057	Brain_Nucleus_accumbens_basal_ganglia	3	*RAP2B*	152883147	rs12695988	0.9293	153122300	A	C	0.43	2.73E-05	3.65E-02	7.27E-02
rs4680057	Brain_Spinal_cord_cervical_c-1	3	*RP11-23D24.2*	153400349	rs4680056	0.9523	153096430	G	A	0.43	5.13E-05	2.15E-02	4.44E-02
rs4680057	Brain_Spinal_cord_cervical_c-1	3	*RP11-23D24.2*	153400349	rs13073679	0.9523	153096642	T	C	0.44	1.03E-03	1.47E-02	7.24E-02
rs4680057	Brain_Spinal_cord_cervical_c-1	3	*RP11-23D24.2*	153400349	rs9822507	0.9293	153096674	C	G	0.48	9.79E-02	1.11E-02	3.79E-02
rs4680057	Brain_Spinal_cord_cervical_c-1	3	*RP11-23D24.2*	153400349	rs4680057	1.0000	153096985	A	G	0.44	9.69E-07	1.51E-02	3.54E-02
rs4680057	Brain_Spinal_cord_cervical_c-1	3	*RP11-23D24.2*	153400349	rs7627262	0.9489	153097522	T	C	0.43	1.84E-05	1.11E-02	4.15E-02
rs4680057	Brain_Spinal_cord_cervical_c-1	3	*RP11-23D24.2*	153400349	rs7639345	0.9489	153097961	C	T	0.43	1.54E-05	1.11E-02	4.08E-02
rs4680057	Brain_Spinal_cord_cervical_c-1	3	*RP11-23D24.2*	153400349	rs2135773	0.9489	153098130	T	C	0.43	1.79E-05	1.11E-02	4.11E-02
rs4680057	Brain_Spinal_cord_cervical_c-1	3	*RP11-23D24.2*	153400349	rs1018179	0.9489	153098264	C	T	0.43	1.66E-05	1.11E-02	4.07E-02
rs4680057	Brain_Spinal_cord_cervical_c-1	3	*RP11-23D24.2*	153400349	rs9852688	0.9489	153098558	C	T	0.43	1.66E-05	1.11E-02	4.07E-02
rs4680057	Brain_Spinal_cord_cervical_c-1	3	*RP11-23D24.2*	153400349	rs955014	0.9451	153099380	C	T	0.43	1.37E-05	2.60E-02	5.88E-02
rs4680057	Brain_Spinal_cord_cervical_c-1	3	*RP11-23D24.2*	153400349	rs13091512	0.9451	153099518	T	A	0.43	1.76E-05	2.72E-02	6.19E-02
rs4680057	Brain_Spinal_cord_cervical_c-1	3	*RP11-23D24.2*	153400349	rs9843491	0.9412	153100477	A	G	0.43	2.01E-05	1.91E-02	5.23E-02
rs4680057	Brain_Spinal_cord_cervical_c-1	3	*RP11-23D24.2*	153400349	rs4679708	0.9372	153102867	G	C	0.43	4.96E-05	1.54E-02	5.02E-02
rs4680057	Brain_Spinal_cord_cervical_c-1	3	*RP11-23D24.2*	153400349	rs4679709	0.9332	153103018	G	A	0.43	5.10E-05	1.54E-02	5.04E-02
rs4680057	Brain_Spinal_cord_cervical_c-1	3	*RP11-23D24.2*	153400349	rs1018176	0.9332	153103636	A	G	0.43	4.96E-05	1.54E-02	5.02E-02
rs4680057	Brain_Spinal_cord_cervical_c-1	3	*RP11-23D24.2*	153400349	rs1018177	0.8535	153103714	G	A	0.48	1.88E-04	7.84E-03	5.02E-02
rs4680057	Brain_Spinal_cord_cervical_c-1	3	*RP11-23D24.2*	153400349	rs9864621	0.9294	153104577	T	C	0.43	2.99E-05	1.54E-02	4.86E-02
rs4680057	Brain_Spinal_cord_cervical_c-1	3	*RP11-23D24.2*	153400349	rs9815053	0.9332	153105128	A	G	0.43	4.62E-05	1.54E-02	4.98E-02
rs4680057	Brain_Spinal_cord_cervical_c-1	3	*RP11-23D24.2*	153400349	rs9815215	0.9332	153105380	T	C	0.43	2.97E-05	1.54E-02	4.75E-02
rs4680057	Brain_Spinal_cord_cervical_c-1	3	*RP11-23D24.2*	153400349	rs9835730	0.9332	153105773	C	T	0.43	4.71E-05	1.54E-02	4.99E-02
rs4680057	Brain_Spinal_cord_cervical_c-1	3	*RP11-23D24.2*	153400349	rs9819928	0.9332	153105912	A	G	0.43	4.07E-05	1.54E-02	4.95E-02
rs4680057	Brain_Spinal_cord_cervical_c-1	3	*RP11-23D24.2*	153400349	rs9858222	0.9332	153106464	G	A	0.43	4.24E-05	1.54E-02	4.95E-02
rs4680057	Brain_Spinal_cord_cervical_c-1	3	*RP11-23D24.2*	153400349	rs6777504	0.9294	153106837	T	C	0.43	3.60E-05	1.54E-02	4.91E-02
rs4680057	Brain_Spinal_cord_cervical_c-1	3	*RP11-23D24.2*	153400349	rs9831094	0.8535	153108239	A	G	0.48	1.61E-04	7.84E-03	4.90E-02
rs4680057	Brain_Spinal_cord_cervical_c-1	3	*RP11-23D24.2*	153400349	rs9831443	0.9332	153108551	G	C	0.43	3.66E-05	1.54E-02	4.92E-02
rs4680057	Brain_Spinal_cord_cervical_c-1	3	*RP11-23D24.2*	153400349	rs1827993	0.9294	153110407	T	G	0.43	7.17E-05	3.27E-02	7.25E-02
rs4680057	Brain_Spinal_cord_cervical_c-1	3	*RP11-23D24.2*	153400349	rs9862101	0.9332	153110559	A	T	0.43	3.66E-05	1.54E-02	4.92E-02
rs4680057	Brain_Spinal_cord_cervical_c-1	3	*RP11-23D24.2*	153400349	rs2135774	0.9332	153113202	T	C	0.43	4.33E-05	1.69E-02	5.17E-02
rs4680057	Brain_Spinal_cord_cervical_c-1	3	*RP11-23D24.2*	153400349	rs1580988	0.8573	153114027	C	A	0.48	2.19E-04	8.81E-03	5.24E-02

rs4680057	Brain_Spinal_cord_cervical_c-1	3	*RP11-23D24.2*	153400349	rs6790236	0.9331	153115590	G	C	0.43	4.20E-05	1.54E-02	4.85E-02
rs4680057	Brain_Spinal_cord_cervical_c-1	3	*RP11-23D24.2*	153400349	rs6790408	0.9331	153115593	A	G	0.43	4.20E-05	1.54E-02	4.85E-02
rs4680057	Brain_Spinal_cord_cervical_c-1	3	*RP11-23D24.2*	153400349	rs9289909	0.9255	153116825	C	G	0.43	7.61E-05	3.27E-02	7.26E-02
rs4680057	Brain_Spinal_cord_cervical_c-1	3	*RP11-23D24.2*	153400349	rs6794116	0.8498	153116888	A	G	0.48	1.83E-04	7.70E-03	4.91E-02
rs4680057	Brain_Spinal_cord_cervical_c-1	3	*RP11-23D24.2*	153400349	rs9850272	0.9293	153116954	A	C	0.43	4.46E-05	1.54E-02	4.96E-02
rs4680057	Brain_Spinal_cord_cervical_c-1	3	*RP11-23D24.2*	153400349	rs9289910	0.9293	153117193	G	A	0.43	4.04E-05	1.54E-02	4.90E-02
rs4680057	Brain_Spinal_cord_cervical_c-1	3	*RP11-23D24.2*	153400349	rs1507160	0.8462	153117704	G	A	0.47	2.69E-04	8.35E-03	5.56E-02
rs4680057	Brain_Spinal_cord_cervical_c-1	3	*RP11-23D24.2*	153400349	rs10049279	0.8498	153118601	A	G	0.48	1.77E-04	7.84E-03	4.86E-02
rs4680057	Brain_Spinal_cord_cervical_c-1	3	*RP11-23D24.2*	153400349	rs4680059	0.8498	153118849	A	G	0.48	2.20E-04	3.55E-02	8.81E-02
rs4680057	Brain_Spinal_cord_cervical_c-1	3	*RP11-23D24.2*	153400349	rs12695988	0.9293	153122300	A	C	0.43	2.73E-05	2.02E-02	5.30E-02
rs4680057	Brain_Substantia_nigra	3	*ARHGEF26*	153907204	rs4680056	0.9523	153096430	G	A	0.43	5.13E-05	2.04E-02	4.31E-02
rs4680057	Brain_Substantia_nigra	3	*ARHGEF26*	153907204	rs9822507	0.9293	153096674	C	G	0.48	9.79E-02	4.47E-02	7.95E-02
rs4680057	Brain_Substantia_nigra	3	*ARHGEF26*	153907204	rs7627262	0.9489	153097522	T	C	0.43	1.84E-05	4.46E-02	8.33E-02
rs4680057	Brain_Substantia_nigra	3	*ARHGEF26*	153907204	rs7639345	0.9489	153097961	C	T	0.43	1.54E-05	4.46E-02	8.26E-02
rs4680057	Brain_Substantia_nigra	3	*ARHGEF26*	153907204	rs2135773	0.9489	153098130	T	C	0.43	1.79E-05	4.46E-02	8.29E-02
rs4680057	Brain_Substantia_nigra	3	*ARHGEF26*	153907204	rs1018179	0.9489	153098264	C	T	0.43	1.66E-05	4.46E-02	8.25E-02
rs4680057	Brain_Substantia_nigra	3	*ARHGEF26*	153907204	rs9852688	0.9489	153098558	C	T	0.43	1.66E-05	4.46E-02	8.25E-02
rs4680057	Brain_Substantia_nigra	3	*ARHGEF26*	153907204	rs955014	0.9451	153099380	C	T	0.43	1.37E-05	3.95E-02	7.44E-02
rs4680057	Brain_Substantia_nigra	3	*ARHGEF26*	153907204	rs13091512	0.9451	153099518	T	A	0.43	1.76E-05	2.36E-02	5.75E-02
rs4680057	Brain_Substantia_nigra	3	*ARHGEF26*	153907204	rs9843491	0.9412	153100477	A	G	0.43	2.01E-05	3.95E-02	7.67E-02
rs4680057	Brain_Substantia_nigra	3	*ARHGEF26*	153907204	rs4679708	0.9372	153102867	G	C	0.43	4.96E-05	3.95E-02	8.01E-02
rs4680057	Brain_Substantia_nigra	3	*ARHGEF26*	153907204	rs4679709	0.9332	153103018	G	A	0.43	5.10E-05	3.95E-02	8.02E-02
rs4680057	Brain_Substantia_nigra	3	*ARHGEF26*	153907204	rs1018176	0.9332	153103636	A	G	0.43	4.96E-05	3.95E-02	8.01E-02
rs4680057	Brain_Substantia_nigra	3	*ARHGEF26*	153907204	rs9864621	0.9294	153104577	T	C	0.43	2.99E-05	3.95E-02	7.83E-02
rs4680057	Brain_Substantia_nigra	3	*ARHGEF26*	153907204	rs9815053	0.9332	153105128	A	G	0.43	4.62E-05	3.95E-02	7.96E-02
rs4680057	Brain_Substantia_nigra	3	*ARHGEF26*	153907204	rs9815215	0.9332	153105380	T	C	0.43	2.97E-05	3.95E-02	7.71E-02
rs4680057	Brain_Substantia_nigra	3	*ARHGEF26*	153907204	rs9835730	0.9332	153105773	C	T	0.43	4.71E-05	3.95E-02	7.97E-02
rs4680057	Brain_Substantia_nigra	3	*ARHGEF26*	153907204	rs9819928	0.9332	153105912	A	G	0.43	4.07E-05	3.95E-02	7.93E-02
rs4680057	Brain_Substantia_nigra	3	*ARHGEF26*	153907204	rs9858222	0.9332	153106464	G	A	0.43	4.24E-05	3.95E-02	7.93E-02
rs4680057	Brain_Substantia_nigra	3	*ARHGEF26*	153907204	rs6777504	0.9294	153106837	T	C	0.43	3.60E-05	3.95E-02	7.88E-02
rs4680057	Brain_Substantia_nigra	3	*ARHGEF26*	153907204	rs9831443	0.9332	153108551	G	C	0.43	3.66E-05	3.95E-02	7.89E-02
rs4680057	Brain_Substantia_nigra	3	*ARHGEF26*	153907204	rs9862101	0.9332	153110559	A	T	0.43	3.66E-05	4.04E-02	7.99E-02
rs4680057	Brain_Substantia_nigra	3	*ARHGEF26*	153907204	rs2135774	0.9332	153113202	T	C	0.43	4.33E-05	3.49E-02	7.42E-02
rs4680057	Brain_Substantia_nigra	3	*ARHGEF26*	153907204	rs6790236	0.9331	153115590	G	C	0.43	4.20E-05	3.95E-02	7.83E-02
rs4680057	Brain_Substantia_nigra	3	*ARHGEF26*	153907204	rs6790408	0.9331	153115593	A	G	0.43	4.20E-05	3.95E-02	7.83E-02
rs4680057	Brain_Substantia_nigra	3	*ARHGEF26*	153907204	rs9850272	0.9293	153116954	A	C	0.43	4.46E-05	2.81E-02	6.62E-02
rs4680057	Brain_Substantia_nigra	3	*ARHGEF26*	153907204	rs9289910	0.9293	153117193	G	A	0.43	4.04E-05	3.95E-02	7.87E-02
rs4680057	Whole_Blood	3	*ARHGEF26*	153907204	rs4680056	0.9523	153096430	G	A	0.43	5.13E-05	1.16E-03	1.06E-02
rs4680057	Whole_Blood	3	*ARHGEF26*	153907204	rs13073679	0.9523	153096642	T	C	0.44	1.03E-03	1.53E-03	4.18E-02
rs4680057	Whole_Blood	3	*ARHGEF26*	153907204	rs9822507	0.9293	153096674	C	G	0.48	9.79E-02	1.09E-03	1.55E-02

rs4680057	Whole_Blood	3	*ARHGEF26*	153907204	rs4680057	1.0000	153096985	A	G	0.44	9.69E-07	1.31E-03	1.07E-02
rs4680057	Whole_Blood	3	*ARHGEF26*	153907204	rs7627262	0.9489	153097522	T	C	0.43	1.84E-05	4.44E-04	1.43E-02
rs4680057	Whole_Blood	3	*ARHGEF26*	153907204	rs7639345	0.9489	153097961	C	T	0.43	1.54E-05	5.79E-04	1.48E-02
rs4680057	Whole_Blood	3	*ARHGEF26*	153907204	rs2135773	0.9489	153098130	T	C	0.43	1.79E-05	7.72E-04	1.62E-02
rs4680057	Whole_Blood	3	*ARHGEF26*	153907204	rs1018179	0.9489	153098264	C	T	0.43	1.66E-05	5.79E-04	1.47E-02
rs4680057	Whole_Blood	3	*ARHGEF26*	153907204	rs9852688	0.9489	153098558	C	T	0.43	1.66E-05	5.45E-04	1.45E-02
rs4680057	Whole_Blood	3	*ARHGEF26*	153907204	rs955014	0.9451	153099380	C	T	0.43	1.37E-05	6.59E-04	1.37E-02
rs4680057	Whole_Blood	3	*ARHGEF26*	153907204	rs13091512	0.9451	153099518	T	A	0.43	1.76E-05	1.26E-03	1.77E-02
rs4680057	Whole_Blood	3	*ARHGEF26*	153907204	rs9843491	0.9412	153100477	A	G	0.43	2.01E-05	6.08E-04	1.48E-02
rs4680057	Whole_Blood	3	*ARHGEF26*	153907204	rs4679708	0.9372	153102867	G	C	0.43	4.96E-05	6.18E-04	1.71E-02
rs4680057	Whole_Blood	3	*ARHGEF26*	153907204	rs4679709	0.9332	153103018	G	A	0.43	5.10E-05	5.40E-04	1.67E-02
rs4680057	Whole_Blood	3	*ARHGEF26*	153907204	rs1018176	0.9332	153103636	A	G	0.43	4.96E-05	7.23E-04	1.78E-02
rs4680057	Whole_Blood	3	*ARHGEF26*	153907204	rs1018177	0.8535	153103714	G	A	0.48	1.88E-04	2.90E-03	3.79E-02
rs4680057	Whole_Blood	3	*ARHGEF26*	153907204	rs9864621	0.9294	153104577	T	C	0.43	2.99E-05	7.23E-04	1.66E-02
rs4680057	Whole_Blood	3	*ARHGEF26*	153907204	rs9815053	0.9332	153105128	A	G	0.43	4.62E-05	7.23E-04	1.75E-02
rs4680057	Whole_Blood	3	*ARHGEF26*	153907204	rs9815215	0.9332	153105380	T	C	0.43	2.97E-05	7.31E-04	1.59E-02
rs4680057	Whole_Blood	3	*ARHGEF26*	153907204	rs9835730	0.9332	153105773	C	T	0.43	4.71E-05	7.23E-04	1.76E-02
rs4680057	Whole_Blood	3	*ARHGEF26*	153907204	rs9819928	0.9332	153105912	A	G	0.43	4.07E-05	7.23E-04	1.73E-02
rs4680057	Whole_Blood	3	*ARHGEF26*	153907204	rs9858222	0.9332	153106464	G	A	0.43	4.24E-05	5.86E-04	1.64E-02
rs4680057	Whole_Blood	3	*ARHGEF26*	153907204	rs6777504	0.9294	153106837	T	C	0.43	3.60E-05	7.23E-04	1.69E-02
rs4680057	Whole_Blood	3	*ARHGEF26*	153907204	rs9831094	0.8535	153108239	A	G	0.48	1.61E-04	3.02E-03	3.72E-02
rs4680057	Whole_Blood	3	*ARHGEF26*	153907204	rs9831443	0.9332	153108551	G	C	0.43	3.66E-05	7.23E-04	1.70E-02
rs4680057	Whole_Blood	3	*ARHGEF26*	153907204	rs1827993	0.9294	153110407	T	G	0.43	7.17E-05	9.90E-04	1.95E-02
rs4680057	Whole_Blood	3	*ARHGEF26*	153907204	rs9862101	0.9332	153110559	A	T	0.43	3.66E-05	6.78E-04	1.67E-02
rs4680057	Whole_Blood	3	*ARHGEF26*	153907204	rs2135774	0.9332	153113202	T	C	0.43	4.33E-05	7.60E-04	1.75E-02
rs4680057	Whole_Blood	3	*ARHGEF26*	153907204	rs1580988	0.8573	153114027	C	A	0.48	2.19E-04	4.59E-03	4.31E-02
rs4680057	Whole_Blood	3	*ARHGEF26*	153907204	rs6790236	0.9331	153115590	G	C	0.43	4.20E-05	5.24E-04	1.52E-02
rs4680057	Whole_Blood	3	*ARHGEF26*	153907204	rs6790408	0.9331	153115593	A	G	0.43	4.20E-05	5.24E-04	1.52E-02
rs4680057	Whole_Blood	3	*ARHGEF26*	153907204	rs3903613	0.8498	153116369	C	T	0.48	1.75E-04	1.61E-03	3.18E-02
rs4680057	Whole_Blood	3	*ARHGEF26*	153907204	rs9289909	0.9255	153116825	C	G	0.43	7.61E-05	8.92E-04	1.89E-02
rs4680057	Whole_Blood	3	*ARHGEF26*	153907204	rs6794116	0.8498	153116888	A	G	0.48	1.83E-04	2.72E-03	3.65E-02
rs4680057	Whole_Blood	3	*ARHGEF26*	153907204	rs9850272	0.9293	153116954	A	C	0.43	4.46E-05	1.11E-03	1.94E-02
rs4680057	Whole_Blood	3	*ARHGEF26*	153907204	rs9289910	0.9293	153117193	G	A	0.43	4.04E-05	5.97E-04	1.61E-02
rs4680057	Whole_Blood	3	*ARHGEF26*	153907204	rs1507160	0.8462	153117704	G	A	0.47	2.69E-04	3.41E-03	4.37E-02
rs4680057	Whole_Blood	3	*ARHGEF26*	153907204	rs10049279	0.8498	153118601	A	G	0.48	1.77E-04	1.73E-03	3.20E-02
rs4680057	Whole_Blood	3	*ARHGEF26*	153907204	rs4680059	0.8498	153118849	A	G	0.48	2.20E-04	1.66E-03	3.25E-02
rs4680057	Whole_Blood	3	*ARHGEF26*	153907204	rs12695988	0.9293	153122300	A	C	0.43	2.73E-05	1.43E-03	1.85E-02
rs12908891	Brain_Amygdala	15	*RAB8B*	63520824	rs1460544	0.8736	64201766	T	C	0.51	9.06E-04	4.32E-02	1.61E-01
rs12908891	Brain_Amygdala	15	*SNX22*	64446798	rs1460544	0.8736	64201766	T	C	0.51	9.06E-04	3.81E-02	1.56E-01
rs12908891	Brain_Amygdala	15	*APH1B*	63584771	rs1037846	0.8696	64204870	G	A	0.51	6.33E-04	2.95E-02	6.06E-02

rs12908891	Brain_Amygdala	15	*SNX22*	64446798	rs1037846	0.8696	64204870	G	A	0.51	6.33E-04	4.99E-02	8.31E-02
rs12908891	Brain_Amygdala	15	*RAB8B*	63520824	rs11071773	0.8883	64206765	A	G	0.51	8.95E-04	4.27E-02	1.34E-01
rs12908891	Brain_Amygdala	15	*RAB8B*	63520824	rs12909081	0.8809	64208260	A	C	0.51	1.00E-03	4.27E-02	1.38E-01
rs12908891	Brain_Amygdala	15	*RAB8B*	63520824	rs2414844	0.8846	64210675	A	G	0.51	9.01E-04	4.27E-02	1.35E-01
rs12908891	Brain_Amygdala	15	*RAB8B*	63520824	rs12592060	0.8846	64210780	T	C	0.51	9.01E-04	4.27E-02	1.35E-01
rs12908891	Brain_Amygdala	15	*RAB8B*	63520824	rs1471282	0.8846	64212275	G	T	0.51	1.07E-03	4.27E-02	1.41E-01
rs12908891	Brain_Amygdala	15	*RAB8B*	63520824	rs11635779	0.8808	64213826	T	C	0.50	6.34E-05	4.37E-02	8.57E-02
rs12908891	Brain_Amygdala	15	*RAB8B*	63520824	rs12904374	0.8808	64214670	T	A	0.51	8.63E-04	4.37E-02	7.92E-02
rs12908891	Brain_Amygdala	15	*APH1B*	63584771	rs920762	0.9379	64214954	C	G	0.47	1.24E-05	3.48E-02	5.47E-02
rs12908891	Brain_Amygdala	15	*RAB8B*	63520824	rs12907405	0.8884	64220046	T	C	0.50	4.35E-05	1.55E-02	4.87E-02
rs12908891	Brain_Amygdala	15	*RAB8B*	63520824	rs11071775	0.8808	64220995	A	G	0.50	4.81E-05	1.39E-02	4.65E-02
rs12908891	Brain_Amygdala	15	*RAB8B*	63520824	rs11071776	0.8808	64221010	T	A	0.50	5.49E-05	1.39E-02	4.74E-02
rs12908891	Brain_Amygdala	15	*RAB8B*	63520824	rs11637858	0.8959	64224107	C	T	0.49	4.60E-05	9.55E-03	3.91E-02
rs12908891	Brain_Amygdala	15	*RAB8B*	63520824	rs12916395	0.9921	64225563	T	C	0.51	1.86E-06	2.38E-02	3.98E-02
rs12908891	Brain_Amygdala	15	*RAB8B*	63520824	rs11630587	0.9921	64225908	C	T	0.51	1.77E-06	2.04E-02	3.57E-02
rs12908891	Brain_Amygdala	15	*APH1B*	63584771	rs11630587	0.9921	64225908	C	T	0.51	1.77E-06	4.56E-02	6.38E-02
rs12908891	Brain_Amygdala	15	*RAB8B*	63520824	rs4776677	0.9921	64227044	C	T	0.51	1.54E-06	1.04E-02	2.27E-02
rs12908891	Brain_Amygdala	15	*APH1B*	63584771	rs4776677	0.9921	64227044	C	T	0.51	1.54E-06	3.82E-02	5.57E-02
rs12908891	Brain_Amygdala	15	*RAB8B*	63520824	rs11071777	0.8557	64229285	C	G	0.55	9.20E-06	4.65E-02	6.74E-02
rs12908891	Brain_Amygdala	15	*APH1B*	63584771	rs11071777	0.8557	64229285	C	G	0.55	9.20E-06	8.73E-03	2.25E-02
rs12908891	Brain_Amygdala	15	*SNX1*	64412305	rs11071777	0.8557	64229285	C	G	0.55	9.20E-06	3.31E-02	5.29E-02
rs12908891	Brain_Amygdala	15	*RAB8B*	63520824	rs1563886	0.9272	64230852	G	C	0.49	8.06E-06	9.14E-03	4.12E-02
rs12908891	Brain_Amygdala	15	*RAB8B*	63520824	rs10744961	0.9960	64230930	A	G	0.51	1.86E-06	2.04E-02	3.61E-02
rs12908891	Brain_Amygdala	15	*APH1B*	63584771	rs10744961	0.9960	64230930	A	G	0.51	1.86E-06	4.56E-02	6.42E-02
rs12908891	Brain_Amygdala	15	*RAB8B*	63520824	rs6494454	0.8031	64233563	A	C	0.56	2.52E-05	7.44E-03	2.37E-02
rs12908891	Brain_Amygdala	15	*RAB8B*	63520824	rs894660	0.8031	64234334	C	T	0.56	2.52E-05	7.44E-03	2.37E-02
rs12908891	Brain_Amygdala	15	*RAB8B*	63520824	rs4776266	0.8031	64236175	G	T	0.56	2.23E-05	7.44E-03	2.35E-02
rs12908891	Brain_Amygdala	15	*RAB8B*	63520824	rs4776268	1.0000	64236398	A	G	0.51	6.19E-06	1.47E-02	6.59E-02
rs12908891	Brain_Amygdala	15	*RAB8B*	63520824	rs12908891	1.0000	64236441	A	G	0.52	1.39E-06	1.98E-02	3.51E-02
rs12908891	Brain_Amygdala	15	*RAB8B*	63520824	rs11071779	0.9881	64239044	G	A	0.51	1.57E-06	1.84E-02	3.39E-02
rs12908891	Brain_Amygdala	15	*RAB8B*	63520824	rs11071780	0.9842	64241691	A	G	0.51	4.74E-06	2.04E-02	7.68E-02
rs12908891	Brain_Amygdala	15	*RAB8B*	63520824	rs11854537	0.9842	64241733	A	G	0.51	4.74E-06	2.04E-02	7.68E-02
rs12908891	Brain_Amygdala	15	*RAB8B*	63520824	rs1380844	0.9881	64242007	G	T	0.51	4.74E-06	2.04E-02	7.68E-02
rs12908891	Brain_Amygdala	15	*RAB8B*	63520824	rs12916806	0.9881	64242770	C	A	0.51	4.64E-06	2.04E-02	7.67E-02
rs12908891	Brain_Amygdala	15	*RAB8B*	63520824	rs11071781	0.9195	64243933	G	A	0.49	4.75E-06	1.35E-02	4.98E-02
rs12908891	Brain_Amygdala	15	*RAB8B*	63520824	rs1304365	0.9881	64245946	A	G	0.51	4.67E-06	2.04E-02	7.68E-02
rs12908891	Brain_Amygdala	15	*RAB8B*	63520824	rs115415409	0.8420	64249559	T	C	0.45	1.45E-05	2.48E-02	4.97E-02
rs12908891	Brain_Amygdala	15	*USP3*	63841816	rs115415409	0.8420	64249559	T	C	0.45	1.45E-05	3.30E-02	5.93E-02
rs12908891	Brain_Amygdala	15	*RAB8B*	63520824	rs145159458	0.9119	64252880	T	C	0.49	1.21E-05	1.30E-02	5.94E-02
rs12908891	Brain_Amygdala	15	*RAB8B*	63520824	rs4776692	0.9881	64255083	G	A	0.50	1.06E-05	2.04E-02	8.61E-02

rs12908891	Brain_Amygdala	15	*RAB8B*	63520824	rs28822416	0.8420	64257845	C	T	0.45	1.54E-05	3.76E-02	6.47E-02
rs12908891	Brain_Amygdala	15	*RAB8B*	63520824	rs8035776	0.8420	64258018	G	A	0.45	1.54E-05	3.76E-02	6.47E-02
rs12908891	Brain_Amygdala	15	*RAB8B*	63520824	rs6494456	0.8381	64258260	C	T	0.45	1.54E-05	3.76E-02	6.47E-02
rs12908891	Brain_Amygdala	15	*RAB8B*	63520824	rs4776695	0.8350	64259115	T	C	0.46	9.12E-06	1.47E-02	3.42E-02
rs12908891	Brain_Amygdala	15	*OAZ2*	64987626	rs4776695	0.8350	64259115	T	C	0.46	9.12E-06	4.57E-02	7.03E-02
rs12908891	Brain_Anterior_cingulate_cortex_BA24	15	*HERC1*	64013479	rs1460544	0.8736	64201766	T	C	0.51	9.06E-04	1.95E-04	8.49E-02
rs12908891	Brain_Anterior_cingulate_cortex_BA24	15	*DAPK2*	64281733	rs1460544	0.8736	64201766	T	C	0.51	9.06E-04	5.55E-03	1.12E-01
rs12908891	Brain_Anterior_cingulate_cortex_BA24	15	*HERC1*	64013479	rs1037846	0.8696	64204870	G	A	0.51	6.33E-04	7.97E-05	6.94E-03
rs12908891	Brain_Anterior_cingulate_cortex_BA24	15	*DAPK2*	64281733	rs1037846	0.8696	64204870	G	A	0.51	6.33E-04	2.20E-03	1.83E-02
rs12908891	Brain_Anterior_cingulate_cortex_BA24	15	*HERC1*	64013479	rs11071773	0.8883	64206765	A	G	0.51	8.95E-04	9.19E-05	5.36E-02
rs12908891	Brain_Anterior_cingulate_cortex_BA24	15	*DAPK2*	64281733	rs11071773	0.8883	64206765	A	G	0.51	8.95E-04	3.05E-03	7.57E-02
rs12908891	Brain_Anterior_cingulate_cortex_BA24	15	*HERC1*	64013479	rs12909081	0.8809	64208260	A	C	0.51	1.00E-03	9.19E-05	5.74E-02
rs12908891	Brain_Anterior_cingulate_cortex_BA24	15	*DAPK2*	64281733	rs12909081	0.8809	64208260	A	C	0.51	1.00E-03	3.05E-03	7.96E-02
rs12908891	Brain_Anterior_cingulate_cortex_BA24	15	*HERC1*	64013479	rs749468	0.9493	64210279	T	C	0.53	2.79E-04	9.14E-05	6.88E-03
rs12908891	Brain_Anterior_cingulate_cortex_BA24	15	*DAPK2*	64281733	rs749468	0.9493	64210279	T	C	0.53	2.79E-04	4.56E-03	2.39E-02
rs12908891	Brain_Anterior_cingulate_cortex_BA24	15	*HERC1*	64013479	rs2414844	0.8846	64210675	A	G	0.51	9.01E-04	9.19E-05	5.44E-02
rs12908891	Brain_Anterior_cingulate_cortex_BA24	15	*DAPK2*	64281733	rs2414844	0.8846	64210675	A	G	0.51	9.01E-04	3.05E-03	7.65E-02
rs12908891	Brain_Anterior_cingulate_cortex_BA24	15	*HERC1*	64013479	rs12592060	0.8846	64210780	T	C	0.51	9.01E-04	9.19E-05	5.44E-02
rs12908891	Brain_Anterior_cingulate_cortex_BA24	15	*DAPK2*	64281733	rs12592060	0.8846	64210780	T	C	0.51	9.01E-04	3.05E-03	7.65E-02
rs12908891	Brain_Anterior_cingulate_cortex_BA24	15	*HERC1*	64013479	rs1471282	0.8846	64212275	G	T	0.51	1.07E-03	9.19E-05	6.07E-02
rs12908891	Brain_Anterior_cingulate_cortex_BA24	15	*DAPK2*	64281733	rs1471282	0.8846	64212275	G	T	0.51	1.07E-03	3.05E-03	8.30E-02
rs12908891	Brain_Anterior_cingulate_cortex_BA24	15	*HERC1*	64013479	rs11635779	0.8808	64213826	T	C	0.50	6.34E-05	9.33E-05	1.20E-02
rs12908891	Brain_Anterior_cingulate_cortex_BA24	15	*DAPK2*	64281733	rs11635779	0.8808	64213826	T	C	0.50	6.34E-05	1.83E-03	2.38E-02
rs12908891	Brain_Anterior_cingulate_cortex_BA24	15	*HERC1*	64013479	rs12904374	0.8808	64214670	T	A	0.51	8.63E-04	9.33E-05	8.48E-03
rs12908891	Brain_Anterior_cingulate_cortex_BA24	15	*DAPK2*	64281733	rs12904374	0.8808	64214670	T	A	0.51	8.63E-04	1.83E-03	1.90E-02
rs12908891	Brain_Anterior_cingulate_cortex_BA24	15	*HERC1*	64013479	rs920762	0.9379	64214954	C	G	0.47	1.24E-05	6.51E-05	2.47E-03
rs12908891	Brain_Anterior_cingulate_cortex_BA24	15	*DAPK2*	64281733	rs920762	0.9379	64214954	C	G	0.47	1.24E-05	3.38E-03	1.32E-02
rs12908891	Brain_Anterior_cingulate_cortex_BA24	15	*HERC1*	64013479	rs11853632	0.8921	64217845	C	G	0.50	4.21E-05	3.66E-05	8.59E-03
rs12908891	Brain_Anterior_cingulate_cortex_BA24	15	*DAPK2*	64281733	rs11853632	0.8921	64217845	C	G	0.50	4.21E-05	1.62E-03	2.06E-02
rs12908891	Brain_Anterior_cingulate_cortex_BA24	15	*HERC1*	64013479	rs12907405	0.8884	64220046	T	C	0.50	4.35E-05	9.19E-05	1.03E-02
rs12908891	Brain_Anterior_cingulate_cortex_BA24	15	*DAPK2*	64281733	rs12907405	0.8884	64220046	T	C	0.50	4.35E-05	3.05E-03	2.56E-02
rs12908891	Brain_Anterior_cingulate_cortex_BA24	15	*HERC1*	64013479	rs11071775	0.8808	64220995	A	G	0.50	4.81E-05	9.19E-05	1.04E-02
rs12908891	Brain_Anterior_cingulate_cortex_BA24	15	*DAPK2*	64281733	rs11071775	0.8808	64220995	A	G	0.50	4.81E-05	3.05E-03	2.56E-02
rs12908891	Brain_Anterior_cingulate_cortex_BA24	15	*HERC1*	64013479	rs11071776	0.8808	64221010	T	A	0.50	5.49E-05	9.19E-05	1.09E-02
rs12908891	Brain_Anterior_cingulate_cortex_BA24	15	*DAPK2*	64281733	rs11071776	0.8808	64221010	T	A	0.50	5.49E-05	3.05E-03	2.64E-02
rs12908891	Brain_Anterior_cingulate_cortex_BA24	15	*HERC1*	64013479	rs11637858	0.8959	64224107	C	T	0.49	4.60E-05	7.76E-05	9.83E-03
rs12908891	Brain_Anterior_cingulate_cortex_BA24	15	*DAPK2*	64281733	rs11637858	0.8959	64224107	C	T	0.49	4.60E-05	6.06E-03	3.25E-02
rs12908891	Brain_Anterior_cingulate_cortex_BA24	15	*HERC1*	64013479	rs12916395	0.9921	64225563	T	C	0.51	1.86E-06	8.12E-05	2.05E-03
rs12908891	Brain_Anterior_cingulate_cortex_BA24	15	*DAPK2*	64281733	rs12916395	0.9921	64225563	T	C	0.51	1.86E-06	1.19E-02	2.49E-02
rs12908891	Brain_Anterior_cingulate_cortex_BA24	15	*HERC1*	64013479	rs11630587	0.9921	64225908	C	T	0.51	1.77E-06	2.48E-04	3.21E-03

rs12908891	Brain_Anterior_cingulate_cortex_BA24	15	*DAPK2*	64281733	rs11630587	0.9921	64225908	C	T	0.51	1.77E-06	1.59E-02	3.01E-02
rs12908891	Brain_Anterior_cingulate_cortex_BA24	15	*HERC1*	64013479	rs4776677	0.9921	64227044	C	T	0.51	1.54E-06	5.11E-05	1.68E-03
rs12908891	Brain_Anterior_cingulate_cortex_BA24	15	*DAPK2*	64281733	rs4776677	0.9921	64227044	C	T	0.51	1.54E-06	1.03E-02	2.26E-02
rs12908891	Brain_Anterior_cingulate_cortex_BA24	15	*HERC1*	64013479	rs11071777	0.8557	64229285	C	G	0.55	9.20E-06	5.29E-06	1.17E-03
rs12908891	Brain_Anterior_cingulate_cortex_BA24	15	*DAPK2*	64281733	rs11071777	0.8557	64229285	C	G	0.55	9.20E-06	3.21E-02	5.18E-02
rs12908891	Brain_Anterior_cingulate_cortex_BA24	15	*HERC1*	64013479	rs1563886	0.9272	64230852	G	C	0.49	8.06E-06	9.64E-05	1.20E-02
rs12908891	Brain_Anterior_cingulate_cortex_BA24	15	*DAPK2*	64281733	rs1563886	0.9272	64230852	G	C	0.49	8.06E-06	9.08E-03	4.11E-02
rs12908891	Brain_Anterior_cingulate_cortex_BA24	15	*HERC1*	64013479	rs10744961	0.9960	64230930	A	G	0.51	1.86E-06	5.11E-05	1.80E-03
rs12908891	Brain_Anterior_cingulate_cortex_BA24	15	*DAPK2*	64281733	rs10744961	0.9960	64230930	A	G	0.51	1.86E-06	1.03E-02	2.31E-02
rs12908891	Brain_Anterior_cingulate_cortex_BA24	15	*HERC1*	64013479	rs6494454	0.8031	64233563	A	C	0.56	2.52E-05	1.61E-03	1.14E-02
rs12908891	Brain_Anterior_cingulate_cortex_BA24	15	*HERC1*	64013479	rs894660	0.8031	64234334	C	T	0.56	2.52E-05	1.61E-03	1.14E-02
rs12908891	Brain_Anterior_cingulate_cortex_BA24	15	*HERC1*	64013479	rs4776266	0.8031	64236175	G	T	0.56	2.23E-05	1.61E-03	1.13E-02
rs12908891	Brain_Anterior_cingulate_cortex_BA24	15	*HERC1*	64013479	rs4776268	1.0000	64236398	A	G	0.51	6.19E-06	7.87E-05	2.24E-02
rs12908891	Brain_Anterior_cingulate_cortex_BA24	15	*DAPK2*	64281733	rs4776268	1.0000	64236398	A	G	0.51	6.19E-06	1.52E-02	6.66E-02
rs12908891	Brain_Anterior_cingulate_cortex_BA24	15	*HERC1*	64013479	rs12908891	1.0000	64236441	A	G	0.52	1.39E-06	7.02E-05	1.94E-03
rs12908891	Brain_Anterior_cingulate_cortex_BA24	15	*DAPK2*	64281733	rs12908891	1.0000	64236441	A	G	0.52	1.39E-06	1.40E-02	2.77E-02
rs12908891	Brain_Anterior_cingulate_cortex_BA24	15	*HERC1*	64013479	rs11071779	0.9881	64239044	G	A	0.51	1.57E-06	2.29E-05	1.40E-03
rs12908891	Brain_Anterior_cingulate_cortex_BA24	15	*DAPK2*	64281733	rs11071779	0.9881	64239044	G	A	0.51	1.57E-06	2.16E-02	3.77E-02
rs12908891	Brain_Anterior_cingulate_cortex_BA24	15	*HERC1*	64013479	rs11071780	0.9842	64241691	A	G	0.51	4.74E-06	2.52E-05	2.17E-02
rs12908891	Brain_Anterior_cingulate_cortex_BA24	15	*DAPK2*	64281733	rs11071780	0.9842	64241691	A	G	0.51	4.74E-06	3.41E-02	9.38E-02
rs12908891	Brain_Anterior_cingulate_cortex_BA24	15	*HERC1*	64013479	rs11854537	0.9842	64241733	A	G	0.51	4.74E-06	2.52E-05	2.17E-02
rs12908891	Brain_Anterior_cingulate_cortex_BA24	15	*DAPK2*	64281733	rs11854537	0.9842	64241733	A	G	0.51	4.74E-06	3.41E-02	9.38E-02
rs12908891	Brain_Anterior_cingulate_cortex_BA24	15	*HERC1*	64013479	rs1380844	0.9881	64242007	G	T	0.51	4.74E-06	2.52E-05	2.17E-02
rs12908891	Brain_Anterior_cingulate_cortex_BA24	15	*DAPK2*	64281733	rs1380844	0.9881	64242007	G	T	0.51	4.74E-06	3.41E-02	9.38E-02
rs12908891	Brain_Anterior_cingulate_cortex_BA24	15	*HERC1*	64013479	rs12916806	0.9881	64242770	C	A	0.51	4.64E-06	2.52E-05	2.17E-02
rs12908891	Brain_Anterior_cingulate_cortex_BA24	15	*DAPK2*	64281733	rs12916806	0.9881	64242770	C	A	0.51	4.64E-06	3.41E-02	9.37E-02
rs12908891	Brain_Anterior_cingulate_cortex_BA24	15	*HERC1*	64013479	rs11071781	0.9195	64243933	G	A	0.49	4.75E-06	2.22E-05	1.02E-02
rs12908891	Brain_Anterior_cingulate_cortex_BA24	15	*DAPK2*	64281733	rs11071781	0.9195	64243933	G	A	0.49	4.75E-06	9.02E-03	4.23E-02
rs12908891	Brain_Anterior_cingulate_cortex_BA24	15	*HERC1*	64013479	rs1304365	0.9881	64245946	A	G	0.51	4.67E-06	2.29E-05	2.15E-02
rs12908891	Brain_Anterior_cingulate_cortex_BA24	15	*DAPK2*	64281733	rs1304365	0.9881	64245946	A	G	0.51	4.67E-06	2.16E-02	7.84E-02
rs12908891	Brain_Anterior_cingulate_cortex_BA24	15	*HERC1*	64013479	rs115415409	0.8420	64249559	T	C	0.45	1.45E-05	1.52E-04	5.70E-03
rs12908891	Brain_Anterior_cingulate_cortex_BA24	15	*DAPK2*	64281733	rs115415409	0.8420	64249559	T	C	0.45	1.45E-05	3.05E-02	5.65E-02
rs12908891	Brain_Anterior_cingulate_cortex_BA24	15	*HERC1*	64013479	rs145159458	0.9119	64252880	T	C	0.49	1.21E-05	2.86E-05	1.72E-02
rs12908891	Brain_Anterior_cingulate_cortex_BA24	15	*DAPK2*	64281733	rs145159458	0.9119	64252880	T	C	0.49	1.21E-05	1.33E-02	5.98E-02
rs12908891	Brain_Anterior_cingulate_cortex_BA24	15	*HERC1*	64013479	rs4776692	0.9881	64255083	G	A	0.50	1.06E-05	2.52E-05	2.90E-02
rs12908891	Brain_Anterior_cingulate_cortex_BA24	15	*DAPK2*	64281733	rs4776692	0.9881	64255083	G	A	0.50	1.06E-05	3.41E-02	1.03E-01
rs12908891	Brain_Anterior_cingulate_cortex_BA24	15	*HERC1*	64013479	rs28822416	0.8420	64257845	C	T	0.45	1.54E-05	5.53E-05	4.39E-03
rs12908891	Brain_Anterior_cingulate_cortex_BA24	15	*DAPK2*	64281733	rs28822416	0.8420	64257845	C	T	0.45	1.54E-05	3.89E-02	6.61E-02
rs12908891	Brain_Anterior_cingulate_cortex_BA24	15	*HERC1*	64013479	rs8035776	0.8420	64258018	G	A	0.45	1.54E-05	5.53E-05	4.39E-03
rs12908891	Brain_Anterior_cingulate_cortex_BA24	15	*DAPK2*	64281733	rs8035776	0.8420	64258018	G	A	0.45	1.54E-05	3.89E-02	6.61E-02

rs12908891	Brain_Anterior_cingulate_cortex_BA24	15	*HERC1*	64013479	rs6494456	0.8381	64258260	C	T	0.45	1.54E-05	5.53E-05	4.39E-03
rs12908891	Brain_Anterior_cingulate_cortex_BA24	15	*DAPK2*	64281733	rs6494456	0.8381	64258260	C	T	0.45	1.54E-05	3.89E-02	6.61E-02
rs12908891	Brain_Anterior_cingulate_cortex_BA24	15	*HERC1*	64013479	rs4776695	0.8350	64259115	T	C	0.46	9.12E-06	9.66E-05	3.98E-03
rs12908891	Brain_Anterior_cingulate_cortex_BA24	15	*KIAA0101*	64668539	rs4776695	0.8350	64259115	T	C	0.46	9.12E-06	4.15E-02	6.58E-02
rs12908891	Brain_Caudate_basal_ganglia	15	*RP11-321G12.1*	63705841	rs11853632	0.8921	64217845	C	G	0.50	4.21E-05	2.95E-02	6.66E-02
rs12908891	Brain_Caudate_basal_ganglia	15	*RP11-321G12.1*	63705841	rs12907405	0.8884	64220046	T	C	0.50	4.35E-05	3.58E-02	7.42E-02
rs12908891	Brain_Caudate_basal_ganglia	15	*RP11-321G12.1*	63705841	rs11071775	0.8808	64220995	A	G	0.50	4.81E-05	3.55E-02	7.39E-02
rs12908891	Brain_Caudate_basal_ganglia	15	*RP11-321G12.1*	63705841	rs11071776	0.8808	64221010	T	A	0.50	5.49E-05	3.55E-02	7.49E-02
rs12908891	Brain_Caudate_basal_ganglia	15	*RP11-321G12.1*	63705841	rs11637858	0.8959	64224107	C	T	0.49	4.60E-05	3.55E-02	7.35E-02
rs12908891	Brain_Caudate_basal_ganglia	15	*APH1B*	63584771	rs11071777	0.8557	64229285	C	G	0.55	9.20E-06	2.56E-02	4.43E-02
rs12908891	Brain_Caudate_basal_ganglia	15	*RP11-321G12.1*	63705841	rs1563886	0.9272	64230852	G	C	0.49	8.06E-06	4.21E-02	8.39E-02
rs12908891	Brain_Caudate_basal_ganglia	15	*RP11-321G12.1*	63705841	rs4776268	1.0000	64236398	A	G	0.51	6.19E-06	1.43E-02	6.53E-02
rs12908891	Brain_Caudate_basal_ganglia	15	*RP11-321G12.1*	63705841	rs11071779	0.9881	64239044	G	A	0.51	1.57E-06	1.78E-02	3.31E-02
rs12908891	Brain_Caudate_basal_ganglia	15	*RP11-321G12.1*	63705841	rs11071780	0.9842	64241691	A	G	0.51	4.74E-06	1.95E-02	7.56E-02
rs12908891	Brain_Caudate_basal_ganglia	15	*RP11-321G12.1*	63705841	rs11854537	0.9842	64241733	A	G	0.51	4.74E-06	1.95E-02	7.56E-02
rs12908891	Brain_Caudate_basal_ganglia	15	*RP11-321G12.1*	63705841	rs1380844	0.9881	64242007	G	T	0.51	4.74E-06	1.95E-02	7.56E-02
rs12908891	Brain_Caudate_basal_ganglia	15	*RP11-321G12.1*	63705841	rs12916806	0.9881	64242770	C	A	0.51	4.64E-06	1.95E-02	7.56E-02
rs12908891	Brain_Caudate_basal_ganglia	15	*RP11-321G12.1*	63705841	rs11071781	0.9195	64243933	G	A	0.49	4.75E-06	2.62E-02	6.70E-02
rs12908891	Brain_Caudate_basal_ganglia	15	*RP11-321G12.1*	63705841	rs1304365	0.9881	64245946	A	G	0.51	4.67E-06	9.76E-03	6.02E-02
rs12908891	Brain_Caudate_basal_ganglia	15	*RP11-321G12.1*	63705841	rs115415409	0.8420	64249559	T	C	0.45	1.45E-05	2.17E-02	4.59E-02
rs12908891	Brain_Caudate_basal_ganglia	15	*RP11-321G12.1*	63705841	rs145159458	0.9119	64252880	T	C	0.49	1.21E-05	3.11E-02	8.37E-02
rs12908891	Brain_Caudate_basal_ganglia	15	*RP11-321G12.1*	63705841	rs4776692	0.9881	64255083	G	A	0.50	1.06E-05	1.95E-02	8.49E-02
rs12908891	Brain_Caudate_basal_ganglia	15	*RP11-321G12.1*	63705841	rs28822416	0.8420	64257845	C	T	0.45	1.54E-05	2.41E-02	4.91E-02
rs12908891	Brain_Caudate_basal_ganglia	15	*RP11-321G12.1*	63705841	rs8035776	0.8420	64258018	G	A	0.45	1.54E-05	2.41E-02	4.91E-02
rs12908891	Brain_Caudate_basal_ganglia	15	*RP11-321G12.1*	63705841	rs6494456	0.8381	64258260	C	T	0.45	1.54E-05	2.41E-02	4.91E-02
rs12908891	Brain_Caudate_basal_ganglia	15	*RP11-321G12.1*	63705841	rs4776695	0.8350	64259115	T	C	0.46	9.12E-06	1.28E-02	3.15E-02
rs12908891	Brain_Cerebellar_Hemisphere	15	*ANKDD1A*	65227571	rs11637858	0.8959	64224107	C	T	0.49	4.60E-05	3.27E-02	7.03E-02
rs12908891	Brain_Cerebellar_Hemisphere	15	*ANKDD1A*	65227571	rs12916395	0.9921	64225563	T	C	0.51	1.86E-06	4.03E-02	5.82E-02
rs12908891	Brain_Cerebellar_Hemisphere	15	*ANKDD1A*	65227571	rs11630587	0.9921	64225908	C	T	0.51	1.77E-06	2.84E-02	4.50E-02
rs12908891	Brain_Cerebellar_Hemisphere	15	*ANKDD1A*	65227571	rs4776677	0.9921	64227044	C	T	0.51	1.54E-06	3.07E-02	4.75E-02
rs12908891	Brain_Cerebellar_Hemisphere	15	*ANKDD1A*	65227571	rs1563886	0.9272	64230852	G	C	0.49	8.06E-06	2.20E-02	6.03E-02
rs12908891	Brain_Cerebellar_Hemisphere	15	*ANKDD1A*	65227571	rs10744961	0.9960	64230930	A	G	0.51	1.86E-06	3.04E-02	4.77E-02
rs12908891	Brain_Cerebellar_Hemisphere	15	*USP3-AS1*	63864970	rs6494454	0.8031	64233563	A	C	0.56	2.52E-05	3.90E-02	6.35E-02
rs12908891	Brain_Cerebellar_Hemisphere	15	*ANKDD1A*	65227571	rs6494454	0.8031	64233563	A	C	0.56	2.52E-05	1.66E-02	3.70E-02
rs12908891	Brain_Cerebellar_Hemisphere	15	*USP3-AS1*	63864970	rs894660	0.8031	64234334	C	T	0.56	2.52E-05	3.90E-02	6.35E-02
rs12908891	Brain_Cerebellar_Hemisphere	15	*ANKDD1A*	65227571	rs894660	0.8031	64234334	C	T	0.56	2.52E-05	1.66E-02	3.70E-02
rs12908891	Brain_Cerebellar_Hemisphere	15	*USP3-AS1*	63864970	rs4776266	0.8031	64236175	G	T	0.56	2.23E-05	4.33E-02	6.80E-02
rs12908891	Brain_Cerebellar_Hemisphere	15	*SNX22*	64446798	rs4776266	0.8031	64236175	G	T	0.56	2.23E-05	4.73E-02	7.23E-02
rs12908891	Brain_Cerebellar_Hemisphere	15	*ANKDD1A*	65227571	rs4776266	0.8031	64236175	G	T	0.56	2.23E-05	2.39E-02	4.61E-02
rs12908891	Brain_Cerebellar_Hemisphere	15	*ANKDD1A*	65227571	rs4776268	1.0000	64236398	A	G	0.51	6.19E-06	1.27E-02	6.27E-02

rs12908891	Brain_Cerebellar_Hemisphere	15	*ANKDD1A*	65227571	rs12908891	1.0000	64236441	A	G	0.52	1.39E-06	3.20E-02	4.90E-02
rs12908891	Brain_Cerebellar_Hemisphere	15	*ANKDD1A*	65227571	rs11071779	0.9881	64239044	G	A	0.51	1.57E-06	3.56E-02	5.37E-02
rs12908891	Brain_Cerebellar_Hemisphere	15	*ANKDD1A*	65227571	rs11071780	0.9842	64241691	A	G	0.51	4.74E-06	2.78E-02	8.63E-02
rs12908891	Brain_Cerebellar_Hemisphere	15	*ANKDD1A*	65227571	rs11854537	0.9842	64241733	A	G	0.51	4.74E-06	2.78E-02	8.63E-02
rs12908891	Brain_Cerebellar_Hemisphere	15	*ANKDD1A*	65227571	rs1380844	0.9881	64242007	G	T	0.51	4.74E-06	2.78E-02	8.63E-02
rs12908891	Brain_Cerebellar_Hemisphere	15	*ANKDD1A*	65227571	rs12916806	0.9881	64242770	C	A	0.51	4.64E-06	2.78E-02	8.63E-02
rs12908891	Brain_Cerebellar_Hemisphere	15	*ANKDD1A*	65227571	rs1304365	0.9881	64245946	A	G	0.51	4.67E-06	2.78E-02	8.64E-02
rs12908891	Brain_Cerebellar_Hemisphere	15	*SNX22*	64446798	rs115415409	0.8420	64249559	T	C	0.45	1.45E-05	9.84E-03	2.96E-02
rs12908891	Brain_Cerebellar_Hemisphere	15	*ANKDD1A*	65227571	rs115415409	0.8420	64249559	T	C	0.45	1.45E-05	3.78E-02	6.46E-02
rs12908891	Brain_Cerebellar_Hemisphere	15	*ANKDD1A*	65227571	rs145159458	0.9119	64252880	T	C	0.49	1.21E-05	4.58E-02	1.00E-01
rs12908891	Brain_Cerebellar_Hemisphere	15	*ANKDD1A*	65227571	rs4776692	0.9881	64255083	G	A	0.50	1.06E-05	2.78E-02	9.56E-02
rs12908891	Brain_Cerebellar_Hemisphere	15	*SNX22*	64446798	rs28822416	0.8420	64257845	C	T	0.45	1.54E-05	1.33E-02	3.49E-02
rs12908891	Brain_Cerebellar_Hemisphere	15	*SNX22*	64446798	rs8035776	0.8420	64258018	G	A	0.45	1.54E-05	1.33E-02	3.49E-02
rs12908891	Brain_Cerebellar_Hemisphere	15	*SNX22*	64446798	rs6494456	0.8381	64258260	C	T	0.45	1.54E-05	1.33E-02	3.49E-02
rs12908891	Brain_Cerebellar_Hemisphere	15	*PLEKHO2*	65147147	rs4776695	0.8350	64259115	T	C	0.46	9.12E-06	3.37E-02	5.72E-02
rs12908891	Brain_Cerebellum	15	*RP11-244F12.3*	63339909	rs1460544	0.8736	64201766	T	C	0.51	9.06E-04	2.73E-02	1.45E-01
rs12908891	Brain_Cerebellum	15	*CA12*	63643968	rs1460544	0.8736	64201766	T	C	0.51	9.06E-04	1.29E-02	1.26E-01
rs12908891	Brain_Cerebellum	15	*DAPK2*	64281733	rs1460544	0.8736	64201766	T	C	0.51	9.06E-04	6.63E-04	9.15E-02
rs12908891	Brain_Cerebellum	15	*RP11-244F12.3*	63339909	rs1037846	0.8696	64204870	G	A	0.51	6.33E-04	2.68E-02	5.74E-02
rs12908891	Brain_Cerebellum	15	*CA12*	63643968	rs1037846	0.8696	64204870	G	A	0.51	6.33E-04	2.86E-02	5.95E-02
rs12908891	Brain_Cerebellum	15	*DAPK2*	64281733	rs1037846	0.8696	64204870	G	A	0.51	6.33E-04	1.14E-03	1.45E-02
rs12908891	Brain_Cerebellum	15	*RP11-244F12.3*	63339909	rs11071773	0.8883	64206765	A	G	0.51	8.95E-04	2.54E-02	1.15E-01
rs12908891	Brain_Cerebellum	15	*CA12*	63643968	rs11071773	0.8883	64206765	A	G	0.51	8.95E-04	4.66E-02	1.38E-01
rs12908891	Brain_Cerebellum	15	*DAPK2*	64281733	rs11071773	0.8883	64206765	A	G	0.51	8.95E-04	9.40E-04	6.54E-02
rs12908891	Brain_Cerebellum	15	*RP11-244F12.3*	63339909	rs12909081	0.8809	64208260	A	C	0.51	1.00E-03	2.42E-02	1.18E-01
rs12908891	Brain_Cerebellum	15	*CA12*	63643968	rs12909081	0.8809	64208260	A	C	0.51	1.00E-03	2.75E-02	1.22E-01
rs12908891	Brain_Cerebellum	15	*DAPK2*	64281733	rs12909081	0.8809	64208260	A	C	0.51	1.00E-03	5.59E-04	6.58E-02
rs12908891	Brain_Cerebellum	15	*RP11-244F12.3*	63339909	rs749468	0.9493	64210279	T	C	0.53	2.79E-04	3.01E-02	6.07E-02
rs12908891	Brain_Cerebellum	15	*CA12*	63643968	rs749468	0.9493	64210279	T	C	0.53	2.79E-04	3.40E-02	6.52E-02
rs12908891	Brain_Cerebellum	15	*FBXL22*	63892089	rs749468	0.9493	64210279	T	C	0.53	2.79E-04	4.10E-02	7.30E-02
rs12908891	Brain_Cerebellum	15	*DAPK2*	64281733	rs749468	0.9493	64210279	T	C	0.53	2.79E-04	3.59E-04	9.85E-03
rs12908891	Brain_Cerebellum	15	*RP11-244F12.3*	63339909	rs2414844	0.8846	64210675	A	G	0.51	9.01E-04	2.42E-02	1.15E-01
rs12908891	Brain_Cerebellum	15	*CA12*	63643968	rs2414844	0.8846	64210675	A	G	0.51	9.01E-04	2.75E-02	1.19E-01
rs12908891	Brain_Cerebellum	15	*DAPK2*	64281733	rs2414844	0.8846	64210675	A	G	0.51	9.01E-04	5.59E-04	6.28E-02
rs12908891	Brain_Cerebellum	15	*RP11-244F12.3*	63339909	rs12592060	0.8846	64210780	T	C	0.51	9.01E-04	2.42E-02	1.15E-01
rs12908891	Brain_Cerebellum	15	*CA12*	63643968	rs12592060	0.8846	64210780	T	C	0.51	9.01E-04	2.75E-02	1.19E-01
rs12908891	Brain_Cerebellum	15	*DAPK2*	64281733	rs12592060	0.8846	64210780	T	C	0.51	9.01E-04	5.59E-04	6.28E-02
rs12908891	Brain_Cerebellum	15	*RP11-244F12.3*	63339909	rs1471282	0.8846	64212275	G	T	0.51	1.07E-03	2.42E-02	1.21E-01
rs12908891	Brain_Cerebellum	15	*CA12*	63643968	rs1471282	0.8846	64212275	G	T	0.51	1.07E-03	2.75E-02	1.25E-01
rs12908891	Brain_Cerebellum	15	*DAPK2*	64281733	rs1471282	0.8846	64212275	G	T	0.51	1.07E-03	5.59E-04	6.92E-02

rs12908891	Brain_Cerebellum	15	*RP11-244F12.3*	63339909	rs11635779	0.8808	64213826	T	C	0.50	6.34E-05	3.41E-02	7.51E-02
rs12908891	Brain_Cerebellum	15	*CA12*	63643968	rs11635779	0.8808	64213826	T	C	0.50	6.34E-05	4.03E-02	8.20E-02
rs12908891	Brain_Cerebellum	15	*DAPK2*	64281733	rs11635779	0.8808	64213826	T	C	0.50	6.34E-05	5.71E-04	1.75E-02
rs12908891	Brain_Cerebellum	15	*RP11-244F12.3*	63339909	rs12904374	0.8808	64214670	T	A	0.51	8.63E-04	3.50E-02	6.96E-02
rs12908891	Brain_Cerebellum	15	*DAPK2*	64281733	rs12904374	0.8808	64214670	T	A	0.51	8.63E-04	9.25E-04	1.53E-02
rs12908891	Brain_Cerebellum	15	*RP11-244F12.3*	63339909	rs920762	0.9379	64214954	C	G	0.47	1.24E-05	3.72E-02	5.73E-02
rs12908891	Brain_Cerebellum	15	*LACTB*	63424129	rs920762	0.9379	64214954	C	G	0.47	1.24E-05	4.98E-02	7.08E-02
rs12908891	Brain_Cerebellum	15	*DAPK2*	64281733	rs920762	0.9379	64214954	C	G	0.47	1.24E-05	1.31E-03	8.25E-03
rs12908891	Brain_Cerebellum	15	*RP11-244F12.3*	63339909	rs11853632	0.8921	64217845	C	G	0.50	4.21E-05	1.92E-02	5.36E-02
rs12908891	Brain_Cerebellum	15	*CA12*	63643968	rs11853632	0.8921	64217845	C	G	0.50	4.21E-05	1.99E-02	5.46E-02
rs12908891	Brain_Cerebellum	15	*DAPK2*	64281733	rs11853632	0.8921	64217845	C	G	0.50	4.21E-05	6.27E-03	3.30E-02
rs12908891	Brain_Cerebellum	15	*RP11-244F12.3*	63339909	rs12907405	0.8884	64220046	T	C	0.50	4.35E-05	2.32E-02	5.92E-02
rs12908891	Brain_Cerebellum	15	*CA12*	63643968	rs12907405	0.8884	64220046	T	C	0.50	4.35E-05	2.58E-02	6.24E-02
rs12908891	Brain_Cerebellum	15	*DAPK2*	64281733	rs12907405	0.8884	64220046	T	C	0.50	4.35E-05	5.70E-04	1.56E-02
rs12908891	Brain_Cerebellum	15	*RP11-244F12.3*	63339909	rs11071775	0.8808	64220995	A	G	0.50	4.81E-05	3.69E-02	7.55E-02
rs12908891	Brain_Cerebellum	15	*CA12*	63643968	rs11071775	0.8808	64220995	A	G	0.50	4.81E-05	1.94E-02	5.42E-02
rs12908891	Brain_Cerebellum	15	*DAPK2*	64281733	rs11071775	0.8808	64220995	A	G	0.50	4.81E-05	1.10E-03	1.87E-02
rs12908891	Brain_Cerebellum	15	*RP11-244F12.3*	63339909	rs11071776	0.8808	64221010	T	A	0.50	5.49E-05	3.69E-02	7.65E-02
rs12908891	Brain_Cerebellum	15	*CA12*	63643968	rs11071776	0.8808	64221010	T	A	0.50	5.49E-05	1.94E-02	5.52E-02
rs12908891	Brain_Cerebellum	15	*DAPK2*	64281733	rs11071776	0.8808	64221010	T	A	0.50	5.49E-05	1.10E-03	1.94E-02
rs12908891	Brain_Cerebellum	15	*CA12*	63643968	rs11637858	0.8959	64224107	C	T	0.49	4.60E-05	2.10E-02	5.60E-02
rs12908891	Brain_Cerebellum	15	*DAPK2*	64281733	rs11637858	0.8959	64224107	C	T	0.49	4.60E-05	1.29E-03	1.93E-02
rs12908891	Brain_Cerebellum	15	*LACTB*	63424129	rs12916395	0.9921	64225563	T	C	0.51	1.86E-06	3.84E-02	5.61E-02
rs12908891	Brain_Cerebellum	15	*DAPK2*	64281733	rs12916395	0.9921	64225563	T	C	0.51	1.86E-06	1.57E-03	7.72E-03
rs12908891	Brain_Cerebellum	15	*LACTB*	63424129	rs11630587	0.9921	64225908	C	T	0.51	1.77E-06	2.78E-02	4.43E-02
rs12908891	Brain_Cerebellum	15	*CA12*	63643968	rs11630587	0.9921	64225908	C	T	0.51	1.77E-06	3.66E-02	5.41E-02
rs12908891	Brain_Cerebellum	15	*DAPK2*	64281733	rs11630587	0.9921	64225908	C	T	0.51	1.77E-06	2.70E-03	1.03E-02
rs12908891	Brain_Cerebellum	15	*LACTB*	63424129	rs4776677	0.9921	64227044	C	T	0.51	1.54E-06	3.45E-02	5.16E-02
rs12908891	Brain_Cerebellum	15	*DAPK2*	64281733	rs4776677	0.9921	64227044	C	T	0.51	1.54E-06	2.54E-03	9.84E-03
rs12908891	Brain_Cerebellum	15	*LACTB*	63424129	rs11071777	0.8557	64229285	C	G	0.55	9.20E-06	5.09E-03	1.65E-02
rs12908891	Brain_Cerebellum	15	*USP3-AS1*	63864970	rs11071777	0.8557	64229285	C	G	0.55	9.20E-06	4.89E-02	7.00E-02
rs12908891	Brain_Cerebellum	15	*DAPK2*	64281733	rs11071777	0.8557	64229285	C	G	0.55	9.20E-06	8.60E-04	6.82E-03
rs12908891	Brain_Cerebellum	15	*RP11-244F12.3*	63339909	rs1563886	0.9272	64230852	G	C	0.49	8.06E-06	4.91E-02	9.15E-02
rs12908891	Brain_Cerebellum	15	*CA12*	63643968	rs1563886	0.9272	64230852	G	C	0.49	8.06E-06	1.90E-02	5.63E-02
rs12908891	Brain_Cerebellum	15	*DAPK2*	64281733	rs1563886	0.9272	64230852	G	C	0.49	8.06E-06	1.01E-03	2.02E-02
rs12908891	Brain_Cerebellum	15	*LACTB*	63424129	rs10744961	0.9960	64230930	A	G	0.51	1.86E-06	3.98E-02	5.80E-02
rs12908891	Brain_Cerebellum	15	*CA12*	63643968	rs10744961	0.9960	64230930	A	G	0.51	1.86E-06	3.53E-02	5.31E-02
rs12908891	Brain_Cerebellum	15	*DAPK2*	64281733	rs10744961	0.9960	64230930	A	G	0.51	1.86E-06	2.08E-03	9.15E-03
rs12908891	Brain_Cerebellum	15	*LACTB*	63424129	rs6494454	0.8031	64233563	A	C	0.56	2.52E-05	3.21E-02	5.59E-02
rs12908891	Brain_Cerebellum	15	*DAPK2*	64281733	rs6494454	0.8031	64233563	A	C	0.56	2.52E-05	1.65E-03	1.15E-02

rs12908891	Brain_Cerebellum	15	*LACTB*	63424129	rs894660	0.8031	64234334	C	T	0.56	2.52E-05	3.21E-02	5.59E-02
rs12908891	Brain_Cerebellum	15	*DAPK2*	64281733	rs894660	0.8031	64234334	C	T	0.56	2.52E-05	1.65E-03	1.15E-02
rs12908891	Brain_Cerebellum	15	*LACTB*	63424129	rs4776266	0.8031	64236175	G	T	0.56	2.23E-05	2.82E-02	5.12E-02
rs12908891	Brain_Cerebellum	15	*DAPK2*	64281733	rs4776266	0.8031	64236175	G	T	0.56	2.23E-05	1.50E-03	1.10E-02
rs12908891	Brain_Cerebellum	15	*CA12*	63643968	rs4776268	1.0000	64236398	A	G	0.51	6.19E-06	2.83E-02	8.43E-02
rs12908891	Brain_Cerebellum	15	*DAPK2*	64281733	rs4776268	1.0000	64236398	A	G	0.51	6.19E-06	8.11E-04	3.18E-02
rs12908891	Brain_Cerebellum	15	*RP11-244F12.3*	63339909	rs12908891	1.0000	64236441	A	G	0.52	1.39E-06	4.94E-02	6.77E-02
rs12908891	Brain_Cerebellum	15	*LACTB*	63424129	rs12908891	1.0000	64236441	A	G	0.52	1.39E-06	2.10E-02	3.64E-02
rs12908891	Brain_Cerebellum	15	*CA12*	63643968	rs12908891	1.0000	64236441	A	G	0.52	1.39E-06	2.44E-02	4.04E-02
rs12908891	Brain_Cerebellum	15	*USP3-AS1*	63864970	rs12908891	1.0000	64236441	A	G	0.52	1.39E-06	4.85E-02	6.69E-02
rs12908891	Brain_Cerebellum	15	*DAPK2*	64281733	rs12908891	1.0000	64236441	A	G	0.52	1.39E-06	1.95E-03	8.63E-03
rs12908891	Brain_Cerebellum	15	*RP11-244F12.3*	63339909	rs11071779	0.9881	64239044	G	A	0.51	1.57E-06	4.92E-02	6.83E-02
rs12908891	Brain_Cerebellum	15	*CA12*	63643968	rs11071779	0.9881	64239044	G	A	0.51	1.57E-06	3.33E-02	5.12E-02
rs12908891	Brain_Cerebellum	15	*DAPK2*	64281733	rs11071779	0.9881	64239044	G	A	0.51	1.57E-06	3.09E-03	1.15E-02
rs12908891	Brain_Cerebellum	15	*RP11-244F12.3*	63339909	rs11071780	0.9842	64241691	A	G	0.51	4.74E-06	4.06E-02	1.01E-01
rs12908891	Brain_Cerebellum	15	*LACTB*	63424129	rs11071780	0.9842	64241691	A	G	0.51	4.74E-06	4.74E-02	1.08E-01
rs12908891	Brain_Cerebellum	15	*CA12*	63643968	rs11071780	0.9842	64241691	A	G	0.51	4.74E-06	2.04E-02	7.67E-02
rs12908891	Brain_Cerebellum	15	*DAPK2*	64281733	rs11071780	0.9842	64241691	A	G	0.51	4.74E-06	1.06E-03	3.57E-02
rs12908891	Brain_Cerebellum	15	*RP11-244F12.3*	63339909	rs11854537	0.9842	64241733	A	G	0.51	4.74E-06	4.06E-02	1.01E-01
rs12908891	Brain_Cerebellum	15	*LACTB*	63424129	rs11854537	0.9842	64241733	A	G	0.51	4.74E-06	4.74E-02	1.08E-01
rs12908891	Brain_Cerebellum	15	*CA12*	63643968	rs11854537	0.9842	64241733	A	G	0.51	4.74E-06	2.04E-02	7.67E-02
rs12908891	Brain_Cerebellum	15	*DAPK2*	64281733	rs11854537	0.9842	64241733	A	G	0.51	4.74E-06	1.06E-03	3.57E-02
rs12908891	Brain_Cerebellum	15	*RP11-244F12.3*	63339909	rs1380844	0.9881	64242007	G	T	0.51	4.74E-06	4.06E-02	1.01E-01
rs12908891	Brain_Cerebellum	15	*LACTB*	63424129	rs1380844	0.9881	64242007	G	T	0.51	4.74E-06	4.74E-02	1.08E-01
rs12908891	Brain_Cerebellum	15	*CA12*	63643968	rs1380844	0.9881	64242007	G	T	0.51	4.74E-06	2.04E-02	7.67E-02
rs12908891	Brain_Cerebellum	15	*DAPK2*	64281733	rs1380844	0.9881	64242007	G	T	0.51	4.74E-06	1.06E-03	3.57E-02
rs12908891	Brain_Cerebellum	15	*RP11-244F12.3*	63339909	rs12916806	0.9881	64242770	C	A	0.51	4.64E-06	4.06E-02	1.01E-01
rs12908891	Brain_Cerebellum	15	*LACTB*	63424129	rs12916806	0.9881	64242770	C	A	0.51	4.64E-06	4.74E-02	1.08E-01
rs12908891	Brain_Cerebellum	15	*CA12*	63643968	rs12916806	0.9881	64242770	C	A	0.51	4.64E-06	2.04E-02	7.67E-02
rs12908891	Brain_Cerebellum	15	*DAPK2*	64281733	rs12916806	0.9881	64242770	C	A	0.51	4.64E-06	1.06E-03	3.57E-02
rs12908891	Brain_Cerebellum	15	*RP11-244F12.3*	63339909	rs11071781	0.9195	64243933	G	A	0.49	4.75E-06	4.57E-02	8.93E-02
rs12908891	Brain_Cerebellum	15	*CA12*	63643968	rs11071781	0.9195	64243933	G	A	0.49	4.75E-06	2.46E-02	6.50E-02
rs12908891	Brain_Cerebellum	15	*DAPK2*	64281733	rs11071781	0.9195	64243933	G	A	0.49	4.75E-06	1.62E-03	2.41E-02
rs12908891	Brain_Cerebellum	15	*RP11-244F12.3*	63339909	rs1304365	0.9881	64245946	A	G	0.51	4.67E-06	4.38E-02	1.05E-01
rs12908891	Brain_Cerebellum	15	*LACTB*	63424129	rs1304365	0.9881	64245946	A	G	0.51	4.67E-06	4.15E-02	1.02E-01
rs12908891	Brain_Cerebellum	15	*CA12*	63643968	rs1304365	0.9881	64245946	A	G	0.51	4.67E-06	3.27E-02	9.22E-02
rs12908891	Brain_Cerebellum	15	*DAPK2*	64281733	rs1304365	0.9881	64245946	A	G	0.51	4.67E-06	1.03E-03	3.55E-02
rs12908891	Brain_Cerebellum	15	*RP11-244F12.3*	63339909	rs115415409	0.8420	64249559	T	C	0.45	1.45E-05	5.18E-03	2.15E-02
rs12908891	Brain_Cerebellum	15	*LACTB*	63424129	rs115415409	0.8420	64249559	T	C	0.45	1.45E-05	4.43E-02	7.18E-02
rs12908891	Brain_Cerebellum	15	*USP3-AS1*	63864970	rs115415409	0.8420	64249559	T	C	0.45	1.45E-05	4.04E-02	6.75E-02

rs12908891	Brain_Cerebellum	15	*DAPK2*	64281733	rs115415409	0.8420	64249559	T	C	0.45	1.45E-05	5.74E-03	2.26E-02
rs12908891	Brain_Cerebellum	15	*RP11-244F12.3*	63339909	rs145159458	0.9119	64252880	T	C	0.49	1.21E-05	3.52E-02	8.85E-02
rs12908891	Brain_Cerebellum	15	*CA12*	63643968	rs145159458	0.9119	64252880	T	C	0.49	1.21E-05	2.22E-02	7.27E-02
rs12908891	Brain_Cerebellum	15	*DAPK2*	64281733	rs145159458	0.9119	64252880	T	C	0.49	1.21E-05	1.28E-03	3.13E-02
rs12908891	Brain_Cerebellum	15	*RP11-244F12.3*	63339909	rs4776692	0.9881	64255083	G	A	0.50	1.06E-05	4.06E-02	1.10E-01
rs12908891	Brain_Cerebellum	15	*LACTB*	63424129	rs4776692	0.9881	64255083	G	A	0.50	1.06E-05	4.74E-02	1.17E-01
rs12908891	Brain_Cerebellum	15	*CA12*	63643968	rs4776692	0.9881	64255083	G	A	0.50	1.06E-05	2.04E-02	8.60E-02
rs12908891	Brain_Cerebellum	15	*DAPK2*	64281733	rs4776692	0.9881	64255083	G	A	0.50	1.06E-05	1.06E-03	4.41E-02
rs12908891	Brain_Cerebellum	15	*RP11-244F12.3*	63339909	rs28822416	0.8420	64257845	C	T	0.45	1.54E-05	6.08E-03	2.34E-02
rs12908891	Brain_Cerebellum	15	*USP3-AS1*	63864970	rs28822416	0.8420	64257845	C	T	0.45	1.54E-05	3.33E-02	5.99E-02
rs12908891	Brain_Cerebellum	15	*DAPK2*	64281733	rs28822416	0.8420	64257845	C	T	0.45	1.54E-05	1.84E-03	1.38E-02
rs12908891	Brain_Cerebellum	15	*RP11-244F12.3*	63339909	rs8035776	0.8420	64258018	G	A	0.45	1.54E-05	6.08E-03	2.34E-02
rs12908891	Brain_Cerebellum	15	*USP3-AS1*	63864970	rs8035776	0.8420	64258018	G	A	0.45	1.54E-05	3.33E-02	5.99E-02
rs12908891	Brain_Cerebellum	15	*DAPK2*	64281733	rs8035776	0.8420	64258018	G	A	0.45	1.54E-05	1.84E-03	1.38E-02
rs12908891	Brain_Cerebellum	15	*RP11-244F12.3*	63339909	rs6494456	0.8381	64258260	C	T	0.45	1.54E-05	6.08E-03	2.34E-02
rs12908891	Brain_Cerebellum	15	*USP3-AS1*	63864970	rs6494456	0.8381	64258260	C	T	0.45	1.54E-05	3.33E-02	5.99E-02
rs12908891	Brain_Cerebellum	15	*DAPK2*	64281733	rs6494456	0.8381	64258260	C	T	0.45	1.54E-05	1.84E-03	1.38E-02
rs12908891	Brain_Cerebellum	15	*DAPK2*	64281733	rs4776695	0.8350	64259115	T	C	0.46	9.12E-06	2.27E-03	1.30E-02
rs12908891	Brain_Cortex	15	*PLEKHO2*	65147147	rs1037846	0.8696	64204870	G	A	0.51	6.33E-04	3.60E-02	6.81E-02
rs12908891	Brain_Cortex	15	*PLEKHO2*	65147147	rs749468	0.9493	64210279	T	C	0.53	2.79E-04	4.85E-02	8.10E-02
rs12908891	Brain_Cortex	15	*PLEKHO2*	65147147	rs920762	0.9379	64214954	C	G	0.47	1.24E-05	3.99E-02	6.02E-02
rs12908891	Brain_Cortex	15	*LACTB*	63424129	rs11071775	0.8808	64220995	A	G	0.50	4.81E-05	4.96E-02	8.92E-02
rs12908891	Brain_Cortex	15	*LACTB*	63424129	rs11071776	0.8808	64221010	T	A	0.50	5.49E-05	4.96E-02	9.02E-02
rs12908891	Brain_Cortex	15	*RBPMS2*	65049938	rs11071777	0.8557	64229285	C	G	0.55	9.20E-06	3.04E-02	4.98E-02
rs12908891	Brain_Cortex	15	*ANKDD1A*	65227571	rs6494454	0.8031	64233563	A	C	0.56	2.52E-05	4.45E-02	6.94E-02
rs12908891	Brain_Cortex	15	*ANKDD1A*	65227571	rs894660	0.8031	64234334	C	T	0.56	2.52E-05	4.45E-02	6.94E-02
rs12908891	Brain_Cortex	15	*LACTB*	63424129	rs28822416	0.8420	64257845	C	T	0.45	1.54E-05	2.89E-02	5.49E-02
rs12908891	Brain_Cortex	15	*LACTB*	63424129	rs8035776	0.8420	64258018	G	A	0.45	1.54E-05	3.60E-02	6.29E-02
rs12908891	Brain_Cortex	15	*LACTB*	63424129	rs6494456	0.8381	64258260	C	T	0.45	1.54E-05	2.89E-02	5.49E-02
rs12908891	Brain_Cortex	15	*PLEKHO2*	65147147	rs4776695	0.8350	64259115	T	C	0.46	9.12E-06	4.97E-02	7.45E-02
rs12908891	Brain_Frontal_Cortex_BA9	15	*RP11-244F12.3*	63339909	rs1460544	0.8736	64201766	T	C	0.51	9.06E-04	4.00E-02	1.58E-01
rs12908891	Brain_Frontal_Cortex_BA9	15	*AC100830.3*	65002624	rs1037846	0.8696	64204870	G	A	0.51	6.33E-04	2.81E-02	5.89E-02
rs12908891	Brain_Frontal_Cortex_BA9	15	*PLEKHO2*	65147147	rs1037846	0.8696	64204870	G	A	0.51	6.33E-04	3.01E-02	6.13E-02
rs12908891	Brain_Frontal_Cortex_BA9	15	*RP11-244F12.3*	63339909	rs11071773	0.8883	64206765	A	G	0.51	8.95E-04	4.33E-02	1.35E-01
rs12908891	Brain_Frontal_Cortex_BA9	15	*AC100830.3*	65002624	rs11071773	0.8883	64206765	A	G	0.51	8.95E-04	4.31E-02	1.35E-01
rs12908891	Brain_Frontal_Cortex_BA9	15	*RP11-244F12.3*	63339909	rs12909081	0.8809	64208260	A	C	0.51	1.00E-03	3.91E-02	1.34E-01
rs12908891	Brain_Frontal_Cortex_BA9	15	*AC100830.3*	65002624	rs749468	0.9493	64210279	T	C	0.53	2.79E-04	1.94E-02	4.75E-02
rs12908891	Brain_Frontal_Cortex_BA9	15	*RP11-244F12.3*	63339909	rs2414844	0.8846	64210675	A	G	0.51	9.01E-04	3.91E-02	1.32E-01
rs12908891	Brain_Frontal_Cortex_BA9	15	*RP11-244F12.3*	63339909	rs12592060	0.8846	64210780	T	C	0.51	9.01E-04	3.91E-02	1.32E-01
rs12908891	Brain_Frontal_Cortex_BA9	15	*RP11-244F12.3*	63339909	rs1471282	0.8846	64212275	G	T	0.51	1.07E-03	3.91E-02	1.38E-01

rs12908891	Brain_Frontal_Cortex_BA9	15	*RP11-244F12.3*	63339909	rs11635779	0.8808	64213826	T	C	0.50	6.34E-05	3.66E-02	7.79E-02
rs12908891	Brain_Frontal_Cortex_BA9	15	*AC100830.3*	65002624	rs11635779	0.8808	64213826	T	C	0.50	6.34E-05	4.47E-02	8.68E-02
rs12908891	Brain_Frontal_Cortex_BA9	15	*RP11-244F12.3*	63339909	rs12904374	0.8808	64214670	T	A	0.51	8.63E-04	4.02E-02	7.54E-02
rs12908891	Brain_Frontal_Cortex_BA9	15	*AC100830.3*	65002624	rs12904374	0.8808	64214670	T	A	0.51	8.63E-04	3.81E-02	7.31E-02
rs12908891	Brain_Frontal_Cortex_BA9	15	*AC100830.3*	65002624	rs920762	0.9379	64214954	C	G	0.47	1.24E-05	1.17E-02	2.67E-02
rs12908891	Brain_Frontal_Cortex_BA9	15	*PLEKHO2*	65147147	rs920762	0.9379	64214954	C	G	0.47	1.24E-05	4.71E-02	6.80E-02
rs12908891	Brain_Frontal_Cortex_BA9	15	*RP11-244F12.3*	63339909	rs11853632	0.8921	64217845	C	G	0.50	4.21E-05	3.91E-02	7.76E-02
rs12908891	Brain_Frontal_Cortex_BA9	15	*RP11-244F12.3*	63339909	rs12907405	0.8884	64220046	T	C	0.50	4.35E-05	3.59E-02	7.43E-02
rs12908891	Brain_Frontal_Cortex_BA9	15	*AC100830.3*	65002624	rs12907405	0.8884	64220046	T	C	0.50	4.35E-05	4.74E-02	8.68E-02
rs12908891	Brain_Frontal_Cortex_BA9	15	*RP11-244F12.3*	63339909	rs11071775	0.8808	64220995	A	G	0.50	4.81E-05	3.59E-02	7.44E-02
rs12908891	Brain_Frontal_Cortex_BA9	15	*AC100830.3*	65002624	rs11071775	0.8808	64220995	A	G	0.50	4.81E-05	4.74E-02	8.69E-02
rs12908891	Brain_Frontal_Cortex_BA9	15	*RP11-244F12.3*	63339909	rs11071776	0.8808	64221010	T	A	0.50	5.49E-05	3.59E-02	7.54E-02
rs12908891	Brain_Frontal_Cortex_BA9	15	*AC100830.3*	65002624	rs11071776	0.8808	64221010	T	A	0.50	5.49E-05	4.74E-02	8.79E-02
rs12908891	Brain_Frontal_Cortex_BA9	15	*RP11-244F12.3*	63339909	rs11637858	0.8959	64224107	C	T	0.49	4.60E-05	1.85E-02	5.26E-02
rs12908891	Brain_Frontal_Cortex_BA9	15	*AC100830.3*	65002624	rs12916395	0.9921	64225563	T	C	0.51	1.86E-06	6.46E-03	1.70E-02
rs12908891	Brain_Frontal_Cortex_BA9	15	*PLEKHO2*	65147147	rs12916395	0.9921	64225563	T	C	0.51	1.86E-06	3.59E-02	5.34E-02
rs12908891	Brain_Frontal_Cortex_BA9	15	*AC100830.3*	65002624	rs11630587	0.9921	64225908	C	T	0.51	1.77E-06	1.52E-02	2.93E-02
rs12908891	Brain_Frontal_Cortex_BA9	15	*AC100830.3*	65002624	rs4776677	0.9921	64227044	C	T	0.51	1.54E-06	6.10E-03	1.63E-02
rs12908891	Brain_Frontal_Cortex_BA9	15	*PLEKHO2*	65147147	rs4776677	0.9921	64227044	C	T	0.51	1.54E-06	3.62E-02	5.35E-02
rs12908891	Brain_Frontal_Cortex_BA9	15	*AC100830.3*	65002624	rs11071777	0.8557	64229285	C	G	0.55	9.20E-06	2.75E-02	4.65E-02
rs12908891	Brain_Frontal_Cortex_BA9	15	*RP11-244F12.3*	63339909	rs1563886	0.9272	64230852	G	C	0.49	8.06E-06	4.55E-02	8.76E-02
rs12908891	Brain_Frontal_Cortex_BA9	15	*AC100830.3*	65002624	rs1563886	0.9272	64230852	G	C	0.49	8.06E-06	4.61E-02	8.83E-02
rs12908891	Brain_Frontal_Cortex_BA9	15	*AC100830.3*	65002624	rs10744961	0.9960	64230930	A	G	0.51	1.86E-06	1.03E-02	2.30E-02
rs12908891	Brain_Frontal_Cortex_BA9	15	*PLEKHO2*	65147147	rs10744961	0.9960	64230930	A	G	0.51	1.86E-06	4.77E-02	6.64E-02
rs12908891	Brain_Frontal_Cortex_BA9	15	*RP11-244F12.3*	63339909	rs6494454	0.8031	64233563	A	C	0.56	2.52E-05	4.26E-02	6.74E-02
rs12908891	Brain_Frontal_Cortex_BA9	15	*AC100830.3*	65002624	rs6494454	0.8031	64233563	A	C	0.56	2.52E-05	2.29E-02	4.51E-02
rs12908891	Brain_Frontal_Cortex_BA9	15	*PLEKHO2*	65147147	rs6494454	0.8031	64233563	A	C	0.56	2.52E-05	3.66E-02	6.09E-02
rs12908891	Brain_Frontal_Cortex_BA9	15	*RP11-244F12.3*	63339909	rs894660	0.8031	64234334	C	T	0.56	2.52E-05	4.26E-02	6.74E-02
rs12908891	Brain_Frontal_Cortex_BA9	15	*AC100830.3*	65002624	rs894660	0.8031	64234334	C	T	0.56	2.52E-05	2.29E-02	4.51E-02
rs12908891	Brain_Frontal_Cortex_BA9	15	*PLEKHO2*	65147147	rs894660	0.8031	64234334	C	T	0.56	2.52E-05	3.66E-02	6.09E-02
rs12908891	Brain_Frontal_Cortex_BA9	15	*AC100830.3*	65002624	rs4776266	0.8031	64236175	G	T	0.56	2.23E-05	1.78E-02	3.85E-02
rs12908891	Brain_Frontal_Cortex_BA9	15	*PLEKHO2*	65147147	rs4776266	0.8031	64236175	G	T	0.56	2.23E-05	2.36E-02	4.57E-02
rs12908891	Brain_Frontal_Cortex_BA9	15	*RP11-244F12.3*	63339909	rs4776268	1.0000	64236398	A	G	0.51	6.19E-06	3.51E-02	9.23E-02
rs12908891	Brain_Frontal_Cortex_BA9	15	*AC100830.3*	65002624	rs4776268	1.0000	64236398	A	G	0.51	6.19E-06	3.67E-02	9.41E-02
rs12908891	Brain_Frontal_Cortex_BA9	15	*AC100830.3*	65002624	rs12908891	1.0000	64236441	A	G	0.52	1.39E-06	1.67E-02	3.12E-02
rs12908891	Brain_Frontal_Cortex_BA9	15	*PLEKHO2*	65147147	rs12908891	1.0000	64236441	A	G	0.52	1.39E-06	4.73E-02	6.56E-02
rs12908891	Brain_Frontal_Cortex_BA9	15	*AC100830.3*	65002624	rs11071779	0.9881	64239044	G	A	0.51	1.57E-06	1.95E-02	3.52E-02
rs12908891	Brain_Frontal_Cortex_BA9	15	*RP11-244F12.3*	63339909	rs11071780	0.9842	64241691	A	G	0.51	4.74E-06	4.10E-02	1.01E-01
rs12908891	Brain_Frontal_Cortex_BA9	15	*AC100830.3*	65002624	rs11071780	0.9842	64241691	A	G	0.51	4.74E-06	3.78E-02	9.80E-02
rs12908891	Brain_Frontal_Cortex_BA9	15	*RP11-244F12.3*	63339909	rs11854537	0.9842	64241733	A	G	0.51	4.74E-06	4.10E-02	1.01E-01

rs12908891	Brain_Frontal_Cortex_BA9	15	*AC100830.3*	65002624	rs11854537	0.9842	64241733	A	G	0.51	4.74E-06	3.78E-02	9.80E-02
rs12908891	Brain_Frontal_Cortex_BA9	15	*RP11-244F12.3*	63339909	rs1380844	0.9881	64242007	G	T	0.51	4.74E-06	4.10E-02	1.01E-01
rs12908891	Brain_Frontal_Cortex_BA9	15	*AC100830.3*	65002624	rs1380844	0.9881	64242007	G	T	0.51	4.74E-06	3.78E-02	9.80E-02
rs12908891	Brain_Frontal_Cortex_BA9	15	*RP11-244F12.3*	63339909	rs12916806	0.9881	64242770	C	A	0.51	4.64E-06	4.10E-02	1.01E-01
rs12908891	Brain_Frontal_Cortex_BA9	15	*AC100830.3*	65002624	rs12916806	0.9881	64242770	C	A	0.51	4.64E-06	3.78E-02	9.79E-02
rs12908891	Brain_Frontal_Cortex_BA9	15	*RP11-244F12.3*	63339909	rs11071781	0.9195	64243933	G	A	0.49	4.75E-06	1.85E-02	5.70E-02
rs12908891	Brain_Frontal_Cortex_BA9	15	*RP11-244F12.3*	63339909	rs1304365	0.9881	64245946	A	G	0.51	4.67E-06	3.51E-02	9.50E-02
rs12908891	Brain_Frontal_Cortex_BA9	15	*AC100830.3*	65002624	rs1304365	0.9881	64245946	A	G	0.51	4.67E-06	3.67E-02	9.67E-02
rs12908891	Brain_Frontal_Cortex_BA9	15	*RP11-244F12.3*	63339909	s145159458	0.9119	64252880	T	C	0.49	1.21E-05	4.55E-02	9.98E-02
rs12908891	Brain_Frontal_Cortex_BA9	15	*AC100830.3*	65002624	s145159458	0.9119	64252880	T	C	0.49	1.21E-05	4.61E-02	1.00E-01
rs12908891	Brain_Frontal_Cortex_BA9	15	*RP11-244F12.3*	63339909	rs4776692	0.9881	64255083	G	A	0.50	1.06E-05	4.10E-02	1.11E-01
rs12908891	Brain_Frontal_Cortex_BA9	15	*AC100830.3*	65002624	rs4776692	0.9881	64255083	G	A	0.50	1.06E-05	3.78E-02	1.07E-01
rs12908891	Brain_Frontal_Cortex_BA9	15	*RP11-244F12.3*	63339909	rs28822416	0.8420	64257845	C	T	0.45	1.54E-05	4.82E-02	7.62E-02
rs12908891	Brain_Frontal_Cortex_BA9	15	*AC100830.3*	65002624	rs28822416	0.8420	64257845	C	T	0.45	1.54E-05	4.02E-02	6.75E-02
rs12908891	Brain_Frontal_Cortex_BA9	15	*RP11-244F12.3*	63339909	rs8035776	0.8420	64258018	G	A	0.45	1.54E-05	4.82E-02	7.62E-02
rs12908891	Brain_Frontal_Cortex_BA9	15	*AC100830.3*	65002624	rs8035776	0.8420	64258018	G	A	0.45	1.54E-05	4.02E-02	6.75E-02
rs12908891	Brain_Frontal_Cortex_BA9	15	*RP11-244F12.3*	63339909	rs6494456	0.8381	64258260	C	T	0.45	1.54E-05	4.82E-02	7.62E-02
rs12908891	Brain_Frontal_Cortex_BA9	15	*AC100830.3*	65002624	rs6494456	0.8381	64258260	C	T	0.45	1.54E-05	4.02E-02	6.75E-02
rs12908891	Brain_Hippocampus	15	*AC100830.4*	64987516	rs1460544	0.8736	64201766	T	C	0.51	9.06E-04	4.92E-02	1.67E-01
rs12908891	Brain_Hippocampus	15	*AC100830.4*	64987516	rs11071773	0.8883	64206765	A	G	0.51	8.95E-04	4.52E-02	1.37E-01
rs12908891	Brain_Hippocampus	15	*AC100830.4*	64987516	rs12909081	0.8809	64208260	A	C	0.51	1.00E-03	4.68E-02	1.42E-01
rs12908891	Brain_Hippocampus	15	*AC100830.4*	64987516	rs2414844	0.8846	64210675	A	G	0.51	9.01E-04	4.68E-02	1.39E-01
rs12908891	Brain_Hippocampus	15	*AC100830.4*	64987516	rs12592060	0.8846	64210780	T	C	0.51	9.01E-04	4.68E-02	1.39E-01
rs12908891	Brain_Hippocampus	15	*AC100830.4*	64987516	rs1471282	0.8846	64212275	G	T	0.51	1.07E-03	4.68E-02	1.45E-01
rs12908891	Brain_Hippocampus	15	*AC100830.4*	64987516	rs11853632	0.8921	64217845	C	G	0.50	4.21E-05	4.28E-02	8.16E-02
rs12908891	Brain_Hippocampus	15	*AC100830.4*	64987516	rs12907405	0.8884	64220046	T	C	0.50	4.35E-05	3.03E-02	6.78E-02
rs12908891	Brain_Hippocampus	15	*AC100830.4*	64987516	rs11071775	0.8808	64220995	A	G	0.50	4.81E-05	3.74E-02	7.60E-02
rs12908891	Brain_Hippocampus	15	*AC100830.4*	64987516	rs11071776	0.8808	64221010	T	A	0.50	5.49E-05	3.74E-02	7.70E-02
rs12908891	Brain_Hippocampus	15	*AC100830.4*	64987516	rs11637858	0.8959	64224107	C	T	0.49	4.60E-05	3.74E-02	7.56E-02
rs12908891	Brain_Hippocampus	15	*AC100830.4*	64987516	rs12916395	0.9921	64225563	T	C	0.51	1.86E-06	3.79E-02	5.56E-02
rs12908891	Brain_Hippocampus	15	*AC100830.4*	64987516	rs11630587	0.9921	64225908	C	T	0.51	1.77E-06	4.89E-02	6.72E-02
rs12908891	Brain_Hippocampus	15	*LACTB*	63424129	rs11071777	0.8557	64229285	C	G	0.55	9.20E-06	2.62E-02	4.50E-02
rs12908891	Brain_Hippocampus	15	*AC100830.4*	64987516	rs11071777	0.8557	64229285	C	G	0.55	9.20E-06	7.92E-03	2.12E-02
rs12908891	Brain_Hippocampus	15	*AC100830.4*	64987516	rs1563886	0.9272	64230852	G	C	0.49	8.06E-06	3.28E-02	7.36E-02
rs12908891	Brain_Hippocampus	15	*AC100830.4*	64987516	rs10744961	0.9960	64230930	A	G	0.51	1.86E-06	4.73E-02	6.60E-02
rs12908891	Brain_Hippocampus	15	*AC100830.4*	64987516	rs4776268	1.0000	64236398	A	G	0.51	6.19E-06	2.25E-02	7.70E-02
rs12908891	Brain_Hippocampus	15	*FBXL22*	63892089	rs12908891	1.0000	64236441	A	G	0.52	1.39E-06	4.97E-02	6.81E-02
rs12908891	Brain_Hippocampus	15	*AC100830.4*	64987516	rs12908891	1.0000	64236441	A	G	0.52	1.39E-06	4.44E-02	6.25E-02
rs12908891	Brain_Hippocampus	15	*AC100830.4*	64987516	rs11071779	0.9881	64239044	G	A	0.51	1.57E-06	2.58E-02	4.27E-02
rs12908891	Brain_Hippocampus	15	*AC100830.4*	64987516	rs11071780	0.9842	64241691	A	G	0.51	4.74E-06	2.25E-02	7.96E-02

rs12908891	Brain_Hippocampus	15	*AC100830.4*	64987516	rs11854537	0.9842	64241733	A	G	0.51	4.74E-06	2.25E-02	7.96E-02
rs12908891	Brain_Hippocampus	15	*AC100830.4*	64987516	rs1380844	0.9881	64242007	G	T	0.51	4.74E-06	2.25E-02	7.96E-02
rs12908891	Brain_Hippocampus	15	*AC100830.4*	64987516	rs12916806	0.9881	64242770	C	A	0.51	4.64E-06	2.25E-02	7.96E-02
rs12908891	Brain_Hippocampus	15	*AC100830.4*	64987516	rs11071781	0.9195	64243933	G	A	0.49	4.75E-06	3.74E-02	8.02E-02
rs12908891	Brain_Hippocampus	15	*AC100830.4*	64987516	rs1304365	0.9881	64245946	A	G	0.51	4.67E-06	2.25E-02	7.97E-02
rs12908891	Brain_Hippocampus	15	*AC100830.4*	64987516	rs145159458	0.9119	64252880	T	C	0.49	1.21E-05	3.28E-02	8.58E-02
rs12908891	Brain_Hippocampus	15	*AC100830.4*	64987516	rs4776692	0.9881	64255083	G	A	0.50	1.06E-05	2.25E-02	8.89E-02
rs12908891	Brain_Hippocampus	15	*FBXL22*	63892089	rs28822416	0.8420	64257845	C	T	0.45	1.54E-05	4.15E-02	6.90E-02
rs12908891	Brain_Hippocampus	15	*FBXL22*	63892089	rs8035776	0.8420	64258018	G	A	0.45	1.54E-05	4.15E-02	6.90E-02
rs12908891	Brain_Hippocampus	15	*FBXL22*	63892089	rs6494456	0.8381	64258260	C	T	0.45	1.54E-05	4.15E-02	6.90E-02
rs12908891	Brain_Hippocampus	15	*RAB8B*	63520824	rs4776695	0.8350	64259115	T	C	0.46	9.12E-06	3.32E-02	5.67E-02
rs12908891	Brain_Hypothalamus	15	*CA12*	63643968	rs749468	0.9493	64210279	T	C	0.53	2.79E-04	4.01E-02	7.20E-02
rs12908891	Brain_Hypothalamus	15	*CA12*	63643968	rs12904374	0.8808	64214670	T	A	0.51	8.63E-04	4.81E-02	8.40E-02
rs12908891	Brain_Hypothalamus	15	*CA12*	63643968	rs920762	0.9379	64214954	C	G	0.47	1.24E-05	2.58E-02	4.45E-02
rs12908891	Brain_Hypothalamus	15	*CA12*	63643968	rs6494454	0.8031	64233563	A	C	0.56	2.52E-05	2.48E-02	4.73E-02
rs12908891	Brain_Hypothalamus	15	*CA12*	63643968	rs894660	0.8031	64234334	C	T	0.56	2.52E-05	2.48E-02	4.73E-02
rs12908891	Brain_Hypothalamus	15	*CA12*	63643968	rs4776266	0.8031	64236175	G	T	0.56	2.23E-05	2.48E-02	4.71E-02
rs12908891	Brain_Nucleus_accumbens_basal_ganglia	15	*TPM1*	63349472	rs1460544	0.8736	64201766	T	C	0.51	9.06E-04	3.59E-02	1.54E-01
rs12908891	Brain_Nucleus_accumbens_basal_ganglia	15	*USP3-AS1*	63864970	rs1460544	0.8736	64201766	T	C	0.51	9.06E-04	1.07E-02	1.22E-01
rs12908891	Brain_Nucleus_accumbens_basal_ganglia	15	*PPIB*	64453853	rs1460544	0.8736	64201766	T	C	0.51	9.06E-04	3.26E-02	1.51E-01
rs12908891	Brain_Nucleus_accumbens_basal_ganglia	15	*TPM1*	63349472	rs1037846	0.8696	64204870	G	A	0.51	6.33E-04	4.44E-02	7.73E-02
rs12908891	Brain_Nucleus_accumbens_basal_ganglia	15	*USP3-AS1*	63864970	rs1037846	0.8696	64204870	G	A	0.51	6.33E-04	1.55E-02	4.28E-02
rs12908891	Brain_Nucleus_accumbens_basal_ganglia	15	*PPIB*	64453853	rs1037846	0.8696	64204870	G	A	0.51	6.33E-04	4.00E-02	7.25E-02
rs12908891	Brain_Nucleus_accumbens_basal_ganglia	15	*TPM1*	63349472	rs11071773	0.8883	64206765	A	G	0.51	8.95E-04	3.22E-02	1.23E-01
rs12908891	Brain_Nucleus_accumbens_basal_ganglia	15	*USP3-AS1*	63864970	rs11071773	0.8883	64206765	A	G	0.51	8.95E-04	2.28E-02	1.12E-01
rs12908891	Brain_Nucleus_accumbens_basal_ganglia	15	*PPIB*	64453853	rs11071773	0.8883	64206765	A	G	0.51	8.95E-04	2.62E-02	1.16E-01
rs12908891	Brain_Nucleus_accumbens_basal_ganglia	15	*RP11-244F12.3*	63339909	rs12909081	0.8809	64208260	A	C	0.51	1.00E-03	4.46E-02	1.40E-01
rs12908891	Brain_Nucleus_accumbens_basal_ganglia	15	*TPM1*	63349472	rs12909081	0.8809	64208260	A	C	0.51	1.00E-03	3.77E-02	1.33E-01
rs12908891	Brain_Nucleus_accumbens_basal_ganglia	15	*USP3-AS1*	63864970	rs12909081	0.8809	64208260	A	C	0.51	1.00E-03	1.38E-02	1.03E-01
rs12908891	Brain_Nucleus_accumbens_basal_ganglia	15	*PPIB*	64453853	rs12909081	0.8809	64208260	A	C	0.51	1.00E-03	1.97E-02	1.12E-01
rs12908891	Brain_Nucleus_accumbens_basal_ganglia	15	*TPM1*	63349472	rs749468	0.9493	64210279	T	C	0.53	2.79E-04	4.28E-02	7.50E-02
rs12908891	Brain_Nucleus_accumbens_basal_ganglia	15	*USP3-AS1*	63864970	rs749468	0.9493	64210279	T	C	0.53	2.79E-04	2.16E-02	5.04E-02
rs12908891	Brain_Nucleus_accumbens_basal_ganglia	15	*PPIB*	64453853	rs749468	0.9493	64210279	T	C	0.53	2.79E-04	3.46E-02	6.58E-02
rs12908891	Brain_Nucleus_accumbens_basal_ganglia	15	*RP11-244F12.3*	63339909	rs2414844	0.8846	64210675	A	G	0.51	9.01E-04	4.46E-02	1.37E-01
rs12908891	Brain_Nucleus_accumbens_basal_ganglia	15	*TPM1*	63349472	rs2414844	0.8846	64210675	A	G	0.51	9.01E-04	3.77E-02	1.30E-01
rs12908891	Brain_Nucleus_accumbens_basal_ganglia	15	*USP3-AS1*	63864970	rs2414844	0.8846	64210675	A	G	0.51	9.01E-04	1.38E-02	1.00E-01
rs12908891	Brain_Nucleus_accumbens_basal_ganglia	15	*PPIB*	64453853	rs2414844	0.8846	64210675	A	G	0.51	9.01E-04	1.97E-02	1.09E-01
rs12908891	Brain_Nucleus_accumbens_basal_ganglia	15	*RP11-244F12.3*	63339909	rs12592060	0.8846	64210780	T	C	0.51	9.01E-04	4.46E-02	1.37E-01
rs12908891	Brain_Nucleus_accumbens_basal_ganglia	15	*TPM1*	63349472	rs12592060	0.8846	64210780	T	C	0.51	9.01E-04	3.77E-02	1.30E-01
rs12908891	Brain_Nucleus_accumbens_basal_ganglia	15	*USP3-AS1*	63864970	rs12592060	0.8846	64210780	T	C	0.51	9.01E-04	1.38E-02	1.00E-01

rs12908891	Brain_Nucleus_accumbens_basal_ganglia	15	*PPIB*	64453853	rs12592060	0.8846	64210780	T	C	0.51	9.01E-04	1.97E-02	1.09E-01
rs12908891	Brain_Nucleus_accumbens_basal_ganglia	15	*RP11-244F12.3*	63339909	rs1471282	0.8846	64212275	G	T	0.51	1.07E-03	4.46E-02	1.43E-01
rs12908891	Brain_Nucleus_accumbens_basal_ganglia	15	*TPM1*	63349472	rs1471282	0.8846	64212275	G	T	0.51	1.07E-03	3.77E-02	1.36E-01
rs12908891	Brain_Nucleus_accumbens_basal_ganglia	15	*USP3-AS1*	63864970	rs1471282	0.8846	64212275	G	T	0.51	1.07E-03	1.38E-02	1.07E-01
rs12908891	Brain_Nucleus_accumbens_basal_ganglia	15	*PPIB*	64453853	rs1471282	0.8846	64212275	G	T	0.51	1.07E-03	1.97E-02	1.15E-01
rs12908891	Brain_Nucleus_accumbens_basal_ganglia	15	*RP11-244F12.3*	63339909	rs11635779	0.8808	64213826	T	C	0.50	6.34E-05	4.09E-02	8.26E-02
rs12908891	Brain_Nucleus_accumbens_basal_ganglia	15	*TPM1*	63349472	rs11635779	0.8808	64213826	T	C	0.50	6.34E-05	3.08E-02	7.12E-02
rs12908891	Brain_Nucleus_accumbens_basal_ganglia	15	*USP3-AS1*	63864970	rs11635779	0.8808	64213826	T	C	0.50	6.34E-05	1.86E-02	5.58E-02
rs12908891	Brain_Nucleus_accumbens_basal_ganglia	15	*PPIB*	64453853	rs11635779	0.8808	64213826	T	C	0.50	6.34E-05	2.39E-02	6.28E-02
rs12908891	Brain_Nucleus_accumbens_basal_ganglia	15	*TPM1*	63349472	rs12904374	0.8808	64214670	T	A	0.51	8.63E-04	2.61E-02	5.92E-02
rs12908891	Brain_Nucleus_accumbens_basal_ganglia	15	*USP3-AS1*	63864970	rs12904374	0.8808	64214670	T	A	0.51	8.63E-04	2.94E-02	6.31E-02
rs12908891	Brain_Nucleus_accumbens_basal_ganglia	15	*PPIB*	64453853	rs12904374	0.8808	64214670	T	A	0.51	8.63E-04	3.11E-02	6.51E-02
rs12908891	Brain_Nucleus_accumbens_basal_ganglia	15	*USP3-AS1*	63864970	rs920762	0.9379	64214954	C	G	0.47	1.24E-05	2.71E-02	4.60E-02
rs12908891	Brain_Nucleus_accumbens_basal_ganglia	15	*TPM1*	63349472	rs11853632	0.8921	64217845	C	G	0.50	4.21E-05	3.51E-02	7.31E-02
rs12908891	Brain_Nucleus_accumbens_basal_ganglia	15	*USP3-AS1*	63864970	rs11853632	0.8921	64217845	C	G	0.50	4.21E-05	1.50E-02	4.77E-02
rs12908891	Brain_Nucleus_accumbens_basal_ganglia	15	*PPIB*	64453853	rs11853632	0.8921	64217845	C	G	0.50	4.21E-05	3.15E-02	6.89E-02
rs12908891	Brain_Nucleus_accumbens_basal_ganglia	15	*RP11-244F12.3*	63339909	rs12907405	0.8884	64220046	T	C	0.50	4.35E-05	3.87E-02	7.74E-02
rs12908891	Brain_Nucleus_accumbens_basal_ganglia	15	*TPM1*	63349472	rs12907405	0.8884	64220046	T	C	0.50	4.35E-05	4.61E-02	8.54E-02
rs12908891	Brain_Nucleus_accumbens_basal_ganglia	15	*USP3-AS1*	63864970	rs12907405	0.8884	64220046	T	C	0.50	4.35E-05	1.62E-02	4.97E-02
rs12908891	Brain_Nucleus_accumbens_basal_ganglia	15	*PPIB*	64453853	rs12907405	0.8884	64220046	T	C	0.50	4.35E-05	2.79E-02	6.50E-02
rs12908891	Brain_Nucleus_accumbens_basal_ganglia	15	*RP11-244F12.3*	63339909	rs11071775	0.8808	64220995	A	G	0.50	4.81E-05	3.09E-02	6.86E-02
rs12908891	Brain_Nucleus_accumbens_basal_ganglia	15	*TPM1*	63349472	rs11071775	0.8808	64220995	A	G	0.50	4.81E-05	4.50E-02	8.44E-02
rs12908891	Brain_Nucleus_accumbens_basal_ganglia	15	*USP3-AS1*	63864970	rs11071775	0.8808	64220995	A	G	0.50	4.81E-05	2.01E-02	5.52E-02
rs12908891	Brain_Nucleus_accumbens_basal_ganglia	15	*PPIB*	64453853	rs11071775	0.8808	64220995	A	G	0.50	4.81E-05	2.72E-02	6.42E-02
rs12908891	Brain_Nucleus_accumbens_basal_ganglia	15	*RP11-244F12.3*	63339909	rs11071776	0.8808	64221010	T	A	0.50	5.49E-05	3.09E-02	6.96E-02
rs12908891	Brain_Nucleus_accumbens_basal_ganglia	15	*TPM1*	63349472	rs11071776	0.8808	64221010	T	A	0.50	5.49E-05	4.50E-02	8.54E-02
rs12908891	Brain_Nucleus_accumbens_basal_ganglia	15	*USP3-AS1*	63864970	rs11071776	0.8808	64221010	T	A	0.50	5.49E-05	2.01E-02	5.62E-02
rs12908891	Brain_Nucleus_accumbens_basal_ganglia	15	*PPIB*	64453853	rs11071776	0.8808	64221010	T	A	0.50	5.49E-05	2.72E-02	6.51E-02
rs12908891	Brain_Nucleus_accumbens_basal_ganglia	15	*RP11-244F12.3*	63339909	rs11637858	0.8959	64224107	C	T	0.49	4.60E-05	3.49E-02	7.28E-02
rs12908891	Brain_Nucleus_accumbens_basal_ganglia	15	*USP3-AS1*	63864970	rs11637858	0.8959	64224107	C	T	0.49	4.60E-05	1.45E-02	4.69E-02
rs12908891	Brain_Nucleus_accumbens_basal_ganglia	15	*PPIB*	64453853	rs11637858	0.8959	64224107	C	T	0.49	4.60E-05	2.67E-02	6.32E-02
rs12908891	Brain_Nucleus_accumbens_basal_ganglia	15	*USP3-AS1*	63864970	rs12916395	0.9921	64225563	T	C	0.51	1.86E-06	1.16E-02	2.46E-02
rs12908891	Brain_Nucleus_accumbens_basal_ganglia	15	*SNX22*	64446798	rs12916395	0.9921	64225563	T	C	0.51	1.86E-06	3.81E-02	5.58E-02
rs12908891	Brain_Nucleus_accumbens_basal_ganglia	15	*USP3-AS1*	63864970	rs11630587	0.9921	64225908	C	T	0.51	1.77E-06	2.20E-02	3.76E-02
rs12908891	Brain_Nucleus_accumbens_basal_ganglia	15	*USP3-AS1*	63864970	rs4776677	0.9921	64227044	C	T	0.51	1.54E-06	1.14E-02	2.40E-02
rs12908891	Brain_Nucleus_accumbens_basal_ganglia	15	*USP3-AS1*	63864970	rs11071777	0.8557	64229285	C	G	0.55	9.20E-06	5.13E-03	1.66E-02
rs12908891	Brain_Nucleus_accumbens_basal_ganglia	15	*SNX22*	64446798	rs11071777	0.8557	64229285	C	G	0.55	9.20E-06	1.77E-02	3.48E-02
rs12908891	Brain_Nucleus_accumbens_basal_ganglia	15	*RP11-244F12.3*	63339909	rs1563886	0.9272	64230852	G	C	0.49	8.06E-06	4.28E-02	8.47E-02
rs12908891	Brain_Nucleus_accumbens_basal_ganglia	15	*RP11-321G12.1*	63705841	rs1563886	0.9272	64230852	G	C	0.49	8.06E-06	4.61E-02	8.82E-02
rs12908891	Brain_Nucleus_accumbens_basal_ganglia	15	*USP3-AS1*	63864970	rs1563886	0.9272	64230852	G	C	0.49	8.06E-06	2.07E-02	5.86E-02

rs12908891	Brain_Nucleus_accumbens_basal_ganglia	15	*PPIB*	64453853	rs1563886	0.9272	64230852	G	C	0.49	8.06E-06	3.22E-02	7.28E-02
rs12908891	Brain_Nucleus_accumbens_basal_ganglia	15	*USP3-AS1*	63864970	rs10744961	0.9960	64230930	A	G	0.51	1.86E-06	1.64E-02	3.11E-02
rs12908891	Brain_Nucleus_accumbens_basal_ganglia	15	*USP3-AS1*	63864970	rs6494454	0.8031	64233563	A	C	0.56	2.52E-05	9.58E-03	2.71E-02
rs12908891	Brain_Nucleus_accumbens_basal_ganglia	15	*USP3-AS1*	63864970	rs894660	0.8031	64234334	C	T	0.56	2.52E-05	9.58E-03	2.71E-02
rs12908891	Brain_Nucleus_accumbens_basal_ganglia	15	*USP3-AS1*	63864970	rs4776266	0.8031	64236175	G	T	0.56	2.23E-05	7.63E-03	2.38E-02
rs12908891	Brain_Nucleus_accumbens_basal_ganglia	15	*USP3-AS1*	63864970	rs4776268	1.0000	64236398	A	G	0.51	6.19E-06	1.44E-02	6.55E-02
rs12908891	Brain_Nucleus_accumbens_basal_ganglia	15	*SNX22*	64446798	rs4776268	1.0000	64236398	A	G	0.51	6.19E-06	4.81E-02	1.06E-01
rs12908891	Brain_Nucleus_accumbens_basal_ganglia	15	*PPIB*	64453853	rs4776268	1.0000	64236398	A	G	0.51	6.19E-06	4.24E-02	1.00E-01
rs12908891	Brain_Nucleus_accumbens_basal_ganglia	15	*RP11-244F12.3*	63339909	rs12908891	1.0000	64236441	A	G	0.52	1.39E-06	3.96E-02	5.74E-02
rs12908891	Brain_Nucleus_accumbens_basal_ganglia	15	*USP3-AS1*	63864970	rs12908891	1.0000	64236441	A	G	0.52	1.39E-06	7.63E-03	1.88E-02
rs12908891	Brain_Nucleus_accumbens_basal_ganglia	15	*SNX22*	64446798	rs12908891	1.0000	64236441	A	G	0.52	1.39E-06	3.18E-02	4.89E-02
rs12908891	Brain_Nucleus_accumbens_basal_ganglia	15	*USP3-AS1*	63864970	rs11071779	0.9881	64239044	G	A	0.51	1.57E-06	7.78E-03	1.95E-02
rs12908891	Brain_Nucleus_accumbens_basal_ganglia	15	*SNX22*	64446798	rs11071779	0.9881	64239044	G	A	0.51	1.57E-06	1.65E-02	3.14E-02
rs12908891	Brain_Nucleus_accumbens_basal_ganglia	15	*PPIB*	64453853	rs11071779	0.9881	64239044	G	A	0.51	1.57E-06	4.44E-02	6.31E-02
rs12908891	Brain_Nucleus_accumbens_basal_ganglia	15	*RP11-321G12.1*	63705841	rs11071780	0.9842	64241691	A	G	0.51	4.74E-06	4.48E-02	1.06E-01
rs12908891	Brain_Nucleus_accumbens_basal_ganglia	15	*USP3-AS1*	63864970	rs11071780	0.9842	64241691	A	G	0.51	4.74E-06	8.42E-03	5.76E-02
rs12908891	Brain_Nucleus_accumbens_basal_ganglia	15	*SNX22*	64446798	rs11071780	0.9842	64241691	A	G	0.51	4.74E-06	1.83E-02	7.39E-02
rs12908891	Brain_Nucleus_accumbens_basal_ganglia	15	*PPIB*	64453853	rs11071780	0.9842	64241691	A	G	0.51	4.74E-06	2.22E-02	7.92E-02
rs12908891	Brain_Nucleus_accumbens_basal_ganglia	15	*RP11-321G12.1*	63705841	rs11854537	0.9842	64241733	A	G	0.51	4.74E-06	4.48E-02	1.06E-01
rs12908891	Brain_Nucleus_accumbens_basal_ganglia	15	*USP3-AS1*	63864970	rs11854537	0.9842	64241733	A	G	0.51	4.74E-06	8.42E-03	5.76E-02
rs12908891	Brain_Nucleus_accumbens_basal_ganglia	15	*SNX22*	64446798	rs11854537	0.9842	64241733	A	G	0.51	4.74E-06	1.83E-02	7.39E-02
rs12908891	Brain_Nucleus_accumbens_basal_ganglia	15	*PPIB*	64453853	rs11854537	0.9842	64241733	A	G	0.51	4.74E-06	2.22E-02	7.92E-02
rs12908891	Brain_Nucleus_accumbens_basal_ganglia	15	*RP11-321G12.1*	63705841	rs1380844	0.9881	64242007	G	T	0.51	4.74E-06	4.48E-02	1.06E-01
rs12908891	Brain_Nucleus_accumbens_basal_ganglia	15	*USP3-AS1*	63864970	rs1380844	0.9881	64242007	G	T	0.51	4.74E-06	8.42E-03	5.76E-02
rs12908891	Brain_Nucleus_accumbens_basal_ganglia	15	*SNX22*	64446798	rs1380844	0.9881	64242007	G	T	0.51	4.74E-06	1.83E-02	7.39E-02
rs12908891	Brain_Nucleus_accumbens_basal_ganglia	15	*PPIB*	64453853	rs1380844	0.9881	64242007	G	T	0.51	4.74E-06	2.22E-02	7.92E-02
rs12908891	Brain_Nucleus_accumbens_basal_ganglia	15	*RP11-321G12.1*	63705841	rs12916806	0.9881	64242770	C	A	0.51	4.64E-06	4.48E-02	1.06E-01
rs12908891	Brain_Nucleus_accumbens_basal_ganglia	15	*USP3-AS1*	63864970	rs12916806	0.9881	64242770	C	A	0.51	4.64E-06	8.42E-03	5.76E-02
rs12908891	Brain_Nucleus_accumbens_basal_ganglia	15	*SNX22*	64446798	rs12916806	0.9881	64242770	C	A	0.51	4.64E-06	1.83E-02	7.39E-02
rs12908891	Brain_Nucleus_accumbens_basal_ganglia	15	*PPIB*	64453853	rs12916806	0.9881	64242770	C	A	0.51	4.64E-06	2.22E-02	7.91E-02
rs12908891	Brain_Nucleus_accumbens_basal_ganglia	15	*TPM1*	63349472	rs11071781	0.9195	64243933	G	A	0.49	4.75E-06	4.89E-02	9.27E-02
rs12908891	Brain_Nucleus_accumbens_basal_ganglia	15	*USP3-AS1*	63864970	rs11071781	0.9195	64243933	G	A	0.49	4.75E-06	8.63E-03	4.16E-02
rs12908891	Brain_Nucleus_accumbens_basal_ganglia	15	*SNX22*	64446798	rs11071781	0.9195	64243933	G	A	0.49	4.75E-06	2.13E-02	6.07E-02
rs12908891	Brain_Nucleus_accumbens_basal_ganglia	15	*PPIB*	64453853	rs11071781	0.9195	64243933	G	A	0.49	4.75E-06	1.37E-02	5.00E-02
rs12908891	Brain_Nucleus_accumbens_basal_ganglia	15	*RP11-321G12.1*	63705841	rs1304365	0.9881	64245946	A	G	0.51	4.67E-06	4.48E-02	1.06E-01
rs12908891	Brain_Nucleus_accumbens_basal_ganglia	15	*USP3-AS1*	63864970	rs1304365	0.9881	64245946	A	G	0.51	4.67E-06	8.42E-03	5.77E-02
rs12908891	Brain_Nucleus_accumbens_basal_ganglia	15	*SNX22*	64446798	rs1304365	0.9881	64245946	A	G	0.51	4.67E-06	1.83E-02	7.40E-02
rs12908891	Brain_Nucleus_accumbens_basal_ganglia	15	*PPIB*	64453853	rs1304365	0.9881	64245946	A	G	0.51	4.67E-06	2.22E-02	7.92E-02
rs12908891	Brain_Nucleus_accumbens_basal_ganglia	15	*RP11-321G12.1*	63705841	rs145159458	0.9119	64252880	T	C	0.49	1.21E-05	3.31E-02	8.61E-02
rs12908891	Brain_Nucleus_accumbens_basal_ganglia	15	*USP3-AS1*	63864970	rs145159458	0.9119	64252880	T	C	0.49	1.21E-05	1.25E-02	5.86E-02

rs12908891	Brain_Nucleus_accumbens_basal_ganglia	15	*SNX22*	64446798	rs145159458	0.9119	64252880	T	C	0.49	1.21E-05	2.03E-02	7.01E-02
rs12908891	Brain_Nucleus_accumbens_basal_ganglia	15	*PPIB*	64453853	rs145159458	0.9119	64252880	T	C	0.49	1.21E-05	1.67E-02	6.51E-02
rs12908891	Brain_Nucleus_accumbens_basal_ganglia	15	*RP11-321G12.1*	63705841	rs4776692	0.9881	64255083	G	A	0.50	1.06E-05	4.48E-02	1.15E-01
rs12908891	Brain_Nucleus_accumbens_basal_ganglia	15	*USP3-AS1*	63864970	rs4776692	0.9881	64255083	G	A	0.50	1.06E-05	8.42E-03	6.68E-02
rs12908891	Brain_Nucleus_accumbens_basal_ganglia	15	*SNX22*	64446798	rs4776692	0.9881	64255083	G	A	0.50	1.06E-05	1.83E-02	8.32E-02
rs12908891	Brain_Nucleus_accumbens_basal_ganglia	15	*PPIB*	64453853	rs4776692	0.9881	64255083	G	A	0.50	1.06E-05	2.22E-02	8.85E-02
rs12908891	Brain_Nucleus_accumbens_basal_ganglia	15	*USP3-AS1*	63864970	rs28822416	0.8420	64257845	C	T	0.45	1.54E-05	4.76E-02	7.55E-02
rs12908891	Brain_Nucleus_accumbens_basal_ganglia	15	*USP3-AS1*	63864970	rs8035776	0.8420	64258018	G	A	0.45	1.54E-05	4.76E-02	7.55E-02
rs12908891	Brain_Nucleus_accumbens_basal_ganglia	15	*USP3-AS1*	63864970	rs6494456	0.8381	64258260	C	T	0.45	1.54E-05	4.76E-02	7.55E-02
rs12908891	Brain_Nucleus_accumbens_basal_ganglia	15	*PPIB*	64453853	rs4776695	0.8350	64259115	T	C	0.46	9.12E-06	2.97E-02	5.26E-02
rs12908891	Brain_Nucleus_accumbens_basal_ganglia	15	*AC100830.4*	64987516	rs4776695	0.8350	64259115	T	C	0.46	9.12E-06	4.60E-02	7.06E-02
rs12908891	Brain_Putamen_basal_ganglia	15	*TPM1*	63349472	rs1460544	0.8736	64201766	T	C	0.51	9.06E-04	2.76E-02	1.45E-01
rs12908891	Brain_Putamen_basal_ganglia	15	*RP11-321G12.1*	63705841	rs1460544	0.8736	64201766	T	C	0.51	9.06E-04	2.82E-02	1.46E-01
rs12908891	Brain_Putamen_basal_ganglia	15	*SNX22*	64446798	rs1460544	0.8736	64201766	T	C	0.51	9.06E-04	1.21E-02	1.24E-01
rs12908891	Brain_Putamen_basal_ganglia	15	*RBPMS2*	65049938	rs1460544	0.8736	64201766	T	C	0.51	9.06E-04	3.87E-02	1.57E-01
rs12908891	Brain_Putamen_basal_ganglia	15	*TPM1*	63349472	rs1037846	0.8696	64204870	G	A	0.51	6.33E-04	3.99E-02	7.24E-02
rs12908891	Brain_Putamen_basal_ganglia	15	*RP11-321G12.1*	63705841	rs1037846	0.8696	64204870	G	A	0.51	6.33E-04	4.24E-02	7.51E-02
rs12908891	Brain_Putamen_basal_ganglia	15	*SNX22*	64446798	rs1037846	0.8696	64204870	G	A	0.51	6.33E-04	9.30E-03	3.33E-02
rs12908891	Brain_Putamen_basal_ganglia	15	*TPM1*	63349472	rs11071773	0.8883	64206765	A	G	0.51	8.95E-04	2.76E-02	1.18E-01
rs12908891	Brain_Putamen_basal_ganglia	15	*RP11-321G12.1*	63705841	rs11071773	0.8883	64206765	A	G	0.51	8.95E-04	2.73E-02	1.18E-01
rs12908891	Brain_Putamen_basal_ganglia	15	*FBXL22*	63892089	rs11071773	0.8883	64206765	A	G	0.51	8.95E-04	4.27E-02	1.35E-01
rs12908891	Brain_Putamen_basal_ganglia	15	*SNX22*	64446798	rs11071773	0.8883	64206765	A	G	0.51	8.95E-04	2.06E-02	1.09E-01
rs12908891	Brain_Putamen_basal_ganglia	15	*RBPMS2*	65049938	rs11071773	0.8883	64206765	A	G	0.51	8.95E-04	4.74E-02	1.39E-01
rs12908891	Brain_Putamen_basal_ganglia	15	*TPM1*	63349472	rs12909081	0.8809	64208260	A	C	0.51	1.00E-03	2.03E-02	1.13E-01
rs12908891	Brain_Putamen_basal_ganglia	15	*RP11-321G12.1*	63705841	rs12909081	0.8809	64208260	A	C	0.51	1.00E-03	2.64E-02	1.20E-01
rs12908891	Brain_Putamen_basal_ganglia	15	*FBXL22*	63892089	rs12909081	0.8809	64208260	A	C	0.51	1.00E-03	3.80E-02	1.33E-01
rs12908891	Brain_Putamen_basal_ganglia	15	*SNX22*	64446798	rs12909081	0.8809	64208260	A	C	0.51	1.00E-03	1.35E-02	1.03E-01
rs12908891	Brain_Putamen_basal_ganglia	15	*RBPMS2*	65049938	rs12909081	0.8809	64208260	A	C	0.51	1.00E-03	4.62E-02	1.42E-01
rs12908891	Brain_Putamen_basal_ganglia	15	*TPM1*	63349472	rs749468	0.9493	64210279	T	C	0.53	2.79E-04	1.16E-02	3.65E-02
rs12908891	Brain_Putamen_basal_ganglia	15	*RP11-321G12.1*	63705841	rs749468	0.9493	64210279	T	C	0.53	2.79E-04	2.71E-02	5.71E-02
rs12908891	Brain_Putamen_basal_ganglia	15	*FBXL22*	63892089	rs749468	0.9493	64210279	T	C	0.53	2.79E-04	3.12E-02	6.20E-02
rs12908891	Brain_Putamen_basal_ganglia	15	*SNX22*	64446798	rs749468	0.9493	64210279	T	C	0.53	2.79E-04	8.85E-03	3.20E-02
rs12908891	Brain_Putamen_basal_ganglia	15	*TRIP4*	64713724	rs749468	0.9493	64210279	T	C	0.53	2.79E-04	4.14E-02	7.34E-02
rs12908891	Brain_Putamen_basal_ganglia	15	*RBPMS2*	65049938	rs749468	0.9493	64210279	T	C	0.53	2.79E-04	2.95E-02	6.00E-02
rs12908891	Brain_Putamen_basal_ganglia	15	*TPM1*	63349472	rs2414844	0.8846	64210675	A	G	0.51	9.01E-04	2.03E-02	1.09E-01
rs12908891	Brain_Putamen_basal_ganglia	15	*RP11-321G12.1*	63705841	rs2414844	0.8846	64210675	A	G	0.51	9.01E-04	2.64E-02	1.17E-01
rs12908891	Brain_Putamen_basal_ganglia	15	*FBXL22*	63892089	rs2414844	0.8846	64210675	A	G	0.51	9.01E-04	3.80E-02	1.30E-01
rs12908891	Brain_Putamen_basal_ganglia	15	*SNX22*	64446798	rs2414844	0.8846	64210675	A	G	0.51	9.01E-04	1.35E-02	9.95E-02
rs12908891	Brain_Putamen_basal_ganglia	15	*RBPMS2*	65049938	rs2414844	0.8846	64210675	A	G	0.51	9.01E-04	4.62E-02	1.39E-01
rs12908891	Brain_Putamen_basal_ganglia	15	*TPM1*	63349472	rs12592060	0.8846	64210780	T	C	0.51	9.01E-04	2.03E-02	1.09E-01

rs12908891	Brain_Putamen_basal_ganglia	15	*RP11-321G12.1*	63705841	rs12592060	0.8846	64210780	T	C	0.51	9.01E-04	2.64E-02	1.17E-01
rs12908891	Brain_Putamen_basal_ganglia	15	*FBXL22*	63892089	rs12592060	0.8846	64210780	T	C	0.51	9.01E-04	3.80E-02	1.30E-01
rs12908891	Brain_Putamen_basal_ganglia	15	*SNX22*	64446798	rs12592060	0.8846	64210780	T	C	0.51	9.01E-04	1.35E-02	9.95E-02
rs12908891	Brain_Putamen_basal_ganglia	15	*RBPMS2*	65049938	rs12592060	0.8846	64210780	T	C	0.51	9.01E-04	4.62E-02	1.39E-01
rs12908891	Brain_Putamen_basal_ganglia	15	*TPM1*	63349472	rs1471282	0.8846	64212275	G	T	0.51	1.07E-03	2.03E-02	1.16E-01
rs12908891	Brain_Putamen_basal_ganglia	15	*RP11-321G12.1*	63705841	rs1471282	0.8846	64212275	G	T	0.51	1.07E-03	2.64E-02	1.24E-01
rs12908891	Brain_Putamen_basal_ganglia	15	*FBXL22*	63892089	rs1471282	0.8846	64212275	G	T	0.51	1.07E-03	3.80E-02	1.37E-01
rs12908891	Brain_Putamen_basal_ganglia	15	*SNX22*	64446798	rs1471282	0.8846	64212275	G	T	0.51	1.07E-03	1.35E-02	1.06E-01
rs12908891	Brain_Putamen_basal_ganglia	15	*RBPMS2*	65049938	rs1471282	0.8846	64212275	G	T	0.51	1.07E-03	4.62E-02	1.45E-01
rs12908891	Brain_Putamen_basal_ganglia	15	*TPM1*	63349472	rs11635779	0.8808	64213826	T	C	0.50	6.34E-05	1.03E-02	4.33E-02
rs12908891	Brain_Putamen_basal_ganglia	15	*RP11-321G12.1*	63705841	rs11635779	0.8808	64213826	T	C	0.50	6.34E-05	2.76E-02	6.74E-02
rs12908891	Brain_Putamen_basal_ganglia	15	*FBXL22*	63892089	rs11635779	0.8808	64213826	T	C	0.50	6.34E-05	3.34E-02	7.42E-02
rs12908891	Brain_Putamen_basal_ganglia	15	*SNX22*	64446798	rs11635779	0.8808	64213826	T	C	0.50	6.34E-05	9.93E-03	4.26E-02
rs12908891	Brain_Putamen_basal_ganglia	15	*RBPMS2*	65049938	rs11635779	0.8808	64213826	T	C	0.50	6.34E-05	4.76E-02	8.99E-02
rs12908891	Brain_Putamen_basal_ganglia	15	*TPM1*	63349472	rs12904374	0.8808	64214670	T	A	0.51	8.63E-04	1.43E-02	4.36E-02
rs12908891	Brain_Putamen_basal_ganglia	15	*RP11-321G12.1*	63705841	rs12904374	0.8808	64214670	T	A	0.51	8.63E-04	2.81E-02	6.15E-02
rs12908891	Brain_Putamen_basal_ganglia	15	*FBXL22*	63892089	rs12904374	0.8808	64214670	T	A	0.51	8.63E-04	3.71E-02	7.20E-02
rs12908891	Brain_Putamen_basal_ganglia	15	*SNX22*	64446798	rs12904374	0.8808	64214670	T	A	0.51	8.63E-04	1.52E-02	4.48E-02
rs12908891	Brain_Putamen_basal_ganglia	15	*RBPMS2*	65049938	rs12904374	0.8808	64214670	T	A	0.51	8.63E-04	4.82E-02	8.40E-02
rs12908891	Brain_Putamen_basal_ganglia	15	*TPM1*	63349472	rs920762	0.9379	64214954	C	G	0.47	1.24E-05	2.80E-02	4.71E-02
rs12908891	Brain_Putamen_basal_ganglia	15	*FBXL22*	63892089	rs920762	0.9379	64214954	C	G	0.47	1.24E-05	3.53E-02	5.52E-02
rs12908891	Brain_Putamen_basal_ganglia	15	*SNX22*	64446798	rs920762	0.9379	64214954	C	G	0.47	1.24E-05	7.09E-03	1.99E-02
rs12908891	Brain_Putamen_basal_ganglia	15	*TRIP4*	64713724	rs920762	0.9379	64214954	C	G	0.47	1.24E-05	4.06E-02	6.11E-02
rs12908891	Brain_Putamen_basal_ganglia	15	*RBPMS2*	65049938	rs920762	0.9379	64214954	C	G	0.47	1.24E-05	4.98E-02	7.08E-02
rs12908891	Brain_Putamen_basal_ganglia	15	*TPM1*	63349472	rs11853632	0.8921	64217845	C	G	0.50	4.21E-05	2.03E-02	5.51E-02
rs12908891	Brain_Putamen_basal_ganglia	15	*RP11-321G12.1*	63705841	rs11853632	0.8921	64217845	C	G	0.50	4.21E-05	2.64E-02	6.29E-02
rs12908891	Brain_Putamen_basal_ganglia	15	*FBXL22*	63892089	rs11853632	0.8921	64217845	C	G	0.50	4.21E-05	3.80E-02	7.64E-02
rs12908891	Brain_Putamen_basal_ganglia	15	*SNX22*	64446798	rs11853632	0.8921	64217845	C	G	0.50	4.21E-05	1.35E-02	4.55E-02
rs12908891	Brain_Putamen_basal_ganglia	15	*RBPMS2*	65049938	rs11853632	0.8921	64217845	C	G	0.50	4.21E-05	4.62E-02	8.53E-02
rs12908891	Brain_Putamen_basal_ganglia	15	*TPM1*	63349472	rs12907405	0.8884	64220046	T	C	0.50	4.35E-05	2.58E-02	6.23E-02
rs12908891	Brain_Putamen_basal_ganglia	15	*RP11-321G12.1*	63705841	rs12907405	0.8884	64220046	T	C	0.50	4.35E-05	1.15E-02	4.26E-02
rs12908891	Brain_Putamen_basal_ganglia	15	*FBXL22*	63892089	rs12907405	0.8884	64220046	T	C	0.50	4.35E-05	3.59E-02	7.42E-02
rs12908891	Brain_Putamen_basal_ganglia	15	*SNX22*	64446798	rs12907405	0.8884	64220046	T	C	0.50	4.35E-05	1.07E-02	4.13E-02
rs12908891	Brain_Putamen_basal_ganglia	15	*TPM1*	63349472	rs11071775	0.8808	64220995	A	G	0.50	4.81E-05	1.41E-02	4.68E-02
rs12908891	Brain_Putamen_basal_ganglia	15	*RP11-321G12.1*	63705841	rs11071775	0.8808	64220995	A	G	0.50	4.81E-05	1.61E-02	4.96E-02
rs12908891	Brain_Putamen_basal_ganglia	15	*FBXL22*	63892089	rs11071775	0.8808	64220995	A	G	0.50	4.81E-05	4.65E-02	8.59E-02
rs12908891	Brain_Putamen_basal_ganglia	15	*SNX22*	64446798	rs11071775	0.8808	64220995	A	G	0.50	4.81E-05	1.35E-02	4.59E-02
rs12908891	Brain_Putamen_basal_ganglia	15	*TRIP4*	64713724	rs11071775	0.8808	64220995	A	G	0.50	4.81E-05	3.88E-02	7.76E-02
rs12908891	Brain_Putamen_basal_ganglia	15	*TPM1*	63349472	rs11071776	0.8808	64221010	T	A	0.50	5.49E-05	1.41E-02	4.77E-02
rs12908891	Brain_Putamen_basal_ganglia	15	*RP11-321G12.1*	63705841	rs11071776	0.8808	64221010	T	A	0.50	5.49E-05	1.61E-02	5.05E-02

rs12908891	Brain_Putamen_basal_ganglia	15	*FBXL22*	63892089	rs11071776	0.8808	64221010	T	A	0.50	5.49E-05	4.65E-02	8.69E-02
rs12908891	Brain_Putamen_basal_ganglia	15	*SNX22*	64446798	rs11071776	0.8808	64221010	T	A	0.50	5.49E-05	1.35E-02	4.68E-02
rs12908891	Brain_Putamen_basal_ganglia	15	*TRIP4*	64713724	rs11071776	0.8808	64221010	T	A	0.50	5.49E-05	3.88E-02	7.86E-02
rs12908891	Brain_Putamen_basal_ganglia	15	*TPM1*	63349472	rs11637858	0.8959	64224107	C	T	0.49	4.60E-05	1.28E-02	4.43E-02
rs12908891	Brain_Putamen_basal_ganglia	15	*RP11-321G12.1*	63705841	rs11637858	0.8959	64224107	C	T	0.49	4.60E-05	1.84E-02	5.24E-02
rs12908891	Brain_Putamen_basal_ganglia	15	*SNX22*	64446798	rs11637858	0.8959	64224107	C	T	0.49	4.60E-05	1.06E-02	4.08E-02
rs12908891	Brain_Putamen_basal_ganglia	15	*TRIP4*	64713724	rs11637858	0.8959	64224107	C	T	0.49	4.60E-05	3.43E-02	7.21E-02
rs12908891	Brain_Putamen_basal_ganglia	15	*RBPMS2*	65049938	rs11637858	0.8959	64224107	C	T	0.49	4.60E-05	4.73E-02	8.65E-02
rs12908891	Brain_Putamen_basal_ganglia	15	*TPM1*	63349472	rs12916395	0.9921	64225563	T	C	0.51	1.86E-06	2.99E-02	4.68E-02
rs12908891	Brain_Putamen_basal_ganglia	15	*SNX22*	64446798	rs12916395	0.9921	64225563	T	C	0.51	1.86E-06	6.00E-03	1.63E-02
rs12908891	Brain_Putamen_basal_ganglia	15	*TRIP4*	64713724	rs12916395	0.9921	64225563	T	C	0.51	1.86E-06	2.14E-02	3.70E-02
rs12908891	Brain_Putamen_basal_ganglia	15	*TPM1*	63349472	rs11630587	0.9921	64225908	C	T	0.51	1.77E-06	1.39E-02	2.76E-02
rs12908891	Brain_Putamen_basal_ganglia	15	*RPS27L*	63434145	rs11630587	0.9921	64225908	C	T	0.51	1.77E-06	4.31E-02	6.10E-02
rs12908891	Brain_Putamen_basal_ganglia	15	*SNX22*	64446798	rs11630587	0.9921	64225908	C	T	0.51	1.77E-06	6.52E-03	1.71E-02
rs12908891	Brain_Putamen_basal_ganglia	15	*TRIP4*	64713724	rs11630587	0.9921	64225908	C	T	0.51	1.77E-06	1.28E-02	2.62E-02
rs12908891	Brain_Putamen_basal_ganglia	15	*TPM1*	63349472	rs4776677	0.9921	64227044	C	T	0.51	1.54E-06	2.91E-02	4.57E-02
rs12908891	Brain_Putamen_basal_ganglia	15	*RPS27L*	63434145	rs4776677	0.9921	64227044	C	T	0.51	1.54E-06	4.39E-02	6.18E-02
rs12908891	Brain_Putamen_basal_ganglia	15	*CA12*	63643968	rs4776677	0.9921	64227044	C	T	0.51	1.54E-06	3.63E-02	5.36E-02
rs12908891	Brain_Putamen_basal_ganglia	15	*SNX22*	64446798	rs4776677	0.9921	64227044	C	T	0.51	1.54E-06	3.73E-03	1.22E-02
rs12908891	Brain_Putamen_basal_ganglia	15	*TRIP4*	64713724	rs4776677	0.9921	64227044	C	T	0.51	1.54E-06	2.87E-02	4.52E-02
rs12908891	Brain_Putamen_basal_ganglia	15	*CA12*	63643968	rs11071777	0.8557	64229285	C	G	0.55	9.20E-06	2.02E-02	3.79E-02
rs12908891	Brain_Putamen_basal_ganglia	15	*RP11-321G12.1*	63705841	rs11071777	0.8557	64229285	C	G	0.55	9.20E-06	2.20E-02	4.01E-02
rs12908891	Brain_Putamen_basal_ganglia	15	*SNX22*	64446798	rs11071777	0.8557	64229285	C	G	0.55	9.20E-06	1.11E-03	7.68E-03
rs12908891	Brain_Putamen_basal_ganglia	15	*TPM1*	63349472	rs1563886	0.9272	64230852	G	C	0.49	8.06E-06	1.11E-02	4.45E-02
rs12908891	Brain_Putamen_basal_ganglia	15	*RP11-321G12.1*	63705841	rs1563886	0.9272	64230852	G	C	0.49	8.06E-06	1.14E-02	4.50E-02
rs12908891	Brain_Putamen_basal_ganglia	15	*SNX22*	64446798	rs1563886	0.9272	64230852	G	C	0.49	8.06E-06	1.10E-02	4.43E-02
rs12908891	Brain_Putamen_basal_ganglia	15	*TRIP4*	64713724	rs1563886	0.9272	64230852	G	C	0.49	8.06E-06	3.50E-02	7.60E-02
rs12908891	Brain_Putamen_basal_ganglia	15	*RBPMS2*	65049938	rs1563886	0.9272	64230852	G	C	0.49	8.06E-06	3.68E-02	7.81E-02
rs12908891	Brain_Putamen_basal_ganglia	15	*TPM1*	63349472	rs10744961	0.9960	64230930	A	G	0.51	1.86E-06	2.99E-02	4.72E-02
rs12908891	Brain_Putamen_basal_ganglia	15	*SNX22*	64446798	rs10744961	0.9960	64230930	A	G	0.51	1.86E-06	6.00E-03	1.66E-02
rs12908891	Brain_Putamen_basal_ganglia	15	*TRIP4*	64713724	rs10744961	0.9960	64230930	A	G	0.51	1.86E-06	2.14E-02	3.73E-02
rs12908891	Brain_Putamen_basal_ganglia	15	*LACTB*	63424129	rs6494454	0.8031	64233563	A	C	0.56	2.52E-05	3.81E-02	6.25E-02
rs12908891	Brain_Putamen_basal_ganglia	15	*CA12*	63643968	rs6494454	0.8031	64233563	A	C	0.56	2.52E-05	3.58E-02	6.00E-02
rs12908891	Brain_Putamen_basal_ganglia	15	*TRIP4*	64713724	rs6494454	0.8031	64233563	A	C	0.56	2.52E-05	2.32E-02	4.54E-02
rs12908891	Brain_Putamen_basal_ganglia	15	*RBPMS2*	65049938	rs6494454	0.8031	64233563	A	C	0.56	2.52E-05	4.31E-02	6.80E-02
rs12908891	Brain_Putamen_basal_ganglia	15	*LACTB*	63424129	rs894660	0.8031	64234334	C	T	0.56	2.52E-05	3.81E-02	6.25E-02
rs12908891	Brain_Putamen_basal_ganglia	15	*CA12*	63643968	rs894660	0.8031	64234334	C	T	0.56	2.52E-05	3.58E-02	6.00E-02
rs12908891	Brain_Putamen_basal_ganglia	15	*TRIP4*	64713724	rs894660	0.8031	64234334	C	T	0.56	2.52E-05	2.32E-02	4.54E-02
rs12908891	Brain_Putamen_basal_ganglia	15	*RBPMS2*	65049938	rs894660	0.8031	64234334	C	T	0.56	2.52E-05	4.31E-02	6.80E-02
rs12908891	Brain_Putamen_basal_ganglia	15	*LACTB*	63424129	rs4776266	0.8031	64236175	G	T	0.56	2.23E-05	3.75E-02	6.17E-02

rs12908891	Brain_Putamen_basal_ganglia	15	*CA12*	63643968	rs4776266	0.8031	64236175	G	T	0.56	2.23E-05	2.70E-02	4.97E-02
rs12908891	Brain_Putamen_basal_ganglia	15	*TRIP4*	64713724	rs4776266	0.8031	64236175	G	T	0.56	2.23E-05	2.88E-02	5.19E-02
rs12908891	Brain_Putamen_basal_ganglia	15	*TPM1*	63349472	rs4776268	1.0000	64236398	A	G	0.51	6.19E-06	1.27E-02	6.27E-02
rs12908891	Brain_Putamen_basal_ganglia	15	*RP11-321G12.1*	63705841	rs4776268	1.0000	64236398	A	G	0.51	6.19E-06	2.35E-02	7.83E-02
rs12908891	Brain_Putamen_basal_ganglia	15	*SNX22*	64446798	rs4776268	1.0000	64236398	A	G	0.51	6.19E-06	7.91E-03	5.41E-02
rs12908891	Brain_Putamen_basal_ganglia	15	*TRIP4*	64713724	rs4776268	1.0000	64236398	A	G	0.51	6.19E-06	2.33E-02	7.81E-02
rs12908891	Brain_Putamen_basal_ganglia	15	*RBPMS2*	65049938	rs4776268	1.0000	64236398	A	G	0.51	6.19E-06	3.98E-02	9.76E-02
rs12908891	Brain_Putamen_basal_ganglia	15	*TPM1*	63349472	rs12908891	1.0000	64236441	A	G	0.52	1.39E-06	3.55E-02	5.29E-02
rs12908891	Brain_Putamen_basal_ganglia	15	*SNX22*	64446798	rs12908891	1.0000	64236441	A	G	0.52	1.39E-06	4.62E-03	1.39E-02
rs12908891	Brain_Putamen_basal_ganglia	15	*TRIP4*	64713724	rs12908891	1.0000	64236441	A	G	0.52	1.39E-06	2.45E-02	4.06E-02
rs12908891	Brain_Putamen_basal_ganglia	15	*RBPMS2*	65049938	rs12908891	1.0000	64236441	A	G	0.52	1.39E-06	4.15E-02	5.94E-02
rs12908891	Brain_Putamen_basal_ganglia	15	*TPM1*	63349472	rs11071779	0.9881	64239044	G	A	0.51	1.57E-06	2.43E-02	4.09E-02
rs12908891	Brain_Putamen_basal_ganglia	15	*CA12*	63643968	rs11071779	0.9881	64239044	G	A	0.51	1.57E-06	3.68E-02	5.50E-02
rs12908891	Brain_Putamen_basal_ganglia	15	*RP11-321G12.1*	63705841	rs11071779	0.9881	64239044	G	A	0.51	1.57E-06	3.48E-02	5.28E-02
rs12908891	Brain_Putamen_basal_ganglia	15	*FBXL22*	63892089	rs11071779	0.9881	64239044	G	A	0.51	1.57E-06	4.28E-02	6.14E-02
rs12908891	Brain_Putamen_basal_ganglia	15	*SNX22*	64446798	rs11071779	0.9881	64239044	G	A	0.51	1.57E-06	4.08E-03	1.34E-02
rs12908891	Brain_Putamen_basal_ganglia	15	*TRIP4*	64713724	rs11071779	0.9881	64239044	G	A	0.51	1.57E-06	1.90E-02	3.45E-02
rs12908891	Brain_Putamen_basal_ganglia	15	*TPM1*	63349472	rs11071780	0.9842	64241691	A	G	0.51	4.74E-06	2.32E-02	8.06E-02
rs12908891	Brain_Putamen_basal_ganglia	15	*CA12*	63643968	rs11071780	0.9842	64241691	A	G	0.51	4.74E-06	4.42E-02	1.05E-01
rs12908891	Brain_Putamen_basal_ganglia	15	*RP11-321G12.1*	63705841	rs11071780	0.9842	64241691	A	G	0.51	4.74E-06	1.88E-02	7.46E-02
rs12908891	Brain_Putamen_basal_ganglia	15	*SNX22*	64446798	rs11071780	0.9842	64241691	A	G	0.51	4.74E-06	4.52E-03	4.87E-02
rs12908891	Brain_Putamen_basal_ganglia	15	*TRIP4*	64713724	rs11071780	0.9842	64241691	A	G	0.51	4.74E-06	2.65E-02	8.47E-02
rs12908891	Brain_Putamen_basal_ganglia	15	*RBPMS2*	65049938	rs11071780	0.9842	64241691	A	G	0.51	4.74E-06	4.95E-02	1.10E-01
rs12908891	Brain_Putamen_basal_ganglia	15	*TPM1*	63349472	rs11854537	0.9842	64241733	A	G	0.51	4.74E-06	2.32E-02	8.06E-02
rs12908891	Brain_Putamen_basal_ganglia	15	*CA12*	63643968	rs11854537	0.9842	64241733	A	G	0.51	4.74E-06	4.42E-02	1.05E-01
rs12908891	Brain_Putamen_basal_ganglia	15	*RP11-321G12.1*	63705841	rs11854537	0.9842	64241733	A	G	0.51	4.74E-06	1.88E-02	7.46E-02
rs12908891	Brain_Putamen_basal_ganglia	15	*SNX22*	64446798	rs11854537	0.9842	64241733	A	G	0.51	4.74E-06	4.52E-03	4.87E-02
rs12908891	Brain_Putamen_basal_ganglia	15	*TRIP4*	64713724	rs11854537	0.9842	64241733	A	G	0.51	4.74E-06	2.65E-02	8.47E-02
rs12908891	Brain_Putamen_basal_ganglia	15	*RBPMS2*	65049938	rs11854537	0.9842	64241733	A	G	0.51	4.74E-06	4.95E-02	1.10E-01
rs12908891	Brain_Putamen_basal_ganglia	15	*TPM1*	63349472	rs1380844	0.9881	64242007	G	T	0.51	4.74E-06	2.32E-02	8.06E-02
rs12908891	Brain_Putamen_basal_ganglia	15	*CA12*	63643968	rs1380844	0.9881	64242007	G	T	0.51	4.74E-06	4.42E-02	1.05E-01
rs12908891	Brain_Putamen_basal_ganglia	15	*RP11-321G12.1*	63705841	rs1380844	0.9881	64242007	G	T	0.51	4.74E-06	1.88E-02	7.46E-02
rs12908891	Brain_Putamen_basal_ganglia	15	*SNX22*	64446798	rs1380844	0.9881	64242007	G	T	0.51	4.74E-06	4.52E-03	4.87E-02
rs12908891	Brain_Putamen_basal_ganglia	15	*TRIP4*	64713724	rs1380844	0.9881	64242007	G	T	0.51	4.74E-06	2.65E-02	8.47E-02
rs12908891	Brain_Putamen_basal_ganglia	15	*RBPMS2*	65049938	rs1380844	0.9881	64242007	G	T	0.51	4.74E-06	4.95E-02	1.10E-01
rs12908891	Brain_Putamen_basal_ganglia	15	*TPM1*	63349472	rs12916806	0.9881	64242770	C	A	0.51	4.64E-06	2.32E-02	8.05E-02
rs12908891	Brain_Putamen_basal_ganglia	15	*CA12*	63643968	rs12916806	0.9881	64242770	C	A	0.51	4.64E-06	4.42E-02	1.05E-01
rs12908891	Brain_Putamen_basal_ganglia	15	*RP11-321G12.1*	63705841	rs12916806	0.9881	64242770	C	A	0.51	4.64E-06	1.88E-02	7.45E-02
rs12908891	Brain_Putamen_basal_ganglia	15	*SNX22*	64446798	rs12916806	0.9881	64242770	C	A	0.51	4.64E-06	4.52E-03	4.87E-02
rs12908891	Brain_Putamen_basal_ganglia	15	*TRIP4*	64713724	rs12916806	0.9881	64242770	C	A	0.51	4.64E-06	2.65E-02	8.47E-02

rs12908891	Brain_Putamen_basal_ganglia	15	*RBPMS2*	65049938	rs12916806	0.9881	64242770	C	A	0.51	4.64E-06	4.95E-02	1.10E-01
rs12908891	Brain_Putamen_basal_ganglia	15	*TPM1*	63349472	rs11071781	0.9195	64243933	G	A	0.49	4.75E-06	2.11E-02	6.05E-02
rs12908891	Brain_Putamen_basal_ganglia	15	*RPS27L*	63434145	rs11071781	0.9195	64243933	G	A	0.49	4.75E-06	4.41E-02	8.75E-02
rs12908891	Brain_Putamen_basal_ganglia	15	*RP11-321G12.1*	63705841	rs11071781	0.9195	64243933	G	A	0.49	4.75E-06	1.89E-02	5.76E-02
rs12908891	Brain_Putamen_basal_ganglia	15	*SNX22*	64446798	rs11071781	0.9195	64243933	G	A	0.49	4.75E-06	4.99E-03	3.41E-02
rs12908891	Brain_Putamen_basal_ganglia	15	*TRIP4*	64713724	rs11071781	0.9195	64243933	G	A	0.49	4.75E-06	3.24E-02	7.45E-02
rs12908891	Brain_Putamen_basal_ganglia	15	*TPM1*	63349472	rs1304365	0.9881	64245946	A	G	0.51	4.67E-06	2.00E-02	7.62E-02
rs12908891	Brain_Putamen_basal_ganglia	15	*CA12*	63643968	rs1304365	0.9881	64245946	A	G	0.51	4.67E-06	4.61E-02	1.07E-01
rs12908891	Brain_Putamen_basal_ganglia	15	*RP11-321G12.1*	63705841	rs1304365	0.9881	64245946	A	G	0.51	4.67E-06	2.34E-02	8.08E-02
rs12908891	Brain_Putamen_basal_ganglia	15	*SNX22*	64446798	rs1304365	0.9881	64245946	A	G	0.51	4.67E-06	3.58E-03	4.61E-02
rs12908891	Brain_Putamen_basal_ganglia	15	*TRIP4*	64713724	rs1304365	0.9881	64245946	A	G	0.51	4.67E-06	2.15E-02	7.83E-02
rs12908891	Brain_Putamen_basal_ganglia	15	*TPM1*	63349472	rs115415409	0.8420	64249559	T	C	0.45	1.45E-05	4.25E-02	6.98E-02
rs12908891	Brain_Putamen_basal_ganglia	15	*RP11-321G12.1*	63705841	rs115415409	0.8420	64249559	T	C	0.45	1.45E-05	1.66E-02	3.93E-02
rs12908891	Brain_Putamen_basal_ganglia	15	*SNX22*	64446798	rs115415409	0.8420	64249559	T	C	0.45	1.45E-05	2.74E-02	5.28E-02
rs12908891	Brain_Putamen_basal_ganglia	15	*TPM1*	63349472	rs145159458	0.9119	64252880	T	C	0.49	1.21E-05	1.79E-02	6.68E-02
rs12908891	Brain_Putamen_basal_ganglia	15	*CA12*	63643968	rs145159458	0.9119	64252880	T	C	0.49	1.21E-05	4.85E-02	1.03E-01
rs12908891	Brain_Putamen_basal_ganglia	15	*RP11-321G12.1*	63705841	rs145159458	0.9119	64252880	T	C	0.49	1.21E-05	1.15E-02	5.68E-02
rs12908891	Brain_Putamen_basal_ganglia	15	*SNX22*	64446798	rs145159458	0.9119	64252880	T	C	0.49	1.21E-05	5.07E-03	4.40E-02
rs12908891	Brain_Putamen_basal_ganglia	15	*TRIP4*	64713724	rs145159458	0.9119	64252880	T	C	0.49	1.21E-05	3.24E-02	8.53E-02
rs12908891	Brain_Putamen_basal_ganglia	15	*TPM1*	63349472	rs4776692	0.9881	64255083	G	A	0.50	1.06E-05	2.32E-02	8.99E-02
rs12908891	Brain_Putamen_basal_ganglia	15	*CA12*	63643968	rs4776692	0.9881	64255083	G	A	0.50	1.06E-05	4.42E-02	1.14E-01
rs12908891	Brain_Putamen_basal_ganglia	15	*RP11-321G12.1*	63705841	rs4776692	0.9881	64255083	G	A	0.50	1.06E-05	1.88E-02	8.39E-02
rs12908891	Brain_Putamen_basal_ganglia	15	*SNX22*	64446798	rs4776692	0.9881	64255083	G	A	0.50	1.06E-05	4.52E-03	5.77E-02
rs12908891	Brain_Putamen_basal_ganglia	15	*TRIP4*	64713724	rs4776692	0.9881	64255083	G	A	0.50	1.06E-05	2.65E-02	9.40E-02
rs12908891	Brain_Putamen_basal_ganglia	15	*RBPMS2*	65049938	rs4776692	0.9881	64255083	G	A	0.50	1.06E-05	4.95E-02	1.20E-01
rs12908891	Brain_Putamen_basal_ganglia	15	*TPM1*	63349472	rs28822416	0.8420	64257845	C	T	0.45	1.54E-05	3.98E-02	6.71E-02
rs12908891	Brain_Putamen_basal_ganglia	15	*RP11-321G12.1*	63705841	rs28822416	0.8420	64257845	C	T	0.45	1.54E-05	1.68E-02	3.98E-02
rs12908891	Brain_Putamen_basal_ganglia	15	*SNX22*	64446798	rs28822416	0.8420	64257845	C	T	0.45	1.54E-05	2.36E-02	4.85E-02
rs12908891	Brain_Putamen_basal_ganglia	15	*RBPMS2*	65049938	rs28822416	0.8420	64257845	C	T	0.45	1.54E-05	4.31E-02	7.07E-02
rs12908891	Brain_Putamen_basal_ganglia	15	*TPM1*	63349472	rs8035776	0.8420	64258018	G	A	0.45	1.54E-05	3.98E-02	6.71E-02
rs12908891	Brain_Putamen_basal_ganglia	15	*RP11-321G12.1*	63705841	rs8035776	0.8420	64258018	G	A	0.45	1.54E-05	1.68E-02	3.98E-02
rs12908891	Brain_Putamen_basal_ganglia	15	*SNX22*	64446798	rs8035776	0.8420	64258018	G	A	0.45	1.54E-05	2.36E-02	4.85E-02
rs12908891	Brain_Putamen_basal_ganglia	15	*RBPMS2*	65049938	rs8035776	0.8420	64258018	G	A	0.45	1.54E-05	4.31E-02	7.07E-02
rs12908891	Brain_Putamen_basal_ganglia	15	*TPM1*	63349472	rs6494456	0.8381	64258260	C	T	0.45	1.54E-05	3.98E-02	6.71E-02
rs12908891	Brain_Putamen_basal_ganglia	15	*RP11-321G12.1*	63705841	rs6494456	0.8381	64258260	C	T	0.45	1.54E-05	1.68E-02	3.98E-02
rs12908891	Brain_Putamen_basal_ganglia	15	*SNX22*	64446798	rs6494456	0.8381	64258260	C	T	0.45	1.54E-05	2.36E-02	4.85E-02
rs12908891	Brain_Putamen_basal_ganglia	15	*RBPMS2*	65049938	rs6494456	0.8381	64258260	C	T	0.45	1.54E-05	4.31E-02	7.07E-02
rs12908891	Brain_Putamen_basal_ganglia	15	*TPM1*	63349472	rs4776695	0.8350	64259115	T	C	0.46	9.12E-06	7.89E-03	2.41E-02
rs12908891	Brain_Putamen_basal_ganglia	15	*RPS27L*	63434145	rs4776695	0.8350	64259115	T	C	0.46	9.12E-06	2.70E-02	4.96E-02
rs12908891	Brain_Putamen_basal_ganglia	15	*RP11-321G12.1*	63705841	rs4776695	0.8350	64259115	T	C	0.46	9.12E-06	4.12E-02	6.54E-02

rs12908891	Brain_Putamen_basal_ganglia	15	*SNX22*	64446798	rs4776695	0.8350	64259115	T	C	0.46	9.12E-06	1.18E-02	3.00E-02
rs12908891	Brain_Putamen_basal_ganglia	15	*ANKDD1A*	65227571	rs4776695	0.8350	64259115	T	C	0.46	9.12E-06	4.14E-02	6.57E-02
rs12908891	Brain_Spinal_cord_cervical_c-1	15	*APH1B*	63584771	rs1460544	0.8736	64201766	T	C	0.51	9.06E-04	3.33E-02	1.51E-01
rs12908891	Brain_Spinal_cord_cervical_c-1	15	*APH1B*	63584771	rs1037846	0.8696	64204870	G	A	0.51	6.33E-04	2.72E-02	5.78E-02
rs12908891	Brain_Spinal_cord_cervical_c-1	15	*FBXL22*	63892089	rs1037846	0.8696	64204870	G	A	0.51	6.33E-04	4.70E-02	8.00E-02
rs12908891	Brain_Spinal_cord_cervical_c-1	15	*APH1B*	63584771	rs11071773	0.8883	64206765	A	G	0.51	8.95E-04	3.88E-02	1.30E-01
rs12908891	Brain_Spinal_cord_cervical_c-1	15	*APH1B*	63584771	rs12909081	0.8809	64208260	A	C	0.51	1.00E-03	4.40E-02	1.40E-01
rs12908891	Brain_Spinal_cord_cervical_c-1	15	*APH1B*	63584771	rs749468	0.9493	64210279	T	C	0.53	2.79E-04	4.48E-02	7.70E-02
rs12908891	Brain_Spinal_cord_cervical_c-1	15	*FBXL22*	63892089	rs749468	0.9493	64210279	T	C	0.53	2.79E-04	3.18E-02	6.27E-02
rs12908891	Brain_Spinal_cord_cervical_c-1	15	*APH1B*	63584771	rs2414844	0.8846	64210675	A	G	0.51	9.01E-04	4.40E-02	1.37E-01
rs12908891	Brain_Spinal_cord_cervical_c-1	15	*APH1B*	63584771	rs12592060	0.8846	64210780	T	C	0.51	9.01E-04	4.40E-02	1.37E-01
rs12908891	Brain_Spinal_cord_cervical_c-1	15	*APH1B*	63584771	rs1471282	0.8846	64212275	G	T	0.51	1.07E-03	4.40E-02	1.43E-01
rs12908891	Brain_Spinal_cord_cervical_c-1	15	*APH1B*	63584771	rs11635779	0.8808	64213826	T	C	0.50	6.34E-05	4.40E-02	8.60E-02
rs12908891	Brain_Spinal_cord_cervical_c-1	15	*APH1B*	63584771	rs12904374	0.8808	64214670	T	A	0.51	8.63E-04	3.88E-02	7.38E-02
rs12908891	Brain_Spinal_cord_cervical_c-1	15	*APH1B*	63584771	rs920762	0.9379	64214954	C	G	0.47	1.24E-05	2.24E-02	4.05E-02
rs12908891	Brain_Spinal_cord_cervical_c-1	15	*FBXL22*	63892089	rs920762	0.9379	64214954	C	G	0.47	1.24E-05	3.26E-02	5.22E-02
rs12908891	Brain_Spinal_cord_cervical_c-1	15	*APH1B*	63584771	rs11853632	0.8921	64217845	C	G	0.50	4.21E-05	3.34E-02	7.11E-02
rs12908891	Brain_Spinal_cord_cervical_c-1	15	*FBXL22*	63892089	rs11853632	0.8921	64217845	C	G	0.50	4.21E-05	4.27E-02	8.16E-02
rs12908891	Brain_Spinal_cord_cervical_c-1	15	*APH1B*	63584771	rs12907405	0.8884	64220046	T	C	0.50	4.35E-05	4.40E-02	8.32E-02
rs12908891	Brain_Spinal_cord_cervical_c-1	15	*APH1B*	63584771	rs11071775	0.8808	64220995	A	G	0.50	4.81E-05	4.23E-02	8.15E-02
rs12908891	Brain_Spinal_cord_cervical_c-1	15	*FBXL22*	63892089	rs11071775	0.8808	64220995	A	G	0.50	4.81E-05	2.57E-02	6.23E-02
rs12908891	Brain_Spinal_cord_cervical_c-1	15	*CSNK1G1*	64553079	rs11071775	0.8808	64220995	A	G	0.50	4.81E-05	4.23E-02	8.14E-02
rs12908891	Brain_Spinal_cord_cervical_c-1	15	*APH1B*	63584771	rs11071776	0.8808	64221010	T	A	0.50	5.49E-05	4.23E-02	8.24E-02
rs12908891	Brain_Spinal_cord_cervical_c-1	15	*FBXL22*	63892089	rs11071776	0.8808	64221010	T	A	0.50	5.49E-05	2.57E-02	6.33E-02
rs12908891	Brain_Spinal_cord_cervical_c-1	15	*CSNK1G1*	64553079	rs11071776	0.8808	64221010	T	A	0.50	5.49E-05	4.23E-02	8.24E-02
rs12908891	Brain_Spinal_cord_cervical_c-1	15	*FBXL22*	63892089	rs11637858	0.8959	64224107	C	T	0.49	4.60E-05	3.56E-02	7.36E-02
rs12908891	Brain_Spinal_cord_cervical_c-1	15	*CSNK1G1*	64553079	rs11637858	0.8959	64224107	C	T	0.49	4.60E-05	3.33E-02	7.10E-02
rs12908891	Brain_Spinal_cord_cervical_c-1	15	*FBXL22*	63892089	rs12916395	0.9921	64225563	T	C	0.51	1.86E-06	2.61E-02	4.24E-02
rs12908891	Brain_Spinal_cord_cervical_c-1	15	*FBXL22*	63892089	rs11630587	0.9921	64225908	C	T	0.51	1.77E-06	2.61E-02	4.23E-02
rs12908891	Brain_Spinal_cord_cervical_c-1	15	*RAB8B*	63520824	rs4776677	0.9921	64227044	C	T	0.51	1.54E-06	3.83E-02	5.58E-02
rs12908891	Brain_Spinal_cord_cervical_c-1	15	*FBXL22*	63892089	rs4776677	0.9921	64227044	C	T	0.51	1.54E-06	2.46E-02	4.05E-02
rs12908891	Brain_Spinal_cord_cervical_c-1	15	*TRIP4*	64713724	rs4776677	0.9921	64227044	C	T	0.51	1.54E-06	4.65E-02	6.46E-02
rs12908891	Brain_Spinal_cord_cervical_c-1	15	*APH1B*	63584771	rs11071777	0.8557	64229285	C	G	0.55	9.20E-06	4.29E-02	6.35E-02
rs12908891	Brain_Spinal_cord_cervical_c-1	15	*FBXL22*	63892089	rs11071777	0.8557	64229285	C	G	0.55	9.20E-06	1.77E-02	3.48E-02
rs12908891	Brain_Spinal_cord_cervical_c-1	15	*FBXL22*	63892089	rs1563886	0.9272	64230852	G	C	0.49	8.06E-06	1.64E-02	5.27E-02
rs12908891	Brain_Spinal_cord_cervical_c-1	15	*FBXL22*	63892089	rs10744961	0.9960	64230930	A	G	0.51	1.86E-06	2.61E-02	4.28E-02
rs12908891	Brain_Spinal_cord_cervical_c-1	15	*FBXL22*	63892089	rs6494454	0.8031	64233563	A	C	0.56	2.52E-05	2.37E-02	4.60E-02
rs12908891	Brain_Spinal_cord_cervical_c-1	15	*RP11-111E14.1*	64224312	rs6494454	0.8031	64233563	A	C	0.56	2.52E-05	1.56E-02	3.57E-02
rs12908891	Brain_Spinal_cord_cervical_c-1	15	*FBXL22*	63892089	rs894660	0.8031	64234334	C	T	0.56	2.52E-05	2.37E-02	4.60E-02
rs12908891	Brain_Spinal_cord_cervical_c-1	15	*RP11-111E14.1*	64224312	rs894660	0.8031	64234334	C	T	0.56	2.52E-05	1.56E-02	3.57E-02

rs12908891	Brain_Spinal_cord_cervical_c-1	15	*FBXL22*	63892089	rs4776266	0.8031	64236175	G	T	0.56	2.23E-05	2.04E-02	4.18E-02
rs12908891	Brain_Spinal_cord_cervical_c-1	15	*RP11-111E14.1*	64224312	rs4776266	0.8031	64236175	G	T	0.56	2.23E-05	2.42E-02	4.64E-02
rs12908891	Brain_Spinal_cord_cervical_c-1	15	*FBXL22*	63892089	rs4776268	1.0000	64236398	A	G	0.51	6.19E-06	1.62E-02	6.83E-02
rs12908891	Brain_Spinal_cord_cervical_c-1	15	*RAB8B*	63520824	rs12908891	1.0000	64236441	A	G	0.52	1.39E-06	4.81E-02	6.64E-02
rs12908891	Brain_Spinal_cord_cervical_c-1	15	*FBXL22*	63892089	rs12908891	1.0000	64236441	A	G	0.52	1.39E-06	1.29E-02	2.63E-02
rs12908891	Brain_Spinal_cord_cervical_c-1	15	*TRIP4*	64713724	rs12908891	1.0000	64236441	A	G	0.52	1.39E-06	4.60E-02	6.42E-02
rs12908891	Brain_Spinal_cord_cervical_c-1	15	*FBXL22*	63892089	rs11071779	0.9881	64239044	G	A	0.51	1.57E-06	2.75E-02	4.46E-02
rs12908891	Brain_Spinal_cord_cervical_c-1	15	*RP11-111E14.1*	64224312	rs11071779	0.9881	64239044	G	A	0.51	1.57E-06	2.96E-02	4.70E-02
rs12908891	Brain_Spinal_cord_cervical_c-1	15	*APH1B*	63584771	rs11071780	0.9842	64241691	A	G	0.51	4.74E-06	4.99E-02	1.11E-01
rs12908891	Brain_Spinal_cord_cervical_c-1	15	*FBXL22*	63892089	rs11071780	0.9842	64241691	A	G	0.51	4.74E-06	2.42E-02	8.18E-02
rs12908891	Brain_Spinal_cord_cervical_c-1	15	*RP11-111E14.1*	64224312	rs11071780	0.9842	64241691	A	G	0.51	4.74E-06	3.41E-02	9.38E-02
rs12908891	Brain_Spinal_cord_cervical_c-1	15	*APH1B*	63584771	rs11854537	0.9842	64241733	A	G	0.51	4.74E-06	4.99E-02	1.11E-01
rs12908891	Brain_Spinal_cord_cervical_c-1	15	*FBXL22*	63892089	rs11854537	0.9842	64241733	A	G	0.51	4.74E-06	2.42E-02	8.18E-02
rs12908891	Brain_Spinal_cord_cervical_c-1	15	*RP11-111E14.1*	64224312	rs11854537	0.9842	64241733	A	G	0.51	4.74E-06	3.41E-02	9.38E-02
rs12908891	Brain_Spinal_cord_cervical_c-1	15	*APH1B*	63584771	rs1380844	0.9881	64242007	G	T	0.51	4.74E-06	4.99E-02	1.11E-01
rs12908891	Brain_Spinal_cord_cervical_c-1	15	*FBXL22*	63892089	rs1380844	0.9881	64242007	G	T	0.51	4.74E-06	2.42E-02	8.18E-02
rs12908891	Brain_Spinal_cord_cervical_c-1	15	*RP11-111E14.1*	64224312	rs1380844	0.9881	64242007	G	T	0.51	4.74E-06	3.41E-02	9.38E-02
rs12908891	Brain_Spinal_cord_cervical_c-1	15	*APH1B*	63584771	rs12916806	0.9881	64242770	C	A	0.51	4.64E-06	4.99E-02	1.11E-01
rs12908891	Brain_Spinal_cord_cervical_c-1	15	*FBXL22*	63892089	rs12916806	0.9881	64242770	C	A	0.51	4.64E-06	2.42E-02	8.18E-02
rs12908891	Brain_Spinal_cord_cervical_c-1	15	*RP11-111E14.1*	64224312	rs12916806	0.9881	64242770	C	A	0.51	4.64E-06	3.41E-02	9.37E-02
rs12908891	Brain_Spinal_cord_cervical_c-1	15	*APH1B*	63584771	rs11071781	0.9195	64243933	G	A	0.49	4.75E-06	4.98E-02	9.36E-02
rs12908891	Brain_Spinal_cord_cervical_c-1	15	*APH1B*	63584771	rs1304365	0.9881	64245946	A	G	0.51	4.67E-06	4.99E-02	1.11E-01
rs12908891	Brain_Spinal_cord_cervical_c-1	15	*FBXL22*	63892089	rs1304365	0.9881	64245946	A	G	0.51	4.67E-06	2.42E-02	8.19E-02
rs12908891	Brain_Spinal_cord_cervical_c-1	15	*RP11-111E14.1*	64224312	rs1304365	0.9881	64245946	A	G	0.51	4.67E-06	3.41E-02	9.38E-02
rs12908891	Brain_Spinal_cord_cervical_c-1	15	*APH1B*	63584771	rs115415409	0.8420	64249559	T	C	0.45	1.45E-05	1.56E-02	3.80E-02
rs12908891	Brain_Spinal_cord_cervical_c-1	15	*FBXL22*	63892089	rs115415409	0.8420	64249559	T	C	0.45	1.45E-05	3.36E-02	6.00E-02
rs12908891	Brain_Spinal_cord_cervical_c-1	15	*FBXL22*	63892089	rs145159458	0.9119	64252880	T	C	0.49	1.21E-05	2.45E-02	7.56E-02
rs12908891	Brain_Spinal_cord_cervical_c-1	15	*RP11-111E14.1*	64224312	rs145159458	0.9119	64252880	T	C	0.49	1.21E-05	4.84E-02	1.03E-01
rs12908891	Brain_Spinal_cord_cervical_c-1	15	*APH1B*	63584771	rs4776692	0.9881	64255083	G	A	0.50	1.06E-05	4.99E-02	1.20E-01
rs12908891	Brain_Spinal_cord_cervical_c-1	15	*FBXL22*	63892089	rs4776692	0.9881	64255083	G	A	0.50	1.06E-05	2.42E-02	9.11E-02
rs12908891	Brain_Spinal_cord_cervical_c-1	15	*RP11-111E14.1*	64224312	rs4776692	0.9881	64255083	G	A	0.50	1.06E-05	3.41E-02	1.03E-01
rs12908891	Brain_Spinal_cord_cervical_c-1	15	*APH1B*	63584771	rs28822416	0.8420	64257845	C	T	0.45	1.54E-05	1.76E-02	4.08E-02
rs12908891	Brain_Spinal_cord_cervical_c-1	15	*FBXL22*	63892089	rs28822416	0.8420	64257845	C	T	0.45	1.54E-05	2.29E-02	4.77E-02
rs12908891	Brain_Spinal_cord_cervical_c-1	15	*APH1B*	63584771	rs8035776	0.8420	64258018	G	A	0.45	1.54E-05	1.76E-02	4.08E-02
rs12908891	Brain_Spinal_cord_cervical_c-1	15	*FBXL22*	63892089	rs8035776	0.8420	64258018	G	A	0.45	1.54E-05	2.29E-02	4.77E-02
rs12908891	Brain_Spinal_cord_cervical_c-1	15	*APH1B*	63584771	rs6494456	0.8381	64258260	C	T	0.45	1.54E-05	1.76E-02	4.08E-02
rs12908891	Brain_Spinal_cord_cervical_c-1	15	*FBXL22*	63892089	rs6494456	0.8381	64258260	C	T	0.45	1.54E-05	2.29E-02	4.77E-02
rs12908891	Brain_Spinal_cord_cervical_c-1	15	*APH1B*	63584771	rs4776695	0.8350	64259115	T	C	0.46	9.12E-06	3.44E-02	5.80E-02
rs12908891	Brain_Spinal_cord_cervical_c-1	15	*FBXL22*	63892089	rs4776695	0.8350	64259115	T	C	0.46	9.12E-06	4.93E-02	7.41E-02
rs12908891	Brain_Spinal_cord_cervical_c-1	15	*RP11-111E14.1*	64224312	rs4776695	0.8350	64259115	T	C	0.46	9.12E-06	4.54E-02	7.00E-02

rs12908891	Brain_Substantia_nigra	15	*FBXL22*	63892089	rs1460544	0.8736	64201766	T	C	0.51	9.06E-04	9.01E-03	1.19E-01
rs12908891	Brain_Substantia_nigra	15	*CA12*	63643968	rs1037846	0.8696	64204870	G	A	0.51	6.33E-04	3.22E-02	6.38E-02
rs12908891	Brain_Substantia_nigra	15	*FBXL22*	63892089	rs1037846	0.8696	64204870	G	A	0.51	6.33E-04	2.22E-02	5.17E-02
rs12908891	Brain_Substantia_nigra	15	*ZNF609*	64865602	rs1037846	0.8696	64204870	G	A	0.51	6.33E-04	3.73E-02	6.95E-02
rs12908891	Brain_Substantia_nigra	15	*FBXL22*	63892089	rs11071773	0.8883	64206765	A	G	0.51	8.95E-04	6.97E-03	8.65E-02
rs12908891	Brain_Substantia_nigra	15	*ZNF609*	64865602	rs11071773	0.8883	64206765	A	G	0.51	8.95E-04	3.87E-02	1.30E-01
rs12908891	Brain_Substantia_nigra	15	*CA12*	63643968	rs12909081	0.8809	64208260	A	C	0.51	1.00E-03	4.53E-02	1.41E-01
rs12908891	Brain_Substantia_nigra	15	*FBXL22*	63892089	rs12909081	0.8809	64208260	A	C	0.51	1.00E-03	6.35E-03	8.90E-02
rs12908891	Brain_Substantia_nigra	15	*FBXL22*	63892089	rs749468	0.9493	64210279	T	C	0.53	2.79E-04	3.28E-03	2.09E-02
rs12908891	Brain_Substantia_nigra	15	*CA12*	63643968	rs2414844	0.8846	64210675	A	G	0.51	9.01E-04	4.53E-02	1.38E-01
rs12908891	Brain_Substantia_nigra	15	*FBXL22*	63892089	rs2414844	0.8846	64210675	A	G	0.51	9.01E-04	6.35E-03	8.59E-02
rs12908891	Brain_Substantia_nigra	15	*CA12*	63643968	rs12592060	0.8846	64210780	T	C	0.51	9.01E-04	4.53E-02	1.38E-01
rs12908891	Brain_Substantia_nigra	15	*FBXL22*	63892089	rs12592060	0.8846	64210780	T	C	0.51	9.01E-04	6.35E-03	8.59E-02
rs12908891	Brain_Substantia_nigra	15	*CA12*	63643968	rs1471282	0.8846	64212275	G	T	0.51	1.07E-03	4.53E-02	1.44E-01
rs12908891	Brain_Substantia_nigra	15	*FBXL22*	63892089	rs1471282	0.8846	64212275	G	T	0.51	1.07E-03	6.35E-03	9.24E-02
rs12908891	Brain_Substantia_nigra	15	*CA12*	63643968	rs11635779	0.8808	64213826	T	C	0.50	6.34E-05	3.07E-02	7.11E-02
rs12908891	Brain_Substantia_nigra	15	*FBXL22*	63892089	rs11635779	0.8808	64213826	T	C	0.50	6.34E-05	4.27E-03	3.12E-02
rs12908891	Brain_Substantia_nigra	15	*CSNK1G1*	64553079	rs11635779	0.8808	64213826	T	C	0.50	6.34E-05	4.31E-02	8.51E-02
rs12908891	Brain_Substantia_nigra	15	*CA12*	63643968	rs12904374	0.8808	64214670	T	A	0.51	8.63E-04	4.00E-02	7.52E-02
rs12908891	Brain_Substantia_nigra	15	*FBXL22*	63892089	rs12904374	0.8808	64214670	T	A	0.51	8.63E-04	4.51E-03	2.64E-02
rs12908891	Brain_Substantia_nigra	15	*CA12*	63643968	rs920762	0.9379	64214954	C	G	0.47	1.24E-05	4.14E-02	6.19E-02
rs12908891	Brain_Substantia_nigra	15	*FBXL22*	63892089	rs920762	0.9379	64214954	C	G	0.47	1.24E-05	1.61E-02	3.27E-02
rs12908891	Brain_Substantia_nigra	15	*ZNF609*	64865602	rs920762	0.9379	64214954	C	G	0.47	1.24E-05	4.62E-02	6.71E-02
rs12908891	Brain_Substantia_nigra	15	*CA12*	63643968	rs11853632	0.8921	64217845	C	G	0.50	4.21E-05	3.51E-02	7.31E-02
rs12908891	Brain_Substantia_nigra	15	*FBXL22*	63892089	rs11853632	0.8921	64217845	C	G	0.50	4.21E-05	1.16E-02	4.26E-02
rs12908891	Brain_Substantia_nigra	15	*CSNK1G1*	64553079	rs11853632	0.8921	64217845	C	G	0.50	4.21E-05	2.84E-02	6.52E-02
rs12908891	Brain_Substantia_nigra	15	*ZNF609*	64865602	rs11853632	0.8921	64217845	C	G	0.50	4.21E-05	3.37E-02	7.15E-02
rs12908891	Brain_Substantia_nigra	15	*CA12*	63643968	rs12907405	0.8884	64220046	T	C	0.50	4.35E-05	3.10E-02	6.87E-02
rs12908891	Brain_Substantia_nigra	15	*FBXL22*	63892089	rs12907405	0.8884	64220046	T	C	0.50	4.35E-05	5.55E-03	3.17E-02
rs12908891	Brain_Substantia_nigra	15	*CA12*	63643968	rs11071775	0.8808	64220995	A	G	0.50	4.81E-05	3.80E-02	7.67E-02
rs12908891	Brain_Substantia_nigra	15	*FBXL22*	63892089	rs11071775	0.8808	64220995	A	G	0.50	4.81E-05	1.07E-02	4.13E-02
rs12908891	Brain_Substantia_nigra	15	*CA12*	63643968	rs11071776	0.8808	64221010	T	A	0.50	5.49E-05	3.80E-02	7.77E-02
rs12908891	Brain_Substantia_nigra	15	*FBXL22*	63892089	rs11071776	0.8808	64221010	T	A	0.50	5.49E-05	1.07E-02	4.22E-02
rs12908891	Brain_Substantia_nigra	15	*CA12*	63643968	rs11637858	0.8959	64224107	C	T	0.49	4.60E-05	3.10E-02	6.84E-02
rs12908891	Brain_Substantia_nigra	15	*FBXL22*	63892089	rs11637858	0.8959	64224107	C	T	0.49	4.60E-05	5.55E-03	3.14E-02
rs12908891	Brain_Substantia_nigra	15	*CA12*	63643968	rs12916395	0.9921	64225563	T	C	0.51	1.86E-06	3.93E-02	5.71E-02
rs12908891	Brain_Substantia_nigra	15	*FBXL22*	63892089	rs12916395	0.9921	64225563	T	C	0.51	1.86E-06	3.86E-02	5.64E-02
rs12908891	Brain_Substantia_nigra	15	*CA12*	63643968	rs11630587	0.9921	64225908	C	T	0.51	1.77E-06	2.99E-02	4.67E-02
rs12908891	Brain_Substantia_nigra	15	*FBXL22*	63892089	rs11630587	0.9921	64225908	C	T	0.51	1.77E-06	3.27E-02	4.99E-02
rs12908891	Brain_Substantia_nigra	15	*CSNK1G1*	64553079	rs11630587	0.9921	64225908	C	T	0.51	1.77E-06	3.86E-02	5.62E-02

rs12908891	Brain_Substantia_nigra	15	*ZNF609*	64865602	rs11630587	0.9921	64225908	C	T	0.51	1.77E-06	3.66E-02	5.41E-02
rs12908891	Brain_Substantia_nigra	15	*RAB8B*	63520824	rs4776677	0.9921	64227044	C	T	0.51	1.54E-06	2.85E-02	4.50E-02
rs12908891	Brain_Substantia_nigra	15	*CA12*	63643968	rs4776677	0.9921	64227044	C	T	0.51	1.54E-06	4.98E-02	6.80E-02
rs12908891	Brain_Substantia_nigra	15	*FBXL22*	63892089	rs4776677	0.9921	64227044	C	T	0.51	1.54E-06	3.00E-02	4.66E-02
rs12908891	Brain_Substantia_nigra	15	*RAB8B*	63520824	rs11071777	0.8557	64229285	C	G	0.55	9.20E-06	2.42E-02	4.27E-02
rs12908891	Brain_Substantia_nigra	15	*FBXL22*	63892089	rs11071777	0.8557	64229285	C	G	0.55	9.20E-06	2.23E-02	4.04E-02
rs12908891	Brain_Substantia_nigra	15	*CA12*	63643968	rs1563886	0.9272	64230852	G	C	0.49	8.06E-06	4.99E-02	9.23E-02
rs12908891	Brain_Substantia_nigra	15	*FBXL22*	63892089	rs1563886	0.9272	64230852	G	C	0.49	8.06E-06	4.17E-03	3.09E-02
rs12908891	Brain_Substantia_nigra	15	*RAB8B*	63520824	rs10744961	0.9960	64230930	A	G	0.51	1.86E-06	4.87E-02	6.75E-02
rs12908891	Brain_Substantia_nigra	15	*CA12*	63643968	rs10744961	0.9960	64230930	A	G	0.51	1.86E-06	3.17E-02	4.91E-02
rs12908891	Brain_Substantia_nigra	15	*FBXL22*	63892089	rs10744961	0.9960	64230930	A	G	0.51	1.86E-06	2.90E-02	4.61E-02
rs12908891	Brain_Substantia_nigra	15	*CA12*	63643968	rs6494454	0.8031	64233563	A	C	0.56	2.52E-05	4.45E-02	6.94E-02
rs12908891	Brain_Substantia_nigra	15	*CA12*	63643968	rs894660	0.8031	64234334	C	T	0.56	2.52E-05	4.45E-02	6.94E-02
rs12908891	Brain_Substantia_nigra	15	*CA12*	63643968	rs4776266	0.8031	64236175	G	T	0.56	2.23E-05	4.45E-02	6.92E-02
rs12908891	Brain_Substantia_nigra	15	*CA12*	63643968	rs4776268	1.0000	64236398	A	G	0.51	6.19E-06	4.99E-02	1.08E-01
rs12908891	Brain_Substantia_nigra	15	*FBXL22*	63892089	rs4776268	1.0000	64236398	A	G	0.51	6.19E-06	6.52E-03	5.11E-02
rs12908891	Brain_Substantia_nigra	15	*FBXL22*	63892089	rs12908891	1.0000	64236441	A	G	0.52	1.39E-06	2.05E-02	3.58E-02
rs12908891	Brain_Substantia_nigra	15	*FBXL22*	63892089	rs11071779	0.9881	64239044	G	A	0.51	1.57E-06	2.26E-02	3.90E-02
rs12908891	Brain_Substantia_nigra	15	*FBXL22*	63892089	rs11071780	0.9842	64241691	A	G	0.51	4.74E-06	9.83E-03	6.03E-02
rs12908891	Brain_Substantia_nigra	15	*FBXL22*	63892089	rs11854537	0.9842	64241733	A	G	0.51	4.74E-06	9.83E-03	6.03E-02
rs12908891	Brain_Substantia_nigra	15	*FBXL22*	63892089	rs1380844	0.9881	64242007	G	T	0.51	4.74E-06	9.83E-03	6.03E-02
rs12908891	Brain_Substantia_nigra	15	*FBXL22*	63892089	rs12916806	0.9881	64242770	C	A	0.51	4.64E-06	9.83E-03	6.03E-02
rs12908891	Brain_Substantia_nigra	15	*CA12*	63643968	rs11071781	0.9195	64243933	G	A	0.49	4.75E-06	4.08E-02	8.39E-02
rs12908891	Brain_Substantia_nigra	15	*FBXL22*	63892089	rs11071781	0.9195	64243933	G	A	0.49	4.75E-06	8.21E-03	4.08E-02
rs12908891	Brain_Substantia_nigra	15	*FBXL22*	63892089	rs1304365	0.9881	64245946	A	G	0.51	4.67E-06	9.83E-03	6.04E-02
rs12908891	Brain_Substantia_nigra	15	*RAB8B*	63520824	rs115415409	0.8420	64249559	T	C	0.45	1.45E-05	4.15E-02	6.87E-02
rs12908891	Brain_Substantia_nigra	15	*AC100830.4*	64987516	rs115415409	0.8420	64249559	T	C	0.45	1.45E-05	3.24E-02	5.86E-02
rs12908891	Brain_Substantia_nigra	15	*FBXL22*	63892089	rs145159458	0.9119	64252880	T	C	0.49	1.21E-05	6.49E-03	4.73E-02
rs12908891	Brain_Substantia_nigra	15	*FBXL22*	63892089	rs4776692	0.9881	64255083	G	A	0.50	1.06E-05	9.83E-03	6.95E-02
rs12908891	Brain_Substantia_nigra	15	*RAB8B*	63520824	rs28822416	0.8420	64257845	C	T	0.45	1.54E-05	3.60E-02	6.30E-02
rs12908891	Brain_Substantia_nigra	15	*AC100830.4*	64987516	rs28822416	0.8420	64257845	C	T	0.45	1.54E-05	4.50E-02	7.27E-02
rs12908891	Brain_Substantia_nigra	15	*RAB8B*	63520824	rs8035776	0.8420	64258018	G	A	0.45	1.54E-05	3.60E-02	6.30E-02
rs12908891	Brain_Substantia_nigra	15	*AC100830.4*	64987516	rs8035776	0.8420	64258018	G	A	0.45	1.54E-05	4.50E-02	7.27E-02
rs12908891	Brain_Substantia_nigra	15	*RAB8B*	63520824	rs6494456	0.8381	64258260	C	T	0.45	1.54E-05	3.60E-02	6.30E-02
rs12908891	Brain_Substantia_nigra	15	*AC100830.4*	64987516	rs6494456	0.8381	64258260	C	T	0.45	1.54E-05	4.50E-02	7.27E-02
rs12908891	Brain_Substantia_nigra	15	*CA12*	63643968	rs4776695	0.8350	64259115	T	C	0.46	9.12E-06	2.63E-02	4.87E-02
rs12908891	Brain_Substantia_nigra	15	*FBXL22*	63892089	rs4776695	0.8350	64259115	T	C	0.46	9.12E-06	1.34E-03	1.03E-02
rs12908891	Whole_Blood	15	*DAPK2*	64281733	rs1460544	0.8736	64201766	T	C	0.51	9.06E-04	2.55E-02	1.43E-01
rs12908891	Whole_Blood	15	*KIAA0101*	64668539	rs1460544	0.8736	64201766	T	C	0.51	9.06E-04	1.51E-02	1.29E-01
rs12908891	Whole_Blood	15	*DAPK2*	64281733	rs1037846	0.8696	64204870	G	A	0.51	6.33E-04	1.74E-02	4.54E-02

rs12908891	Whole_Blood	15	*KIAA0101*	64668539	rs1037846	0.8696	64204870	G	A	0.51	6.33E-04	1.38E-02	4.03E-02
rs12908891	Whole_Blood	15	*TPM1*	63349472	rs11071773	0.8883	64206765	A	G	0.51	8.95E-04	4.83E-02	1.40E-01
rs12908891	Whole_Blood	15	*FBXL22*	63892089	rs11071773	0.8883	64206765	A	G	0.51	8.95E-04	4.83E-02	1.40E-01
rs12908891	Whole_Blood	15	*DAPK2*	64281733	rs11071773	0.8883	64206765	A	G	0.51	8.95E-04	2.12E-02	1.10E-01
rs12908891	Whole_Blood	15	*KIAA0101*	64668539	rs11071773	0.8883	64206765	A	G	0.51	8.95E-04	3.27E-02	1.24E-01
rs12908891	Whole_Blood	15	*DAPK2*	64281733	rs12909081	0.8809	64208260	A	C	0.51	1.00E-03	1.51E-02	1.05E-01
rs12908891	Whole_Blood	15	*KIAA0101*	64668539	rs12909081	0.8809	64208260	A	C	0.51	1.00E-03	3.23E-02	1.27E-01
rs12908891	Whole_Blood	15	*TPM1*	63349472	rs749468	0.9493	64210279	T	C	0.53	2.79E-04	2.76E-02	5.77E-02
rs12908891	Whole_Blood	15	*DAPK2*	64281733	rs749468	0.9493	64210279	T	C	0.53	2.79E-04	1.90E-02	4.70E-02
rs12908891	Whole_Blood	15	*FBXL22*	63892089	rs2414844	0.8846	64210675	A	G	0.51	9.01E-04	4.91E-02	1.42E-01
rs12908891	Whole_Blood	15	*DAPK2*	64281733	rs2414844	0.8846	64210675	A	G	0.51	9.01E-04	1.88E-02	1.07E-01
rs12908891	Whole_Blood	15	*KIAA0101*	64668539	rs2414844	0.8846	64210675	A	G	0.51	9.01E-04	2.63E-02	1.17E-01
rs12908891	Whole_Blood	15	*FBXL22*	63892089	rs12592060	0.8846	64210780	T	C	0.51	9.01E-04	4.91E-02	1.42E-01
rs12908891	Whole_Blood	15	*DAPK2*	64281733	rs12592060	0.8846	64210780	T	C	0.51	9.01E-04	1.88E-02	1.07E-01
rs12908891	Whole_Blood	15	*KIAA0101*	64668539	rs12592060	0.8846	64210780	T	C	0.51	9.01E-04	2.63E-02	1.17E-01
rs12908891	Whole_Blood	15	*FBXL22*	63892089	rs1471282	0.8846	64212275	G	T	0.51	1.07E-03	4.91E-02	1.48E-01
rs12908891	Whole_Blood	15	*DAPK2*	64281733	rs1471282	0.8846	64212275	G	T	0.51	1.07E-03	1.88E-02	1.14E-01
rs12908891	Whole_Blood	15	*KIAA0101*	64668539	rs1471282	0.8846	64212275	G	T	0.51	1.07E-03	2.63E-02	1.24E-01
rs12908891	Whole_Blood	15	*DAPK2*	64281733	rs11635779	0.8808	64213826	T	C	0.50	6.34E-05	1.48E-02	5.04E-02
rs12908891	Whole_Blood	15	*KIAA0101*	64668539	rs11635779	0.8808	64213826	T	C	0.50	6.34E-05	3.89E-02	8.04E-02
rs12908891	Whole_Blood	15	*DAPK2*	64281733	rs12904374	0.8808	64214670	T	A	0.51	8.63E-04	1.62E-02	4.62E-02
rs12908891	Whole_Blood	15	*KIAA0101*	64668539	rs12904374	0.8808	64214670	T	A	0.51	8.63E-04	4.24E-02	7.79E-02
rs12908891	Whole_Blood	15	*DAPK2*	64281733	rs920762	0.9379	64214954	C	G	0.47	1.24E-05	1.17E-02	2.67E-02
rs12908891	Whole_Blood	15	*KIAA0101*	64668539	rs920762	0.9379	64214954	C	G	0.47	1.24E-05	1.56E-02	3.21E-02
rs12908891	Whole_Blood	15	*FBXL22*	63892089	rs11853632	0.8921	64217845	C	G	0.50	4.21E-05	4.56E-02	8.47E-02
rs12908891	Whole_Blood	15	*DAPK2*	64281733	rs11853632	0.8921	64217845	C	G	0.50	4.21E-05	1.55E-02	4.84E-02
rs12908891	Whole_Blood	15	*KIAA0101*	64668539	rs11853632	0.8921	64217845	C	G	0.50	4.21E-05	2.93E-02	6.64E-02
rs12908891	Whole_Blood	15	*FBXL22*	63892089	rs12907405	0.8884	64220046	T	C	0.50	4.35E-05	4.91E-02	8.86E-02
rs12908891	Whole_Blood	15	*DAPK2*	64281733	rs12907405	0.8884	64220046	T	C	0.50	4.35E-05	1.88E-02	5.33E-02
rs12908891	Whole_Blood	15	*KIAA0101*	64668539	rs12907405	0.8884	64220046	T	C	0.50	4.35E-05	2.63E-02	6.31E-02
rs12908891	Whole_Blood	15	*DAPK2*	64281733	rs11071775	0.8808	64220995	A	G	0.50	4.81E-05	2.77E-02	6.48E-02
rs12908891	Whole_Blood	15	*KIAA0101*	64668539	rs11071775	0.8808	64220995	A	G	0.50	4.81E-05	1.28E-02	4.48E-02
rs12908891	Whole_Blood	15	*DAPK2*	64281733	rs11071776	0.8808	64221010	T	A	0.50	5.49E-05	2.77E-02	6.57E-02
rs12908891	Whole_Blood	15	*KIAA0101*	64668539	rs11071776	0.8808	64221010	T	A	0.50	5.49E-05	1.28E-02	4.57E-02
rs12908891	Whole_Blood	15	*DAPK2*	64281733	rs11637858	0.8959	64224107	C	T	0.49	4.60E-05	4.50E-02	8.40E-02
rs12908891	Whole_Blood	15	*KIAA0101*	64668539	rs11637858	0.8959	64224107	C	T	0.49	4.60E-05	1.49E-02	4.75E-02
rs12908891	Whole_Blood	15	*TPM1*	63349472	rs12916395	0.9921	64225563	T	C	0.51	1.86E-06	2.44E-02	4.05E-02
rs12908891	Whole_Blood	15	*FBXL22*	63892089	rs12916395	0.9921	64225563	T	C	0.51	1.86E-06	3.28E-02	5.00E-02
rs12908891	Whole_Blood	15	*DAPK2*	64281733	rs12916395	0.9921	64225563	T	C	0.51	1.86E-06	3.13E-02	4.84E-02
rs12908891	Whole_Blood	15	*KIAA0101*	64668539	rs12916395	0.9921	64225563	T	C	0.51	1.86E-06	2.12E-02	3.67E-02

rs12908891	Whole_Blood	15	*TPM1*	63349472	rs11630587	0.9921	64225908	C	T	0.51	1.77E-06	2.32E-02	3.90E-02
rs12908891	Whole_Blood	15	*FBXL22*	63892089	rs11630587	0.9921	64225908	C	T	0.51	1.77E-06	1.95E-02	3.45E-02
rs12908891	Whole_Blood	15	*KIAA0101*	64668539	rs11630587	0.9921	64225908	C	T	0.51	1.77E-06	3.56E-02	5.30E-02
rs12908891	Whole_Blood	15	*TPM1*	63349472	rs4776677	0.9921	64227044	C	T	0.51	1.54E-06	3.00E-02	4.66E-02
rs12908891	Whole_Blood	15	*DAPK2*	64281733	rs4776677	0.9921	64227044	C	T	0.51	1.54E-06	4.61E-02	6.41E-02
rs12908891	Whole_Blood	15	*KIAA0101*	64668539	rs4776677	0.9921	64227044	C	T	0.51	1.54E-06	2.18E-02	3.73E-02
rs12908891	Whole_Blood	15	*FBXL22*	63892089	rs11071777	0.8557	64229285	C	G	0.55	9.20E-06	3.44E-02	5.43E-02
rs12908891	Whole_Blood	15	*KIAA0101*	64668539	rs11071777	0.8557	64229285	C	G	0.55	9.20E-06	1.27E-02	2.82E-02
rs12908891	Whole_Blood	15	*FBXL22*	63892089	rs1563886	0.9272	64230852	G	C	0.49	8.06E-06	2.71E-02	6.68E-02
rs12908891	Whole_Blood	15	*KIAA0101*	64668539	rs1563886	0.9272	64230852	G	C	0.49	8.06E-06	1.19E-02	4.59E-02
rs12908891	Whole_Blood	15	*TPM1*	63349472	rs10744961	0.9960	64230930	A	G	0.51	1.86E-06	2.73E-02	4.41E-02
rs12908891	Whole_Blood	15	*FBXL22*	63892089	rs10744961	0.9960	64230930	A	G	0.51	1.86E-06	2.98E-02	4.70E-02
rs12908891	Whole_Blood	15	*KIAA0101*	64668539	rs10744961	0.9960	64230930	A	G	0.51	1.86E-06	4.89E-03	1.47E-02
rs12908891	Whole_Blood	15	*TPM1*	63349472	rs6494454	0.8031	64233563	A	C	0.56	2.52E-05	1.63E-02	3.66E-02
rs12908891	Whole_Blood	15	*KIAA0101*	64668539	rs6494454	0.8031	64233563	A	C	0.56	2.52E-05	4.43E-02	6.92E-02
rs12908891	Whole_Blood	15	*TPM1*	63349472	rs894660	0.8031	64234334	C	T	0.56	2.52E-05	1.56E-02	3.57E-02
rs12908891	Whole_Blood	15	*KIAA0101*	64668539	rs894660	0.8031	64234334	C	T	0.56	2.52E-05	4.35E-02	6.84E-02
rs12908891	Whole_Blood	15	*TPM1*	63349472	rs4776266	0.8031	64236175	G	T	0.56	2.23E-05	2.46E-02	4.70E-02
rs12908891	Whole_Blood	15	*KIAA0101*	64668539	rs4776266	0.8031	64236175	G	T	0.56	2.23E-05	1.95E-02	4.06E-02
rs12908891	Whole_Blood	15	*TPM1*	63349472	rs4776268	1.0000	64236398	A	G	0.51	6.19E-06	1.88E-02	7.20E-02
rs12908891	Whole_Blood	15	*FBXL22*	63892089	rs4776268	1.0000	64236398	A	G	0.51	6.19E-06	2.80E-02	8.39E-02
rs12908891	Whole_Blood	15	*KIAA0101*	64668539	rs4776268	1.0000	64236398	A	G	0.51	6.19E-06	2.04E-02	7.41E-02
rs12908891	Whole_Blood	15	*TPM1*	63349472	rs12908891	1.0000	64236441	A	G	0.52	1.39E-06	2.66E-02	4.30E-02
rs12908891	Whole_Blood	15	*KIAA0101*	64668539	rs12908891	1.0000	64236441	A	G	0.52	1.39E-06	1.86E-02	3.35E-02
rs12908891	Whole_Blood	15	*TPM1*	63349472	rs11071779	0.9881	64239044	G	A	0.51	1.57E-06	3.26E-02	5.04E-02
rs12908891	Whole_Blood	15	*CA12*	63643968	rs11071779	0.9881	64239044	G	A	0.51	1.57E-06	2.64E-02	4.34E-02
rs12908891	Whole_Blood	15	*DAPK2*	64281733	rs11071779	0.9881	64239044	G	A	0.51	1.57E-06	2.99E-02	4.73E-02
rs12908891	Whole_Blood	15	*KIAA0101*	64668539	rs11071779	0.9881	64239044	G	A	0.51	1.57E-06	3.79E-03	1.28E-02
rs12908891	Whole_Blood	15	*TPM1*	63349472	rs11071780	0.9842	64241691	A	G	0.51	4.74E-06	3.14E-02	9.07E-02
rs12908891	Whole_Blood	15	*KIAA0101*	64668539	rs11071780	0.9842	64241691	A	G	0.51	4.74E-06	1.93E-02	7.52E-02
rs12908891	Whole_Blood	15	*TPM1*	63349472	rs11854537	0.9842	64241733	A	G	0.51	4.74E-06	3.14E-02	9.07E-02
rs12908891	Whole_Blood	15	*KIAA0101*	64668539	rs11854537	0.9842	64241733	A	G	0.51	4.74E-06	1.93E-02	7.52E-02
rs12908891	Whole_Blood	15	*TPM1*	63349472	rs1380844	0.9881	64242007	G	T	0.51	4.74E-06	3.14E-02	9.07E-02
rs12908891	Whole_Blood	15	*KIAA0101*	64668539	rs1380844	0.9881	64242007	G	T	0.51	4.74E-06	1.93E-02	7.52E-02
rs12908891	Whole_Blood	15	*TPM1*	63349472	rs12916806	0.9881	64242770	C	A	0.51	4.64E-06	3.14E-02	9.07E-02
rs12908891	Whole_Blood	15	*KIAA0101*	64668539	rs12916806	0.9881	64242770	C	A	0.51	4.64E-06	1.93E-02	7.52E-02
rs12908891	Whole_Blood	15	*KIAA0101*	64668539	rs11071781	0.9195	64243933	G	A	0.49	4.75E-06	5.90E-03	3.61E-02
rs12908891	Whole_Blood	15	*TPM1*	63349472	rs1304365	0.9881	64245946	A	G	0.51	4.67E-06	3.14E-02	9.07E-02
rs12908891	Whole_Blood	15	*KIAA0101*	64668539	rs1304365	0.9881	64245946	A	G	0.51	4.67E-06	1.93E-02	7.53E-02
rs12908891	Whole_Blood	15	*LACTB*	63424129	rs115415409	0.8420	64249559	T	C	0.45	1.45E-05	2.90E-02	5.47E-02

rs12908891	Whole_Blood	15	*RPS27L*	63434145	s115415409	0.8420	64249559	T	C	0.45	1.45E-05	4.97E-02	7.74E-02
rs12908891	Whole_Blood	15	*CA12*	63643968	s115415409	0.8420	64249559	T	C	0.45	1.45E-05	4.54E-02	7.30E-02
rs12908891	Whole_Blood	15	*DAPK2*	64281733	s115415409	0.8420	64249559	T	C	0.45	1.45E-05	1.57E-02	3.80E-02
rs12908891	Whole_Blood	15	*FBXL22*	63892089	s145159458	0.9119	64252880	T	C	0.49	1.21E-05	4.23E-02	9.64E-02
rs12908891	Whole_Blood	15	*KIAA0101*	64668539	s145159458	0.9119	64252880	T	C	0.49	1.21E-05	9.32E-03	5.30E-02
rs12908891	Whole_Blood	15	*TPM1*	63349472	rs4776692	0.9881	64255083	G	A	0.50	1.06E-05	2.94E-02	9.75E-02
rs12908891	Whole_Blood	15	*KIAA0101*	64668539	rs4776692	0.9881	64255083	G	A	0.50	1.06E-05	1.47E-02	7.78E-02
rs12908891	Whole_Blood	15	*LACTB*	63424129	rs28822416	0.8420	64257845	C	T	0.45	1.54E-05	4.15E-02	6.89E-02
rs12908891	Whole_Blood	15	*DAPK2*	64281733	rs28822416	0.8420	64257845	C	T	0.45	1.54E-05	2.19E-02	4.63E-02
rs12908891	Whole_Blood	15	*KIAA0101*	64668539	rs28822416	0.8420	64257845	C	T	0.45	1.54E-05	2.47E-02	4.98E-02
rs12908891	Whole_Blood	15	*LACTB*	63424129	rs8035776	0.8420	64258018	G	A	0.45	1.54E-05	4.63E-02	7.41E-02
rs12908891	Whole_Blood	15	*RPS27L*	63434145	rs8035776	0.8420	64258018	G	A	0.45	1.54E-05	4.60E-02	7.38E-02
rs12908891	Whole_Blood	15	*DAPK2*	64281733	rs8035776	0.8420	64258018	G	A	0.45	1.54E-05	2.21E-02	4.66E-02
rs12908891	Whole_Blood	15	*KIAA0101*	64668539	rs8035776	0.8420	64258018	G	A	0.45	1.54E-05	2.29E-02	4.76E-02
rs12908891	Whole_Blood	15	*LACTB*	63424129	rs6494456	0.8381	64258260	C	T	0.45	1.54E-05	4.15E-02	6.89E-02
rs12908891	Whole_Blood	15	*DAPK2*	64281733	rs6494456	0.8381	64258260	C	T	0.45	1.54E-05	2.19E-02	4.63E-02
rs12908891	Whole_Blood	15	*KIAA0101*	64668539	rs6494456	0.8381	64258260	C	T	0.45	1.54E-05	2.47E-02	4.98E-02
rs12908891	Whole_Blood	15	*DAPK2*	64281733	rs4776695	0.8350	64259115	T	C	0.46	9.12E-06	4.36E-02	6.81E-02
rs12908891	Whole_Blood	15	*KIAA0101*	64668539	rs4776695	0.8350	64259115	T	C	0.46	9.12E-06	4.55E-02	7.01E-02
rs7377304	Brain_Amygdala	4	*KIAA1430*	186105738	rs7377304	1.0000	187129780	G	T	0.45	2.46E-06	2.21E-02	3.88E-02
rs7377304	Brain_Anterior_cingulate_cortex_BA24	4	*SORBS2*	186692202	rs7377304	1.0000	187129780	G	T	0.45	2.46E-06	3.88E-02	5.77E-02
rs7377304	Brain_Anterior_cingulate_cortex_BA24	4	*FAM149A*	187059697	rs7377304	1.0000	187129780	G	T	0.45	2.46E-06	1.35E-02	2.81E-02
rs7377304	Brain_Anterior_cingulate_cortex_BA24	4	*F11*	187198967	rs7377304	1.0000	187129780	G	T	0.45	2.46E-06	8.33E-03	2.08E-02
rs7377304	Brain_Anterior_cingulate_cortex_BA24	4	*MTNR1A*	187465765	rs7377304	1.0000	187129780	G	T	0.45	2.46E-06	1.51E-02	3.02E-02
rs7377304	Brain_Anterior_cingulate_cortex_BA24	4	*SORBS2*	186692202	rs1053094	1.0000	187133031	T	A	0.49	2.19E-03	4.37E-02	6.48E-02
rs7377304	Brain_Anterior_cingulate_cortex_BA24	4	*FAM149A*	187059697	rs1053094	1.0000	187133031	T	A	0.49	2.19E-03	1.94E-02	3.72E-02
rs7377304	Brain_Anterior_cingulate_cortex_BA24	4	*F11*	187198967	rs1053094	1.0000	187133031	T	A	0.49	2.19E-03	1.41E-02	3.05E-02
rs7377304	Brain_Anterior_cingulate_cortex_BA24	4	*MTNR1A*	187465765	rs1053094	1.0000	187133031	T	A	0.49	2.19E-03	1.55E-02	3.22E-02
rs7377304	Brain_Caudate_basal_ganglia	4	*CYP4V2*	187123642	rs7377304	1.0000	187129780	G	T	0.45	2.46E-06	1.48E-02	2.97E-02
rs7377304	Brain_Caudate_basal_ganglia	4	*F11*	187198967	rs7377304	1.0000	187129780	G	T	0.45	2.46E-06	2.71E-02	4.47E-02
rs7377304	Brain_Caudate_basal_ganglia	4	*CYP4V2*	187123642	rs1053094	1.0000	187133031	T	A	0.49	2.19E-03	1.69E-02	3.41E-02
rs7377304	Brain_Caudate_basal_ganglia	4	*F11*	187198967	rs1053094	1.0000	187133031	T	A	0.49	2.19E-03	3.70E-02	5.75E-02
rs7377304	Brain_Cerebellar_Hemisphere	4	*C4orf47*	186359187	rs7377304	1.0000	187129780	G	T	0.45	2.46E-06	2.02E-02	3.66E-02
rs7377304	Brain_Cerebellar_Hemisphere	4	*C4orf47*	186359187	rs1053094	1.0000	187133031	T	A	0.49	2.19E-03	1.60E-02	3.28E-02
rs7377304	Brain_Cerebellum	4	*UFSP2*	186333916	rs7377304	1.0000	187129780	G	T	0.45	2.46E-06	4.55E-02	6.48E-02
rs7377304	Brain_Cerebellum	4	*PDLIM3*	186439808	rs7377304	1.0000	187129780	G	T	0.45	2.46E-06	7.07E-03	1.88E-02
rs7377304	Brain_Cerebellum	4	*ORAOV1P1*	187092214	rs7377304	1.0000	187129780	G	T	0.45	2.46E-06	2.97E-02	4.77E-02
rs7377304	Brain_Cerebellum	4	*PDLIM3*	186439808	rs1053094	1.0000	187133031	T	A	0.49	2.19E-03	9.19E-03	2.35E-02
rs7377304	Brain_Cerebellum	4	*ORAOV1P1*	187092214	rs1053094	1.0000	187133031	T	A	0.49	2.19E-03	3.21E-02	5.21E-02
rs7377304	Brain_Cortex	4	*F11*	187198967	rs7377304	1.0000	187129780	G	T	0.45	2.46E-06	1.10E-05	1.19E-03

rs7377304	Brain_Cortex	4	*F11*	187198967	rs1053094	1.0000	187133031	T	A	0.49	2.19E-03	9.31E-06	1.43E-03
rs7377304	Brain_Frontal_Cortex_BA9	4	*RP11-714G18.1*	186302025	rs7377304	1.0000	187129780	G	T	0.45	2.46E-06	2.27E-02	3.96E-02
rs7377304	Brain_Frontal_Cortex_BA9	4	*F11*	187198967	rs7377304	1.0000	187129780	G	T	0.45	2.46E-06	4.91E-02	6.87E-02
rs7377304	Brain_Frontal_Cortex_BA9	4	*RP11-714G18.1*	186302025	rs1053094	1.0000	187133031	T	A	0.49	2.19E-03	3.48E-02	5.52E-02
rs7377304	Brain_Hippocampus	4	*F11*	187198967	rs7377304	1.0000	187129780	G	T	0.45	2.46E-06	2.85E-04	3.80E-03
rs7377304	Brain_Hippocampus	4	*F11*	187198967	rs1053094	1.0000	187133031	T	A	0.49	2.19E-03	5.66E-04	5.83E-03
rs7377304	Brain_Hypothalamus	4	*AC110771.1*	187112269	rs1053094	1.0000	187133031	T	A	0.49	2.19E-03	4.08E-02	6.17E-02
rs7377304	Brain_Nucleus_accumbens_basal_ganglia	4	*CYP4V2*	187123642	rs7377304	1.0000	187129780	G	T	0.45	2.46E-06	2.81E-02	4.58E-02
rs7377304	Brain_Nucleus_accumbens_basal_ganglia	4	*CYP4V2*	187123642	rs1053094	1.0000	187133031	T	A	0.49	2.19E-03	3.68E-02	5.74E-02
rs7377304	Brain_Putamen_basal_ganglia	4	*F11*	187198967	rs7377304	1.0000	187129780	G	T	0.45	2.46E-06	5.04E-03	1.55E-02
rs7377304	Brain_Putamen_basal_ganglia	4	*FAT1*	187578406	rs7377304	1.0000	187129780	G	T	0.45	2.46E-06	1.06E-02	2.40E-02
rs7377304	Brain_Putamen_basal_ganglia	4	*F11*	187198967	rs1053094	1.0000	187133031	T	A	0.49	2.19E-03	5.58E-03	1.77E-02
rs7377304	Brain_Putamen_basal_ganglia	4	*FAT1*	187578406	rs1053094	1.0000	187133031	T	A	0.49	2.19E-03	1.46E-02	3.11E-02
rs7377304	Brain_Spinal_cord_cervical_c-1	4	*TLR3*	186999764	rs7377304	1.0000	187129780	G	T	0.45	2.46E-06	4.35E-02	6.27E-02
rs7377304	Brain_Spinal_cord_cervical_c-1	4	*TLR3*	186999764	rs1053094	1.0000	187133031	T	A	0.49	2.19E-03	4.35E-02	6.45E-02
rs7377304	Brain_Substantia_nigra	4	*F11*	187198967	rs7377304	1.0000	187129780	G	T	0.45	2.46E-06	1.19E-02	2.59E-02
rs7377304	Brain_Substantia_nigra	4	*F11*	187198967	rs1053094	1.0000	187133031	T	A	0.49	2.19E-03	1.05E-02	2.54E-02
rs7377304	Whole_Blood	4	*UFSP2*	186333916	rs7377304	1.0000	187129780	G	T	0.45	2.46E-06	3.65E-04	4.22E-03
rs7377304	Whole_Blood	4	*UFSP2*	186333916	rs1053094	1.0000	187133031	T	A	0.49	2.19E-03	5.98E-04	5.97E-03
rs55708341	Brain_Amygdala	21	*LINC00319*	44870127	rs59380543	0.9415	45617996	T	C	0.20	1.09E-05	4.09E-02	7.05E-02
rs55708341	Brain_Amygdala	21	*AP001053.11*	45229043	rs59380543	0.9415	45617996	T	C	0.20	1.09E-05	4.74E-03	2.25E-02
rs55708341	Brain_Amygdala	21	*AP001065.15*	45907815	rs59380543	0.9415	45617996	T	C	0.20	1.09E-05	4.68E-02	7.70E-02
rs55708341	Brain_Amygdala	21	*SUMO3*	46232113	rs59380543	0.9415	45617996	T	C	0.20	1.09E-05	1.13E-02	3.40E-02
rs55708341	Brain_Amygdala	21	*AP001053.11*	45229043	rs58139755	0.9415	45618128	G	T	0.20	2.00E-04	2.54E-02	7.51E-02
rs55708341	Brain_Amygdala	21	*AP001065.15*	45907815	rs58139755	0.9415	45618128	G	T	0.20	2.00E-04	2.86E-02	7.90E-02
rs55708341	Brain_Amygdala	21	*SUMO3*	46232113	rs58139755	0.9415	45618128	G	T	0.20	2.00E-04	1.65E-02	6.31E-02
rs55708341	Brain_Amygdala	21	*LINC00319*	44870127	rs113526534	0.9545	45621230	A	G	0.20	2.01E-05	4.41E-02	7.70E-02
rs55708341	Brain_Amygdala	21	*AP001053.11*	45229043	rs113526534	0.9545	45621230	A	G	0.20	2.01E-05	1.31E-02	3.93E-02
rs55708341	Brain_Amygdala	21	*AP001065.15*	45907815	rs113526534	0.9545	45621230	A	G	0.20	2.01E-05	3.07E-02	6.20E-02
rs55708341	Brain_Amygdala	21	*SUMO3*	46232113	rs113526534	0.9545	45621230	A	G	0.20	2.01E-05	1.69E-02	4.48E-02
rs55708341	Brain_Amygdala	21	*LINC00319*	44870127	rs62228210	0.9545	45621292	C	G	0.20	2.01E-05	4.41E-02	7.70E-02
rs55708341	Brain_Amygdala	21	*AP001053.11*	45229043	rs62228210	0.9545	45621292	C	G	0.20	2.01E-05	1.31E-02	3.93E-02
rs55708341	Brain_Amygdala	21	*AP001065.15*	45907815	rs62228210	0.9545	45621292	C	G	0.20	2.01E-05	3.07E-02	6.20E-02
rs55708341	Brain_Amygdala	21	*SUMO3*	46232113	rs62228210	0.9545	45621292	C	G	0.20	2.01E-05	1.69E-02	4.48E-02
rs55708341	Brain_Amygdala	21	*AP001053.11*	45229043	rs6518350	0.9740	45621817	G	A	0.20	5.13E-06	2.53E-02	5.03E-02
rs55708341	Brain_Amygdala	21	*AP001065.15*	45907815	rs6518350	0.9740	45621817	G	A	0.20	5.13E-06	4.66E-02	7.42E-02
rs55708341	Brain_Amygdala	21	*SUMO3*	46232113	rs6518350	0.9740	45621817	G	A	0.20	5.13E-06	2.80E-02	5.35E-02
rs55708341	Brain_Amygdala	21	*AP001053.11*	45229043	rs6518351	0.9740	45621939	G	T	0.20	3.98E-06	2.53E-02	4.97E-02
rs55708341	Brain_Amygdala	21	*AP001065.15*	45907815	rs6518351	0.9740	45621939	G	T	0.20	3.98E-06	4.66E-02	7.36E-02
rs55708341	Brain_Amygdala	21	*SUMO3*	46232113	rs6518351	0.9740	45621939	G	T	0.20	3.98E-06	2.80E-02	5.29E-02

rs55708341	Brain_Amygdala	21	*LINC00319*	44870127	rs56299324	0.9740	45622609	G	A	0.20	7.67E-06	4.54E-02	7.43E-02
rs55708341	Brain_Amygdala	21	*AP001053.11*	45229043	rs56299324	0.9740	45622609	G	A	0.20	7.67E-06	3.20E-02	5.96E-02
rs55708341	Brain_Amygdala	21	*LINC00319*	44870127	rs56132007	0.9740	45622705	A	G	0.20	6.83E-06	4.54E-02	7.40E-02
rs55708341	Brain_Amygdala	21	*AP001053.11*	45229043	rs56132007	0.9740	45622705	A	G	0.20	6.83E-06	3.20E-02	5.93E-02
rs55708341	Brain_Amygdala	21	*LINC00319*	44870127	rs55965762	0.9740	45622816	T	A	0.20	6.83E-06	4.54E-02	7.40E-02
rs55708341	Brain_Amygdala	21	*AP001053.11*	45229043	rs55965762	0.9740	45622816	T	A	0.20	6.83E-06	3.20E-02	5.93E-02
rs55708341	Brain_Amygdala	21	*LINC00319*	44870127	rs55736164	0.9804	45622925	C	G	0.20	6.83E-06	4.06E-02	6.88E-02
rs55708341	Brain_Amygdala	21	*AP001053.11*	45229043	rs55736164	0.9804	45622925	C	G	0.20	6.83E-06	1.57E-02	3.91E-02
rs55708341	Brain_Amygdala	21	*AP001053.11*	45229043	rs56178904	0.9740	45624551	T	C	0.20	7.10E-06	2.97E-02	5.69E-02
rs55708341	Brain_Amygdala	21	*SUMO3*	46232113	rs56178904	0.9740	45624551	T	C	0.20	7.10E-06	4.00E-02	6.85E-02
rs55708341	Brain_Amygdala	21	*AP001053.11*	45229043	rs3746959	0.9804	45625253	A	G	0.20	5.99E-06	2.51E-02	5.07E-02
rs55708341	Brain_Amygdala	21	*SUMO3*	46232113	rs3746959	0.9804	45625253	A	G	0.20	5.99E-06	1.79E-02	4.17E-02
rs55708341	Brain_Amygdala	21	*AP001053.11*	45229043	rs55708341	1.0000	45627581	T	A	0.20	2.51E-06	4.54E-02	7.10E-02
rs55708341	Brain_Amygdala	21	*AP001053.11*	45229043	rs58911644	0.9869	45629121	T	A	0.20	2.96E-06	4.54E-02	7.17E-02
rs55708341	Brain_Anterior_cingulate_cortex_BA24	21	*CSTB*	45194359	rs59380543	0.9415	45617996	T	C	0.20	1.09E-05	3.10E-02	5.95E-02
rs55708341	Brain_Anterior_cingulate_cortex_BA24	21	*ITGB2-AS1*	46345308	rs59380543	0.9415	45617996	T	C	0.20	1.09E-05	4.57E-02	7.57E-02
rs55708341	Brain_Anterior_cingulate_cortex_BA24	21	*CSTB*	45194359	rs58139755	0.9415	45618128	G	T	0.20	2.00E-04	4.28E-02	9.51E-02
rs55708341	Brain_Anterior_cingulate_cortex_BA24	21	*RRP1*	45217284	rs58139755	0.9415	45618128	G	T	0.20	2.00E-04	3.70E-02	8.88E-02
rs55708341	Brain_Anterior_cingulate_cortex_BA24	21	*CSTB*	45194359	rs113526534	0.9545	45621230	A	G	0.20	2.01E-05	2.44E-02	5.44E-02
rs55708341	Brain_Anterior_cingulate_cortex_BA24	21	*RRP1*	45217284	rs113526534	0.9545	45621230	A	G	0.20	2.01E-05	9.24E-03	3.32E-02
rs55708341	Brain_Anterior_cingulate_cortex_BA24	21	*CSTB*	45194359	rs62228210	0.9545	45621292	C	G	0.20	2.01E-05	2.44E-02	5.44E-02
rs55708341	Brain_Anterior_cingulate_cortex_BA24	21	*RRP1*	45217284	rs62228210	0.9545	45621292	C	G	0.20	2.01E-05	9.24E-03	3.32E-02
rs55708341	Brain_Anterior_cingulate_cortex_BA24	21	*RRP1*	45217284	rs6518350	0.9740	45621817	G	A	0.20	5.13E-06	9.24E-03	2.86E-02
rs55708341	Brain_Anterior_cingulate_cortex_BA24	21	*RRP1*	45217284	rs6518351	0.9740	45621939	G	T	0.20	3.98E-06	9.24E-03	2.81E-02
rs55708341	Brain_Anterior_cingulate_cortex_BA24	21	*RRP1*	45217284	rs56299324	0.9740	45622609	G	A	0.20	7.67E-06	9.24E-03	2.98E-02
rs55708341	Brain_Anterior_cingulate_cortex_BA24	21	*RRP1*	45217284	rs56132007	0.9740	45622705	A	G	0.20	6.83E-06	9.24E-03	2.96E-02
rs55708341	Brain_Anterior_cingulate_cortex_BA24	21	*RRP1*	45217284	rs55965762	0.9740	45622816	T	A	0.20	6.83E-06	9.24E-03	2.96E-02
rs55708341	Brain_Anterior_cingulate_cortex_BA24	21	*RRP1*	45217284	rs55736164	0.9804	45622925	C	G	0.20	6.83E-06	1.34E-02	3.59E-02
rs55708341	Brain_Anterior_cingulate_cortex_BA24	21	*FAM207A*	46378414	rs55736164	0.9804	45622925	C	G	0.20	6.83E-06	4.85E-02	7.73E-02
rs55708341	Brain_Anterior_cingulate_cortex_BA24	21	*CSTB*	45194359	rs62228216	0.9804	45623518	T	C	0.20	6.83E-06	4.76E-02	7.64E-02
rs55708341	Brain_Anterior_cingulate_cortex_BA24	21	*RRP1*	45217284	rs62228216	0.9804	45623518	T	C	0.20	6.83E-06	5.65E-03	2.33E-02
rs55708341	Brain_Anterior_cingulate_cortex_BA24	21	*CSTB*	45194359	rs56178904	0.9740	45624551	T	C	0.20	7.10E-06	2.17E-02	4.72E-02
rs55708341	Brain_Anterior_cingulate_cortex_BA24	21	*RRP1*	45217284	rs56178904	0.9740	45624551	T	C	0.20	7.10E-06	5.59E-03	2.33E-02
rs55708341	Brain_Anterior_cingulate_cortex_BA24	21	*RRP1*	45217284	rs3746959	0.9804	45625253	A	G	0.20	5.99E-06	3.34E-02	6.04E-02
rs55708341	Brain_Anterior_cingulate_cortex_BA24	21	*CSTB*	45194359	rs55708341	1.0000	45627581	T	A	0.20	2.51E-06	3.97E-02	6.49E-02
rs55708341	Brain_Anterior_cingulate_cortex_BA24	21	*RRP1*	45217284	rs55708341	1.0000	45627581	T	A	0.20	2.51E-06	8.64E-03	2.61E-02
rs55708341	Brain_Anterior_cingulate_cortex_BA24	21	*CSTB*	45194359	rs58911644	0.9869	45629121	T	A	0.20	2.96E-06	3.97E-02	6.55E-02
rs55708341	Brain_Anterior_cingulate_cortex_BA24	21	*RRP1*	45217284	rs58911644	0.9869	45629121	T	A	0.20	2.96E-06	8.64E-03	2.67E-02
rs55708341	Brain_Caudate_basal_ganglia	21	*TRAPPC10*	45479316	rs59380543	0.9415	45617996	T	C	0.20	1.09E-05	1.86E-02	4.43E-02
rs55708341	Brain_Caudate_basal_ganglia	21	*UBE2G2*	46205444	rs59380543	0.9415	45617996	T	C	0.20	1.09E-05	1.02E-02	3.23E-02

rs55708341	Brain_Caudate_basal_ganglia	21	*TRAPPC10*	45479316	rs58139755	0.9415	45618128	G	T	0.20	2.00E-04	4.21E-02	9.45E-02
rs55708341	Brain_Caudate_basal_ganglia	21	*UBE2G2*	46205444	rs58139755	0.9415	45618128	G	T	0.20	2.00E-04	4.63E-02	9.89E-02
rs55708341	Brain_Caudate_basal_ganglia	21	*TRAPPC10*	45479316	rs113526534	0.9545	45621230	A	G	0.20	2.01E-05	4.33E-02	7.61E-02
rs55708341	Brain_Caudate_basal_ganglia	21	*UBE2G2*	46205444	rs113526534	0.9545	45621230	A	G	0.20	2.01E-05	3.05E-02	6.18E-02
rs55708341	Brain_Caudate_basal_ganglia	21	*TRAPPC10*	45479316	rs62228210	0.9545	45621292	C	G	0.20	2.01E-05	3.29E-02	6.45E-02
rs55708341	Brain_Caudate_basal_ganglia	21	*UBE2G2*	46205444	rs62228210	0.9545	45621292	C	G	0.20	2.01E-05	3.84E-02	7.08E-02
rs55708341	Brain_Caudate_basal_ganglia	21	*TRAPPC10*	45479316	rs6518350	0.9740	45621817	G	A	0.20	5.13E-06	3.14E-02	5.74E-02
rs55708341	Brain_Caudate_basal_ganglia	21	*TRAPPC10*	45479316	rs6518351	0.9740	45621939	G	T	0.20	3.98E-06	3.14E-02	5.68E-02
rs55708341	Brain_Caudate_basal_ganglia	21	*TRAPPC10*	45479316	rs56299324	0.9740	45622609	G	A	0.20	7.67E-06	3.14E-02	5.88E-02
rs55708341	Brain_Caudate_basal_ganglia	21	*TRAPPC10*	45479316	rs56132007	0.9740	45622705	A	G	0.20	6.83E-06	3.14E-02	5.86E-02
rs55708341	Brain_Caudate_basal_ganglia	21	*TRAPPC10*	45479316	rs55965762	0.9740	45622816	T	A	0.20	6.83E-06	3.14E-02	5.86E-02
rs55708341	Brain_Caudate_basal_ganglia	21	*TRAPPC10*	45479316	rs55736164	0.9804	45622925	C	G	0.20	6.83E-06	3.79E-02	6.59E-02
rs55708341	Brain_Caudate_basal_ganglia	21	*UBE2G2*	46205444	rs55736164	0.9804	45622925	C	G	0.20	6.83E-06	4.16E-02	7.00E-02
rs55708341	Brain_Caudate_basal_ganglia	21	*TRAPPC10*	45479316	rs62228216	0.9804	45623518	T	C	0.20	6.83E-06	3.64E-02	6.42E-02
rs55708341	Brain_Caudate_basal_ganglia	21	*TRAPPC10*	45479316	rs56178904	0.9740	45624551	T	C	0.20	7.10E-06	3.82E-02	6.64E-02
rs55708341	Brain_Caudate_basal_ganglia	21	*UBE2G2*	46205444	rs56178904	0.9740	45624551	T	C	0.20	7.10E-06	4.66E-02	7.55E-02
rs55708341	Brain_Caudate_basal_ganglia	21	*TRAPPC10*	45479316	rs3746959	0.9804	45625253	A	G	0.20	5.99E-06	3.08E-02	5.75E-02
rs55708341	Brain_Caudate_basal_ganglia	21	*AP001058.3*	45626845	rs3746959	0.9804	45625253	A	G	0.20	5.99E-06	4.20E-02	6.99E-02
rs55708341	Brain_Caudate_basal_ganglia	21	*MTND5P1*	45894301	rs3746959	0.9804	45625253	A	G	0.20	5.99E-06	2.65E-02	5.25E-02
rs55708341	Brain_Caudate_basal_ganglia	21	*TRAPPC10*	45479316	rs55708341	1.0000	45627581	T	A	0.20	2.51E-06	2.90E-02	5.29E-02
rs55708341	Brain_Caudate_basal_ganglia	21	*UBE2G2*	46205444	rs55708341	1.0000	45627581	T	A	0.20	2.51E-06	3.66E-02	6.15E-02
rs55708341	Brain_Caudate_basal_ganglia	21	*TRAPPC10*	45479316	rs58911644	0.9869	45629121	T	A	0.20	2.96E-06	2.90E-02	5.35E-02
rs55708341	Brain_Caudate_basal_ganglia	21	*UBE2G2*	46205444	rs58911644	0.9869	45629121	T	A	0.20	2.96E-06	3.66E-02	6.22E-02
rs55708341	Brain_Cerebellar_Hemisphere	21	*RRP1B*	45097693	rs59380543	0.9415	45617996	T	C	0.20	1.09E-05	6.26E-03	2.55E-02
rs55708341	Brain_Cerebellar_Hemisphere	21	*AP001055.6*	45579291	rs59380543	0.9415	45617996	T	C	0.20	1.09E-05	1.19E-02	3.49E-02
rs55708341	Brain_Cerebellar_Hemisphere	21	*RRP1*	45217284	rs58139755	0.9415	45618128	G	T	0.20	2.00E-04	1.85E-02	6.59E-02
rs55708341	Brain_Cerebellar_Hemisphere	21	*AP001055.6*	45579291	rs58139755	0.9415	45618128	G	T	0.20	2.00E-04	2.81E-03	3.60E-02
rs55708341	Brain_Cerebellar_Hemisphere	21	*AP001058.3*	45626845	rs58139755	0.9415	45618128	G	T	0.20	2.00E-04	2.13E-02	6.98E-02
rs55708341	Brain_Cerebellar_Hemisphere	21	*RRP1*	45217284	rs113526534	0.9545	45621230	A	G	0.20	2.01E-05	3.67E-02	6.88E-02
rs55708341	Brain_Cerebellar_Hemisphere	21	*AP001055.6*	45579291	rs113526534	0.9545	45621230	A	G	0.20	2.01E-05	1.00E-02	3.45E-02
rs55708341	Brain_Cerebellar_Hemisphere	21	*AP001058.3*	45626845	rs113526534	0.9545	45621230	A	G	0.20	2.01E-05	2.69E-02	5.75E-02
rs55708341	Brain_Cerebellar_Hemisphere	21	*RRP1*	45217284	rs62228210	0.9545	45621292	C	G	0.20	2.01E-05	3.67E-02	6.88E-02
rs55708341	Brain_Cerebellar_Hemisphere	21	*AP001055.6*	45579291	rs62228210	0.9545	45621292	C	G	0.20	2.01E-05	1.00E-02	3.45E-02
rs55708341	Brain_Cerebellar_Hemisphere	21	*AP001058.3*	45626845	rs62228210	0.9545	45621292	C	G	0.20	2.01E-05	2.69E-02	5.75E-02
rs55708341	Brain_Cerebellar_Hemisphere	21	*RRP1*	45217284	rs6518350	0.9740	45621817	G	A	0.20	5.13E-06	2.90E-02	5.47E-02
rs55708341	Brain_Cerebellar_Hemisphere	21	*AP001055.6*	45579291	rs6518350	0.9740	45621817	G	A	0.20	5.13E-06	9.82E-03	2.95E-02
rs55708341	Brain_Cerebellar_Hemisphere	21	*AP001055.1*	45590699	rs6518350	0.9740	45621817	G	A	0.20	5.13E-06	3.80E-02	6.49E-02
rs55708341	Brain_Cerebellar_Hemisphere	21	*AP001058.3*	45626845	rs6518350	0.9740	45621817	G	A	0.20	5.13E-06	3.17E-02	5.78E-02
rs55708341	Brain_Cerebellar_Hemisphere	21	*RRP1*	45217284	rs6518351	0.9740	45621939	G	T	0.20	3.98E-06	2.90E-02	5.41E-02
rs55708341	Brain_Cerebellar_Hemisphere	21	*AP001055.6*	45579291	rs6518351	0.9740	45621939	G	T	0.20	3.98E-06	9.82E-03	2.90E-02

rs55708341	Brain_Cerebellar_Hemisphere	21	*AP001055.1*	45590699	rs6518351	0.9740	45621939	G	T	0.20	3.98E-06	3.80E-02	6.43E-02
rs55708341	Brain_Cerebellar_Hemisphere	21	*AP001058.3*	45626845	rs6518351	0.9740	45621939	G	T	0.20	3.98E-06	3.17E-02	5.72E-02
rs55708341	Brain_Cerebellar_Hemisphere	21	*RRP1*	45217284	rs56299324	0.9740	45622609	G	A	0.20	7.67E-06	2.90E-02	5.61E-02
rs55708341	Brain_Cerebellar_Hemisphere	21	*AP001055.6*	45579291	rs56299324	0.9740	45622609	G	A	0.20	7.67E-06	9.82E-03	3.07E-02
rs55708341	Brain_Cerebellar_Hemisphere	21	*AP001055.1*	45590699	rs56299324	0.9740	45622609	G	A	0.20	7.67E-06	3.80E-02	6.63E-02
rs55708341	Brain_Cerebellar_Hemisphere	21	*AP001058.3*	45626845	rs56299324	0.9740	45622609	G	A	0.20	7.67E-06	3.17E-02	5.92E-02
rs55708341	Brain_Cerebellar_Hemisphere	21	*RRP1*	45217284	rs56132007	0.9740	45622705	A	G	0.20	6.83E-06	2.90E-02	5.58E-02
rs55708341	Brain_Cerebellar_Hemisphere	21	*AP001055.6*	45579291	rs56132007	0.9740	45622705	A	G	0.20	6.83E-06	9.82E-03	3.05E-02
rs55708341	Brain_Cerebellar_Hemisphere	21	*AP001055.1*	45590699	rs56132007	0.9740	45622705	A	G	0.20	6.83E-06	3.80E-02	6.60E-02
rs55708341	Brain_Cerebellar_Hemisphere	21	*AP001058.3*	45626845	rs56132007	0.9740	45622705	A	G	0.20	6.83E-06	3.17E-02	5.90E-02
rs55708341	Brain_Cerebellar_Hemisphere	21	*RRP1*	45217284	rs55965762	0.9740	45622816	T	A	0.20	6.83E-06	2.90E-02	5.58E-02
rs55708341	Brain_Cerebellar_Hemisphere	21	*AP001055.6*	45579291	rs55965762	0.9740	45622816	T	A	0.20	6.83E-06	9.82E-03	3.05E-02
rs55708341	Brain_Cerebellar_Hemisphere	21	*AP001055.1*	45590699	rs55965762	0.9740	45622816	T	A	0.20	6.83E-06	3.80E-02	6.60E-02
rs55708341	Brain_Cerebellar_Hemisphere	21	*AP001058.3*	45626845	rs55965762	0.9740	45622816	T	A	0.20	6.83E-06	3.17E-02	5.90E-02
rs55708341	Brain_Cerebellar_Hemisphere	21	*RRP1*	45217284	rs55736164	0.9804	45622925	C	G	0.20	6.83E-06	2.90E-02	5.58E-02
rs55708341	Brain_Cerebellar_Hemisphere	21	*AP001055.6*	45579291	rs55736164	0.9804	45622925	C	G	0.20	6.83E-06	9.82E-03	3.05E-02
rs55708341	Brain_Cerebellar_Hemisphere	21	*AP001055.1*	45590699	rs55736164	0.9804	45622925	C	G	0.20	6.83E-06	3.80E-02	6.60E-02
rs55708341	Brain_Cerebellar_Hemisphere	21	*AP001058.3*	45626845	rs55736164	0.9804	45622925	C	G	0.20	6.83E-06	3.17E-02	5.90E-02
rs55708341	Brain_Cerebellar_Hemisphere	21	*RRP1*	45217284	rs62228216	0.9804	45623518	T	C	0.20	6.83E-06	1.99E-02	4.47E-02
rs55708341	Brain_Cerebellar_Hemisphere	21	*AP001055.6*	45579291	rs62228216	0.9804	45623518	T	C	0.20	6.83E-06	7.28E-03	2.63E-02
rs55708341	Brain_Cerebellar_Hemisphere	21	*AP001055.1*	45590699	rs62228216	0.9804	45623518	T	C	0.20	6.83E-06	3.63E-02	6.41E-02
rs55708341	Brain_Cerebellar_Hemisphere	21	*AP001058.3*	45626845	rs62228216	0.9804	45623518	T	C	0.20	6.83E-06	2.18E-02	4.71E-02
rs55708341	Brain_Cerebellar_Hemisphere	21	*RRP1B*	45097693	rs56178904	0.9740	45624551	T	C	0.20	7.10E-06	4.42E-02	7.29E-02
rs55708341	Brain_Cerebellar_Hemisphere	21	*CSTB*	45194359	rs56178904	0.9740	45624551	T	C	0.20	7.10E-06	4.60E-02	7.50E-02
rs55708341	Brain_Cerebellar_Hemisphere	21	*RRP1*	45217284	rs56178904	0.9740	45624551	T	C	0.20	7.10E-06	2.55E-02	5.18E-02
rs55708341	Brain_Cerebellar_Hemisphere	21	*AP001055.6*	45579291	rs56178904	0.9740	45624551	T	C	0.20	7.10E-06	7.44E-03	2.67E-02
rs55708341	Brain_Cerebellar_Hemisphere	21	*AP001058.3*	45626845	rs56178904	0.9740	45624551	T	C	0.20	7.10E-06	1.83E-02	4.28E-02
rs55708341	Brain_Cerebellar_Hemisphere	21	*RRP1B*	45097693	rs3746959	0.9804	45625253	A	G	0.20	5.99E-06	1.12E-02	3.23E-02
rs55708341	Brain_Cerebellar_Hemisphere	21	*AP001055.6*	45579291	rs3746959	0.9804	45625253	A	G	0.20	5.99E-06	2.79E-02	5.41E-02
rs55708341	Brain_Cerebellar_Hemisphere	21	*MTND5P1*	45894301	rs3746959	0.9804	45625253	A	G	0.20	5.99E-06	4.27E-02	7.07E-02
rs55708341	Brain_Cerebellar_Hemisphere	21	*RRP1B*	45097693	rs55708341	1.0000	45627581	T	A	0.20	2.51E-06	2.72E-02	5.08E-02
rs55708341	Brain_Cerebellar_Hemisphere	21	*CSTB*	45194359	rs55708341	1.0000	45627581	T	A	0.20	2.51E-06	4.25E-02	6.79E-02
rs55708341	Brain_Cerebellar_Hemisphere	21	*AP001055.6*	45579291	rs55708341	1.0000	45627581	T	A	0.20	2.51E-06	1.11E-02	2.99E-02
rs55708341	Brain_Cerebellar_Hemisphere	21	*AP001058.3*	45626845	rs55708341	1.0000	45627581	T	A	0.20	2.51E-06	3.48E-02	5.95E-02
rs55708341	Brain_Cerebellar_Hemisphere	21	*RRP1B*	45097693	rs58911644	0.9869	45629121	T	A	0.20	2.96E-06	2.72E-02	5.14E-02
rs55708341	Brain_Cerebellar_Hemisphere	21	*CSTB*	45194359	rs58911644	0.9869	45629121	T	A	0.20	2.96E-06	4.25E-02	6.86E-02
rs55708341	Brain_Cerebellar_Hemisphere	21	*AP001055.6*	45579291	rs58911644	0.9869	45629121	T	A	0.20	2.96E-06	1.11E-02	3.05E-02
rs55708341	Brain_Cerebellar_Hemisphere	21	*AP001058.3*	45626845	rs58911644	0.9869	45629121	T	A	0.20	2.96E-06	3.48E-02	6.01E-02
rs55708341	Brain_Cerebellum	21	*C21orf67*	46356278	rs58139755	0.9415	45618128	G	T	0.20	2.00E-04	4.09E-02	9.31E-02
rs55708341	Brain_Cerebellum	21	*C21orf67*	46356278	rs113526534	0.9545	45621230	A	G	0.20	2.01E-05	4.28E-02	7.55E-02

rs55708341	Brain_Cerebellum	21	*C21orf67*	46356278	rs62228210	0.9545	45621292	C	G	0.20	2.01E-05	4.28E-02	7.55E-02
rs55708341	Brain_Cerebellum	21	*C21orf67*	46356278	rs6518350	0.9740	45621817	G	A	0.20	5.13E-06	1.23E-02	3.33E-02
rs55708341	Brain_Cerebellum	21	*C21orf67*	46356278	rs6518351	0.9740	45621939	G	T	0.20	3.98E-06	1.23E-02	3.28E-02
rs55708341	Brain_Cerebellum	21	*PFKL*	45733596	rs56299324	0.9740	45622609	G	A	0.20	7.67E-06	4.72E-02	7.62E-02
rs55708341	Brain_Cerebellum	21	*C21orf67*	46356278	rs56299324	0.9740	45622609	G	A	0.20	7.67E-06	1.36E-02	3.64E-02
rs55708341	Brain_Cerebellum	21	*PFKL*	45733596	rs56132007	0.9740	45622705	A	G	0.20	6.83E-06	4.72E-02	7.60E-02
rs55708341	Brain_Cerebellum	21	*C21orf67*	46356278	rs56132007	0.9740	45622705	A	G	0.20	6.83E-06	1.36E-02	3.61E-02
rs55708341	Brain_Cerebellum	21	*PFKL*	45733596	rs55965762	0.9740	45622816	T	A	0.20	6.83E-06	4.72E-02	7.60E-02
rs55708341	Brain_Cerebellum	21	*C21orf67*	46356278	rs55965762	0.9740	45622816	T	A	0.20	6.83E-06	1.36E-02	3.61E-02
rs55708341	Brain_Cerebellum	21	*C21orf67*	46356278	rs55736164	0.9804	45622925	C	G	0.20	6.83E-06	9.34E-03	2.97E-02
rs55708341	Brain_Cerebellum	21	*C21orf67*	46356278	rs62228216	0.9804	45623518	T	C	0.20	6.83E-06	9.79E-03	3.05E-02
rs55708341	Brain_Cerebellum	21	*C21orf67*	46356278	rs56178904	0.9740	45624551	T	C	0.20	7.10E-06	3.47E-02	6.25E-02
rs55708341	Brain_Cerebellum	21	*C21orf67*	46356278	rs3746959	0.9804	45625253	A	G	0.20	5.99E-06	3.03E-02	5.69E-02
rs55708341	Brain_Cerebellum	21	*C21orf67*	46356278	rs55708341	1.0000	45627581	T	A	0.20	2.51E-06	2.07E-02	4.29E-02
rs55708341	Brain_Cerebellum	21	*C21orf67*	46356278	rs58911644	0.9869	45629121	T	A	0.20	2.96E-06	2.07E-02	4.35E-02
rs55708341	Brain_Cortex	21	*RRP1*	45217284	rs59380543	0.9415	45617996	T	C	0.20	1.09E-05	3.07E-02	5.91E-02
rs55708341	Brain_Cortex	21	*AP001055.1*	45590699	rs59380543	0.9415	45617996	T	C	0.20	1.09E-05	3.56E-02	6.48E-02
rs55708341	Brain_Cortex	21	*TSPEAR-AS1*	45931214	rs59380543	0.9415	45617996	T	C	0.20	1.09E-05	3.59E-02	6.51E-02
rs55708341	Brain_Cortex	21	*LL21NC02-1C16.1*	46349810	rs59380543	0.9415	45617996	T	C	0.20	1.09E-05	2.33E-02	5.03E-02
rs55708341	Brain_Cortex	21	*AP001055.1*	45590699	rs58139755	0.9415	45618128	G	T	0.20	2.00E-04	3.98E-02	9.19E-02
rs55708341	Brain_Cortex	21	*LL21NC02-1C16.1*	46349810	rs58139755	0.9415	45618128	G	T	0.20	2.00E-04	1.16E-02	5.54E-02
rs55708341	Brain_Cortex	21	*RRP1*	45217284	rs113526534	0.9545	45621230	A	G	0.20	2.01E-05	3.84E-02	7.08E-02
rs55708341	Brain_Cortex	21	*AP001055.1*	45590699	rs113526534	0.9545	45621230	A	G	0.20	2.01E-05	2.58E-02	5.62E-02
rs55708341	Brain_Cortex	21	*LL21NC02-1C16.1*	46349810	rs113526534	0.9545	45621230	A	G	0.20	2.01E-05	9.31E-03	3.33E-02
rs55708341	Brain_Cortex	21	*RRP1*	45217284	rs62228210	0.9545	45621292	C	G	0.20	2.01E-05	3.84E-02	7.08E-02
rs55708341	Brain_Cortex	21	*AP001055.1*	45590699	rs62228210	0.9545	45621292	C	G	0.20	2.01E-05	2.58E-02	5.62E-02
rs55708341	Brain_Cortex	21	*LL21NC02-1C16.1*	46349810	rs62228210	0.9545	45621292	C	G	0.20	2.01E-05	9.31E-03	3.33E-02
rs55708341	Brain_Cortex	21	*RRP1*	45217284	rs6518350	0.9740	45621817	G	A	0.20	5.13E-06	3.75E-02	6.43E-02
rs55708341	Brain_Cortex	21	*AP001055.1*	45590699	rs6518350	0.9740	45621817	G	A	0.20	5.13E-06	2.19E-02	4.61E-02
rs55708341	Brain_Cortex	21	*LL21NC02-1C16.1*	46349810	rs6518350	0.9740	45621817	G	A	0.20	5.13E-06	1.00E-02	2.98E-02
rs55708341	Brain_Cortex	21	*RRP1*	45217284	rs6518351	0.9740	45621939	G	T	0.20	3.98E-06	3.75E-02	6.37E-02
rs55708341	Brain_Cortex	21	*AP001055.1*	45590699	rs6518351	0.9740	45621939	G	T	0.20	3.98E-06	2.19E-02	4.55E-02
rs55708341	Brain_Cortex	21	*LL21NC02-1C16.1*	46349810	rs6518351	0.9740	45621939	G	T	0.20	3.98E-06	1.00E-02	2.93E-02
rs55708341	Brain_Cortex	21	*RRP1*	45217284	rs56299324	0.9740	45622609	G	A	0.20	7.67E-06	4.62E-02	7.51E-02
rs55708341	Brain_Cortex	21	*AP001055.1*	45590699	rs56299324	0.9740	45622609	G	A	0.20	7.67E-06	3.90E-02	6.74E-02
rs55708341	Brain_Cortex	21	*LL21NC02-1C16.1*	46349810	rs56299324	0.9740	45622609	G	A	0.20	7.67E-06	8.75E-03	2.90E-02
rs55708341	Brain_Cortex	21	*RRP1*	45217284	rs56132007	0.9740	45622705	A	G	0.20	6.83E-06	4.62E-02	7.49E-02
rs55708341	Brain_Cortex	21	*AP001055.1*	45590699	rs56132007	0.9740	45622705	A	G	0.20	6.83E-06	3.90E-02	6.71E-02
rs55708341	Brain_Cortex	21	*LL21NC02-1C16.1*	46349810	rs56132007	0.9740	45622705	A	G	0.20	6.83E-06	8.75E-03	2.88E-02
rs55708341	Brain_Cortex	21	*RRP1*	45217284	rs55965762	0.9740	45622816	T	A	0.20	6.83E-06	4.62E-02	7.49E-02

rs55708341	Brain_Cortex	21	*AP001055.1*	45590699	rs55965762	0.9740	45622816	T	A	0.20	6.83E-06	3.90E-02	6.71E-02
rs55708341	Brain_Cortex	21	*LL21NC02-1C16.1*	46349810	rs55965762	0.9740	45622816	T	A	0.20	6.83E-06	8.75E-03	2.88E-02
rs55708341	Brain_Cortex	21	*RRP1*	45217284	rs55736164	0.9804	45622925	C	G	0.20	6.83E-06	4.62E-02	7.49E-02
rs55708341	Brain_Cortex	21	*AP001055.1*	45590699	rs55736164	0.9804	45622925	C	G	0.20	6.83E-06	3.90E-02	6.71E-02
rs55708341	Brain_Cortex	21	*LL21NC02-1C16.1*	46349810	rs55736164	0.9804	45622925	C	G	0.20	6.83E-06	8.75E-03	2.88E-02
rs55708341	Brain_Cortex	21	*LL21NC02-1C16.1*	46349810	rs62228216	0.9804	45623518	T	C	0.20	6.83E-06	8.16E-03	2.78E-02
rs55708341	Brain_Cortex	21	*LL21NC02-1C16.1*	46349810	rs56178904	0.9740	45624551	T	C	0.20	7.10E-06	7.45E-03	2.67E-02
rs55708341	Brain_Cortex	21	*LL21NC02-1C16.1*	46349810	rs3746959	0.9804	45625253	A	G	0.20	5.99E-06	1.35E-02	3.57E-02
rs55708341	Brain_Cortex	21	*LL21NC02-1C16.1*	46349810	rs55708341	1.0000	45627581	T	A	0.20	2.51E-06	7.94E-03	2.50E-02
rs55708341	Brain_Cortex	21	*LL21NC02-1C16.1*	46349810	rs58911644	0.9869	45629121	T	A	0.20	2.96E-06	7.94E-03	2.55E-02
rs55708341	Brain_Frontal_Cortex_BA9	21	*LINC00313*	44890694	rs59380543	0.9415	45617996	T	C	0.20	1.09E-05	4.34E-02	7.33E-02
rs55708341	Brain_Frontal_Cortex_BA9	21	*HSF2BP*	45014223	rs59380543	0.9415	45617996	T	C	0.20	1.09E-05	3.23E-02	6.10E-02
rs55708341	Brain_Frontal_Cortex_BA9	21	*CSTB*	45194359	rs59380543	0.9415	45617996	T	C	0.20	1.09E-05	2.22E-02	4.89E-02
rs55708341	Brain_Frontal_Cortex_BA9	21	*AP001058.3*	45626845	rs59380543	0.9415	45617996	T	C	0.20	1.09E-05	1.23E-02	3.55E-02
rs55708341	Brain_Frontal_Cortex_BA9	21	*LINC00313*	44890694	rs58139755	0.9415	45618128	G	T	0.20	2.00E-04	4.76E-02	1.00E-01
rs55708341	Brain_Frontal_Cortex_BA9	21	*CSTB*	45194359	rs58139755	0.9415	45618128	G	T	0.20	2.00E-04	5.40E-03	4.32E-02
rs55708341	Brain_Frontal_Cortex_BA9	21	*AP001058.3*	45626845	rs58139755	0.9415	45618128	G	T	0.20	2.00E-04	7.07E-04	2.63E-02
rs55708341	Brain_Frontal_Cortex_BA9	21	*LINC00313*	44890694	s113526534	0.9545	45621230	A	G	0.20	2.01E-05	4.76E-02	8.07E-02
rs55708341	Brain_Frontal_Cortex_BA9	21	*CSTB*	45194359	s113526534	0.9545	45621230	A	G	0.20	2.01E-05	5.40E-03	2.61E-02
rs55708341	Brain_Frontal_Cortex_BA9	21	*AP001058.3*	45626845	s113526534	0.9545	45621230	A	G	0.20	2.01E-05	7.07E-04	1.25E-02
rs55708341	Brain_Frontal_Cortex_BA9	21	*LINC00313*	44890694	rs62228210	0.9545	45621292	C	G	0.20	2.01E-05	4.76E-02	8.07E-02
rs55708341	Brain_Frontal_Cortex_BA9	21	*CSTB*	45194359	rs62228210	0.9545	45621292	C	G	0.20	2.01E-05	5.40E-03	2.61E-02
rs55708341	Brain_Frontal_Cortex_BA9	21	*AP001058.3*	45626845	rs62228210	0.9545	45621292	C	G	0.20	2.01E-05	7.07E-04	1.25E-02
rs55708341	Brain_Frontal_Cortex_BA9	21	*LINC00313*	44890694	rs6518350	0.9740	45621817	G	A	0.20	5.13E-06	3.47E-02	6.12E-02
rs55708341	Brain_Frontal_Cortex_BA9	21	*CSTB*	45194359	rs6518350	0.9740	45621817	G	A	0.20	5.13E-06	8.92E-03	2.81E-02
rs55708341	Brain_Frontal_Cortex_BA9	21	*AP001058.3*	45626845	rs6518350	0.9740	45621817	G	A	0.20	5.13E-06	3.71E-04	7.53E-03
rs55708341	Brain_Frontal_Cortex_BA9	21	*LINC00313*	44890694	rs6518351	0.9740	45621939	G	T	0.20	3.98E-06	3.47E-02	6.06E-02
rs55708341	Brain_Frontal_Cortex_BA9	21	*CSTB*	45194359	rs6518351	0.9740	45621939	G	T	0.20	3.98E-06	8.92E-03	2.76E-02
rs55708341	Brain_Frontal_Cortex_BA9	21	*AP001058.3*	45626845	rs6518351	0.9740	45621939	G	T	0.20	3.98E-06	3.71E-04	7.25E-03
rs55708341	Brain_Frontal_Cortex_BA9	21	*LINC00313*	44890694	rs56299324	0.9740	45622609	G	A	0.20	7.67E-06	3.48E-02	6.27E-02
rs55708341	Brain_Frontal_Cortex_BA9	21	*HSF2BP*	45014223	rs56299324	0.9740	45622609	G	A	0.20	7.67E-06	4.09E-02	6.95E-02
rs55708341	Brain_Frontal_Cortex_BA9	21	*CSTB*	45194359	rs56299324	0.9740	45622609	G	A	0.20	7.67E-06	1.27E-02	3.51E-02
rs55708341	Brain_Frontal_Cortex_BA9	21	*AP001058.3*	45626845	rs56299324	0.9740	45622609	G	A	0.20	7.67E-06	2.09E-04	6.88E-03
rs55708341	Brain_Frontal_Cortex_BA9	21	*LINC00313*	44890694	rs56132007	0.9740	45622705	A	G	0.20	6.83E-06	3.48E-02	6.24E-02
rs55708341	Brain_Frontal_Cortex_BA9	21	*HSF2BP*	45014223	rs56132007	0.9740	45622705	A	G	0.20	6.83E-06	4.09E-02	6.93E-02
rs55708341	Brain_Frontal_Cortex_BA9	21	*CSTB*	45194359	rs56132007	0.9740	45622705	A	G	0.20	6.83E-06	1.27E-02	3.49E-02
rs55708341	Brain_Frontal_Cortex_BA9	21	*AP001058.3*	45626845	rs56132007	0.9740	45622705	A	G	0.20	6.83E-06	2.09E-04	6.77E-03
rs55708341	Brain_Frontal_Cortex_BA9	21	*LINC00313*	44890694	rs55965762	0.9740	45622816	T	A	0.20	6.83E-06	3.48E-02	6.24E-02
rs55708341	Brain_Frontal_Cortex_BA9	21	*HSF2BP*	45014223	rs55965762	0.9740	45622816	T	A	0.20	6.83E-06	4.09E-02	6.93E-02
rs55708341	Brain_Frontal_Cortex_BA9	21	*CSTB*	45194359	rs55965762	0.9740	45622816	T	A	0.20	6.83E-06	1.27E-02	3.49E-02

rs55708341	Brain_Frontal_Cortex_BA9	21	*AP001058.3*	45626845	rs55965762	0.9740	45622816	T	A	0.20	6.83E-06	2.09E-04	6.77E-03
rs55708341	Brain_Frontal_Cortex_BA9	21	*LINC00313*	44890694	rs55736164	0.9804	45622925	C	G	0.20	6.83E-06	4.04E-02	6.87E-02
rs55708341	Brain_Frontal_Cortex_BA9	21	*CSTB*	45194359	rs55736164	0.9804	45622925	C	G	0.20	6.83E-06	1.24E-02	3.45E-02
rs55708341	Brain_Frontal_Cortex_BA9	21	*AP001058.3*	45626845	rs55736164	0.9804	45622925	C	G	0.20	6.83E-06	1.77E-04	6.45E-03
rs55708341	Brain_Frontal_Cortex_BA9	21	*LINC00313*	44890694	rs62228216	0.9804	45623518	T	C	0.20	6.83E-06	3.48E-02	6.24E-02
rs55708341	Brain_Frontal_Cortex_BA9	21	*HSF2BP*	45014223	rs62228216	0.9804	45623518	T	C	0.20	6.83E-06	4.09E-02	6.93E-02
rs55708341	Brain_Frontal_Cortex_BA9	21	*CSTB*	45194359	rs62228216	0.9804	45623518	T	C	0.20	6.83E-06	1.27E-02	3.49E-02
rs55708341	Brain_Frontal_Cortex_BA9	21	*AP001058.3*	45626845	rs62228216	0.9804	45623518	T	C	0.20	6.83E-06	2.09E-04	6.77E-03
rs55708341	Brain_Frontal_Cortex_BA9	21	*LINC00313*	44890694	rs56178904	0.9740	45624551	T	C	0.20	7.10E-06	4.76E-02	7.66E-02
rs55708341	Brain_Frontal_Cortex_BA9	21	*HSF2BP*	45014223	rs56178904	0.9740	45624551	T	C	0.20	7.10E-06	3.62E-02	6.43E-02
rs55708341	Brain_Frontal_Cortex_BA9	21	*CSTB*	45194359	rs56178904	0.9740	45624551	T	C	0.20	7.10E-06	7.87E-03	2.75E-02
rs55708341	Brain_Frontal_Cortex_BA9	21	*AP001058.3*	45626845	rs56178904	0.9740	45624551	T	C	0.20	7.10E-06	4.10E-04	8.45E-03
rs55708341	Brain_Frontal_Cortex_BA9	21	*LINC00313*	44890694	rs3746959	0.9804	45625253	A	G	0.20	5.99E-06	4.86E-02	7.70E-02
rs55708341	Brain_Frontal_Cortex_BA9	21	*HSF2BP*	45014223	rs3746959	0.9804	45625253	A	G	0.20	5.99E-06	2.28E-02	4.80E-02
rs55708341	Brain_Frontal_Cortex_BA9	21	*CSTB*	45194359	rs3746959	0.9804	45625253	A	G	0.20	5.99E-06	2.65E-02	5.24E-02
rs55708341	Brain_Frontal_Cortex_BA9	21	*AP001058.3*	45626845	rs3746959	0.9804	45625253	A	G	0.20	5.99E-06	1.75E-03	1.38E-02
rs55708341	Brain_Frontal_Cortex_BA9	21	*LINC00313*	44890694	rs55708341	1.0000	45627581	T	A	0.20	2.51E-06	4.70E-02	7.27E-02
rs55708341	Brain_Frontal_Cortex_BA9	21	*HSF2BP*	45014223	rs55708341	1.0000	45627581	T	A	0.20	2.51E-06	4.84E-02	7.42E-02
rs55708341	Brain_Frontal_Cortex_BA9	21	*CSTB*	45194359	rs55708341	1.0000	45627581	T	A	0.20	2.51E-06	5.21E-03	2.02E-02
rs55708341	Brain_Frontal_Cortex_BA9	21	*AP001058.3*	45626845	rs55708341	1.0000	45627581	T	A	0.20	2.51E-06	6.86E-04	8.38E-03
rs55708341	Brain_Frontal_Cortex_BA9	21	*LINC00313*	44890694	rs58911644	0.9869	45629121	T	A	0.20	2.96E-06	4.70E-02	7.34E-02
rs55708341	Brain_Frontal_Cortex_BA9	21	*HSF2BP*	45014223	rs58911644	0.9869	45629121	T	A	0.20	2.96E-06	4.84E-02	7.49E-02
rs55708341	Brain_Frontal_Cortex_BA9	21	*CSTB*	45194359	rs58911644	0.9869	45629121	T	A	0.20	2.96E-06	5.21E-03	2.07E-02
rs55708341	Brain_Frontal_Cortex_BA9	21	*AP001058.3*	45626845	rs58911644	0.9869	45629121	T	A	0.20	2.96E-06	6.86E-04	8.71E-03
rs55708341	Brain_Hippocampus	21	*LRRC3DN*	45880946	rs59380543	0.9415	45617996	T	C	0.20	1.09E-05	1.56E-02	4.03E-02
rs55708341	Brain_Hippocampus	21	*ITGB2-AS1*	46345308	rs59380543	0.9415	45617996	T	C	0.20	1.09E-05	1.81E-02	4.36E-02
rs55708341	Brain_Hippocampus	21	*LL21NC02-1C16.2*	46356543	rs59380543	0.9415	45617996	T	C	0.20	1.09E-05	1.21E-02	3.52E-02
rs55708341	Brain_Hippocampus	21	*LRRC3DN*	45880946	rs58139755	0.9415	45618128	G	T	0.20	2.00E-04	4.31E-02	9.55E-02
rs55708341	Brain_Hippocampus	21	*LL21NC02-1C16.2*	46356543	rs58139755	0.9415	45618128	G	T	0.20	2.00E-04	3.17E-02	8.27E-02
rs55708341	Brain_Hippocampus	21	*LRRC3DN*	45880946	s113526534	0.9545	45621230	A	G	0.20	2.01E-05	3.30E-02	6.47E-02
rs55708341	Brain_Hippocampus	21	*LL21NC02-1C16.2*	46356543	s113526534	0.9545	45621230	A	G	0.20	2.01E-05	2.99E-02	6.11E-02
rs55708341	Brain_Hippocampus	21	*LRRC3DN*	45880946	rs62228210	0.9545	45621292	C	G	0.20	2.01E-05	3.30E-02	6.47E-02
rs55708341	Brain_Hippocampus	21	*LL21NC02-1C16.2*	46356543	rs62228210	0.9545	45621292	C	G	0.20	2.01E-05	2.99E-02	6.11E-02
rs55708341	Brain_Hippocampus	21	*LRRC3DN*	45880946	rs6518350	0.9740	45621817	G	A	0.20	5.13E-06	3.99E-02	6.69E-02
rs55708341	Brain_Hippocampus	21	*ITGB2-AS1*	46345308	rs6518350	0.9740	45621817	G	A	0.20	5.13E-06	3.54E-02	6.20E-02
rs55708341	Brain_Hippocampus	21	*LL21NC02-1C16.2*	46356543	rs6518350	0.9740	45621817	G	A	0.20	5.13E-06	2.35E-02	4.82E-02
rs55708341	Brain_Hippocampus	21	*LRRC3DN*	45880946	rs6518351	0.9740	45621939	G	T	0.20	3.98E-06	3.99E-02	6.63E-02
rs55708341	Brain_Hippocampus	21	*ITGB2-AS1*	46345308	rs6518351	0.9740	45621939	G	T	0.20	3.98E-06	3.54E-02	6.14E-02
rs55708341	Brain_Hippocampus	21	*LL21NC02-1C16.2*	46356543	rs6518351	0.9740	45621939	G	T	0.20	3.98E-06	2.35E-02	4.76E-02
rs55708341	Brain_Hippocampus	21	*LRRC3DN*	45880946	rs56299324	0.9740	45622609	G	A	0.20	7.67E-06	3.99E-02	6.83E-02

rs55708341	Brain_Hippocampus	21	*ITGB2-AS1*	46345308	rs56299324	0.9740	45622609	G	A	0.20	7.67E-06	3.54E-02	6.34E-02
rs55708341	Brain_Hippocampus	21	*LL21NC02-1C16.2*	46356543	rs56299324	0.9740	45622609	G	A	0.20	7.67E-06	2.35E-02	4.95E-02
rs55708341	Brain_Hippocampus	21	*LRRC3DN*	45880946	rs56132007	0.9740	45622705	A	G	0.20	6.83E-06	3.99E-02	6.81E-02
rs55708341	Brain_Hippocampus	21	*ITGB2-AS1*	46345308	rs56132007	0.9740	45622705	A	G	0.20	6.83E-06	3.54E-02	6.32E-02
rs55708341	Brain_Hippocampus	21	*LL21NC02-1C16.2*	46356543	rs56132007	0.9740	45622705	A	G	0.20	6.83E-06	2.35E-02	4.93E-02
rs55708341	Brain_Hippocampus	21	*LRRC3DN*	45880946	rs55965762	0.9740	45622816	T	A	0.20	6.83E-06	3.99E-02	6.81E-02
rs55708341	Brain_Hippocampus	21	*ITGB2-AS1*	46345308	rs55965762	0.9740	45622816	T	A	0.20	6.83E-06	3.54E-02	6.32E-02
rs55708341	Brain_Hippocampus	21	*LL21NC02-1C16.2*	46356543	rs55965762	0.9740	45622816	T	A	0.20	6.83E-06	2.35E-02	4.93E-02
rs55708341	Brain_Hippocampus	21	*ITGB2-AS1*	46345308	rs55736164	0.9804	45622925	C	G	0.20	6.83E-06	3.15E-02	5.87E-02
rs55708341	Brain_Hippocampus	21	*LL21NC02-1C16.2*	46356543	rs55736164	0.9804	45622925	C	G	0.20	6.83E-06	2.25E-02	4.80E-02
rs55708341	Brain_Hippocampus	21	*LL21NC02-1C16.2*	46356543	rs62228216	0.9804	45623518	T	C	0.20	6.83E-06	3.38E-02	6.13E-02
rs55708341	Brain_Hippocampus	21	*ADARB1*	46570481	rs62228216	0.9804	45623518	T	C	0.20	6.83E-06	4.57E-02	7.44E-02
rs55708341	Brain_Hippocampus	21	*LRRC3DN*	45880946	rs56178904	0.9740	45624551	T	C	0.20	7.10E-06	4.21E-02	7.07E-02
rs55708341	Brain_Hippocampus	21	*LL21NC02-1C16.2*	46356543	rs56178904	0.9740	45624551	T	C	0.20	7.10E-06	4.17E-02	7.03E-02
rs55708341	Brain_Hippocampus	21	*AP001065.2*	45839814	rs3746959	0.9804	45625253	A	G	0.20	5.99E-06	4.12E-02	6.91E-02
rs55708341	Brain_Hippocampus	21	*LRRC3DN*	45880946	rs3746959	0.9804	45625253	A	G	0.20	5.99E-06	2.42E-02	4.97E-02
rs55708341	Brain_Hippocampus	21	*ITGB2-AS1*	46345308	rs3746959	0.9804	45625253	A	G	0.20	5.99E-06	2.31E-02	4.83E-02
rs55708341	Brain_Hippocampus	21	*LL21NC02-1C16.2*	46356543	rs3746959	0.9804	45625253	A	G	0.20	5.99E-06	1.20E-02	3.35E-02
rs55708341	Brain_Hippocampus	21	*LL21NC02-1C16.2*	46356543	rs55708341	1.0000	45627581	T	A	0.20	2.51E-06	2.38E-02	4.67E-02
rs55708341	Brain_Hippocampus	21	*LL21NC02-1C16.2*	46356543	rs58911644	0.9869	45629121	T	A	0.20	2.96E-06	2.38E-02	4.74E-02
rs55708341	Brain_Hypothalamus	21	*HSF2BP*	45014223	rs59380543	0.9415	45617996	T	C	0.20	1.09E-05	3.24E-02	6.11E-02
rs55708341	Brain_Hypothalamus	21	*AGPAT3*	45345742	rs59380543	0.9415	45617996	T	C	0.20	1.09E-05	3.58E-02	6.49E-02
rs55708341	Brain_Hypothalamus	21	*HSF2BP*	45014223	rs58139755	0.9415	45618128	G	T	0.20	2.00E-04	2.98E-02	8.05E-02
rs55708341	Brain_Hypothalamus	21	*CSTB*	45194359	rs58139755	0.9415	45618128	G	T	0.20	2.00E-04	2.57E-02	7.54E-02
rs55708341	Brain_Hypothalamus	21	*AP001065.7*	45891105	rs58139755	0.9415	45618128	G	T	0.20	2.00E-04	2.17E-02	7.03E-02
rs55708341	Brain_Hypothalamus	21	*HSF2BP*	45014223	rs113526534	0.9545	45621230	A	G	0.20	2.01E-05	3.80E-02	7.03E-02
rs55708341	Brain_Hypothalamus	21	*CSTB*	45194359	rs113526534	0.9545	45621230	A	G	0.20	2.01E-05	3.61E-02	6.82E-02
rs55708341	Brain_Hypothalamus	21	*AP001065.7*	45891105	rs113526534	0.9545	45621230	A	G	0.20	2.01E-05	2.12E-02	5.05E-02
rs55708341	Brain_Hypothalamus	21	*HSF2BP*	45014223	rs62228210	0.9545	45621292	C	G	0.20	2.01E-05	3.80E-02	7.03E-02
rs55708341	Brain_Hypothalamus	21	*CSTB*	45194359	rs62228210	0.9545	45621292	C	G	0.20	2.01E-05	3.61E-02	6.82E-02
rs55708341	Brain_Hypothalamus	21	*AP001065.7*	45891105	rs62228210	0.9545	45621292	C	G	0.20	2.01E-05	2.12E-02	5.05E-02
rs55708341	Brain_Hypothalamus	21	*HSF2BP*	45014223	rs6518350	0.9740	45621817	G	A	0.20	5.13E-06	3.15E-02	5.76E-02
rs55708341	Brain_Hypothalamus	21	*CSTB*	45194359	rs6518350	0.9740	45621817	G	A	0.20	5.13E-06	2.81E-02	5.36E-02
rs55708341	Brain_Hypothalamus	21	*AP001055.1*	45590699	rs6518350	0.9740	45621817	G	A	0.20	5.13E-06	4.98E-02	7.76E-02
rs55708341	Brain_Hypothalamus	21	*AP001065.7*	45891105	rs6518350	0.9740	45621817	G	A	0.20	5.13E-06	2.64E-02	5.16E-02
rs55708341	Brain_Hypothalamus	21	*HSF2BP*	45014223	rs6518351	0.9740	45621939	G	T	0.20	3.98E-06	3.15E-02	5.70E-02
rs55708341	Brain_Hypothalamus	21	*CSTB*	45194359	rs6518351	0.9740	45621939	G	T	0.20	3.98E-06	2.81E-02	5.31E-02
rs55708341	Brain_Hypothalamus	21	*AP001055.1*	45590699	rs6518351	0.9740	45621939	G	T	0.20	3.98E-06	4.98E-02	7.70E-02
rs55708341	Brain_Hypothalamus	21	*AP001065.7*	45891105	rs6518351	0.9740	45621939	G	T	0.20	3.98E-06	2.64E-02	5.10E-02
rs55708341	Brain_Hypothalamus	21	*HSF2BP*	45014223	rs56299324	0.9740	45622609	G	A	0.20	7.67E-06	3.15E-02	5.90E-02

rs55708341	Brain_Hypothalamus	21	*CSTB*	45194359	rs56299324	0.9740	45622609	G	A	0.20	7.67E-06	2.81E-02	5.50E-02
rs55708341	Brain_Hypothalamus	21	*AP001055.1*	45590699	rs56299324	0.9740	45622609	G	A	0.20	7.67E-06	4.98E-02	7.90E-02
rs55708341	Brain_Hypothalamus	21	*AP001065.7*	45891105	rs56299324	0.9740	45622609	G	A	0.20	7.67E-06	2.64E-02	5.30E-02
rs55708341	Brain_Hypothalamus	21	*HSF2BP*	45014223	rs56132007	0.9740	45622705	A	G	0.20	6.83E-06	3.15E-02	5.87E-02
rs55708341	Brain_Hypothalamus	21	*CSTB*	45194359	rs56132007	0.9740	45622705	A	G	0.20	6.83E-06	2.81E-02	5.48E-02
rs55708341	Brain_Hypothalamus	21	*AP001055.1*	45590699	rs56132007	0.9740	45622705	A	G	0.20	6.83E-06	4.98E-02	7.87E-02
rs55708341	Brain_Hypothalamus	21	*AP001065.7*	45891105	rs56132007	0.9740	45622705	A	G	0.20	6.83E-06	2.64E-02	5.27E-02
rs55708341	Brain_Hypothalamus	21	*HSF2BP*	45014223	rs55965762	0.9740	45622816	T	A	0.20	6.83E-06	3.15E-02	5.87E-02
rs55708341	Brain_Hypothalamus	21	*CSTB*	45194359	rs55965762	0.9740	45622816	T	A	0.20	6.83E-06	2.81E-02	5.48E-02
rs55708341	Brain_Hypothalamus	21	*AP001055.1*	45590699	rs55965762	0.9740	45622816	T	A	0.20	6.83E-06	4.98E-02	7.87E-02
rs55708341	Brain_Hypothalamus	21	*AP001065.7*	45891105	rs55965762	0.9740	45622816	T	A	0.20	6.83E-06	2.64E-02	5.27E-02
rs55708341	Brain_Hypothalamus	21	*HSF2BP*	45014223	rs55736164	0.9804	45622925	C	G	0.20	6.83E-06	2.56E-02	5.18E-02
rs55708341	Brain_Hypothalamus	21	*CSTB*	45194359	rs55736164	0.9804	45622925	C	G	0.20	6.83E-06	4.55E-02	7.42E-02
rs55708341	Brain_Hypothalamus	21	*AP001055.1*	45590699	rs55736164	0.9804	45622925	C	G	0.20	6.83E-06	4.58E-02	7.45E-02
rs55708341	Brain_Hypothalamus	21	*AP001065.7*	45891105	rs55736164	0.9804	45622925	C	G	0.20	6.83E-06	2.53E-02	5.14E-02
rs55708341	Brain_Hypothalamus	21	*HSF2BP*	45014223	rs62228216	0.9804	45623518	T	C	0.20	6.83E-06	3.19E-02	5.91E-02
rs55708341	Brain_Hypothalamus	21	*CSTB*	45194359	rs62228216	0.9804	45623518	T	C	0.20	6.83E-06	2.09E-02	4.60E-02
rs55708341	Brain_Hypothalamus	21	*AP001055.1*	45590699	rs62228216	0.9804	45623518	T	C	0.20	6.83E-06	3.44E-02	6.21E-02
rs55708341	Brain_Hypothalamus	21	*AP001065.7*	45891105	rs62228216	0.9804	45623518	T	C	0.20	6.83E-06	3.44E-02	6.20E-02
rs55708341	Brain_Hypothalamus	21	*HSF2BP*	45014223	rs56178904	0.9740	45624551	T	C	0.20	7.10E-06	3.82E-02	6.65E-02
rs55708341	Brain_Hypothalamus	21	*CSTB*	45194359	rs56178904	0.9740	45624551	T	C	0.20	7.10E-06	2.71E-02	5.38E-02
rs55708341	Brain_Hypothalamus	21	*AP001055.1*	45590699	rs56178904	0.9740	45624551	T	C	0.20	7.10E-06	4.80E-02	7.70E-02
rs55708341	Brain_Hypothalamus	21	*AP001065.7*	45891105	rs56178904	0.9740	45624551	T	C	0.20	7.10E-06	2.77E-02	5.45E-02
rs55708341	Brain_Hypothalamus	21	*HSF2BP*	45014223	rs3746959	0.9804	45625253	A	G	0.20	5.99E-06	1.39E-02	3.62E-02
rs55708341	Brain_Hypothalamus	21	*HSF2BP*	45014223	rs55708341	1.0000	45627581	T	A	0.20	2.51E-06	3.71E-02	6.20E-02
rs55708341	Brain_Hypothalamus	21	*CSTB*	45194359	rs55708341	1.0000	45627581	T	A	0.20	2.51E-06	1.83E-02	3.98E-02
rs55708341	Brain_Hypothalamus	21	*AP001065.7*	45891105	rs55708341	1.0000	45627581	T	A	0.20	2.51E-06	3.73E-02	6.23E-02
rs55708341	Brain_Hypothalamus	21	*HSF2BP*	45014223	rs58911644	0.9869	45629121	T	A	0.20	2.96E-06	3.71E-02	6.27E-02
rs55708341	Brain_Hypothalamus	21	*CSTB*	45194359	rs58911644	0.9869	45629121	T	A	0.20	2.96E-06	1.83E-02	4.04E-02
rs55708341	Brain_Hypothalamus	21	*AP001065.7*	45891105	rs58911644	0.9869	45629121	T	A	0.20	2.96E-06	3.73E-02	6.30E-02
rs55708341	Brain_Nucleus_accumbens_basal_ganglia	21	*LRRC3*	45877054	rs59380543	0.9415	45617996	T	C	0.20	1.09E-05	4.59E-03	2.22E-02
rs55708341	Brain_Nucleus_accumbens_basal_ganglia	21	*LRRC3DN*	45880946	rs59380543	0.9415	45617996	T	C	0.20	1.09E-05	2.53E-02	5.28E-02
rs55708341	Brain_Nucleus_accumbens_basal_ganglia	21	*AP001058.3*	45626845	rs58139755	0.9415	45618128	G	T	0.20	2.00E-04	4.91E-02	1.02E-01
rs55708341	Brain_Nucleus_accumbens_basal_ganglia	21	*LRRC3*	45877054	rs58139755	0.9415	45618128	G	T	0.20	2.00E-04	2.12E-02	6.96E-02
rs55708341	Brain_Nucleus_accumbens_basal_ganglia	21	*AP001058.3*	45626845	s113526534	0.9545	45621230	A	G	0.20	2.01E-05	4.71E-02	8.02E-02
rs55708341	Brain_Nucleus_accumbens_basal_ganglia	21	*LRRC3*	45877054	s113526534	0.9545	45621230	A	G	0.20	2.01E-05	1.50E-02	4.20E-02
rs55708341	Brain_Nucleus_accumbens_basal_ganglia	21	*LRRC3*	45877054	rs62228210	0.9545	45621292	C	G	0.20	2.01E-05	1.62E-02	4.38E-02
rs55708341	Brain_Nucleus_accumbens_basal_ganglia	21	*LRRC3*	45877054	rs6518350	0.9740	45621817	G	A	0.20	5.13E-06	2.55E-02	5.05E-02
rs55708341	Brain_Nucleus_accumbens_basal_ganglia	21	*LRRC3*	45877054	rs6518351	0.9740	45621939	G	T	0.20	3.98E-06	2.55E-02	4.99E-02
rs55708341	Brain_Nucleus_accumbens_basal_ganglia	21	*LRRC3*	45877054	rs56299324	0.9740	45622609	G	A	0.20	7.67E-06	2.67E-02	5.33E-02

rs55708341	Brain_Nucleus_accumbens_basal_ganglia	21	*C21orf90*	45941467	rs56299324	0.9740	45622609	G	A	0.20	7.67E-06	4.36E-02	7.24E-02
rs55708341	Brain_Nucleus_accumbens_basal_ganglia	21	*LRRC3*	45877054	rs56132007	0.9740	45622705	A	G	0.20	6.83E-06	2.67E-02	5.31E-02
rs55708341	Brain_Nucleus_accumbens_basal_ganglia	21	*C21orf90*	45941467	rs56132007	0.9740	45622705	A	G	0.20	6.83E-06	4.36E-02	7.22E-02
rs55708341	Brain_Nucleus_accumbens_basal_ganglia	21	*LRRC3*	45877054	rs55965762	0.9740	45622816	T	A	0.20	6.83E-06	2.67E-02	5.31E-02
rs55708341	Brain_Nucleus_accumbens_basal_ganglia	21	*C21orf90*	45941467	rs55965762	0.9740	45622816	T	A	0.20	6.83E-06	4.36E-02	7.22E-02
rs55708341	Brain_Nucleus_accumbens_basal_ganglia	21	*LRRC3*	45877054	rs55736164	0.9804	45622925	C	G	0.20	6.83E-06	4.41E-02	7.27E-02
rs55708341	Brain_Nucleus_accumbens_basal_ganglia	21	*AP001058.3*	45626845	rs62228216	0.9804	45623518	T	C	0.20	6.83E-06	4.09E-02	6.92E-02
rs55708341	Brain_Nucleus_accumbens_basal_ganglia	21	*LRRC3*	45877054	rs62228216	0.9804	45623518	T	C	0.20	6.83E-06	2.83E-02	5.50E-02
rs55708341	Brain_Nucleus_accumbens_basal_ganglia	21	*C21orf90*	45941467	rs62228216	0.9804	45623518	T	C	0.20	6.83E-06	1.38E-02	3.65E-02
rs55708341	Brain_Nucleus_accumbens_basal_ganglia	21	*AP001058.3*	45626845	rs56178904	0.9740	45624551	T	C	0.20	7.10E-06	3.59E-02	6.39E-02
rs55708341	Brain_Nucleus_accumbens_basal_ganglia	21	*LRRC3*	45877054	rs56178904	0.9740	45624551	T	C	0.20	7.10E-06	1.82E-02	4.26E-02
rs55708341	Brain_Nucleus_accumbens_basal_ganglia	21	*C21orf90*	45941467	rs56178904	0.9740	45624551	T	C	0.20	7.10E-06	1.11E-02	3.27E-02
rs55708341	Brain_Nucleus_accumbens_basal_ganglia	21	*AP001058.3*	45626845	rs3746959	0.9804	45625253	A	G	0.20	5.99E-06	2.09E-02	4.55E-02
rs55708341	Brain_Nucleus_accumbens_basal_ganglia	21	*LRRC3*	45877054	rs3746959	0.9804	45625253	A	G	0.20	5.99E-06	2.79E-03	1.67E-02
rs55708341	Brain_Nucleus_accumbens_basal_ganglia	21	*LRRC3DN*	45880946	rs3746959	0.9804	45625253	A	G	0.20	5.99E-06	4.44E-02	7.26E-02
rs55708341	Brain_Nucleus_accumbens_basal_ganglia	21	*C21orf90*	45941467	rs3746959	0.9804	45625253	A	G	0.20	5.99E-06	3.47E-02	6.20E-02
rs55708341	Brain_Nucleus_accumbens_basal_ganglia	21	*AP001058.3*	45626845	rs55708341	1.0000	45627581	T	A	0.20	2.51E-06	4.65E-02	7.22E-02
rs55708341	Brain_Nucleus_accumbens_basal_ganglia	21	*ICOSLG*	45651861	rs55708341	1.0000	45627581	T	A	0.20	2.51E-06	4.79E-02	7.37E-02
rs55708341	Brain_Nucleus_accumbens_basal_ganglia	21	*LRRC3*	45877054	rs55708341	1.0000	45627581	T	A	0.20	2.51E-06	3.77E-02	6.27E-02
rs55708341	Brain_Nucleus_accumbens_basal_ganglia	21	*C21orf90*	45941467	rs55708341	1.0000	45627581	T	A	0.20	2.51E-06	1.11E-02	2.99E-02
rs55708341	Brain_Nucleus_accumbens_basal_ganglia	21	*AP001058.3*	45626845	rs58911644	0.9869	45629121	T	A	0.20	2.96E-06	4.65E-02	7.29E-02
rs55708341	Brain_Nucleus_accumbens_basal_ganglia	21	*ICOSLG*	45651861	rs58911644	0.9869	45629121	T	A	0.20	2.96E-06	4.79E-02	7.44E-02
rs55708341	Brain_Nucleus_accumbens_basal_ganglia	21	*LRRC3*	45877054	rs58911644	0.9869	45629121	T	A	0.20	2.96E-06	3.77E-02	6.34E-02
rs55708341	Brain_Nucleus_accumbens_basal_ganglia	21	*C21orf90*	45941467	rs58911644	0.9869	45629121	T	A	0.20	2.96E-06	1.11E-02	3.05E-02
rs55708341	Brain_Putamen_basal_ganglia	21	*LRRC3DN*	45880946	rs58139755	0.9415	45618128	G	T	0.20	2.00E-04	2.19E-02	7.06E-02
rs55708341	Brain_Putamen_basal_ganglia	21	*LRRC3DN*	45880946	s113526534	0.9545	45621230	A	G	0.20	2.01E-05	2.41E-02	5.41E-02
rs55708341	Brain_Putamen_basal_ganglia	21	*LRRC3DN*	45880946	rs62228210	0.9545	45621292	C	G	0.20	2.01E-05	2.41E-02	5.41E-02
rs55708341	Brain_Putamen_basal_ganglia	21	*LRRC3DN*	45880946	rs6518350	0.9740	45621817	G	A	0.20	5.13E-06	2.21E-02	4.63E-02
rs55708341	Brain_Putamen_basal_ganglia	21	*LRRC3DN*	45880946	rs6518351	0.9740	45621939	G	T	0.20	3.98E-06	2.21E-02	4.58E-02
rs55708341	Brain_Putamen_basal_ganglia	21	*LRRC3DN*	45880946	rs56299324	0.9740	45622609	G	A	0.20	7.67E-06	4.22E-02	7.08E-02
rs55708341	Brain_Putamen_basal_ganglia	21	*LRRC3DN*	45880946	rs56132007	0.9740	45622705	A	G	0.20	6.83E-06	4.22E-02	7.06E-02
rs55708341	Brain_Putamen_basal_ganglia	21	*LRRC3DN*	45880946	rs55965762	0.9740	45622816	T	A	0.20	6.83E-06	4.22E-02	7.06E-02
rs55708341	Brain_Putamen_basal_ganglia	21	*LRRC3DN*	45880946	rs55736164	0.9804	45622925	C	G	0.20	6.83E-06	3.86E-02	6.66E-02
rs55708341	Brain_Putamen_basal_ganglia	21	*LRRC3DN*	45880946	rs62228216	0.9804	45623518	T	C	0.20	6.83E-06	4.14E-02	6.97E-02
rs55708341	Brain_Putamen_basal_ganglia	21	*LRRC3DN*	45880946	rs56178904	0.9740	45624551	T	C	0.20	7.10E-06	4.43E-02	7.31E-02
rs55708341	Brain_Putamen_basal_ganglia	21	*SUMO3*	46232113	rs56178904	0.9740	45624551	T	C	0.20	7.10E-06	4.90E-02	7.81E-02
rs55708341	Brain_Putamen_basal_ganglia	21	*LRRC3DN*	45880946	rs3746959	0.9804	45625253	A	G	0.20	5.99E-06	4.50E-02	7.32E-02
rs55708341	Brain_Putamen_basal_ganglia	21	*SUMO3*	46232113	rs55708341	1.0000	45627581	T	A	0.20	2.51E-06	3.26E-02	5.71E-02
rs55708341	Brain_Putamen_basal_ganglia	21	*SUMO3*	46232113	rs58911644	0.9869	45629121	T	A	0.20	2.96E-06	3.26E-02	5.77E-02
rs55708341	Brain_Spinal_cord_cervical_c-1	21	*AP001058.3*	45626845	rs58139755	0.9415	45618128	G	T	0.20	2.00E-04	3.64E-02	8.81E-02

rs55708341	Brain_Spinal_cord_cervical_c-1	21	*AP001058.3*	45626845	s113526534	0.9545	45621230	A	G	0.20	2.01E-05	3.64E-02	6.85E-02
rs55708341	Brain_Spinal_cord_cervical_c-1	21	*AP001058.3*	45626845	rs62228210	0.9545	45621292	C	G	0.20	2.01E-05	3.64E-02	6.85E-02
rs55708341	Brain_Spinal_cord_cervical_c-1	21	*AP001058.3*	45626845	rs6518350	0.9740	45621817	G	A	0.20	5.13E-06	3.64E-02	6.31E-02
rs55708341	Brain_Spinal_cord_cervical_c-1	21	*AP001058.3*	45626845	rs6518351	0.9740	45621939	G	T	0.20	3.98E-06	3.64E-02	6.25E-02
rs55708341	Brain_Spinal_cord_cervical_c-1	21	*LINC00313*	44890694	rs3746959	0.9804	45625253	A	G	0.20	5.99E-06	3.41E-02	6.13E-02
rs55708341	Brain_Substantia_nigra	21	*AP001056.1*	45595854	rs59380543	0.9415	45617996	T	C	0.20	1.09E-05	2.51E-02	5.25E-02
rs55708341	Brain_Substantia_nigra	21	*TSPEAR-AS1*	45931214	rs59380543	0.9415	45617996	T	C	0.20	1.09E-05	4.99E-02	8.02E-02
rs55708341	Brain_Substantia_nigra	21	*C21orf90*	45941467	rs59380543	0.9415	45617996	T	C	0.20	1.09E-05	2.06E-02	4.69E-02
rs55708341	Brain_Substantia_nigra	21	*LINC00163*	46411890	rs59380543	0.9415	45617996	T	C	0.20	1.09E-05	3.66E-02	6.59E-02
rs55708341	Brain_Substantia_nigra	21	*AP001505.9*	46414688	rs59380543	0.9415	45617996	T	C	0.20	1.09E-05	1.95E-02	4.55E-02
rs55708341	Brain_Substantia_nigra	21	*C21orf90*	45941467	s113526534	0.9545	45621230	A	G	0.20	2.01E-05	3.92E-02	7.16E-02
rs55708341	Brain_Substantia_nigra	21	*C21orf90*	45941467	rs62228210	0.9545	45621292	C	G	0.20	2.01E-05	3.92E-02	7.16E-02
rs55708341	Brain_Substantia_nigra	21	*AIRE*	45711932	rs6518350	0.9740	45621817	G	A	0.20	5.13E-06	4.87E-02	7.63E-02
rs55708341	Brain_Substantia_nigra	21	*AIRE*	45711932	rs6518351	0.9740	45621939	G	T	0.20	3.98E-06	4.87E-02	7.57E-02
rs55708341	Brain_Substantia_nigra	21	*AP001056.1*	45595854	rs55708341	1.0000	45627581	T	A	0.20	2.51E-06	4.07E-02	6.60E-02
rs55708341	Brain_Substantia_nigra	21	*AP001056.1*	45595854	rs58911644	0.9869	45629121	T	A	0.20	2.96E-06	4.07E-02	6.66E-02
rs55708341	Whole_Blood	21	*AP001046.5*	44780128	rs59380543	0.9415	45617996	T	C	0.20	1.09E-05	4.15E-02	7.13E-02
rs55708341	Whole_Blood	21	*AP001046.5*	44780128	rs58139755	0.9415	45618128	G	T	0.20	2.00E-04	4.41E-02	9.66E-02
rs55708341	Whole_Blood	21	*RRP1B*	45097693	rs55708341	1.0000	45627581	T	A	0.20	2.51E-06	4.30E-02	6.84E-02
rs55708341	Whole_Blood	21	*RRP1B*	45097693	rs58911644	0.9869	45629121	T	A	0.20	2.96E-06	4.30E-02	6.91E-02
rs9831119	Brain_Amygdala	3	*CADM2*	85565855	rs72907315	0.9653	84681958	T	G	0.12	8.57E-05	8.28E-03	3.43E-02
rs9831119	Brain_Amygdala	3	*CADM2*	85565855	rs4856537	0.8395	84685281	A	T	0.10	3.63E-04	2.13E-02	6.44E-02
rs9831119	Brain_Amygdala	3	*CADM2*	85565855	rs76468827	0.8510	84690197	T	G	0.10	2.46E-04	2.13E-02	5.74E-02
rs9831119	Brain_Amygdala	3	*CADM2*	85565855	rs11127858	0.8510	84695356	T	C	0.10	2.61E-04	2.13E-02	5.76E-02
rs9831119	Brain_Amygdala	3	*CADM2*	85565855	rs11918179	1.0000	84704503	T	C	0.13	2.77E-05	3.37E-03	1.81E-02
rs9831119	Brain_Amygdala	3	*CADM2*	85565855	rs9866055	1.0000	84705135	G	A	0.13	5.56E-06	2.34E-03	1.23E-02
rs9831119	Brain_Amygdala	3	*CADM2*	85565855	rs9816303	1.0000	84705806	C	A	0.13	3.74E-06	3.79E-03	1.51E-02
rs9831119	Brain_Amygdala	3	*CADM2*	85565855	rs72907370	0.8510	84707037	C	T	0.10	1.65E-05	2.13E-02	4.40E-02
rs9831119	Brain_Amygdala	3	*CADM2*	85565855	rs9809351	1.0000	84707686	C	T	0.13	3.74E-06	2.34E-03	1.18E-02
rs9831119	Brain_Amygdala	3	*CADM2*	85565855	rs9809799	1.0000	84707940	C	T	0.13	3.74E-06	2.34E-03	1.18E-02
rs9831119	Brain_Amygdala	3	*CADM2*	85565855	rs9870836	1.0000	84709251	T	C	0.13	3.55E-06	2.34E-03	1.18E-02
rs9831119	Brain_Amygdala	3	*CADM2*	85565855	rs9819597	1.0000	84709515	C	T	0.13	3.74E-06	2.34E-03	1.18E-02
rs9831119	Brain_Amygdala	3	*CADM2*	85565855	rs72487709	1.0000	84710808	G	A	0.13	4.67E-06	2.34E-03	1.22E-02
rs9831119	Brain_Amygdala	3	*CADM2*	85565855	rs9881643	1.0000	84711164	A	G	0.13	3.81E-06	2.34E-03	1.18E-02
rs9831119	Brain_Amygdala	3	*CADM2*	85565855	rs9811523	1.0000	84712018	C	G	0.13	3.81E-06	2.34E-03	1.18E-02
rs9831119	Brain_Amygdala	3	*CADM2*	85565855	rs9831119	1.0000	84712077	C	T	0.13	2.98E-06	1.50E-03	9.29E-03
rs9831119	Brain_Amygdala	3	*CADM2*	85565855	rs9816064	1.0000	84712805	A	C	0.13	3.81E-06	1.19E-02	2.84E-02
rs9831119	Brain_Amygdala	3	*CADM2*	85565855	rs11127860	1.0000	84713028	G	A	0.13	3.81E-06	2.88E-03	1.31E-02
rs9831119	Brain_Amygdala	3	*CADM2*	85565855	rs11921076	0.9885	84713974	C	T	0.13	3.50E-06	6.81E-03	2.05E-02
rs9831119	Brain_Amygdala	3	*CADM2*	85565855	rs77137058	0.8282	84747357	G	A	0.10	5.44E-05	2.13E-02	4.70E-02

rs9831119	Brain_Cortex	3	*CADM2*	85565855	rs72907315	0.9653	84681958	T	G	0.12	8.57E-05	5.61E-03	2.91E-02
rs9831119	Brain_Cortex	3	*CADM2*	85565855	rs4856537	0.8395	84685281	A	T	0.10	3.63E-04	1.07E-02	4.86E-02
rs9831119	Brain_Cortex	3	*CADM2*	85565855	rs76468827	0.8510	84690197	T	G	0.10	2.46E-04	1.25E-02	4.48E-02
rs9831119	Brain_Cortex	3	*CADM2*	85565855	rs11127858	0.8510	84695356	T	C	0.10	2.61E-04	1.25E-02	4.50E-02
rs9831119	Brain_Cortex	3	*CADM2*	85565855	rs11918179	1.0000	84704503	T	C	0.13	2.77E-05	1.05E-02	3.12E-02
rs9831119	Brain_Cortex	3	*CADM2*	85565855	rs9866055	1.0000	84705135	G	A	0.13	5.56E-06	8.36E-03	2.37E-02
rs9831119	Brain_Cortex	3	*CADM2*	85565855	rs9816303	1.0000	84705806	C	A	0.13	3.74E-06	3.25E-02	5.36E-02
rs9831119	Brain_Cortex	3	*CADM2*	85565855	rs72907370	0.8510	84707037	C	T	0.10	1.65E-05	3.61E-02	6.12E-02
rs9831119	Brain_Cortex	3	*CADM2*	85565855	rs9809351	1.0000	84707686	C	T	0.13	3.74E-06	3.25E-02	5.36E-02
rs9831119	Brain_Cortex	3	*CADM2*	85565855	rs9809799	1.0000	84707940	C	T	0.13	3.74E-06	3.25E-02	5.36E-02
rs9831119	Brain_Cortex	3	*CADM2*	85565855	rs9870836	1.0000	84709251	T	C	0.13	3.55E-06	3.25E-02	5.36E-02
rs9831119	Brain_Cortex	3	*CADM2*	85565855	rs9819597	1.0000	84709515	C	T	0.13	3.74E-06	3.25E-02	5.36E-02
rs9831119	Brain_Cortex	3	*CADM2*	85565855	rs72487709	1.0000	84710808	G	A	0.13	4.67E-06	3.25E-02	5.42E-02
rs9831119	Brain_Cortex	3	*CADM2*	85565855	rs9881643	1.0000	84711164	A	G	0.13	3.81E-06	3.15E-02	5.26E-02
rs9831119	Brain_Cortex	3	*CADM2*	85565855	rs9811523	1.0000	84712018	C	G	0.13	3.81E-06	3.25E-02	5.37E-02
rs9831119	Brain_Cortex	3	*CADM2*	85565855	rs9831119	1.0000	84712077	C	T	0.13	2.98E-06	2.43E-02	4.37E-02
rs9831119	Brain_Cortex	3	*CADM2*	85565855	rs9816064	1.0000	84712805	A	C	0.13	3.81E-06	2.75E-02	4.80E-02
rs9831119	Brain_Cortex	3	*CADM2*	85565855	rs11127860	1.0000	84713028	G	A	0.13	3.81E-06	3.91E-02	6.10E-02
rs9831119	Brain_Cortex	3	*CADM2*	85565855	rs11921076	0.9885	84713974	C	T	0.13	3.50E-06	1.62E-02	3.41E-02
rs9831119	Brain_Cortex	3	*CADM2*	85565855	rs77137058	0.8282	84747357	G	A	0.10	5.44E-05	1.49E-02	3.85E-02
rs9531483	Brain_Frontal_Cortex_BA9	13	*SLITRK1*	84453936	rs2329283	0.8623	84218239	T	A	0.72	3.41E-04	4.83E-02	8.53E-02
rs9531483	Brain_Frontal_Cortex_BA9	13	*SLITRK1*	84453936	rs9546467	0.9754	84223852	A	G	0.70	9.48E-06	4.99E-02	7.53E-02
rs6722000	Brain_Amygdala	2	*PLEKHM3*	208791655	rs9967885	0.9647	209067275	G	A	0.79	4.71E-05	1.93E-02	4.41E-02
rs6722000	Brain_Amygdala	2	*PLEKHM3*	208791655	rs6731294	0.9765	209071325	A	G	0.79	1.95E-05	4.42E-02	7.01E-02
rs6722000	Brain_Amygdala	2	*PLEKHM3*	208791655	rs6731295	0.9765	209071327	A	G	0.79	1.95E-05	4.42E-02	7.01E-02
rs6722000	Brain_Amygdala	2	*PLEKHM3*	208791655	rs13025862	0.9881	209072106	T	C	0.79	1.85E-05	1.93E-02	4.12E-02
rs6722000	Brain_Amygdala	2	*PLEKHM3*	208791655	rs13387151	0.9881	209074635	C	A	0.22	1.39E-05	1.04E-02	2.99E-02
rs6722000	Brain_Amygdala	2	*PLEKHM3*	208791655	rs12623549	0.9881	209075793	C	T	0.79	8.32E-06	2.07E-02	4.25E-02
rs6722000	Brain_Amygdala	2	*PLEKHM3*	208791655	rs12474494	0.9702	209076580	A	G	0.79	2.12E-05	2.54E-02	4.95E-02
rs6722000	Brain_Anterior_cingulate_cortex_BA24	2	*AC096772.6*	208686753	rs9967885	0.9647	209067275	G	A	0.79	4.71E-05	5.04E-03	2.23E-02
rs6722000	Brain_Anterior_cingulate_cortex_BA24	2	*AC096772.6*	208686753	rs6731294	0.9765	209071325	A	G	0.79	1.95E-05	5.27E-03	2.05E-02
rs6722000	Brain_Anterior_cingulate_cortex_BA24	2	*AC096772.6*	208686753	rs6731295	0.9765	209071327	A	G	0.79	1.95E-05	5.27E-03	2.05E-02
rs6722000	Brain_Anterior_cingulate_cortex_BA24	2	*AC096772.6*	208686753	rs13025862	0.9881	209072106	T	C	0.79	1.85E-05	5.04E-03	1.99E-02
rs6722000	Brain_Anterior_cingulate_cortex_BA24	2	*AC096772.6*	208686753	rs13387151	0.9881	209074635	C	A	0.22	1.39E-05	6.62E-03	2.37E-02
rs6722000	Brain_Anterior_cingulate_cortex_BA24	2	*AC096772.6*	208686753	rs12623549	0.9881	209075793	C	T	0.79	8.32E-06	5.04E-03	1.95E-02
rs6722000	Brain_Anterior_cingulate_cortex_BA24	2	*AC096772.6*	208686753	rs6722000	1.0000	209075957	G	A	0.21	4.96E-06	7.00E-03	2.15E-02
rs6722000	Brain_Anterior_cingulate_cortex_BA24	2	*AC096772.6*	208686753	rs12474494	0.9702	209076580	A	G	0.79	2.12E-05	5.04E-03	2.05E-02
rs6722000	Brain_Caudate_basal_ganglia	2	*CREB1*	208431337	rs6722000	1.0000	209075957	G	A	0.21	4.96E-06	2.49E-02	4.57E-02
rs6722000	Brain_Cerebellar_Hemisphere	2	*CREB1*	208431337	rs9967885	0.9647	209067275	G	A	0.79	4.71E-05	4.40E-02	7.28E-02
rs6722000	Brain_Cerebellar_Hemisphere	2	*CREB1*	208431337	rs6731294	0.9765	209071325	A	G	0.79	1.95E-05	4.81E-02	7.42E-02

rs6722000	Brain_Cerebellar_Hemisphere	2	*CREB1*	208431337	rs6731295	0.9765	209071327	A	G	0.79	1.95E-05	4.81E-02	7.42E-02
rs6722000	Brain_Cerebellar_Hemisphere	2	*CREB1*	208431337	rs13025862	0.9881	209072106	T	C	0.79	1.85E-05	3.80E-02	6.32E-02
rs6722000	Brain_Cerebellar_Hemisphere	2	*CREB1*	208431337	rs12623549	0.9881	209075793	C	T	0.79	8.32E-06	4.29E-02	6.79E-02
rs6722000	Brain_Cerebellar_Hemisphere	2	*CREB1*	208431337	rs12474494	0.9702	209076580	A	G	0.79	2.12E-05	4.84E-02	7.52E-02
rs6722000	Brain_Cerebellum	2	*C2orf80*	209042432	rs9967885	0.9647	209067275	G	A	0.79	4.71E-05	3.73E-02	6.55E-02
rs6722000	Brain_Cerebellum	2	*PIKFYVE*	209177233	rs9967885	0.9647	209067275	G	A	0.79	4.71E-05	3.35E-02	6.13E-02
rs6722000	Brain_Cerebellum	2	*C2orf80*	209042432	rs6731294	0.9765	209071325	A	G	0.79	1.95E-05	1.15E-02	3.08E-02
rs6722000	Brain_Cerebellum	2	*PIKFYVE*	209177233	rs6731294	0.9765	209071325	A	G	0.79	1.95E-05	1.31E-02	3.32E-02
rs6722000	Brain_Cerebellum	2	*C2orf80*	209042432	rs6731295	0.9765	209071327	A	G	0.79	1.95E-05	1.15E-02	3.08E-02
rs6722000	Brain_Cerebellum	2	*PIKFYVE*	209177233	rs6731295	0.9765	209071327	A	G	0.79	1.95E-05	1.31E-02	3.32E-02
rs6722000	Brain_Cerebellum	2	*C2orf80*	209042432	rs13025862	0.9881	209072106	T	C	0.79	1.85E-05	1.80E-02	3.96E-02
rs6722000	Brain_Cerebellum	2	*C2orf80*	209042432	rs13387151	0.9881	209074635	C	A	0.22	1.39E-05	9.02E-03	2.78E-02
rs6722000	Brain_Cerebellum	2	*C2orf80*	209042432	rs12623549	0.9881	209075793	C	T	0.79	8.32E-06	3.05E-02	5.42E-02
rs6722000	Brain_Cerebellum	2	*PIKFYVE*	209177233	rs12623549	0.9881	209075793	C	T	0.79	8.32E-06	1.75E-02	3.84E-02
rs6722000	Brain_Cerebellum	2	*C2orf80*	209042432	rs6722000	1.0000	209075957	G	A	0.21	4.96E-06	6.70E-03	2.10E-02
rs6722000	Brain_Cerebellum	2	*PIKFYVE*	209177233	rs6722000	1.0000	209075957	G	A	0.21	4.96E-06	2.27E-02	4.31E-02
rs6722000	Brain_Cerebellum	2	*C2orf80*	209042432	rs12474494	0.9702	209076580	A	G	0.79	2.12E-05	2.32E-02	4.69E-02
rs6722000	Brain_Cerebellum	2	*PIKFYVE*	209177233	rs12474494	0.9702	209076580	A	G	0.79	2.12E-05	2.33E-02	4.70E-02
rs6722000	Brain_Cortex	2	*IDH1-AS1*	209120438	rs9967885	0.9647	209067275	G	A	0.79	4.71E-05	2.56E-03	1.66E-02
rs6722000	Brain_Cortex	2	*C2orf80*	209042432	rs6731294	0.9765	209071325	A	G	0.79	1.95E-05	4.15E-02	6.72E-02
rs6722000	Brain_Cortex	2	*IDH1-AS1*	209120438	rs6731294	0.9765	209071325	A	G	0.79	1.95E-05	6.45E-03	2.27E-02
rs6722000	Brain_Cortex	2	*C2orf80*	209042432	rs6731295	0.9765	209071327	A	G	0.79	1.95E-05	4.15E-02	6.72E-02
rs6722000	Brain_Cortex	2	*IDH1-AS1*	209120438	rs6731295	0.9765	209071327	A	G	0.79	1.95E-05	6.45E-03	2.27E-02
rs6722000	Brain_Cortex	2	*IDH1-AS1*	209120438	rs13025862	0.9881	209072106	T	C	0.79	1.85E-05	5.07E-03	2.00E-02
rs6722000	Brain_Cortex	2	*IDH1-AS1*	209120438	rs13387151	0.9881	209074635	C	A	0.22	1.39E-05	2.85E-03	1.60E-02
rs6722000	Brain_Cortex	2	*C2orf80*	209042432	rs12623549	0.9881	209075793	C	T	0.79	8.32E-06	4.48E-02	7.00E-02
rs6722000	Brain_Cortex	2	*IDH1-AS1*	209120438	rs12623549	0.9881	209075793	C	T	0.79	8.32E-06	4.51E-03	1.85E-02
rs6722000	Brain_Cortex	2	*IDH1-AS1*	209120438	rs6722000	1.0000	209075957	G	A	0.21	4.96E-06	4.81E-03	1.76E-02
rs6722000	Brain_Cortex	2	*IDH1-AS1*	209120438	rs12474494	0.9702	209076580	A	G	0.79	2.12E-05	2.71E-03	1.54E-02
rs6722000	Brain_Hippocampus	2	*PTH2R*	209471832	rs9967885	0.9647	209067275	G	A	0.79	4.71E-05	4.52E-02	7.41E-02
rs6722000	Brain_Hippocampus	2	*IDH1-AS1*	209120438	rs13387151	0.9881	209074635	C	A	0.22	1.39E-05	4.41E-02	7.10E-02
rs6722000	Brain_Hippocampus	2	*PIKFYVE*	209177233	rs12623549	0.9881	209075793	C	T	0.79	8.32E-06	1.98E-02	4.14E-02
rs6722000	Brain_Hippocampus	2	*PTH2R*	209471832	rs12623549	0.9881	209075793	C	T	0.79	8.32E-06	2.06E-02	4.24E-02
rs6722000	Brain_Hippocampus	2	*IDH1-AS1*	209120438	rs6722000	1.0000	209075957	G	A	0.21	4.96E-06	1.65E-02	3.54E-02
rs6722000	Brain_Hippocampus	2	*PIKFYVE*	209177233	rs12474494	0.9702	209076580	A	G	0.79	2.12E-05	1.92E-02	4.19E-02
rs6722000	Brain_Hippocampus	2	*PTH2R*	209471832	rs12474494	0.9702	209076580	A	G	0.79	2.12E-05	1.50E-02	3.63E-02
rs6722000	Brain_Hypothalamus	2	*PTH2R*	209471832	rs9967885	0.9647	209067275	G	A	0.79	4.71E-05	2.68E-02	5.35E-02
rs6722000	Brain_Hypothalamus	2	*PTH2R*	209471832	rs6731294	0.9765	209071325	A	G	0.79	1.95E-05	2.23E-02	4.52E-02
rs6722000	Brain_Hypothalamus	2	*PTH2R*	209471832	rs6731295	0.9765	209071327	A	G	0.79	1.95E-05	2.23E-02	4.52E-02
rs6722000	Brain_Hypothalamus	2	*PTH2R*	209471832	rs13025862	0.9881	209072106	T	C	0.79	1.85E-05	4.55E-02	7.12E-02

rs6722000	Brain_Hypothalamus	2	*PTH2R*	209471832	rs13387151	0.9881	209074635	C	A	0.22	1.39E-05	3.06E-02	5.60E-02
rs6722000	Brain_Hypothalamus	2	*PTH2R*	209471832	rs12623549	0.9881	209075793	C	T	0.79	8.32E-06	2.74E-02	5.06E-02
rs6722000	Brain_Hypothalamus	2	*PTH2R*	209471832	rs6722000	1.0000	209075957	G	A	0.21	4.96E-06	3.53E-02	5.77E-02
rs6722000	Brain_Hypothalamus	2	*PTH2R*	209471832	rs12474494	0.9702	209076580	A	G	0.79	2.12E-05	2.38E-02	4.76E-02
rs6722000	Brain_Nucleus_accumbens_basal_ganglia	2	*PIKFYVE*	209177233	rs9967885	0.9647	209067275	G	A	0.79	4.71E-05	3.24E-02	6.00E-02
rs6722000	Brain_Nucleus_accumbens_basal_ganglia	2	*PIKFYVE*	209177233	rs6731294	0.9765	209071325	A	G	0.79	1.95E-05	3.03E-02	5.47E-02
rs6722000	Brain_Nucleus_accumbens_basal_ganglia	2	*PIKFYVE*	209177233	rs6731295	0.9765	209071327	A	G	0.79	1.95E-05	3.03E-02	5.47E-02
rs6722000	Brain_Nucleus_accumbens_basal_ganglia	2	*PIKFYVE*	209177233	rs13025862	0.9881	209072106	T	C	0.79	1.85E-05	4.41E-02	6.97E-02
rs6722000	Brain_Nucleus_accumbens_basal_ganglia	2	*PIKFYVE*	209177233	rs12623549	0.9881	209075793	C	T	0.79	8.32E-06	4.98E-02	7.52E-02
rs6722000	Brain_Substantia_nigra	2	*CCNYL1*	208601413	rs9967885	0.9647	209067275	G	A	0.79	4.71E-05	4.84E-02	7.75E-02
rs6722000	Brain_Substantia_nigra	2	*CRYGEP*	208975096	rs9967885	0.9647	209067275	G	A	0.79	4.71E-05	4.27E-02	7.14E-02
rs6722000	Brain_Substantia_nigra	2	*CCNYL1*	208601413	rs6731294	0.9765	209071325	A	G	0.79	1.95E-05	4.14E-02	6.71E-02
rs6722000	Brain_Substantia_nigra	2	*CRYGEP*	208975096	rs6731294	0.9765	209071325	A	G	0.79	1.95E-05	2.40E-02	4.72E-02
rs6722000	Brain_Substantia_nigra	2	*CCNYL1*	208601413	rs6731295	0.9765	209071327	A	G	0.79	1.95E-05	4.14E-02	6.71E-02
rs6722000	Brain_Substantia_nigra	2	*CRYGEP*	208975096	rs6731295	0.9765	209071327	A	G	0.79	1.95E-05	2.40E-02	4.72E-02
rs6722000	Brain_Substantia_nigra	2	*CCNYL1*	208601413	rs13025862	0.9881	209072106	T	C	0.79	1.85E-05	3.22E-02	5.66E-02
rs6722000	Brain_Substantia_nigra	2	*CRYGEP*	208975096	rs13025862	0.9881	209072106	T	C	0.79	1.85E-05	3.15E-02	5.59E-02
rs6722000	Brain_Substantia_nigra	2	*CRYGEP*	208975096	rs13387151	0.9881	209074635	C	A	0.22	1.39E-05	2.64E-02	5.10E-02
rs6722000	Brain_Substantia_nigra	2	*CRYGEP*	208975096	rs12623549	0.9881	209075793	C	T	0.79	8.32E-06	4.02E-02	6.50E-02
rs6722000	Brain_Substantia_nigra	2	*CRYGD*	208987778	rs12623549	0.9881	209075793	C	T	0.79	8.32E-06	4.27E-02	6.78E-02
rs6722000	Brain_Substantia_nigra	2	*CCNYL1*	208601413	rs6722000	1.0000	209075957	G	A	0.21	4.96E-06	4.92E-02	7.26E-02
rs6722000	Brain_Substantia_nigra	2	*CRYGEP*	208975096	rs6722000	1.0000	209075957	G	A	0.21	4.96E-06	4.90E-02	7.24E-02
rs6722000	Whole_Blood	2	*AC007879.2*	208076115	rs6731294	0.9765	209071325	A	G	0.79	1.95E-05	4.70E-02	7.30E-02
rs6722000	Whole_Blood	2	*PIKFYVE*	209177233	rs6731294	0.9765	209071325	A	G	0.79	1.95E-05	2.82E-02	5.23E-02
rs6722000	Whole_Blood	2	*AC007879.2*	208076115	rs6731295	0.9765	209071327	A	G	0.79	1.95E-05	4.70E-02	7.30E-02
rs6722000	Whole_Blood	2	*PIKFYVE*	209177233	rs6731295	0.9765	209071327	A	G	0.79	1.95E-05	2.82E-02	5.23E-02
rs6722000	Whole_Blood	2	*PIKFYVE*	209177233	rs12623549	0.9881	209075793	C	T	0.79	8.32E-06	1.87E-02	4.00E-02
rs6722000	Whole_Blood	2	*AC007879.2*	208076115	rs6722000	1.0000	209075957	G	A	0.21	4.96E-06	4.39E-02	6.70E-02
rs6722000	Whole_Blood	2	*PIKFYVE*	209177233	rs6722000	1.0000	209075957	G	A	0.21	4.96E-06	1.08E-02	2.75E-02
rs11923588	Brain_Amygdala	3	*YEATS2-AS1*	183526253	rs9828886	0.9494	184449677	T	C	0.06	1.37E-05	9.55E-03	2.37E-02
rs11923588	Brain_Amygdala	3	*PARL*	183574934	rs9828886	0.9494	184449677	T	C	0.06	1.37E-05	4.43E-02	6.50E-02
rs11923588	Brain_Amygdala	3	*ABCC5*	183686762	rs9828886	0.9494	184449677	T	C	0.06	1.37E-05	5.98E-03	1.81E-02
rs11923588	Brain_Amygdala	3	*CAMK2N2*	183978312	rs9828886	0.9494	184449677	T	C	0.06	1.37E-05	3.59E-02	5.60E-02
rs11923588	Brain_Amygdala	3	*YEATS2-AS1*	183526253	rs12635235	0.8823	184459005	G	C	0.05	9.09E-05	6.23E-03	2.82E-02
rs11923588	Brain_Amygdala	3	*ABCC5*	183686762	rs12635235	0.8823	184459005	G	C	0.05	9.09E-05	1.06E-02	3.59E-02
rs11923588	Brain_Amygdala	3	*CAMK2N2*	183978312	rs12635235	0.8823	184459005	G	C	0.05	9.09E-05	2.46E-02	5.51E-02
rs11923588	Brain_Amygdala	3	*YEATS2-AS1*	183526253	rs4472033	1.0000	184459499	A	G	0.06	7.20E-06	7.92E-03	2.04E-02
rs11923588	Brain_Amygdala	3	*PARL*	183574934	rs4472033	1.0000	184459499	A	G	0.06	7.20E-06	1.56E-02	3.11E-02
rs11923588	Brain_Amygdala	3	*ABCC5*	183686762	rs4472033	1.0000	184459499	A	G	0.06	7.20E-06	3.63E-03	1.31E-02
rs11923588	Brain_Amygdala	3	*CAMK2N2*	183978312	rs4472033	1.0000	184459499	A	G	0.06	7.20E-06	2.62E-02	4.40E-02

rs11923588	Brain_Amygdala	3	*VPS8*	184650166	rs4472033	1.0000	184459499	A	G	0.06	7.20E-06	2.94E-02	4.77E-02
rs11923588	Brain_Amygdala	3	*YEATS2-AS1*	183526253	rs11923588	1.0000	184459667	T	C	0.06	5.66E-06	7.92E-03	2.02E-02
rs11923588	Brain_Amygdala	3	*PARL*	183574934	rs11923588	1.0000	184459667	T	C	0.06	5.66E-06	1.56E-02	3.08E-02
rs11923588	Brain_Amygdala	3	*ABCC5*	183686762	rs11923588	1.0000	184459667	T	C	0.06	5.66E-06	3.63E-03	1.29E-02
rs11923588	Brain_Amygdala	3	*CAMK2N2*	183978312	rs11923588	1.0000	184459667	T	C	0.06	5.66E-06	2.62E-02	4.37E-02
rs11923588	Brain_Amygdala	3	*VPS8*	184650166	rs11923588	1.0000	184459667	T	C	0.06	5.66E-06	2.94E-02	4.74E-02
rs11923588	Brain_Anterior_cingulate_cortex_BA24	3	*YEATS2-AS1*	183526253	rs9828886	0.9494	184449677	T	C	0.06	1.37E-05	7.60E-03	2.07E-02
rs11923588	Brain_Anterior_cingulate_cortex_BA24	3	*ABCC5*	183686762	rs9828886	0.9494	184449677	T	C	0.06	1.37E-05	1.70E-02	3.38E-02
rs11923588	Brain_Anterior_cingulate_cortex_BA24	3	*SENP2*	185325811	rs9828886	0.9494	184449677	T	C	0.06	1.37E-05	4.93E-02	7.03E-02
rs11923588	Brain_Anterior_cingulate_cortex_BA24	3	*YEATS2-AS1*	183526253	rs12635235	0.8823	184459005	G	C	0.05	9.09E-05	9.82E-03	3.46E-02
rs11923588	Brain_Anterior_cingulate_cortex_BA24	3	*ABCC5*	183686762	rs12635235	0.8823	184459005	G	C	0.05	9.09E-05	1.30E-02	3.96E-02
rs11923588	Brain_Anterior_cingulate_cortex_BA24	3	*DVL3*	183882287	rs12635235	0.8823	184459005	G	C	0.05	9.09E-05	4.86E-02	8.22E-02
rs11923588	Brain_Anterior_cingulate_cortex_BA24	3	*VPS8*	184650166	rs12635235	0.8823	184459005	G	C	0.05	9.09E-05	2.52E-02	5.59E-02
rs11923588	Brain_Anterior_cingulate_cortex_BA24	3	*YEATS2-AS1*	183526253	rs4472033	1.0000	184459499	A	G	0.06	7.20E-06	1.68E-02	3.26E-02
rs11923588	Brain_Anterior_cingulate_cortex_BA24	3	*ABCC5*	183686762	rs4472033	1.0000	184459499	A	G	0.06	7.20E-06	8.10E-03	2.07E-02
rs11923588	Brain_Anterior_cingulate_cortex_BA24	3	*VPS8*	184650166	rs4472033	1.0000	184459499	A	G	0.06	7.20E-06	1.96E-02	3.61E-02
rs11923588	Brain_Anterior_cingulate_cortex_BA24	3	*YEATS2-AS1*	183526253	rs11923588	1.0000	184459667	T	C	0.06	5.66E-06	1.68E-02	3.23E-02
rs11923588	Brain_Anterior_cingulate_cortex_BA24	3	*ABCC5*	183686762	rs11923588	1.0000	184459667	T	C	0.06	5.66E-06	8.10E-03	2.05E-02
rs11923588	Brain_Anterior_cingulate_cortex_BA24	3	*VPS8*	184650166	rs11923588	1.0000	184459667	T	C	0.06	5.66E-06	1.96E-02	3.59E-02
rs11923588	Brain_Caudate_basal_ganglia	3	*EIF4G1*	184042714	rs9828886	0.9494	184449677	T	C	0.06	1.37E-05	2.30E-02	4.12E-02
rs11923588	Brain_Caudate_basal_ganglia	3	*FAM131A*	184058843	rs9828886	0.9494	184449677	T	C	0.06	1.37E-05	4.65E-02	6.74E-02
rs11923588	Brain_Caudate_basal_ganglia	3	*RP11-433C9.2*	184138380	rs9828886	0.9494	184449677	T	C	0.06	1.37E-05	3.36E-02	5.34E-02
rs11923588	Brain_Caudate_basal_ganglia	3	*C3orf70*	184833320	rs9828886	0.9494	184449677	T	C	0.06	1.37E-05	4.60E-02	6.68E-02
rs11923588	Brain_Caudate_basal_ganglia	3	*SENP2*	185325811	rs9828886	0.9494	184449677	T	C	0.06	1.37E-05	1.16E-02	2.67E-02
rs11923588	Brain_Caudate_basal_ganglia	3	*EIF4G1*	184042714	rs12635235	0.8823	184459005	G	C	0.05	9.09E-05	1.01E-02	3.50E-02
rs11923588	Brain_Caudate_basal_ganglia	3	*FAM131A*	184058843	rs12635235	0.8823	184459005	G	C	0.05	9.09E-05	4.55E-02	7.89E-02
rs11923588	Brain_Caudate_basal_ganglia	3	*YEATS2-AS1*	183526253	rs4472033	1.0000	184459499	A	G	0.06	7.20E-06	4.74E-02	6.72E-02
rs11923588	Brain_Caudate_basal_ganglia	3	*FAM131A*	184058843	rs4472033	1.0000	184459499	A	G	0.06	7.20E-06	3.06E-02	4.90E-02
rs11923588	Brain_Caudate_basal_ganglia	3	*SENP2*	185325811	rs4472033	1.0000	184459499	A	G	0.06	7.20E-06	1.91E-02	3.55E-02
rs11923588	Brain_Caudate_basal_ganglia	3	*YEATS2-AS1*	183526253	rs11923588	1.0000	184459667	T	C	0.06	5.66E-06	4.74E-02	6.69E-02
rs11923588	Brain_Caudate_basal_ganglia	3	*FAM131A*	184058843	rs11923588	1.0000	184459667	T	C	0.06	5.66E-06	3.06E-02	4.87E-02
rs11923588	Brain_Caudate_basal_ganglia	3	*SENP2*	185325811	rs11923588	1.0000	184459667	T	C	0.06	5.66E-06	1.91E-02	3.53E-02
rs11923588	Brain_Cerebellar_Hemisphere	3	*HSP90AA5P*	183834413	rs9828886	0.9494	184449677	T	C	0.06	1.37E-05	4.24E-02	6.30E-02
rs11923588	Brain_Cerebellar_Hemisphere	3	*HSP90AA5P*	183834413	rs4472033	1.0000	184459499	A	G	0.06	7.20E-06	2.43E-02	4.17E-02
rs11923588	Brain_Cerebellar_Hemisphere	3	*RP11-329B9.5*	184460839	rs4472033	1.0000	184459499	A	G	0.06	7.20E-06	1.48E-02	3.01E-02
rs11923588	Brain_Cerebellar_Hemisphere	3	*HSP90AA5P*	183834413	rs11923588	1.0000	184459667	T	C	0.06	5.66E-06	2.43E-02	4.14E-02
rs11923588	Brain_Cerebellar_Hemisphere	3	*RP11-329B9.5*	184460839	rs11923588	1.0000	184459667	T	C	0.06	5.66E-06	1.48E-02	2.98E-02
rs11923588	Brain_Cerebellum	3	*MAP6D1*	183538523	rs9828886	0.9494	184449677	T	C	0.06	1.37E-05	2.38E-02	4.22E-02
rs11923588	Brain_Cerebellum	3	*MIR1224*	183959235	rs9828886	0.9494	184449677	T	C	0.06	1.37E-05	4.37E-02	6.44E-02
rs11923588	Brain_Cerebellum	3	*EHHADH*	184954095	rs9828886	0.9494	184449677	T	C	0.06	1.37E-05	2.96E-02	4.89E-02

rs11923588	Brain_Cerebellum	3	*MAP6D1*	183538523	rs12635235	0.8823	184459005	G	C	0.05	9.09E-05	1.03E-02	3.54E-02
rs11923588	Brain_Cerebellum	3	*EPHB3*	184289884	rs12635235	0.8823	184459005	G	C	0.05	9.09E-05	3.10E-02	6.28E-02
rs11923588	Brain_Cerebellum	3	*EHHADH*	184954095	rs12635235	0.8823	184459005	G	C	0.05	9.09E-05	9.57E-03	3.41E-02
rs11923588	Brain_Cerebellum	3	*RP11-537I16.2*	185324512	rs12635235	0.8823	184459005	G	C	0.05	9.09E-05	1.68E-02	4.49E-02
rs11923588	Brain_Cerebellum	3	*CAMK2N2*	183978312	rs4472033	1.0000	184459499	A	G	0.06	7.20E-06	1.98E-02	3.64E-02
rs11923588	Brain_Cerebellum	3	*CAMK2N2*	183978312	rs11923588	1.0000	184459667	T	C	0.06	5.66E-06	1.98E-02	3.61E-02
rs11923588	Brain_Cortex	3	*YEATS2-AS1*	183526253	rs12635235	0.8823	184459005	G	C	0.05	9.09E-05	2.08E-02	5.03E-02
rs11923588	Brain_Frontal_Cortex_BA9	3	*YEATS2-AS1*	183526253	rs9828886	0.9494	184449677	T	C	0.06	1.37E-05	3.67E-02	5.69E-02
rs11923588	Brain_Hippocampus	3	*YEATS2-AS1*	183526253	rs9828886	0.9494	184449677	T	C	0.06	1.37E-05	2.10E-02	3.88E-02
rs11923588	Brain_Hippocampus	3	*ABCC5*	183686762	rs9828886	0.9494	184449677	T	C	0.06	1.37E-05	3.43E-02	5.42E-02
rs11923588	Brain_Hippocampus	3	*TMEM41A*	185205564	rs9828886	0.9494	184449677	T	C	0.06	1.37E-05	2.91E-02	4.84E-02
rs11923588	Brain_Hippocampus	3	*RP11-778D9.4*	183867857	rs12635235	0.8823	184459005	G	C	0.05	9.09E-05	2.90E-02	6.04E-02
rs11923588	Brain_Hippocampus	3	*RP11-329B9.4*	184454300	rs4472033	1.0000	184459499	A	G	0.06	7.20E-06	3.38E-02	5.26E-02
rs11923588	Brain_Hippocampus	3	*RP11-329B9.4*	184454300	rs11923588	1.0000	184459667	T	C	0.06	5.66E-06	3.38E-02	5.23E-02
rs11923588	Brain_Nucleus_accumbens_basal_ganglia	3	*AP2M1*	183897178	rs9828886	0.9494	184449677	T	C	0.06	1.37E-05	2.88E-02	4.80E-02
rs11923588	Brain_Nucleus_accumbens_basal_ganglia	3	*ABCF3*	183907803	rs9828886	0.9494	184449677	T	C	0.06	1.37E-05	2.50E-02	4.35E-02
rs11923588	Brain_Nucleus_accumbens_basal_ganglia	3	*SENP2*	185325811	rs9828886	0.9494	184449677	T	C	0.06	1.37E-05	4.81E-02	6.90E-02
rs11923588	Brain_Nucleus_accumbens_basal_ganglia	3	*ABCF3*	183907803	rs12635235	0.8823	184459005	G	C	0.05	9.09E-05	3.90E-02	7.18E-02
rs11923588	Brain_Nucleus_accumbens_basal_ganglia	3	*AP2M1*	183897178	rs4472033	1.0000	184459499	A	G	0.06	7.20E-06	3.05E-02	4.89E-02
rs11923588	Brain_Nucleus_accumbens_basal_ganglia	3	*AP2M1*	183897178	rs11923588	1.0000	184459667	T	C	0.06	5.66E-06	3.05E-02	4.86E-02
rs11923588	Brain_Putamen_basal_ganglia	3	*EPHB3*	184289884	rs9828886	0.9494	184449677	T	C	0.06	1.37E-05	2.89E-02	4.81E-02
rs11923588	Brain_Putamen_basal_ganglia	3	*RP11-433C9.2*	184138380	rs12635235	0.8823	184459005	G	C	0.05	9.09E-05	3.84E-02	7.12E-02
rs11923588	Brain_Putamen_basal_ganglia	3	*RP11-778D9.12*	183852416	rs4472033	1.0000	184459499	A	G	0.06	7.20E-06	4.80E-02	6.78E-02
rs11923588	Brain_Putamen_basal_ganglia	3	*EPHB3*	184289884	rs4472033	1.0000	184459499	A	G	0.06	7.20E-06	4.75E-02	6.73E-02
rs11923588	Brain_Putamen_basal_ganglia	3	*RP11-778D9.12*	183852416	rs11923588	1.0000	184459667	T	C	0.06	5.66E-06	4.80E-02	6.75E-02
rs11923588	Brain_Putamen_basal_ganglia	3	*EPHB3*	184289884	rs11923588	1.0000	184459667	T	C	0.06	5.66E-06	4.75E-02	6.70E-02
rs11923588	Brain_Spinal_cord_cervical_c-1	3	*ABCC5*	183686762	rs9828886	0.9494	184449677	T	C	0.06	1.37E-05	3.08E-02	5.03E-02
rs11923588	Brain_Spinal_cord_cervical_c-1	3	*CAMK2N2*	183978312	rs9828886	0.9494	184449677	T	C	0.06	1.37E-05	4.81E-02	6.90E-02
rs11923588	Brain_Spinal_cord_cervical_c-1	3	*YEATS2-AS1*	183526253	rs12635235	0.8823	184459005	G	C	0.05	9.09E-05	3.15E-02	6.34E-02
rs11923588	Brain_Spinal_cord_cervical_c-1	3	*ABCC5*	183686762	rs12635235	0.8823	184459005	G	C	0.05	9.09E-05	3.64E-02	6.90E-02
rs11923588	Brain_Spinal_cord_cervical_c-1	3	*CAMK2N2*	183978312	rs12635235	0.8823	184459005	G	C	0.05	9.09E-05	2.44E-02	5.48E-02
rs11923588	Brain_Spinal_cord_cervical_c-1	3	*CAMK2N2*	183978312	rs4472033	1.0000	184459499	A	G	0.06	7.20E-06	4.73E-02	6.71E-02
rs11923588	Brain_Spinal_cord_cervical_c-1	3	*CAMK2N2*	183978312	rs11923588	1.0000	184459667	T	C	0.06	5.66E-06	4.73E-02	6.68E-02
rs11923588	Brain_Substantia_nigra	3	*LIPH*	185247225	rs9828886	0.9494	184449677	T	C	0.06	1.37E-05	3.08E-02	5.03E-02
rs11923588	Brain_Substantia_nigra	3	*CAMK2N2*	183978312	rs4472033	1.0000	184459499	A	G	0.06	7.20E-06	4.15E-02	6.10E-02
rs11923588	Brain_Substantia_nigra	3	*LIPH*	185247225	rs4472033	1.0000	184459499	A	G	0.06	7.20E-06	3.57E-02	5.46E-02
rs11923588	Brain_Substantia_nigra	3	*CAMK2N2*	183978312	rs11923588	1.0000	184459667	T	C	0.06	5.66E-06	4.15E-02	6.07E-02
rs11923588	Brain_Substantia_nigra	3	*LIPH*	185247225	rs11923588	1.0000	184459667	T	C	0.06	5.66E-06	3.57E-02	5.43E-02
rs11923588	Whole_Blood	3	*YEATS2-AS1*	183526253	rs9828886	0.9494	184449677	T	C	0.06	1.37E-05	3.05E-02	4.99E-02
rs11923588	Whole_Blood	3	*CHRD*	184102739	rs9828886	0.9494	184449677	T	C	0.06	1.37E-05	1.15E-02	2.65E-02

rs11923588	Whole_Blood	3	*PARL*	183574934	rs12635235	0.8823	184459005	G	C	0.05	9.09E-05	2.42E-02	5.47E-02
rs11923588	Whole_Blood	3	*CHRD*	184102739	rs4472033	1.0000	184459499	A	G	0.06	7.20E-06	3.51E-02	5.40E-02
rs11923588	Whole_Blood	3	*CHRD*	184102739	rs11923588	1.0000	184459667	T	C	0.06	5.66E-06	3.51E-02	5.37E-02
rs66837203	Brain_Amygdala	4	*ARAP2*	36097987	s139213496	0.9382	36858139	C	A	0.06	7.97E-05	2.73E-02	5.94E-02
rs66837203	Brain_Amygdala	4	*ARAP2*	36097987	rs16992769	0.9382	36865768	A	G	0.06	1.00E-04	3.10E-02	6.42E-02
rs66837203	Brain_Amygdala	4	*ARAP2*	36097987	rs4832839	0.9382	36875527	A	T	0.06	1.00E-04	2.73E-02	5.99E-02
rs66837203	Brain_Amygdala	4	*ARAP2*	36097987	rs72624105	0.9382	36890300	C	G	0.06	7.11E-05	2.73E-02	5.87E-02
rs66837203	Brain_Amygdala	4	*ARAP2*	36097987	rs72624106	0.9382	36895800	T	G	0.06	9.17E-05	2.73E-02	5.94E-02
rs66837203	Brain_Cerebellum	4	*DTHD1*	36315311	s139213496	0.9382	36858139	C	A	0.06	7.97E-05	1.42E-02	4.22E-02
rs66837203	Brain_Cerebellum	4	*DTHD1*	36315311	rs16992769	0.9382	36865768	A	G	0.06	1.00E-04	1.14E-02	3.85E-02
rs66837203	Brain_Cerebellum	4	*DTHD1*	36315311	rs4832839	0.9382	36875527	A	T	0.06	1.00E-04	2.12E-02	5.23E-02
rs66837203	Brain_Cerebellum	4	*DTHD1*	36315311	rs72624105	0.9382	36890300	C	G	0.06	7.11E-05	3.03E-02	6.23E-02
rs66837203	Brain_Cerebellum	4	*DTHD1*	36315311	rs72624106	0.9382	36895800	T	G	0.06	9.17E-05	1.35E-02	4.13E-02
rs66837203	Brain_Cerebellum	4	*DTHD1*	36315311	rs66837203	1.0000	36897136	T	C	0.07	6.03E-06	1.20E-02	2.98E-02
rs66837203	Brain_Hypothalamus	4	*ARAP2*	36097987	s139213496	0.9382	36858139	C	A	0.06	7.97E-05	3.09E-02	6.37E-02
rs66837203	Brain_Hypothalamus	4	*ARAP2*	36097987	rs16992769	0.9382	36865768	A	G	0.06	1.00E-04	3.09E-02	6.42E-02
rs66837203	Brain_Hypothalamus	4	*ARAP2*	36097987	rs4832839	0.9382	36875527	A	T	0.06	1.00E-04	3.09E-02	6.42E-02
rs66837203	Brain_Hypothalamus	4	*ARAP2*	36097987	rs1978975	0.9588	36884958	A	G	0.07	2.36E-04	4.89E-02	9.28E-02
rs66837203	Brain_Hypothalamus	4	*ARAP2*	36097987	rs1982578	0.9588	36885972	T	G	0.07	2.33E-04	4.89E-02	9.27E-02
rs66837203	Brain_Hypothalamus	4	*ARAP2*	36097987	rs28872338	0.9004	36889599	G	A	0.07	1.82E-04	4.89E-02	9.08E-02
rs66837203	Brain_Hypothalamus	4	*ARAP2*	36097987	rs72624105	0.9382	36890300	C	G	0.06	7.11E-05	3.09E-02	6.30E-02
rs66837203	Brain_Hypothalamus	4	*ARAP2*	36097987	rs6817693	0.9588	36890895	T	G	0.07	2.09E-04	4.89E-02	9.18E-02
rs66837203	Brain_Hypothalamus	4	*ARAP2*	36097987	rs72624106	0.9382	36895800	T	G	0.06	9.17E-05	3.09E-02	6.36E-02
rs66837203	Brain_Hypothalamus	4	*ARAP2*	36097987	rs66837203	1.0000	36897136	T	C	0.07	6.03E-06	2.68E-03	1.36E-02
rs66837203	Brain_Nucleus_accumbens_basal_ganglia	4	*RP11-177C12.1*	37870756	rs1978975	0.9588	36884958	A	G	0.07	2.36E-04	2.52E-02	6.60E-02
rs66837203	Brain_Nucleus_accumbens_basal_ganglia	4	*RP11-177C12.1*	37870756	rs1982578	0.9588	36885972	T	G	0.07	2.33E-04	2.52E-02	6.59E-02
rs66837203	Brain_Nucleus_accumbens_basal_ganglia	4	*RP11-177C12.1*	37870756	rs28872338	0.9004	36889599	G	A	0.07	1.82E-04	2.36E-02	6.19E-02
rs66837203	Brain_Nucleus_accumbens_basal_ganglia	4	*RP11-177C12.1*	37870756	rs6817693	0.9588	36890895	T	G	0.07	2.09E-04	2.52E-02	6.50E-02
rs66837203	Brain_Putamen_basal_ganglia	4	*ARAP2*	36097987	s139213496	0.9382	36858139	C	A	0.06	7.97E-05	3.81E-02	7.19E-02
rs66837203	Brain_Putamen_basal_ganglia	4	*ARAP2*	36097987	rs16992769	0.9382	36865768	A	G	0.06	1.00E-04	3.81E-02	7.24E-02
rs66837203	Brain_Putamen_basal_ganglia	4	*ARAP2*	36097987	rs4832839	0.9382	36875527	A	T	0.06	1.00E-04	3.81E-02	7.24E-02
rs66837203	Brain_Putamen_basal_ganglia	4	*ARAP2*	36097987	rs72624105	0.9382	36890300	C	G	0.06	7.11E-05	3.81E-02	7.12E-02
rs66837203	Brain_Putamen_basal_ganglia	4	*ARAP2*	36097987	rs72624106	0.9382	36895800	T	G	0.06	9.17E-05	3.81E-02	7.19E-02
rs66837203	Brain_Putamen_basal_ganglia	4	*ARAP2*	36097987	rs66837203	1.0000	36897136	T	C	0.07	6.03E-06	4.88E-02	7.29E-02
rs66837203	Brain_Putamen_basal_ganglia	4	*RP11-431M7.3*	36260790	rs66837203	1.0000	36897136	T	C	0.07	6.03E-06	1.57E-02	3.49E-02
rs66837203	Brain_Substantia_nigra	4	*RP11-431M7.3*	36260790	s139213496	0.9382	36858139	C	A	0.06	7.97E-05	2.97E-02	6.23E-02
rs66837203	Brain_Substantia_nigra	4	*RP11-431M7.3*	36260790	rs16992769	0.9382	36865768	A	G	0.06	1.00E-04	3.20E-02	6.55E-02
rs66837203	Brain_Substantia_nigra	4	*RP11-431M7.3*	36260790	rs4832839	0.9382	36875527	A	T	0.06	1.00E-04	2.97E-02	6.28E-02
rs66837203	Brain_Substantia_nigra	4	*KIAA1239*	37348464	rs28872338	0.9004	36889599	G	A	0.07	1.82E-04	4.82E-02	9.01E-02
rs66837203	Brain_Substantia_nigra	4	*RP11-431M7.3*	36260790	rs72624105	0.9382	36890300	C	G	0.06	7.11E-05	2.97E-02	6.16E-02

rs66837203	Brain_Substantia_nigra	4	*RP11-431M7.3*	36260790	rs72624106	0.9382	36895800	T	G	0.06	9.17E-05	2.97E-02	6.22E-02
rs66837203	Brain_Substantia_nigra	4	*RP11-431M7.3*	36260790	rs66837203	1.0000	36897136	T	C	0.07	6.03E-06	2.53E-02	4.69E-02
rs200028958	Brain_Amygdala	4	*DCK*	71877443	rs17148162	0.9213	70974741	A	C	0.10	4.65E-04	3.09E-02	7.64E-02
rs200028958	Brain_Amygdala	4	*DCK*	71877443	rs4694043	0.9213	70975541	C	G	0.10	4.29E-04	3.09E-02	7.61E-02
rs200028958	Brain_Amygdala	4	*DCK*	71877443	rs28645990	0.9213	70977161	G	T	0.10	4.29E-04	3.09E-02	7.61E-02
rs200028958	Brain_Amygdala	4	*DCK*	71877443	rs55989074	0.9213	70982256	A	T	0.10	2.45E-04	3.09E-02	7.44E-02
rs200028958	Brain_Amygdala	4	*DCK*	71877443	rs138807640	0.9213	70984155	A	G	0.10	2.36E-04	3.09E-02	7.41E-02
rs200028958	Brain_Cerebellar_Hemisphere	4	*RP11-46J23.1*	71569836	rs9994880	0.8324	70811070	C	T	0.10	1.95E-04	4.02E-02	8.29E-02
rs200028958	Brain_Cerebellar_Hemisphere	4	*RP11-46J23.1*	71569836	rs10013848	0.8324	70816927	A	T	0.10	1.50E-04	3.92E-02	8.09E-02
rs200028958	Brain_Cerebellar_Hemisphere	4	*RP11-46J23.1*	71569836	rs2734570	0.8324	70821713	A	G	0.10	1.77E-04	3.78E-02	8.07E-02
rs200028958	Brain_Cerebellar_Hemisphere	4	*RP11-46J23.1*	71569836	rs2673720	0.8324	70825933	G	A	0.10	1.68E-04	3.92E-02	8.22E-02
rs200028958	Brain_Cerebellar_Hemisphere	4	*RP11-46J23.1*	71569836	rs2247569	0.8324	70826437	C	T	0.10	1.68E-04	3.92E-02	8.22E-02
rs200028958	Brain_Cerebellar_Hemisphere	4	*RP11-46J23.1*	71569836	rs186123891	0.8324	70828593	A	C	0.10	1.88E-04	4.73E-02	9.06E-02
rs200028958	Brain_Cerebellar_Hemisphere	4	*RP11-46J23.1*	71569836	rs28377851	0.8423	70831724	T	A	0.10	3.63E-04	1.32E-02	5.73E-02
rs200028958	Brain_Cerebellar_Hemisphere	4	*RP11-46J23.1*	71569836	rs35772950	0.8423	70832941	A	G	0.10	3.63E-04	1.32E-02	5.73E-02
rs200028958	Brain_Cerebellar_Hemisphere	4	*RP11-46J23.1*	71569836	rs28720737	0.8423	70833223	G	A	0.10	3.63E-04	1.76E-02	6.39E-02
rs200028958	Brain_Cerebellar_Hemisphere	4	*RP11-46J23.1*	71569836	rs10031238	0.8423	70833357	G	T	0.10	3.63E-04	1.74E-02	6.37E-02
rs200028958	Brain_Cerebellar_Hemisphere	4	*RP11-46J23.1*	71569836	rs9994584	0.8423	70833723	T	G	0.10	3.63E-04	1.32E-02	5.73E-02
rs200028958	Brain_Cerebellar_Hemisphere	4	*RP11-46J23.1*	71569836	rs10031845	0.8423	70834238	C	A	0.10	1.44E-04	3.92E-02	8.09E-02
rs200028958	Brain_Cerebellar_Hemisphere	4	*RP11-46J23.1*	71569836	rs17146478	0.8423	70834683	G	T	0.10	3.72E-04	1.46E-02	5.98E-02
rs200028958	Brain_Cerebellar_Hemisphere	4	*RP11-46J23.1*	71569836	rs79406241	0.8423	70834693	G	A	0.10	1.44E-04	4.35E-02	8.56E-02
rs200028958	Brain_Cerebellar_Hemisphere	4	*RP11-46J23.1*	71569836	rs17146480	0.8423	70834740	G	A	0.10	3.60E-04	1.46E-02	5.97E-02
rs200028958	Brain_Cerebellar_Hemisphere	4	*RP11-46J23.1*	71569836	rs59453376	0.8423	70834877	T	C	0.10	3.72E-04	1.32E-02	5.76E-02
rs200028958	Brain_Cerebellar_Hemisphere	4	*RP11-46J23.1*	71569836	rs10000684	0.8423	70835403	T	G	0.10	3.72E-04	1.32E-02	5.76E-02
rs200028958	Brain_Cerebellar_Hemisphere	4	*RP11-46J23.1*	71569836	rs10000776	0.8423	70835520	C	G	0.10	1.44E-04	3.92E-02	8.09E-02
rs200028958	Brain_Cerebellar_Hemisphere	4	*RP11-46J23.1*	71569836	rs28711127	0.8423	70836136	T	G	0.10	5.41E-04	3.92E-02	8.67E-02
rs200028958	Brain_Cerebellar_Hemisphere	4	*RP11-46J23.1*	71569836	rs28508161	0.8423	70836565	C	T	0.10	5.41E-04	3.92E-02	8.67E-02
rs200028958	Brain_Cerebellar_Hemisphere	4	*RP11-46J23.1*	71569836	rs28798425	0.8423	70838306	G	A	0.10	3.25E-04	3.92E-02	8.47E-02
rs200028958	Brain_Cerebellar_Hemisphere	4	*RP11-46J23.1*	71569836	rs4694261	0.8423	70839766	T	C	0.10	3.27E-04	3.92E-02	8.48E-02
rs200028958	Brain_Cerebellar_Hemisphere	4	*RP11-46J23.1*	71569836	rs4694262	0.8423	70839850	A	G	0.10	3.27E-04	3.92E-02	8.48E-02
rs200028958	Brain_Cerebellar_Hemisphere	4	*RP11-46J23.1*	71569836	rs7356202	0.8423	70840436	T	C	0.10	3.27E-04	4.70E-02	9.32E-02
rs200028958	Brain_Cerebellar_Hemisphere	4	*RP11-46J23.1*	71569836	rs77141872	0.8423	70841126	T	C	0.10	3.27E-04	3.92E-02	8.48E-02
rs200028958	Brain_Cerebellar_Hemisphere	4	*RP11-46J23.1*	71569836	rs12648752	0.8423	70841254	C	T	0.10	3.27E-04	3.92E-02	8.48E-02
rs200028958	Brain_Cerebellar_Hemisphere	4	*RP11-46J23.1*	71569836	rs28786470	0.8423	70842730	T	C	0.10	3.25E-04	3.92E-02	8.47E-02
rs200028958	Brain_Cerebellar_Hemisphere	4	*RP11-46J23.1*	71569836	rs17147891	0.8423	70849978	T	A	0.10	3.72E-04	3.92E-02	8.52E-02
rs200028958	Brain_Cerebellar_Hemisphere	4	*RP11-46J23.1*	71569836	rs28661681	0.8843	70854978	T	C	0.10	3.80E-04	3.92E-02	8.41E-02
rs200028958	Brain_Cerebellar_Hemisphere	4	*RP11-46J23.1*	71569836	rs4694263	0.8843	70862927	C	T	0.10	2.79E-04	3.92E-02	8.38E-02
rs200028958	Brain_Cerebellar_Hemisphere	4	*RP11-46J23.1*	71569836	rs3775764	0.8843	70864065	A	G	0.10	2.79E-04	3.92E-02	8.38E-02
rs200028958	Brain_Cerebellar_Hemisphere	4	*RP11-46J23.1*	71569836	rs1231535	0.8743	70875649	T	C	0.13	8.58E-05	1.43E-02	4.54E-02
rs200028958	Brain_Cerebellar_Hemisphere	4	*RP11-46J23.1*	71569836	rs776837	0.8743	70876153	C	T	0.13	6.46E-05	1.56E-02	4.55E-02

rs200028958	Brain_Cerebellar_Hemisphere	4	*RP11-46J23.1*	71569836	rs1606878	0.8843	70876844	A	T	0.10	2.79E-04	3.92E-02	8.38E-02
rs200028958	Brain_Cerebellar_Hemisphere	4	*RP11-46J23.1*	71569836	rs28799286	0.8843	70878782	T	C	0.10	2.79E-04	4.43E-02	8.94E-02
rs200028958	Brain_Cerebellar_Hemisphere	4	*RP11-46J23.1*	71569836	rs79704056	0.8843	70885815	G	A	0.10	2.93E-04	2.37E-02	6.61E-02
rs200028958	Brain_Cerebellar_Hemisphere	4	*RP11-46J23.1*	71569836	rs1849937	0.8843	70898907	T	C	0.10	2.93E-04	2.59E-02	6.88E-02
rs200028958	Brain_Cerebellar_Hemisphere	4	*RP11-46J23.1*	71569836	rs28707754	0.8843	70900864	T	C	0.10	2.67E-04	3.92E-02	8.34E-02
rs200028958	Brain_Cerebellar_Hemisphere	4	*RP11-46J23.1*	71569836	rs4694040	0.8843	70901653	A	G	0.10	2.67E-04	3.85E-02	8.26E-02
rs200028958	Brain_Cerebellar_Hemisphere	4	*RP11-46J23.1*	71569836	rs7654757	0.8843	70902412	T	C	0.10	2.67E-04	3.92E-02	8.34E-02
rs200028958	Brain_Cerebellar_Hemisphere	4	*RP11-46J23.1*	71569836	rs13434441	0.9206	70905397	C	T	0.10	1.93E-04	2.37E-02	6.31E-02
rs200028958	Brain_Cerebellar_Hemisphere	4	*RP11-46J23.1*	71569836	rs28643541	0.9407	70912133	A	G	0.11	7.92E-05	4.21E-02	7.78E-02
rs200028958	Brain_Cerebellar_Hemisphere	4	*RP11-46J23.1*	71569836	rs6857697	0.9407	70914609	A	C	0.11	6.25E-05	4.21E-02	7.64E-02
rs200028958	Brain_Cerebellar_Hemisphere	4	*RP11-46J23.1*	71569836	rs3862054	0.9407	70916273	T	C	0.10	1.52E-04	2.90E-02	6.85E-02
rs200028958	Brain_Cerebellar_Hemisphere	4	*RP11-46J23.1*	71569836	rs6818370	0.9307	70921069	T	G	0.10	2.23E-05	4.35E-02	7.50E-02
rs200028958	Brain_Cerebellar_Hemisphere	4	*RP11-46J23.1*	71569836	rs10019826	0.9407	70922064	A	G	0.11	4.30E-05	4.21E-02	7.64E-02
rs200028958	Brain_Cerebellar_Hemisphere	4	*RP11-46J23.1*	71569836	rs28869909	0.9407	70922226	C	A	0.10	1.09E-04	2.90E-02	6.72E-02
rs200028958	Brain_Cerebellar_Hemisphere	4	*RP11-46J23.1*	71569836	rs115749891	1.0000	70923658	A	G	0.10	8.26E-05	2.90E-02	6.96E-02
rs200028958	Brain_Cerebellar_Hemisphere	4	*RP11-46J23.1*	71569836	rs200028958	1.0000	70923661	A	G	0.10	6.25E-06	2.90E-02	5.35E-02
rs200028958	Brain_Cerebellar_Hemisphere	4	*RP11-46J23.1*	71569836	rs201171800	1.0000	70923663	A	G	0.10	6.25E-06	2.90E-02	5.35E-02
rs200028958	Brain_Cerebellar_Hemisphere	4	*RP11-46J23.1*	71569836	rs202245754	1.0000	70923665	C	A	0.10	6.25E-06	2.90E-02	5.35E-02
rs200028958	Brain_Cerebellar_Hemisphere	4	*RP11-46J23.1*	71569836	rs200349247	1.0000	70923666	A	T	0.10	6.25E-06	2.90E-02	5.35E-02
rs200028958	Brain_Cerebellar_Hemisphere	4	*RP11-46J23.1*	71569836	rs28790602	0.9407	70928040	A	G	0.10	1.10E-04	2.90E-02	6.73E-02
rs200028958	Brain_Cerebellar_Hemisphere	4	*RP11-46J23.1*	71569836	rs4694265	0.9407	70928571	A	G	0.10	1.10E-04	2.90E-02	6.73E-02
rs200028958	Brain_Cerebellar_Hemisphere	4	*RP11-46J23.1*	71569836	rs28635879	0.9407	70929763	T	C	0.10	1.22E-04	1.78E-02	5.39E-02
rs200028958	Brain_Cerebellar_Hemisphere	4	*RP11-46J23.1*	71569836	rs28622022	0.9407	70929889	T	A	0.10	1.12E-04	2.90E-02	6.74E-02
rs200028958	Brain_Cerebellar_Hemisphere	4	*RP11-46J23.1*	71569836	rs6832546	0.9407	70930584	T	G	0.10	1.22E-04	1.78E-02	5.39E-02
rs200028958	Brain_Cerebellar_Hemisphere	4	*RP11-46J23.1*	71569836	rs17148034	0.9122	70937524	G	C	0.10	2.93E-04	2.90E-02	7.05E-02
rs200028958	Brain_Cerebellar_Hemisphere	4	*RP11-46J23.1*	71569836	rs7660022	0.9314	70943619	A	C	0.10	2.25E-04	1.78E-02	5.61E-02
rs200028958	Brain_Cerebellar_Hemisphere	4	*RP11-46J23.1*	71569836	rs10004471	0.9314	70950312	C	A	0.10	2.25E-04	2.82E-02	6.95E-02
rs200028958	Brain_Cerebellar_Hemisphere	4	*RP11-46J23.1*	71569836	rs28489946	0.9314	70952099	C	T	0.10	2.25E-04	1.78E-02	5.61E-02
rs200028958	Brain_Cerebellar_Hemisphere	4	*RP11-46J23.1*	71569836	rs7666225	0.9314	70959484	C	T	0.10	2.24E-04	1.23E-02	4.79E-02
rs200028958	Brain_Cerebellar_Hemisphere	4	*RP11-46J23.1*	71569836	rs4694041	0.9314	70961196	G	C	0.10	2.24E-04	1.78E-02	5.60E-02
rs200028958	Brain_Cerebellar_Hemisphere	4	*RP11-46J23.1*	71569836	rs28434698	0.9314	70965054	C	T	0.10	2.10E-04	1.78E-02	5.57E-02
rs200028958	Brain_Cerebellar_Hemisphere	4	*RP11-46J23.1*	71569836	rs12331580	0.9314	70973499	A	C	0.10	2.87E-04	2.33E-02	6.41E-02
rs200028958	Brain_Cerebellar_Hemisphere	4	*RP11-46J23.1*	71569836	rs17148162	0.9213	70974741	A	C	0.10	4.65E-04	4.75E-02	9.48E-02
rs200028958	Brain_Cerebellar_Hemisphere	4	*RP11-46J23.1*	71569836	rs4694043	0.9213	70975541	C	G	0.10	4.29E-04	3.73E-02	8.34E-02
rs200028958	Brain_Cerebellar_Hemisphere	4	*RP11-46J23.1*	71569836	rs28645990	0.9213	70977161	G	T	0.10	4.29E-04	3.25E-02	7.79E-02
rs200028958	Brain_Cerebellar_Hemisphere	4	*RP11-46J23.1*	71569836	rs17148181	0.9213	70981462	T	C	0.10	3.67E-04	2.86E-02	7.17E-02
rs200028958	Brain_Cerebellar_Hemisphere	4	*RP11-46J23.1*	71569836	rs55989074	0.9213	70982256	A	T	0.10	2.45E-04	3.25E-02	7.62E-02
rs200028958	Brain_Cerebellar_Hemisphere	4	*RP11-46J23.1*	71569836	rs138807640	0.9213	70984155	A	G	0.10	2.36E-04	3.25E-02	7.59E-02
rs200028958	Brain_Cerebellar_Hemisphere	4	*RP11-46J23.1*	71569836	rs116572259	0.9213	70984463	C	T	0.10	1.99E-04	2.86E-02	6.97E-02
rs200028958	Brain_Cerebellar_Hemisphere	4	*RP11-46J23.1*	71569836	rs10011994	0.9021	70987026	A	T	0.10	1.78E-04	2.86E-02	6.91E-02

rs200028958	Brain_Cerebellar_Hemisphere	4	*RP11-46J23.1*	71569836	rs28866208	0.9021	70987317	G	A	0.10	1.78E-04	2.85E-02	6.91E-02
rs200028958	Brain_Cerebellar_Hemisphere	4	*RP11-46J23.1*	71569836	rs7356162	0.9021	70987982	G	T	0.11	4.61E-04	4.65E-02	9.48E-02
rs200028958	Brain_Cerebellar_Hemisphere	4	*RP11-46J23.1*	71569836	rs1607989	0.9021	70990224	G	A	0.11	3.41E-04	4.30E-02	8.96E-02
rs200028958	Brain_Cerebellar_Hemisphere	4	*RP11-46J23.1*	71569836	rs10007698	0.9021	70990879	C	T	0.10	1.26E-04	1.02E-02	4.24E-02
rs200028958	Brain_Cerebellum	4	*UTP3*	71555231	rs776837	0.8743	70876153	C	T	0.13	6.46E-05	4.08E-02	7.62E-02
rs200028958	Brain_Cortex	4	*RUFY3*	71621775	rs1231535	0.8743	70875649	T	C	0.13	8.58E-05	2.74E-02	6.26E-02
rs200028958	Brain_Cortex	4	*RUFY3*	71621775	rs776837	0.8743	70876153	C	T	0.13	6.46E-05	4.33E-02	7.90E-02
rs200028958	Brain_Cortex	4	*RUFY3*	71621775	rs12331580	0.9314	70973499	A	C	0.10	2.87E-04	4.70E-02	9.13E-02
rs200028958	Brain_Cortex	4	*RUFY3*	71621775	rs17148184	0.9213	70981602	T	A	0.10	4.29E-04	4.32E-02	8.99E-02
rs200028958	Brain_Frontal_Cortex_BA9	4	*RP11-46J23.1*	71569836	rs10031238	0.8423	70833357	G	T	0.10	3.63E-04	4.06E-02	9.20E-02
rs200028958	Brain_Hippocampus	4	*MOB1B*	71810964	rs7660022	0.9314	70943619	A	C	0.10	2.25E-04	4.04E-02	8.35E-02
rs200028958	Brain_Hippocampus	4	*MOB1B*	71810964	rs28489946	0.9314	70952099	C	T	0.10	2.25E-04	4.04E-02	8.35E-02
rs200028958	Brain_Hippocampus	4	*MOB1B*	71810964	rs7666225	0.9314	70959484	C	T	0.10	2.24E-04	2.66E-02	6.75E-02
rs200028958	Brain_Hippocampus	4	*MOB1B*	71810964	rs4694041	0.9314	70961196	G	C	0.10	2.24E-04	4.04E-02	8.35E-02
rs200028958	Brain_Hippocampus	4	*MOB1B*	71810964	rs28434698	0.9314	70965054	C	T	0.10	2.10E-04	4.04E-02	8.31E-02
rs200028958	Brain_Hippocampus	4	*MOB1B*	71810964	rs12331580	0.9314	70973499	A	C	0.10	2.87E-04	4.99E-02	9.44E-02
rs200028958	Brain_Hippocampus	4	*RP11-46J23.1*	71569836	rs17148184	0.9213	70981602	T	A	0.10	4.29E-04	3.72E-02	8.33E-02
rs200028958	Brain_Hypothalamus	4	*UTP3*	71555231	rs2029572	0.8324	70810063	A	G	0.10	1.75E-04	3.57E-02	7.76E-02
rs200028958	Brain_Hypothalamus	4	*UTP3*	71555231	rs9994880	0.8324	70811070	C	T	0.10	1.95E-04	1.85E-02	5.66E-02
rs200028958	Brain_Hypothalamus	4	*UTP3*	71555231	rs9992853	0.8324	70811521	G	A	0.10	1.95E-04	1.60E-02	5.30E-02
rs200028958	Brain_Hypothalamus	4	*UTP3*	71555231	rs10013848	0.8324	70816927	A	T	0.10	1.50E-04	1.85E-02	5.57E-02
rs200028958	Brain_Hypothalamus	4	*UTP3*	71555231	rs2734570	0.8324	70821713	A	G	0.10	1.77E-04	1.79E-02	5.62E-02
rs200028958	Brain_Hypothalamus	4	*UTP3*	71555231	rs2673720	0.8324	70825933	G	A	0.10	1.68E-04	1.78E-02	5.60E-02
rs200028958	Brain_Hypothalamus	4	*UTP3*	71555231	rs2247569	0.8324	70826437	C	T	0.10	1.68E-04	1.85E-02	5.70E-02
rs200028958	Brain_Hypothalamus	4	*UTP3*	71555231	rs186123891	0.8324	70828593	A	C	0.10	1.88E-04	1.88E-02	5.70E-02
rs200028958	Brain_Hypothalamus	4	*UTP3*	71555231	rs28377851	0.8423	70831724	T	A	0.10	3.63E-04	2.68E-02	7.60E-02
rs200028958	Brain_Hypothalamus	4	*UTP3*	71555231	rs35772950	0.8423	70832941	A	G	0.10	3.63E-04	2.68E-02	7.60E-02
rs200028958	Brain_Hypothalamus	4	*UTP3*	71555231	rs28720737	0.8423	70833223	G	A	0.10	3.63E-04	2.68E-02	7.60E-02
rs200028958	Brain_Hypothalamus	4	*UTP3*	71555231	rs10031238	0.8423	70833357	G	T	0.10	3.63E-04	1.94E-02	6.65E-02
rs200028958	Brain_Hypothalamus	4	*UTP3*	71555231	rs9994584	0.8423	70833723	T	G	0.10	3.63E-04	2.68E-02	7.60E-02
rs200028958	Brain_Hypothalamus	4	*UTP3*	71555231	rs10031845	0.8423	70834238	C	A	0.10	1.44E-04	1.68E-02	5.33E-02
rs200028958	Brain_Hypothalamus	4	*RUFY3*	71621775	rs10031845	0.8423	70834238	C	A	0.10	1.44E-04	4.77E-02	9.01E-02
rs200028958	Brain_Hypothalamus	4	*UTP3*	71555231	rs17146478	0.8423	70834683	G	T	0.10	3.72E-04	4.80E-02	1.00E-01
rs200028958	Brain_Hypothalamus	4	*UTP3*	71555231	rs79406241	0.8423	70834693	G	A	0.10	1.44E-04	3.29E-02	7.37E-02
rs200028958	Brain_Hypothalamus	4	*UTP3*	71555231	rs17146480	0.8423	70834740	G	A	0.10	3.60E-04	4.80E-02	1.00E-01
rs200028958	Brain_Hypothalamus	4	*UTP3*	71555231	rs59453376	0.8423	70834877	T	C	0.10	3.72E-04	2.68E-02	7.64E-02
rs200028958	Brain_Hypothalamus	4	*UTP3*	71555231	rs10000684	0.8423	70835403	T	G	0.10	3.72E-04	2.68E-02	7.64E-02
rs200028958	Brain_Hypothalamus	4	*UTP3*	71555231	rs10000776	0.8423	70835520	C	G	0.10	1.44E-04	1.68E-02	5.33E-02
rs200028958	Brain_Hypothalamus	4	*RUFY3*	71621775	rs10000776	0.8423	70835520	C	G	0.10	1.44E-04	4.77E-02	9.01E-02
rs200028958	Brain_Hypothalamus	4	*UTP3*	71555231	rs28711127	0.8423	70836136	T	G	0.10	5.41E-04	1.68E-02	5.89E-02

rs20002895ɛ	Brain_Hypothalamus	4	*RUFY3*	71621775	rs28711127	0.8423	70836136	T	G	0.10	5.41E-04	4.77E-02	9.58E-02
rs20002895ɛ	Brain_Hypothalamus	4	*UTP3*	71555231	rs28508161	0.8423	70836565	C	T	0.10	5.41E-04	1.68E-02	5.89E-02
rs20002895ɛ	Brain_Hypothalamus	4	*RUFY3*	71621775	rs28508161	0.8423	70836565	C	T	0.10	5.41E-04	4.77E-02	9.58E-02
rs20002895ɛ	Brain_Hypothalamus	4	*UTP3*	71555231	rs28798425	0.8423	70838306	G	A	0.10	3.25E-04	1.68E-02	5.70E-02
rs20002895ɛ	Brain_Hypothalamus	4	*RUFY3*	71621775	rs28798425	0.8423	70838306	G	A	0.10	3.25E-04	4.77E-02	9.39E-02
rs20002895ɛ	Brain_Hypothalamus	4	*UTP3*	71555231	rs4694261	0.8423	70839766	T	C	0.10	3.27E-04	1.68E-02	5.70E-02
rs20002895ɛ	Brain_Hypothalamus	4	*RUFY3*	71621775	rs4694261	0.8423	70839766	T	C	0.10	3.27E-04	4.77E-02	9.40E-02
rs20002895ɛ	Brain_Hypothalamus	4	*UTP3*	71555231	rs4694262	0.8423	70839850	A	G	0.10	3.27E-04	1.68E-02	5.70E-02
rs20002895ɛ	Brain_Hypothalamus	4	*RUFY3*	71621775	rs4694262	0.8423	70839850	A	G	0.10	3.27E-04	4.77E-02	9.40E-02
rs20002895ɛ	Brain_Hypothalamus	4	*UTP3*	71555231	rs7356202	0.8423	70840436	T	C	0.10	3.27E-04	1.43E-02	5.34E-02
rs20002895ɛ	Brain_Hypothalamus	4	*RUFY3*	71621775	rs7356202	0.8423	70840436	T	C	0.10	3.27E-04	4.39E-02	8.99E-02
rs20002895ɛ	Brain_Hypothalamus	4	*UTP3*	71555231	rs77141872	0.8423	70841126	T	C	0.10	3.27E-04	1.68E-02	5.70E-02
rs20002895ɛ	Brain_Hypothalamus	4	*RUFY3*	71621775	rs77141872	0.8423	70841126	T	C	0.10	3.27E-04	4.77E-02	9.40E-02
rs20002895ɛ	Brain_Hypothalamus	4	*UTP3*	71555231	rs12648752	0.8423	70841254	C	T	0.10	3.27E-04	1.68E-02	5.70E-02
rs20002895ɛ	Brain_Hypothalamus	4	*RUFY3*	71621775	rs12648752	0.8423	70841254	C	T	0.10	3.27E-04	4.77E-02	9.40E-02
rs20002895ɛ	Brain_Hypothalamus	4	*UTP3*	71555231	rs28786470	0.8423	70842730	T	C	0.10	3.25E-04	1.68E-02	5.70E-02
rs20002895ɛ	Brain_Hypothalamus	4	*RUFY3*	71621775	rs28786470	0.8423	70842730	T	C	0.10	3.25E-04	4.77E-02	9.39E-02
rs20002895ɛ	Brain_Hypothalamus	4	*UTP3*	71555231	rs17147891	0.8423	70849978	T	A	0.10	3.72E-04	1.68E-02	5.75E-02
rs20002895ɛ	Brain_Hypothalamus	4	*RUFY3*	71621775	rs17147891	0.8423	70849978	T	A	0.10	3.72E-04	4.77E-02	9.44E-02
rs20002895ɛ	Brain_Hypothalamus	4	*UTP3*	71555231	rs28661681	0.8843	70854978	T	C	0.10	3.80E-04	1.68E-02	5.64E-02
rs20002895ɛ	Brain_Hypothalamus	4	*RUFY3*	71621775	rs28661681	0.8843	70854978	T	C	0.10	3.80E-04	4.77E-02	9.33E-02
rs20002895ɛ	Brain_Hypothalamus	4	*UTP3*	71555231	rs4694263	0.8843	70862927	C	T	0.10	2.79E-04	1.68E-02	5.61E-02
rs20002895ɛ	Brain_Hypothalamus	4	*RUFY3*	71621775	rs4694263	0.8843	70862927	C	T	0.10	2.79E-04	4.77E-02	9.30E-02
rs20002895ɛ	Brain_Hypothalamus	4	*DCK*	71877443	rs4694263	0.8843	70862927	C	T	0.10	2.79E-04	7.65E-03	4.12E-02
rs20002895ɛ	Brain_Hypothalamus	4	*UTP3*	71555231	rs3775764	0.8843	70864065	A	G	0.10	2.79E-04	1.68E-02	5.61E-02
rs20002895ɛ	Brain_Hypothalamus	4	*RUFY3*	71621775	rs3775764	0.8843	70864065	A	G	0.10	2.79E-04	4.77E-02	9.30E-02
rs20002895ɛ	Brain_Hypothalamus	4	*DCK*	71877443	rs3775764	0.8843	70864065	A	G	0.10	2.79E-04	7.65E-03	4.12E-02
rs20002895ɛ	Brain_Hypothalamus	4	*UTP3*	71555231	rs1231535	0.8743	70875649	T	C	0.13	8.58E-05	4.81E-02	8.59E-02
rs20002895ɛ	Brain_Hypothalamus	4	*UTP3*	71555231	rs776837	0.8743	70876153	C	T	0.13	6.46E-05	3.11E-02	6.53E-02
rs20002895ɛ	Brain_Hypothalamus	4	*DCK*	71877443	rs776837	0.8743	70876153	C	T	0.13	6.46E-05	1.77E-02	4.84E-02
rs20002895ɛ	Brain_Hypothalamus	4	*UTP3*	71555231	rs1606878	0.8843	70876844	A	T	0.10	2.79E-04	1.68E-02	5.61E-02
rs20002895ɛ	Brain_Hypothalamus	4	*RUFY3*	71621775	rs1606878	0.8843	70876844	A	T	0.10	2.79E-04	4.77E-02	9.30E-02
rs20002895ɛ	Brain_Hypothalamus	4	*DCK*	71877443	rs1606878	0.8843	70876844	A	T	0.10	2.79E-04	7.65E-03	4.12E-02
rs20002895ɛ	Brain_Hypothalamus	4	*UTP3*	71555231	rs28799286	0.8843	70878782	T	C	0.10	2.79E-04	3.29E-02	7.66E-02
rs20002895ɛ	Brain_Hypothalamus	4	*DCK*	71877443	rs28799286	0.8843	70878782	T	C	0.10	2.79E-04	7.00E-03	3.99E-02
rs20002895ɛ	Brain_Hypothalamus	4	*UTP3*	71555231	rs1083135	0.8843	70881127	C	T	0.11	8.15E-05	1.17E-02	3.98E-02
rs20002895ɛ	Brain_Hypothalamus	4	*RUFY3*	71621775	rs1083135	0.8843	70881127	C	T	0.11	8.15E-05	2.46E-02	5.75E-02
rs20002895ɛ	Brain_Hypothalamus	4	*DCK*	71877443	rs1083135	0.8843	70881127	C	T	0.11	8.15E-05	9.45E-03	3.61E-02
rs20002895ɛ	Brain_Hypothalamus	4	*UTP3*	71555231	rs79704056	0.8843	70885815	G	A	0.10	2.93E-04	1.17E-02	4.90E-02
rs20002895ɛ	Brain_Hypothalamus	4	*RUFY3*	71621775	rs79704056	0.8843	70885815	G	A	0.10	2.93E-04	2.46E-02	6.72E-02

rs200028958	Brain_Hypothalamus	4	*DCK*	71877443	rs79704056	0.8843	70885815	G	A	0.10	2.93E-04	9.45E-03	4.51E-02
rs200028958	Brain_Hypothalamus	4	*UTP3*	71555231	rs776841	0.8843	70889307	C	G	0.11	8.15E-05	1.17E-02	3.98E-02
rs200028958	Brain_Hypothalamus	4	*RUFY3*	71621775	rs776841	0.8843	70889307	C	G	0.11	8.15E-05	2.46E-02	5.75E-02
rs200028958	Brain_Hypothalamus	4	*DCK*	71877443	rs776841	0.8843	70889307	C	G	0.11	8.15E-05	9.45E-03	3.61E-02
rs200028958	Brain_Hypothalamus	4	*UTP3*	71555231	rs10017681	0.8843	70889847	G	A	0.10	2.67E-04	3.18E-02	7.50E-02
rs200028958	Brain_Hypothalamus	4	*RUFY3*	71621775	rs10017681	0.8843	70889847	G	A	0.10	2.67E-04	4.28E-02	8.73E-02
rs200028958	Brain_Hypothalamus	4	*DCK*	71877443	rs10017681	0.8843	70889847	G	A	0.10	2.67E-04	1.10E-02	4.69E-02
rs200028958	Brain_Hypothalamus	4	*UTP3*	71555231	rs800756	0.8843	70897507	C	T	0.11	8.15E-05	1.17E-02	3.98E-02
rs200028958	Brain_Hypothalamus	4	*RUFY3*	71621775	rs800756	0.8843	70897507	C	T	0.11	8.15E-05	2.46E-02	5.75E-02
rs200028958	Brain_Hypothalamus	4	*DCK*	71877443	rs800756	0.8843	70897507	C	T	0.11	8.15E-05	9.45E-03	3.61E-02
rs200028958	Brain_Hypothalamus	4	*UTP3*	71555231	rs1849937	0.8843	70898907	T	C	0.10	2.93E-04	2.34E-02	6.57E-02
rs200028958	Brain_Hypothalamus	4	*RUFY3*	71621775	rs1849937	0.8843	70898907	T	C	0.10	2.93E-04	3.10E-02	7.51E-02
rs200028958	Brain_Hypothalamus	4	*DCK*	71877443	rs1849937	0.8843	70898907	T	C	0.10	2.93E-04	8.85E-03	4.40E-02
rs200028958	Brain_Hypothalamus	4	*UTP3*	71555231	rs28707754	0.8843	70900864	T	C	0.10	2.67E-04	1.68E-02	5.58E-02
rs200028958	Brain_Hypothalamus	4	*RUFY3*	71621775	rs28707754	0.8843	70900864	T	C	0.10	2.67E-04	4.77E-02	9.26E-02
rs200028958	Brain_Hypothalamus	4	*DCK*	71877443	rs28707754	0.8843	70900864	T	C	0.10	2.67E-04	7.65E-03	4.08E-02
rs200028958	Brain_Hypothalamus	4	*UTP3*	71555231	rs4694040	0.8843	70901653	A	G	0.10	2.67E-04	1.60E-02	5.46E-02
rs200028958	Brain_Hypothalamus	4	*RUFY3*	71621775	rs4694040	0.8843	70901653	A	G	0.10	2.67E-04	4.38E-02	8.84E-02
rs200028958	Brain_Hypothalamus	4	*DCK*	71877443	rs4694040	0.8843	70901653	A	G	0.10	2.67E-04	7.47E-03	4.05E-02
rs200028958	Brain_Hypothalamus	4	*UTP3*	71555231	rs7654757	0.8843	70902412	T	C	0.10	2.67E-04	1.68E-02	5.58E-02
rs200028958	Brain_Hypothalamus	4	*RUFY3*	71621775	rs7654757	0.8843	70902412	T	C	0.10	2.67E-04	4.77E-02	9.26E-02
rs200028958	Brain_Hypothalamus	4	*DCK*	71877443	rs7654757	0.8843	70902412	T	C	0.10	2.67E-04	7.65E-03	4.08E-02
rs200028958	Brain_Hypothalamus	4	*DCK*	71877443	rs148867082	0.9206	70903438	T	C	0.11	3.76E-05	1.52E-03	1.58E-02
rs200028958	Brain_Hypothalamus	4	*DCK*	71877443	rs146349142	0.9206	70903526	G	A	0.11	3.37E-05	3.64E-03	2.16E-02
rs200028958	Brain_Hypothalamus	4	*UTP3*	71555231	rs6855909	0.9206	70904992	C	A	0.10	5.05E-05	1.17E-02	3.79E-02
rs200028958	Brain_Hypothalamus	4	*RUFY3*	71621775	rs6855909	0.9206	70904992	C	A	0.10	5.05E-05	2.46E-02	5.54E-02
rs200028958	Brain_Hypothalamus	4	*DCK*	71877443	rs6855909	0.9206	70904992	C	A	0.10	5.05E-05	9.45E-03	3.42E-02
rs200028958	Brain_Hypothalamus	4	*UTP3*	71555231	rs13434441	0.9206	70905397	C	T	0.10	1.93E-04	1.17E-02	4.61E-02
rs200028958	Brain_Hypothalamus	4	*RUFY3*	71621775	rs13434441	0.9206	70905397	C	T	0.10	1.93E-04	2.46E-02	6.42E-02
rs200028958	Brain_Hypothalamus	4	*DCK*	71877443	rs13434441	0.9206	70905397	C	T	0.10	1.93E-04	9.45E-03	4.23E-02
rs200028958	Brain_Hypothalamus	4	*UTP3*	71555231	rs6843256	0.9113	70906988	A	G	0.11	4.05E-05	4.85E-02	8.16E-02
rs200028958	Brain_Hypothalamus	4	*DCK*	71877443	rs6843256	0.9113	70906988	A	G	0.11	4.05E-05	3.36E-03	2.15E-02
rs200028958	Brain_Hypothalamus	4	*UTP3*	71555231	rs6845010	0.9206	70907119	A	C	0.10	3.01E-05	1.17E-02	3.62E-02
rs200028958	Brain_Hypothalamus	4	*RUFY3*	71621775	rs6845010	0.9206	70907119	A	C	0.10	3.01E-05	2.46E-02	5.35E-02
rs200028958	Brain_Hypothalamus	4	*DCK*	71877443	rs6845010	0.9206	70907119	A	C	0.10	3.01E-05	9.45E-03	3.26E-02
rs200028958	Brain_Hypothalamus	4	*UTP3*	71555231	rs28643541	0.9407	70912133	A	G	0.11	7.92E-05	8.36E-03	3.43E-02
rs200028958	Brain_Hypothalamus	4	*RUFY3*	71621775	rs28643541	0.9407	70912133	A	G	0.11	7.92E-05	1.40E-02	4.34E-02
rs200028958	Brain_Hypothalamus	4	*DCK*	71877443	rs28643541	0.9407	70912133	A	G	0.11	7.92E-05	3.47E-03	2.41E-02
rs200028958	Brain_Hypothalamus	4	*UTP3*	71555231	rs6857697	0.9407	70914609	A	C	0.11	6.25E-05	1.02E-02	3.61E-02
rs200028958	Brain_Hypothalamus	4	*RUFY3*	71621775	rs6857697	0.9407	70914609	A	C	0.11	6.25E-05	1.45E-02	4.27E-02

rs200028958	Brain_Hypothalamus	4	*DCK*	71877443	rs6857697	0.9407	70914609	A	C	0.11	6.25E-05	1.50E-02	4.35E-02
rs200028958	Brain_Hypothalamus	4	*UTP3*	71555231	rs3862054	0.9407	70916273	T	C	0.10	1.52E-04	1.41E-02	4.88E-02
rs200028958	Brain_Hypothalamus	4	*RUFY3*	71621775	rs3862054	0.9407	70916273	T	C	0.10	1.52E-04	2.78E-02	6.70E-02
rs200028958	Brain_Hypothalamus	4	*DCK*	71877443	rs3862054	0.9407	70916273	T	C	0.10	1.52E-04	1.25E-02	4.63E-02
rs200028958	Brain_Hypothalamus	4	*UTP3*	71555231	rs6818370	0.9307	70921069	T	G	0.10	2.23E-05	1.06E-02	3.42E-02
rs200028958	Brain_Hypothalamus	4	*RUFY3*	71621775	rs6818370	0.9307	70921069	T	G	0.10	2.23E-05	1.85E-02	4.56E-02
rs200028958	Brain_Hypothalamus	4	*DCK*	71877443	rs6818370	0.9307	70921069	T	G	0.10	2.23E-05	3.30E-02	6.33E-02
rs200028958	Brain_Hypothalamus	4	*UTP3*	71555231	rs10019826	0.9407	70922064	A	G	0.11	4.30E-05	8.36E-03	3.31E-02
rs200028958	Brain_Hypothalamus	4	*RUFY3*	71621775	rs10019826	0.9407	70922064	A	G	0.11	4.30E-05	1.40E-02	4.20E-02
rs200028958	Brain_Hypothalamus	4	*DCK*	71877443	rs10019826	0.9407	70922064	A	G	0.11	4.30E-05	3.47E-03	2.30E-02
rs200028958	Brain_Hypothalamus	4	*UTP3*	71555231	rs28869909	0.9407	70922226	C	A	0.10	1.09E-04	1.41E-02	4.76E-02
rs200028958	Brain_Hypothalamus	4	*RUFY3*	71621775	rs28869909	0.9407	70922226	C	A	0.10	1.09E-04	2.78E-02	6.57E-02
rs200028958	Brain_Hypothalamus	4	*DCK*	71877443	rs28869909	0.9407	70922226	C	A	0.10	1.09E-04	1.25E-02	4.51E-02
rs200028958	Brain_Hypothalamus	4	*UTP3*	71555231	rs115749891	1.0000	70923658	A	G	0.10	8.26E-05	1.41E-02	4.99E-02
rs200028958	Brain_Hypothalamus	4	*RUFY3*	71621775	rs115749891	1.0000	70923658	A	G	0.10	8.26E-05	2.78E-02	6.81E-02
rs200028958	Brain_Hypothalamus	4	*DCK*	71877443	rs115749891	1.0000	70923658	A	G	0.10	8.26E-05	1.25E-02	4.73E-02
rs200028958	Brain_Hypothalamus	4	*UTP3*	71555231	rs200028958	1.0000	70923661	A	G	0.10	6.25E-06	1.41E-02	3.49E-02
rs200028958	Brain_Hypothalamus	4	*RUFY3*	71621775	rs200028958	1.0000	70923661	A	G	0.10	6.25E-06	2.78E-02	5.21E-02
rs200028958	Brain_Hypothalamus	4	*DCK*	71877443	rs200028958	1.0000	70923661	A	G	0.10	6.25E-06	1.25E-02	3.26E-02
rs200028958	Brain_Hypothalamus	4	*UTP3*	71555231	rs201171800	1.0000	70923663	A	G	0.10	6.25E-06	1.41E-02	3.49E-02
rs200028958	Brain_Hypothalamus	4	*RUFY3*	71621775	rs201171800	1.0000	70923663	A	G	0.10	6.25E-06	2.78E-02	5.21E-02
rs200028958	Brain_Hypothalamus	4	*DCK*	71877443	rs201171800	1.0000	70923663	A	G	0.10	6.25E-06	1.25E-02	3.26E-02
rs200028958	Brain_Hypothalamus	4	*UTP3*	71555231	rs202245754	1.0000	70923665	C	A	0.10	6.25E-06	1.41E-02	3.49E-02
rs200028958	Brain_Hypothalamus	4	*RUFY3*	71621775	rs202245754	1.0000	70923665	C	A	0.10	6.25E-06	2.78E-02	5.21E-02
rs200028958	Brain_Hypothalamus	4	*DCK*	71877443	rs202245754	1.0000	70923665	C	A	0.10	6.25E-06	1.25E-02	3.26E-02
rs200028958	Brain_Hypothalamus	4	*UTP3*	71555231	rs200349247	1.0000	70923666	A	T	0.10	6.25E-06	1.41E-02	3.49E-02
rs200028958	Brain_Hypothalamus	4	*RUFY3*	71621775	rs200349247	1.0000	70923666	A	T	0.10	6.25E-06	2.78E-02	5.21E-02
rs200028958	Brain_Hypothalamus	4	*DCK*	71877443	rs200349247	1.0000	70923666	A	T	0.10	6.25E-06	1.25E-02	3.26E-02
rs200028958	Brain_Hypothalamus	4	*UTP3*	71555231	rs28790602	0.9407	70928040	A	G	0.10	1.10E-04	1.54E-02	4.95E-02
rs200028958	Brain_Hypothalamus	4	*RUFY3*	71621775	rs28790602	0.9407	70928040	A	G	0.10	1.10E-04	3.00E-02	6.84E-02
rs200028958	Brain_Hypothalamus	4	*DCK*	71877443	rs28790602	0.9407	70928040	A	G	0.10	1.10E-04	1.21E-02	4.45E-02
rs200028958	Brain_Hypothalamus	4	*UTP3*	71555231	rs4694265	0.9407	70928571	A	G	0.10	1.10E-04	1.41E-02	4.77E-02
rs200028958	Brain_Hypothalamus	4	*RUFY3*	71621775	rs4694265	0.9407	70928571	A	G	0.10	1.10E-04	2.78E-02	6.58E-02
rs200028958	Brain_Hypothalamus	4	*DCK*	71877443	rs4694265	0.9407	70928571	A	G	0.10	1.10E-04	1.25E-02	4.52E-02
rs200028958	Brain_Hypothalamus	4	*UTP3*	71555231	rs28635879	0.9407	70929763	T	C	0.10	1.22E-04	1.02E-02	4.22E-02
rs200028958	Brain_Hypothalamus	4	*RUFY3*	71621775	rs28635879	0.9407	70929763	T	C	0.10	1.22E-04	1.45E-02	4.90E-02
rs200028958	Brain_Hypothalamus	4	*DCK*	71877443	rs28635879	0.9407	70929763	T	C	0.10	1.22E-04	1.50E-02	4.98E-02
rs200028958	Brain_Hypothalamus	4	*UTP3*	71555231	rs28622022	0.9407	70929889	T	A	0.10	1.12E-04	1.11E-02	4.31E-02
rs200028958	Brain_Hypothalamus	4	*RUFY3*	71621775	rs28622022	0.9407	70929889	T	A	0.10	1.12E-04	2.41E-02	6.15E-02
rs200028958	Brain_Hypothalamus	4	*DCK*	71877443	rs28622022	0.9407	70929889	T	A	0.10	1.12E-04	7.30E-03	3.63E-02

rs200028958	Brain_Hypothalamus	4	*UTP3*	71555231	rs6832546	0.9407	70930584	T	G	0.10	1.22E-04	1.02E-02	4.22E-02
rs200028958	Brain_Hypothalamus	4	*RUFY3*	71621775	rs6832546	0.9407	70930584	T	G	0.10	1.22E-04	1.45E-02	4.90E-02
rs200028958	Brain_Hypothalamus	4	*DCK*	71877443	rs6832546	0.9407	70930584	T	G	0.10	1.22E-04	1.50E-02	4.98E-02
rs200028958	Brain_Hypothalamus	4	*UTP3*	71555231	rs17148034	0.9122	70937524	G	C	0.10	2.93E-04	1.02E-02	4.45E-02
rs200028958	Brain_Hypothalamus	4	*RUFY3*	71621775	rs17148034	0.9122	70937524	G	C	0.10	2.93E-04	2.35E-02	6.37E-02
rs200028958	Brain_Hypothalamus	4	*DCK*	71877443	rs17148034	0.9122	70937524	G	C	0.10	2.93E-04	6.59E-03	3.77E-02
rs200028958	Brain_Hypothalamus	4	*UTP3*	71555231	rs7660022	0.9314	70943619	A	C	0.10	2.25E-04	7.88E-03	4.01E-02
rs200028958	Brain_Hypothalamus	4	*RUFY3*	71621775	rs7660022	0.9314	70943619	A	C	0.10	2.25E-04	1.24E-02	4.80E-02
rs200028958	Brain_Hypothalamus	4	*DCK*	71877443	rs7660022	0.9314	70943619	A	C	0.10	2.25E-04	8.49E-03	4.13E-02
rs200028958	Brain_Hypothalamus	4	*UTP3*	71555231	rs10004471	0.9314	70950312	C	A	0.10	2.25E-04	1.72E-02	5.52E-02
rs200028958	Brain_Hypothalamus	4	*RUFY3*	71621775	rs10004471	0.9314	70950312	C	A	0.10	2.25E-04	1.75E-02	5.56E-02
rs200028958	Brain_Hypothalamus	4	*DCK*	71877443	rs10004471	0.9314	70950312	C	A	0.10	2.25E-04	1.36E-02	4.99E-02
rs200028958	Brain_Hypothalamus	4	*UTP3*	71555231	rs28489946	0.9314	70952099	C	T	0.10	2.25E-04	7.88E-03	4.01E-02
rs200028958	Brain_Hypothalamus	4	*RUFY3*	71621775	rs28489946	0.9314	70952099	C	T	0.10	2.25E-04	1.24E-02	4.80E-02
rs200028958	Brain_Hypothalamus	4	*DCK*	71877443	rs28489946	0.9314	70952099	C	T	0.10	2.25E-04	8.49E-03	4.13E-02
rs200028958	Brain_Hypothalamus	4	*UTP3*	71555231	rs7666225	0.9314	70959484	C	T	0.10	2.24E-04	1.25E-02	4.81E-02
rs200028958	Brain_Hypothalamus	4	*RUFY3*	71621775	rs7666225	0.9314	70959484	C	T	0.10	2.24E-04	1.94E-02	5.82E-02
rs200028958	Brain_Hypothalamus	4	*DCK*	71877443	rs7666225	0.9314	70959484	C	T	0.10	2.24E-04	1.36E-02	4.99E-02
rs200028958	Brain_Hypothalamus	4	*UTP3*	71555231	rs4694041	0.9314	70961196	G	C	0.10	2.24E-04	7.88E-03	4.01E-02
rs200028958	Brain_Hypothalamus	4	*RUFY3*	71621775	rs4694041	0.9314	70961196	G	C	0.10	2.24E-04	1.24E-02	4.80E-02
rs200028958	Brain_Hypothalamus	4	*DCK*	71877443	rs4694041	0.9314	70961196	G	C	0.10	2.24E-04	8.49E-03	4.13E-02
rs200028958	Brain_Hypothalamus	4	*UTP3*	71555231	rs28434698	0.9314	70965054	C	T	0.10	2.10E-04	7.88E-03	3.98E-02
rs200028958	Brain_Hypothalamus	4	*RUFY3*	71621775	rs28434698	0.9314	70965054	C	T	0.10	2.10E-04	1.24E-02	4.76E-02
rs200028958	Brain_Hypothalamus	4	*DCK*	71877443	rs28434698	0.9314	70965054	C	T	0.10	2.10E-04	8.49E-03	4.09E-02
rs200028958	Brain_Hypothalamus	4	*UTP3*	71555231	rs12331580	0.9314	70973499	A	C	0.10	2.87E-04	6.41E-03	3.78E-02
rs200028958	Brain_Hypothalamus	4	*RUFY3*	71621775	rs12331580	0.9314	70973499	A	C	0.10	2.87E-04	1.33E-02	5.01E-02
rs200028958	Brain_Hypothalamus	4	*DCK*	71877443	rs12331580	0.9314	70973499	A	C	0.10	2.87E-04	2.23E-03	2.69E-02
rs200028958	Brain_Hypothalamus	4	*UTP3*	71555231	rs17148162	0.9213	70974741	A	C	0.10	4.65E-04	1.11E-02	4.94E-02
rs200028958	Brain_Hypothalamus	4	*RUFY3*	71621775	rs17148162	0.9213	70974741	A	C	0.10	4.65E-04	2.98E-02	7.51E-02
rs200028958	Brain_Hypothalamus	4	*DCK*	71877443	rs17148162	0.9213	70974741	A	C	0.10	4.65E-04	6.53E-03	4.08E-02
rs200028958	Brain_Hypothalamus	4	*UTP3*	71555231	rs4694043	0.9213	70975541	C	G	0.10	4.29E-04	2.53E-02	6.92E-02
rs200028958	Brain_Hypothalamus	4	*RUFY3*	71621775	rs4694043	0.9213	70975541	C	G	0.10	4.29E-04	3.22E-02	7.76E-02
rs200028958	Brain_Hypothalamus	4	*DCK*	71877443	rs4694043	0.9213	70975541	C	G	0.10	4.29E-04	5.02E-03	3.71E-02
rs200028958	Brain_Hypothalamus	4	*UTP3*	71555231	rs28645990	0.9213	70977161	G	T	0.10	4.29E-04	1.36E-02	5.31E-02
rs200028958	Brain_Hypothalamus	4	*RUFY3*	71621775	rs28645990	0.9213	70977161	G	T	0.10	4.29E-04	2.65E-02	7.07E-02
rs200028958	Brain_Hypothalamus	4	*DCK*	71877443	rs28645990	0.9213	70977161	G	T	0.10	4.29E-04	5.62E-03	3.85E-02
rs200028958	Brain_Hypothalamus	4	*UTP3*	71555231	rs17148181	0.9213	70981462	T	C	0.10	3.67E-04	1.09E-02	4.72E-02
rs200028958	Brain_Hypothalamus	4	*RUFY3*	71621775	rs17148181	0.9213	70981462	T	C	0.10	3.67E-04	2.41E-02	6.61E-02
rs200028958	Brain_Hypothalamus	4	*DCK*	71877443	rs17148181	0.9213	70981462	T	C	0.10	3.67E-04	6.74E-03	3.95E-02
rs200028958	Brain_Hypothalamus	4	*UTP3*	71555231	rs17148184	0.9213	70981602	T	A	0.10	4.29E-04	3.01E-02	7.50E-02

rs200028958	Brain_Hypothalamus	4	*RUFY3*	71621775	rs17148184	0.9213	70981602	T	A	0.10	4.29E-04	2.30E-02	6.63E-02
rs200028958	Brain_Hypothalamus	4	*DCK*	71877443	rs17148184	0.9213	70981602	T	A	0.10	4.29E-04	1.04E-02	4.77E-02
rs200028958	Brain_Hypothalamus	4	*UTP3*	71555231	rs55989074	0.9213	70982256	A	T	0.10	2.45E-04	1.36E-02	5.15E-02
rs200028958	Brain_Hypothalamus	4	*RUFY3*	71621775	rs55989074	0.9213	70982256	A	T	0.10	2.45E-04	2.65E-02	6.91E-02
rs200028958	Brain_Hypothalamus	4	*DCK*	71877443	rs55989074	0.9213	70982256	A	T	0.10	2.45E-04	5.62E-03	3.70E-02
rs200028958	Brain_Hypothalamus	4	*UTP3*	71555231	s138807640	0.9213	70984155	A	G	0.10	2.36E-04	1.36E-02	5.11E-02
rs200028958	Brain_Hypothalamus	4	*RUFY3*	71621775	s138807640	0.9213	70984155	A	G	0.10	2.36E-04	2.65E-02	6.87E-02
rs200028958	Brain_Hypothalamus	4	*DCK*	71877443	s138807640	0.9213	70984155	A	G	0.10	2.36E-04	5.62E-03	3.67E-02
rs200028958	Brain_Hypothalamus	4	*UTP3*	71555231	s116572259	0.9213	70984463	C	T	0.10	1.99E-04	1.09E-02	4.53E-02
rs200028958	Brain_Hypothalamus	4	*RUFY3*	71621775	s116572259	0.9213	70984463	C	T	0.10	1.99E-04	2.41E-02	6.41E-02
rs200028958	Brain_Hypothalamus	4	*DCK*	71877443	s116572259	0.9213	70984463	C	T	0.10	1.99E-04	6.74E-03	3.76E-02
rs200028958	Brain_Hypothalamus	4	*UTP3*	71555231	rs10011994	0.9021	70987026	A	T	0.10	1.78E-04	1.09E-02	4.48E-02
rs200028958	Brain_Hypothalamus	4	*RUFY3*	71621775	rs10011994	0.9021	70987026	A	T	0.10	1.78E-04	2.41E-02	6.35E-02
rs200028958	Brain_Hypothalamus	4	*DCK*	71877443	rs10011994	0.9021	70987026	A	T	0.10	1.78E-04	6.74E-03	3.72E-02
rs200028958	Brain_Hypothalamus	4	*UTP3*	71555231	rs28866208	0.9021	70987317	G	A	0.10	1.78E-04	1.09E-02	4.48E-02
rs200028958	Brain_Hypothalamus	4	*RUFY3*	71621775	rs28866208	0.9021	70987317	G	A	0.10	1.78E-04	2.41E-02	6.35E-02
rs200028958	Brain_Hypothalamus	4	*DCK*	71877443	rs28866208	0.9021	70987317	G	A	0.10	1.78E-04	6.74E-03	3.72E-02
rs200028958	Brain_Hypothalamus	4	*UTP3*	71555231	rs7356162	0.9021	70987982	G	T	0.11	4.61E-04	2.94E-02	7.57E-02
rs200028958	Brain_Hypothalamus	4	*RUFY3*	71621775	rs7356162	0.9021	70987982	G	T	0.11	4.61E-04	4.66E-02	9.49E-02
rs200028958	Brain_Hypothalamus	4	*DCK*	71877443	rs7356162	0.9021	70987982	G	T	0.11	4.61E-04	2.02E-02	6.39E-02
rs200028958	Brain_Hypothalamus	4	*UTP3*	71555231	rs28870732	0.9021	70989432	A	C	0.11	4.67E-04	2.30E-02	6.78E-02
rs200028958	Brain_Hypothalamus	4	*RUFY3*	71621775	rs28870732	0.9021	70989432	A	C	0.11	4.67E-04	4.88E-02	9.74E-02
rs200028958	Brain_Hypothalamus	4	*DCK*	71877443	rs28870732	0.9021	70989432	A	C	0.11	4.67E-04	2.44E-02	6.96E-02
rs200028958	Brain_Hypothalamus	4	*UTP3*	71555231	rs28793967	0.9021	70989703	T	C	0.11	4.67E-04	2.30E-02	6.78E-02
rs200028958	Brain_Hypothalamus	4	*RUFY3*	71621775	rs28793967	0.9021	70989703	T	C	0.11	4.67E-04	4.88E-02	9.74E-02
rs200028958	Brain_Hypothalamus	4	*DCK*	71877443	rs28793967	0.9021	70989703	T	C	0.11	4.67E-04	2.44E-02	6.96E-02
rs200028958	Brain_Hypothalamus	4	*UTP3*	71555231	rs1607989	0.9021	70990224	G	A	0.11	3.41E-04	1.88E-02	6.08E-02
rs200028958	Brain_Hypothalamus	4	*DCK*	71877443	rs1607989	0.9021	70990224	G	A	0.11	3.41E-04	2.97E-02	7.47E-02
rs200028958	Brain_Hypothalamus	4	*UTP3*	71555231	rs10007698	0.9021	70990879	C	T	0.10	1.26E-04	3.86E-02	7.94E-02
rs200028958	Brain_Hypothalamus	4	*RUFY3*	71621775	rs10007698	0.9021	70990879	C	T	0.10	1.26E-04	2.01E-02	5.71E-02
rs200028958	Brain_Hypothalamus	4	*DCK*	71877443	rs10007698	0.9021	70990879	C	T	0.10	1.26E-04	1.24E-02	4.60E-02
rs200028958	Brain_Nucleus_accumbens_basal_ganglia	4	*MOB1B*	71810964	rs10004471	0.9314	70950312	C	A	0.10	2.25E-04	3.85E-02	8.13E-02
rs200028958	Brain_Nucleus_accumbens_basal_ganglia	4	*MOB1B*	71810964	rs7666225	0.9314	70959484	C	T	0.10	2.24E-04	3.30E-02	7.51E-02
rs200028958	Brain_Nucleus_accumbens_basal_ganglia	4	*DCK*	71877443	rs4694043	0.9213	70975541	C	G	0.10	4.29E-04	3.12E-02	7.63E-02
rs200028958	Brain_Nucleus_accumbens_basal_ganglia	4	*DCK*	71877443	rs28645990	0.9213	70977161	G	T	0.10	4.29E-04	3.01E-02	7.51E-02
rs200028958	Brain_Nucleus_accumbens_basal_ganglia	4	*DCK*	71877443	rs10007698	0.9021	70990879	C	T	0.10	1.26E-04	4.10E-02	8.20E-02
rs200028958	Brain_Putamen_basal_ganglia	4	*UTP3*	71555231	rs1231535	0.8743	70875649	T	C	0.13	8.58E-05	3.71E-02	7.39E-02
rs200028958	Brain_Spinal_cord_cervical_c-1	4	*UTP3*	71555231	rs2029572	0.8324	70810063	A	G	0.10	1.75E-04	1.96E-02	5.79E-02
rs200028958	Brain_Spinal_cord_cervical_c-1	4	*UTP3*	71555231	rs9994880	0.8324	70811070	C	T	0.10	1.95E-04	8.00E-03	4.00E-02
rs200028958	Brain_Spinal_cord_cervical_c-1	4	*RP11-46J23.1*	71569836	rs9994880	0.8324	70811070	C	T	0.10	1.95E-04	3.72E-02	7.96E-02

rs200028958	Brain_Spinal_cord_cervical_c-1	4	*UTP3*	71555231	rs9992853	0.8324	70811521	G	A	0.10	1.95E-04	8.00E-03	4.00E-02
rs200028958	Brain_Spinal_cord_cervical_c-1	4	*RP11-46J23.1*	71569836	rs9992853	0.8324	70811521	G	A	0.10	1.95E-04	3.72E-02	7.96E-02
rs200028958	Brain_Spinal_cord_cervical_c-1	4	*UTP3*	71555231	rs10013848	0.8324	70816927	A	T	0.10	1.50E-04	8.00E-03	3.92E-02
rs200028958	Brain_Spinal_cord_cervical_c-1	4	*RP11-46J23.1*	71569836	rs10013848	0.8324	70816927	A	T	0.10	1.50E-04	3.72E-02	7.86E-02
rs200028958	Brain_Spinal_cord_cervical_c-1	4	*UTP3*	71555231	rs2637816	0.8324	70821025	G	A	0.10	1.77E-04	3.58E-02	7.85E-02
rs200028958	Brain_Spinal_cord_cervical_c-1	4	*UTP3*	71555231	rs2734570	0.8324	70821713	A	G	0.10	1.77E-04	9.27E-03	4.28E-02
rs200028958	Brain_Spinal_cord_cervical_c-1	4	*RP11-46J23.1*	71569836	rs2734570	0.8324	70821713	A	G	0.10	1.77E-04	3.47E-02	7.73E-02
rs200028958	Brain_Spinal_cord_cervical_c-1	4	*UTP3*	71555231	rs2673720	0.8324	70825933	G	A	0.10	1.68E-04	1.01E-02	4.41E-02
rs200028958	Brain_Spinal_cord_cervical_c-1	4	*RP11-46J23.1*	71569836	rs2673720	0.8324	70825933	G	A	0.10	1.68E-04	3.50E-02	7.74E-02
rs200028958	Brain_Spinal_cord_cervical_c-1	4	*UTP3*	71555231	rs2247569	0.8324	70826437	C	T	0.10	1.68E-04	8.00E-03	4.03E-02
rs200028958	Brain_Spinal_cord_cervical_c-1	4	*RP11-46J23.1*	71569836	rs2247569	0.8324	70826437	C	T	0.10	1.68E-04	3.72E-02	7.99E-02
rs200028958	Brain_Spinal_cord_cervical_c-1	4	*UTP3*	71555231	rs186123891	0.8324	70828593	A	C	0.10	1.88E-04	1.77E-02	5.55E-02
rs200028958	Brain_Spinal_cord_cervical_c-1	4	*RP11-46J23.1*	71569836	rs28377851	0.8423	70831724	T	A	0.10	3.63E-04	3.92E-02	9.06E-02
rs200028958	Brain_Spinal_cord_cervical_c-1	4	*RP11-46J23.1*	71569836	rs35772950	0.8423	70832941	A	G	0.10	3.63E-04	3.92E-02	9.06E-02
rs200028958	Brain_Spinal_cord_cervical_c-1	4	*RP11-46J23.1*	71569836	rs28720737	0.8423	70833223	G	A	0.10	3.63E-04	4.37E-02	9.55E-02
rs200028958	Brain_Spinal_cord_cervical_c-1	4	*UTP3*	71555231	rs10031238	0.8423	70833357	G	T	0.10	3.63E-04	4.06E-02	9.21E-02
rs200028958	Brain_Spinal_cord_cervical_c-1	4	*RP11-46J23.1*	71569836	rs10031238	0.8423	70833357	G	T	0.10	3.63E-04	4.53E-02	9.72E-02
rs200028958	Brain_Spinal_cord_cervical_c-1	4	*RP11-46J23.1*	71569836	rs9994584	0.8423	70833723	T	G	0.10	3.63E-04	3.92E-02	9.06E-02
rs200028958	Brain_Spinal_cord_cervical_c-1	4	*UTP3*	71555231	rs10031845	0.8423	70834238	C	A	0.10	1.44E-04	3.93E-02	8.10E-02
rs200028958	Brain_Spinal_cord_cervical_c-1	4	*RP11-46J23.1*	71569836	rs10031845	0.8423	70834238	C	A	0.10	1.44E-04	2.67E-02	6.64E-02
rs200028958	Brain_Spinal_cord_cervical_c-1	4	*RP11-46J23.1*	71569836	rs79406241	0.8423	70834693	G	A	0.10	1.44E-04	3.68E-02	7.82E-02
rs200028958	Brain_Spinal_cord_cervical_c-1	4	*RP11-46J23.1*	71569836	rs59453376	0.8423	70834877	T	C	0.10	3.72E-04	3.92E-02	9.09E-02
rs200028958	Brain_Spinal_cord_cervical_c-1	4	*RP11-46J23.1*	71569836	rs10000684	0.8423	70835403	T	G	0.10	3.72E-04	3.92E-02	9.09E-02
rs200028958	Brain_Spinal_cord_cervical_c-1	4	*UTP3*	71555231	rs10000776	0.8423	70835520	C	G	0.10	1.44E-04	3.93E-02	8.10E-02
rs200028958	Brain_Spinal_cord_cervical_c-1	4	*RP11-46J23.1*	71569836	rs10000776	0.8423	70835520	C	G	0.10	1.44E-04	2.67E-02	6.64E-02
rs200028958	Brain_Spinal_cord_cervical_c-1	4	*UTP3*	71555231	rs28711127	0.8423	70836136	T	G	0.10	5.41E-04	3.93E-02	8.67E-02
rs200028958	Brain_Spinal_cord_cervical_c-1	4	*RP11-46J23.1*	71569836	rs28711127	0.8423	70836136	T	G	0.10	5.41E-04	2.67E-02	7.21E-02
rs200028958	Brain_Spinal_cord_cervical_c-1	4	*UTP3*	71555231	rs28508161	0.8423	70836565	C	T	0.10	5.41E-04	4.46E-02	9.25E-02
rs200028958	Brain_Spinal_cord_cervical_c-1	4	*RP11-46J23.1*	71569836	rs28508161	0.8423	70836565	C	T	0.10	5.41E-04	4.29E-02	9.06E-02
rs200028958	Brain_Spinal_cord_cervical_c-1	4	*UTP3*	71555231	rs28798425	0.8423	70838306	G	A	0.10	3.25E-04	3.93E-02	8.48E-02
rs200028958	Brain_Spinal_cord_cervical_c-1	4	*RP11-46J23.1*	71569836	rs28798425	0.8423	70838306	G	A	0.10	3.25E-04	2.67E-02	7.02E-02
rs200028958	Brain_Spinal_cord_cervical_c-1	4	*UTP3*	71555231	rs4694261	0.8423	70839766	T	C	0.10	3.27E-04	3.93E-02	8.48E-02
rs200028958	Brain_Spinal_cord_cervical_c-1	4	*RP11-46J23.1*	71569836	rs4694261	0.8423	70839766	T	C	0.10	3.27E-04	2.67E-02	7.02E-02
rs200028958	Brain_Spinal_cord_cervical_c-1	4	*UTP3*	71555231	rs4694262	0.8423	70839850	A	G	0.10	3.27E-04	3.93E-02	8.48E-02
rs200028958	Brain_Spinal_cord_cervical_c-1	4	*RP11-46J23.1*	71569836	rs4694262	0.8423	70839850	A	G	0.10	3.27E-04	2.67E-02	7.02E-02
rs200028958	Brain_Spinal_cord_cervical_c-1	4	*UTP3*	71555231	rs7356202	0.8423	70840436	T	C	0.10	3.27E-04	3.74E-02	8.27E-02
rs200028958	Brain_Spinal_cord_cervical_c-1	4	*RP11-46J23.1*	71569836	rs7356202	0.8423	70840436	T	C	0.10	3.27E-04	2.80E-02	7.18E-02
rs200028958	Brain_Spinal_cord_cervical_c-1	4	*UTP3*	71555231	rs77141872	0.8423	70841126	T	C	0.10	3.27E-04	3.93E-02	8.48E-02
rs200028958	Brain_Spinal_cord_cervical_c-1	4	*RP11-46J23.1*	71569836	rs77141872	0.8423	70841126	T	C	0.10	3.27E-04	2.67E-02	7.02E-02
rs200028958	Brain_Spinal_cord_cervical_c-1	4	*UTP3*	71555231	rs12648752	0.8423	70841254	C	T	0.10	3.27E-04	3.93E-02	8.48E-02

rs200028958	Brain_Spinal_cord_cervical_c-1	4	*RP11-46J23.1*	71569836	rs12648752	0.8423	70841254	C	T	0.10	3.27E-04	2.67E-02	7.02E-02
rs200028958	Brain_Spinal_cord_cervical_c-1	4	*UTP3*	71555231	rs28786470	0.8423	70842730	T	C	0.10	3.25E-04	3.93E-02	8.48E-02
rs200028958	Brain_Spinal_cord_cervical_c-1	4	*RP11-46J23.1*	71569836	rs28786470	0.8423	70842730	T	C	0.10	3.25E-04	2.67E-02	7.02E-02
rs200028958	Brain_Spinal_cord_cervical_c-1	4	*UTP3*	71555231	rs17147891	0.8423	70849978	T	A	0.10	3.72E-04	3.93E-02	8.53E-02
rs200028958	Brain_Spinal_cord_cervical_c-1	4	*RP11-46J23.1*	71569836	rs17147891	0.8423	70849978	T	A	0.10	3.72E-04	2.67E-02	7.07E-02
rs200028958	Brain_Spinal_cord_cervical_c-1	4	*RP11-46J23.1*	71569836	rs28661681	0.8843	70854978	T	C	0.10	3.80E-04	3.98E-02	8.48E-02
rs200028958	Brain_Spinal_cord_cervical_c-1	4	*RP11-46J23.1*	71569836	rs4694263	0.8843	70862927	C	T	0.10	2.79E-04	3.98E-02	8.45E-02
rs200028958	Brain_Spinal_cord_cervical_c-1	4	*RP11-46J23.1*	71569836	rs3775764	0.8843	70864065	A	G	0.10	2.79E-04	3.98E-02	8.45E-02
rs200028958	Brain_Spinal_cord_cervical_c-1	4	*RP11-46J23.1*	71569836	rs1606878	0.8843	70876844	A	T	0.10	2.79E-04	3.98E-02	8.45E-02
rs200028958	Brain_Spinal_cord_cervical_c-1	4	*RP11-46J23.1*	71569836	rs1083135	0.8843	70881127	C	T	0.11	8.15E-05	4.41E-02	7.99E-02
rs200028958	Brain_Spinal_cord_cervical_c-1	4	*RP11-46J23.1*	71569836	rs79704056	0.8843	70885815	G	A	0.10	2.93E-04	3.98E-02	8.50E-02
rs200028958	Brain_Spinal_cord_cervical_c-1	4	*RP11-46J23.1*	71569836	rs776841	0.8843	70889307	C	G	0.11	8.15E-05	4.41E-02	7.99E-02
rs200028958	Brain_Spinal_cord_cervical_c-1	4	*RP11-46J23.1*	71569836	rs800756	0.8843	70897507	C	T	0.11	8.15E-05	4.41E-02	7.99E-02
rs200028958	Brain_Spinal_cord_cervical_c-1	4	*RP11-46J23.1*	71569836	rs28707754	0.8843	70900864	T	C	0.10	2.67E-04	3.98E-02	8.41E-02
rs200028958	Brain_Spinal_cord_cervical_c-1	4	*RP11-46J23.1*	71569836	rs4694040	0.8843	70901653	A	G	0.10	2.67E-04	4.72E-02	9.20E-02
rs200028958	Brain_Spinal_cord_cervical_c-1	4	*RP11-46J23.1*	71569836	rs7654757	0.8843	70902412	T	C	0.10	2.67E-04	3.98E-02	8.41E-02
rs200028958	Brain_Spinal_cord_cervical_c-1	4	*RP11-46J23.1*	71569836	rs148867082	0.9206	70903438	T	C	0.11	3.76E-05	3.35E-02	6.50E-02
rs200028958	Brain_Spinal_cord_cervical_c-1	4	*RP11-46J23.1*	71569836	rs6855909	0.9206	70904992	C	A	0.10	5.05E-05	4.92E-02	8.32E-02
rs200028958	Brain_Spinal_cord_cervical_c-1	4	*RP11-46J23.1*	71569836	rs13434441	0.9206	70905397	C	T	0.10	1.93E-04	4.57E-02	8.84E-02
rs200028958	Brain_Spinal_cord_cervical_c-1	4	*RP11-46J23.1*	71569836	rs6845010	0.9206	70907119	A	C	0.10	3.01E-05	4.92E-02	8.12E-02
rs200028958	Brain_Spinal_cord_cervical_c-1	4	*UTP3*	71555231	rs6818370	0.9307	70921069	T	G	0.10	2.23E-05	3.35E-02	6.39E-02
rs200028958	Brain_Substantia_nigra	4	*RUFY3*	71621775	rs2029572	0.8324	70810063	A	G	0.10	1.75E-04	1.12E-02	4.54E-02
rs200028958	Brain_Substantia_nigra	4	*RUFY3*	71621775	rs9994880	0.8324	70811070	C	T	0.10	1.95E-04	2.33E-02	6.29E-02
rs200028958	Brain_Substantia_nigra	4	*RUFY3*	71621775	rs9992853	0.8324	70811521	G	A	0.10	1.95E-04	2.38E-02	6.37E-02
rs200028958	Brain_Substantia_nigra	4	*RUFY3*	71621775	rs10013848	0.8324	70816927	A	T	0.10	1.50E-04	2.38E-02	6.28E-02
rs200028958	Brain_Substantia_nigra	4	*RUFY3*	71621775	rs2637816	0.8324	70821025	G	A	0.10	1.77E-04	1.12E-02	4.61E-02
rs200028958	Brain_Substantia_nigra	4	*RUFY3*	71621775	rs2734570	0.8324	70821713	A	G	0.10	1.77E-04	1.97E-02	5.88E-02
rs200028958	Brain_Substantia_nigra	4	*RUFY3*	71621775	rs2673720	0.8324	70825933	G	A	0.10	1.68E-04	2.38E-02	6.40E-02
rs200028958	Brain_Substantia_nigra	4	*RUFY3*	71621775	rs2247569	0.8324	70826437	C	T	0.10	1.68E-04	2.38E-02	6.40E-02
rs200028958	Brain_Substantia_nigra	4	*RUFY3*	71621775	rs186123891	0.8324	70828593	A	C	0.10	1.88E-04	4.52E-02	8.83E-02
rs200028958	Brain_Substantia_nigra	4	*RUFY3*	71621775	rs28377851	0.8423	70831724	T	A	0.10	3.63E-04	2.07E-02	6.82E-02
rs200028958	Brain_Substantia_nigra	4	*RUFY3*	71621775	rs35772950	0.8423	70832941	A	G	0.10	3.63E-04	2.07E-02	6.82E-02
rs200028958	Brain_Substantia_nigra	4	*RUFY3*	71621775	rs28720737	0.8423	70833223	G	A	0.10	3.63E-04	2.19E-02	6.98E-02
rs200028958	Brain_Substantia_nigra	4	*RUFY3*	71621775	rs10031238	0.8423	70833357	G	T	0.10	3.63E-04	2.00E-02	6.72E-02
rs200028958	Brain_Substantia_nigra	4	*RUFY3*	71621775	rs9994584	0.8423	70833723	T	G	0.10	3.63E-04	2.11E-02	6.88E-02
rs200028958	Brain_Substantia_nigra	4	*RUFY3*	71621775	rs10031845	0.8423	70834238	C	A	0.10	1.44E-04	2.02E-02	5.81E-02
rs200028958	Brain_Substantia_nigra	4	*RUFY3*	71621775	rs17146478	0.8423	70834683	G	T	0.10	3.72E-04	2.07E-02	6.85E-02
rs200028958	Brain_Substantia_nigra	4	*RUFY3*	71621775	rs79406241	0.8423	70834693	G	A	0.10	1.44E-04	1.97E-02	5.74E-02
rs200028958	Brain_Substantia_nigra	4	*RUFY3*	71621775	rs17146480	0.8423	70834740	G	A	0.10	3.60E-04	2.07E-02	6.84E-02
rs200028958	Brain_Substantia_nigra	4	*RUFY3*	71621775	rs59453376	0.8423	70834877	T	C	0.10	3.72E-04	2.07E-02	6.85E-02

rs20002895	Brain_Substantia_nigra	4	*RUFY3*	71621775	rs10000684	0.8423	70835403	T	G	0.10	3.72E-04	2.07E-02	6.85E-02
rs20002895	Brain_Substantia_nigra	4	*RUFY3*	71621775	rs10000776	0.8423	70835520	C	G	0.10	1.44E-04	1.97E-02	5.74E-02
rs20002895	Brain_Substantia_nigra	4	*RUFY3*	71621775	rs28711127	0.8423	70836136	T	G	0.10	5.41E-04	1.97E-02	6.30E-02
rs20002895	Brain_Substantia_nigra	4	*RUFY3*	71621775	rs28508161	0.8423	70836565	C	T	0.10	5.41E-04	4.16E-02	8.92E-02
rs20002895	Brain_Substantia_nigra	4	*RUFY3*	71621775	rs28798425	0.8423	70838306	G	A	0.10	3.25E-04	2.02E-02	6.18E-02
rs20002895	Brain_Substantia_nigra	4	*RUFY3*	71621775	rs4694261	0.8423	70839766	T	C	0.10	3.27E-04	2.14E-02	6.34E-02
rs20002895	Brain_Substantia_nigra	4	*RUFY3*	71621775	rs4694262	0.8423	70839850	A	G	0.10	3.27E-04	1.97E-02	6.12E-02
rs20002895	Brain_Substantia_nigra	4	*RUFY3*	71621775	rs7356202	0.8423	70840436	T	C	0.10	3.27E-04	1.93E-02	6.06E-02
rs20002895	Brain_Substantia_nigra	4	*RUFY3*	71621775	rs77141872	0.8423	70841126	T	C	0.10	3.27E-04	1.97E-02	6.12E-02
rs20002895	Brain_Substantia_nigra	4	*RUFY3*	71621775	rs12648752	0.8423	70841254	C	T	0.10	3.27E-04	1.97E-02	6.12E-02
rs20002895	Brain_Substantia_nigra	4	*RUFY3*	71621775	rs28786470	0.8423	70842730	T	C	0.10	3.25E-04	1.97E-02	6.11E-02
rs20002895	Brain_Substantia_nigra	4	*RUFY3*	71621775	rs17147891	0.8423	70849978	T	A	0.10	3.72E-04	1.97E-02	6.17E-02
rs20002895	Brain_Substantia_nigra	4	*RUFY3*	71621775	rs1231535	0.8743	70875649	T	C	0.13	8.58E-05	3.71E-02	7.39E-02
rs20002895	Brain_Substantia_nigra	4	*UTP3*	71555231	rs776837	0.8743	70876153	C	T	0.13	6.46E-05	4.76E-02	8.35E-02
rs20002895	Brain_Substantia_nigra	4	*RUFY3*	71621775	rs776837	0.8743	70876153	C	T	0.13	6.46E-05	4.51E-02	8.08E-02
rs20002895	Brain_Substantia_nigra	4	*RUFY3*	71621775	rs1083135	0.8843	70881127	C	T	0.11	8.15E-05	2.74E-02	6.10E-02
rs20002895	Brain_Substantia_nigra	4	*RUFY3*	71621775	rs79704056	0.8843	70885815	G	A	0.10	2.93E-04	4.58E-02	9.16E-02
rs20002895	Brain_Substantia_nigra	4	*RUFY3*	71621775	rs776841	0.8843	70889307	C	G	0.11	8.15E-05	2.74E-02	6.10E-02
rs20002895	Brain_Substantia_nigra	4	*RUFY3*	71621775	rs10017681	0.8843	70889847	G	A	0.10	2.67E-04	3.54E-02	7.92E-02
rs20002895	Brain_Substantia_nigra	4	*RUFY3*	71621775	rs800756	0.8843	70897507	C	T	0.11	8.15E-05	2.74E-02	6.10E-02
rs20002895	Brain_Substantia_nigra	4	*RUFY3*	71621775	rs1849937	0.8843	70898907	T	C	0.10	2.93E-04	4.58E-02	9.16E-02
rs20002895	Brain_Substantia_nigra	4	*RUFY3*	71621775	rs148867082	0.9206	70903438	T	C	0.11	3.76E-05	3.55E-02	6.72E-02
rs20002895	Brain_Substantia_nigra	4	*RUFY3*	71621775	rs146349142	0.9206	70903526	G	A	0.11	3.37E-05	3.42E-02	6.52E-02
rs20002895	Brain_Substantia_nigra	4	*RUFY3*	71621775	rs6855909	0.9206	70904992	C	A	0.10	5.05E-05	2.74E-02	5.89E-02
rs20002895	Brain_Substantia_nigra	4	*RUFY3*	71621775	rs13434441	0.9206	70905397	C	T	0.10	1.93E-04	4.58E-02	8.86E-02
rs20002895	Brain_Substantia_nigra	4	*RUFY3*	71621775	rs6843256	0.9113	70906988	A	G	0.11	4.05E-05	3.55E-02	6.74E-02
rs20002895	Brain_Substantia_nigra	4	*RUFY3*	71621775	rs6845010	0.9206	70907119	A	C	0.10	3.01E-05	2.74E-02	5.70E-02
rs20002895	Brain_Substantia_nigra	4	*RUFY3*	71621775	rs28643541	0.9407	70912133	A	G	0.11	7.92E-05	3.69E-02	7.21E-02
rs20002895	Brain_Substantia_nigra	4	*RUFY3*	71621775	rs6857697	0.9407	70914609	A	C	0.11	6.25E-05	3.52E-02	6.87E-02
rs20002895	Brain_Substantia_nigra	4	*RUFY3*	71621775	rs6818370	0.9307	70921069	T	G	0.10	2.23E-05	3.52E-02	6.59E-02
rs20002895	Brain_Substantia_nigra	4	*RUFY3*	71621775	rs10019826	0.9407	70922064	A	G	0.11	4.30E-05	3.69E-02	7.07E-02
rs20002895	Brain_Substantia_nigra	4	*RUFY3*	71621775	rs7660022	0.9314	70943619	A	C	0.10	2.25E-04	4.88E-02	9.25E-02
rs20002895	Brain_Substantia_nigra	4	*RUFY3*	71621775	rs28489946	0.9314	70952099	C	T	0.10	2.25E-04	4.88E-02	9.25E-02
rs20002895	Brain_Substantia_nigra	4	*RUFY3*	71621775	rs7666225	0.9314	70959484	C	T	0.10	2.24E-04	4.86E-02	9.23E-02
rs20002895	Brain_Substantia_nigra	4	*RUFY3*	71621775	rs4694041	0.9314	70961196	G	C	0.10	2.24E-04	4.88E-02	9.25E-02
rs20002895	Brain_Substantia_nigra	4	*RUFY3*	71621775	rs28434698	0.9314	70965054	C	T	0.10	2.10E-04	4.88E-02	9.21E-02
rs20002895	Brain_Substantia_nigra	4	*RUFY3*	71621775	rs17148162	0.9213	70974741	A	C	0.10	4.65E-04	3.21E-02	7.78E-02
rs20002895	Brain_Substantia_nigra	4	*RUFY3*	71621775	rs4694043	0.9213	70975541	C	G	0.10	4.29E-04	3.05E-02	7.56E-02
rs20002895	Brain_Substantia_nigra	4	*RUFY3*	71621775	rs17148181	0.9213	70981462	T	C	0.10	3.67E-04	2.34E-02	6.52E-02
rs20002895	Brain_Substantia_nigra	4	*RUFY3*	71621775	rs55989074	0.9213	70982256	A	T	0.10	2.45E-04	3.05E-02	7.39E-02

rs200028958	Brain_Substantia_nigra	4	*RUFY3*	71621775	rs138807640	0.9213	70984155	A	G	0.10	2.36E-04	3.05E-02	7.36E-02
rs200028958	Brain_Substantia_nigra	4	*RUFY3*	71621775	rs116572259	0.9213	70984463	C	T	0.10	1.99E-04	2.34E-02	6.32E-02
rs200028958	Brain_Substantia_nigra	4	*RUFY3*	71621775	rs10011994	0.9021	70987026	A	T	0.10	1.78E-04	2.34E-02	6.27E-02
rs200028958	Brain_Substantia_nigra	4	*RUFY3*	71621775	rs28866208	0.9021	70987317	G	A	0.10	1.78E-04	2.81E-02	6.86E-02
rs200028958	Brain_Substantia_nigra	4	*RUFY3*	71621775	rs7356162	0.9021	70987982	G	T	0.11	4.61E-04	2.09E-02	6.49E-02
rs200028958	Brain_Substantia_nigra	4	*RUFY3*	71621775	rs28870732	0.9021	70989432	A	C	0.11	4.67E-04	2.08E-02	6.49E-02
rs200028958	Brain_Substantia_nigra	4	*RUFY3*	71621775	rs28793967	0.9021	70989703	T	C	0.11	4.67E-04	2.08E-02	6.49E-02
rs200028958	Brain_Substantia_nigra	4	*RUFY3*	71621775	rs1607989	0.9021	70990224	G	A	0.11	3.41E-04	2.67E-02	7.10E-02
rs200028958	Whole_Blood	4	*GRSF1*	71693580	rs2029572	0.8324	70810063	A	G	0.10	1.75E-04	6.89E-03	3.76E-02
rs200028958	Whole_Blood	4	*GRSF1*	71693580	rs9994880	0.8324	70811070	C	T	0.10	1.95E-04	7.94E-03	3.99E-02
rs200028958	Whole_Blood	4	*GRSF1*	71693580	rs9992853	0.8324	70811521	G	A	0.10	1.95E-04	7.84E-03	3.97E-02
rs200028958	Whole_Blood	4	*GRSF1*	71693580	rs10013848	0.8324	70816927	A	T	0.10	1.50E-04	6.05E-03	3.53E-02
rs200028958	Whole_Blood	4	*GRSF1*	71693580	rs2637816	0.8324	70821025	G	A	0.10	1.77E-04	1.47E-02	5.17E-02
rs200028958	Whole_Blood	4	*GRSF1*	71693580	rs2734570	0.8324	70821713	A	G	0.10	1.77E-04	4.43E-03	3.28E-02
rs200028958	Whole_Blood	4	*GRSF1*	71693580	rs2673720	0.8324	70825933	G	A	0.10	1.68E-04	6.41E-03	3.72E-02
rs200028958	Whole_Blood	4	*GRSF1*	71693580	rs2247569	0.8324	70826437	C	T	0.10	1.68E-04	3.26E-03	2.96E-02
rs200028958	Whole_Blood	4	*GRSF1*	71693580	rs186123891	0.8324	70828593	A	C	0.10	1.88E-04	7.53E-03	3.91E-02
rs200028958	Whole_Blood	4	*GRSF1*	71693580	rs28377851	0.8423	70831724	T	A	0.10	3.63E-04	1.61E-02	6.17E-02
rs200028958	Whole_Blood	4	*GRSF1*	71693580	rs35772950	0.8423	70832941	A	G	0.10	3.63E-04	7.54E-03	4.72E-02
rs200028958	Whole_Blood	4	*GRSF1*	71693580	rs28720737	0.8423	70833223	G	A	0.10	3.63E-04	9.60E-03	5.12E-02
rs200028958	Whole_Blood	4	*GRSF1*	71693580	rs10031238	0.8423	70833357	G	T	0.10	3.63E-04	9.01E-03	5.01E-02
rs200028958	Whole_Blood	4	*GRSF1*	71693580	rs9994584	0.8423	70833723	T	G	0.10	3.63E-04	8.25E-03	4.86E-02
rs200028958	Whole_Blood	4	*GRSF1*	71693580	rs10031845	0.8423	70834238	C	A	0.10	1.44E-04	5.66E-03	3.45E-02
rs200028958	Whole_Blood	4	*GRSF1*	71693580	rs17146478	0.8423	70834683	G	T	0.10	3.72E-04	1.04E-02	5.29E-02
rs200028958	Whole_Blood	4	*GRSF1*	71693580	rs79406241	0.8423	70834693	G	A	0.10	1.44E-04	7.42E-03	3.81E-02
rs200028958	Whole_Blood	4	*GRSF1*	71693580	rs17146480	0.8423	70834740	G	A	0.10	3.60E-04	9.84E-03	5.18E-02
rs200028958	Whole_Blood	4	*GRSF1*	71693580	rs59453376	0.8423	70834877	T	C	0.10	3.72E-04	1.24E-02	5.63E-02
rs200028958	Whole_Blood	4	*GRSF1*	71693580	rs10000684	0.8423	70835403	T	G	0.10	3.72E-04	8.25E-03	4.89E-02
rs200028958	Whole_Blood	4	*GRSF1*	71693580	rs10000776	0.8423	70835520	C	G	0.10	1.44E-04	5.91E-03	3.50E-02
rs200028958	Whole_Blood	4	*GRSF1*	71693580	rs28711127	0.8423	70836136	T	G	0.10	5.41E-04	5.82E-03	3.99E-02
rs200028958	Whole_Blood	4	*GRSF1*	71693580	rs28508161	0.8423	70836565	C	T	0.10	5.41E-04	1.13E-02	5.04E-02
rs200028958	Whole_Blood	4	*GRSF1*	71693580	rs28798425	0.8423	70838306	G	A	0.10	3.25E-04	6.26E-03	3.91E-02
rs200028958	Whole_Blood	4	*GRSF1*	71693580	rs4694261	0.8423	70839766	T	C	0.10	3.27E-04	5.40E-03	3.73E-02
rs200028958	Whole_Blood	4	*GRSF1*	71693580	rs4694262	0.8423	70839850	A	G	0.10	3.27E-04	5.82E-03	3.82E-02
rs200028958	Whole_Blood	4	*GRSF1*	71693580	rs7356202	0.8423	70840436	T	C	0.10	3.27E-04	1.09E-02	4.80E-02
rs200028958	Whole_Blood	4	*GRSF1*	71693580	rs77141872	0.8423	70841126	T	C	0.10	3.27E-04	5.82E-03	3.82E-02
rs200028958	Whole_Blood	4	*GRSF1*	71693580	rs12648752	0.8423	70841254	C	T	0.10	3.27E-04	5.82E-03	3.82E-02
rs200028958	Whole_Blood	4	*GRSF1*	71693580	rs28786470	0.8423	70842730	T	C	0.10	3.25E-04	1.32E-02	5.16E-02
rs200028958	Whole_Blood	4	*GRSF1*	71693580	rs17147891	0.8423	70849978	T	A	0.10	3.72E-04	5.82E-03	3.87E-02
rs200028958	Whole_Blood	4	*GRSF1*	71693580	rs28661681	0.8843	70854978	T	C	0.10	3.80E-04	3.59E-03	3.22E-02

rs200028958	Whole_Blood	4	*GRSF1*	71693580	rs4694263	0.8843	70862927	C	T	0.10	2.79E-04	3.59E-03	3.19E-02
rs200028958	Whole_Blood	4	*GRSF1*	71693580	rs3775764	0.8843	70864065	A	G	0.10	2.79E-04	3.59E-03	3.19E-02
rs200028958	Whole_Blood	4	*GRSF1*	71693580	rs1231535	0.8743	70875649	T	C	0.13	8.58E-05	1.42E-02	4.52E-02
rs200028958	Whole_Blood	4	*GRSF1*	71693580	rs776837	0.8743	70876153	C	T	0.13	6.46E-05	3.14E-02	6.57E-02
rs200028958	Whole_Blood	4	*GRSF1*	71693580	rs1606878	0.8843	70876844	A	T	0.10	2.79E-04	3.87E-03	3.27E-02
rs200028958	Whole_Blood	4	*GRSF1*	71693580	rs28799286	0.8843	70878782	T	C	0.10	2.79E-04	4.62E-03	3.46E-02
rs200028958	Whole_Blood	4	*GRSF1*	71693580	rs1083135	0.8843	70881127	C	T	0.11	8.15E-05	1.64E-02	4.67E-02
rs200028958	Whole_Blood	4	*GRSF1*	71693580	rs79704056	0.8843	70885815	G	A	0.10	2.93E-04	3.40E-03	3.19E-02
rs200028958	Whole_Blood	4	*GRSF1*	71693580	rs776841	0.8843	70889307	C	G	0.11	8.15E-05	7.16E-03	3.20E-02
rs200028958	Whole_Blood	4	*GRSF1*	71693580	rs10017681	0.8843	70889847	G	A	0.10	2.67E-04	4.71E-03	3.45E-02
rs200028958	Whole_Blood	4	*GRSF1*	71693580	rs800756	0.8843	70897507	C	T	0.11	8.15E-05	6.69E-03	3.11E-02
rs200028958	Whole_Blood	4	*GRSF1*	71693580	rs1849937	0.8843	70898907	T	C	0.10	2.93E-04	4.26E-03	3.42E-02
rs200028958	Whole_Blood	4	*GRSF1*	71693580	rs28707754	0.8843	70900864	T	C	0.10	2.67E-04	3.59E-03	3.16E-02
rs200028958	Whole_Blood	4	*GRSF1*	71693580	rs4694040	0.8843	70901653	A	G	0.10	2.67E-04	3.44E-03	3.12E-02
rs200028958	Whole_Blood	4	*GRSF1*	71693580	rs7654757	0.8843	70902412	T	C	0.10	2.67E-04	3.59E-03	3.16E-02
rs200028958	Whole_Blood	4	*GRSF1*	71693580	rs148867082	0.9206	70903438	T	C	0.11	3.76E-05	3.77E-02	6.97E-02
rs200028958	Whole_Blood	4	*GRSF1*	71693580	rs146349142	0.9206	70903526	G	A	0.11	3.37E-05	3.85E-02	7.00E-02
rs200028958	Whole_Blood	4	*GRSF1*	71693580	rs6855909	0.9206	70904992	C	A	0.10	5.05E-05	2.35E-02	5.41E-02
rs200028958	Whole_Blood	4	*MOB1B*	71810964	rs6855909	0.9206	70904992	C	A	0.10	5.05E-05	3.13E-02	6.34E-02
rs200028958	Whole_Blood	4	*GRSF1*	71693580	rs13434441	0.9206	70905397	C	T	0.10	1.93E-04	5.23E-03	3.39E-02
rs200028958	Whole_Blood	4	*MOB1B*	71810964	rs13434441	0.9206	70905397	C	T	0.10	1.93E-04	4.08E-02	8.31E-02
rs200028958	Whole_Blood	4	*GRSF1*	71693580	rs6843256	0.9113	70906988	A	G	0.11	4.05E-05	3.21E-02	6.36E-02
rs200028958	Whole_Blood	4	*GRSF1*	71693580	rs6845010	0.9206	70907119	A	C	0.10	3.01E-05	2.35E-02	5.23E-02
rs200028958	Whole_Blood	4	*MOB1B*	71810964	rs6845010	0.9206	70907119	A	C	0.10	3.01E-05	3.13E-02	6.15E-02
rs200028958	Whole_Blood	4	*GRSF1*	71693580	rs28643541	0.9407	70912133	A	G	0.11	7.92E-05	4.80E-02	8.42E-02
rs200028958	Whole_Blood	4	*MOB1B*	71810964	rs28643541	0.9407	70912133	A	G	0.11	7.92E-05	4.59E-02	8.20E-02
rs200028958	Whole_Blood	4	*GRSF1*	71693580	rs6857697	0.9407	70914609	A	C	0.11	6.25E-05	2.55E-02	5.73E-02
rs200028958	Whole_Blood	4	*MOB1B*	71810964	rs6857697	0.9407	70914609	A	C	0.11	6.25E-05	3.14E-02	6.44E-02
rs200028958	Whole_Blood	4	*GRSF1*	71693580	rs3862054	0.9407	70916273	T	C	0.10	1.52E-04	4.05E-03	3.01E-02
rs200028958	Whole_Blood	4	*GRSF1*	71693580	rs6818370	0.9307	70921069	T	G	0.10	2.23E-05	1.37E-02	3.89E-02
rs200028958	Whole_Blood	4	*MOB1B*	71810964	rs6818370	0.9307	70921069	T	G	0.10	2.23E-05	4.17E-02	7.30E-02
rs200028958	Whole_Blood	4	*GRSF1*	71693580	rs10019826	0.9407	70922064	A	G	0.11	4.30E-05	4.39E-02	7.83E-02
rs200028958	Whole_Blood	4	*MOB1B*	71810964	rs10019826	0.9407	70922064	A	G	0.11	4.30E-05	3.21E-02	6.52E-02
rs200028958	Whole_Blood	4	*GRSF1*	71693580	rs28869909	0.9407	70922226	C	A	0.10	1.09E-04	1.01E-02	4.12E-02
rs200028958	Whole_Blood	4	*GRSF1*	71693580	rs115749891	1.0000	70923658	A	G	0.10	8.26E-05	6.40E-03	3.64E-02
rs200028958	Whole_Blood	4	*GRSF1*	71693580	rs200028958	1.0000	70923661	A	G	0.10	6.25E-06	6.40E-03	2.29E-02
rs200028958	Whole_Blood	4	*GRSF1*	71693580	rs201171800	1.0000	70923663	A	G	0.10	6.25E-06	6.40E-03	2.29E-02
rs200028958	Whole_Blood	4	*GRSF1*	71693580	rs202245754	1.0000	70923665	C	A	0.10	6.25E-06	6.40E-03	2.29E-02
rs200028958	Whole_Blood	4	*GRSF1*	71693580	rs200349247	1.0000	70923666	A	T	0.10	6.25E-06	6.40E-03	2.29E-02
rs200028958	Whole_Blood	4	*GRSF1*	71693580	rs28790602	0.9407	70928040	A	G	0.10	1.10E-04	6.40E-03	3.44E-02

rs200028958	Whole_Blood	4	*GRSF1*	71693580	rs4694265	0.9407	70928571	A	G	0.10	1.10E-04	6.40E-03	3.44E-02
rs200028958	Whole_Blood	4	*GRSF1*	71693580	rs28635879	0.9407	70929763	T	C	0.10	1.22E-04	6.02E-03	3.44E-02
rs200028958	Whole_Blood	4	*MOB1B*	71810964	rs28635879	0.9407	70929763	T	C	0.10	1.22E-04	4.12E-02	8.21E-02
rs200028958	Whole_Blood	4	*GRSF1*	71693580	rs28622022	0.9407	70929889	T	A	0.10	1.12E-04	6.05E-03	3.38E-02
rs200028958	Whole_Blood	4	*MOB1B*	71810964	rs28622022	0.9407	70929889	T	A	0.10	1.12E-04	4.01E-02	8.02E-02
rs200028958	Whole_Blood	4	*GRSF1*	71693580	rs6832546	0.9407	70930584	T	G	0.10	1.22E-04	6.02E-03	3.44E-02
rs200028958	Whole_Blood	4	*MOB1B*	71810964	rs6832546	0.9407	70930584	T	G	0.10	1.22E-04	4.12E-02	8.21E-02
rs200028958	Whole_Blood	4	*GRSF1*	71693580	rs17148034	0.9122	70937524	G	C	0.10	2.93E-04	1.42E-02	5.09E-02
rs200028958	Whole_Blood	4	*GRSF1*	71693580	rs7660022	0.9314	70943619	A	C	0.10	2.25E-04	4.09E-03	3.19E-02
rs200028958	Whole_Blood	4	*GRSF1*	71693580	rs10004471	0.9314	70950312	C	A	0.10	2.25E-04	3.32E-03	2.98E-02
rs200028958	Whole_Blood	4	*GRSF1*	71693580	rs28489946	0.9314	70952099	C	T	0.10	2.25E-04	4.09E-03	3.19E-02
rs200028958	Whole_Blood	4	*GRSF1*	71693580	rs7666225	0.9314	70959484	C	T	0.10	2.24E-04	3.12E-03	2.92E-02
rs200028958	Whole_Blood	4	*MOB1B*	71810964	rs7666225	0.9314	70959484	C	T	0.10	2.24E-04	4.74E-02	9.10E-02
rs200028958	Whole_Blood	4	*GRSF1*	71693580	rs4694041	0.9314	70961196	G	C	0.10	2.24E-04	1.07E-02	4.51E-02
rs200028958	Whole_Blood	4	*GRSF1*	71693580	rs28434698	0.9314	70965054	C	T	0.10	2.10E-04	4.49E-03	3.25E-02
rs200028958	Whole_Blood	4	*GRSF1*	71693580	rs12331580	0.9314	70973499	A	C	0.10	2.87E-04	1.51E-02	5.29E-02
rs200028958	Whole_Blood	4	*GRSF1*	71693580	rs17148162	0.9213	70974741	A	C	0.10	4.65E-04	1.07E-02	4.86E-02
rs200028958	Whole_Blood	4	*GRSF1*	71693580	rs4694043	0.9213	70975541	C	G	0.10	4.29E-04	2.53E-02	6.92E-02
rs200028958	Whole_Blood	4	*GRSF1*	71693580	rs28645990	0.9213	70977161	G	T	0.10	4.29E-04	4.10E-03	3.48E-02
rs200028958	Whole_Blood	4	*GRSF1*	71693580	rs17148181	0.9213	70981462	T	C	0.10	3.67E-04	7.79E-03	4.16E-02
rs200028958	Whole_Blood	4	*GRSF1*	71693580	rs17148184	0.9213	70981602	T	A	0.10	4.29E-04	5.22E-03	3.75E-02
rs200028958	Whole_Blood	4	*GRSF1*	71693580	rs55989074	0.9213	70982256	A	T	0.10	2.45E-04	5.49E-03	3.67E-02
rs200028958	Whole_Blood	4	*GRSF1*	71693580	rs138807640	0.9213	70984155	A	G	0.10	2.36E-04	6.45E-03	3.85E-02
rs200028958	Whole_Blood	4	*GRSF1*	71693580	rs116572259	0.9213	70984463	C	T	0.10	1.99E-04	3.01E-03	2.87E-02
rs200028958	Whole_Blood	4	*GRSF1*	71693580	rs10011994	0.9021	70987026	A	T	0.10	1.78E-04	3.01E-03	2.82E-02
rs200028958	Whole_Blood	4	*GRSF1*	71693580	rs28866208	0.9021	70987317	G	A	0.10	1.78E-04	6.87E-03	3.74E-02
rs200028958	Whole_Blood	4	*GRSF1*	71693580	rs7356162	0.9021	70987982	G	T	0.11	4.61E-04	1.97E-03	2.93E-02
rs200028958	Whole_Blood	4	*GRSF1*	71693580	rs28870732	0.9021	70989432	A	C	0.11	4.67E-04	5.94E-03	4.06E-02
rs200028958	Whole_Blood	4	*GRSF1*	71693580	rs28793967	0.9021	70989703	T	C	0.11	4.67E-04	2.28E-03	3.05E-02
rs200028958	Whole_Blood	4	*GRSF1*	71693580	rs1607989	0.9021	70990224	G	A	0.11	3.41E-04	8.24E-03	4.39E-02
rs200028958	Whole_Blood	4	*GRSF1*	71693580	rs10007698	0.9021	70990879	C	T	0.10	1.26E-04	4.85E-02	9.01E-02
rs4526799	Brain_Amygdala	12	*RPS26*	56436876	rs9919772	0.8450	57260027	T	C	0.33	1.03E-02	5.52E-03	1.89E-01
rs4526799	Brain_Amygdala	12	*RP11-603J24.5*	56518610	rs9919772	0.8450	57260027	T	C	0.33	1.03E-02	3.85E-02	2.27E-01
rs4526799	Brain_Amygdala	12	*ATP5B*	57035878	rs9919772	0.8450	57260027	T	C	0.33	1.03E-02	7.55E-03	1.93E-01
rs4526799	Brain_Amygdala	12	*PRIM1*	57135785	rs9919772	0.8450	57260027	T	C	0.33	1.03E-02	1.68E-03	1.78E-01
rs4526799	Brain_Amygdala	12	*TMEM194A*	57465636	rs9919772	0.8450	57260027	T	C	0.33	1.03E-02	1.11E-02	1.99E-01
rs4526799	Brain_Amygdala	12	*AC025165.8*	58008874	rs9919772	0.8450	57260027	T	C	0.33	1.03E-02	3.20E-02	2.21E-01
rs4526799	Brain_Amygdala	12	*RP11-571M6.8*	58118261	rs9919772	0.8450	57260027	T	C	0.33	1.03E-02	3.58E-02	2.24E-01
rs4526799	Brain_Amygdala	12	*AVIL*	58201929	rs9919772	0.8450	57260027	T	C	0.33	1.03E-02	7.42E-03	1.93E-01
rs4526799	Brain_Amygdala	12	*RPS26*	56436876	rs4495925	0.8650	57268116	C	G	0.33	1.04E-02	3.19E-03	1.80E-01

rs4526799	Brain_Amygdala	12	*RP11-603J24.5*	56518610	rs4495925	0.8650	57268116	C	G	0.33	1.04E-02	4.37E-02	2.28E-01
rs4526799	Brain_Amygdala	12	*ATP5B*	57035878	rs4495925	0.8650	57268116	C	G	0.33	1.04E-02	1.12E-02	1.96E-01
rs4526799	Brain_Amygdala	12	*PRIM1*	57135785	rs4495925	0.8650	57268116	C	G	0.33	1.04E-02	1.39E-03	1.74E-01
rs4526799	Brain_Amygdala	12	*TMEM194A*	57465636	rs4495925	0.8650	57268116	C	G	0.33	1.04E-02	7.65E-03	1.90E-01
rs4526799	Brain_Amygdala	12	*R3HDM2*	57736167	rs4495925	0.8650	57268116	C	G	0.33	1.04E-02	2.75E-02	2.14E-01
rs4526799	Brain_Amygdala	12	*AC025165.8*	58008874	rs4495925	0.8650	57268116	C	G	0.33	1.04E-02	4.49E-02	2.29E-01
rs4526799	Brain_Amygdala	12	*AVIL*	58201929	rs4495925	0.8650	57268116	C	G	0.33	1.04E-02	9.66E-03	1.94E-01
rs4526799	Brain_Amygdala	12	*RPS26*	56436876	rs4471472	0.8650	57268985	A	G	0.32	9.99E-03	3.70E-03	1.78E-01
rs4526799	Brain_Amygdala	12	*RP11-603J24.5*	56518610	rs4471472	0.8650	57268985	A	G	0.32	9.99E-03	3.30E-02	2.15E-01
rs4526799	Brain_Amygdala	12	*ATP5B*	57035878	rs4471472	0.8650	57268985	A	G	0.32	9.99E-03	8.81E-03	1.88E-01
rs4526799	Brain_Amygdala	12	*PRIM1*	57135785	rs4471472	0.8650	57268985	A	G	0.32	9.99E-03	1.40E-03	1.69E-01
rs4526799	Brain_Amygdala	12	*TMEM194A*	57465636	rs4471472	0.8650	57268985	A	G	0.32	9.99E-03	7.69E-03	1.86E-01
rs4526799	Brain_Amygdala	12	*R3HDM2*	57736167	rs4471472	0.8650	57268985	A	G	0.32	9.99E-03	3.47E-02	2.17E-01
rs4526799	Brain_Amygdala	12	*AC025165.8*	58008874	rs4471472	0.8650	57268985	A	G	0.32	9.99E-03	3.21E-02	2.15E-01
rs4526799	Brain_Amygdala	12	*AVIL*	58201929	rs4471472	0.8650	57268985	A	G	0.32	9.99E-03	7.32E-03	1.86E-01
rs4526799	Brain_Amygdala	12	*RPS26*	56436876	rs4633499	0.8610	57269264	A	T	0.32	1.43E-02	1.64E-03	2.10E-01
rs4526799	Brain_Amygdala	12	*RP11-603J24.5*	56518610	rs4633499	0.8610	57269264	A	T	0.32	1.43E-02	2.87E-02	2.46E-01
rs4526799	Brain_Amygdala	12	*ATP5B*	57035878	rs4633499	0.8610	57269264	A	T	0.32	1.43E-02	9.93E-03	2.27E-01
rs4526799	Brain_Amygdala	12	*PRIM1*	57135785	rs4633499	0.8610	57269264	A	T	0.32	1.43E-02	2.64E-03	2.13E-01
rs4526799	Brain_Amygdala	12	*TMEM194A*	57465636	rs4633499	0.8610	57269264	A	T	0.32	1.43E-02	4.00E-03	2.17E-01
rs4526799	Brain_Amygdala	12	*AC025165.8*	58008874	rs4633499	0.8610	57269264	A	T	0.32	1.43E-02	3.15E-02	2.48E-01
rs4526799	Brain_Amygdala	12	*AVIL*	58201929	rs4633499	0.8610	57269264	A	T	0.32	1.43E-02	7.73E-03	2.24E-01
rs4526799	Brain_Amygdala	12	*ERBB3*	56485465	rs12300079	0.9956	57273194	T	C	0.36	3.73E-03	3.43E-02	1.69E-01
rs4526799	Brain_Amygdala	12	*ATP5B*	57035878	rs12300079	0.9956	57273194	T	C	0.36	3.73E-03	1.95E-02	1.53E-01
rs4526799	Brain_Amygdala	12	*PRIM1*	57135785	rs12300079	0.9956	57273194	T	C	0.36	3.73E-03	4.71E-03	1.27E-01
rs4526799	Brain_Amygdala	12	*AC025165.8*	58008874	rs12300079	0.9956	57273194	T	C	0.36	3.73E-03	2.87E-02	1.63E-01
rs4526799	Brain_Amygdala	12	*TSPAN31*	58138756	rs12300079	0.9956	57273194	T	C	0.36	3.73E-03	4.57E-02	1.80E-01
rs4526799	Brain_Amygdala	12	*AVIL*	58201929	rs12300079	0.9956	57273194	T	C	0.36	3.73E-03	2.48E-03	1.20E-01
rs4526799	Brain_Amygdala	12	*ERBB3*	56485465	rs12300191	0.9956	57273289	A	G	0.36	3.59E-03	3.43E-02	1.67E-01
rs4526799	Brain_Amygdala	12	*ATP5B*	57035878	rs12300191	0.9956	57273289	A	G	0.36	3.59E-03	1.95E-02	1.50E-01
rs4526799	Brain_Amygdala	12	*PRIM1*	57135785	rs12300191	0.9956	57273289	A	G	0.36	3.59E-03	4.71E-03	1.25E-01
rs4526799	Brain_Amygdala	12	*AC025165.8*	58008874	rs12300191	0.9956	57273289	A	G	0.36	3.59E-03	2.87E-02	1.61E-01
rs4526799	Brain_Amygdala	12	*TSPAN31*	58138756	rs12300191	0.9956	57273289	A	G	0.36	3.59E-03	4.57E-02	1.78E-01
rs4526799	Brain_Amygdala	12	*AVIL*	58201929	rs12300191	0.9956	57273289	A	G	0.36	3.59E-03	2.48E-03	1.18E-01
rs4526799	Brain_Amygdala	12	*ERBB3*	56485465	rs4514464	0.9956	57276375	C	T	0.36	2.92E-03	3.43E-02	1.64E-01
rs4526799	Brain_Amygdala	12	*ATP5B*	57035878	rs4514464	0.9956	57276375	C	T	0.36	2.92E-03	1.95E-02	1.47E-01
rs4526799	Brain_Amygdala	12	*PRIM1*	57135785	rs4514464	0.9956	57276375	C	T	0.36	2.92E-03	4.71E-03	1.22E-01
rs4526799	Brain_Amygdala	12	*AC025165.8*	58008874	rs4514464	0.9956	57276375	C	T	0.36	2.92E-03	2.87E-02	1.58E-01
rs4526799	Brain_Amygdala	12	*TSPAN31*	58138756	rs4514464	0.9956	57276375	C	T	0.36	2.92E-03	4.57E-02	1.75E-01
rs4526799	Brain_Amygdala	12	*AVIL*	58201929	rs4514464	0.9956	57276375	C	T	0.36	2.92E-03	2.48E-03	1.14E-01

rs4526799	Brain_Amygdala	12	*ERBB3*	56485465	rs4417325	0.9956	57277302	G	A	0.36	3.59E-03	3.43E-02	1.67E-01
rs4526799	Brain_Amygdala	12	*ATP5B*	57035878	rs4417325	0.9956	57277302	G	A	0.36	3.59E-03	1.95E-02	1.50E-01
rs4526799	Brain_Amygdala	12	*PRIM1*	57135785	rs4417325	0.9956	57277302	G	A	0.36	3.59E-03	4.71E-03	1.25E-01
rs4526799	Brain_Amygdala	12	*AC025165.8*	58008874	rs4417325	0.9956	57277302	G	A	0.36	3.59E-03	2.87E-02	1.61E-01
rs4526799	Brain_Amygdala	12	*TSPAN31*	58138756	rs4417325	0.9956	57277302	G	A	0.36	3.59E-03	4.57E-02	1.78E-01
rs4526799	Brain_Amygdala	12	*AVIL*	58201929	rs4417325	0.9956	57277302	G	A	0.36	3.59E-03	2.48E-03	1.18E-01
rs4526799	Brain_Amygdala	12	*ERBB3*	56485465	rs11172030	0.9956	57278076	A	C	0.36	3.63E-03	3.43E-02	1.67E-01
rs4526799	Brain_Amygdala	12	*ATP5B*	57035878	rs11172030	0.9956	57278076	A	C	0.36	3.63E-03	1.95E-02	1.51E-01
rs4526799	Brain_Amygdala	12	*PRIM1*	57135785	rs11172030	0.9956	57278076	A	C	0.36	3.63E-03	4.71E-03	1.26E-01
rs4526799	Brain_Amygdala	12	*AC025165.8*	58008874	rs11172030	0.9956	57278076	A	C	0.36	3.63E-03	2.87E-02	1.61E-01
rs4526799	Brain_Amygdala	12	*TSPAN31*	58138756	rs11172030	0.9956	57278076	A	C	0.36	3.63E-03	4.57E-02	1.78E-01
rs4526799	Brain_Amygdala	12	*AVIL*	58201929	rs11172030	0.9956	57278076	A	C	0.36	3.63E-03	2.48E-03	1.18E-01
rs4526799	Brain_Amygdala	12	*ERBB3*	56485465	rs10876944	0.9956	57279372	T	A	0.36	3.63E-03	3.43E-02	1.67E-01
rs4526799	Brain_Amygdala	12	*ATP5B*	57035878	rs10876944	0.9956	57279372	T	A	0.36	3.63E-03	1.95E-02	1.51E-01
rs4526799	Brain_Amygdala	12	*PRIM1*	57135785	rs10876944	0.9956	57279372	T	A	0.36	3.63E-03	4.71E-03	1.26E-01
rs4526799	Brain_Amygdala	12	*AC025165.8*	58008874	rs10876944	0.9956	57279372	T	A	0.36	3.63E-03	2.87E-02	1.61E-01
rs4526799	Brain_Amygdala	12	*TSPAN31*	58138756	rs10876944	0.9956	57279372	T	A	0.36	3.63E-03	4.57E-02	1.78E-01
rs4526799	Brain_Amygdala	12	*AVIL*	58201929	rs10876944	0.9956	57279372	T	A	0.36	3.63E-03	2.48E-03	1.18E-01
rs4526799	Brain_Amygdala	12	*ERBB3*	56485465	rs4326839	0.9956	57280374	G	C	0.36	3.58E-03	3.43E-02	1.67E-01
rs4526799	Brain_Amygdala	12	*ATP5B*	57035878	rs4326839	0.9956	57280374	G	C	0.36	3.58E-03	1.95E-02	1.50E-01
rs4526799	Brain_Amygdala	12	*PRIM1*	57135785	rs4326839	0.9956	57280374	G	C	0.36	3.58E-03	4.71E-03	1.25E-01
rs4526799	Brain_Amygdala	12	*AC025165.8*	58008874	rs4326839	0.9956	57280374	G	C	0.36	3.58E-03	2.87E-02	1.61E-01
rs4526799	Brain_Amygdala	12	*TSPAN31*	58138756	rs4326839	0.9956	57280374	G	C	0.36	3.58E-03	4.57E-02	1.77E-01
rs4526799	Brain_Amygdala	12	*AVIL*	58201929	rs4326839	0.9956	57280374	G	C	0.36	3.58E-03	2.48E-03	1.18E-01
rs4526799	Brain_Amygdala	12	*ERBB3*	56485465	rs4526799	1.0000	57280586	T	C	0.34	7.26E-06	3.43E-02	6.91E-02
rs4526799	Brain_Amygdala	12	*ATP5B*	57035878	rs4526799	1.0000	57280586	T	C	0.34	7.26E-06	1.95E-02	5.10E-02
rs4526799	Brain_Amygdala	12	*PRIM1*	57135785	rs4526799	1.0000	57280586	T	C	0.34	7.26E-06	4.71E-03	2.70E-02
rs4526799	Brain_Amygdala	12	*AC025165.8*	58008874	rs4526799	1.0000	57280586	T	C	0.34	7.26E-06	2.87E-02	6.25E-02
rs4526799	Brain_Amygdala	12	*TSPAN31*	58138756	rs4526799	1.0000	57280586	T	C	0.34	7.26E-06	4.57E-02	8.16E-02
rs4526799	Brain_Amygdala	12	*AVIL*	58201929	rs4526799	1.0000	57280586	T	C	0.34	7.26E-06	2.48E-03	2.13E-02
rs4526799	Brain_Amygdala	12	*ERBB3*	56485465	rs28876529	0.9956	57285301	T	A	0.36	2.25E-03	3.43E-02	1.52E-01
rs4526799	Brain_Amygdala	12	*ATP5B*	57035878	rs28876529	0.9956	57285301	T	A	0.36	2.25E-03	1.95E-02	1.34E-01
rs4526799	Brain_Amygdala	12	*PRIM1*	57135785	rs28876529	0.9956	57285301	T	A	0.36	2.25E-03	4.71E-03	1.08E-01
rs4526799	Brain_Amygdala	12	*AC025165.8*	58008874	rs28876529	0.9956	57285301	T	A	0.36	2.25E-03	2.87E-02	1.45E-01
rs4526799	Brain_Amygdala	12	*TSPAN31*	58138756	rs28876529	0.9956	57285301	T	A	0.36	2.25E-03	4.57E-02	1.63E-01
rs4526799	Brain_Amygdala	12	*AVIL*	58201929	rs28876529	0.9956	57285301	T	A	0.36	2.25E-03	2.48E-03	1.01E-01
rs4526799	Brain_Amygdala	12	*ERBB3*	56485465	rs11172037	0.9956	57285427	T	A	0.36	2.25E-03	3.43E-02	1.52E-01
rs4526799	Brain_Amygdala	12	*ATP5B*	57035878	rs11172037	0.9956	57285427	T	A	0.36	2.25E-03	1.95E-02	1.34E-01
rs4526799	Brain_Amygdala	12	*PRIM1*	57135785	rs11172037	0.9956	57285427	T	A	0.36	2.25E-03	4.71E-03	1.08E-01
rs4526799	Brain_Amygdala	12	*AC025165.8*	58008874	rs11172037	0.9956	57285427	T	A	0.36	2.25E-03	2.87E-02	1.45E-01

rs4526799	Brain_Amygdala	12	*TSPAN31*	58138756	rs11172037	0.9956	57285427	T	A	0.36	2.25E-03	4.57E-02	1.63E-01
rs4526799	Brain_Amygdala	12	*AVIL*	58201929	rs11172037	0.9956	57285427	T	A	0.36	2.25E-03	2.48E-03	1.01E-01
rs4526799	Brain_Amygdala	12	*ERBB3*	56485465	rs12321987	0.9956	57288449	G	A	0.36	4.47E-03	3.43E-02	1.88E-01
rs4526799	Brain_Amygdala	12	*ATP5B*	57035878	rs12321987	0.9956	57288449	G	A	0.36	4.47E-03	1.95E-02	1.72E-01
rs4526799	Brain_Amygdala	12	*PRIM1*	57135785	rs12321987	0.9956	57288449	G	A	0.36	4.47E-03	4.71E-03	1.48E-01
rs4526799	Brain_Amygdala	12	*AC025165.8*	58008874	rs12321987	0.9956	57288449	G	A	0.36	4.47E-03	2.87E-02	1.82E-01
rs4526799	Brain_Amygdala	12	*TSPAN31*	58138756	rs12321987	0.9956	57288449	G	A	0.36	4.47E-03	4.57E-02	1.98E-01
rs4526799	Brain_Amygdala	12	*AVIL*	58201929	rs12321987	0.9956	57288449	G	A	0.36	4.47E-03	2.48E-03	1.41E-01
rs4526799	Brain_Amygdala	12	*ATP5B*	57035878	rs11172043	0.9869	57293182	G	A	0.35	4.58E-03	3.62E-02	1.88E-01
rs4526799	Brain_Amygdala	12	*PRIM1*	57135785	rs11172043	0.9869	57293182	G	A	0.35	4.58E-03	1.53E-02	1.65E-01
rs4526799	Brain_Amygdala	12	*AC025165.8*	58008874	rs11172043	0.9869	57293182	G	A	0.35	4.58E-03	2.59E-02	1.78E-01
rs4526799	Brain_Amygdala	12	*AVIL*	58201929	rs11172043	0.9869	57293182	G	A	0.35	4.58E-03	4.93E-03	1.47E-01
rs4526799	Brain_Amygdala	12	*ATP5B*	57035878	rs12426816	0.9869	57294074	A	C	0.35	4.50E-03	3.62E-02	1.88E-01
rs4526799	Brain_Amygdala	12	*PRIM1*	57135785	rs12426816	0.9869	57294074	A	C	0.35	4.50E-03	1.53E-02	1.65E-01
rs4526799	Brain_Amygdala	12	*AC025165.8*	58008874	rs12426816	0.9869	57294074	A	C	0.35	4.50E-03	2.59E-02	1.77E-01
rs4526799	Brain_Amygdala	12	*AVIL*	58201929	rs12426816	0.9869	57294074	A	C	0.35	4.50E-03	4.93E-03	1.47E-01
rs4526799	Brain_Amygdala	12	*ATP5B*	57035878	rs11172047	0.9869	57298080	T	C	0.35	6.51E-03	3.62E-02	2.01E-01
rs4526799	Brain_Amygdala	12	*PRIM1*	57135785	rs11172047	0.9869	57298080	T	C	0.35	6.51E-03	1.53E-02	1.80E-01
rs4526799	Brain_Amygdala	12	*AC025165.8*	58008874	rs11172047	0.9869	57298080	T	C	0.35	6.51E-03	2.59E-02	1.92E-01
rs4526799	Brain_Amygdala	12	*AVIL*	58201929	rs11172047	0.9869	57298080	T	C	0.35	6.51E-03	4.93E-03	1.62E-01
rs4526799	Brain_Amygdala	12	*ATP5B*	57035878	rs2371631	0.9869	57298614	T	A	0.35	6.32E-03	3.62E-02	1.98E-01
rs4526799	Brain_Amygdala	12	*PRIM1*	57135785	rs2371631	0.9869	57298614	T	A	0.35	6.32E-03	1.53E-02	1.76E-01
rs4526799	Brain_Amygdala	12	*AC025165.8*	58008874	rs2371631	0.9869	57298614	T	A	0.35	6.32E-03	2.59E-02	1.89E-01
rs4526799	Brain_Amygdala	12	*AVIL*	58201929	rs2371631	0.9869	57298614	T	A	0.35	6.32E-03	4.93E-03	1.59E-01
rs4526799	Brain_Amygdala	12	*ATP5B*	57035878	rs12305763	0.9869	57299263	G	A	0.35	6.82E-03	3.62E-02	2.03E-01
rs4526799	Brain_Amygdala	12	*PRIM1*	57135785	rs12305763	0.9869	57299263	G	A	0.35	6.82E-03	1.53E-02	1.81E-01
rs4526799	Brain_Amygdala	12	*AC025165.8*	58008874	rs12305763	0.9869	57299263	G	A	0.35	6.82E-03	2.59E-02	1.93E-01
rs4526799	Brain_Amygdala	12	*AVIL*	58201929	rs12305763	0.9869	57299263	G	A	0.35	6.82E-03	4.93E-03	1.63E-01
rs4526799	Brain_Amygdala	12	*RP11-603J24.5*	56518610	rs11172049	0.9128	57304203	T	C	0.35	1.15E-02	3.55E-02	2.02E-01
rs4526799	Brain_Amygdala	12	*PRIM1*	57135785	rs11172049	0.9128	57304203	T	C	0.35	1.15E-02	1.10E-02	1.75E-01
rs4526799	Brain_Amygdala	12	*NAB2*	57485933	rs11172049	0.9128	57304203	T	C	0.35	1.15E-02	4.68E-02	2.12E-01
rs4526799	Brain_Amygdala	12	*AC025165.8*	58008874	rs11172049	0.9128	57304203	T	C	0.35	1.15E-02	1.78E-02	1.84E-01
rs4526799	Brain_Amygdala	12	*AVIL*	58201929	rs11172049	0.9128	57304203	T	C	0.35	1.15E-02	1.16E-02	1.76E-01
rs4526799	Brain_Amygdala	12	*RP11-603J24.5*	56518610	rs1874888	0.9085	57305138	A	C	0.35	1.15E-02	3.55E-02	2.02E-01
rs4526799	Brain_Amygdala	12	*PRIM1*	57135785	rs1874888	0.9085	57305138	A	C	0.35	1.15E-02	1.10E-02	1.75E-01
rs4526799	Brain_Amygdala	12	*NAB2*	57485933	rs1874888	0.9085	57305138	A	C	0.35	1.15E-02	4.68E-02	2.12E-01
rs4526799	Brain_Amygdala	12	*AC025165.8*	58008874	rs1874888	0.9085	57305138	A	C	0.35	1.15E-02	1.78E-02	1.84E-01
rs4526799	Brain_Amygdala	12	*AVIL*	58201929	rs1874888	0.9085	57305138	A	C	0.35	1.15E-02	1.16E-02	1.76E-01
rs4526799	Brain_Amygdala	12	*PRIM1*	57135785	rs10506349	0.9128	57306412	T	C	0.37	1.10E-02	3.37E-03	1.49E-01
rs4526799	Brain_Amygdala	12	*NAB2*	57485933	rs10506349	0.9128	57306412	T	C	0.37	1.10E-02	3.14E-02	1.89E-01

rs4526799	Brain_Amygdala	12	*AC025165.8*	58008874	rs10506349	0.9128	57306412	T	C	0.37	1.10E-02	2.92E-03	1.48E-01
rs4526799	Brain_Amygdala	12	*B4GALNT1*	58023536	rs10506349	0.9128	57306412	T	C	0.37	1.10E-02	3.47E-02	1.93E-01
rs4526799	Brain_Amygdala	12	*TSPAN31*	58138756	rs10506349	0.9128	57306412	T	C	0.37	1.10E-02	4.28E-02	2.00E-01
rs4526799	Brain_Amygdala	12	*AVIL*	58201929	rs10506349	0.9128	57306412	T	C	0.37	1.10E-02	1.24E-02	1.67E-01
rs4526799	Brain_Amygdala	12	*RP11-603J24.5*	56518610	rs10876951	0.9085	57306430	T	G	0.35	1.15E-02	3.55E-02	2.02E-01
rs4526799	Brain_Amygdala	12	*PRIM1*	57135785	rs10876951	0.9085	57306430	T	G	0.35	1.15E-02	1.10E-02	1.75E-01
rs4526799	Brain_Amygdala	12	*NAB2*	57485933	rs10876951	0.9085	57306430	T	G	0.35	1.15E-02	4.68E-02	2.12E-01
rs4526799	Brain_Amygdala	12	*AC025165.8*	58008874	rs10876951	0.9085	57306430	T	G	0.35	1.15E-02	1.78E-02	1.84E-01
rs4526799	Brain_Amygdala	12	*AVIL*	58201929	rs10876951	0.9085	57306430	T	G	0.35	1.15E-02	1.16E-02	1.76E-01
rs4526799	Brain_Amygdala	12	*PRIM1*	57135785	rs10747774	0.9128	57307079	T	C	0.36	1.16E-02	3.37E-03	1.55E-01
rs4526799	Brain_Amygdala	12	*NAB2*	57485933	rs10747774	0.9128	57307079	T	C	0.36	1.16E-02	3.14E-02	1.95E-01
rs4526799	Brain_Amygdala	12	*AC025165.8*	58008874	rs10747774	0.9128	57307079	T	C	0.36	1.16E-02	2.92E-03	1.54E-01
rs4526799	Brain_Amygdala	12	*B4GALNT1*	58023536	rs10747774	0.9128	57307079	T	C	0.36	1.16E-02	3.47E-02	1.98E-01
rs4526799	Brain_Amygdala	12	*TSPAN31*	58138756	rs10747774	0.9128	57307079	T	C	0.36	1.16E-02	4.28E-02	2.05E-01
rs4526799	Brain_Amygdala	12	*AVIL*	58201929	rs10747774	0.9128	57307079	T	C	0.36	1.16E-02	1.24E-02	1.73E-01
rs4526799	Brain_Amygdala	12	*RP11-603J24.5*	56518610	rs10783812	0.9085	57308723	C	T	0.35	1.19E-02	3.55E-02	2.05E-01
rs4526799	Brain_Amygdala	12	*PRIM1*	57135785	rs10783812	0.9085	57308723	C	T	0.35	1.19E-02	1.10E-02	1.78E-01
rs4526799	Brain_Amygdala	12	*NAB2*	57485933	rs10783812	0.9085	57308723	C	T	0.35	1.19E-02	4.68E-02	2.15E-01
rs4526799	Brain_Amygdala	12	*AC025165.8*	58008874	rs10783812	0.9085	57308723	C	T	0.35	1.19E-02	1.78E-02	1.87E-01
rs4526799	Brain_Amygdala	12	*AVIL*	58201929	rs10783812	0.9085	57308723	C	T	0.35	1.19E-02	1.16E-02	1.79E-01
rs4526799	Brain_Amygdala	12	*PRIM1*	57135785	rs11172056	0.9089	57308975	C	T	0.37	1.10E-02	3.37E-03	1.50E-01
rs4526799	Brain_Amygdala	12	*NAB2*	57485933	rs11172056	0.9089	57308975	C	T	0.37	1.10E-02	3.14E-02	1.90E-01
rs4526799	Brain_Amygdala	12	*AC025165.8*	58008874	rs11172056	0.9089	57308975	C	T	0.37	1.10E-02	2.92E-03	1.48E-01
rs4526799	Brain_Amygdala	12	*B4GALNT1*	58023536	rs11172056	0.9089	57308975	C	T	0.37	1.10E-02	3.47E-02	1.93E-01
rs4526799	Brain_Amygdala	12	*TSPAN31*	58138756	rs11172056	0.9089	57308975	C	T	0.37	1.10E-02	4.28E-02	2.00E-01
rs4526799	Brain_Amygdala	12	*AVIL*	58201929	rs11172056	0.9089	57308975	C	T	0.37	1.10E-02	1.24E-02	1.68E-01
rs4526799	Brain_Amygdala	12	*PRIM1*	57135785	rs7302420	0.9085	57309884	G	C	0.36	1.54E-02	1.24E-02	1.97E-01
rs4526799	Brain_Amygdala	12	*AC025165.8*	58008874	rs7302420	0.9085	57309884	G	C	0.36	1.54E-02	1.16E-02	1.96E-01
rs4526799	Brain_Amygdala	12	*TSPAN31*	58138756	rs7302420	0.9085	57309884	G	C	0.36	1.54E-02	1.97E-02	2.06E-01
rs4526799	Brain_Amygdala	12	*AVIL*	58201929	rs7302420	0.9085	57309884	G	C	0.36	1.54E-02	6.42E-03	1.87E-01
rs4526799	Brain_Amygdala	12	*RNF41*	56607001	rs12228618	0.9128	57311229	T	C	0.37	1.29E-02	4.86E-02	2.13E-01
rs4526799	Brain_Amygdala	12	*PRIM1*	57135785	rs12228618	0.9128	57311229	T	C	0.37	1.29E-02	9.05E-04	1.48E-01
rs4526799	Brain_Amygdala	12	*NAB2*	57485933	rs12228618	0.9128	57311229	T	C	0.37	1.29E-02	2.51E-02	1.92E-01
rs4526799	Brain_Amygdala	12	*AC025165.8*	58008874	rs12228618	0.9128	57311229	T	C	0.37	1.29E-02	5.09E-03	1.64E-01
rs4526799	Brain_Amygdala	12	*B4GALNT1*	58023536	rs12228618	0.9128	57311229	T	C	0.37	1.29E-02	4.44E-02	2.10E-01
rs4526799	Brain_Amygdala	12	*TSPAN31*	58138756	rs12228618	0.9128	57311229	T	C	0.37	1.29E-02	3.19E-02	1.99E-01
rs4526799	Brain_Amygdala	12	*AVIL*	58201929	rs12228618	0.9128	57311229	T	C	0.37	1.29E-02	2.59E-02	1.93E-01
rs4526799	Brain_Amygdala	12	*PRIM1*	57135785	rs9739473	0.9012	57313335	A	T	0.37	2.65E-02	1.93E-03	2.30E-01
rs4526799	Brain_Amygdala	12	*NAB2*	57485933	rs9739473	0.9012	57313335	A	T	0.37	2.65E-02	2.88E-02	2.63E-01
rs4526799	Brain_Amygdala	12	*AC025165.8*	58008874	rs9739473	0.9012	57313335	A	T	0.37	2.65E-02	4.10E-03	2.35E-01

rs4526799	Brain_Amygdala	12	*TSPAN31*	58138756	rs9739473	0.9012	57313335	A	T	0.37	2.65E-02	3.65E-02	2.69E-01
rs4526799	Brain_Amygdala	12	*AVIL*	58201929	rs9739473	0.9012	57313335	A	T	0.37	2.65E-02	1.35E-02	2.49E-01
rs4526799	Brain_Anterior_cingulate_cortex_BA24	12	*DGKA*	56334604	rs9919772	0.8450	57260027	T	C	0.33	1.03E-02	2.85E-02	2.18E-01
rs4526799	Brain_Anterior_cingulate_cortex_BA24	12	*MYL6*	56554355	rs9919772	0.8450	57260027	T	C	0.33	1.03E-02	3.73E-02	2.26E-01
rs4526799	Brain_Anterior_cingulate_cortex_BA24	12	*RP11-977G19.11*	56701259	rs9919772	0.8450	57260027	T	C	0.33	1.03E-02	5.34E-03	1.89E-01
rs4526799	Brain_Anterior_cingulate_cortex_BA24	12	*CNPY2*	56706816	rs9919772	0.8450	57260027	T	C	0.33	1.03E-02	2.93E-02	2.19E-01
rs4526799	Brain_Anterior_cingulate_cortex_BA24	12	*TIMELESS*	56827045	rs9919772	0.8450	57260027	T	C	0.33	1.03E-02	1.37E-02	2.02E-01
rs4526799	Brain_Anterior_cingulate_cortex_BA24	12	*TMEM194A*	57465636	rs9919772	0.8450	57260027	T	C	0.33	1.03E-02	2.04E-04	1.67E-01
rs4526799	Brain_Anterior_cingulate_cortex_BA24	12	*INHBE*	57849584	rs9919772	0.8450	57260027	T	C	0.33	1.03E-02	5.48E-03	1.89E-01
rs4526799	Brain_Anterior_cingulate_cortex_BA24	12	*RP11-571M6.17*	58197909	rs9919772	0.8450	57260027	T	C	0.33	1.03E-02	4.15E-02	2.29E-01
rs4526799	Brain_Anterior_cingulate_cortex_BA24	12	*MYL6*	56554355	rs4495925	0.8650	57268116	C	G	0.33	1.04E-02	4.69E-02	2.31E-01
rs4526799	Brain_Anterior_cingulate_cortex_BA24	12	*RP11-977G19.11*	56701259	rs4495925	0.8650	57268116	C	G	0.33	1.04E-02	7.67E-03	1.90E-01
rs4526799	Brain_Anterior_cingulate_cortex_BA24	12	*CNPY2*	56706816	rs4495925	0.8650	57268116	C	G	0.33	1.04E-02	3.58E-02	2.22E-01
rs4526799	Brain_Anterior_cingulate_cortex_BA24	12	*TIMELESS*	56827045	rs4495925	0.8650	57268116	C	G	0.33	1.04E-02	2.95E-02	2.16E-01
rs4526799	Brain_Anterior_cingulate_cortex_BA24	12	*TMEM194A*	57465636	rs4495925	0.8650	57268116	C	G	0.33	1.04E-02	1.82E-04	1.63E-01
rs4526799	Brain_Anterior_cingulate_cortex_BA24	12	*INHBE*	57849584	rs4495925	0.8650	57268116	C	G	0.33	1.04E-02	7.64E-04	1.70E-01
rs4526799	Brain_Anterior_cingulate_cortex_BA24	12	*RP11-571M6.17*	58197909	rs4495925	0.8650	57268116	C	G	0.33	1.04E-02	3.90E-02	2.24E-01
rs4526799	Brain_Anterior_cingulate_cortex_BA24	12	*RP11-571M6.18*	58208493	rs4495925	0.8650	57268116	C	G	0.33	1.04E-02	4.19E-02	2.27E-01
rs4526799	Brain_Anterior_cingulate_cortex_BA24	12	*MYL6*	56554355	rs4471472	0.8650	57268985	A	G	0.32	9.99E-03	4.25E-02	2.23E-01
rs4526799	Brain_Anterior_cingulate_cortex_BA24	12	*RP11-977G19.11*	56701259	rs4471472	0.8650	57268985	A	G	0.32	9.99E-03	7.46E-03	1.86E-01
rs4526799	Brain_Anterior_cingulate_cortex_BA24	12	*CNPY2*	56706816	rs4471472	0.8650	57268985	A	G	0.32	9.99E-03	2.71E-02	2.10E-01
rs4526799	Brain_Anterior_cingulate_cortex_BA24	12	*TIMELESS*	56827045	rs4471472	0.8650	57268985	A	G	0.32	9.99E-03	2.96E-02	2.12E-01
rs4526799	Brain_Anterior_cingulate_cortex_BA24	12	*TMEM194A*	57465636	rs4471472	0.8650	57268985	A	G	0.32	9.99E-03	2.10E-04	1.59E-01
rs4526799	Brain_Anterior_cingulate_cortex_BA24	12	*INHBE*	57849584	rs4471472	0.8650	57268985	A	G	0.32	9.99E-03	7.69E-04	1.66E-01
rs4526799	Brain_Anterior_cingulate_cortex_BA24	12	*RP11-571M6.17*	58197909	rs4471472	0.8650	57268985	A	G	0.32	9.99E-03	3.70E-02	2.19E-01
rs4526799	Brain_Anterior_cingulate_cortex_BA24	12	*AVIL*	58201929	rs4471472	0.8650	57268985	A	G	0.32	9.99E-03	4.84E-02	2.28E-01
rs4526799	Brain_Anterior_cingulate_cortex_BA24	12	*RP11-571M6.18*	58208493	rs4471472	0.8650	57268985	A	G	0.32	9.99E-03	4.17E-02	2.23E-01
rs4526799	Brain_Anterior_cingulate_cortex_BA24	12	*MYL6*	56554355	rs4633499	0.8610	57269264	A	T	0.32	1.43E-02	3.97E-02	2.55E-01
rs4526799	Brain_Anterior_cingulate_cortex_BA24	12	*RP11-977G19.11*	56701259	rs4633499	0.8610	57269264	A	T	0.32	1.43E-02	1.45E-02	2.33E-01
rs4526799	Brain_Anterior_cingulate_cortex_BA24	12	*CNPY2*	56706816	rs4633499	0.8610	57269264	A	T	0.32	1.43E-02	1.86E-02	2.37E-01
rs4526799	Brain_Anterior_cingulate_cortex_BA24	12	*TIMELESS*	56827045	rs4633499	0.8610	57269264	A	T	0.32	1.43E-02	3.30E-02	2.50E-01
rs4526799	Brain_Anterior_cingulate_cortex_BA24	12	*TMEM194A*	57465636	rs4633499	0.8610	57269264	A	T	0.32	1.43E-02	1.49E-04	1.98E-01
rs4526799	Brain_Anterior_cingulate_cortex_BA24	12	*INHBE*	57849584	rs4633499	0.8610	57269264	A	T	0.32	1.43E-02	4.42E-04	2.02E-01
rs4526799	Brain_Anterior_cingulate_cortex_BA24	12	*RP11-571M6.17*	58197909	rs4633499	0.8610	57269264	A	T	0.32	1.43E-02	3.65E-02	2.52E-01
rs4526799	Brain_Anterior_cingulate_cortex_BA24	12	*RP11-571M6.18*	58208493	rs4633499	0.8610	57269264	A	T	0.32	1.43E-02	3.11E-02	2.48E-01
rs4526799	Brain_Anterior_cingulate_cortex_BA24	12	*MYL6*	56554355	rs12300079	0.9956	57273194	T	C	0.36	3.73E-03	2.00E-02	1.53E-01
rs4526799	Brain_Anterior_cingulate_cortex_BA24	12	*CNPY2*	56706816	rs12300079	0.9956	57273194	T	C	0.36	3.73E-03	4.26E-02	1.77E-01
rs4526799	Brain_Anterior_cingulate_cortex_BA24	12	*BAZ2A*	57009990	rs12300079	0.9956	57273194	T	C	0.36	3.73E-03	2.49E-02	1.59E-01
rs4526799	Brain_Anterior_cingulate_cortex_BA24	12	*TMEM194A*	57465636	rs12300079	0.9956	57273194	T	C	0.36	3.73E-03	1.24E-03	1.14E-01
rs4526799	Brain_Anterior_cingulate_cortex_BA24	12	*INHBE*	57849584	rs12300079	0.9956	57273194	T	C	0.36	3.73E-03	2.13E-03	1.19E-01

rs4526799	Brain_Anterior_cingulate_cortex_BA24	12	*RP11-571M6.17*	58197909	rs12300079	0.9956	57273194	T	C	0.36	3.73E-03	4.77E-02	1.82E-01
rs4526799	Brain_Anterior_cingulate_cortex_BA24	12	*AVIL*	58201929	rs12300079	0.9956	57273194	T	C	0.36	3.73E-03	1.61E-02	1.48E-01
rs4526799	Brain_Anterior_cingulate_cortex_BA24	12	*MYL6*	56554355	rs12300191	0.9956	57273289	A	G	0.36	3.59E-03	2.10E-02	1.52E-01
rs4526799	Brain_Anterior_cingulate_cortex_BA24	12	*CNPY2*	56706816	rs12300191	0.9956	57273289	A	G	0.36	3.59E-03	4.40E-02	1.76E-01
rs4526799	Brain_Anterior_cingulate_cortex_BA24	12	*BAZ2A*	57009990	rs12300191	0.9956	57273289	A	G	0.36	3.59E-03	2.80E-02	1.60E-01
rs4526799	Brain_Anterior_cingulate_cortex_BA24	12	*TMEM194A*	57465636	rs12300191	0.9956	57273289	A	G	0.36	3.59E-03	9.25E-04	1.10E-01
rs4526799	Brain_Anterior_cingulate_cortex_BA24	12	*INHBE*	57849584	rs12300191	0.9956	57273289	A	G	0.36	3.59E-03	1.93E-03	1.15E-01
rs4526799	Brain_Anterior_cingulate_cortex_BA24	12	*RP11-571M6.17*	58197909	rs12300191	0.9956	57273289	A	G	0.36	3.59E-03	4.87E-02	1.80E-01
rs4526799	Brain_Anterior_cingulate_cortex_BA24	12	*AVIL*	58201929	rs12300191	0.9956	57273289	A	G	0.36	3.59E-03	1.68E-02	1.47E-01
rs4526799	Brain_Anterior_cingulate_cortex_BA24	12	*MYL6*	56554355	rs4514464	0.9956	57276375	C	T	0.36	2.92E-03	2.10E-02	1.49E-01
rs4526799	Brain_Anterior_cingulate_cortex_BA24	12	*CNPY2*	56706816	rs4514464	0.9956	57276375	C	T	0.36	2.92E-03	4.40E-02	1.73E-01
rs4526799	Brain_Anterior_cingulate_cortex_BA24	12	*BAZ2A*	57009990	rs4514464	0.9956	57276375	C	T	0.36	2.92E-03	2.80E-02	1.57E-01
rs4526799	Brain_Anterior_cingulate_cortex_BA24	12	*TMEM194A*	57465636	rs4514464	0.9956	57276375	C	T	0.36	2.92E-03	9.25E-04	1.06E-01
rs4526799	Brain_Anterior_cingulate_cortex_BA24	12	*INHBE*	57849584	rs4514464	0.9956	57276375	C	T	0.36	2.92E-03	1.93E-03	1.12E-01
rs4526799	Brain_Anterior_cingulate_cortex_BA24	12	*RP11-571M6.17*	58197909	rs4514464	0.9956	57276375	C	T	0.36	2.92E-03	4.87E-02	1.77E-01
rs4526799	Brain_Anterior_cingulate_cortex_BA24	12	*AVIL*	58201929	rs4514464	0.9956	57276375	C	T	0.36	2.92E-03	1.68E-02	1.43E-01
rs4526799	Brain_Anterior_cingulate_cortex_BA24	12	*MYL6*	56554355	rs4417325	0.9956	57277302	G	A	0.36	3.59E-03	2.10E-02	1.52E-01
rs4526799	Brain_Anterior_cingulate_cortex_BA24	12	*CNPY2*	56706816	rs4417325	0.9956	57277302	G	A	0.36	3.59E-03	4.40E-02	1.76E-01
rs4526799	Brain_Anterior_cingulate_cortex_BA24	12	*BAZ2A*	57009990	rs4417325	0.9956	57277302	G	A	0.36	3.59E-03	2.80E-02	1.60E-01
rs4526799	Brain_Anterior_cingulate_cortex_BA24	12	*TMEM194A*	57465636	rs4417325	0.9956	57277302	G	A	0.36	3.59E-03	9.25E-04	1.10E-01
rs4526799	Brain_Anterior_cingulate_cortex_BA24	12	*INHBE*	57849584	rs4417325	0.9956	57277302	G	A	0.36	3.59E-03	1.93E-03	1.15E-01
rs4526799	Brain_Anterior_cingulate_cortex_BA24	12	*RP11-571M6.17*	58197909	rs4417325	0.9956	57277302	G	A	0.36	3.59E-03	4.87E-02	1.80E-01
rs4526799	Brain_Anterior_cingulate_cortex_BA24	12	*AVIL*	58201929	rs4417325	0.9956	57277302	G	A	0.36	3.59E-03	1.68E-02	1.47E-01
rs4526799	Brain_Anterior_cingulate_cortex_BA24	12	*MYL6*	56554355	rs11172030	0.9956	57278076	A	C	0.36	3.63E-03	2.10E-02	1.53E-01
rs4526799	Brain_Anterior_cingulate_cortex_BA24	12	*CNPY2*	56706816	rs11172030	0.9956	57278076	A	C	0.36	3.63E-03	4.40E-02	1.77E-01
rs4526799	Brain_Anterior_cingulate_cortex_BA24	12	*BAZ2A*	57009990	rs11172030	0.9956	57278076	A	C	0.36	3.63E-03	2.80E-02	1.61E-01
rs4526799	Brain_Anterior_cingulate_cortex_BA24	12	*TMEM194A*	57465636	rs11172030	0.9956	57278076	A	C	0.36	3.63E-03	9.25E-04	1.10E-01
rs4526799	Brain_Anterior_cingulate_cortex_BA24	12	*INHBE*	57849584	rs11172030	0.9956	57278076	A	C	0.36	3.63E-03	1.93E-03	1.16E-01
rs4526799	Brain_Anterior_cingulate_cortex_BA24	12	*RP11-571M6.17*	58197909	rs11172030	0.9956	57278076	A	C	0.36	3.63E-03	4.87E-02	1.81E-01
rs4526799	Brain_Anterior_cingulate_cortex_BA24	12	*AVIL*	58201929	rs11172030	0.9956	57278076	A	C	0.36	3.63E-03	1.68E-02	1.47E-01
rs4526799	Brain_Anterior_cingulate_cortex_BA24	12	*MYL6*	56554355	rs10876944	0.9956	57279372	T	A	0.36	3.63E-03	2.10E-02	1.53E-01
rs4526799	Brain_Anterior_cingulate_cortex_BA24	12	*CNPY2*	56706816	rs10876944	0.9956	57279372	T	A	0.36	3.63E-03	4.40E-02	1.77E-01
rs4526799	Brain_Anterior_cingulate_cortex_BA24	12	*BAZ2A*	57009990	rs10876944	0.9956	57279372	T	A	0.36	3.63E-03	2.80E-02	1.61E-01
rs4526799	Brain_Anterior_cingulate_cortex_BA24	12	*TMEM194A*	57465636	rs10876944	0.9956	57279372	T	A	0.36	3.63E-03	9.25E-04	1.10E-01
rs4526799	Brain_Anterior_cingulate_cortex_BA24	12	*INHBE*	57849584	rs10876944	0.9956	57279372	T	A	0.36	3.63E-03	1.93E-03	1.16E-01
rs4526799	Brain_Anterior_cingulate_cortex_BA24	12	*RP11-571M6.17*	58197909	rs10876944	0.9956	57279372	T	A	0.36	3.63E-03	4.87E-02	1.81E-01
rs4526799	Brain_Anterior_cingulate_cortex_BA24	12	*AVIL*	58201929	rs10876944	0.9956	57279372	T	A	0.36	3.63E-03	1.68E-02	1.47E-01
rs4526799	Brain_Anterior_cingulate_cortex_BA24	12	*MYL6*	56554355	rs4326839	0.9956	57280374	G	C	0.36	3.58E-03	2.10E-02	1.52E-01
rs4526799	Brain_Anterior_cingulate_cortex_BA24	12	*CNPY2*	56706816	rs4326839	0.9956	57280374	G	C	0.36	3.58E-03	4.40E-02	1.76E-01
rs4526799	Brain_Anterior_cingulate_cortex_BA24	12	*BAZ2A*	57009990	rs4326839	0.9956	57280374	G	C	0.36	3.58E-03	2.80E-02	1.60E-01

rs4526799	Brain_Anterior_cingulate_cortex_BA24	12	*TMEM194A*	57465636	rs4326839	0.9956	57280374	G	C	0.36	3.58E-03	9.25E-04	1.09E-01
rs4526799	Brain_Anterior_cingulate_cortex_BA24	12	*INHBE*	57849584	rs4326839	0.9956	57280374	G	C	0.36	3.58E-03	1.93E-03	1.15E-01
rs4526799	Brain_Anterior_cingulate_cortex_BA24	12	*RP11-571M6.17*	58197909	rs4326839	0.9956	57280374	G	C	0.36	3.58E-03	4.87E-02	1.80E-01
rs4526799	Brain_Anterior_cingulate_cortex_BA24	12	*AVIL*	58201929	rs4326839	0.9956	57280374	G	C	0.36	3.58E-03	1.68E-02	1.46E-01
rs4526799	Brain_Anterior_cingulate_cortex_BA24	12	*MYL6*	56554355	rs4526799	1.0000	57280586	T	C	0.34	7.26E-06	2.10E-02	5.30E-02
rs4526799	Brain_Anterior_cingulate_cortex_BA24	12	*CNPY2*	56706816	rs4526799	1.0000	57280586	T	C	0.34	7.26E-06	4.40E-02	7.98E-02
rs4526799	Brain_Anterior_cingulate_cortex_BA24	12	*BAZ2A*	57009990	rs4526799	1.0000	57280586	T	C	0.34	7.26E-06	2.80E-02	6.17E-02
rs4526799	Brain_Anterior_cingulate_cortex_BA24	12	*TMEM194A*	57465636	rs4526799	1.0000	57280586	T	C	0.34	7.26E-06	9.25E-04	1.54E-02
rs4526799	Brain_Anterior_cingulate_cortex_BA24	12	*INHBE*	57849584	rs4526799	1.0000	57280586	T	C	0.34	7.26E-06	1.93E-03	1.95E-02
rs4526799	Brain_Anterior_cingulate_cortex_BA24	12	*RP11-571M6.17*	58197909	rs4526799	1.0000	57280586	T	C	0.34	7.26E-06	4.87E-02	8.48E-02
rs4526799	Brain_Anterior_cingulate_cortex_BA24	12	*AVIL*	58201929	rs4526799	1.0000	57280586	T	C	0.34	7.26E-06	1.68E-02	4.73E-02
rs4526799	Brain_Anterior_cingulate_cortex_BA24	12	*MYL6*	56554355	rs28876529	0.9956	57285301	T	A	0.36	2.25E-03	2.10E-02	1.36E-01
rs4526799	Brain_Anterior_cingulate_cortex_BA24	12	*CNPY2*	56706816	rs28876529	0.9956	57285301	T	A	0.36	2.25E-03	4.40E-02	1.61E-01
rs4526799	Brain_Anterior_cingulate_cortex_BA24	12	*BAZ2A*	57009990	rs28876529	0.9956	57285301	T	A	0.36	2.25E-03	2.80E-02	1.45E-01
rs4526799	Brain_Anterior_cingulate_cortex_BA24	12	*TMEM194A*	57465636	rs28876529	0.9956	57285301	T	A	0.36	2.25E-03	9.25E-04	9.28E-02
rs4526799	Brain_Anterior_cingulate_cortex_BA24	12	*INHBE*	57849584	rs28876529	0.9956	57285301	T	A	0.36	2.25E-03	1.93E-03	9.88E-02
rs4526799	Brain_Anterior_cingulate_cortex_BA24	12	*RP11-571M6.17*	58197909	rs28876529	0.9956	57285301	T	A	0.36	2.25E-03	4.87E-02	1.66E-01
rs4526799	Brain_Anterior_cingulate_cortex_BA24	12	*AVIL*	58201929	rs28876529	0.9956	57285301	T	A	0.36	2.25E-03	1.68E-02	1.31E-01
rs4526799	Brain_Anterior_cingulate_cortex_BA24	12	*MYL6*	56554355	rs11172037	0.9956	57285427	T	A	0.36	2.25E-03	2.10E-02	1.36E-01
rs4526799	Brain_Anterior_cingulate_cortex_BA24	12	*CNPY2*	56706816	rs11172037	0.9956	57285427	T	A	0.36	2.25E-03	4.40E-02	1.61E-01
rs4526799	Brain_Anterior_cingulate_cortex_BA24	12	*BAZ2A*	57009990	rs11172037	0.9956	57285427	T	A	0.36	2.25E-03	2.80E-02	1.45E-01
rs4526799	Brain_Anterior_cingulate_cortex_BA24	12	*TMEM194A*	57465636	rs11172037	0.9956	57285427	T	A	0.36	2.25E-03	9.25E-04	9.28E-02
rs4526799	Brain_Anterior_cingulate_cortex_BA24	12	*INHBE*	57849584	rs11172037	0.9956	57285427	T	A	0.36	2.25E-03	1.93E-03	9.88E-02
rs4526799	Brain_Anterior_cingulate_cortex_BA24	12	*RP11-571M6.17*	58197909	rs11172037	0.9956	57285427	T	A	0.36	2.25E-03	4.87E-02	1.66E-01
rs4526799	Brain_Anterior_cingulate_cortex_BA24	12	*AVIL*	58201929	rs11172037	0.9956	57285427	T	A	0.36	2.25E-03	1.68E-02	1.31E-01
rs4526799	Brain_Anterior_cingulate_cortex_BA24	12	*MYL6*	56554355	rs12321987	0.9956	57288449	G	A	0.36	4.47E-03	2.10E-02	1.74E-01
rs4526799	Brain_Anterior_cingulate_cortex_BA24	12	*CNPY2*	56706816	rs12321987	0.9956	57288449	G	A	0.36	4.47E-03	4.40E-02	1.96E-01
rs4526799	Brain_Anterior_cingulate_cortex_BA24	12	*BAZ2A*	57009990	rs12321987	0.9956	57288449	G	A	0.36	4.47E-03	2.80E-02	1.81E-01
rs4526799	Brain_Anterior_cingulate_cortex_BA24	12	*TMEM194A*	57465636	rs12321987	0.9956	57288449	G	A	0.36	4.47E-03	9.25E-04	1.33E-01
rs4526799	Brain_Anterior_cingulate_cortex_BA24	12	*INHBE*	57849584	rs12321987	0.9956	57288449	G	A	0.36	4.47E-03	1.93E-03	1.39E-01
rs4526799	Brain_Anterior_cingulate_cortex_BA24	12	*RP11-571M6.17*	58197909	rs12321987	0.9956	57288449	G	A	0.36	4.47E-03	4.87E-02	2.00E-01
rs4526799	Brain_Anterior_cingulate_cortex_BA24	12	*AVIL*	58201929	rs12321987	0.9956	57288449	G	A	0.36	4.47E-03	1.68E-02	1.69E-01
rs4526799	Brain_Anterior_cingulate_cortex_BA24	12	*MYL6*	56554355	rs11172043	0.9869	57293182	G	A	0.35	4.58E-03	1.26E-02	1.61E-01
rs4526799	Brain_Anterior_cingulate_cortex_BA24	12	*BAZ2A*	57009990	rs11172043	0.9869	57293182	G	A	0.35	4.58E-03	2.32E-02	1.75E-01
rs4526799	Brain_Anterior_cingulate_cortex_BA24	12	*TMEM194A*	57465636	rs11172043	0.9869	57293182	G	A	0.35	4.58E-03	5.92E-04	1.29E-01
rs4526799	Brain_Anterior_cingulate_cortex_BA24	12	*INHBE*	57849584	rs11172043	0.9869	57293182	G	A	0.35	4.58E-03	9.49E-04	1.32E-01
rs4526799	Brain_Anterior_cingulate_cortex_BA24	12	*AVIL*	58201929	rs11172043	0.9869	57293182	G	A	0.35	4.58E-03	2.30E-02	1.75E-01
rs4526799	Brain_Anterior_cingulate_cortex_BA24	12	*MYL6*	56554355	rs12426816	0.9869	57294074	A	C	0.35	4.50E-03	1.26E-02	1.61E-01
rs4526799	Brain_Anterior_cingulate_cortex_BA24	12	*BAZ2A*	57009990	rs12426816	0.9869	57294074	A	C	0.35	4.50E-03	2.32E-02	1.74E-01
rs4526799	Brain_Anterior_cingulate_cortex_BA24	12	*TMEM194A*	57465636	rs12426816	0.9869	57294074	A	C	0.35	4.50E-03	5.92E-04	1.29E-01

rs4526799	Brain_Anterior_cingulate_cortex_BA24	12	*INHBE*	57849584	rs12426816	0.9869	57294074	A	C	0.35	4.50E-03	9.49E-04	1.32E-01
rs4526799	Brain_Anterior_cingulate_cortex_BA24	12	*AVIL*	58201929	rs12426816	0.9869	57294074	A	C	0.35	4.50E-03	2.30E-02	1.74E-01
rs4526799	Brain_Anterior_cingulate_cortex_BA24	12	*MYL6*	56554355	rs11172047	0.9869	57298080	T	C	0.35	6.51E-03	1.26E-02	1.76E-01
rs4526799	Brain_Anterior_cingulate_cortex_BA24	12	*BAZ2A*	57009990	rs11172047	0.9869	57298080	T	C	0.35	6.51E-03	2.32E-02	1.89E-01
rs4526799	Brain_Anterior_cingulate_cortex_BA24	12	*TMEM194A*	57465636	rs11172047	0.9869	57298080	T	C	0.35	6.51E-03	5.92E-04	1.45E-01
rs4526799	Brain_Anterior_cingulate_cortex_BA24	12	*INHBE*	57849584	rs11172047	0.9869	57298080	T	C	0.35	6.51E-03	9.49E-04	1.47E-01
rs4526799	Brain_Anterior_cingulate_cortex_BA24	12	*AVIL*	58201929	rs11172047	0.9869	57298080	T	C	0.35	6.51E-03	2.30E-02	1.89E-01
rs4526799	Brain_Anterior_cingulate_cortex_BA24	12	*MYL6*	56554355	rs2371631	0.9869	57298614	T	A	0.35	6.32E-03	1.26E-02	1.73E-01
rs4526799	Brain_Anterior_cingulate_cortex_BA24	12	*BAZ2A*	57009990	rs2371631	0.9869	57298614	T	A	0.35	6.32E-03	2.32E-02	1.86E-01
rs4526799	Brain_Anterior_cingulate_cortex_BA24	12	*TMEM194A*	57465636	rs2371631	0.9869	57298614	T	A	0.35	6.32E-03	5.92E-04	1.41E-01
rs4526799	Brain_Anterior_cingulate_cortex_BA24	12	*INHBE*	57849584	rs2371631	0.9869	57298614	T	A	0.35	6.32E-03	9.49E-04	1.44E-01
rs4526799	Brain_Anterior_cingulate_cortex_BA24	12	*AVIL*	58201929	rs2371631	0.9869	57298614	T	A	0.35	6.32E-03	2.30E-02	1.85E-01
rs4526799	Brain_Anterior_cingulate_cortex_BA24	12	*MYL6*	56554355	rs12305763	0.9869	57299263	G	A	0.35	6.82E-03	1.26E-02	1.77E-01
rs4526799	Brain_Anterior_cingulate_cortex_BA24	12	*BAZ2A*	57009990	rs12305763	0.9869	57299263	G	A	0.35	6.82E-03	2.32E-02	1.90E-01
rs4526799	Brain_Anterior_cingulate_cortex_BA24	12	*TMEM194A*	57465636	rs12305763	0.9869	57299263	G	A	0.35	6.82E-03	5.92E-04	1.46E-01
rs4526799	Brain_Anterior_cingulate_cortex_BA24	12	*INHBE*	57849584	rs12305763	0.9869	57299263	G	A	0.35	6.82E-03	9.49E-04	1.49E-01
rs4526799	Brain_Anterior_cingulate_cortex_BA24	12	*AVIL*	58201929	rs12305763	0.9869	57299263	G	A	0.35	6.82E-03	2.30E-02	1.90E-01
rs4526799	Brain_Anterior_cingulate_cortex_BA24	12	*MYL6*	56554355	rs11172049	0.9128	57304203	T	C	0.35	1.15E-02	6.61E-03	1.67E-01
rs4526799	Brain_Anterior_cingulate_cortex_BA24	12	*TMEM194A*	57465636	rs11172049	0.9128	57304203	T	C	0.35	1.15E-02	2.64E-04	1.42E-01
rs4526799	Brain_Anterior_cingulate_cortex_BA24	12	*INHBE*	57849584	rs11172049	0.9128	57304203	T	C	0.35	1.15E-02	4.92E-04	1.45E-01
rs4526799	Brain_Anterior_cingulate_cortex_BA24	12	*METTL21B*	58170856	rs11172049	0.9128	57304203	T	C	0.35	1.15E-02	4.78E-03	1.63E-01
rs4526799	Brain_Anterior_cingulate_cortex_BA24	12	*RP11-571M6.17*	58197909	rs11172049	0.9128	57304203	T	C	0.35	1.15E-02	2.89E-02	1.96E-01
rs4526799	Brain_Anterior_cingulate_cortex_BA24	12	*MYL6*	56554355	rs1874888	0.9085	57305138	A	C	0.35	1.15E-02	6.61E-03	1.67E-01
rs4526799	Brain_Anterior_cingulate_cortex_BA24	12	*TMEM194A*	57465636	rs1874888	0.9085	57305138	A	C	0.35	1.15E-02	2.64E-04	1.42E-01
rs4526799	Brain_Anterior_cingulate_cortex_BA24	12	*INHBE*	57849584	rs1874888	0.9085	57305138	A	C	0.35	1.15E-02	4.92E-04	1.45E-01
rs4526799	Brain_Anterior_cingulate_cortex_BA24	12	*METTL21B*	58170856	rs1874888	0.9085	57305138	A	C	0.35	1.15E-02	4.78E-03	1.63E-01
rs4526799	Brain_Anterior_cingulate_cortex_BA24	12	*RP11-571M6.17*	58197909	rs1874888	0.9085	57305138	A	C	0.35	1.15E-02	2.89E-02	1.96E-01
rs4526799	Brain_Anterior_cingulate_cortex_BA24	12	*MYL6*	56554355	rs10506349	0.9128	57306412	T	C	0.37	1.10E-02	3.85E-03	1.51E-01
rs4526799	Brain_Anterior_cingulate_cortex_BA24	12	*TMEM194A*	57465636	rs10506349	0.9128	57306412	T	C	0.37	1.10E-02	5.00E-04	1.35E-01
rs4526799	Brain_Anterior_cingulate_cortex_BA24	12	*INHBE*	57849584	rs10506349	0.9128	57306412	T	C	0.37	1.10E-02	2.33E-04	1.31E-01
rs4526799	Brain_Anterior_cingulate_cortex_BA24	12	*METTL21B*	58170856	rs10506349	0.9128	57306412	T	C	0.37	1.10E-02	2.21E-02	1.80E-01
rs4526799	Brain_Anterior_cingulate_cortex_BA24	12	*RP11-571M6.17*	58197909	rs10506349	0.9128	57306412	T	C	0.37	1.10E-02	1.10E-02	1.65E-01
rs4526799	Brain_Anterior_cingulate_cortex_BA24	12	*AVIL*	58201929	rs10506349	0.9128	57306412	T	C	0.37	1.10E-02	4.03E-02	1.98E-01
rs4526799	Brain_Anterior_cingulate_cortex_BA24	12	*MYL6*	56554355	rs10876951	0.9085	57306430	T	G	0.35	1.15E-02	6.61E-03	1.67E-01
rs4526799	Brain_Anterior_cingulate_cortex_BA24	12	*TMEM194A*	57465636	rs10876951	0.9085	57306430	T	G	0.35	1.15E-02	2.64E-04	1.42E-01
rs4526799	Brain_Anterior_cingulate_cortex_BA24	12	*INHBE*	57849584	rs10876951	0.9085	57306430	T	G	0.35	1.15E-02	4.92E-04	1.45E-01
rs4526799	Brain_Anterior_cingulate_cortex_BA24	12	*METTL21B*	58170856	rs10876951	0.9085	57306430	T	G	0.35	1.15E-02	4.78E-03	1.63E-01
rs4526799	Brain_Anterior_cingulate_cortex_BA24	12	*RP11-571M6.17*	58197909	rs10876951	0.9085	57306430	T	G	0.35	1.15E-02	2.89E-02	1.96E-01
rs4526799	Brain_Anterior_cingulate_cortex_BA24	12	*MYL6*	56554355	rs10747774	0.9128	57307079	T	C	0.36	1.16E-02	3.85E-03	1.57E-01
rs4526799	Brain_Anterior_cingulate_cortex_BA24	12	*TMEM194A*	57465636	rs10747774	0.9128	57307079	T	C	0.36	1.16E-02	5.00E-04	1.41E-01

rs4526799	Brain_Anterior_cingulate_cortex_BA24	12	*INHBE*	57849584	rs10747774	0.9128	57307079	T	C	0.36	1.16E-02	2.33E-04	1.37E-01
rs4526799	Brain_Anterior_cingulate_cortex_BA24	12	*METTL21B*	58170856	rs10747774	0.9128	57307079	T	C	0.36	1.16E-02	2.21E-02	1.85E-01
rs4526799	Brain_Anterior_cingulate_cortex_BA24	12	*RP11-571M6.17*	58197909	rs10747774	0.9128	57307079	T	C	0.36	1.16E-02	1.10E-02	1.71E-01
rs4526799	Brain_Anterior_cingulate_cortex_BA24	12	*AVIL*	58201929	rs10747774	0.9128	57307079	T	C	0.36	1.16E-02	4.03E-02	2.03E-01
rs4526799	Brain_Anterior_cingulate_cortex_BA24	12	*MYL6*	56554355	rs10783812	0.9085	57308723	C	T	0.35	1.19E-02	5.76E-03	1.69E-01
rs4526799	Brain_Anterior_cingulate_cortex_BA24	12	*TMEM194A*	57465636	rs10783812	0.9085	57308723	C	T	0.35	1.19E-02	2.81E-04	1.46E-01
rs4526799	Brain_Anterior_cingulate_cortex_BA24	12	*INHBE*	57849584	rs10783812	0.9085	57308723	C	T	0.35	1.19E-02	3.50E-04	1.47E-01
rs4526799	Brain_Anterior_cingulate_cortex_BA24	12	*METTL21B*	58170856	rs10783812	0.9085	57308723	C	T	0.35	1.19E-02	5.07E-03	1.67E-01
rs4526799	Brain_Anterior_cingulate_cortex_BA24	12	*RP11-571M6.17*	58197909	rs10783812	0.9085	57308723	C	T	0.35	1.19E-02	2.62E-02	1.96E-01
rs4526799	Brain_Anterior_cingulate_cortex_BA24	12	*MYL6*	56554355	rs11172056	0.9089	57308975	C	T	0.37	1.10E-02	3.85E-03	1.51E-01
rs4526799	Brain_Anterior_cingulate_cortex_BA24	12	*TMEM194A*	57465636	rs11172056	0.9089	57308975	C	T	0.37	1.10E-02	5.00E-04	1.35E-01
rs4526799	Brain_Anterior_cingulate_cortex_BA24	12	*INHBE*	57849584	rs11172056	0.9089	57308975	C	T	0.37	1.10E-02	2.33E-04	1.31E-01
rs4526799	Brain_Anterior_cingulate_cortex_BA24	12	*METTL21B*	58170856	rs11172056	0.9089	57308975	C	T	0.37	1.10E-02	2.21E-02	1.80E-01
rs4526799	Brain_Anterior_cingulate_cortex_BA24	12	*RP11-571M6.17*	58197909	rs11172056	0.9089	57308975	C	T	0.37	1.10E-02	1.10E-02	1.66E-01
rs4526799	Brain_Anterior_cingulate_cortex_BA24	12	*AVIL*	58201929	rs11172056	0.9089	57308975	C	T	0.37	1.10E-02	4.03E-02	1.98E-01
rs4526799	Brain_Anterior_cingulate_cortex_BA24	12	*MYL6*	56554355	rs7302420	0.9085	57309884	G	C	0.36	1.54E-02	4.31E-03	1.83E-01
rs4526799	Brain_Anterior_cingulate_cortex_BA24	12	*TMEM194A*	57465636	rs7302420	0.9085	57309884	G	C	0.36	1.54E-02	2.85E-04	1.64E-01
rs4526799	Brain_Anterior_cingulate_cortex_BA24	12	*INHBE*	57849584	rs7302420	0.9085	57309884	G	C	0.36	1.54E-02	6.95E-04	1.69E-01
rs4526799	Brain_Anterior_cingulate_cortex_BA24	12	*METTL21B*	58170856	rs7302420	0.9085	57309884	G	C	0.36	1.54E-02	5.46E-03	1.85E-01
rs4526799	Brain_Anterior_cingulate_cortex_BA24	12	*RP11-571M6.17*	58197909	rs7302420	0.9085	57309884	G	C	0.36	1.54E-02	3.33E-02	2.19E-01
rs4526799	Brain_Anterior_cingulate_cortex_BA24	12	*AVIL*	58201929	rs7302420	0.9085	57309884	G	C	0.36	1.54E-02	4.28E-02	2.27E-01
rs4526799	Brain_Anterior_cingulate_cortex_BA24	12	*MYL6*	56554355	rs12228618	0.9128	57311229	T	C	0.37	1.29E-02	6.64E-03	1.67E-01
rs4526799	Brain_Anterior_cingulate_cortex_BA24	12	*TMEM194A*	57465636	rs12228618	0.9128	57311229	T	C	0.37	1.29E-02	1.51E-03	1.52E-01
rs4526799	Brain_Anterior_cingulate_cortex_BA24	12	*INHBE*	57849584	rs12228618	0.9128	57311229	T	C	0.37	1.29E-02	1.70E-03	1.53E-01
rs4526799	Brain_Anterior_cingulate_cortex_BA24	12	*ARHGAP9*	57874321	rs12228618	0.9128	57311229	T	C	0.37	1.29E-02	4.75E-02	2.12E-01
rs4526799	Brain_Anterior_cingulate_cortex_BA24	12	*RP11-571M6.17*	58197909	rs12228618	0.9128	57311229	T	C	0.37	1.29E-02	1.00E-02	1.73E-01
rs4526799	Brain_Anterior_cingulate_cortex_BA24	12	*AVIL*	58201929	rs12228618	0.9128	57311229	T	C	0.37	1.29E-02	4.95E-02	2.14E-01
rs4526799	Brain_Anterior_cingulate_cortex_BA24	12	*MYL6*	56554355	rs9739473	0.9012	57313335	A	T	0.37	2.65E-02	6.77E-03	2.40E-01
rs4526799	Brain_Anterior_cingulate_cortex_BA24	12	*TMEM194A*	57465636	rs9739473	0.9012	57313335	A	T	0.37	2.65E-02	6.60E-04	2.24E-01
rs4526799	Brain_Anterior_cingulate_cortex_BA24	12	*INHBE*	57849584	rs9739473	0.9012	57313335	A	T	0.37	2.65E-02	3.40E-04	2.21E-01
rs4526799	Brain_Anterior_cingulate_cortex_BA24	12	*METTL21B*	58170856	rs9739473	0.9012	57313335	A	T	0.37	2.65E-02	4.59E-02	2.75E-01
rs4526799	Brain_Anterior_cingulate_cortex_BA24	12	*RP11-571M6.17*	58197909	rs9739473	0.9012	57313335	A	T	0.37	2.65E-02	7.24E-03	2.41E-01
rs4526799	Brain_Anterior_cingulate_cortex_BA24	12	*AVIL*	58201929	rs9739473	0.9012	57313335	A	T	0.37	2.65E-02	3.24E-02	2.66E-01
rs4526799	Brain_Caudate_basal_ganglia	12	*RPS26*	56436876	rs9919772	0.8450	57260027	T	C	0.33	1.03E-02	1.08E-02	1.98E-01
rs4526799	Brain_Caudate_basal_ganglia	12	*PA2G4*	56502897	rs9919772	0.8450	57260027	T	C	0.33	1.03E-02	2.91E-02	2.19E-01
rs4526799	Brain_Caudate_basal_ganglia	12	*RP11-603J24.5*	56518610	rs9919772	0.8450	57260027	T	C	0.33	1.03E-02	1.41E-02	2.03E-01
rs4526799	Brain_Caudate_basal_ganglia	12	*ANKRD52*	56641903	rs9919772	0.8450	57260027	T	C	0.33	1.03E-02	1.65E-02	2.06E-01
rs4526799	Brain_Caudate_basal_ganglia	12	*COQ10A*	56662696	rs9919772	0.8450	57260027	T	C	0.33	1.03E-02	1.28E-02	2.01E-01
rs4526799	Brain_Caudate_basal_ganglia	12	*IL23A*	56733428	rs9919772	0.8450	57260027	T	C	0.33	1.03E-02	1.23E-02	2.01E-01
rs4526799	Brain_Caudate_basal_ganglia	12	*RN7SL809P*	57064383	rs9919772	0.8450	57260027	T	C	0.33	1.03E-02	3.37E-02	2.23E-01

rs4526799	Brain_Caudate_basal_ganglia	12	*HSD17B6*	57163759	rs9919772	0.8450	57260027	T	C	0.33	1.03E-02	2.88E-02	2.18E-01
rs4526799	Brain_Caudate_basal_ganglia	12	*RP11-74M13.4*	57242410	rs9919772	0.8450	57260027	T	C	0.33	1.03E-02	3.87E-02	2.27E-01
rs4526799	Brain_Caudate_basal_ganglia	12	*AGAP2-AS1*	58121424	rs9919772	0.8450	57260027	T	C	0.33	1.03E-02	4.81E-02	2.34E-01
rs4526799	Brain_Caudate_basal_ganglia	12	*RPS26*	56436876	rs4495925	0.8650	57268116	C	G	0.33	1.04E-02	3.15E-03	1.80E-01
rs4526799	Brain_Caudate_basal_ganglia	12	*PA2G4*	56502897	rs4495925	0.8650	57268116	C	G	0.33	1.04E-02	2.88E-02	2.15E-01
rs4526799	Brain_Caudate_basal_ganglia	12	*RP11-603J24.5*	56518610	rs4495925	0.8650	57268116	C	G	0.33	1.04E-02	3.20E-02	2.18E-01
rs4526799	Brain_Caudate_basal_ganglia	12	*ANKRD52*	56641903	rs4495925	0.8650	57268116	C	G	0.33	1.04E-02	4.21E-02	2.27E-01
rs4526799	Brain_Caudate_basal_ganglia	12	*COQ10A*	56662696	rs4495925	0.8650	57268116	C	G	0.33	1.04E-02	1.39E-02	2.00E-01
rs4526799	Brain_Caudate_basal_ganglia	12	*IL23A*	56733428	rs4495925	0.8650	57268116	C	G	0.33	1.04E-02	1.40E-02	2.00E-01
rs4526799	Brain_Caudate_basal_ganglia	12	*RN7SL809P*	57064383	rs4495925	0.8650	57268116	C	G	0.33	1.04E-02	1.76E-02	2.04E-01
rs4526799	Brain_Caudate_basal_ganglia	12	*HSD17B6*	57163759	rs4495925	0.8650	57268116	C	G	0.33	1.04E-02	2.68E-02	2.14E-01
rs4526799	Brain_Caudate_basal_ganglia	12	*RPS26*	56436876	rs4471472	0.8650	57268985	A	G	0.32	9.99E-03	3.67E-03	1.78E-01
rs4526799	Brain_Caudate_basal_ganglia	12	*PA2G4*	56502897	rs4471472	0.8650	57268985	A	G	0.32	9.99E-03	2.64E-02	2.09E-01
rs4526799	Brain_Caudate_basal_ganglia	12	*RP11-603J24.5*	56518610	rs4471472	0.8650	57268985	A	G	0.32	9.99E-03	2.73E-02	2.10E-01
rs4526799	Brain_Caudate_basal_ganglia	12	*ANKRD52*	56641903	rs4471472	0.8650	57268985	A	G	0.32	9.99E-03	4.08E-02	2.22E-01
rs4526799	Brain_Caudate_basal_ganglia	12	*COQ10A*	56662696	rs4471472	0.8650	57268985	A	G	0.32	9.99E-03	1.25E-02	1.94E-01
rs4526799	Brain_Caudate_basal_ganglia	12	*IL23A*	56733428	rs4471472	0.8650	57268985	A	G	0.32	9.99E-03	1.14E-02	1.92E-01
rs4526799	Brain_Caudate_basal_ganglia	12	*RN7SL809P*	57064383	rs4471472	0.8650	57268985	A	G	0.32	9.99E-03	2.06E-02	2.03E-01
rs4526799	Brain_Caudate_basal_ganglia	12	*HSD17B6*	57163759	rs4471472	0.8650	57268985	A	G	0.32	9.99E-03	3.11E-02	2.14E-01
rs4526799	Brain_Caudate_basal_ganglia	12	*RPS26*	56436876	rs4633499	0.8610	57269264	A	T	0.32	1.43E-02	3.70E-03	2.16E-01
rs4526799	Brain_Caudate_basal_ganglia	12	*PA2G4*	56502897	rs4633499	0.8610	57269264	A	T	0.32	1.43E-02	1.59E-02	2.34E-01
rs4526799	Brain_Caudate_basal_ganglia	12	*RP11-603J24.5*	56518610	rs4633499	0.8610	57269264	A	T	0.32	1.43E-02	3.09E-02	2.48E-01
rs4526799	Brain_Caudate_basal_ganglia	12	*ANKRD52*	56641903	rs4633499	0.8610	57269264	A	T	0.32	1.43E-02	3.46E-02	2.51E-01
rs4526799	Brain_Caudate_basal_ganglia	12	*COQ10A*	56662696	rs4633499	0.8610	57269264	A	T	0.32	1.43E-02	1.33E-02	2.31E-01
rs4526799	Brain_Caudate_basal_ganglia	12	*IL23A*	56733428	rs4633499	0.8610	57269264	A	T	0.32	1.43E-02	8.46E-03	2.25E-01
rs4526799	Brain_Caudate_basal_ganglia	12	*RN7SL809P*	57064383	rs4633499	0.8610	57269264	A	T	0.32	1.43E-02	3.03E-02	2.47E-01
rs4526799	Brain_Caudate_basal_ganglia	12	*HSD17B6*	57163759	rs4633499	0.8610	57269264	A	T	0.32	1.43E-02	2.51E-02	2.43E-01
rs4526799	Brain_Caudate_basal_ganglia	12	*RP11-74M13.4*	57242410	rs4633499	0.8610	57269264	A	T	0.32	1.43E-02	4.97E-02	2.62E-01
rs4526799	Brain_Caudate_basal_ganglia	12	*PA2G4*	56502897	rs12300079	0.9956	57273194	T	C	0.36	3.73E-03	1.52E-02	1.47E-01
rs4526799	Brain_Caudate_basal_ganglia	12	*COQ10A*	56662696	rs12300079	0.9956	57273194	T	C	0.36	3.73E-03	3.05E-02	1.65E-01
rs4526799	Brain_Caudate_basal_ganglia	12	*IL23A*	56733428	rs12300079	0.9956	57273194	T	C	0.36	3.73E-03	1.41E-02	1.45E-01
rs4526799	Brain_Caudate_basal_ganglia	12	*RP11-74M13.4*	57242410	rs12300079	0.9956	57273194	T	C	0.36	3.73E-03	1.44E-02	1.46E-01
rs4526799	Brain_Caudate_basal_ganglia	12	*MYO1A*	57433641	rs12300079	0.9956	57273194	T	C	0.36	3.73E-03	2.36E-02	1.57E-01
rs4526799	Brain_Caudate_basal_ganglia	12	*PA2G4*	56502897	rs12300191	0.9956	57273289	A	G	0.36	3.59E-03	1.56E-02	1.45E-01
rs4526799	Brain_Caudate_basal_ganglia	12	*COQ10A*	56662696	rs12300191	0.9956	57273289	A	G	0.36	3.59E-03	2.82E-02	1.60E-01
rs4526799	Brain_Caudate_basal_ganglia	12	*IL23A*	56733428	rs12300191	0.9956	57273289	A	G	0.36	3.59E-03	1.34E-02	1.42E-01
rs4526799	Brain_Caudate_basal_ganglia	12	*RP11-74M13.4*	57242410	rs12300191	0.9956	57273289	A	G	0.36	3.59E-03	1.97E-02	1.50E-01
rs4526799	Brain_Caudate_basal_ganglia	12	*MYO1A*	57433641	rs12300191	0.9956	57273289	A	G	0.36	3.59E-03	1.86E-02	1.49E-01
rs4526799	Brain_Caudate_basal_ganglia	12	*PA2G4*	56502897	rs4514464	0.9956	57276375	C	T	0.36	2.92E-03	1.56E-02	1.42E-01
rs4526799	Brain_Caudate_basal_ganglia	12	*COQ10A*	56662696	rs4514464	0.9956	57276375	C	T	0.36	2.92E-03	2.82E-02	1.57E-01

rs4526799	Brain_Caudate_basal_ganglia	12	*IL23A*	56733428	rs4514464	0.9956	57276375	C	T	0.36	2.92E-03	1.34E-02	1.38E-01
rs4526799	Brain_Caudate_basal_ganglia	12	*RP11-74M13.4*	57242410	rs4514464	0.9956	57276375	C	T	0.36	2.92E-03	1.97E-02	1.47E-01
rs4526799	Brain_Caudate_basal_ganglia	12	*MYO1A*	57433641	rs4514464	0.9956	57276375	C	T	0.36	2.92E-03	1.86E-02	1.46E-01
rs4526799	Brain_Caudate_basal_ganglia	12	*PA2G4*	56502897	rs4417325	0.9956	57277302	G	A	0.36	3.59E-03	1.56E-02	1.45E-01
rs4526799	Brain_Caudate_basal_ganglia	12	*COQ10A*	56662696	rs4417325	0.9956	57277302	G	A	0.36	3.59E-03	2.82E-02	1.60E-01
rs4526799	Brain_Caudate_basal_ganglia	12	*IL23A*	56733428	rs4417325	0.9956	57277302	G	A	0.36	3.59E-03	1.34E-02	1.42E-01
rs4526799	Brain_Caudate_basal_ganglia	12	*RP11-74M13.4*	57242410	rs4417325	0.9956	57277302	G	A	0.36	3.59E-03	1.97E-02	1.50E-01
rs4526799	Brain_Caudate_basal_ganglia	12	*MYO1A*	57433641	rs4417325	0.9956	57277302	G	A	0.36	3.59E-03	1.86E-02	1.49E-01
rs4526799	Brain_Caudate_basal_ganglia	12	*PA2G4*	56502897	rs11172030	0.9956	57278076	A	C	0.36	3.63E-03	1.56E-02	1.45E-01
rs4526799	Brain_Caudate_basal_ganglia	12	*COQ10A*	56662696	rs11172030	0.9956	57278076	A	C	0.36	3.63E-03	2.82E-02	1.61E-01
rs4526799	Brain_Caudate_basal_ganglia	12	*IL23A*	56733428	rs11172030	0.9956	57278076	A	C	0.36	3.63E-03	1.34E-02	1.42E-01
rs4526799	Brain_Caudate_basal_ganglia	12	*RP11-74M13.4*	57242410	rs11172030	0.9956	57278076	A	C	0.36	3.63E-03	1.97E-02	1.51E-01
rs4526799	Brain_Caudate_basal_ganglia	12	*MYO1A*	57433641	rs11172030	0.9956	57278076	A	C	0.36	3.63E-03	1.86E-02	1.49E-01
rs4526799	Brain_Caudate_basal_ganglia	12	*PA2G4*	56502897	rs10876944	0.9956	57279372	T	A	0.36	3.63E-03	1.56E-02	1.45E-01
rs4526799	Brain_Caudate_basal_ganglia	12	*COQ10A*	56662696	rs10876944	0.9956	57279372	T	A	0.36	3.63E-03	2.82E-02	1.61E-01
rs4526799	Brain_Caudate_basal_ganglia	12	*IL23A*	56733428	rs10876944	0.9956	57279372	T	A	0.36	3.63E-03	1.34E-02	1.42E-01
rs4526799	Brain_Caudate_basal_ganglia	12	*RP11-74M13.4*	57242410	rs10876944	0.9956	57279372	T	A	0.36	3.63E-03	1.97E-02	1.51E-01
rs4526799	Brain_Caudate_basal_ganglia	12	*MYO1A*	57433641	rs10876944	0.9956	57279372	T	A	0.36	3.63E-03	1.86E-02	1.49E-01
rs4526799	Brain_Caudate_basal_ganglia	12	*PA2G4*	56502897	rs4326839	0.9956	57280374	G	C	0.36	3.58E-03	1.56E-02	1.45E-01
rs4526799	Brain_Caudate_basal_ganglia	12	*COQ10A*	56662696	rs4326839	0.9956	57280374	G	C	0.36	3.58E-03	2.82E-02	1.60E-01
rs4526799	Brain_Caudate_basal_ganglia	12	*IL23A*	56733428	rs4326839	0.9956	57280374	G	C	0.36	3.58E-03	1.34E-02	1.42E-01
rs4526799	Brain_Caudate_basal_ganglia	12	*RP11-74M13.4*	57242410	rs4326839	0.9956	57280374	G	C	0.36	3.58E-03	1.97E-02	1.50E-01
rs4526799	Brain_Caudate_basal_ganglia	12	*MYO1A*	57433641	rs4326839	0.9956	57280374	G	C	0.36	3.58E-03	1.86E-02	1.49E-01
rs4526799	Brain_Caudate_basal_ganglia	12	*PA2G4*	56502897	rs4526799	1.0000	57280586	T	C	0.34	7.26E-06	1.56E-02	4.56E-02
rs4526799	Brain_Caudate_basal_ganglia	12	*COQ10A*	56662696	rs4526799	1.0000	57280586	T	C	0.34	7.26E-06	2.82E-02	6.19E-02
rs4526799	Brain_Caudate_basal_ganglia	12	*IL23A*	56733428	rs4526799	1.0000	57280586	T	C	0.34	7.26E-06	1.34E-02	4.24E-02
rs4526799	Brain_Caudate_basal_ganglia	12	*RP11-74M13.4*	57242410	rs4526799	1.0000	57280586	T	C	0.34	7.26E-06	1.97E-02	5.12E-02
rs4526799	Brain_Caudate_basal_ganglia	12	*MYO1A*	57433641	rs4526799	1.0000	57280586	T	C	0.34	7.26E-06	1.86E-02	4.97E-02
rs4526799	Brain_Caudate_basal_ganglia	12	*PA2G4*	56502897	rs28876529	0.9956	57285301	T	A	0.36	2.25E-03	1.56E-02	1.29E-01
rs4526799	Brain_Caudate_basal_ganglia	12	*COQ10A*	56662696	rs28876529	0.9956	57285301	T	A	0.36	2.25E-03	2.82E-02	1.45E-01
rs4526799	Brain_Caudate_basal_ganglia	12	*IL23A*	56733428	rs28876529	0.9956	57285301	T	A	0.36	2.25E-03	1.34E-02	1.26E-01
rs4526799	Brain_Caudate_basal_ganglia	12	*RP11-74M13.4*	57242410	rs28876529	0.9956	57285301	T	A	0.36	2.25E-03	1.97E-02	1.35E-01
rs4526799	Brain_Caudate_basal_ganglia	12	*MYO1A*	57433641	rs28876529	0.9956	57285301	T	A	0.36	2.25E-03	1.86E-02	1.33E-01
rs4526799	Brain_Caudate_basal_ganglia	12	*PA2G4*	56502897	rs11172037	0.9956	57285427	T	A	0.36	2.25E-03	1.56E-02	1.29E-01
rs4526799	Brain_Caudate_basal_ganglia	12	*COQ10A*	56662696	rs11172037	0.9956	57285427	T	A	0.36	2.25E-03	2.82E-02	1.45E-01
rs4526799	Brain_Caudate_basal_ganglia	12	*IL23A*	56733428	rs11172037	0.9956	57285427	T	A	0.36	2.25E-03	1.34E-02	1.26E-01
rs4526799	Brain_Caudate_basal_ganglia	12	*RP11-74M13.4*	57242410	rs11172037	0.9956	57285427	T	A	0.36	2.25E-03	1.97E-02	1.35E-01
rs4526799	Brain_Caudate_basal_ganglia	12	*MYO1A*	57433641	rs11172037	0.9956	57285427	T	A	0.36	2.25E-03	1.86E-02	1.33E-01
rs4526799	Brain_Caudate_basal_ganglia	12	*PA2G4*	56502897	rs12321987	0.9956	57288449	G	A	0.36	4.47E-03	1.56E-02	1.67E-01
rs4526799	Brain_Caudate_basal_ganglia	12	*COQ10A*	56662696	rs12321987	0.9956	57288449	G	A	0.36	4.47E-03	2.82E-02	1.82E-01

rs4526799	Brain_Caudate_basal_ganglia	12	*IL23A*	56733428	rs12321987	0.9956	57288449	G	A	0.36	4.47E-03	1.34E-02	1.64E-01
rs4526799	Brain_Caudate_basal_ganglia	12	*RP11-74M13.4*	57242410	rs12321987	0.9956	57288449	G	A	0.36	4.47E-03	1.97E-02	1.72E-01
rs4526799	Brain_Caudate_basal_ganglia	12	*MYO1A*	57433641	rs12321987	0.9956	57288449	G	A	0.36	4.47E-03	1.86E-02	1.71E-01
rs4526799	Brain_Caudate_basal_ganglia	12	*IL23A*	56733428	rs11172043	0.9869	57293182	G	A	0.35	4.58E-03	3.00E-02	1.82E-01
rs4526799	Brain_Caudate_basal_ganglia	12	*HSD17B6*	57163759	rs11172043	0.9869	57293182	G	A	0.35	4.58E-03	2.99E-02	1.82E-01
rs4526799	Brain_Caudate_basal_ganglia	12	*RP11-74M13.4*	57242410	rs11172043	0.9869	57293182	G	A	0.35	4.58E-03	3.02E-02	1.82E-01
rs4526799	Brain_Caudate_basal_ganglia	12	*IL23A*	56733428	rs12426816	0.9869	57294074	A	C	0.35	4.50E-03	3.00E-02	1.82E-01
rs4526799	Brain_Caudate_basal_ganglia	12	*HSD17B6*	57163759	rs12426816	0.9869	57294074	A	C	0.35	4.50E-03	2.99E-02	1.82E-01
rs4526799	Brain_Caudate_basal_ganglia	12	*RP11-74M13.4*	57242410	rs12426816	0.9869	57294074	A	C	0.35	4.50E-03	3.02E-02	1.82E-01
rs4526799	Brain_Caudate_basal_ganglia	12	*IL23A*	56733428	rs11172047	0.9869	57298080	T	C	0.35	6.51E-03	3.00E-02	1.96E-01
rs4526799	Brain_Caudate_basal_ganglia	12	*HSD17B6*	57163759	rs11172047	0.9869	57298080	T	C	0.35	6.51E-03	2.99E-02	1.96E-01
rs4526799	Brain_Caudate_basal_ganglia	12	*RP11-74M13.4*	57242410	rs11172047	0.9869	57298080	T	C	0.35	6.51E-03	3.02E-02	1.96E-01
rs4526799	Brain_Caudate_basal_ganglia	12	*IL23A*	56733428	rs2371631	0.9869	57298614	T	A	0.35	6.32E-03	3.00E-02	1.93E-01
rs4526799	Brain_Caudate_basal_ganglia	12	*HSD17B6*	57163759	rs2371631	0.9869	57298614	T	A	0.35	6.32E-03	2.99E-02	1.93E-01
rs4526799	Brain_Caudate_basal_ganglia	12	*RP11-74M13.4*	57242410	rs2371631	0.9869	57298614	T	A	0.35	6.32E-03	3.02E-02	1.93E-01
rs4526799	Brain_Caudate_basal_ganglia	12	*IL23A*	56733428	rs12305763	0.9869	57299263	G	A	0.35	6.82E-03	3.00E-02	1.97E-01
rs4526799	Brain_Caudate_basal_ganglia	12	*HSD17B6*	57163759	rs12305763	0.9869	57299263	G	A	0.35	6.82E-03	2.99E-02	1.97E-01
rs4526799	Brain_Caudate_basal_ganglia	12	*RP11-74M13.4*	57242410	rs12305763	0.9869	57299263	G	A	0.35	6.82E-03	3.02E-02	1.97E-01
rs4526799	Brain_Caudate_basal_ganglia	12	*PA2G4*	56502897	rs11172049	0.9128	57304203	T	C	0.35	1.15E-02	3.58E-02	2.03E-01
rs4526799	Brain_Caudate_basal_ganglia	12	*IL23A*	56733428	rs11172049	0.9128	57304203	T	C	0.35	1.15E-02	3.21E-02	1.99E-01
rs4526799	Brain_Caudate_basal_ganglia	12	*RP11-74M13.4*	57242410	rs11172049	0.9128	57304203	T	C	0.35	1.15E-02	2.40E-02	1.91E-01
rs4526799	Brain_Caudate_basal_ganglia	12	*PA2G4*	56502897	rs1874888	0.9085	57305138	A	C	0.35	1.15E-02	3.58E-02	2.03E-01
rs4526799	Brain_Caudate_basal_ganglia	12	*IL23A*	56733428	rs1874888	0.9085	57305138	A	C	0.35	1.15E-02	3.21E-02	1.99E-01
rs4526799	Brain_Caudate_basal_ganglia	12	*RP11-74M13.4*	57242410	rs1874888	0.9085	57305138	A	C	0.35	1.15E-02	2.40E-02	1.91E-01
rs4526799	Brain_Caudate_basal_ganglia	12	*PA2G4*	56502897	rs10506349	0.9128	57306412	T	C	0.37	1.10E-02	2.78E-02	1.86E-01
rs4526799	Brain_Caudate_basal_ganglia	12	*COQ10A*	56662696	rs10506349	0.9128	57306412	T	C	0.37	1.10E-02	1.93E-02	1.77E-01
rs4526799	Brain_Caudate_basal_ganglia	12	*IL23A*	56733428	rs10506349	0.9128	57306412	T	C	0.37	1.10E-02	4.77E-02	2.04E-01
rs4526799	Brain_Caudate_basal_ganglia	12	*HSD17B6*	57163759	rs10506349	0.9128	57306412	T	C	0.37	1.10E-02	4.68E-02	2.03E-01
rs4526799	Brain_Caudate_basal_ganglia	12	*RP11-74M13.4*	57242410	rs10506349	0.9128	57306412	T	C	0.37	1.10E-02	2.52E-02	1.83E-01
rs4526799	Brain_Caudate_basal_ganglia	12	*PA2G4*	56502897	rs10876951	0.9085	57306430	T	G	0.35	1.15E-02	3.58E-02	2.03E-01
rs4526799	Brain_Caudate_basal_ganglia	12	*IL23A*	56733428	rs10876951	0.9085	57306430	T	G	0.35	1.15E-02	3.21E-02	1.99E-01
rs4526799	Brain_Caudate_basal_ganglia	12	*RP11-74M13.4*	57242410	rs10876951	0.9085	57306430	T	G	0.35	1.15E-02	2.40E-02	1.91E-01
rs4526799	Brain_Caudate_basal_ganglia	12	*PA2G4*	56502897	rs10747774	0.9128	57307079	T	C	0.36	1.16E-02	2.41E-02	1.87E-01
rs4526799	Brain_Caudate_basal_ganglia	12	*COQ10A*	56662696	rs10747774	0.9128	57307079	T	C	0.36	1.16E-02	2.06E-02	1.83E-01
rs4526799	Brain_Caudate_basal_ganglia	12	*IL23A*	56733428	rs10747774	0.9128	57307079	T	C	0.36	1.16E-02	4.89E-02	2.10E-01
rs4526799	Brain_Caudate_basal_ganglia	12	*HSD17B6*	57163759	rs10747774	0.9128	57307079	T	C	0.36	1.16E-02	4.21E-02	2.04E-01
rs4526799	Brain_Caudate_basal_ganglia	12	*RP11-74M13.4*	57242410	rs10747774	0.9128	57307079	T	C	0.36	1.16E-02	2.38E-02	1.87E-01
rs4526799	Brain_Caudate_basal_ganglia	12	*PA2G4*	56502897	rs10783812	0.9085	57308723	C	T	0.35	1.19E-02	3.12E-02	2.01E-01
rs4526799	Brain_Caudate_basal_ganglia	12	*IL23A*	56733428	rs10783812	0.9085	57308723	C	T	0.35	1.19E-02	2.96E-02	2.00E-01
rs4526799	Brain_Caudate_basal_ganglia	12	*RP11-74M13.4*	57242410	rs10783812	0.9085	57308723	C	T	0.35	1.19E-02	2.55E-02	1.96E-01

rs4526799	Brain_Caudate_basal_ganglia	12	*PA2G4*	56502897	rs11172056	0.9089	57308975	C	T	0.37	1.10E-02	2.41E-02	1.82E-01
rs4526799	Brain_Caudate_basal_ganglia	12	*COQ10A*	56662696	rs11172056	0.9089	57308975	C	T	0.37	1.10E-02	2.06E-02	1.78E-01
rs4526799	Brain_Caudate_basal_ganglia	12	*IL23A*	56733428	rs11172056	0.9089	57308975	C	T	0.37	1.10E-02	4.89E-02	2.05E-01
rs4526799	Brain_Caudate_basal_ganglia	12	*HSD17B6*	57163759	rs11172056	0.9089	57308975	C	T	0.37	1.10E-02	4.21E-02	1.99E-01
rs4526799	Brain_Caudate_basal_ganglia	12	*RP11-74M13.4*	57242410	rs11172056	0.9089	57308975	C	T	0.37	1.10E-02	2.38E-02	1.82E-01
rs4526799	Brain_Caudate_basal_ganglia	12	*PA2G4*	56502897	rs7302420	0.9085	57309884	G	C	0.36	1.54E-02	5.98E-03	1.86E-01
rs4526799	Brain_Caudate_basal_ganglia	12	*IL23A*	56733428	rs7302420	0.9085	57309884	G	C	0.36	1.54E-02	4.78E-03	1.84E-01
rs4526799	Brain_Caudate_basal_ganglia	12	*HSD17B6*	57163759	rs7302420	0.9085	57309884	G	C	0.36	1.54E-02	3.10E-02	2.17E-01
rs4526799	Brain_Caudate_basal_ganglia	12	*RP11-74M13.4*	57242410	rs7302420	0.9085	57309884	G	C	0.36	1.54E-02	3.08E-02	2.17E-01
rs4526799	Brain_Caudate_basal_ganglia	12	*MARS*	57889799	rs7302420	0.9085	57309884	G	C	0.36	1.54E-02	4.92E-02	2.32E-01
rs4526799	Brain_Caudate_basal_ganglia	12	*TSPAN31*	58138756	rs7302420	0.9085	57309884	G	C	0.36	1.54E-02	2.26E-02	2.09E-01
rs4526799	Brain_Caudate_basal_ganglia	12	*PA2G4*	56502897	rs12228618	0.9128	57311229	T	C	0.37	1.29E-02	8.25E-03	1.70E-01
rs4526799	Brain_Caudate_basal_ganglia	12	*COQ10A*	56662696	rs12228618	0.9128	57311229	T	C	0.37	1.29E-02	8.77E-03	1.71E-01
rs4526799	Brain_Caudate_basal_ganglia	12	*IL23A*	56733428	rs12228618	0.9128	57311229	T	C	0.37	1.29E-02	3.26E-02	1.99E-01
rs4526799	Brain_Caudate_basal_ganglia	12	*RP11-74M13.4*	57242410	rs12228618	0.9128	57311229	T	C	0.37	1.29E-02	2.65E-02	1.93E-01
rs4526799	Brain_Caudate_basal_ganglia	12	*PA2G4*	56502897	rs9739473	0.9012	57313335	A	T	0.37	2.65E-02	6.66E-03	2.40E-01
rs4526799	Brain_Caudate_basal_ganglia	12	*COQ10A*	56662696	rs9739473	0.9012	57313335	A	T	0.37	2.65E-02	9.94E-03	2.45E-01
rs4526799	Brain_Caudate_basal_ganglia	12	*IL23A*	56733428	rs9739473	0.9012	57313335	A	T	0.37	2.65E-02	4.29E-02	2.73E-01
rs4526799	Brain_Caudate_basal_ganglia	12	*HSD17B6*	57163759	rs9739473	0.9012	57313335	A	T	0.37	2.65E-02	4.14E-02	2.72E-01
rs4526799	Brain_Caudate_basal_ganglia	12	*RP11-74M13.4*	57242410	rs9739473	0.9012	57313335	A	T	0.37	2.65E-02	2.15E-02	2.57E-01
rs4526799	Brain_Cerebellar_Hemisphere	12	*RPS26*	56436876	rs9919772	0.8450	57260027	T	C	0.33	1.03E-02	4.72E-02	2.34E-01
rs4526799	Brain_Cerebellar_Hemisphere	12	*SLC39A5*	56627711	rs9919772	0.8450	57260027	T	C	0.33	1.03E-02	4.86E-02	2.35E-01
rs4526799	Brain_Cerebellar_Hemisphere	12	*ATP5B*	57035878	rs9919772	0.8450	57260027	T	C	0.33	1.03E-02	1.54E-02	2.04E-01
rs4526799	Brain_Cerebellar_Hemisphere	12	*RP11-603J24.7*	56374517	rs4495925	0.8650	57268116	C	G	0.33	1.04E-02	4.54E-02	2.29E-01
rs4526799	Brain_Cerebellar_Hemisphere	12	*RPS26*	56436876	rs4495925	0.8650	57268116	C	G	0.33	1.04E-02	2.94E-02	2.16E-01
rs4526799	Brain_Cerebellar_Hemisphere	12	*ATP5B*	57035878	rs4495925	0.8650	57268116	C	G	0.33	1.04E-02	1.73E-02	2.04E-01
rs4526799	Brain_Cerebellar_Hemisphere	12	*CYP27B1*	58158578	rs4495925	0.8650	57268116	C	G	0.33	1.04E-02	4.88E-02	2.32E-01
rs4526799	Brain_Cerebellar_Hemisphere	12	*RP11-603J24.7*	56374517	rs4471472	0.8650	57268985	A	G	0.32	9.99E-03	4.34E-02	2.24E-01
rs4526799	Brain_Cerebellar_Hemisphere	12	*RPS26*	56436876	rs4471472	0.8650	57268985	A	G	0.32	9.99E-03	3.33E-02	2.16E-01
rs4526799	Brain_Cerebellar_Hemisphere	12	*ATP5B*	57035878	rs4471472	0.8650	57268985	A	G	0.32	9.99E-03	1.70E-02	1.99E-01
rs4526799	Brain_Cerebellar_Hemisphere	12	*CYP27B1*	58158578	rs4471472	0.8650	57268985	A	G	0.32	9.99E-03	3.85E-02	2.20E-01
rs4526799	Brain_Cerebellar_Hemisphere	12	*RP11-603J24.7*	56374517	rs4633499	0.8610	57269264	A	T	0.32	1.43E-02	4.84E-02	2.61E-01
rs4526799	Brain_Cerebellar_Hemisphere	12	*RPS26*	56436876	rs4633499	0.8610	57269264	A	T	0.32	1.43E-02	2.01E-02	2.38E-01
rs4526799	Brain_Cerebellar_Hemisphere	12	*ATP5B*	57035878	rs4633499	0.8610	57269264	A	T	0.32	1.43E-02	1.10E-02	2.28E-01
rs4526799	Brain_Cerebellar_Hemisphere	12	*PMEL*	56357495	rs12300079	0.9956	57273194	T	C	0.36	3.73E-03	9.90E-03	1.38E-01
rs4526799	Brain_Cerebellar_Hemisphere	12	*RP11-603J24.7*	56374517	rs12300079	0.9956	57273194	T	C	0.36	3.73E-03	9.33E-03	1.37E-01
rs4526799	Brain_Cerebellar_Hemisphere	12	*MYL6*	56554355	rs12300079	0.9956	57273194	T	C	0.36	3.73E-03	1.40E-02	1.45E-01
rs4526799	Brain_Cerebellar_Hemisphere	12	*METTL21B*	58170856	rs12300079	0.9956	57273194	T	C	0.36	3.73E-03	4.24E-02	1.77E-01
rs4526799	Brain_Cerebellar_Hemisphere	12	*PMEL*	56357495	rs12300191	0.9956	57273289	A	G	0.36	3.59E-03	9.90E-03	1.36E-01
rs4526799	Brain_Cerebellar_Hemisphere	12	*RP11-603J24.7*	56374517	rs12300191	0.9956	57273289	A	G	0.36	3.59E-03	9.33E-03	1.35E-01

rs4526799	Brain_Cerebellar_Hemisphere	12	*MYL6*	56554355	rs12300191	0.9956	57273289	A	G	0.36	3.59E-03	1.40E-02	1.43E-01
rs4526799	Brain_Cerebellar_Hemisphere	12	*METTL21B*	58170856	rs12300191	0.9956	57273289	A	G	0.36	3.59E-03	4.24E-02	1.75E-01
rs4526799	Brain_Cerebellar_Hemisphere	12	*PMEL*	56357495	rs4514464	0.9956	57276375	C	T	0.36	2.92E-03	9.90E-03	1.33E-01
rs4526799	Brain_Cerebellar_Hemisphere	12	*RP11-603J24.7*	56374517	rs4514464	0.9956	57276375	C	T	0.36	2.92E-03	9.33E-03	1.32E-01
rs4526799	Brain_Cerebellar_Hemisphere	12	*MYL6*	56554355	rs4514464	0.9956	57276375	C	T	0.36	2.92E-03	1.40E-02	1.39E-01
rs4526799	Brain_Cerebellar_Hemisphere	12	*METTL21B*	58170856	rs4514464	0.9956	57276375	C	T	0.36	2.92E-03	4.24E-02	1.72E-01
rs4526799	Brain_Cerebellar_Hemisphere	12	*PMEL*	56357495	rs4417325	0.9956	57277302	G	A	0.36	3.59E-03	9.90E-03	1.36E-01
rs4526799	Brain_Cerebellar_Hemisphere	12	*RP11-603J24.7*	56374517	rs4417325	0.9956	57277302	G	A	0.36	3.59E-03	9.33E-03	1.35E-01
rs4526799	Brain_Cerebellar_Hemisphere	12	*MYL6*	56554355	rs4417325	0.9956	57277302	G	A	0.36	3.59E-03	1.40E-02	1.43E-01
rs4526799	Brain_Cerebellar_Hemisphere	12	*METTL21B*	58170856	rs4417325	0.9956	57277302	G	A	0.36	3.59E-03	4.24E-02	1.75E-01
rs4526799	Brain_Cerebellar_Hemisphere	12	*PMEL*	56357495	rs11172030	0.9956	57278076	A	C	0.36	3.63E-03	9.90E-03	1.37E-01
rs4526799	Brain_Cerebellar_Hemisphere	12	*RP11-603J24.7*	56374517	rs11172030	0.9956	57278076	A	C	0.36	3.63E-03	9.33E-03	1.36E-01
rs4526799	Brain_Cerebellar_Hemisphere	12	*MYL6*	56554355	rs11172030	0.9956	57278076	A	C	0.36	3.63E-03	1.40E-02	1.43E-01
rs4526799	Brain_Cerebellar_Hemisphere	12	*METTL21B*	58170856	rs11172030	0.9956	57278076	A	C	0.36	3.63E-03	4.24E-02	1.75E-01
rs4526799	Brain_Cerebellar_Hemisphere	12	*PMEL*	56357495	rs10876944	0.9956	57279372	T	A	0.36	3.63E-03	9.90E-03	1.37E-01
rs4526799	Brain_Cerebellar_Hemisphere	12	*RP11-603J24.7*	56374517	rs10876944	0.9956	57279372	T	A	0.36	3.63E-03	9.33E-03	1.36E-01
rs4526799	Brain_Cerebellar_Hemisphere	12	*MYL6*	56554355	rs10876944	0.9956	57279372	T	A	0.36	3.63E-03	1.40E-02	1.43E-01
rs4526799	Brain_Cerebellar_Hemisphere	12	*METTL21B*	58170856	rs10876944	0.9956	57279372	T	A	0.36	3.63E-03	4.24E-02	1.75E-01
rs4526799	Brain_Cerebellar_Hemisphere	12	*PMEL*	56357495	rs4326839	0.9956	57280374	G	C	0.36	3.58E-03	9.90E-03	1.36E-01
rs4526799	Brain_Cerebellar_Hemisphere	12	*RP11-603J24.7*	56374517	rs4326839	0.9956	57280374	G	C	0.36	3.58E-03	9.33E-03	1.35E-01
rs4526799	Brain_Cerebellar_Hemisphere	12	*MYL6*	56554355	rs4326839	0.9956	57280374	G	C	0.36	3.58E-03	1.40E-02	1.42E-01
rs4526799	Brain_Cerebellar_Hemisphere	12	*METTL21B*	58170856	rs4326839	0.9956	57280374	G	C	0.36	3.58E-03	4.24E-02	1.74E-01
rs4526799	Brain_Cerebellar_Hemisphere	12	*PMEL*	56357495	rs4526799	1.0000	57280586	T	C	0.34	7.26E-06	9.90E-03	3.69E-02
rs4526799	Brain_Cerebellar_Hemisphere	12	*RP11-603J24.7*	56374517	rs4526799	1.0000	57280586	T	C	0.34	7.26E-06	9.33E-03	3.59E-02
rs4526799	Brain_Cerebellar_Hemisphere	12	*MYL6*	56554355	rs4526799	1.0000	57280586	T	C	0.34	7.26E-06	1.40E-02	4.33E-02
rs4526799	Brain_Cerebellar_Hemisphere	12	*METTL21B*	58170856	rs4526799	1.0000	57280586	T	C	0.34	7.26E-06	4.24E-02	7.81E-02
rs4526799	Brain_Cerebellar_Hemisphere	12	*PMEL*	56357495	rs28876529	0.9956	57285301	T	A	0.36	2.25E-03	9.90E-03	1.20E-01
rs4526799	Brain_Cerebellar_Hemisphere	12	*RP11-603J24.7*	56374517	rs28876529	0.9956	57285301	T	A	0.36	2.25E-03	9.33E-03	1.19E-01
rs4526799	Brain_Cerebellar_Hemisphere	12	*MYL6*	56554355	rs28876529	0.9956	57285301	T	A	0.36	2.25E-03	1.40E-02	1.27E-01
rs4526799	Brain_Cerebellar_Hemisphere	12	*METTL21B*	58170856	rs28876529	0.9956	57285301	T	A	0.36	2.25E-03	4.24E-02	1.60E-01
rs4526799	Brain_Cerebellar_Hemisphere	12	*PMEL*	56357495	rs11172037	0.9956	57285427	T	A	0.36	2.25E-03	9.90E-03	1.20E-01
rs4526799	Brain_Cerebellar_Hemisphere	12	*RP11-603J24.7*	56374517	rs11172037	0.9956	57285427	T	A	0.36	2.25E-03	9.33E-03	1.19E-01
rs4526799	Brain_Cerebellar_Hemisphere	12	*MYL6*	56554355	rs11172037	0.9956	57285427	T	A	0.36	2.25E-03	1.40E-02	1.27E-01
rs4526799	Brain_Cerebellar_Hemisphere	12	*METTL21B*	58170856	rs11172037	0.9956	57285427	T	A	0.36	2.25E-03	4.24E-02	1.60E-01
rs4526799	Brain_Cerebellar_Hemisphere	12	*PMEL*	56357495	rs12321987	0.9956	57288449	G	A	0.36	4.47E-03	9.90E-03	1.59E-01
rs4526799	Brain_Cerebellar_Hemisphere	12	*RP11-603J24.7*	56374517	rs12321987	0.9956	57288449	G	A	0.36	4.47E-03	9.33E-03	1.58E-01
rs4526799	Brain_Cerebellar_Hemisphere	12	*MYL6*	56554355	rs12321987	0.9956	57288449	G	A	0.36	4.47E-03	1.40E-02	1.65E-01
rs4526799	Brain_Cerebellar_Hemisphere	12	*METTL21B*	58170856	rs12321987	0.9956	57288449	G	A	0.36	4.47E-03	4.24E-02	1.95E-01
rs4526799	Brain_Cerebellar_Hemisphere	12	*PMEL*	56357495	rs11172043	0.9869	57293182	G	A	0.35	4.58E-03	4.29E-02	1.94E-01
rs4526799	Brain_Cerebellar_Hemisphere	12	*RP11-603J24.7*	56374517	rs11172043	0.9869	57293182	G	A	0.35	4.58E-03	5.63E-03	1.49E-01

rs4526799	Brain_Cerebellar_Hemisphere	12	*MYL6*	56554355	rs11172043	0.9869	57293182	G	A	0.35	4.58E-03	1.93E-02	1.70E-01
rs4526799	Brain_Cerebellar_Hemisphere	12	*ATP5B*	57035878	rs11172043	0.9869	57293182	G	A	0.35	4.58E-03	3.70E-02	1.89E-01
rs4526799	Brain_Cerebellar_Hemisphere	12	*PMEL*	56357495	rs12426816	0.9869	57294074	A	C	0.35	4.50E-03	4.29E-02	1.94E-01
rs4526799	Brain_Cerebellar_Hemisphere	12	*RP11-603J24.7*	56374517	rs12426816	0.9869	57294074	A	C	0.35	4.50E-03	5.63E-03	1.48E-01
rs4526799	Brain_Cerebellar_Hemisphere	12	*MYL6*	56554355	rs12426816	0.9869	57294074	A	C	0.35	4.50E-03	1.93E-02	1.70E-01
rs4526799	Brain_Cerebellar_Hemisphere	12	*ATP5B*	57035878	rs12426816	0.9869	57294074	A	C	0.35	4.50E-03	3.70E-02	1.88E-01
rs4526799	Brain_Cerebellar_Hemisphere	12	*PMEL*	56357495	rs11172047	0.9869	57298080	T	C	0.35	6.51E-03	4.29E-02	2.07E-01
rs4526799	Brain_Cerebellar_Hemisphere	12	*RP11-603J24.7*	56374517	rs11172047	0.9869	57298080	T	C	0.35	6.51E-03	5.63E-03	1.64E-01
rs4526799	Brain_Cerebellar_Hemisphere	12	*MYL6*	56554355	rs11172047	0.9869	57298080	T	C	0.35	6.51E-03	1.93E-02	1.84E-01
rs4526799	Brain_Cerebellar_Hemisphere	12	*ATP5B*	57035878	rs11172047	0.9869	57298080	T	C	0.35	6.51E-03	3.70E-02	2.02E-01
rs4526799	Brain_Cerebellar_Hemisphere	12	*PMEL*	56357495	rs2371631	0.9869	57298614	T	A	0.35	6.32E-03	4.29E-02	2.04E-01
rs4526799	Brain_Cerebellar_Hemisphere	12	*RP11-603J24.7*	56374517	rs2371631	0.9869	57298614	T	A	0.35	6.32E-03	5.63E-03	1.60E-01
rs4526799	Brain_Cerebellar_Hemisphere	12	*MYL6*	56554355	rs2371631	0.9869	57298614	T	A	0.35	6.32E-03	1.93E-02	1.81E-01
rs4526799	Brain_Cerebellar_Hemisphere	12	*ATP5B*	57035878	rs2371631	0.9869	57298614	T	A	0.35	6.32E-03	3.70E-02	1.99E-01
rs4526799	Brain_Cerebellar_Hemisphere	12	*PMEL*	56357495	rs12305763	0.9869	57299263	G	A	0.35	6.82E-03	4.29E-02	2.09E-01
rs4526799	Brain_Cerebellar_Hemisphere	12	*RP11-603J24.7*	56374517	rs12305763	0.9869	57299263	G	A	0.35	6.82E-03	5.63E-03	1.65E-01
rs4526799	Brain_Cerebellar_Hemisphere	12	*MYL6*	56554355	rs12305763	0.9869	57299263	G	A	0.35	6.82E-03	1.93E-02	1.86E-01
rs4526799	Brain_Cerebellar_Hemisphere	12	*ATP5B*	57035878	rs12305763	0.9869	57299263	G	A	0.35	6.82E-03	3.70E-02	2.03E-01
rs4526799	Brain_Cerebellar_Hemisphere	12	*RP11-603J24.7*	56374517	rs11172049	0.9128	57304203	T	C	0.35	1.15E-02	1.61E-02	1.82E-01
rs4526799	Brain_Cerebellar_Hemisphere	12	*RP11-603J24.5*	56518610	rs11172049	0.9128	57304203	T	C	0.35	1.15E-02	1.66E-02	1.83E-01
rs4526799	Brain_Cerebellar_Hemisphere	12	*ATP5B*	57035878	rs11172049	0.9128	57304203	T	C	0.35	1.15E-02	3.72E-02	2.04E-01
rs4526799	Brain_Cerebellar_Hemisphere	12	*RP11-603J24.7*	56374517	rs1874888	0.9085	57305138	A	C	0.35	1.15E-02	1.61E-02	1.82E-01
rs4526799	Brain_Cerebellar_Hemisphere	12	*RP11-603J24.5*	56518610	rs1874888	0.9085	57305138	A	C	0.35	1.15E-02	1.66E-02	1.83E-01
rs4526799	Brain_Cerebellar_Hemisphere	12	*ATP5B*	57035878	rs1874888	0.9085	57305138	A	C	0.35	1.15E-02	3.72E-02	2.04E-01
rs4526799	Brain_Cerebellar_Hemisphere	12	*RP11-603J24.7*	56374517	rs10506349	0.9128	57306412	T	C	0.37	1.10E-02	1.70E-02	1.74E-01
rs4526799	Brain_Cerebellar_Hemisphere	12	*RP11-603J24.5*	56518610	rs10506349	0.9128	57306412	T	C	0.37	1.10E-02	1.58E-02	1.72E-01
rs4526799	Brain_Cerebellar_Hemisphere	12	*ATP5B*	57035878	rs10506349	0.9128	57306412	T	C	0.37	1.10E-02	4.83E-02	2.05E-01
rs4526799	Brain_Cerebellar_Hemisphere	12	*METTL21B*	58170856	rs10506349	0.9128	57306412	T	C	0.37	1.10E-02	4.90E-02	2.05E-01
rs4526799	Brain_Cerebellar_Hemisphere	12	*RP11-603J24.7*	56374517	rs10876951	0.9085	57306430	T	G	0.35	1.15E-02	1.61E-02	1.82E-01
rs4526799	Brain_Cerebellar_Hemisphere	12	*RP11-603J24.5*	56518610	rs10876951	0.9085	57306430	T	G	0.35	1.15E-02	1.66E-02	1.83E-01
rs4526799	Brain_Cerebellar_Hemisphere	12	*ATP5B*	57035878	rs10876951	0.9085	57306430	T	G	0.35	1.15E-02	3.72E-02	2.04E-01
rs4526799	Brain_Cerebellar_Hemisphere	12	*RP11-603J24.7*	56374517	rs10747774	0.9128	57307079	T	C	0.36	1.16E-02	1.70E-02	1.79E-01
rs4526799	Brain_Cerebellar_Hemisphere	12	*RP11-603J24.5*	56518610	rs10747774	0.9128	57307079	T	C	0.36	1.16E-02	1.58E-02	1.78E-01
rs4526799	Brain_Cerebellar_Hemisphere	12	*ATP5B*	57035878	rs10747774	0.9128	57307079	T	C	0.36	1.16E-02	4.83E-02	2.10E-01
rs4526799	Brain_Cerebellar_Hemisphere	12	*METTL21B*	58170856	rs10747774	0.9128	57307079	T	C	0.36	1.16E-02	4.90E-02	2.10E-01
rs4526799	Brain_Cerebellar_Hemisphere	12	*RP11-603J24.7*	56374517	rs10783812	0.9085	57308723	C	T	0.35	1.19E-02	1.61E-02	1.85E-01
rs4526799	Brain_Cerebellar_Hemisphere	12	*RP11-603J24.5*	56518610	rs10783812	0.9085	57308723	C	T	0.35	1.19E-02	1.66E-02	1.86E-01
rs4526799	Brain_Cerebellar_Hemisphere	12	*ATP5B*	57035878	rs10783812	0.9085	57308723	C	T	0.35	1.19E-02	3.72E-02	2.07E-01
rs4526799	Brain_Cerebellar_Hemisphere	12	*RP11-603J24.7*	56374517	rs11172056	0.9089	57308975	C	T	0.37	1.10E-02	1.70E-02	1.74E-01
rs4526799	Brain_Cerebellar_Hemisphere	12	*RP11-603J24.5*	56518610	rs11172056	0.9089	57308975	C	T	0.37	1.10E-02	1.58E-02	1.72E-01

rs4526799	Brain_Cerebellar_Hemisphere	12	*ATP5B*	57035878	rs11172056	0.9089	57308975	C	T	0.37	1.10E-02	4.83E-02	2.05E-01
rs4526799	Brain_Cerebellar_Hemisphere	12	*METTL21B*	58170856	rs11172056	0.9089	57308975	C	T	0.37	1.10E-02	4.90E-02	2.05E-01
rs4526799	Brain_Cerebellar_Hemisphere	12	*PMEL*	56357495	rs7302420	0.9085	57309884	G	C	0.36	1.54E-02	4.44E-02	2.28E-01
rs4526799	Brain_Cerebellar_Hemisphere	12	*RP11-603J24.7*	56374517	rs7302420	0.9085	57309884	G	C	0.36	1.54E-02	2.46E-02	2.11E-01
rs4526799	Brain_Cerebellar_Hemisphere	12	*RP11-603J24.5*	56518610	rs7302420	0.9085	57309884	G	C	0.36	1.54E-02	4.29E-02	2.27E-01
rs4526799	Brain_Cerebellar_Hemisphere	12	*ATP5B*	57035878	rs7302420	0.9085	57309884	G	C	0.36	1.54E-02	4.66E-02	2.30E-01
rs4526799	Brain_Cerebellar_Hemisphere	12	*RP11-603J24.7*	56374517	rs12228618	0.9128	57311229	T	C	0.37	1.29E-02	2.09E-02	1.87E-01
rs4526799	Brain_Cerebellar_Hemisphere	12	*ATP5B*	57035878	rs12228618	0.9128	57311229	T	C	0.37	1.29E-02	3.71E-02	2.03E-01
rs4526799	Brain_Cerebellar_Hemisphere	12	*METTL21B*	58170856	rs12228618	0.9128	57311229	T	C	0.37	1.29E-02	4.46E-02	2.10E-01
rs4526799	Brain_Cerebellar_Hemisphere	12	*RP11-603J24.7*	56374517	rs9739473	0.9012	57313335	A	T	0.37	2.65E-02	3.13E-02	2.65E-01
rs4526799	Brain_Cerebellar_Hemisphere	12	*RP11-603J24.5*	56518610	rs9739473	0.9012	57313335	A	T	0.37	2.65E-02	4.90E-02	2.77E-01
rs4526799	Brain_Cerebellar_Hemisphere	12	*ATP5B*	57035878	rs9739473	0.9012	57313335	A	T	0.37	2.65E-02	4.46E-02	2.74E-01
rs4526799	Brain_Cerebellar_Hemisphere	12	*METTL21B*	58170856	rs9739473	0.9012	57313335	A	T	0.37	2.65E-02	2.92E-02	2.63E-01
rs4526799	Brain_Cerebellum	12	*RPS26*	56436876	rs9919772	0.8450	57260027	T	C	0.33	1.03E-02	1.84E-02	2.08E-01
rs4526799	Brain_Cerebellum	12	*MYL6*	56554355	rs9919772	0.8450	57260027	T	C	0.33	1.03E-02	3.55E-02	2.24E-01
rs4526799	Brain_Cerebellum	12	*RP11-977G19.5*	56570105	rs9919772	0.8450	57260027	T	C	0.33	1.03E-02	1.67E-02	2.06E-01
rs4526799	Brain_Cerebellum	12	*CS*	56679829	rs9919772	0.8450	57260027	T	C	0.33	1.03E-02	2.72E-02	2.17E-01
rs4526799	Brain_Cerebellum	12	*TIMELESS*	56827045	rs9919772	0.8450	57260027	T	C	0.33	1.03E-02	4.55E-02	2.32E-01
rs4526799	Brain_Cerebellum	12	*PTGES3*	57069643	rs9919772	0.8450	57260027	T	C	0.33	1.03E-02	1.35E-02	2.02E-01
rs4526799	Brain_Cerebellum	12	*PRIM1*	57135785	rs9919772	0.8450	57260027	T	C	0.33	1.03E-02	3.17E-02	2.21E-01
rs4526799	Brain_Cerebellum	12	*RDH16*	57349188	rs9919772	0.8450	57260027	T	C	0.33	1.03E-02	1.47E-02	2.04E-01
rs4526799	Brain_Cerebellum	12	*B4GALNT1*	58023536	rs9919772	0.8450	57260027	T	C	0.33	1.03E-02	2.65E-02	2.16E-01
rs4526799	Brain_Cerebellum	12	*CYP27B1*	58158578	rs9919772	0.8450	57260027	T	C	0.33	1.03E-02	2.05E-02	2.10E-01
rs4526799	Brain_Cerebellum	12	*RPS26*	56436876	rs4495925	0.8650	57268116	C	G	0.33	1.04E-02	9.66E-03	1.94E-01
rs4526799	Brain_Cerebellum	12	*ESYT1*	56530147	rs4495925	0.8650	57268116	C	G	0.33	1.04E-02	2.99E-02	2.16E-01
rs4526799	Brain_Cerebellum	12	*RP11-977G19.5*	56570105	rs4495925	0.8650	57268116	C	G	0.33	1.04E-02	1.43E-02	2.00E-01
rs4526799	Brain_Cerebellum	12	*PTGES3*	57069643	rs4495925	0.8650	57268116	C	G	0.33	1.04E-02	1.73E-02	2.04E-01
rs4526799	Brain_Cerebellum	12	*RDH16*	57349188	rs4495925	0.8650	57268116	C	G	0.33	1.04E-02	4.84E-03	1.85E-01
rs4526799	Brain_Cerebellum	12	*B4GALNT1*	58023536	rs4495925	0.8650	57268116	C	G	0.33	1.04E-02	3.91E-02	2.24E-01
rs4526799	Brain_Cerebellum	12	*RPS26*	56436876	rs4471472	0.8650	57268985	A	G	0.32	9.99E-03	9.94E-03	1.90E-01
rs4526799	Brain_Cerebellum	12	*ESYT1*	56530147	rs4471472	0.8650	57268985	A	G	0.32	9.99E-03	3.43E-02	2.17E-01
rs4526799	Brain_Cerebellum	12	*RP11-977G19.5*	56570105	rs4471472	0.8650	57268985	A	G	0.32	9.99E-03	1.75E-02	2.00E-01
rs4526799	Brain_Cerebellum	12	*PTGES3*	57069643	rs4471472	0.8650	57268985	A	G	0.32	9.99E-03	1.76E-02	2.00E-01
rs4526799	Brain_Cerebellum	12	*RDH16*	57349188	rs4471472	0.8650	57268985	A	G	0.32	9.99E-03	4.10E-03	1.79E-01
rs4526799	Brain_Cerebellum	12	*B4GALNT1*	58023536	rs4471472	0.8650	57268985	A	G	0.32	9.99E-03	4.01E-02	2.22E-01
rs4526799	Brain_Cerebellum	12	*RPS26*	56436876	rs4633499	0.8610	57269264	A	T	0.32	1.43E-02	4.61E-03	2.18E-01
rs4526799	Brain_Cerebellum	12	*ESYT1*	56530147	rs4633499	0.8610	57269264	A	T	0.32	1.43E-02	1.08E-02	2.28E-01
rs4526799	Brain_Cerebellum	12	*RP11-977G19.5*	56570105	rs4633499	0.8610	57269264	A	T	0.32	1.43E-02	2.82E-02	2.46E-01
rs4526799	Brain_Cerebellum	12	*PTGES3*	57069643	rs4633499	0.8610	57269264	A	T	0.32	1.43E-02	3.62E-02	2.52E-01
rs4526799	Brain_Cerebellum	12	*RDH16*	57349188	rs4633499	0.8610	57269264	A	T	0.32	1.43E-02	3.58E-03	2.16E-01

rs4526799	Brain_Cerebellum	12	*B4GALNT1*	58023536	rs4633499	0.8610	57269264	A	T	0.32	1.43E-02	2.59E-02	2.44E-01
rs4526799	Brain_Cerebellum	12	*RPS26*	56436876	rs12300079	0.9956	57273194	T	C	0.36	3.73E-03	2.88E-02	1.63E-01
rs4526799	Brain_Cerebellum	12	*CS*	56679829	rs12300079	0.9956	57273194	T	C	0.36	3.73E-03	3.16E-02	1.66E-01
rs4526799	Brain_Cerebellum	12	*MIP*	56853054	rs12300079	0.9956	57273194	T	C	0.36	3.73E-03	4.42E-02	1.78E-01
rs4526799	Brain_Cerebellum	12	*PTGES3*	57069643	rs12300079	0.9956	57273194	T	C	0.36	3.73E-03	3.11E-02	1.66E-01
rs4526799	Brain_Cerebellum	12	*RDH16*	57349188	rs12300079	0.9956	57273194	T	C	0.36	3.73E-03	2.13E-02	1.55E-01
rs4526799	Brain_Cerebellum	12	*RPS26*	56436876	rs12300191	0.9956	57273289	A	G	0.36	3.59E-03	2.88E-02	1.61E-01
rs4526799	Brain_Cerebellum	12	*CS*	56679829	rs12300191	0.9956	57273289	A	G	0.36	3.59E-03	3.16E-02	1.64E-01
rs4526799	Brain_Cerebellum	12	*MIP*	56853054	rs12300191	0.9956	57273289	A	G	0.36	3.59E-03	4.42E-02	1.76E-01
rs4526799	Brain_Cerebellum	12	*PTGES3*	57069643	rs12300191	0.9956	57273289	A	G	0.36	3.59E-03	3.11E-02	1.63E-01
rs4526799	Brain_Cerebellum	12	*RDH16*	57349188	rs12300191	0.9956	57273289	A	G	0.36	3.59E-03	2.13E-02	1.52E-01
rs4526799	Brain_Cerebellum	12	*RPS26*	56436876	rs4514464	0.9956	57276375	C	T	0.36	2.92E-03	2.88E-02	1.58E-01
rs4526799	Brain_Cerebellum	12	*CS*	56679829	rs4514464	0.9956	57276375	C	T	0.36	2.92E-03	3.16E-02	1.61E-01
rs4526799	Brain_Cerebellum	12	*MIP*	56853054	rs4514464	0.9956	57276375	C	T	0.36	2.92E-03	4.42E-02	1.73E-01
rs4526799	Brain_Cerebellum	12	*PTGES3*	57069643	rs4514464	0.9956	57276375	C	T	0.36	2.92E-03	3.11E-02	1.60E-01
rs4526799	Brain_Cerebellum	12	*RDH16*	57349188	rs4514464	0.9956	57276375	C	T	0.36	2.92E-03	2.13E-02	1.49E-01
rs4526799	Brain_Cerebellum	12	*RPS26*	56436876	rs4417325	0.9956	57277302	G	A	0.36	3.59E-03	2.88E-02	1.61E-01
rs4526799	Brain_Cerebellum	12	*CS*	56679829	rs4417325	0.9956	57277302	G	A	0.36	3.59E-03	3.16E-02	1.64E-01
rs4526799	Brain_Cerebellum	12	*MIP*	56853054	rs4417325	0.9956	57277302	G	A	0.36	3.59E-03	4.42E-02	1.76E-01
rs4526799	Brain_Cerebellum	12	*PTGES3*	57069643	rs4417325	0.9956	57277302	G	A	0.36	3.59E-03	3.11E-02	1.63E-01
rs4526799	Brain_Cerebellum	12	*RDH16*	57349188	rs4417325	0.9956	57277302	G	A	0.36	3.59E-03	2.13E-02	1.52E-01
rs4526799	Brain_Cerebellum	12	*RPS26*	56436876	rs11172030	0.9956	57278076	A	C	0.36	3.63E-03	2.88E-02	1.61E-01
rs4526799	Brain_Cerebellum	12	*CS*	56679829	rs11172030	0.9956	57278076	A	C	0.36	3.63E-03	3.16E-02	1.64E-01
rs4526799	Brain_Cerebellum	12	*MIP*	56853054	rs11172030	0.9956	57278076	A	C	0.36	3.63E-03	4.42E-02	1.77E-01
rs4526799	Brain_Cerebellum	12	*PTGES3*	57069643	rs11172030	0.9956	57278076	A	C	0.36	3.63E-03	3.11E-02	1.64E-01
rs4526799	Brain_Cerebellum	12	*RDH16*	57349188	rs11172030	0.9956	57278076	A	C	0.36	3.63E-03	2.13E-02	1.53E-01
rs4526799	Brain_Cerebellum	12	*RPS26*	56436876	rs10876944	0.9956	57279372	T	A	0.36	3.63E-03	2.88E-02	1.61E-01
rs4526799	Brain_Cerebellum	12	*CS*	56679829	rs10876944	0.9956	57279372	T	A	0.36	3.63E-03	3.16E-02	1.64E-01
rs4526799	Brain_Cerebellum	12	*MIP*	56853054	rs10876944	0.9956	57279372	T	A	0.36	3.63E-03	4.42E-02	1.77E-01
rs4526799	Brain_Cerebellum	12	*PTGES3*	57069643	rs10876944	0.9956	57279372	T	A	0.36	3.63E-03	3.11E-02	1.64E-01
rs4526799	Brain_Cerebellum	12	*RDH16*	57349188	rs10876944	0.9956	57279372	T	A	0.36	3.63E-03	2.13E-02	1.53E-01
rs4526799	Brain_Cerebellum	12	*RPS26*	56436876	rs4326839	0.9956	57280374	G	C	0.36	3.58E-03	2.88E-02	1.61E-01
rs4526799	Brain_Cerebellum	12	*CS*	56679829	rs4326839	0.9956	57280374	G	C	0.36	3.58E-03	3.16E-02	1.64E-01
rs4526799	Brain_Cerebellum	12	*MIP*	56853054	rs4326839	0.9956	57280374	G	C	0.36	3.58E-03	4.42E-02	1.76E-01
rs4526799	Brain_Cerebellum	12	*PTGES3*	57069643	rs4326839	0.9956	57280374	G	C	0.36	3.58E-03	3.11E-02	1.63E-01
rs4526799	Brain_Cerebellum	12	*RDH16*	57349188	rs4326839	0.9956	57280374	G	C	0.36	3.58E-03	2.13E-02	1.52E-01
rs4526799	Brain_Cerebellum	12	*RPS26*	56436876	rs4526799	1.0000	57280586	T	C	0.34	7.26E-06	2.88E-02	6.27E-02
rs4526799	Brain_Cerebellum	12	*CS*	56679829	rs4526799	1.0000	57280586	T	C	0.34	7.26E-06	3.16E-02	6.59E-02
rs4526799	Brain_Cerebellum	12	*MIP*	56853054	rs4526799	1.0000	57280586	T	C	0.34	7.26E-06	4.42E-02	8.00E-02
rs4526799	Brain_Cerebellum	12	*PTGES3*	57069643	rs4526799	1.0000	57280586	T	C	0.34	7.26E-06	3.11E-02	6.53E-02

rs4526799	Brain_Cerebellum	12	*RDH16*	57349188	rs4526799	1.0000	57280586	T	C	0.34	7.26E-06	2.13E-02	5.34E-02
rs4526799	Brain_Cerebellum	12	*RPS26*	56436876	rs28876529	0.9956	57285301	T	A	0.36	2.25E-03	2.88E-02	1.46E-01
rs4526799	Brain_Cerebellum	12	*CS*	56679829	rs28876529	0.9956	57285301	T	A	0.36	2.25E-03	3.16E-02	1.49E-01
rs4526799	Brain_Cerebellum	12	*MIP*	56853054	rs28876529	0.9956	57285301	T	A	0.36	2.25E-03	4.42E-02	1.61E-01
rs4526799	Brain_Cerebellum	12	*PTGES3*	57069643	rs28876529	0.9956	57285301	T	A	0.36	2.25E-03	3.11E-02	1.48E-01
rs4526799	Brain_Cerebellum	12	*RDH16*	57349188	rs28876529	0.9956	57285301	T	A	0.36	2.25E-03	2.13E-02	1.37E-01
rs4526799	Brain_Cerebellum	12	*RPS26*	56436876	rs11172037	0.9956	57285427	T	A	0.36	2.25E-03	2.88E-02	1.46E-01
rs4526799	Brain_Cerebellum	12	*CS*	56679829	rs11172037	0.9956	57285427	T	A	0.36	2.25E-03	3.16E-02	1.49E-01
rs4526799	Brain_Cerebellum	12	*MIP*	56853054	rs11172037	0.9956	57285427	T	A	0.36	2.25E-03	4.42E-02	1.61E-01
rs4526799	Brain_Cerebellum	12	*PTGES3*	57069643	rs11172037	0.9956	57285427	T	A	0.36	2.25E-03	3.11E-02	1.48E-01
rs4526799	Brain_Cerebellum	12	*RDH16*	57349188	rs11172037	0.9956	57285427	T	A	0.36	2.25E-03	2.13E-02	1.37E-01
rs4526799	Brain_Cerebellum	12	*RPS26*	56436876	rs12321987	0.9956	57288449	G	A	0.36	4.47E-03	2.88E-02	1.82E-01
rs4526799	Brain_Cerebellum	12	*CS*	56679829	rs12321987	0.9956	57288449	G	A	0.36	4.47E-03	3.16E-02	1.85E-01
rs4526799	Brain_Cerebellum	12	*MIP*	56853054	rs12321987	0.9956	57288449	G	A	0.36	4.47E-03	4.42E-02	1.97E-01
rs4526799	Brain_Cerebellum	12	*PTGES3*	57069643	rs12321987	0.9956	57288449	G	A	0.36	4.47E-03	3.11E-02	1.84E-01
rs4526799	Brain_Cerebellum	12	*RDH16*	57349188	rs12321987	0.9956	57288449	G	A	0.36	4.47E-03	2.13E-02	1.74E-01
rs4526799	Brain_Cerebellum	12	*RPS26*	56436876	rs11172043	0.9869	57293182	G	A	0.35	4.58E-03	1.41E-02	1.64E-01
rs4526799	Brain_Cerebellum	12	*ESYT1*	56530147	rs11172043	0.9869	57293182	G	A	0.35	4.58E-03	1.32E-02	1.62E-01
rs4526799	Brain_Cerebellum	12	*CS*	56679829	rs11172043	0.9869	57293182	G	A	0.35	4.58E-03	3.73E-02	1.89E-01
rs4526799	Brain_Cerebellum	12	*MIP*	56853054	rs11172043	0.9869	57293182	G	A	0.35	4.58E-03	4.41E-02	1.95E-01
rs4526799	Brain_Cerebellum	12	*RDH16*	57349188	rs11172043	0.9869	57293182	G	A	0.35	4.58E-03	1.61E-02	1.66E-01
rs4526799	Brain_Cerebellum	12	*MBD6*	57919188	rs11172043	0.9869	57293182	G	A	0.35	4.58E-03	4.99E-02	2.00E-01
rs4526799	Brain_Cerebellum	12	*KIF5A*	57962098	rs11172043	0.9869	57293182	G	A	0.35	4.58E-03	4.21E-02	1.93E-01
rs4526799	Brain_Cerebellum	12	*B4GALNT1*	58023536	rs11172043	0.9869	57293182	G	A	0.35	4.58E-03	4.63E-02	1.97E-01
rs4526799	Brain_Cerebellum	12	*RPS26*	56436876	rs12426816	0.9869	57294074	A	C	0.35	4.50E-03	1.41E-02	1.63E-01
rs4526799	Brain_Cerebellum	12	*ESYT1*	56530147	rs12426816	0.9869	57294074	A	C	0.35	4.50E-03	1.32E-02	1.62E-01
rs4526799	Brain_Cerebellum	12	*CS*	56679829	rs12426816	0.9869	57294074	A	C	0.35	4.50E-03	3.73E-02	1.89E-01
rs4526799	Brain_Cerebellum	12	*MIP*	56853054	rs12426816	0.9869	57294074	A	C	0.35	4.50E-03	4.41E-02	1.95E-01
rs4526799	Brain_Cerebellum	12	*RDH16*	57349188	rs12426816	0.9869	57294074	A	C	0.35	4.50E-03	1.61E-02	1.66E-01
rs4526799	Brain_Cerebellum	12	*MBD6*	57919188	rs12426816	0.9869	57294074	A	C	0.35	4.50E-03	4.99E-02	2.00E-01
rs4526799	Brain_Cerebellum	12	*KIF5A*	57962098	rs12426816	0.9869	57294074	A	C	0.35	4.50E-03	4.21E-02	1.93E-01
rs4526799	Brain_Cerebellum	12	*B4GALNT1*	58023536	rs12426816	0.9869	57294074	A	C	0.35	4.50E-03	4.63E-02	1.97E-01
rs4526799	Brain_Cerebellum	12	*RPS26*	56436876	rs11172047	0.9869	57298080	T	C	0.35	6.51E-03	1.41E-02	1.78E-01
rs4526799	Brain_Cerebellum	12	*ESYT1*	56530147	rs11172047	0.9869	57298080	T	C	0.35	6.51E-03	1.32E-02	1.77E-01
rs4526799	Brain_Cerebellum	12	*CS*	56679829	rs11172047	0.9869	57298080	T	C	0.35	6.51E-03	3.73E-02	2.02E-01
rs4526799	Brain_Cerebellum	12	*MIP*	56853054	rs11172047	0.9869	57298080	T	C	0.35	6.51E-03	4.41E-02	2.08E-01
rs4526799	Brain_Cerebellum	12	*RDH16*	57349188	rs11172047	0.9869	57298080	T	C	0.35	6.51E-03	1.61E-02	1.81E-01
rs4526799	Brain_Cerebellum	12	*MBD6*	57919188	rs11172047	0.9869	57298080	T	C	0.35	6.51E-03	4.99E-02	2.13E-01
rs4526799	Brain_Cerebellum	12	*KIF5A*	57962098	rs11172047	0.9869	57298080	T	C	0.35	6.51E-03	4.21E-02	2.07E-01
rs4526799	Brain_Cerebellum	12	*B4GALNT1*	58023536	rs11172047	0.9869	57298080	T	C	0.35	6.51E-03	4.63E-02	2.10E-01

rs4526799	Brain_Cerebellum	12	*RPS26*	56436876	rs2371631	0.9869	57298614	T	A	0.35	6.32E-03	1.41E-02	1.75E-01
rs4526799	Brain_Cerebellum	12	*ESYT1*	56530147	rs2371631	0.9869	57298614	T	A	0.35	6.32E-03	1.32E-02	1.73E-01
rs4526799	Brain_Cerebellum	12	*CS*	56679829	rs2371631	0.9869	57298614	T	A	0.35	6.32E-03	3.73E-02	1.99E-01
rs4526799	Brain_Cerebellum	12	*MIP*	56853054	rs2371631	0.9869	57298614	T	A	0.35	6.32E-03	4.41E-02	2.05E-01
rs4526799	Brain_Cerebellum	12	*RDH16*	57349188	rs2371631	0.9869	57298614	T	A	0.35	6.32E-03	1.61E-02	1.77E-01
rs4526799	Brain_Cerebellum	12	*MBD6*	57919188	rs2371631	0.9869	57298614	T	A	0.35	6.32E-03	4.99E-02	2.10E-01
rs4526799	Brain_Cerebellum	12	*KIF5A*	57962098	rs2371631	0.9869	57298614	T	A	0.35	6.32E-03	4.21E-02	2.04E-01
rs4526799	Brain_Cerebellum	12	*B4GALNT1*	58023536	rs2371631	0.9869	57298614	T	A	0.35	6.32E-03	4.63E-02	2.07E-01
rs4526799	Brain_Cerebellum	12	*RPS26*	56436876	rs12305763	0.9869	57299263	G	A	0.35	6.82E-03	1.41E-02	1.79E-01
rs4526799	Brain_Cerebellum	12	*ESYT1*	56530147	rs12305763	0.9869	57299263	G	A	0.35	6.82E-03	1.32E-02	1.78E-01
rs4526799	Brain_Cerebellum	12	*CS*	56679829	rs12305763	0.9869	57299263	G	A	0.35	6.82E-03	3.73E-02	2.04E-01
rs4526799	Brain_Cerebellum	12	*MIP*	56853054	rs12305763	0.9869	57299263	G	A	0.35	6.82E-03	4.41E-02	2.10E-01
rs4526799	Brain_Cerebellum	12	*RDH16*	57349188	rs12305763	0.9869	57299263	G	A	0.35	6.82E-03	1.61E-02	1.82E-01
rs4526799	Brain_Cerebellum	12	*MBD6*	57919188	rs12305763	0.9869	57299263	G	A	0.35	6.82E-03	4.99E-02	2.14E-01
rs4526799	Brain_Cerebellum	12	*KIF5A*	57962098	rs12305763	0.9869	57299263	G	A	0.35	6.82E-03	4.21E-02	2.08E-01
rs4526799	Brain_Cerebellum	12	*B4GALNT1*	58023536	rs12305763	0.9869	57299263	G	A	0.35	6.82E-03	4.63E-02	2.11E-01
rs4526799	Brain_Cerebellum	12	*CDK2*	56363560	rs11172049	0.9128	57304203	T	C	0.35	1.15E-02	2.44E-02	1.92E-01
rs4526799	Brain_Cerebellum	12	*RPS26*	56436876	rs11172049	0.9128	57304203	T	C	0.35	1.15E-02	2.25E-02	1.90E-01
rs4526799	Brain_Cerebellum	12	*ESYT1*	56530147	rs11172049	0.9128	57304203	T	C	0.35	1.15E-02	1.25E-02	1.77E-01
rs4526799	Brain_Cerebellum	12	*CS*	56679829	rs11172049	0.9128	57304203	T	C	0.35	1.15E-02	4.62E-02	2.12E-01
rs4526799	Brain_Cerebellum	12	*RDH16*	57349188	rs11172049	0.9128	57304203	T	C	0.35	1.15E-02	1.36E-02	1.79E-01
rs4526799	Brain_Cerebellum	12	*MBD6*	57919188	rs11172049	0.9128	57304203	T	C	0.35	1.15E-02	3.36E-02	2.01E-01
rs4526799	Brain_Cerebellum	12	*CDK2*	56363560	rs1874888	0.9085	57305138	A	C	0.35	1.15E-02	2.44E-02	1.92E-01
rs4526799	Brain_Cerebellum	12	*RPS26*	56436876	rs1874888	0.9085	57305138	A	C	0.35	1.15E-02	2.25E-02	1.90E-01
rs4526799	Brain_Cerebellum	12	*ESYT1*	56530147	rs1874888	0.9085	57305138	A	C	0.35	1.15E-02	1.25E-02	1.77E-01
rs4526799	Brain_Cerebellum	12	*CS*	56679829	rs1874888	0.9085	57305138	A	C	0.35	1.15E-02	4.62E-02	2.12E-01
rs4526799	Brain_Cerebellum	12	*RDH16*	57349188	rs1874888	0.9085	57305138	A	C	0.35	1.15E-02	1.36E-02	1.79E-01
rs4526799	Brain_Cerebellum	12	*MBD6*	57919188	rs1874888	0.9085	57305138	A	C	0.35	1.15E-02	3.36E-02	2.01E-01
rs4526799	Brain_Cerebellum	12	*ESYT1*	56530147	rs10506349	0.9128	57306412	T	C	0.37	1.10E-02	3.72E-02	1.95E-01
rs4526799	Brain_Cerebellum	12	*MIP*	56853054	rs10506349	0.9128	57306412	T	C	0.37	1.10E-02	4.85E-02	2.05E-01
rs4526799	Brain_Cerebellum	12	*RDH16*	57349188	rs10506349	0.9128	57306412	T	C	0.37	1.10E-02	3.11E-02	1.89E-01
rs4526799	Brain_Cerebellum	12	*KIF5A*	57962098	rs10506349	0.9128	57306412	T	C	0.37	1.10E-02	4.41E-02	2.01E-01
rs4526799	Brain_Cerebellum	12	*CDK2*	56363560	rs10876951	0.9085	57306430	T	G	0.35	1.15E-02	2.44E-02	1.92E-01
rs4526799	Brain_Cerebellum	12	*RPS26*	56436876	rs10876951	0.9085	57306430	T	G	0.35	1.15E-02	2.25E-02	1.90E-01
rs4526799	Brain_Cerebellum	12	*ESYT1*	56530147	rs10876951	0.9085	57306430	T	G	0.35	1.15E-02	1.25E-02	1.77E-01
rs4526799	Brain_Cerebellum	12	*CS*	56679829	rs10876951	0.9085	57306430	T	G	0.35	1.15E-02	4.62E-02	2.12E-01
rs4526799	Brain_Cerebellum	12	*RDH16*	57349188	rs10876951	0.9085	57306430	T	G	0.35	1.15E-02	1.36E-02	1.79E-01
rs4526799	Brain_Cerebellum	12	*MBD6*	57919188	rs10876951	0.9085	57306430	T	G	0.35	1.15E-02	3.36E-02	2.01E-01
rs4526799	Brain_Cerebellum	12	*ESYT1*	56530147	rs10747774	0.9128	57307079	T	C	0.36	1.16E-02	3.72E-02	2.00E-01
rs4526799	Brain_Cerebellum	12	*MIP*	56853054	rs10747774	0.9128	57307079	T	C	0.36	1.16E-02	4.85E-02	2.10E-01

rs4526799	Brain_Cerebellum	12	*RDH16*	57349188	rs10747774	0.9128	57307079	T	C	0.36	1.16E-02	3.11E-02	1.94E-01
rs4526799	Brain_Cerebellum	12	*KIF5A*	57962098	rs10747774	0.9128	57307079	T	C	0.36	1.16E-02	4.41E-02	2.06E-01
rs4526799	Brain_Cerebellum	12	*CDK2*	56363560	rs10783812	0.9085	57308723	C	T	0.35	1.19E-02	2.45E-02	1.95E-01
rs4526799	Brain_Cerebellum	12	*RPS26*	56436876	rs10783812	0.9085	57308723	C	T	0.35	1.19E-02	2.21E-02	1.92E-01
rs4526799	Brain_Cerebellum	12	*ESYT1*	56530147	rs10783812	0.9085	57308723	C	T	0.35	1.19E-02	1.27E-02	1.81E-01
rs4526799	Brain_Cerebellum	12	*CS*	56679829	rs10783812	0.9085	57308723	C	T	0.35	1.19E-02	4.49E-02	2.13E-01
rs4526799	Brain_Cerebellum	12	*RDH16*	57349188	rs10783812	0.9085	57308723	C	T	0.35	1.19E-02	1.30E-02	1.81E-01
rs4526799	Brain_Cerebellum	12	*MBD6*	57919188	rs10783812	0.9085	57308723	C	T	0.35	1.19E-02	3.31E-02	2.03E-01
rs4526799	Brain_Cerebellum	12	*ESYT1*	56530147	rs11172056	0.9089	57308975	C	T	0.37	1.10E-02	3.72E-02	1.95E-01
rs4526799	Brain_Cerebellum	12	*MIP*	56853054	rs11172056	0.9089	57308975	C	T	0.37	1.10E-02	4.85E-02	2.05E-01
rs4526799	Brain_Cerebellum	12	*RDH16*	57349188	rs11172056	0.9089	57308975	C	T	0.37	1.10E-02	3.11E-02	1.89E-01
rs4526799	Brain_Cerebellum	12	*KIF5A*	57962098	rs11172056	0.9089	57308975	C	T	0.37	1.10E-02	4.41E-02	2.01E-01
rs4526799	Brain_Cerebellum	12	*CDK2*	56363560	rs7302420	0.9085	57309884	G	C	0.36	1.54E-02	3.02E-02	2.16E-01
rs4526799	Brain_Cerebellum	12	*RPS26*	56436876	rs7302420	0.9085	57309884	G	C	0.36	1.54E-02	3.50E-02	2.20E-01
rs4526799	Brain_Cerebellum	12	*ESYT1*	56530147	rs7302420	0.9085	57309884	G	C	0.36	1.54E-02	1.13E-02	1.95E-01
rs4526799	Brain_Cerebellum	12	*RDH16*	57349188	rs7302420	0.9085	57309884	G	C	0.36	1.54E-02	2.34E-02	2.10E-01
rs4526799	Brain_Cerebellum	12	*KIF5A*	57962098	rs7302420	0.9085	57309884	G	C	0.36	1.54E-02	4.71E-02	2.30E-01
rs4526799	Brain_Cerebellum	12	*MIP*	56853054	rs12228618	0.9128	57311229	T	C	0.37	1.29E-02	3.55E-02	2.02E-01
rs4526799	Brain_Cerebellum	12	*RDH16*	57349188	rs12228618	0.9128	57311229	T	C	0.37	1.29E-02	2.57E-02	1.93E-01
rs4526799	Brain_Cerebellum	12	*KIF5A*	57962098	rs12228618	0.9128	57311229	T	C	0.37	1.29E-02	3.52E-02	2.02E-01
rs4526799	Brain_Cerebellum	12	*RDH16*	57349188	rs9739473	0.9012	57313335	A	T	0.37	2.65E-02	4.02E-02	2.71E-01
rs4526799	Brain_Cortex	12	*PMEL*	56357495	rs9919772	0.8450	57260027	T	C	0.33	1.03E-02	2.81E-03	1.83E-01
rs4526799	Brain_Cortex	12	*RP11-977G19.11*	56701259	rs9919772	0.8450	57260027	T	C	0.33	1.03E-02	2.40E-02	2.14E-01
rs4526799	Brain_Cortex	12	*TMEM194A*	57465636	rs9919772	0.8450	57260027	T	C	0.33	1.03E-02	1.39E-02	2.03E-01
rs4526799	Brain_Cortex	12	*PMEL*	56357495	rs4495925	0.8650	57268116	C	G	0.33	1.04E-02	2.58E-03	1.78E-01
rs4526799	Brain_Cortex	12	*RPL41*	56511048	rs4495925	0.8650	57268116	C	G	0.33	1.04E-02	2.95E-02	2.16E-01
rs4526799	Brain_Cortex	12	*RP11-977G19.11*	56701259	rs4495925	0.8650	57268116	C	G	0.33	1.04E-02	1.87E-02	2.05E-01
rs4526799	Brain_Cortex	12	*TMEM194A*	57465636	rs4495925	0.8650	57268116	C	G	0.33	1.04E-02	1.35E-02	1.99E-01
rs4526799	Brain_Cortex	12	*PMEL*	56357495	rs4471472	0.8650	57268985	A	G	0.32	9.99E-03	2.58E-03	1.74E-01
rs4526799	Brain_Cortex	12	*RPL41*	56511048	rs4471472	0.8650	57268985	A	G	0.32	9.99E-03	2.95E-02	2.12E-01
rs4526799	Brain_Cortex	12	*RP11-977G19.11*	56701259	rs4471472	0.8650	57268985	A	G	0.32	9.99E-03	1.87E-02	2.01E-01
rs4526799	Brain_Cortex	12	*TMEM194A*	57465636	rs4471472	0.8650	57268985	A	G	0.32	9.99E-03	1.35E-02	1.95E-01
rs4526799	Brain_Cortex	12	*PMEL*	56357495	rs4633499	0.8610	57269264	A	T	0.32	1.43E-02	4.15E-03	2.17E-01
rs4526799	Brain_Cortex	12	*RPS26*	56436876	rs4633499	0.8610	57269264	A	T	0.32	1.43E-02	3.45E-02	2.51E-01
rs4526799	Brain_Cortex	12	*RPL41*	56511048	rs4633499	0.8610	57269264	A	T	0.32	1.43E-02	1.81E-02	2.36E-01
rs4526799	Brain_Cortex	12	*RP11-977G19.11*	56701259	rs4633499	0.8610	57269264	A	T	0.32	1.43E-02	4.31E-03	2.17E-01
rs4526799	Brain_Cortex	12	*TIMELESS*	56827045	rs4633499	0.8610	57269264	A	T	0.32	1.43E-02	4.64E-02	2.60E-01
rs4526799	Brain_Cortex	12	*TMEM194A*	57465636	rs4633499	0.8610	57269264	A	T	0.32	1.43E-02	3.63E-02	2.52E-01
rs4526799	Brain_Cortex	12	*WIBG*	56310770	rs12300079	0.9956	57273194	T	C	0.36	3.73E-03	3.72E-02	1.72E-01
rs4526799	Brain_Cortex	12	*PMEL*	56357495	rs12300079	0.9956	57273194	T	C	0.36	3.73E-03	2.36E-03	1.20E-01

rs4526799	Brain_Cortex	12	*STAC3*	57641106	rs12300079	0.9956	57273194	T	C	0.36	3.73E-03	4.54E-02	1.79E-01
rs4526799	Brain_Cortex	12	*WIBG*	56310770	rs12300191	0.9956	57273289	A	G	0.36	3.59E-03	3.72E-02	1.70E-01
rs4526799	Brain_Cortex	12	*PMEL*	56357495	rs12300191	0.9956	57273289	A	G	0.36	3.59E-03	2.36E-03	1.17E-01
rs4526799	Brain_Cortex	12	*STAC3*	57641106	rs12300191	0.9956	57273289	A	G	0.36	3.59E-03	4.54E-02	1.77E-01
rs4526799	Brain_Cortex	12	*WIBG*	56310770	rs4514464	0.9956	57276375	C	T	0.36	2.92E-03	3.72E-02	1.67E-01
rs4526799	Brain_Cortex	12	*PMEL*	56357495	rs4514464	0.9956	57276375	C	T	0.36	2.92E-03	2.36E-03	1.14E-01
rs4526799	Brain_Cortex	12	*STAC3*	57641106	rs4514464	0.9956	57276375	C	T	0.36	2.92E-03	4.54E-02	1.74E-01
rs4526799	Brain_Cortex	12	*WIBG*	56310770	rs4417325	0.9956	57277302	G	A	0.36	3.59E-03	3.72E-02	1.70E-01
rs4526799	Brain_Cortex	12	*PMEL*	56357495	rs4417325	0.9956	57277302	G	A	0.36	3.59E-03	2.36E-03	1.17E-01
rs4526799	Brain_Cortex	12	*STAC3*	57641106	rs4417325	0.9956	57277302	G	A	0.36	3.59E-03	4.54E-02	1.77E-01
rs4526799	Brain_Cortex	12	*WIBG*	56310770	rs11172030	0.9956	57278076	A	C	0.36	3.63E-03	3.72E-02	1.70E-01
rs4526799	Brain_Cortex	12	*PMEL*	56357495	rs11172030	0.9956	57278076	A	C	0.36	3.63E-03	2.36E-03	1.18E-01
rs4526799	Brain_Cortex	12	*STAC3*	57641106	rs11172030	0.9956	57278076	A	C	0.36	3.63E-03	4.54E-02	1.78E-01
rs4526799	Brain_Cortex	12	*WIBG*	56310770	rs10876944	0.9956	57279372	T	A	0.36	3.63E-03	3.72E-02	1.70E-01
rs4526799	Brain_Cortex	12	*PMEL*	56357495	rs10876944	0.9956	57279372	T	A	0.36	3.63E-03	2.36E-03	1.18E-01
rs4526799	Brain_Cortex	12	*STAC3*	57641106	rs10876944	0.9956	57279372	T	A	0.36	3.63E-03	4.54E-02	1.78E-01
rs4526799	Brain_Cortex	12	*WIBG*	56310770	rs4326839	0.9956	57280374	G	C	0.36	3.58E-03	3.72E-02	1.69E-01
rs4526799	Brain_Cortex	12	*PMEL*	56357495	rs4326839	0.9956	57280374	G	C	0.36	3.58E-03	2.36E-03	1.17E-01
rs4526799	Brain_Cortex	12	*STAC3*	57641106	rs4326839	0.9956	57280374	G	C	0.36	3.58E-03	4.54E-02	1.77E-01
rs4526799	Brain_Cortex	12	*WIBG*	56310770	rs4526799	1.0000	57280586	T	C	0.34	7.26E-06	3.72E-02	7.24E-02
rs4526799	Brain_Cortex	12	*PMEL*	56357495	rs4526799	1.0000	57280586	T	C	0.34	7.26E-06	2.36E-03	2.09E-02
rs4526799	Brain_Cortex	12	*STAC3*	57641106	rs4526799	1.0000	57280586	T	C	0.34	7.26E-06	4.54E-02	8.12E-02
rs4526799	Brain_Cortex	12	*WIBG*	56310770	rs28876529	0.9956	57285301	T	A	0.36	2.25E-03	3.72E-02	1.55E-01
rs4526799	Brain_Cortex	12	*PMEL*	56357495	rs28876529	0.9956	57285301	T	A	0.36	2.25E-03	2.36E-03	1.01E-01
rs4526799	Brain_Cortex	12	*STAC3*	57641106	rs28876529	0.9956	57285301	T	A	0.36	2.25E-03	4.54E-02	1.62E-01
rs4526799	Brain_Cortex	12	*WIBG*	56310770	rs11172037	0.9956	57285427	T	A	0.36	2.25E-03	3.72E-02	1.55E-01
rs4526799	Brain_Cortex	12	*PMEL*	56357495	rs11172037	0.9956	57285427	T	A	0.36	2.25E-03	2.36E-03	1.01E-01
rs4526799	Brain_Cortex	12	*STAC3*	57641106	rs11172037	0.9956	57285427	T	A	0.36	2.25E-03	4.54E-02	1.62E-01
rs4526799	Brain_Cortex	12	*WIBG*	56310770	rs12321987	0.9956	57288449	G	A	0.36	4.47E-03	3.53E-02	1.89E-01
rs4526799	Brain_Cortex	12	*PMEL*	56357495	rs12321987	0.9956	57288449	G	A	0.36	4.47E-03	2.39E-03	1.41E-01
rs4526799	Brain_Cortex	12	*STAC3*	57641106	rs12321987	0.9956	57288449	G	A	0.36	4.47E-03	4.52E-02	1.97E-01
rs4526799	Brain_Cortex	12	*WIBG*	56310770	rs11172043	0.9869	57293182	G	A	0.35	4.58E-03	3.51E-02	1.87E-01
rs4526799	Brain_Cortex	12	*PMEL*	56357495	rs11172043	0.9869	57293182	G	A	0.35	4.58E-03	1.58E-02	1.66E-01
rs4526799	Brain_Cortex	12	*RPS26*	56436876	rs11172043	0.9869	57293182	G	A	0.35	4.58E-03	4.02E-02	1.92E-01
rs4526799	Brain_Cortex	12	*ANKRD52*	56641903	rs11172043	0.9869	57293182	G	A	0.35	4.58E-03	4.17E-02	1.93E-01
rs4526799	Brain_Cortex	12	*RP11-977G19.11*	56701259	rs11172043	0.9869	57293182	G	A	0.35	4.58E-03	2.64E-02	1.78E-01
rs4526799	Brain_Cortex	12	*TIMELESS*	56827045	rs11172043	0.9869	57293182	G	A	0.35	4.58E-03	2.07E-02	1.72E-01
rs4526799	Brain_Cortex	12	*SHMT2*	57625897	rs11172043	0.9869	57293182	G	A	0.35	4.58E-03	4.26E-02	1.94E-01
rs4526799	Brain_Cortex	12	*STAC3*	57641106	rs11172043	0.9869	57293182	G	A	0.35	4.58E-03	3.17E-02	1.84E-01
rs4526799	Brain_Cortex	12	*WIBG*	56310770	rs12426816	0.9869	57294074	A	C	0.35	4.50E-03	3.51E-02	1.87E-01

rs4526799	Brain_Cortex	12	*PMEL*	56357495	rs12426816	0.9869	57294074	A	C	0.35	4.50E-03	1.58E-02	1.65E-01
rs4526799	Brain_Cortex	12	*RPS26*	56436876	rs12426816	0.9869	57294074	A	C	0.35	4.50E-03	4.02E-02	1.91E-01
rs4526799	Brain_Cortex	12	*ANKRD52*	56641903	rs12426816	0.9869	57294074	A	C	0.35	4.50E-03	4.17E-02	1.93E-01
rs4526799	Brain_Cortex	12	*RP11-977G19.11*	56701259	rs12426816	0.9869	57294074	A	C	0.35	4.50E-03	2.64E-02	1.78E-01
rs4526799	Brain_Cortex	12	*TIMELESS*	56827045	rs12426816	0.9869	57294074	A	C	0.35	4.50E-03	2.07E-02	1.72E-01
rs4526799	Brain_Cortex	12	*SHMT2*	57625897	rs12426816	0.9869	57294074	A	C	0.35	4.50E-03	4.26E-02	1.94E-01
rs4526799	Brain_Cortex	12	*STAC3*	57641106	rs12426816	0.9869	57294074	A	C	0.35	4.50E-03	3.17E-02	1.83E-01
rs4526799	Brain_Cortex	12	*WIBG*	56310770	rs11172047	0.9869	57298080	T	C	0.35	6.51E-03	3.51E-02	2.00E-01
rs4526799	Brain_Cortex	12	*PMEL*	56357495	rs11172047	0.9869	57298080	T	C	0.35	6.51E-03	1.58E-02	1.80E-01
rs4526799	Brain_Cortex	12	*RPS26*	56436876	rs11172047	0.9869	57298080	T	C	0.35	6.51E-03	4.02E-02	2.05E-01
rs4526799	Brain_Cortex	12	*ANKRD52*	56641903	rs11172047	0.9869	57298080	T	C	0.35	6.51E-03	4.17E-02	2.06E-01
rs4526799	Brain_Cortex	12	*RP11-977G19.11*	56701259	rs11172047	0.9869	57298080	T	C	0.35	6.51E-03	2.64E-02	1.92E-01
rs4526799	Brain_Cortex	12	*TIMELESS*	56827045	rs11172047	0.9869	57298080	T	C	0.35	6.51E-03	2.07E-02	1.86E-01
rs4526799	Brain_Cortex	12	*SHMT2*	57625897	rs11172047	0.9869	57298080	T	C	0.35	6.51E-03	4.26E-02	2.07E-01
rs4526799	Brain_Cortex	12	*STAC3*	57641106	rs11172047	0.9869	57298080	T	C	0.35	6.51E-03	3.17E-02	1.97E-01
rs4526799	Brain_Cortex	12	*WIBG*	56310770	rs2371631	0.9869	57298614	T	A	0.35	6.32E-03	3.51E-02	1.97E-01
rs4526799	Brain_Cortex	12	*PMEL*	56357495	rs2371631	0.9869	57298614	T	A	0.35	6.32E-03	1.58E-02	1.77E-01
rs4526799	Brain_Cortex	12	*RPS26*	56436876	rs2371631	0.9869	57298614	T	A	0.35	6.32E-03	4.02E-02	2.02E-01
rs4526799	Brain_Cortex	12	*ANKRD52*	56641903	rs2371631	0.9869	57298614	T	A	0.35	6.32E-03	4.17E-02	2.03E-01
rs4526799	Brain_Cortex	12	*RP11-977G19.11*	56701259	rs2371631	0.9869	57298614	T	A	0.35	6.32E-03	2.64E-02	1.89E-01
rs4526799	Brain_Cortex	12	*TIMELESS*	56827045	rs2371631	0.9869	57298614	T	A	0.35	6.32E-03	2.07E-02	1.83E-01
rs4526799	Brain_Cortex	12	*SHMT2*	57625897	rs2371631	0.9869	57298614	T	A	0.35	6.32E-03	4.26E-02	2.04E-01
rs4526799	Brain_Cortex	12	*STAC3*	57641106	rs2371631	0.9869	57298614	T	A	0.35	6.32E-03	3.17E-02	1.94E-01
rs4526799	Brain_Cortex	12	*WIBG*	56310770	rs12305763	0.9869	57299263	G	A	0.35	6.82E-03	3.51E-02	2.02E-01
rs4526799	Brain_Cortex	12	*PMEL*	56357495	rs12305763	0.9869	57299263	G	A	0.35	6.82E-03	1.58E-02	1.81E-01
rs4526799	Brain_Cortex	12	*RPS26*	56436876	rs12305763	0.9869	57299263	G	A	0.35	6.82E-03	4.02E-02	2.06E-01
rs4526799	Brain_Cortex	12	*ANKRD52*	56641903	rs12305763	0.9869	57299263	G	A	0.35	6.82E-03	4.17E-02	2.08E-01
rs4526799	Brain_Cortex	12	*RP11-977G19.11*	56701259	rs12305763	0.9869	57299263	G	A	0.35	6.82E-03	2.64E-02	1.93E-01
rs4526799	Brain_Cortex	12	*TIMELESS*	56827045	rs12305763	0.9869	57299263	G	A	0.35	6.82E-03	2.07E-02	1.87E-01
rs4526799	Brain_Cortex	12	*SHMT2*	57625897	rs12305763	0.9869	57299263	G	A	0.35	6.82E-03	4.26E-02	2.08E-01
rs4526799	Brain_Cortex	12	*STAC3*	57641106	rs12305763	0.9869	57299263	G	A	0.35	6.82E-03	3.17E-02	1.99E-01
rs4526799	Brain_Cortex	12	*WIBG*	56310770	rs11172049	0.9128	57304203	T	C	0.35	1.15E-02	2.10E-02	1.88E-01
rs4526799	Brain_Cortex	12	*PMEL*	56357495	rs11172049	0.9128	57304203	T	C	0.35	1.15E-02	1.10E-02	1.75E-01
rs4526799	Brain_Cortex	12	*ANKRD52*	56641903	rs11172049	0.9128	57304203	T	C	0.35	1.15E-02	4.02E-02	2.06E-01
rs4526799	Brain_Cortex	12	*RP11-977G19.11*	56701259	rs11172049	0.9128	57304203	T	C	0.35	1.15E-02	5.70E-03	1.65E-01
rs4526799	Brain_Cortex	12	*TIMELESS*	56827045	rs11172049	0.9128	57304203	T	C	0.35	1.15E-02	3.24E-02	1.99E-01
rs4526799	Brain_Cortex	12	*MBD6*	57919188	rs11172049	0.9128	57304203	T	C	0.35	1.15E-02	3.27E-02	2.00E-01
rs4526799	Brain_Cortex	12	*WIBG*	56310770	rs1874888	0.9085	57305138	A	C	0.35	1.15E-02	2.10E-02	1.88E-01
rs4526799	Brain_Cortex	12	*PMEL*	56357495	rs1874888	0.9085	57305138	A	C	0.35	1.15E-02	1.10E-02	1.75E-01
rs4526799	Brain_Cortex	12	*ANKRD52*	56641903	rs1874888	0.9085	57305138	A	C	0.35	1.15E-02	4.02E-02	2.06E-01

rs4526799	Brain_Cortex	12	*RP11-977G19.11*	56701259	rs1874888	0.9085	57305138	A	C	0.35	1.15E-02	5.70E-03	1.65E-01
rs4526799	Brain_Cortex	12	*TIMELESS*	56827045	rs1874888	0.9085	57305138	A	C	0.35	1.15E-02	3.24E-02	1.99E-01
rs4526799	Brain_Cortex	12	*MBD6*	57919188	rs1874888	0.9085	57305138	A	C	0.35	1.15E-02	3.27E-02	2.00E-01
rs4526799	Brain_Cortex	12	*WIBG*	56310770	rs10506349	0.9128	57306412	T	C	0.37	1.10E-02	1.32E-02	1.69E-01
rs4526799	Brain_Cortex	12	*PMEL*	56357495	rs10506349	0.9128	57306412	T	C	0.37	1.10E-02	4.67E-02	2.03E-01
rs4526799	Brain_Cortex	12	*RP11-977G19.11*	56701259	rs10506349	0.9128	57306412	T	C	0.37	1.10E-02	1.48E-02	1.71E-01
rs4526799	Brain_Cortex	12	*MBD6*	57919188	rs10506349	0.9128	57306412	T	C	0.37	1.10E-02	3.56E-02	1.93E-01
rs4526799	Brain_Cortex	12	*WIBG*	56310770	rs10876951	0.9085	57306430	T	G	0.35	1.15E-02	2.10E-02	1.88E-01
rs4526799	Brain_Cortex	12	*PMEL*	56357495	rs10876951	0.9085	57306430	T	G	0.35	1.15E-02	1.10E-02	1.75E-01
rs4526799	Brain_Cortex	12	*ANKRD52*	56641903	rs10876951	0.9085	57306430	T	G	0.35	1.15E-02	4.02E-02	2.06E-01
rs4526799	Brain_Cortex	12	*RP11-977G19.11*	56701259	rs10876951	0.9085	57306430	T	G	0.35	1.15E-02	5.70E-03	1.65E-01
rs4526799	Brain_Cortex	12	*TIMELESS*	56827045	rs10876951	0.9085	57306430	T	G	0.35	1.15E-02	3.24E-02	1.99E-01
rs4526799	Brain_Cortex	12	*MBD6*	57919188	rs10876951	0.9085	57306430	T	G	0.35	1.15E-02	3.27E-02	2.00E-01
rs4526799	Brain_Cortex	12	*WIBG*	56310770	rs10747774	0.9128	57307079	T	C	0.36	1.16E-02	1.28E-02	1.73E-01
rs4526799	Brain_Cortex	12	*RP11-977G19.11*	56701259	rs10747774	0.9128	57307079	T	C	0.36	1.16E-02	1.44E-02	1.76E-01
rs4526799	Brain_Cortex	12	*MBD6*	57919188	rs10747774	0.9128	57307079	T	C	0.36	1.16E-02	3.16E-02	1.95E-01
rs4526799	Brain_Cortex	12	*WIBG*	56310770	rs10783812	0.9085	57308723	C	T	0.35	1.19E-02	1.68E-02	1.86E-01
rs4526799	Brain_Cortex	12	*PMEL*	56357495	rs10783812	0.9085	57308723	C	T	0.35	1.19E-02	1.23E-02	1.80E-01
rs4526799	Brain_Cortex	12	*ANKRD52*	56641903	rs10783812	0.9085	57308723	C	T	0.35	1.19E-02	4.02E-02	2.09E-01
rs4526799	Brain_Cortex	12	*RP11-977G19.11*	56701259	rs10783812	0.9085	57308723	C	T	0.35	1.19E-02	5.38E-03	1.68E-01
rs4526799	Brain_Cortex	12	*TIMELESS*	56827045	rs10783812	0.9085	57308723	C	T	0.35	1.19E-02	2.74E-02	1.98E-01
rs4526799	Brain_Cortex	12	*MBD6*	57919188	rs10783812	0.9085	57308723	C	T	0.35	1.19E-02	3.61E-02	2.06E-01
rs4526799	Brain_Cortex	12	*WIBG*	56310770	rs11172056	0.9089	57308975	C	T	0.37	1.10E-02	1.28E-02	1.68E-01
rs4526799	Brain_Cortex	12	*RP11-977G19.11*	56701259	rs11172056	0.9089	57308975	C	T	0.37	1.10E-02	1.44E-02	1.71E-01
rs4526799	Brain_Cortex	12	*MBD6*	57919188	rs11172056	0.9089	57308975	C	T	0.37	1.10E-02	3.16E-02	1.90E-01
rs4526799	Brain_Cortex	12	*WIBG*	56310770	rs7302420	0.9085	57309884	G	C	0.36	1.54E-02	2.91E-02	2.15E-01
rs4526799	Brain_Cortex	12	*PMEL*	56357495	rs7302420	0.9085	57309884	G	C	0.36	1.54E-02	1.47E-02	2.00E-01
rs4526799	Brain_Cortex	12	*ANKRD52*	56641903	rs7302420	0.9085	57309884	G	C	0.36	1.54E-02	4.90E-02	2.32E-01
rs4526799	Brain_Cortex	12	*RP11-977G19.11*	56701259	rs7302420	0.9085	57309884	G	C	0.36	1.54E-02	3.00E-03	1.79E-01
rs4526799	Brain_Cortex	12	*TIMELESS*	56827045	rs7302420	0.9085	57309884	G	C	0.36	1.54E-02	2.73E-02	2.13E-01
rs4526799	Brain_Cortex	12	*SHMT2*	57625897	rs7302420	0.9085	57309884	G	C	0.36	1.54E-02	3.44E-02	2.20E-01
rs4526799	Brain_Cortex	12	*MBD6*	57919188	rs7302420	0.9085	57309884	G	C	0.36	1.54E-02	2.99E-02	2.16E-01
rs4526799	Brain_Cortex	12	*WIBG*	56310770	rs12228618	0.9128	57311229	T	C	0.37	1.29E-02	2.02E-02	1.87E-01
rs4526799	Brain_Cortex	12	*PMEL*	56357495	rs12228618	0.9128	57311229	T	C	0.37	1.29E-02	2.79E-02	1.95E-01
rs4526799	Brain_Cortex	12	*RP11-977G19.11*	56701259	rs12228618	0.9128	57311229	T	C	0.37	1.29E-02	2.60E-02	1.93E-01
rs4526799	Brain_Cortex	12	*MBD6*	57919188	rs12228618	0.9128	57311229	T	C	0.37	1.29E-02	1.51E-02	1.80E-01
rs4526799	Brain_Cortex	12	*WIBG*	56310770	rs9739473	0.9012	57313335	A	T	0.37	2.65E-02	1.27E-02	2.48E-01
rs4526799	Brain_Cortex	12	*PMEL*	56357495	rs9739473	0.9012	57313335	A	T	0.37	2.65E-02	1.97E-02	2.55E-01
rs4526799	Brain_Cortex	12	*CDK2*	56363560	rs9739473	0.9012	57313335	A	T	0.37	2.65E-02	3.46E-02	2.67E-01
rs4526799	Brain_Cortex	12	*RP11-977G19.11*	56701259	rs9739473	0.9012	57313335	A	T	0.37	2.65E-02	1.48E-02	2.51E-01

rs4526799	Brain_Cortex	12	*MBD6*	57919188	rs9739473	0.9012	57313335	A	T	0.37	2.65E-02	2.52E-02	2.60E-01
rs4526799	Brain_Frontal_Cortex_BA9	12	*IKZF4*	56416831	rs9919772	0.8450	57260027	T	C	0.33	1.03E-02	4.11E-02	2.29E-01
rs4526799	Brain_Frontal_Cortex_BA9	12	*RPS26*	56436876	rs9919772	0.8450	57260027	T	C	0.33	1.03E-02	2.61E-02	2.16E-01
rs4526799	Brain_Frontal_Cortex_BA9	12	*COQ10A*	56662696	rs9919772	0.8450	57260027	T	C	0.33	1.03E-02	2.80E-02	2.18E-01
rs4526799	Brain_Frontal_Cortex_BA9	12	*RP11-977G19.11*	56701259	rs9919772	0.8450	57260027	T	C	0.33	1.03E-02	7.01E-04	1.73E-01
rs4526799	Brain_Frontal_Cortex_BA9	12	*DTX3*	58000996	rs9919772	0.8450	57260027	T	C	0.33	1.03E-02	1.57E-02	2.05E-01
rs4526799	Brain_Frontal_Cortex_BA9	12	*IKZF4*	56416831	rs4495925	0.8650	57268116	C	G	0.33	1.04E-02	2.79E-02	2.15E-01
rs4526799	Brain_Frontal_Cortex_BA9	12	*RPS26*	56436876	rs4495925	0.8650	57268116	C	G	0.33	1.04E-02	1.37E-02	1.99E-01
rs4526799	Brain_Frontal_Cortex_BA9	12	*COQ10A*	56662696	rs4495925	0.8650	57268116	C	G	0.33	1.04E-02	2.86E-02	2.15E-01
rs4526799	Brain_Frontal_Cortex_BA9	12	*RP11-977G19.11*	56701259	rs4495925	0.8650	57268116	C	G	0.33	1.04E-02	1.90E-03	1.76E-01
rs4526799	Brain_Frontal_Cortex_BA9	12	*INHBE*	57849584	rs4495925	0.8650	57268116	C	G	0.33	1.04E-02	2.67E-02	2.13E-01
rs4526799	Brain_Frontal_Cortex_BA9	12	*DTX3*	58000996	rs4495925	0.8650	57268116	C	G	0.33	1.04E-02	1.57E-02	2.02E-01
rs4526799	Brain_Frontal_Cortex_BA9	12	*IKZF4*	56416831	rs4471472	0.8650	57268985	A	G	0.32	9.99E-03	2.79E-02	2.11E-01
rs4526799	Brain_Frontal_Cortex_BA9	12	*RPS26*	56436876	rs4471472	0.8650	57268985	A	G	0.32	9.99E-03	1.37E-02	1.95E-01
rs4526799	Brain_Frontal_Cortex_BA9	12	*COQ10A*	56662696	rs4471472	0.8650	57268985	A	G	0.32	9.99E-03	2.86E-02	2.11E-01
rs4526799	Brain_Frontal_Cortex_BA9	12	*RP11-977G19.11*	56701259	rs4471472	0.8650	57268985	A	G	0.32	9.99E-03	1.90E-03	1.72E-01
rs4526799	Brain_Frontal_Cortex_BA9	12	*INHBE*	57849584	rs4471472	0.8650	57268985	A	G	0.32	9.99E-03	2.67E-02	2.10E-01
rs4526799	Brain_Frontal_Cortex_BA9	12	*DTX3*	58000996	rs4471472	0.8650	57268985	A	G	0.32	9.99E-03	1.57E-02	1.98E-01
rs4526799	Brain_Frontal_Cortex_BA9	12	*IKZF4*	56416831	rs4633499	0.8610	57269264	A	T	0.32	1.43E-02	1.87E-02	2.37E-01
rs4526799	Brain_Frontal_Cortex_BA9	12	*RPS26*	56436876	rs4633499	0.8610	57269264	A	T	0.32	1.43E-02	7.78E-03	2.24E-01
rs4526799	Brain_Frontal_Cortex_BA9	12	*COQ10A*	56662696	rs4633499	0.8610	57269264	A	T	0.32	1.43E-02	3.91E-02	2.54E-01
rs4526799	Brain_Frontal_Cortex_BA9	12	*RP11-977G19.11*	56701259	rs4633499	0.8610	57269264	A	T	0.32	1.43E-02	1.43E-03	2.09E-01
rs4526799	Brain_Frontal_Cortex_BA9	12	*INHBE*	57849584	rs4633499	0.8610	57269264	A	T	0.32	1.43E-02	3.25E-02	2.49E-01
rs4526799	Brain_Frontal_Cortex_BA9	12	*DTX3*	58000996	rs4633499	0.8610	57269264	A	T	0.32	1.43E-02	8.36E-03	2.25E-01
rs4526799	Brain_Frontal_Cortex_BA9	12	*IKZF4*	56416831	rs12300079	0.9956	57273194	T	C	0.36	3.73E-03	2.25E-02	1.56E-01
rs4526799	Brain_Frontal_Cortex_BA9	12	*COQ10A*	56662696	rs12300079	0.9956	57273194	T	C	0.36	3.73E-03	4.18E-02	1.76E-01
rs4526799	Brain_Frontal_Cortex_BA9	12	*RP11-977G19.11*	56701259	rs12300079	0.9956	57273194	T	C	0.36	3.73E-03	2.17E-02	1.55E-01
rs4526799	Brain_Frontal_Cortex_BA9	12	*IKZF4*	56416831	rs12300191	0.9956	57273289	A	G	0.36	3.59E-03	2.60E-02	1.58E-01
rs4526799	Brain_Frontal_Cortex_BA9	12	*COQ10A*	56662696	rs12300191	0.9956	57273289	A	G	0.36	3.59E-03	4.08E-02	1.73E-01
rs4526799	Brain_Frontal_Cortex_BA9	12	*RP11-977G19.11*	56701259	rs12300191	0.9956	57273289	A	G	0.36	3.59E-03	1.96E-02	1.50E-01
rs4526799	Brain_Frontal_Cortex_BA9	12	*IKZF4*	56416831	rs4514464	0.9956	57276375	C	T	0.36	2.92E-03	2.60E-02	1.55E-01
rs4526799	Brain_Frontal_Cortex_BA9	12	*COQ10A*	56662696	rs4514464	0.9956	57276375	C	T	0.36	2.92E-03	4.08E-02	1.70E-01
rs4526799	Brain_Frontal_Cortex_BA9	12	*RP11-977G19.11*	56701259	rs4514464	0.9956	57276375	C	T	0.36	2.92E-03	1.96E-02	1.47E-01
rs4526799	Brain_Frontal_Cortex_BA9	12	*IKZF4*	56416831	rs4417325	0.9956	57277302	G	A	0.36	3.59E-03	2.60E-02	1.58E-01
rs4526799	Brain_Frontal_Cortex_BA9	12	*COQ10A*	56662696	rs4417325	0.9956	57277302	G	A	0.36	3.59E-03	4.08E-02	1.73E-01
rs4526799	Brain_Frontal_Cortex_BA9	12	*RP11-977G19.11*	56701259	rs4417325	0.9956	57277302	G	A	0.36	3.59E-03	1.96E-02	1.50E-01
rs4526799	Brain_Frontal_Cortex_BA9	12	*IKZF4*	56416831	rs11172030	0.9956	57278076	A	C	0.36	3.63E-03	2.60E-02	1.58E-01
rs4526799	Brain_Frontal_Cortex_BA9	12	*COQ10A*	56662696	rs11172030	0.9956	57278076	A	C	0.36	3.63E-03	4.08E-02	1.73E-01
rs4526799	Brain_Frontal_Cortex_BA9	12	*RP11-977G19.11*	56701259	rs11172030	0.9956	57278076	A	C	0.36	3.63E-03	1.96E-02	1.51E-01
rs4526799	Brain_Frontal_Cortex_BA9	12	*IKZF4*	56416831	rs10876944	0.9956	57279372	T	A	0.36	3.63E-03	2.60E-02	1.58E-01

rs4526799	Brain_Frontal_Cortex_BA9	12	*COQ10A*	56662696	rs10876944	0.9956	57279372	T	A	0.36	3.63E-03	4.08E-02	1.73E-01
rs4526799	Brain_Frontal_Cortex_BA9	12	*RP11-977G19.11*	56701259	rs10876944	0.9956	57279372	T	A	0.36	3.63E-03	1.96E-02	1.51E-01
rs4526799	Brain_Frontal_Cortex_BA9	12	*IKZF4*	56416831	rs4326839	0.9956	57280374	G	C	0.36	3.58E-03	2.60E-02	1.58E-01
rs4526799	Brain_Frontal_Cortex_BA9	12	*COQ10A*	56662696	rs4326839	0.9956	57280374	G	C	0.36	3.58E-03	4.08E-02	1.73E-01
rs4526799	Brain_Frontal_Cortex_BA9	12	*RP11-977G19.11*	56701259	rs4326839	0.9956	57280374	G	C	0.36	3.58E-03	1.96E-02	1.50E-01
rs4526799	Brain_Frontal_Cortex_BA9	12	*IKZF4*	56416831	rs4526799	1.0000	57280586	T	C	0.34	7.26E-06	2.60E-02	5.92E-02
rs4526799	Brain_Frontal_Cortex_BA9	12	*COQ10A*	56662696	rs4526799	1.0000	57280586	T	C	0.34	7.26E-06	4.08E-02	7.63E-02
rs4526799	Brain_Frontal_Cortex_BA9	12	*RP11-977G19.11*	56701259	rs4526799	1.0000	57280586	T	C	0.34	7.26E-06	1.96E-02	5.11E-02
rs4526799	Brain_Frontal_Cortex_BA9	12	*IKZF4*	56416831	rs28876529	0.9956	57285301	T	A	0.36	2.25E-03	2.60E-02	1.42E-01
rs4526799	Brain_Frontal_Cortex_BA9	12	*COQ10A*	56662696	rs28876529	0.9956	57285301	T	A	0.36	2.25E-03	4.08E-02	1.58E-01
rs4526799	Brain_Frontal_Cortex_BA9	12	*RP11-977G19.11*	56701259	rs28876529	0.9956	57285301	T	A	0.36	2.25E-03	1.96E-02	1.34E-01
rs4526799	Brain_Frontal_Cortex_BA9	12	*IKZF4*	56416831	rs11172037	0.9956	57285427	T	A	0.36	2.25E-03	2.60E-02	1.42E-01
rs4526799	Brain_Frontal_Cortex_BA9	12	*COQ10A*	56662696	rs11172037	0.9956	57285427	T	A	0.36	2.25E-03	4.08E-02	1.58E-01
rs4526799	Brain_Frontal_Cortex_BA9	12	*RP11-977G19.11*	56701259	rs11172037	0.9956	57285427	T	A	0.36	2.25E-03	1.96E-02	1.34E-01
rs4526799	Brain_Frontal_Cortex_BA9	12	*IKZF4*	56416831	rs12321987	0.9956	57288449	G	A	0.36	4.47E-03	2.60E-02	1.79E-01
rs4526799	Brain_Frontal_Cortex_BA9	12	*COQ10A*	56662696	rs12321987	0.9956	57288449	G	A	0.36	4.47E-03	4.08E-02	1.94E-01
rs4526799	Brain_Frontal_Cortex_BA9	12	*RP11-977G19.11*	56701259	rs12321987	0.9956	57288449	G	A	0.36	4.47E-03	1.96E-02	1.72E-01
rs4526799	Brain_Frontal_Cortex_BA9	12	*IKZF4*	56416831	rs11172043	0.9869	57293182	G	A	0.35	4.58E-03	1.78E-02	1.68E-01
rs4526799	Brain_Frontal_Cortex_BA9	12	*RP11-977G19.11*	56701259	rs11172043	0.9869	57293182	G	A	0.35	4.58E-03	1.74E-02	1.68E-01
rs4526799	Brain_Frontal_Cortex_BA9	12	*INHBE*	57849584	rs11172043	0.9869	57293182	G	A	0.35	4.58E-03	4.03E-02	1.92E-01
rs4526799	Brain_Frontal_Cortex_BA9	12	*RP11-571M6.17*	58197909	rs11172043	0.9869	57293182	G	A	0.35	4.58E-03	4.47E-02	1.96E-01
rs4526799	Brain_Frontal_Cortex_BA9	12	*IKZF4*	56416831	rs12426816	0.9869	57294074	A	C	0.35	4.50E-03	1.78E-02	1.68E-01
rs4526799	Brain_Frontal_Cortex_BA9	12	*RP11-977G19.11*	56701259	rs12426816	0.9869	57294074	A	C	0.35	4.50E-03	1.74E-02	1.68E-01
rs4526799	Brain_Frontal_Cortex_BA9	12	*INHBE*	57849584	rs12426816	0.9869	57294074	A	C	0.35	4.50E-03	4.03E-02	1.91E-01
rs4526799	Brain_Frontal_Cortex_BA9	12	*RP11-571M6.17*	58197909	rs12426816	0.9869	57294074	A	C	0.35	4.50E-03	4.47E-02	1.95E-01
rs4526799	Brain_Frontal_Cortex_BA9	12	*IKZF4*	56416831	rs11172047	0.9869	57298080	T	C	0.35	6.51E-03	1.78E-02	1.83E-01
rs4526799	Brain_Frontal_Cortex_BA9	12	*RP11-977G19.11*	56701259	rs11172047	0.9869	57298080	T	C	0.35	6.51E-03	1.74E-02	1.82E-01
rs4526799	Brain_Frontal_Cortex_BA9	12	*INHBE*	57849584	rs11172047	0.9869	57298080	T	C	0.35	6.51E-03	4.03E-02	2.05E-01
rs4526799	Brain_Frontal_Cortex_BA9	12	*RP11-571M6.17*	58197909	rs11172047	0.9869	57298080	T	C	0.35	6.51E-03	4.47E-02	2.09E-01
rs4526799	Brain_Frontal_Cortex_BA9	12	*IKZF4*	56416831	rs2371631	0.9869	57298614	T	A	0.35	6.32E-03	1.78E-02	1.79E-01
rs4526799	Brain_Frontal_Cortex_BA9	12	*RP11-977G19.11*	56701259	rs2371631	0.9869	57298614	T	A	0.35	6.32E-03	1.74E-02	1.79E-01
rs4526799	Brain_Frontal_Cortex_BA9	12	*INHBE*	57849584	rs2371631	0.9869	57298614	T	A	0.35	6.32E-03	4.03E-02	2.02E-01
rs4526799	Brain_Frontal_Cortex_BA9	12	*RP11-571M6.17*	58197909	rs2371631	0.9869	57298614	T	A	0.35	6.32E-03	4.47E-02	2.06E-01
rs4526799	Brain_Frontal_Cortex_BA9	12	*IKZF4*	56416831	rs12305763	0.9869	57299263	G	A	0.35	6.82E-03	1.78E-02	1.84E-01
rs4526799	Brain_Frontal_Cortex_BA9	12	*RP11-977G19.11*	56701259	rs12305763	0.9869	57299263	G	A	0.35	6.82E-03	1.74E-02	1.84E-01
rs4526799	Brain_Frontal_Cortex_BA9	12	*INHBE*	57849584	rs12305763	0.9869	57299263	G	A	0.35	6.82E-03	4.03E-02	2.06E-01
rs4526799	Brain_Frontal_Cortex_BA9	12	*RP11-571M6.17*	58197909	rs12305763	0.9869	57299263	G	A	0.35	6.82E-03	4.47E-02	2.10E-01
rs4526799	Brain_Frontal_Cortex_BA9	12	*IKZF4*	56416831	rs11172049	0.9128	57304203	T	C	0.35	1.15E-02	4.29E-02	2.09E-01
rs4526799	Brain_Frontal_Cortex_BA9	12	*RP11-977G19.11*	56701259	rs11172049	0.9128	57304203	T	C	0.35	1.15E-02	2.76E-02	1.95E-01
rs4526799	Brain_Frontal_Cortex_BA9	12	*TAC3*	57413225	rs11172049	0.9128	57304203	T	C	0.35	1.15E-02	2.80E-02	1.95E-01

rs4526799	Brain_Frontal_Cortex_BA9	12	*NXPH4*	57615405	rs11172049	0.9128	57304203	T	C	0.35	1.15E-02	3.71E-02	2.04E-01
rs4526799	Brain_Frontal_Cortex_BA9	12	*DTX3*	58000996	rs11172049	0.9128	57304203	T	C	0.35	1.15E-02	1.54E-02	1.81E-01
rs4526799	Brain_Frontal_Cortex_BA9	12	*IKZF4*	56416831	rs1874888	0.9085	57305138	A	C	0.35	1.15E-02	4.29E-02	2.09E-01
rs4526799	Brain_Frontal_Cortex_BA9	12	*RP11-977G19.11*	56701259	rs1874888	0.9085	57305138	A	C	0.35	1.15E-02	2.76E-02	1.95E-01
rs4526799	Brain_Frontal_Cortex_BA9	12	*TAC3*	57413225	rs1874888	0.9085	57305138	A	C	0.35	1.15E-02	2.80E-02	1.95E-01
rs4526799	Brain_Frontal_Cortex_BA9	12	*NXPH4*	57615405	rs1874888	0.9085	57305138	A	C	0.35	1.15E-02	3.71E-02	2.04E-01
rs4526799	Brain_Frontal_Cortex_BA9	12	*DTX3*	58000996	rs1874888	0.9085	57305138	A	C	0.35	1.15E-02	1.54E-02	1.81E-01
rs4526799	Brain_Frontal_Cortex_BA9	12	*IKZF4*	56416831	rs10506349	0.9128	57306412	T	C	0.37	1.10E-02	3.03E-02	1.88E-01
rs4526799	Brain_Frontal_Cortex_BA9	12	*RP11-977G19.11*	56701259	rs10506349	0.9128	57306412	T	C	0.37	1.10E-02	2.72E-02	1.85E-01
rs4526799	Brain_Frontal_Cortex_BA9	12	*NXPH4*	57615405	rs10506349	0.9128	57306412	T	C	0.37	1.10E-02	3.11E-02	1.89E-01
rs4526799	Brain_Frontal_Cortex_BA9	12	*DTX3*	58000996	rs10506349	0.9128	57306412	T	C	0.37	1.10E-02	1.93E-02	1.77E-01
rs4526799	Brain_Frontal_Cortex_BA9	12	*IKZF4*	56416831	rs10876951	0.9085	57306430	T	G	0.35	1.15E-02	4.29E-02	2.09E-01
rs4526799	Brain_Frontal_Cortex_BA9	12	*RP11-977G19.11*	56701259	rs10876951	0.9085	57306430	T	G	0.35	1.15E-02	2.76E-02	1.95E-01
rs4526799	Brain_Frontal_Cortex_BA9	12	*TAC3*	57413225	rs10876951	0.9085	57306430	T	G	0.35	1.15E-02	2.80E-02	1.95E-01
rs4526799	Brain_Frontal_Cortex_BA9	12	*NXPH4*	57615405	rs10876951	0.9085	57306430	T	G	0.35	1.15E-02	3.71E-02	2.04E-01
rs4526799	Brain_Frontal_Cortex_BA9	12	*DTX3*	58000996	rs10876951	0.9085	57306430	T	G	0.35	1.15E-02	1.54E-02	1.81E-01
rs4526799	Brain_Frontal_Cortex_BA9	12	*IKZF4*	56416831	rs10747774	0.9128	57307079	T	C	0.36	1.16E-02	2.99E-02	1.93E-01
rs4526799	Brain_Frontal_Cortex_BA9	12	*RP11-977G19.11*	56701259	rs10747774	0.9128	57307079	T	C	0.36	1.16E-02	2.75E-02	1.91E-01
rs4526799	Brain_Frontal_Cortex_BA9	12	*NXPH4*	57615405	rs10747774	0.9128	57307079	T	C	0.36	1.16E-02	2.81E-02	1.91E-01
rs4526799	Brain_Frontal_Cortex_BA9	12	*DTX3*	58000996	rs10747774	0.9128	57307079	T	C	0.36	1.16E-02	1.92E-02	1.82E-01
rs4526799	Brain_Frontal_Cortex_BA9	12	*IKZF4*	56416831	rs10783812	0.9085	57308723	C	T	0.35	1.19E-02	4.29E-02	2.12E-01
rs4526799	Brain_Frontal_Cortex_BA9	12	*RP11-977G19.11*	56701259	rs10783812	0.9085	57308723	C	T	0.35	1.19E-02	2.76E-02	1.98E-01
rs4526799	Brain_Frontal_Cortex_BA9	12	*TAC3*	57413225	rs10783812	0.9085	57308723	C	T	0.35	1.19E-02	2.80E-02	1.98E-01
rs4526799	Brain_Frontal_Cortex_BA9	12	*NXPH4*	57615405	rs10783812	0.9085	57308723	C	T	0.35	1.19E-02	3.71E-02	2.07E-01
rs4526799	Brain_Frontal_Cortex_BA9	12	*DTX3*	58000996	rs10783812	0.9085	57308723	C	T	0.35	1.19E-02	1.54E-02	1.84E-01
rs4526799	Brain_Frontal_Cortex_BA9	12	*IKZF4*	56416831	rs11172056	0.9089	57308975	C	T	0.37	1.10E-02	2.99E-02	1.88E-01
rs4526799	Brain_Frontal_Cortex_BA9	12	*RP11-977G19.11*	56701259	rs11172056	0.9089	57308975	C	T	0.37	1.10E-02	2.75E-02	1.86E-01
rs4526799	Brain_Frontal_Cortex_BA9	12	*NXPH4*	57615405	rs11172056	0.9089	57308975	C	T	0.37	1.10E-02	2.81E-02	1.86E-01
rs4526799	Brain_Frontal_Cortex_BA9	12	*DTX3*	58000996	rs11172056	0.9089	57308975	C	T	0.37	1.10E-02	1.92E-02	1.77E-01
rs4526799	Brain_Frontal_Cortex_BA9	12	*IKZF4*	56416831	rs7302420	0.9085	57309884	G	C	0.36	1.54E-02	4.37E-02	2.27E-01
rs4526799	Brain_Frontal_Cortex_BA9	12	*RP11-977G19.11*	56701259	rs7302420	0.9085	57309884	G	C	0.36	1.54E-02	2.29E-02	2.09E-01
rs4526799	Brain_Frontal_Cortex_BA9	12	*TAC3*	57413225	rs7302420	0.9085	57309884	G	C	0.36	1.54E-02	4.40E-02	2.28E-01
rs4526799	Brain_Frontal_Cortex_BA9	12	*NXPH4*	57615405	rs7302420	0.9085	57309884	G	C	0.36	1.54E-02	1.32E-02	1.98E-01
rs4526799	Brain_Frontal_Cortex_BA9	12	*DTX3*	58000996	rs7302420	0.9085	57309884	G	C	0.36	1.54E-02	2.50E-02	2.11E-01
rs4526799	Brain_Frontal_Cortex_BA9	12	*RP11-977G19.11*	56701259	rs12228618	0.9128	57311229	T	C	0.37	1.29E-02	3.39E-02	2.00E-01
rs4526799	Brain_Frontal_Cortex_BA9	12	*DTX3*	58000996	rs12228618	0.9128	57311229	T	C	0.37	1.29E-02	9.43E-03	1.72E-01
rs4526799	Brain_Frontal_Cortex_BA9	12	*IKZF4*	56416831	rs9739473	0.9012	57313335	A	T	0.37	2.65E-02	3.91E-02	2.71E-01
rs4526799	Brain_Frontal_Cortex_BA9	12	*RP11-977G19.11*	56701259	rs9739473	0.9012	57313335	A	T	0.37	2.65E-02	2.25E-02	2.58E-01
rs4526799	Brain_Frontal_Cortex_BA9	12	*NXPH4*	57615405	rs9739473	0.9012	57313335	A	T	0.37	2.65E-02	3.44E-02	2.67E-01
rs4526799	Brain_Frontal_Cortex_BA9	12	*DTX3*	58000996	rs9739473	0.9012	57313335	A	T	0.37	2.65E-02	1.71E-02	2.53E-01

rs4526799	Brain_Hippocampus	12	*RP11-603J24.5*	56518610	rs9919772	0.8450	57260027	T	C	0.33	1.03E-02	3.83E-02	2.26E-01
rs4526799	Brain_Hippocampus	12	*RP11-977G19.5*	56570105	rs9919772	0.8450	57260027	T	C	0.33	1.03E-02	1.82E-02	2.08E-01
rs4526799	Brain_Hippocampus	12	*RNF41*	56607001	rs9919772	0.8450	57260027	T	C	0.33	1.03E-02	9.09E-03	1.96E-01
rs4526799	Brain_Hippocampus	12	*GLS2*	56873481	rs9919772	0.8450	57260027	T	C	0.33	1.03E-02	2.02E-02	2.10E-01
rs4526799	Brain_Hippocampus	12	*RBMS2*	56950229	rs9919772	0.8450	57260027	T	C	0.33	1.03E-02	1.61E-02	2.05E-01
rs4526799	Brain_Hippocampus	12	*TMEM194A*	57465636	rs9919772	0.8450	57260027	T	C	0.33	1.03E-02	2.54E-02	2.15E-01
rs4526799	Brain_Hippocampus	12	*DCTN2*	57932523	rs9919772	0.8450	57260027	T	C	0.33	1.03E-02	3.56E-02	2.24E-01
rs4526799	Brain_Hippocampus	12	*METTL1*	58164318	rs9919772	0.8450	57260027	T	C	0.33	1.03E-02	2.21E-02	2.12E-01
rs4526799	Brain_Hippocampus	12	*RP11-603J24.5*	56518610	rs4495925	0.8650	57268116	C	G	0.33	1.04E-02	4.79E-02	2.31E-01
rs4526799	Brain_Hippocampus	12	*RP11-977G19.5*	56570105	rs4495925	0.8650	57268116	C	G	0.33	1.04E-02	9.22E-03	1.93E-01
rs4526799	Brain_Hippocampus	12	*RNF41*	56607001	rs4495925	0.8650	57268116	C	G	0.33	1.04E-02	7.01E-03	1.89E-01
rs4526799	Brain_Hippocampus	12	*SPRYD4*	56863518	rs4495925	0.8650	57268116	C	G	0.33	1.04E-02	4.57E-02	2.30E-01
rs4526799	Brain_Hippocampus	12	*GLS2*	56873481	rs4495925	0.8650	57268116	C	G	0.33	1.04E-02	2.37E-02	2.11E-01
rs4526799	Brain_Hippocampus	12	*RBMS2*	56950229	rs4495925	0.8650	57268116	C	G	0.33	1.04E-02	1.82E-02	2.05E-01
rs4526799	Brain_Hippocampus	12	*METTL1*	58164318	rs4495925	0.8650	57268116	C	G	0.33	1.04E-02	2.18E-02	2.09E-01
rs4526799	Brain_Hippocampus	12	*RP11-603J24.5*	56518610	rs4471472	0.8650	57268985	A	G	0.32	9.99E-03	4.79E-02	2.28E-01
rs4526799	Brain_Hippocampus	12	*RP11-977G19.5*	56570105	rs4471472	0.8650	57268985	A	G	0.32	9.99E-03	9.22E-03	1.89E-01
rs4526799	Brain_Hippocampus	12	*RNF41*	56607001	rs4471472	0.8650	57268985	A	G	0.32	9.99E-03	7.01E-03	1.85E-01
rs4526799	Brain_Hippocampus	12	*SPRYD4*	56863518	rs4471472	0.8650	57268985	A	G	0.32	9.99E-03	4.57E-02	2.26E-01
rs4526799	Brain_Hippocampus	12	*GLS2*	56873481	rs4471472	0.8650	57268985	A	G	0.32	9.99E-03	2.37E-02	2.07E-01
rs4526799	Brain_Hippocampus	12	*RBMS2*	56950229	rs4471472	0.8650	57268985	A	G	0.32	9.99E-03	1.82E-02	2.01E-01
rs4526799	Brain_Hippocampus	12	*METTL1*	58164318	rs4471472	0.8650	57268985	A	G	0.32	9.99E-03	2.18E-02	2.05E-01
rs4526799	Brain_Hippocampus	12	*RP11-977G19.5*	56570105	rs4633499	0.8610	57269264	A	T	0.32	1.43E-02	1.56E-02	2.34E-01
rs4526799	Brain_Hippocampus	12	*RNF41*	56607001	rs4633499	0.8610	57269264	A	T	0.32	1.43E-02	6.29E-03	2.21E-01
rs4526799	Brain_Hippocampus	12	*SPRYD4*	56863518	rs4633499	0.8610	57269264	A	T	0.32	1.43E-02	2.77E-02	2.45E-01
rs4526799	Brain_Hippocampus	12	*GLS2*	56873481	rs4633499	0.8610	57269264	A	T	0.32	1.43E-02	4.26E-02	2.57E-01
rs4526799	Brain_Hippocampus	12	*RBMS2*	56950229	rs4633499	0.8610	57269264	A	T	0.32	1.43E-02	3.20E-02	2.49E-01
rs4526799	Brain_Hippocampus	12	*METTL1*	58164318	rs4633499	0.8610	57269264	A	T	0.32	1.43E-02	2.19E-02	2.40E-01
rs4526799	Brain_Hippocampus	12	*RAB5B*	56378093	rs12300079	0.9956	57273194	T	C	0.36	3.73E-03	2.48E-02	1.59E-01
rs4526799	Brain_Hippocampus	12	*RNF41*	56607001	rs12300079	0.9956	57273194	T	C	0.36	3.73E-03	2.32E-02	1.57E-01
rs4526799	Brain_Hippocampus	12	*ANKRD52*	56641903	rs12300079	0.9956	57273194	T	C	0.36	3.73E-03	3.06E-02	1.65E-01
rs4526799	Brain_Hippocampus	12	*ZBTB39*	57396424	rs12300079	0.9956	57273194	T	C	0.36	3.73E-03	4.04E-02	1.75E-01
rs4526799	Brain_Hippocampus	12	*METTL1*	58164318	rs12300079	0.9956	57273194	T	C	0.36	3.73E-03	8.61E-03	1.36E-01
rs4526799	Brain_Hippocampus	12	*RAB5B*	56378093	rs12300191	0.9956	57273289	A	G	0.36	3.59E-03	2.48E-02	1.57E-01
rs4526799	Brain_Hippocampus	12	*RNF41*	56607001	rs12300191	0.9956	57273289	A	G	0.36	3.59E-03	2.32E-02	1.55E-01
rs4526799	Brain_Hippocampus	12	*ANKRD52*	56641903	rs12300191	0.9956	57273289	A	G	0.36	3.59E-03	3.06E-02	1.63E-01
rs4526799	Brain_Hippocampus	12	*ZBTB39*	57396424	rs12300191	0.9956	57273289	A	G	0.36	3.59E-03	4.04E-02	1.73E-01
rs4526799	Brain_Hippocampus	12	*METTL1*	58164318	rs12300191	0.9956	57273289	A	G	0.36	3.59E-03	8.61E-03	1.34E-01
rs4526799	Brain_Hippocampus	12	*RAB5B*	56378093	rs4514464	0.9956	57276375	C	T	0.36	2.92E-03	2.48E-02	1.53E-01
rs4526799	Brain_Hippocampus	12	*RNF41*	56607001	rs4514464	0.9956	57276375	C	T	0.36	2.92E-03	2.32E-02	1.51E-01

rs4526799	Brain_Hippocampus	12	*ANKRD52*	56641903	rs4514464	0.9956	57276375	C	T	0.36	2.92E-03	3.06E-02	1.60E-01
rs4526799	Brain_Hippocampus	12	*ZBTB39*	57396424	rs4514464	0.9956	57276375	C	T	0.36	2.92E-03	4.04E-02	1.70E-01
rs4526799	Brain_Hippocampus	12	*METTL1*	58164318	rs4514464	0.9956	57276375	C	T	0.36	2.92E-03	8.61E-03	1.30E-01
rs4526799	Brain_Hippocampus	12	*RAB5B*	56378093	rs4417325	0.9956	57277302	G	A	0.36	3.59E-03	2.48E-02	1.57E-01
rs4526799	Brain_Hippocampus	12	*RNF41*	56607001	rs4417325	0.9956	57277302	G	A	0.36	3.59E-03	2.32E-02	1.55E-01
rs4526799	Brain_Hippocampus	12	*ANKRD52*	56641903	rs4417325	0.9956	57277302	G	A	0.36	3.59E-03	3.06E-02	1.63E-01
rs4526799	Brain_Hippocampus	12	*ZBTB39*	57396424	rs4417325	0.9956	57277302	G	A	0.36	3.59E-03	4.04E-02	1.73E-01
rs4526799	Brain_Hippocampus	12	*METTL1*	58164318	rs4417325	0.9956	57277302	G	A	0.36	3.59E-03	8.61E-03	1.34E-01
rs4526799	Brain_Hippocampus	12	*RAB5B*	56378093	rs11172030	0.9956	57278076	A	C	0.36	3.63E-03	2.48E-02	1.57E-01
rs4526799	Brain_Hippocampus	12	*RNF41*	56607001	rs11172030	0.9956	57278076	A	C	0.36	3.63E-03	2.32E-02	1.55E-01
rs4526799	Brain_Hippocampus	12	*ANKRD52*	56641903	rs11172030	0.9956	57278076	A	C	0.36	3.63E-03	3.06E-02	1.63E-01
rs4526799	Brain_Hippocampus	12	*ZBTB39*	57396424	rs11172030	0.9956	57278076	A	C	0.36	3.63E-03	4.04E-02	1.73E-01
rs4526799	Brain_Hippocampus	12	*METTL1*	58164318	rs11172030	0.9956	57278076	A	C	0.36	3.63E-03	8.61E-03	1.34E-01
rs4526799	Brain_Hippocampus	12	*RAB5B*	56378093	rs10876944	0.9956	57279372	T	A	0.36	3.63E-03	2.48E-02	1.57E-01
rs4526799	Brain_Hippocampus	12	*RNF41*	56607001	rs10876944	0.9956	57279372	T	A	0.36	3.63E-03	2.32E-02	1.55E-01
rs4526799	Brain_Hippocampus	12	*ANKRD52*	56641903	rs10876944	0.9956	57279372	T	A	0.36	3.63E-03	3.06E-02	1.63E-01
rs4526799	Brain_Hippocampus	12	*ZBTB39*	57396424	rs10876944	0.9956	57279372	T	A	0.36	3.63E-03	4.04E-02	1.73E-01
rs4526799	Brain_Hippocampus	12	*METTL1*	58164318	rs10876944	0.9956	57279372	T	A	0.36	3.63E-03	8.61E-03	1.34E-01
rs4526799	Brain_Hippocampus	12	*RAB5B*	56378093	rs4326839	0.9956	57280374	G	C	0.36	3.58E-03	2.48E-02	1.56E-01
rs4526799	Brain_Hippocampus	12	*RNF41*	56607001	rs4326839	0.9956	57280374	G	C	0.36	3.58E-03	2.32E-02	1.54E-01
rs4526799	Brain_Hippocampus	12	*ANKRD52*	56641903	rs4326839	0.9956	57280374	G	C	0.36	3.58E-03	3.06E-02	1.63E-01
rs4526799	Brain_Hippocampus	12	*ZBTB39*	57396424	rs4326839	0.9956	57280374	G	C	0.36	3.58E-03	4.04E-02	1.72E-01
rs4526799	Brain_Hippocampus	12	*METTL1*	58164318	rs4326839	0.9956	57280374	G	C	0.36	3.58E-03	8.61E-03	1.33E-01
rs4526799	Brain_Hippocampus	12	*RAB5B*	56378093	rs4526799	1.0000	57280586	T	C	0.34	7.26E-06	2.48E-02	5.78E-02
rs4526799	Brain_Hippocampus	12	*RNF41*	56607001	rs4526799	1.0000	57280586	T	C	0.34	7.26E-06	2.32E-02	5.58E-02
rs4526799	Brain_Hippocampus	12	*ANKRD52*	56641903	rs4526799	1.0000	57280586	T	C	0.34	7.26E-06	3.06E-02	6.47E-02
rs4526799	Brain_Hippocampus	12	*ZBTB39*	57396424	rs4526799	1.0000	57280586	T	C	0.34	7.26E-06	4.04E-02	7.58E-02
rs4526799	Brain_Hippocampus	12	*METTL1*	58164318	rs4526799	1.0000	57280586	T	C	0.34	7.26E-06	8.61E-03	3.47E-02
rs4526799	Brain_Hippocampus	12	*RAB5B*	56378093	rs28876529	0.9956	57285301	T	A	0.36	2.25E-03	2.48E-02	1.41E-01
rs4526799	Brain_Hippocampus	12	*RNF41*	56607001	rs28876529	0.9956	57285301	T	A	0.36	2.25E-03	2.32E-02	1.39E-01
rs4526799	Brain_Hippocampus	12	*ANKRD52*	56641903	rs28876529	0.9956	57285301	T	A	0.36	2.25E-03	3.06E-02	1.48E-01
rs4526799	Brain_Hippocampus	12	*ZBTB39*	57396424	rs28876529	0.9956	57285301	T	A	0.36	2.25E-03	4.04E-02	1.58E-01
rs4526799	Brain_Hippocampus	12	*METTL1*	58164318	rs28876529	0.9956	57285301	T	A	0.36	2.25E-03	8.61E-03	1.17E-01
rs4526799	Brain_Hippocampus	12	*RAB5B*	56378093	rs11172037	0.9956	57285427	T	A	0.36	2.25E-03	2.48E-02	1.41E-01
rs4526799	Brain_Hippocampus	12	*RNF41*	56607001	rs11172037	0.9956	57285427	T	A	0.36	2.25E-03	2.32E-02	1.39E-01
rs4526799	Brain_Hippocampus	12	*ANKRD52*	56641903	rs11172037	0.9956	57285427	T	A	0.36	2.25E-03	3.06E-02	1.48E-01
rs4526799	Brain_Hippocampus	12	*ZBTB39*	57396424	rs11172037	0.9956	57285427	T	A	0.36	2.25E-03	4.04E-02	1.58E-01
rs4526799	Brain_Hippocampus	12	*METTL1*	58164318	rs11172037	0.9956	57285427	T	A	0.36	2.25E-03	8.61E-03	1.17E-01
rs4526799	Brain_Hippocampus	12	*RAB5B*	56378093	rs12321987	0.9956	57288449	G	A	0.36	4.47E-03	2.48E-02	1.78E-01
rs4526799	Brain_Hippocampus	12	*RNF41*	56607001	rs12321987	0.9956	57288449	G	A	0.36	4.47E-03	2.32E-02	1.76E-01

rs4526799	Brain_Hippocampus	12	*ANKRD52*	56641903	rs12321987	0.9956	57288449	G	A	0.36	4.47E-03	3.06E-02	1.84E-01
rs4526799	Brain_Hippocampus	12	*ZBTB39*	57396424	rs12321987	0.9956	57288449	G	A	0.36	4.47E-03	4.04E-02	1.93E-01
rs4526799	Brain_Hippocampus	12	*METTL1*	58164318	rs12321987	0.9956	57288449	G	A	0.36	4.47E-03	8.61E-03	1.56E-01
rs4526799	Brain_Hippocampus	12	*RNF41*	56607001	rs11172043	0.9869	57293182	G	A	0.35	4.58E-03	3.36E-02	1.86E-01
rs4526799	Brain_Hippocampus	12	*METTL1*	58164318	rs11172043	0.9869	57293182	G	A	0.35	4.58E-03	2.59E-03	1.40E-01
rs4526799	Brain_Hippocampus	12	*RNF41*	56607001	rs12426816	0.9869	57294074	A	C	0.35	4.50E-03	3.36E-02	1.85E-01
rs4526799	Brain_Hippocampus	12	*METTL1*	58164318	rs12426816	0.9869	57294074	A	C	0.35	4.50E-03	2.59E-03	1.40E-01
rs4526799	Brain_Hippocampus	12	*RNF41*	56607001	rs11172047	0.9869	57298080	T	C	0.35	6.51E-03	3.36E-02	1.99E-01
rs4526799	Brain_Hippocampus	12	*METTL1*	58164318	rs11172047	0.9869	57298080	T	C	0.35	6.51E-03	2.59E-03	1.55E-01
rs4526799	Brain_Hippocampus	12	*RNF41*	56607001	rs2371631	0.9869	57298614	T	A	0.35	6.32E-03	3.36E-02	1.96E-01
rs4526799	Brain_Hippocampus	12	*METTL1*	58164318	rs2371631	0.9869	57298614	T	A	0.35	6.32E-03	2.59E-03	1.52E-01
rs4526799	Brain_Hippocampus	12	*RNF41*	56607001	rs12305763	0.9869	57299263	G	A	0.35	6.82E-03	3.36E-02	2.00E-01
rs4526799	Brain_Hippocampus	12	*METTL1*	58164318	rs12305763	0.9869	57299263	G	A	0.35	6.82E-03	2.59E-03	1.57E-01
rs4526799	Brain_Hippocampus	12	*RPL41*	56511048	rs11172049	0.9128	57304203	T	C	0.35	1.15E-02	3.78E-02	2.04E-01
rs4526799	Brain_Hippocampus	12	*RNF41*	56607001	rs11172049	0.9128	57304203	T	C	0.35	1.15E-02	4.35E-02	2.09E-01
rs4526799	Brain_Hippocampus	12	*ANKRD52*	56641903	rs11172049	0.9128	57304203	T	C	0.35	1.15E-02	3.82E-02	2.05E-01
rs4526799	Brain_Hippocampus	12	*SPRYD4*	56863518	rs11172049	0.9128	57304203	T	C	0.35	1.15E-02	4.57E-02	2.11E-01
rs4526799	Brain_Hippocampus	12	*METTL1*	58164318	rs11172049	0.9128	57304203	T	C	0.35	1.15E-02	7.23E-03	1.69E-01
rs4526799	Brain_Hippocampus	12	*CTDSP2*	58227116	rs11172049	0.9128	57304203	T	C	0.35	1.15E-02	2.59E-02	1.93E-01
rs4526799	Brain_Hippocampus	12	*RPL41*	56511048	rs1874888	0.9085	57305138	A	C	0.35	1.15E-02	3.78E-02	2.04E-01
rs4526799	Brain_Hippocampus	12	*RNF41*	56607001	rs1874888	0.9085	57305138	A	C	0.35	1.15E-02	4.35E-02	2.09E-01
rs4526799	Brain_Hippocampus	12	*ANKRD52*	56641903	rs1874888	0.9085	57305138	A	C	0.35	1.15E-02	3.82E-02	2.05E-01
rs4526799	Brain_Hippocampus	12	*SPRYD4*	56863518	rs1874888	0.9085	57305138	A	C	0.35	1.15E-02	4.57E-02	2.11E-01
rs4526799	Brain_Hippocampus	12	*METTL1*	58164318	rs1874888	0.9085	57305138	A	C	0.35	1.15E-02	7.23E-03	1.69E-01
rs4526799	Brain_Hippocampus	12	*CTDSP2*	58227116	rs1874888	0.9085	57305138	A	C	0.35	1.15E-02	2.59E-02	1.93E-01
rs4526799	Brain_Hippocampus	12	*RAB5B*	56378093	rs10506349	0.9128	57306412	T	C	0.37	1.10E-02	2.06E-02	1.78E-01
rs4526799	Brain_Hippocampus	12	*RPL41*	56511048	rs10506349	0.9128	57306412	T	C	0.37	1.10E-02	6.03E-03	1.56E-01
rs4526799	Brain_Hippocampus	12	*ANKRD52*	56641903	rs10506349	0.9128	57306412	T	C	0.37	1.10E-02	1.59E-02	1.72E-01
rs4526799	Brain_Hippocampus	12	*ZBTB39*	57396424	rs10506349	0.9128	57306412	T	C	0.37	1.10E-02	3.31E-02	1.91E-01
rs4526799	Brain_Hippocampus	12	*MYO1A*	57433641	rs10506349	0.9128	57306412	T	C	0.37	1.10E-02	4.00E-02	1.97E-01
rs4526799	Brain_Hippocampus	12	*PIP4K2C*	57991077	rs10506349	0.9128	57306412	T	C	0.37	1.10E-02	1.63E-02	1.73E-01
rs4526799	Brain_Hippocampus	12	*METTL1*	58164318	rs10506349	0.9128	57306412	T	C	0.37	1.10E-02	1.46E-02	1.71E-01
rs4526799	Brain_Hippocampus	12	*RPL41*	56511048	rs10876951	0.9085	57306430	T	G	0.35	1.15E-02	3.78E-02	2.04E-01
rs4526799	Brain_Hippocampus	12	*RNF41*	56607001	rs10876951	0.9085	57306430	T	G	0.35	1.15E-02	4.35E-02	2.09E-01
rs4526799	Brain_Hippocampus	12	*ANKRD52*	56641903	rs10876951	0.9085	57306430	T	G	0.35	1.15E-02	3.82E-02	2.05E-01
rs4526799	Brain_Hippocampus	12	*SPRYD4*	56863518	rs10876951	0.9085	57306430	T	G	0.35	1.15E-02	4.57E-02	2.11E-01
rs4526799	Brain_Hippocampus	12	*METTL1*	58164318	rs10876951	0.9085	57306430	T	G	0.35	1.15E-02	7.23E-03	1.69E-01
rs4526799	Brain_Hippocampus	12	*CTDSP2*	58227116	rs10876951	0.9085	57306430	T	G	0.35	1.15E-02	2.59E-02	1.93E-01
rs4526799	Brain_Hippocampus	12	*RAB5B*	56378093	rs10747774	0.9128	57307079	T	C	0.36	1.16E-02	2.06E-02	1.83E-01
rs4526799	Brain_Hippocampus	12	*RPL41*	56511048	rs10747774	0.9128	57307079	T	C	0.36	1.16E-02	5.70E-03	1.61E-01

rs4526799	Brain_Hippocampus	12	*ANKRD52*	56641903	rs10747774	0.9128	57307079	T	C	0.36	1.16E-02	1.53E-02	1.77E-01
rs4526799	Brain_Hippocampus	12	*ZBTB39*	57396424	rs10747774	0.9128	57307079	T	C	0.36	1.16E-02	3.08E-02	1.94E-01
rs4526799	Brain_Hippocampus	12	*MYO1A*	57433641	rs10747774	0.9128	57307079	T	C	0.36	1.16E-02	3.44E-02	1.97E-01
rs4526799	Brain_Hippocampus	12	*PIP4K2C*	57991077	rs10747774	0.9128	57307079	T	C	0.36	1.16E-02	1.52E-02	1.77E-01
rs4526799	Brain_Hippocampus	12	*METTL1*	58164318	rs10747774	0.9128	57307079	T	C	0.36	1.16E-02	1.57E-02	1.77E-01
rs4526799	Brain_Hippocampus	12	*RPL41*	56511048	rs10783812	0.9085	57308723	C	T	0.35	1.19E-02	3.78E-02	2.07E-01
rs4526799	Brain_Hippocampus	12	*RNF41*	56607001	rs10783812	0.9085	57308723	C	T	0.35	1.19E-02	4.35E-02	2.12E-01
rs4526799	Brain_Hippocampus	12	*ANKRD52*	56641903	rs10783812	0.9085	57308723	C	T	0.35	1.19E-02	3.82E-02	2.08E-01
rs4526799	Brain_Hippocampus	12	*SPRYD4*	56863518	rs10783812	0.9085	57308723	C	T	0.35	1.19E-02	4.57E-02	2.14E-01
rs4526799	Brain_Hippocampus	12	*METTL1*	58164318	rs10783812	0.9085	57308723	C	T	0.35	1.19E-02	7.23E-03	1.72E-01
rs4526799	Brain_Hippocampus	12	*CTDSP2*	58227116	rs10783812	0.9085	57308723	C	T	0.35	1.19E-02	2.59E-02	1.96E-01
rs4526799	Brain_Hippocampus	12	*RAB5B*	56378093	rs11172056	0.9089	57308975	C	T	0.37	1.10E-02	2.06E-02	1.78E-01
rs4526799	Brain_Hippocampus	12	*RPL41*	56511048	rs11172056	0.9089	57308975	C	T	0.37	1.10E-02	5.70E-03	1.56E-01
rs4526799	Brain_Hippocampus	12	*ANKRD52*	56641903	rs11172056	0.9089	57308975	C	T	0.37	1.10E-02	1.53E-02	1.72E-01
rs4526799	Brain_Hippocampus	12	*ZBTB39*	57396424	rs11172056	0.9089	57308975	C	T	0.37	1.10E-02	3.08E-02	1.89E-01
rs4526799	Brain_Hippocampus	12	*MYO1A*	57433641	rs11172056	0.9089	57308975	C	T	0.37	1.10E-02	3.44E-02	1.93E-01
rs4526799	Brain_Hippocampus	12	*PIP4K2C*	57991077	rs11172056	0.9089	57308975	C	T	0.37	1.10E-02	1.52E-02	1.72E-01
rs4526799	Brain_Hippocampus	12	*METTL1*	58164318	rs11172056	0.9089	57308975	C	T	0.37	1.10E-02	1.57E-02	1.72E-01
rs4526799	Brain_Hippocampus	12	*RAB5B*	56378093	rs7302420	0.9085	57309884	G	C	0.36	1.54E-02	3.96E-02	2.24E-01
rs4526799	Brain_Hippocampus	12	*RPL41*	56511048	rs7302420	0.9085	57309884	G	C	0.36	1.54E-02	1.57E-02	2.01E-01
rs4526799	Brain_Hippocampus	12	*RNF41*	56607001	rs7302420	0.9085	57309884	G	C	0.36	1.54E-02	3.78E-02	2.23E-01
rs4526799	Brain_Hippocampus	12	*ANKRD52*	56641903	rs7302420	0.9085	57309884	G	C	0.36	1.54E-02	1.71E-02	2.03E-01
rs4526799	Brain_Hippocampus	12	*GLS2*	56873481	rs7302420	0.9085	57309884	G	C	0.36	1.54E-02	2.83E-02	2.14E-01
rs4526799	Brain_Hippocampus	12	*MYO1A*	57433641	rs7302420	0.9085	57309884	G	C	0.36	1.54E-02	3.08E-02	2.17E-01
rs4526799	Brain_Hippocampus	12	*PIP4K2C*	57991077	rs7302420	0.9085	57309884	G	C	0.36	1.54E-02	3.88E-02	2.23E-01
rs4526799	Brain_Hippocampus	12	*METTL1*	58164318	rs7302420	0.9085	57309884	G	C	0.36	1.54E-02	1.14E-02	1.96E-01
rs4526799	Brain_Hippocampus	12	*CTDSP2*	58227116	rs7302420	0.9085	57309884	G	C	0.36	1.54E-02	3.52E-02	2.20E-01
rs4526799	Brain_Hippocampus	12	*RAB5B*	56378093	rs12228618	0.9128	57311229	T	C	0.37	1.29E-02	3.02E-02	1.97E-01
rs4526799	Brain_Hippocampus	12	*RPL41*	56511048	rs12228618	0.9128	57311229	T	C	0.37	1.29E-02	2.31E-02	1.90E-01
rs4526799	Brain_Hippocampus	12	*RNF41*	56607001	rs12228618	0.9128	57311229	T	C	0.37	1.29E-02	4.50E-02	2.10E-01
rs4526799	Brain_Hippocampus	12	*ANKRD52*	56641903	rs12228618	0.9128	57311229	T	C	0.37	1.29E-02	1.70E-02	1.83E-01
rs4526799	Brain_Hippocampus	12	*ZBTB39*	57396424	rs12228618	0.9128	57311229	T	C	0.37	1.29E-02	1.74E-02	1.83E-01
rs4526799	Brain_Hippocampus	12	*MYO1A*	57433641	rs12228618	0.9128	57311229	T	C	0.37	1.29E-02	2.09E-02	1.87E-01
rs4526799	Brain_Hippocampus	12	*PIP4K2C*	57991077	rs12228618	0.9128	57311229	T	C	0.37	1.29E-02	1.63E-02	1.82E-01
rs4526799	Brain_Hippocampus	12	*RAB5B*	56378093	rs9739473	0.9012	57313335	A	T	0.37	2.65E-02	1.73E-02	2.53E-01
rs4526799	Brain_Hippocampus	12	*RPL41*	56511048	rs9739473	0.9012	57313335	A	T	0.37	2.65E-02	4.04E-03	2.35E-01
rs4526799	Brain_Hippocampus	12	*ANKRD52*	56641903	rs9739473	0.9012	57313335	A	T	0.37	2.65E-02	1.02E-02	2.45E-01
rs4526799	Brain_Hippocampus	12	*RBMS2*	56950229	rs9739473	0.9012	57313335	A	T	0.37	2.65E-02	3.89E-02	2.70E-01
rs4526799	Brain_Hippocampus	12	*ZBTB39*	57396424	rs9739473	0.9012	57313335	A	T	0.37	2.65E-02	1.24E-02	2.48E-01
rs4526799	Brain_Hippocampus	12	*MYO1A*	57433641	rs9739473	0.9012	57313335	A	T	0.37	2.65E-02	2.51E-02	2.60E-01

rs4526799	Brain_Hippocampus	12	*PIP4K2C*	57991077	rs9739473	0.9012	57313335	A	T	0.37	2.65E-02	1.33E-02	2.49E-01
rs4526799	Brain_Hippocampus	12	*METTL1*	58164318	rs9739473	0.9012	57313335	A	T	0.37	2.65E-02	4.68E-02	2.76E-01
rs4526799	Brain_Hypothalamus	12	*RP11-977G19.11*	56701259	rs9919772	0.8450	57260027	T	C	0.33	1.03E-02	1.01E-02	1.97E-01
rs4526799	Brain_Hypothalamus	12	*IL23A*	56733428	rs9919772	0.8450	57260027	T	C	0.33	1.03E-02	1.42E-02	2.03E-01
rs4526799	Brain_Hypothalamus	12	*STAT2*	56744645	rs9919772	0.8450	57260027	T	C	0.33	1.03E-02	4.76E-02	2.34E-01
rs4526799	Brain_Hypothalamus	12	*PTGES3*	57069643	rs9919772	0.8450	57260027	T	C	0.33	1.03E-02	1.18E-02	2.00E-01
rs4526799	Brain_Hypothalamus	12	*SLC26A10*	58016493	rs9919772	0.8450	57260027	T	C	0.33	1.03E-02	2.90E-02	2.19E-01
rs4526799	Brain_Hypothalamus	12	*RP11-977G19.11*	56701259	rs4495925	0.8650	57268116	C	G	0.33	1.04E-02	1.26E-02	1.98E-01
rs4526799	Brain_Hypothalamus	12	*IL23A*	56733428	rs4495925	0.8650	57268116	C	G	0.33	1.04E-02	1.37E-02	1.99E-01
rs4526799	Brain_Hypothalamus	12	*STAT2*	56744645	rs4495925	0.8650	57268116	C	G	0.33	1.04E-02	4.19E-02	2.27E-01
rs4526799	Brain_Hypothalamus	12	*PTGES3*	57069643	rs4495925	0.8650	57268116	C	G	0.33	1.04E-02	2.39E-02	2.11E-01
rs4526799	Brain_Hypothalamus	12	*SLC26A10*	58016493	rs4495925	0.8650	57268116	C	G	0.33	1.04E-02	4.09E-02	2.26E-01
rs4526799	Brain_Hypothalamus	12	*RP11-977G19.11*	56701259	rs4471472	0.8650	57268985	A	G	0.32	9.99E-03	1.26E-02	1.94E-01
rs4526799	Brain_Hypothalamus	12	*IL23A*	56733428	rs4471472	0.8650	57268985	A	G	0.32	9.99E-03	1.37E-02	1.95E-01
rs4526799	Brain_Hypothalamus	12	*STAT2*	56744645	rs4471472	0.8650	57268985	A	G	0.32	9.99E-03	4.19E-02	2.23E-01
rs4526799	Brain_Hypothalamus	12	*PTGES3*	57069643	rs4471472	0.8650	57268985	A	G	0.32	9.99E-03	2.39E-02	2.07E-01
rs4526799	Brain_Hypothalamus	12	*SLC26A10*	58016493	rs4471472	0.8650	57268985	A	G	0.32	9.99E-03	4.09E-02	2.22E-01
rs4526799	Brain_Hypothalamus	12	*RP11-977G19.11*	56701259	rs4633499	0.8610	57269264	A	T	0.32	1.43E-02	8.30E-03	2.25E-01
rs4526799	Brain_Hypothalamus	12	*IL23A*	56733428	rs4633499	0.8610	57269264	A	T	0.32	1.43E-02	1.68E-02	2.35E-01
rs4526799	Brain_Hypothalamus	12	*STAT2*	56744645	rs4633499	0.8610	57269264	A	T	0.32	1.43E-02	3.77E-02	2.53E-01
rs4526799	Brain_Hypothalamus	12	*PTGES3*	57069643	rs4633499	0.8610	57269264	A	T	0.32	1.43E-02	3.59E-02	2.52E-01
rs4526799	Brain_Hypothalamus	12	*SLC26A10*	58016493	rs4633499	0.8610	57269264	A	T	0.32	1.43E-02	4.45E-02	2.58E-01
rs4526799	Brain_Hypothalamus	12	*PTGES3*	57069643	rs12300079	0.9956	57273194	T	C	0.36	3.73E-03	1.09E-02	1.40E-01
rs4526799	Brain_Hypothalamus	12	*PTGES3*	57069643	rs12300191	0.9956	57273289	A	G	0.36	3.59E-03	1.05E-02	1.37E-01
rs4526799	Brain_Hypothalamus	12	*PTGES3*	57069643	rs4514464	0.9956	57276375	C	T	0.36	2.92E-03	1.05E-02	1.34E-01
rs4526799	Brain_Hypothalamus	12	*PTGES3*	57069643	rs4417325	0.9956	57277302	G	A	0.36	3.59E-03	1.05E-02	1.37E-01
rs4526799	Brain_Hypothalamus	12	*PTGES3*	57069643	rs11172030	0.9956	57278076	A	C	0.36	3.63E-03	1.05E-02	1.38E-01
rs4526799	Brain_Hypothalamus	12	*PTGES3*	57069643	rs10876944	0.9956	57279372	T	A	0.36	3.63E-03	1.05E-02	1.38E-01
rs4526799	Brain_Hypothalamus	12	*PTGES3*	57069643	rs4326839	0.9956	57280374	G	C	0.36	3.58E-03	1.05E-02	1.37E-01
rs4526799	Brain_Hypothalamus	12	*PTGES3*	57069643	rs4526799	1.0000	57280586	T	C	0.34	7.26E-06	1.05E-02	3.79E-02
rs4526799	Brain_Hypothalamus	12	*PTGES3*	57069643	rs28876529	0.9956	57285301	T	A	0.36	2.25E-03	1.05E-02	1.21E-01
rs4526799	Brain_Hypothalamus	12	*PTGES3*	57069643	rs11172037	0.9956	57285427	T	A	0.36	2.25E-03	1.05E-02	1.21E-01
rs4526799	Brain_Hypothalamus	12	*PTGES3*	57069643	rs12321987	0.9956	57288449	G	A	0.36	4.47E-03	1.05E-02	1.60E-01
rs4526799	Brain_Hypothalamus	12	*PTGES3*	57069643	rs11172043	0.9869	57293182	G	A	0.35	4.58E-03	1.19E-02	1.60E-01
rs4526799	Brain_Hypothalamus	12	*RP11-545N8.3*	57539902	rs11172043	0.9869	57293182	G	A	0.35	4.58E-03	4.60E-02	1.97E-01
rs4526799	Brain_Hypothalamus	12	*OS9*	58101539	rs11172043	0.9869	57293182	G	A	0.35	4.58E-03	1.04E-02	1.58E-01
rs4526799	Brain_Hypothalamus	12	*PTGES3*	57069643	rs12426816	0.9869	57294074	A	C	0.35	4.50E-03	1.19E-02	1.60E-01
rs4526799	Brain_Hypothalamus	12	*RP11-545N8.3*	57539902	rs12426816	0.9869	57294074	A	C	0.35	4.50E-03	4.60E-02	1.97E-01
rs4526799	Brain_Hypothalamus	12	*OS9*	58101539	rs12426816	0.9869	57294074	A	C	0.35	4.50E-03	1.04E-02	1.58E-01
rs4526799	Brain_Hypothalamus	12	*PTGES3*	57069643	rs11172047	0.9869	57298080	T	C	0.35	6.51E-03	1.19E-02	1.75E-01

rs4526799	Brain_Hypothalamus	12	*RP11-545N8.3*	57539902	rs11172047	0.9869	57298080	T	C	0.35	6.51E-03	4.60E-02	2.10E-01
rs4526799	Brain_Hypothalamus	12	*OS9*	58101539	rs11172047	0.9869	57298080	T	C	0.35	6.51E-03	1.04E-02	1.73E-01
rs4526799	Brain_Hypothalamus	12	*PTGES3*	57069643	rs2371631	0.9869	57298614	T	A	0.35	6.32E-03	1.19E-02	1.72E-01
rs4526799	Brain_Hypothalamus	12	*RP11-545N8.3*	57539902	rs2371631	0.9869	57298614	T	A	0.35	6.32E-03	4.60E-02	2.07E-01
rs4526799	Brain_Hypothalamus	12	*OS9*	58101539	rs2371631	0.9869	57298614	T	A	0.35	6.32E-03	1.04E-02	1.69E-01
rs4526799	Brain_Hypothalamus	12	*PTGES3*	57069643	rs12305763	0.9869	57299263	G	A	0.35	6.82E-03	1.19E-02	1.76E-01
rs4526799	Brain_Hypothalamus	12	*RP11-545N8.3*	57539902	rs12305763	0.9869	57299263	G	A	0.35	6.82E-03	4.60E-02	2.11E-01
rs4526799	Brain_Hypothalamus	12	*OS9*	58101539	rs12305763	0.9869	57299263	G	A	0.35	6.82E-03	1.04E-02	1.74E-01
rs4526799	Brain_Hypothalamus	12	*RP11-977G19.11*	56701259	rs11172049	0.9128	57304203	T	C	0.35	1.15E-02	2.25E-02	1.90E-01
rs4526799	Brain_Hypothalamus	12	*PTGES3*	57069643	rs11172049	0.9128	57304203	T	C	0.35	1.15E-02	1.06E-02	1.74E-01
rs4526799	Brain_Hypothalamus	12	*OS9*	58101539	rs11172049	0.9128	57304203	T	C	0.35	1.15E-02	2.77E-02	1.95E-01
rs4526799	Brain_Hypothalamus	12	*RP11-977G19.11*	56701259	rs1874888	0.9085	57305138	A	C	0.35	1.15E-02	2.25E-02	1.90E-01
rs4526799	Brain_Hypothalamus	12	*PTGES3*	57069643	rs1874888	0.9085	57305138	A	C	0.35	1.15E-02	1.06E-02	1.74E-01
rs4526799	Brain_Hypothalamus	12	*OS9*	58101539	rs1874888	0.9085	57305138	A	C	0.35	1.15E-02	2.77E-02	1.95E-01
rs4526799	Brain_Hypothalamus	12	*RP11-977G19.11*	56701259	rs10506349	0.9128	57306412	T	C	0.37	1.10E-02	3.67E-02	1.94E-01
rs4526799	Brain_Hypothalamus	12	*PTGES3*	57069643	rs10506349	0.9128	57306412	T	C	0.37	1.10E-02	1.45E-02	1.71E-01
rs4526799	Brain_Hypothalamus	12	*RP11-545N8.3*	57539902	rs10506349	0.9128	57306412	T	C	0.37	1.10E-02	4.65E-02	2.03E-01
rs4526799	Brain_Hypothalamus	12	*RP11-977G19.11*	56701259	rs10876951	0.9085	57306430	T	G	0.35	1.15E-02	2.25E-02	1.90E-01
rs4526799	Brain_Hypothalamus	12	*PTGES3*	57069643	rs10876951	0.9085	57306430	T	G	0.35	1.15E-02	1.06E-02	1.74E-01
rs4526799	Brain_Hypothalamus	12	*OS9*	58101539	rs10876951	0.9085	57306430	T	G	0.35	1.15E-02	2.77E-02	1.95E-01
rs4526799	Brain_Hypothalamus	12	*RP11-977G19.11*	56701259	rs10747774	0.9128	57307079	T	C	0.36	1.16E-02	3.67E-02	2.00E-01
rs4526799	Brain_Hypothalamus	12	*PTGES3*	57069643	rs10747774	0.9128	57307079	T	C	0.36	1.16E-02	1.64E-02	1.78E-01
rs4526799	Brain_Hypothalamus	12	*RP11-545N8.3*	57539902	rs10747774	0.9128	57307079	T	C	0.36	1.16E-02	4.35E-02	2.05E-01
rs4526799	Brain_Hypothalamus	12	*RP11-977G19.11*	56701259	rs10783812	0.9085	57308723	C	T	0.35	1.19E-02	2.25E-02	1.93E-01
rs4526799	Brain_Hypothalamus	12	*PTGES3*	57069643	rs10783812	0.9085	57308723	C	T	0.35	1.19E-02	1.06E-02	1.78E-01
rs4526799	Brain_Hypothalamus	12	*OS9*	58101539	rs10783812	0.9085	57308723	C	T	0.35	1.19E-02	2.77E-02	1.98E-01
rs4526799	Brain_Hypothalamus	12	*RP11-977G19.11*	56701259	rs11172056	0.9089	57308975	C	T	0.37	1.10E-02	3.67E-02	1.95E-01
rs4526799	Brain_Hypothalamus	12	*PTGES3*	57069643	rs11172056	0.9089	57308975	C	T	0.37	1.10E-02	1.64E-02	1.73E-01
rs4526799	Brain_Hypothalamus	12	*RP11-545N8.3*	57539902	rs11172056	0.9089	57308975	C	T	0.37	1.10E-02	4.35E-02	2.01E-01
rs4526799	Brain_Hypothalamus	12	*RP11-977G19.11*	56701259	rs7302420	0.9085	57309884	G	C	0.36	1.54E-02	3.69E-02	2.22E-01
rs4526799	Brain_Hypothalamus	12	*PTGES3*	57069643	rs7302420	0.9085	57309884	G	C	0.36	1.54E-02	1.76E-02	2.03E-01
rs4526799	Brain_Hypothalamus	12	*RP11-977G19.11*	56701259	rs12228618	0.9128	57311229	T	C	0.37	1.29E-02	2.12E-02	1.88E-01
rs4526799	Brain_Hypothalamus	12	*PTGES3*	57069643	rs12228618	0.9128	57311229	T	C	0.37	1.29E-02	2.91E-02	1.96E-01
rs4526799	Brain_Hypothalamus	12	*SLC26A10*	58016493	rs12228618	0.9128	57311229	T	C	0.37	1.29E-02	2.26E-02	1.89E-01
rs4526799	Brain_Hypothalamus	12	*RP11-977G19.11*	56701259	rs9739473	0.9012	57313335	A	T	0.37	2.65E-02	2.28E-02	2.58E-01
rs4526799	Brain_Hypothalamus	12	*PTGES3*	57069643	rs9739473	0.9012	57313335	A	T	0.37	2.65E-02	2.06E-02	2.56E-01
rs4526799	Brain_Hypothalamus	12	*SLC26A10*	58016493	rs9739473	0.9012	57313335	A	T	0.37	2.65E-02	3.33E-02	2.66E-01
rs4526799	Brain_Nucleus_accumbens_basal_ganglia	12	*PMEL*	56357495	rs9919772	0.8450	57260027	T	C	0.33	1.03E-02	3.72E-02	2.26E-01
rs4526799	Brain_Nucleus_accumbens_basal_ganglia	12	*RP11-603J24.5*	56518610	rs9919772	0.8450	57260027	T	C	0.33	1.03E-02	1.45E-02	2.03E-01
rs4526799	Brain_Nucleus_accumbens_basal_ganglia	12	*RP11-977G19.5*	56570105	rs9919772	0.8450	57260027	T	C	0.33	1.03E-02	3.90E-02	2.27E-01

rs4526799	Brain_Nucleus_accumbens_basal_ganglia	12	*RNF41*	56607001	rs9919772	0.8450	57260027	T	C	0.33	1.03E-02	3.18E-02	2.21E-01
rs4526799	Brain_Nucleus_accumbens_basal_ganglia	12	*PAN2*	56718979	rs9919772	0.8450	57260027	T	C	0.33	1.03E-02	2.71E-02	2.17E-01
rs4526799	Brain_Nucleus_accumbens_basal_ganglia	12	*TMEM194A*	57465636	rs9919772	0.8450	57260027	T	C	0.33	1.03E-02	1.30E-02	2.01E-01
rs4526799	Brain_Nucleus_accumbens_basal_ganglia	12	*NAB2*	57485933	rs9919772	0.8450	57260027	T	C	0.33	1.03E-02	4.15E-02	2.29E-01
rs4526799	Brain_Nucleus_accumbens_basal_ganglia	12	*PMEL*	56357495	rs4495925	0.8650	57268116	C	G	0.33	1.04E-02	2.53E-02	2.12E-01
rs4526799	Brain_Nucleus_accumbens_basal_ganglia	12	*RP11-603J24.5*	56518610	rs4495925	0.8650	57268116	C	G	0.33	1.04E-02	1.55E-02	2.02E-01
rs4526799	Brain_Nucleus_accumbens_basal_ganglia	12	*RNF41*	56607001	rs4495925	0.8650	57268116	C	G	0.33	1.04E-02	3.10E-02	2.17E-01
rs4526799	Brain_Nucleus_accumbens_basal_ganglia	12	*PAN2*	56718979	rs4495925	0.8650	57268116	C	G	0.33	1.04E-02	2.87E-02	2.15E-01
rs4526799	Brain_Nucleus_accumbens_basal_ganglia	12	*TMEM194A*	57465636	rs4495925	0.8650	57268116	C	G	0.33	1.04E-02	1.68E-02	2.03E-01
rs4526799	Brain_Nucleus_accumbens_basal_ganglia	12	*NAB2*	57485933	rs4495925	0.8650	57268116	C	G	0.33	1.04E-02	3.88E-02	2.24E-01
rs4526799	Brain_Nucleus_accumbens_basal_ganglia	12	*PMEL*	56357495	rs4471472	0.8650	57268985	A	G	0.32	9.99E-03	3.26E-02	2.15E-01
rs4526799	Brain_Nucleus_accumbens_basal_ganglia	12	*RP11-603J24.5*	56518610	rs4471472	0.8650	57268985	A	G	0.32	9.99E-03	1.68E-02	1.99E-01
rs4526799	Brain_Nucleus_accumbens_basal_ganglia	12	*RNF41*	56607001	rs4471472	0.8650	57268985	A	G	0.32	9.99E-03	2.82E-02	2.11E-01
rs4526799	Brain_Nucleus_accumbens_basal_ganglia	12	*PAN2*	56718979	rs4471472	0.8650	57268985	A	G	0.32	9.99E-03	3.20E-02	2.15E-01
rs4526799	Brain_Nucleus_accumbens_basal_ganglia	12	*TMEM194A*	57465636	rs4471472	0.8650	57268985	A	G	0.32	9.99E-03	1.51E-02	1.97E-01
rs4526799	Brain_Nucleus_accumbens_basal_ganglia	12	*NAB2*	57485933	rs4471472	0.8650	57268985	A	G	0.32	9.99E-03	4.29E-02	2.24E-01
rs4526799	Brain_Nucleus_accumbens_basal_ganglia	12	*RP11-603J24.5*	56518610	rs4633499	0.8610	57269264	A	T	0.32	1.43E-02	2.77E-02	2.45E-01
rs4526799	Brain_Nucleus_accumbens_basal_ganglia	12	*RP11-977G19.5*	56570105	rs4633499	0.8610	57269264	A	T	0.32	1.43E-02	3.98E-02	2.55E-01
rs4526799	Brain_Nucleus_accumbens_basal_ganglia	12	*PAN2*	56718979	rs4633499	0.8610	57269264	A	T	0.32	1.43E-02	2.14E-02	2.40E-01
rs4526799	Brain_Nucleus_accumbens_basal_ganglia	12	*TMEM194A*	57465636	rs4633499	0.8610	57269264	A	T	0.32	1.43E-02	1.91E-02	2.38E-01
rs4526799	Brain_Nucleus_accumbens_basal_ganglia	12	*B4GALNT1*	58023536	rs4633499	0.8610	57269264	A	T	0.32	1.43E-02	4.62E-02	2.59E-01
rs4526799	Brain_Nucleus_accumbens_basal_ganglia	12	*RNF41*	56607001	rs12300079	0.9956	57273194	T	C	0.36	3.73E-03	4.91E-02	1.83E-01
rs4526799	Brain_Nucleus_accumbens_basal_ganglia	12	*BAZ2A*	57009990	rs12300079	0.9956	57273194	T	C	0.36	3.73E-03	3.20E-02	1.67E-01
rs4526799	Brain_Nucleus_accumbens_basal_ganglia	12	*RNF41*	56607001	rs12300191	0.9956	57273289	A	G	0.36	3.59E-03	4.91E-02	1.81E-01
rs4526799	Brain_Nucleus_accumbens_basal_ganglia	12	*BAZ2A*	57009990	rs12300191	0.9956	57273289	A	G	0.36	3.59E-03	3.20E-02	1.64E-01
rs4526799	Brain_Nucleus_accumbens_basal_ganglia	12	*RNF41*	56607001	rs4514464	0.9956	57276375	C	T	0.36	2.92E-03	4.91E-02	1.78E-01
rs4526799	Brain_Nucleus_accumbens_basal_ganglia	12	*BAZ2A*	57009990	rs4514464	0.9956	57276375	C	T	0.36	2.92E-03	3.20E-02	1.61E-01
rs4526799	Brain_Nucleus_accumbens_basal_ganglia	12	*RNF41*	56607001	rs4417325	0.9956	57277302	G	A	0.36	3.59E-03	4.91E-02	1.81E-01
rs4526799	Brain_Nucleus_accumbens_basal_ganglia	12	*BAZ2A*	57009990	rs4417325	0.9956	57277302	G	A	0.36	3.59E-03	3.20E-02	1.64E-01
rs4526799	Brain_Nucleus_accumbens_basal_ganglia	12	*RNF41*	56607001	rs11172030	0.9956	57278076	A	C	0.36	3.63E-03	4.91E-02	1.81E-01
rs4526799	Brain_Nucleus_accumbens_basal_ganglia	12	*BAZ2A*	57009990	rs11172030	0.9956	57278076	A	C	0.36	3.63E-03	3.20E-02	1.65E-01
rs4526799	Brain_Nucleus_accumbens_basal_ganglia	12	*RNF41*	56607001	rs10876944	0.9956	57279372	T	A	0.36	3.63E-03	4.91E-02	1.81E-01
rs4526799	Brain_Nucleus_accumbens_basal_ganglia	12	*BAZ2A*	57009990	rs10876944	0.9956	57279372	T	A	0.36	3.63E-03	3.20E-02	1.65E-01
rs4526799	Brain_Nucleus_accumbens_basal_ganglia	12	*RNF41*	56607001	rs4326839	0.9956	57280374	G	C	0.36	3.58E-03	4.91E-02	1.81E-01
rs4526799	Brain_Nucleus_accumbens_basal_ganglia	12	*BAZ2A*	57009990	rs4326839	0.9956	57280374	G	C	0.36	3.58E-03	3.20E-02	1.64E-01
rs4526799	Brain_Nucleus_accumbens_basal_ganglia	12	*RNF41*	56607001	rs4526799	1.0000	57280586	T	C	0.34	7.26E-06	4.91E-02	8.53E-02
rs4526799	Brain_Nucleus_accumbens_basal_ganglia	12	*BAZ2A*	57009990	rs4526799	1.0000	57280586	T	C	0.34	7.26E-06	3.20E-02	6.64E-02
rs4526799	Brain_Nucleus_accumbens_basal_ganglia	12	*RNF41*	56607001	rs28876529	0.9956	57285301	T	A	0.36	2.25E-03	4.91E-02	1.66E-01
rs4526799	Brain_Nucleus_accumbens_basal_ganglia	12	*BAZ2A*	57009990	rs28876529	0.9956	57285301	T	A	0.36	2.25E-03	3.20E-02	1.49E-01
rs4526799	Brain_Nucleus_accumbens_basal_ganglia	12	*RNF41*	56607001	rs11172037	0.9956	57285427	T	A	0.36	2.25E-03	4.91E-02	1.66E-01

rs4526799	Brain_Nucleus_accumbens_basal_ganglia	12	*BAZ2A*	57009990	rs11172037	0.9956	57285427	T	A	0.36	2.25E-03	3.20E-02	1.49E-01
rs4526799	Brain_Nucleus_accumbens_basal_ganglia	12	*RNF41*	56607001	rs12321987	0.9956	57288449	G	A	0.36	4.47E-03	4.91E-02	2.01E-01
rs4526799	Brain_Nucleus_accumbens_basal_ganglia	12	*BAZ2A*	57009990	rs12321987	0.9956	57288449	G	A	0.36	4.47E-03	3.20E-02	1.85E-01
rs4526799	Brain_Nucleus_accumbens_basal_ganglia	12	*RP11-977G19.5*	56570105	rs11172043	0.9869	57293182	G	A	0.35	4.58E-03	2.70E-02	1.79E-01
rs4526799	Brain_Nucleus_accumbens_basal_ganglia	12	*BAZ2A*	57009990	rs11172043	0.9869	57293182	G	A	0.35	4.58E-03	1.78E-02	1.68E-01
rs4526799	Brain_Nucleus_accumbens_basal_ganglia	12	*HSD17B6*	57163759	rs11172043	0.9869	57293182	G	A	0.35	4.58E-03	7.34E-03	1.52E-01
rs4526799	Brain_Nucleus_accumbens_basal_ganglia	12	*RP11-977G19.5*	56570105	rs12426816	0.9869	57294074	A	C	0.35	4.50E-03	2.70E-02	1.79E-01
rs4526799	Brain_Nucleus_accumbens_basal_ganglia	12	*BAZ2A*	57009990	rs12426816	0.9869	57294074	A	C	0.35	4.50E-03	1.78E-02	1.68E-01
rs4526799	Brain_Nucleus_accumbens_basal_ganglia	12	*HSD17B6*	57163759	rs12426816	0.9869	57294074	A	C	0.35	4.50E-03	7.34E-03	1.52E-01
rs4526799	Brain_Nucleus_accumbens_basal_ganglia	12	*RP11-977G19.5*	56570105	rs11172047	0.9869	57298080	T	C	0.35	6.51E-03	2.70E-02	1.93E-01
rs4526799	Brain_Nucleus_accumbens_basal_ganglia	12	*BAZ2A*	57009990	rs11172047	0.9869	57298080	T	C	0.35	6.51E-03	1.78E-02	1.83E-01
rs4526799	Brain_Nucleus_accumbens_basal_ganglia	12	*HSD17B6*	57163759	rs11172047	0.9869	57298080	T	C	0.35	6.51E-03	7.34E-03	1.67E-01
rs4526799	Brain_Nucleus_accumbens_basal_ganglia	12	*RP11-977G19.5*	56570105	rs2371631	0.9869	57298614	T	A	0.35	6.32E-03	2.70E-02	1.90E-01
rs4526799	Brain_Nucleus_accumbens_basal_ganglia	12	*BAZ2A*	57009990	rs2371631	0.9869	57298614	T	A	0.35	6.32E-03	1.78E-02	1.79E-01
rs4526799	Brain_Nucleus_accumbens_basal_ganglia	12	*HSD17B6*	57163759	rs2371631	0.9869	57298614	T	A	0.35	6.32E-03	7.34E-03	1.64E-01
rs4526799	Brain_Nucleus_accumbens_basal_ganglia	12	*RP11-977G19.5*	56570105	rs12305763	0.9869	57299263	G	A	0.35	6.82E-03	2.70E-02	1.94E-01
rs4526799	Brain_Nucleus_accumbens_basal_ganglia	12	*BAZ2A*	57009990	rs12305763	0.9869	57299263	G	A	0.35	6.82E-03	1.78E-02	1.84E-01
rs4526799	Brain_Nucleus_accumbens_basal_ganglia	12	*HSD17B6*	57163759	rs12305763	0.9869	57299263	G	A	0.35	6.82E-03	7.34E-03	1.69E-01
rs4526799	Brain_Nucleus_accumbens_basal_ganglia	12	*RP11-603J24.5*	56518610	rs11172049	0.9128	57304203	T	C	0.35	1.15E-02	4.77E-02	2.13E-01
rs4526799	Brain_Nucleus_accumbens_basal_ganglia	12	*RP11-977G19.5*	56570105	rs11172049	0.9128	57304203	T	C	0.35	1.15E-02	3.94E-02	2.06E-01
rs4526799	Brain_Nucleus_accumbens_basal_ganglia	12	*HSD17B6*	57163759	rs11172049	0.9128	57304203	T	C	0.35	1.15E-02	9.23E-03	1.72E-01
rs4526799	Brain_Nucleus_accumbens_basal_ganglia	12	*RP11-603J24.5*	56518610	rs1874888	0.9085	57305138	A	C	0.35	1.15E-02	4.77E-02	2.13E-01
rs4526799	Brain_Nucleus_accumbens_basal_ganglia	12	*RP11-977G19.5*	56570105	rs1874888	0.9085	57305138	A	C	0.35	1.15E-02	3.94E-02	2.06E-01
rs4526799	Brain_Nucleus_accumbens_basal_ganglia	12	*HSD17B6*	57163759	rs1874888	0.9085	57305138	A	C	0.35	1.15E-02	9.23E-03	1.72E-01
rs4526799	Brain_Nucleus_accumbens_basal_ganglia	12	*RP11-977G19.5*	56570105	rs10506349	0.9128	57306412	T	C	0.37	1.10E-02	4.04E-02	1.98E-01
rs4526799	Brain_Nucleus_accumbens_basal_ganglia	12	*NABP2*	56619718	rs10506349	0.9128	57306412	T	C	0.37	1.10E-02	2.01E-02	1.78E-01
rs4526799	Brain_Nucleus_accumbens_basal_ganglia	12	*HSD17B6*	57163759	rs10506349	0.9128	57306412	T	C	0.37	1.10E-02	2.49E-02	1.83E-01
rs4526799	Brain_Nucleus_accumbens_basal_ganglia	12	*DDIT3*	57912369	rs10506349	0.9128	57306412	T	C	0.37	1.10E-02	3.37E-02	1.92E-01
rs4526799	Brain_Nucleus_accumbens_basal_ganglia	12	*B4GALNT1*	58023536	rs10506349	0.9128	57306412	T	C	0.37	1.10E-02	4.84E-03	1.53E-01
rs4526799	Brain_Nucleus_accumbens_basal_ganglia	12	*RP11-603J24.5*	56518610	rs10876951	0.9085	57306430	T	G	0.35	1.15E-02	4.77E-02	2.13E-01
rs4526799	Brain_Nucleus_accumbens_basal_ganglia	12	*RP11-977G19.5*	56570105	rs10876951	0.9085	57306430	T	G	0.35	1.15E-02	3.94E-02	2.06E-01
rs4526799	Brain_Nucleus_accumbens_basal_ganglia	12	*HSD17B6*	57163759	rs10876951	0.9085	57306430	T	G	0.35	1.15E-02	9.23E-03	1.72E-01
rs4526799	Brain_Nucleus_accumbens_basal_ganglia	12	*RP11-977G19.5*	56570105	rs10747774	0.9128	57307079	T	C	0.36	1.16E-02	4.29E-02	2.05E-01
rs4526799	Brain_Nucleus_accumbens_basal_ganglia	12	*NABP2*	56619718	rs10747774	0.9128	57307079	T	C	0.36	1.16E-02	1.79E-02	1.80E-01
rs4526799	Brain_Nucleus_accumbens_basal_ganglia	12	*HSD17B6*	57163759	rs10747774	0.9128	57307079	T	C	0.36	1.16E-02	2.28E-02	1.86E-01
rs4526799	Brain_Nucleus_accumbens_basal_ganglia	12	*DDIT3*	57912369	rs10747774	0.9128	57307079	T	C	0.36	1.16E-02	3.27E-02	1.96E-01
rs4526799	Brain_Nucleus_accumbens_basal_ganglia	12	*B4GALNT1*	58023536	rs10747774	0.9128	57307079	T	C	0.36	1.16E-02	4.85E-03	1.59E-01
rs4526799	Brain_Nucleus_accumbens_basal_ganglia	12	*RP11-603J24.5*	56518610	rs10783812	0.9085	57308723	C	T	0.35	1.19E-02	4.86E-02	2.16E-01
rs4526799	Brain_Nucleus_accumbens_basal_ganglia	12	*RP11-977G19.5*	56570105	rs10783812	0.9085	57308723	C	T	0.35	1.19E-02	4.58E-02	2.14E-01
rs4526799	Brain_Nucleus_accumbens_basal_ganglia	12	*HSD17B6*	57163759	rs10783812	0.9085	57308723	C	T	0.35	1.19E-02	1.10E-02	1.78E-01

rs4526799	Brain_Nucleus_accumbens_basal_ganglia	12	*RP11-977G19.5*	56570105	rs11172056	0.9089	57308975	C	T	0.37	1.10E-02	4.29E-02	2.00E-01
rs4526799	Brain_Nucleus_accumbens_basal_ganglia	12	*NABP2*	56619718	rs11172056	0.9089	57308975	C	T	0.37	1.10E-02	1.79E-02	1.75E-01
rs4526799	Brain_Nucleus_accumbens_basal_ganglia	12	*HSD17B6*	57163759	rs11172056	0.9089	57308975	C	T	0.37	1.10E-02	2.28E-02	1.81E-01
rs4526799	Brain_Nucleus_accumbens_basal_ganglia	12	*DDIT3*	57912369	rs11172056	0.9089	57308975	C	T	0.37	1.10E-02	3.27E-02	1.91E-01
rs4526799	Brain_Nucleus_accumbens_basal_ganglia	12	*B4GALNT1*	58023536	rs11172056	0.9089	57308975	C	T	0.37	1.10E-02	4.85E-03	1.54E-01
rs4526799	Brain_Nucleus_accumbens_basal_ganglia	12	*RP11-977G19.5*	56570105	rs7302420	0.9085	57309884	G	C	0.36	1.54E-02	3.97E-02	2.24E-01
rs4526799	Brain_Nucleus_accumbens_basal_ganglia	12	*NABP2*	56619718	rs7302420	0.9085	57309884	G	C	0.36	1.54E-02	1.60E-02	2.02E-01
rs4526799	Brain_Nucleus_accumbens_basal_ganglia	12	*HSD17B6*	57163759	rs7302420	0.9085	57309884	G	C	0.36	1.54E-02	1.64E-02	2.02E-01
rs4526799	Brain_Nucleus_accumbens_basal_ganglia	12	*DDIT3*	57912369	rs7302420	0.9085	57309884	G	C	0.36	1.54E-02	4.86E-02	2.31E-01
rs4526799	Brain_Nucleus_accumbens_basal_ganglia	12	*B4GALNT1*	58023536	rs7302420	0.9085	57309884	G	C	0.36	1.54E-02	1.67E-02	2.02E-01
rs4526799	Brain_Nucleus_accumbens_basal_ganglia	12	*NABP2*	56619718	rs12228618	0.9128	57311229	T	C	0.37	1.29E-02	3.20E-02	1.99E-01
rs4526799	Brain_Nucleus_accumbens_basal_ganglia	12	*SDR9C7*	57322563	rs12228618	0.9128	57311229	T	C	0.37	1.29E-02	3.31E-02	2.00E-01
rs4526799	Brain_Nucleus_accumbens_basal_ganglia	12	*NAB2*	57485933	rs12228618	0.9128	57311229	T	C	0.37	1.29E-02	4.01E-02	2.06E-01
rs4526799	Brain_Nucleus_accumbens_basal_ganglia	12	*B4GALNT1*	58023536	rs12228618	0.9128	57311229	T	C	0.37	1.29E-02	2.68E-03	1.57E-01
rs4526799	Brain_Nucleus_accumbens_basal_ganglia	12	*NABP2*	56619718	rs9739473	0.9012	57313335	A	T	0.37	2.65E-02	2.49E-02	2.60E-01
rs4526799	Brain_Nucleus_accumbens_basal_ganglia	12	*SDR9C7*	57322563	rs9739473	0.9012	57313335	A	T	0.37	2.65E-02	4.63E-02	2.75E-01
rs4526799	Brain_Nucleus_accumbens_basal_ganglia	12	*NAB2*	57485933	rs9739473	0.9012	57313335	A	T	0.37	2.65E-02	4.81E-02	2.77E-01
rs4526799	Brain_Nucleus_accumbens_basal_ganglia	12	*B4GALNT1*	58023536	rs9739473	0.9012	57313335	A	T	0.37	2.65E-02	3.66E-03	2.34E-01
rs4526799	Brain_Nucleus_accumbens_basal_ganglia	12	*TSFM*	58189113	rs9739473	0.9012	57313335	A	T	0.37	2.65E-02	3.91E-02	2.71E-01
rs4526799	Brain_Putamen_basal_ganglia	12	*CDK2*	56363560	rs9919772	0.8450	57260027	T	C	0.33	1.03E-02	4.15E-02	2.29E-01
rs4526799	Brain_Putamen_basal_ganglia	12	*RPS26*	56436876	rs9919772	0.8450	57260027	T	C	0.33	1.03E-02	3.00E-02	2.19E-01
rs4526799	Brain_Putamen_basal_ganglia	12	*MYL6B*	56548905	rs9919772	0.8450	57260027	T	C	0.33	1.03E-02	6.28E-03	1.91E-01
rs4526799	Brain_Putamen_basal_ganglia	12	*CS*	56679829	rs9919772	0.8450	57260027	T	C	0.33	1.03E-02	1.59E-02	2.05E-01
rs4526799	Brain_Putamen_basal_ganglia	12	*RP11-977G19.11*	56701259	rs9919772	0.8450	57260027	T	C	0.33	1.03E-02	1.17E-02	2.00E-01
rs4526799	Brain_Putamen_basal_ganglia	12	*IL23A*	56733428	rs9919772	0.8450	57260027	T	C	0.33	1.03E-02	3.75E-02	2.26E-01
rs4526799	Brain_Putamen_basal_ganglia	12	*NAB2*	57485933	rs9919772	0.8450	57260027	T	C	0.33	1.03E-02	3.51E-02	2.24E-01
rs4526799	Brain_Putamen_basal_ganglia	12	*AVIL*	58201929	rs9919772	0.8450	57260027	T	C	0.33	1.03E-02	1.04E-02	1.98E-01
rs4526799	Brain_Putamen_basal_ganglia	12	*RPS26*	56436876	rs4495925	0.8650	57268116	C	G	0.33	1.04E-02	1.22E-02	1.97E-01
rs4526799	Brain_Putamen_basal_ganglia	12	*MYL6B*	56548905	rs4495925	0.8650	57268116	C	G	0.33	1.04E-02	1.61E-02	2.02E-01
rs4526799	Brain_Putamen_basal_ganglia	12	*CS*	56679829	rs4495925	0.8650	57268116	C	G	0.33	1.04E-02	2.25E-02	2.09E-01
rs4526799	Brain_Putamen_basal_ganglia	12	*RP11-977G19.11*	56701259	rs4495925	0.8650	57268116	C	G	0.33	1.04E-02	1.65E-02	2.03E-01
rs4526799	Brain_Putamen_basal_ganglia	12	*IL23A*	56733428	rs4495925	0.8650	57268116	C	G	0.33	1.04E-02	3.60E-02	2.22E-01
rs4526799	Brain_Putamen_basal_ganglia	12	*NAB2*	57485933	rs4495925	0.8650	57268116	C	G	0.33	1.04E-02	2.16E-02	2.08E-01
rs4526799	Brain_Putamen_basal_ganglia	12	*AVIL*	58201929	rs4495925	0.8650	57268116	C	G	0.33	1.04E-02	1.52E-02	2.01E-01
rs4526799	Brain_Putamen_basal_ganglia	12	*RPS26*	56436876	rs4471472	0.8650	57268985	A	G	0.32	9.99E-03	1.26E-02	1.94E-01
rs4526799	Brain_Putamen_basal_ganglia	12	*MYL6B*	56548905	rs4471472	0.8650	57268985	A	G	0.32	9.99E-03	1.92E-02	2.02E-01
rs4526799	Brain_Putamen_basal_ganglia	12	*CS*	56679829	rs4471472	0.8650	57268985	A	G	0.32	9.99E-03	2.18E-02	2.05E-01
rs4526799	Brain_Putamen_basal_ganglia	12	*RP11-977G19.11*	56701259	rs4471472	0.8650	57268985	A	G	0.32	9.99E-03	1.98E-02	2.03E-01
rs4526799	Brain_Putamen_basal_ganglia	12	*IL23A*	56733428	rs4471472	0.8650	57268985	A	G	0.32	9.99E-03	3.24E-02	2.15E-01
rs4526799	Brain_Putamen_basal_ganglia	12	*NAB2*	57485933	rs4471472	0.8650	57268985	A	G	0.32	9.99E-03	2.58E-02	2.09E-01

rs4526799	Brain_Putamen_basal_ganglia	12	*AVIL*	58201929	rs4471472	0.8650	57268985	A	G	0.32	9.99E-03	1.32E-02	1.95E-01
rs4526799	Brain_Putamen_basal_ganglia	12	*WIBG*	56310770	rs4633499	0.8610	57269264	A	T	0.32	1.43E-02	4.30E-02	2.57E-01
rs4526799	Brain_Putamen_basal_ganglia	12	*RPS26*	56436876	rs4633499	0.8610	57269264	A	T	0.32	1.43E-02	1.00E-02	2.27E-01
rs4526799	Brain_Putamen_basal_ganglia	12	*MYL6B*	56548905	rs4633499	0.8610	57269264	A	T	0.32	1.43E-02	3.52E-02	2.51E-01
rs4526799	Brain_Putamen_basal_ganglia	12	*NABP2*	56619718	rs4633499	0.8610	57269264	A	T	0.32	1.43E-02	3.42E-02	2.51E-01
rs4526799	Brain_Putamen_basal_ganglia	12	*CS*	56679829	rs4633499	0.8610	57269264	A	T	0.32	1.43E-02	1.60E-02	2.34E-01
rs4526799	Brain_Putamen_basal_ganglia	12	*RP11-977G19.11*	56701259	rs4633499	0.8610	57269264	A	T	0.32	1.43E-02	2.61E-02	2.44E-01
rs4526799	Brain_Putamen_basal_ganglia	12	*PAN2*	56718979	rs4633499	0.8610	57269264	A	T	0.32	1.43E-02	3.34E-02	2.50E-01
rs4526799	Brain_Putamen_basal_ganglia	12	*IL23A*	56733428	rs4633499	0.8610	57269264	A	T	0.32	1.43E-02	1.40E-02	2.32E-01
rs4526799	Brain_Putamen_basal_ganglia	12	*NAB2*	57485933	rs4633499	0.8610	57269264	A	T	0.32	1.43E-02	2.77E-02	2.45E-01
rs4526799	Brain_Putamen_basal_ganglia	12	*AVIL*	58201929	rs4633499	0.8610	57269264	A	T	0.32	1.43E-02	1.85E-02	2.37E-01
rs4526799	Brain_Putamen_basal_ganglia	12	*WIBG*	56310770	rs12300079	0.9956	57273194	T	C	0.36	3.73E-03	3.45E-02	1.69E-01
rs4526799	Brain_Putamen_basal_ganglia	12	*CDK2*	56363560	rs12300079	0.9956	57273194	T	C	0.36	3.73E-03	1.90E-02	1.52E-01
rs4526799	Brain_Putamen_basal_ganglia	12	*RPS26*	56436876	rs12300079	0.9956	57273194	T	C	0.36	3.73E-03	1.38E-02	1.45E-01
rs4526799	Brain_Putamen_basal_ganglia	12	*MYL6B*	56548905	rs12300079	0.9956	57273194	T	C	0.36	3.73E-03	1.59E-02	1.48E-01
rs4526799	Brain_Putamen_basal_ganglia	12	*NABP2*	56619718	rs12300079	0.9956	57273194	T	C	0.36	3.73E-03	3.66E-02	1.71E-01
rs4526799	Brain_Putamen_basal_ganglia	12	*CS*	56679829	rs12300079	0.9956	57273194	T	C	0.36	3.73E-03	3.12E-02	1.66E-01
rs4526799	Brain_Putamen_basal_ganglia	12	*RP11-977G19.11*	56701259	rs12300079	0.9956	57273194	T	C	0.36	3.73E-03	3.55E-02	1.70E-01
rs4526799	Brain_Putamen_basal_ganglia	12	*IL23A*	56733428	rs12300079	0.9956	57273194	T	C	0.36	3.73E-03	1.97E-02	1.53E-01
rs4526799	Brain_Putamen_basal_ganglia	12	*NAB2*	57485933	rs12300079	0.9956	57273194	T	C	0.36	3.73E-03	7.23E-03	1.33E-01
rs4526799	Brain_Putamen_basal_ganglia	12	*INHBE*	57849584	rs12300079	0.9956	57273194	T	C	0.36	3.73E-03	3.79E-02	1.72E-01
rs4526799	Brain_Putamen_basal_ganglia	12	*AVIL*	58201929	rs12300079	0.9956	57273194	T	C	0.36	3.73E-03	2.01E-02	1.53E-01
rs4526799	Brain_Putamen_basal_ganglia	12	*WIBG*	56310770	rs12300191	0.9956	57273289	A	G	0.36	3.59E-03	3.45E-02	1.67E-01
rs4526799	Brain_Putamen_basal_ganglia	12	*CDK2*	56363560	rs12300191	0.9956	57273289	A	G	0.36	3.59E-03	1.90E-02	1.49E-01
rs4526799	Brain_Putamen_basal_ganglia	12	*RPS26*	56436876	rs12300191	0.9956	57273289	A	G	0.36	3.59E-03	1.38E-02	1.42E-01
rs4526799	Brain_Putamen_basal_ganglia	12	*MYL6B*	56548905	rs12300191	0.9956	57273289	A	G	0.36	3.59E-03	1.59E-02	1.45E-01
rs4526799	Brain_Putamen_basal_ganglia	12	*NABP2*	56619718	rs12300191	0.9956	57273289	A	G	0.36	3.59E-03	3.66E-02	1.69E-01
rs4526799	Brain_Putamen_basal_ganglia	12	*CS*	56679829	rs12300191	0.9956	57273289	A	G	0.36	3.59E-03	3.12E-02	1.63E-01
rs4526799	Brain_Putamen_basal_ganglia	12	*RP11-977G19.11*	56701259	rs12300191	0.9956	57273289	A	G	0.36	3.59E-03	3.55E-02	1.68E-01
rs4526799	Brain_Putamen_basal_ganglia	12	*IL23A*	56733428	rs12300191	0.9956	57273289	A	G	0.36	3.59E-03	1.97E-02	1.50E-01
rs4526799	Brain_Putamen_basal_ganglia	12	*NAB2*	57485933	rs12300191	0.9956	57273289	A	G	0.36	3.59E-03	7.23E-03	1.31E-01
rs4526799	Brain_Putamen_basal_ganglia	12	*INHBE*	57849584	rs12300191	0.9956	57273289	A	G	0.36	3.59E-03	3.79E-02	1.70E-01
rs4526799	Brain_Putamen_basal_ganglia	12	*AVIL*	58201929	rs12300191	0.9956	57273289	A	G	0.36	3.59E-03	2.01E-02	1.51E-01
rs4526799	Brain_Putamen_basal_ganglia	12	*WIBG*	56310770	rs4514464	0.9956	57276375	C	T	0.36	2.92E-03	3.45E-02	1.64E-01
rs4526799	Brain_Putamen_basal_ganglia	12	*CDK2*	56363560	rs4514464	0.9956	57276375	C	T	0.36	2.92E-03	1.90E-02	1.46E-01
rs4526799	Brain_Putamen_basal_ganglia	12	*RPS26*	56436876	rs4514464	0.9956	57276375	C	T	0.36	2.92E-03	1.38E-02	1.39E-01
rs4526799	Brain_Putamen_basal_ganglia	12	*MYL6B*	56548905	rs4514464	0.9956	57276375	C	T	0.36	2.92E-03	1.59E-02	1.42E-01
rs4526799	Brain_Putamen_basal_ganglia	12	*NABP2*	56619718	rs4514464	0.9956	57276375	C	T	0.36	2.92E-03	3.66E-02	1.66E-01
rs4526799	Brain_Putamen_basal_ganglia	12	*CS*	56679829	rs4514464	0.9956	57276375	C	T	0.36	2.92E-03	3.12E-02	1.60E-01
rs4526799	Brain_Putamen_basal_ganglia	12	*RP11-977G19.11*	56701259	rs4514464	0.9956	57276375	C	T	0.36	2.92E-03	3.55E-02	1.65E-01

rs4526799	Brain_Putamen_basal_ganglia	12	*IL23A*	56733428	rs4514464	0.9956	57276375	C	T	0.36	2.92E-03	1.97E-02	1.47E-01
rs4526799	Brain_Putamen_basal_ganglia	12	*NAB2*	57485933	rs4514464	0.9956	57276375	C	T	0.36	2.92E-03	7.23E-03	1.28E-01
rs4526799	Brain_Putamen_basal_ganglia	12	*INHBE*	57849584	rs4514464	0.9956	57276375	C	T	0.36	2.92E-03	3.79E-02	1.67E-01
rs4526799	Brain_Putamen_basal_ganglia	12	*AVIL*	58201929	rs4514464	0.9956	57276375	C	T	0.36	2.92E-03	2.01E-02	1.48E-01
rs4526799	Brain_Putamen_basal_ganglia	12	*WIBG*	56310770	rs4417325	0.9956	57277302	G	A	0.36	3.59E-03	3.45E-02	1.67E-01
rs4526799	Brain_Putamen_basal_ganglia	12	*CDK2*	56363560	rs4417325	0.9956	57277302	G	A	0.36	3.59E-03	1.90E-02	1.49E-01
rs4526799	Brain_Putamen_basal_ganglia	12	*RPS26*	56436876	rs4417325	0.9956	57277302	G	A	0.36	3.59E-03	1.38E-02	1.42E-01
rs4526799	Brain_Putamen_basal_ganglia	12	*MYL6B*	56548905	rs4417325	0.9956	57277302	G	A	0.36	3.59E-03	1.59E-02	1.45E-01
rs4526799	Brain_Putamen_basal_ganglia	12	*NABP2*	56619718	rs4417325	0.9956	57277302	G	A	0.36	3.59E-03	3.66E-02	1.69E-01
rs4526799	Brain_Putamen_basal_ganglia	12	*CS*	56679829	rs4417325	0.9956	57277302	G	A	0.36	3.59E-03	3.12E-02	1.63E-01
rs4526799	Brain_Putamen_basal_ganglia	12	*RP11-977G19.11*	56701259	rs4417325	0.9956	57277302	G	A	0.36	3.59E-03	3.55E-02	1.68E-01
rs4526799	Brain_Putamen_basal_ganglia	12	*IL23A*	56733428	rs4417325	0.9956	57277302	G	A	0.36	3.59E-03	1.97E-02	1.50E-01
rs4526799	Brain_Putamen_basal_ganglia	12	*NAB2*	57485933	rs4417325	0.9956	57277302	G	A	0.36	3.59E-03	7.23E-03	1.31E-01
rs4526799	Brain_Putamen_basal_ganglia	12	*INHBE*	57849584	rs4417325	0.9956	57277302	G	A	0.36	3.59E-03	3.79E-02	1.70E-01
rs4526799	Brain_Putamen_basal_ganglia	12	*AVIL*	58201929	rs4417325	0.9956	57277302	G	A	0.36	3.59E-03	2.01E-02	1.51E-01
rs4526799	Brain_Putamen_basal_ganglia	12	*WIBG*	56310770	rs11172030	0.9956	57278076	A	C	0.36	3.63E-03	3.45E-02	1.67E-01
rs4526799	Brain_Putamen_basal_ganglia	12	*CDK2*	56363560	rs11172030	0.9956	57278076	A	C	0.36	3.63E-03	1.90E-02	1.50E-01
rs4526799	Brain_Putamen_basal_ganglia	12	*RPS26*	56436876	rs11172030	0.9956	57278076	A	C	0.36	3.63E-03	1.38E-02	1.43E-01
rs4526799	Brain_Putamen_basal_ganglia	12	*MYL6B*	56548905	rs11172030	0.9956	57278076	A	C	0.36	3.63E-03	1.59E-02	1.46E-01
rs4526799	Brain_Putamen_basal_ganglia	12	*NABP2*	56619718	rs11172030	0.9956	57278076	A	C	0.36	3.63E-03	3.66E-02	1.69E-01
rs4526799	Brain_Putamen_basal_ganglia	12	*CS*	56679829	rs11172030	0.9956	57278076	A	C	0.36	3.63E-03	3.12E-02	1.64E-01
rs4526799	Brain_Putamen_basal_ganglia	12	*RP11-977G19.11*	56701259	rs11172030	0.9956	57278076	A	C	0.36	3.63E-03	3.55E-02	1.68E-01
rs4526799	Brain_Putamen_basal_ganglia	12	*IL23A*	56733428	rs11172030	0.9956	57278076	A	C	0.36	3.63E-03	1.97E-02	1.51E-01
rs4526799	Brain_Putamen_basal_ganglia	12	*NAB2*	57485933	rs11172030	0.9956	57278076	A	C	0.36	3.63E-03	7.23E-03	1.31E-01
rs4526799	Brain_Putamen_basal_ganglia	12	*INHBE*	57849584	rs11172030	0.9956	57278076	A	C	0.36	3.63E-03	3.79E-02	1.71E-01
rs4526799	Brain_Putamen_basal_ganglia	12	*AVIL*	58201929	rs11172030	0.9956	57278076	A	C	0.36	3.63E-03	2.01E-02	1.51E-01
rs4526799	Brain_Putamen_basal_ganglia	12	*WIBG*	56310770	rs10876944	0.9956	57279372	T	A	0.36	3.63E-03	3.45E-02	1.67E-01
rs4526799	Brain_Putamen_basal_ganglia	12	*CDK2*	56363560	rs10876944	0.9956	57279372	T	A	0.36	3.63E-03	1.90E-02	1.50E-01
rs4526799	Brain_Putamen_basal_ganglia	12	*RPS26*	56436876	rs10876944	0.9956	57279372	T	A	0.36	3.63E-03	1.38E-02	1.43E-01
rs4526799	Brain_Putamen_basal_ganglia	12	*MYL6B*	56548905	rs10876944	0.9956	57279372	T	A	0.36	3.63E-03	1.59E-02	1.46E-01
rs4526799	Brain_Putamen_basal_ganglia	12	*NABP2*	56619718	rs10876944	0.9956	57279372	T	A	0.36	3.63E-03	3.66E-02	1.69E-01
rs4526799	Brain_Putamen_basal_ganglia	12	*CS*	56679829	rs10876944	0.9956	57279372	T	A	0.36	3.63E-03	3.12E-02	1.64E-01
rs4526799	Brain_Putamen_basal_ganglia	12	*RP11-977G19.11*	56701259	rs10876944	0.9956	57279372	T	A	0.36	3.63E-03	3.55E-02	1.68E-01
rs4526799	Brain_Putamen_basal_ganglia	12	*IL23A*	56733428	rs10876944	0.9956	57279372	T	A	0.36	3.63E-03	1.97E-02	1.51E-01
rs4526799	Brain_Putamen_basal_ganglia	12	*NAB2*	57485933	rs10876944	0.9956	57279372	T	A	0.36	3.63E-03	7.23E-03	1.31E-01
rs4526799	Brain_Putamen_basal_ganglia	12	*INHBE*	57849584	rs10876944	0.9956	57279372	T	A	0.36	3.63E-03	3.79E-02	1.71E-01
rs4526799	Brain_Putamen_basal_ganglia	12	*AVIL*	58201929	rs10876944	0.9956	57279372	T	A	0.36	3.63E-03	2.01E-02	1.51E-01
rs4526799	Brain_Putamen_basal_ganglia	12	*WIBG*	56310770	rs4326839	0.9956	57280374	G	C	0.36	3.58E-03	3.45E-02	1.67E-01
rs4526799	Brain_Putamen_basal_ganglia	12	*CDK2*	56363560	rs4326839	0.9956	57280374	G	C	0.36	3.58E-03	1.90E-02	1.49E-01
rs4526799	Brain_Putamen_basal_ganglia	12	*RPS26*	56436876	rs4326839	0.9956	57280374	G	C	0.36	3.58E-03	1.38E-02	1.42E-01

rs4526799	Brain_Putamen_basal_ganglia	12	*MYL6B*	56548905	rs4326839	0.9956	57280374	G	C	0.36	3.58E-03	1.59E-02	1.45E-01
rs4526799	Brain_Putamen_basal_ganglia	12	*NABP2*	56619718	rs4326839	0.9956	57280374	G	C	0.36	3.58E-03	3.66E-02	1.69E-01
rs4526799	Brain_Putamen_basal_ganglia	12	*CS*	56679829	rs4326839	0.9956	57280374	G	C	0.36	3.58E-03	3.12E-02	1.63E-01
rs4526799	Brain_Putamen_basal_ganglia	12	*RP11-977G19.11*	56701259	rs4326839	0.9956	57280374	G	C	0.36	3.58E-03	3.55E-02	1.68E-01
rs4526799	Brain_Putamen_basal_ganglia	12	*IL23A*	56733428	rs4326839	0.9956	57280374	G	C	0.36	3.58E-03	1.97E-02	1.50E-01
rs4526799	Brain_Putamen_basal_ganglia	12	*NAB2*	57485933	rs4326839	0.9956	57280374	G	C	0.36	3.58E-03	7.23E-03	1.31E-01
rs4526799	Brain_Putamen_basal_ganglia	12	*INHBE*	57849584	rs4326839	0.9956	57280374	G	C	0.36	3.58E-03	3.79E-02	1.70E-01
rs4526799	Brain_Putamen_basal_ganglia	12	*AVIL*	58201929	rs4326839	0.9956	57280374	G	C	0.36	3.58E-03	2.01E-02	1.51E-01
rs4526799	Brain_Putamen_basal_ganglia	12	*WIBG*	56310770	rs4526799	1.0000	57280586	T	C	0.34	7.26E-06	3.45E-02	6.93E-02
rs4526799	Brain_Putamen_basal_ganglia	12	*CDK2*	56363560	rs4526799	1.0000	57280586	T	C	0.34	7.26E-06	1.90E-02	5.03E-02
rs4526799	Brain_Putamen_basal_ganglia	12	*RPS26*	56436876	rs4526799	1.0000	57280586	T	C	0.34	7.26E-06	1.38E-02	4.30E-02
rs4526799	Brain_Putamen_basal_ganglia	12	*MYL6B*	56548905	rs4526799	1.0000	57280586	T	C	0.34	7.26E-06	1.59E-02	4.60E-02
rs4526799	Brain_Putamen_basal_ganglia	12	*NABP2*	56619718	rs4526799	1.0000	57280586	T	C	0.34	7.26E-06	3.66E-02	7.16E-02
rs4526799	Brain_Putamen_basal_ganglia	12	*CS*	56679829	rs4526799	1.0000	57280586	T	C	0.34	7.26E-06	3.12E-02	6.55E-02
rs4526799	Brain_Putamen_basal_ganglia	12	*RP11-977G19.11*	56701259	rs4526799	1.0000	57280586	T	C	0.34	7.26E-06	3.55E-02	7.04E-02
rs4526799	Brain_Putamen_basal_ganglia	12	*IL23A*	56733428	rs4526799	1.0000	57280586	T	C	0.34	7.26E-06	1.97E-02	5.13E-02
rs4526799	Brain_Putamen_basal_ganglia	12	*NAB2*	57485933	rs4526799	1.0000	57280586	T	C	0.34	7.26E-06	7.23E-03	3.22E-02
rs4526799	Brain_Putamen_basal_ganglia	12	*INHBE*	57849584	rs4526799	1.0000	57280586	T	C	0.34	7.26E-06	3.79E-02	7.31E-02
rs4526799	Brain_Putamen_basal_ganglia	12	*AVIL*	58201929	rs4526799	1.0000	57280586	T	C	0.34	7.26E-06	2.01E-02	5.18E-02
rs4526799	Brain_Putamen_basal_ganglia	12	*WIBG*	56310770	rs28876529	0.9956	57285301	T	A	0.36	2.25E-03	3.45E-02	1.52E-01
rs4526799	Brain_Putamen_basal_ganglia	12	*CDK2*	56363560	rs28876529	0.9956	57285301	T	A	0.36	2.25E-03	1.90E-02	1.34E-01
rs4526799	Brain_Putamen_basal_ganglia	12	*RPS26*	56436876	rs28876529	0.9956	57285301	T	A	0.36	2.25E-03	1.38E-02	1.26E-01
rs4526799	Brain_Putamen_basal_ganglia	12	*MYL6B*	56548905	rs28876529	0.9956	57285301	T	A	0.36	2.25E-03	1.59E-02	1.29E-01
rs4526799	Brain_Putamen_basal_ganglia	12	*NABP2*	56619718	rs28876529	0.9956	57285301	T	A	0.36	2.25E-03	3.66E-02	1.54E-01
rs4526799	Brain_Putamen_basal_ganglia	12	*CS*	56679829	rs28876529	0.9956	57285301	T	A	0.36	2.25E-03	3.12E-02	1.48E-01
rs4526799	Brain_Putamen_basal_ganglia	12	*RP11-977G19.11*	56701259	rs28876529	0.9956	57285301	T	A	0.36	2.25E-03	3.55E-02	1.53E-01
rs4526799	Brain_Putamen_basal_ganglia	12	*IL23A*	56733428	rs28876529	0.9956	57285301	T	A	0.36	2.25E-03	1.97E-02	1.35E-01
rs4526799	Brain_Putamen_basal_ganglia	12	*NAB2*	57485933	rs28876529	0.9956	57285301	T	A	0.36	2.25E-03	7.23E-03	1.15E-01
rs4526799	Brain_Putamen_basal_ganglia	12	*INHBE*	57849584	rs28876529	0.9956	57285301	T	A	0.36	2.25E-03	3.79E-02	1.55E-01
rs4526799	Brain_Putamen_basal_ganglia	12	*AVIL*	58201929	rs28876529	0.9956	57285301	T	A	0.36	2.25E-03	2.01E-02	1.35E-01
rs4526799	Brain_Putamen_basal_ganglia	12	*WIBG*	56310770	rs11172037	0.9956	57285427	T	A	0.36	2.25E-03	3.45E-02	1.52E-01
rs4526799	Brain_Putamen_basal_ganglia	12	*CDK2*	56363560	rs11172037	0.9956	57285427	T	A	0.36	2.25E-03	1.90E-02	1.34E-01
rs4526799	Brain_Putamen_basal_ganglia	12	*RPS26*	56436876	rs11172037	0.9956	57285427	T	A	0.36	2.25E-03	1.38E-02	1.26E-01
rs4526799	Brain_Putamen_basal_ganglia	12	*MYL6B*	56548905	rs11172037	0.9956	57285427	T	A	0.36	2.25E-03	1.59E-02	1.29E-01
rs4526799	Brain_Putamen_basal_ganglia	12	*NABP2*	56619718	rs11172037	0.9956	57285427	T	A	0.36	2.25E-03	3.66E-02	1.54E-01
rs4526799	Brain_Putamen_basal_ganglia	12	*CS*	56679829	rs11172037	0.9956	57285427	T	A	0.36	2.25E-03	3.12E-02	1.48E-01
rs4526799	Brain_Putamen_basal_ganglia	12	*RP11-977G19.11*	56701259	rs11172037	0.9956	57285427	T	A	0.36	2.25E-03	3.55E-02	1.53E-01
rs4526799	Brain_Putamen_basal_ganglia	12	*IL23A*	56733428	rs11172037	0.9956	57285427	T	A	0.36	2.25E-03	1.97E-02	1.35E-01
rs4526799	Brain_Putamen_basal_ganglia	12	*NAB2*	57485933	rs11172037	0.9956	57285427	T	A	0.36	2.25E-03	7.23E-03	1.15E-01
rs4526799	Brain_Putamen_basal_ganglia	12	*INHBE*	57849584	rs11172037	0.9956	57285427	T	A	0.36	2.25E-03	3.79E-02	1.55E-01

rs4526799	Brain_Putamen_basal_ganglia	12	*AVIL*	58201929	rs11172037	0.9956	57285427	T	A	0.36	2.25E-03	2.01E-02	1.35E-01
rs4526799	Brain_Putamen_basal_ganglia	12	*WIBG*	56310770	rs12321987	0.9956	57288449	G	A	0.36	4.47E-03	3.45E-02	1.88E-01
rs4526799	Brain_Putamen_basal_ganglia	12	*CDK2*	56363560	rs12321987	0.9956	57288449	G	A	0.36	4.47E-03	1.90E-02	1.71E-01
rs4526799	Brain_Putamen_basal_ganglia	12	*RPS26*	56436876	rs12321987	0.9956	57288449	G	A	0.36	4.47E-03	1.38E-02	1.65E-01
rs4526799	Brain_Putamen_basal_ganglia	12	*MYL6B*	56548905	rs12321987	0.9956	57288449	G	A	0.36	4.47E-03	1.59E-02	1.67E-01
rs4526799	Brain_Putamen_basal_ganglia	12	*NABP2*	56619718	rs12321987	0.9956	57288449	G	A	0.36	4.47E-03	3.66E-02	1.90E-01
rs4526799	Brain_Putamen_basal_ganglia	12	*CS*	56679829	rs12321987	0.9956	57288449	G	A	0.36	4.47E-03	3.12E-02	1.85E-01
rs4526799	Brain_Putamen_basal_ganglia	12	*RP11-977G19.11*	56701259	rs12321987	0.9956	57288449	G	A	0.36	4.47E-03	3.55E-02	1.89E-01
rs4526799	Brain_Putamen_basal_ganglia	12	*IL23A*	56733428	rs12321987	0.9956	57288449	G	A	0.36	4.47E-03	1.97E-02	1.72E-01
rs4526799	Brain_Putamen_basal_ganglia	12	*NAB2*	57485933	rs12321987	0.9956	57288449	G	A	0.36	4.47E-03	7.23E-03	1.54E-01
rs4526799	Brain_Putamen_basal_ganglia	12	*INHBE*	57849584	rs12321987	0.9956	57288449	G	A	0.36	4.47E-03	3.79E-02	1.91E-01
rs4526799	Brain_Putamen_basal_ganglia	12	*AVIL*	58201929	rs12321987	0.9956	57288449	G	A	0.36	4.47E-03	2.01E-02	1.73E-01
rs4526799	Brain_Putamen_basal_ganglia	12	*WIBG*	56310770	rs11172043	0.9869	57293182	G	A	0.35	4.58E-03	3.17E-02	1.84E-01
rs4526799	Brain_Putamen_basal_ganglia	12	*CDK2*	56363560	rs11172043	0.9869	57293182	G	A	0.35	4.58E-03	3.47E-02	1.87E-01
rs4526799	Brain_Putamen_basal_ganglia	12	*RPS26*	56436876	rs11172043	0.9869	57293182	G	A	0.35	4.58E-03	1.68E-02	1.67E-01
rs4526799	Brain_Putamen_basal_ganglia	12	*MYL6B*	56548905	rs11172043	0.9869	57293182	G	A	0.35	4.58E-03	1.32E-02	1.62E-01
rs4526799	Brain_Putamen_basal_ganglia	12	*NABP2*	56619718	rs11172043	0.9869	57293182	G	A	0.35	4.58E-03	1.34E-02	1.63E-01
rs4526799	Brain_Putamen_basal_ganglia	12	*RP11-977G19.14*	56662806	rs11172043	0.9869	57293182	G	A	0.35	4.58E-03	1.76E-02	1.68E-01
rs4526799	Brain_Putamen_basal_ganglia	12	*CS*	56679829	rs11172043	0.9869	57293182	G	A	0.35	4.58E-03	3.19E-02	1.84E-01
rs4526799	Brain_Putamen_basal_ganglia	12	*IL23A*	56733428	rs11172043	0.9869	57293182	G	A	0.35	4.58E-03	8.96E-03	1.55E-01
rs4526799	Brain_Putamen_basal_ganglia	12	*NAB2*	57485933	rs11172043	0.9869	57293182	G	A	0.35	4.58E-03	1.42E-02	1.64E-01
rs4526799	Brain_Putamen_basal_ganglia	12	*INHBE*	57849584	rs11172043	0.9869	57293182	G	A	0.35	4.58E-03	4.04E-02	1.92E-01
rs4526799	Brain_Putamen_basal_ganglia	12	*AVIL*	58201929	rs11172043	0.9869	57293182	G	A	0.35	4.58E-03	1.92E-02	1.70E-01
rs4526799	Brain_Putamen_basal_ganglia	12	*WIBG*	56310770	rs12426816	0.9869	57294074	A	C	0.35	4.50E-03	3.17E-02	1.83E-01
rs4526799	Brain_Putamen_basal_ganglia	12	*CDK2*	56363560	rs12426816	0.9869	57294074	A	C	0.35	4.50E-03	3.47E-02	1.86E-01
rs4526799	Brain_Putamen_basal_ganglia	12	*RPS26*	56436876	rs12426816	0.9869	57294074	A	C	0.35	4.50E-03	1.68E-02	1.67E-01
rs4526799	Brain_Putamen_basal_ganglia	12	*MYL6B*	56548905	rs12426816	0.9869	57294074	A	C	0.35	4.50E-03	1.32E-02	1.62E-01
rs4526799	Brain_Putamen_basal_ganglia	12	*NABP2*	56619718	rs12426816	0.9869	57294074	A	C	0.35	4.50E-03	1.34E-02	1.62E-01
rs4526799	Brain_Putamen_basal_ganglia	12	*RP11-977G19.14*	56662806	rs12426816	0.9869	57294074	A	C	0.35	4.50E-03	1.76E-02	1.68E-01
rs4526799	Brain_Putamen_basal_ganglia	12	*CS*	56679829	rs12426816	0.9869	57294074	A	C	0.35	4.50E-03	3.19E-02	1.84E-01
rs4526799	Brain_Putamen_basal_ganglia	12	*IL23A*	56733428	rs12426816	0.9869	57294074	A	C	0.35	4.50E-03	8.96E-03	1.55E-01
rs4526799	Brain_Putamen_basal_ganglia	12	*NAB2*	57485933	rs12426816	0.9869	57294074	A	C	0.35	4.50E-03	1.42E-02	1.63E-01
rs4526799	Brain_Putamen_basal_ganglia	12	*INHBE*	57849584	rs12426816	0.9869	57294074	A	C	0.35	4.50E-03	4.04E-02	1.92E-01
rs4526799	Brain_Putamen_basal_ganglia	12	*AVIL*	58201929	rs12426816	0.9869	57294074	A	C	0.35	4.50E-03	1.92E-02	1.70E-01
rs4526799	Brain_Putamen_basal_ganglia	12	*WIBG*	56310770	rs11172047	0.9869	57298080	T	C	0.35	6.51E-03	3.17E-02	1.97E-01
rs4526799	Brain_Putamen_basal_ganglia	12	*CDK2*	56363560	rs11172047	0.9869	57298080	T	C	0.35	6.51E-03	3.47E-02	2.00E-01
rs4526799	Brain_Putamen_basal_ganglia	12	*RPS26*	56436876	rs11172047	0.9869	57298080	T	C	0.35	6.51E-03	1.68E-02	1.82E-01
rs4526799	Brain_Putamen_basal_ganglia	12	*MYL6B*	56548905	rs11172047	0.9869	57298080	T	C	0.35	6.51E-03	1.32E-02	1.77E-01
rs4526799	Brain_Putamen_basal_ganglia	12	*NABP2*	56619718	rs11172047	0.9869	57298080	T	C	0.35	6.51E-03	1.34E-02	1.77E-01
rs4526799	Brain_Putamen_basal_ganglia	12	*RP11-977G19.14*	56662806	rs11172047	0.9869	57298080	T	C	0.35	6.51E-03	1.76E-02	1.82E-01

rs4526799	Brain_Putamen_basal_ganglia	12	*CS*	56679829	rs11172047	0.9869	57298080	T	C	0.35	6.51E-03	3.19E-02	1.98E-01
rs4526799	Brain_Putamen_basal_ganglia	12	*IL23A*	56733428	rs11172047	0.9869	57298080	T	C	0.35	6.51E-03	8.96E-03	1.70E-01
rs4526799	Brain_Putamen_basal_ganglia	12	*NAB2*	57485933	rs11172047	0.9869	57298080	T	C	0.35	6.51E-03	1.42E-02	1.78E-01
rs4526799	Brain_Putamen_basal_ganglia	12	*INHBE*	57849584	rs11172047	0.9869	57298080	T	C	0.35	6.51E-03	4.04E-02	2.05E-01
rs4526799	Brain_Putamen_basal_ganglia	12	*AVIL*	58201929	rs11172047	0.9869	57298080	T	C	0.35	6.51E-03	1.92E-02	1.84E-01
rs4526799	Brain_Putamen_basal_ganglia	12	*WIBG*	56310770	rs2371631	0.9869	57298614	T	A	0.35	6.32E-03	3.17E-02	1.94E-01
rs4526799	Brain_Putamen_basal_ganglia	12	*CDK2*	56363560	rs2371631	0.9869	57298614	T	A	0.35	6.32E-03	3.47E-02	1.97E-01
rs4526799	Brain_Putamen_basal_ganglia	12	*RPS26*	56436876	rs2371631	0.9869	57298614	T	A	0.35	6.32E-03	1.68E-02	1.78E-01
rs4526799	Brain_Putamen_basal_ganglia	12	*MYL6B*	56548905	rs2371631	0.9869	57298614	T	A	0.35	6.32E-03	1.32E-02	1.73E-01
rs4526799	Brain_Putamen_basal_ganglia	12	*NABP2*	56619718	rs2371631	0.9869	57298614	T	A	0.35	6.32E-03	1.34E-02	1.74E-01
rs4526799	Brain_Putamen_basal_ganglia	12	*RP11-977G19.14*	56662806	rs2371631	0.9869	57298614	T	A	0.35	6.32E-03	1.76E-02	1.79E-01
rs4526799	Brain_Putamen_basal_ganglia	12	*CS*	56679829	rs2371631	0.9869	57298614	T	A	0.35	6.32E-03	3.19E-02	1.95E-01
rs4526799	Brain_Putamen_basal_ganglia	12	*IL23A*	56733428	rs2371631	0.9869	57298614	T	A	0.35	6.32E-03	8.96E-03	1.67E-01
rs4526799	Brain_Putamen_basal_ganglia	12	*NAB2*	57485933	rs2371631	0.9869	57298614	T	A	0.35	6.32E-03	1.42E-02	1.75E-01
rs4526799	Brain_Putamen_basal_ganglia	12	*INHBE*	57849584	rs2371631	0.9869	57298614	T	A	0.35	6.32E-03	4.04E-02	2.02E-01
rs4526799	Brain_Putamen_basal_ganglia	12	*AVIL*	58201929	rs2371631	0.9869	57298614	T	A	0.35	6.32E-03	1.92E-02	1.81E-01
rs4526799	Brain_Putamen_basal_ganglia	12	*WIBG*	56310770	rs12305763	0.9869	57299263	G	A	0.35	6.82E-03	3.17E-02	1.99E-01
rs4526799	Brain_Putamen_basal_ganglia	12	*CDK2*	56363560	rs12305763	0.9869	57299263	G	A	0.35	6.82E-03	3.47E-02	2.01E-01
rs4526799	Brain_Putamen_basal_ganglia	12	*RPS26*	56436876	rs12305763	0.9869	57299263	G	A	0.35	6.82E-03	1.68E-02	1.83E-01
rs4526799	Brain_Putamen_basal_ganglia	12	*MYL6B*	56548905	rs12305763	0.9869	57299263	G	A	0.35	6.82E-03	1.32E-02	1.78E-01
rs4526799	Brain_Putamen_basal_ganglia	12	*NABP2*	56619718	rs12305763	0.9869	57299263	G	A	0.35	6.82E-03	1.34E-02	1.78E-01
rs4526799	Brain_Putamen_basal_ganglia	12	*RP11-977G19.14*	56662806	rs12305763	0.9869	57299263	G	A	0.35	6.82E-03	1.76E-02	1.84E-01
rs4526799	Brain_Putamen_basal_ganglia	12	*CS*	56679829	rs12305763	0.9869	57299263	G	A	0.35	6.82E-03	3.19E-02	1.99E-01
rs4526799	Brain_Putamen_basal_ganglia	12	*IL23A*	56733428	rs12305763	0.9869	57299263	G	A	0.35	6.82E-03	8.96E-03	1.72E-01
rs4526799	Brain_Putamen_basal_ganglia	12	*NAB2*	57485933	rs12305763	0.9869	57299263	G	A	0.35	6.82E-03	1.42E-02	1.79E-01
rs4526799	Brain_Putamen_basal_ganglia	12	*INHBE*	57849584	rs12305763	0.9869	57299263	G	A	0.35	6.82E-03	4.04E-02	2.06E-01
rs4526799	Brain_Putamen_basal_ganglia	12	*AVIL*	58201929	rs12305763	0.9869	57299263	G	A	0.35	6.82E-03	1.92E-02	1.86E-01
rs4526799	Brain_Putamen_basal_ganglia	12	*WIBG*	56310770	rs11172049	0.9128	57304203	T	C	0.35	1.15E-02	2.53E-02	1.93E-01
rs4526799	Brain_Putamen_basal_ganglia	12	*RPS26*	56436876	rs11172049	0.9128	57304203	T	C	0.35	1.15E-02	3.68E-02	2.04E-01
rs4526799	Brain_Putamen_basal_ganglia	12	*NABP2*	56619718	rs11172049	0.9128	57304203	T	C	0.35	1.15E-02	3.93E-02	2.06E-01
rs4526799	Brain_Putamen_basal_ganglia	12	*RP11-977G19.14*	56662806	rs11172049	0.9128	57304203	T	C	0.35	1.15E-02	2.16E-02	1.89E-01
rs4526799	Brain_Putamen_basal_ganglia	12	*IL23A*	56733428	rs11172049	0.9128	57304203	T	C	0.35	1.15E-02	4.21E-03	1.62E-01
rs4526799	Brain_Putamen_basal_ganglia	12	*NAB2*	57485933	rs11172049	0.9128	57304203	T	C	0.35	1.15E-02	4.16E-02	2.08E-01
rs4526799	Brain_Putamen_basal_ganglia	12	*INHBE*	57849584	rs11172049	0.9128	57304203	T	C	0.35	1.15E-02	3.82E-02	2.05E-01
rs4526799	Brain_Putamen_basal_ganglia	12	*DTX3*	58000996	rs11172049	0.9128	57304203	T	C	0.35	1.15E-02	3.73E-02	2.04E-01
rs4526799	Brain_Putamen_basal_ganglia	12	*AVIL*	58201929	rs11172049	0.9128	57304203	T	C	0.35	1.15E-02	2.68E-02	1.94E-01
rs4526799	Brain_Putamen_basal_ganglia	12	*WIBG*	56310770	rs1874888	0.9085	57305138	A	C	0.35	1.15E-02	2.53E-02	1.93E-01
rs4526799	Brain_Putamen_basal_ganglia	12	*RPS26*	56436876	rs1874888	0.9085	57305138	A	C	0.35	1.15E-02	3.68E-02	2.04E-01
rs4526799	Brain_Putamen_basal_ganglia	12	*NABP2*	56619718	rs1874888	0.9085	57305138	A	C	0.35	1.15E-02	3.93E-02	2.06E-01
rs4526799	Brain_Putamen_basal_ganglia	12	*RP11-977G19.14*	56662806	rs1874888	0.9085	57305138	A	C	0.35	1.15E-02	2.16E-02	1.89E-01

rs4526799	Brain_Putamen_basal_ganglia	12	*IL23A*	56733428	rs1874888	0.9085	57305138	A	C	0.35	1.15E-02	4.21E-03	1.62E-01
rs4526799	Brain_Putamen_basal_ganglia	12	*NAB2*	57485933	rs1874888	0.9085	57305138	A	C	0.35	1.15E-02	4.16E-02	2.08E-01
rs4526799	Brain_Putamen_basal_ganglia	12	*INHBE*	57849584	rs1874888	0.9085	57305138	A	C	0.35	1.15E-02	3.82E-02	2.05E-01
rs4526799	Brain_Putamen_basal_ganglia	12	*DTX3*	58000996	rs1874888	0.9085	57305138	A	C	0.35	1.15E-02	3.73E-02	2.04E-01
rs4526799	Brain_Putamen_basal_ganglia	12	*AVIL*	58201929	rs1874888	0.9085	57305138	A	C	0.35	1.15E-02	2.68E-02	1.94E-01
rs4526799	Brain_Putamen_basal_ganglia	12	*WIBG*	56310770	rs10506349	0.9128	57306412	T	C	0.37	1.10E-02	3.00E-02	1.88E-01
rs4526799	Brain_Putamen_basal_ganglia	12	*RP11-977G19.5*	56570105	rs10506349	0.9128	57306412	T	C	0.37	1.10E-02	4.95E-02	2.06E-01
rs4526799	Brain_Putamen_basal_ganglia	12	*NABP2*	56619718	rs10506349	0.9128	57306412	T	C	0.37	1.10E-02	1.24E-02	1.68E-01
rs4526799	Brain_Putamen_basal_ganglia	12	*RP11-977G19.14*	56662806	rs10506349	0.9128	57306412	T	C	0.37	1.10E-02	9.77E-03	1.63E-01
rs4526799	Brain_Putamen_basal_ganglia	12	*RP11-977G19.11*	56701259	rs10506349	0.9128	57306412	T	C	0.37	1.10E-02	4.00E-02	1.97E-01
rs4526799	Brain_Putamen_basal_ganglia	12	*IL23A*	56733428	rs10506349	0.9128	57306412	T	C	0.37	1.10E-02	6.50E-03	1.57E-01
rs4526799	Brain_Putamen_basal_ganglia	12	*SDR9C7*	57322563	rs10506349	0.9128	57306412	T	C	0.37	1.10E-02	2.15E-02	1.79E-01
rs4526799	Brain_Putamen_basal_ganglia	12	*INHBE*	57849584	rs10506349	0.9128	57306412	T	C	0.37	1.10E-02	2.56E-02	1.84E-01
rs4526799	Brain_Putamen_basal_ganglia	12	*AVIL*	58201929	rs10506349	0.9128	57306412	T	C	0.37	1.10E-02	2.19E-02	1.80E-01
rs4526799	Brain_Putamen_basal_ganglia	12	*WIBG*	56310770	rs10876951	0.9085	57306430	T	G	0.35	1.15E-02	2.53E-02	1.93E-01
rs4526799	Brain_Putamen_basal_ganglia	12	*RPS26*	56436876	rs10876951	0.9085	57306430	T	G	0.35	1.15E-02	3.68E-02	2.04E-01
rs4526799	Brain_Putamen_basal_ganglia	12	*NABP2*	56619718	rs10876951	0.9085	57306430	T	G	0.35	1.15E-02	3.93E-02	2.06E-01
rs4526799	Brain_Putamen_basal_ganglia	12	*RP11-977G19.14*	56662806	rs10876951	0.9085	57306430	T	G	0.35	1.15E-02	2.16E-02	1.89E-01
rs4526799	Brain_Putamen_basal_ganglia	12	*IL23A*	56733428	rs10876951	0.9085	57306430	T	G	0.35	1.15E-02	4.21E-03	1.62E-01
rs4526799	Brain_Putamen_basal_ganglia	12	*NAB2*	57485933	rs10876951	0.9085	57306430	T	G	0.35	1.15E-02	4.16E-02	2.08E-01
rs4526799	Brain_Putamen_basal_ganglia	12	*INHBE*	57849584	rs10876951	0.9085	57306430	T	G	0.35	1.15E-02	3.82E-02	2.05E-01
rs4526799	Brain_Putamen_basal_ganglia	12	*DTX3*	58000996	rs10876951	0.9085	57306430	T	G	0.35	1.15E-02	3.73E-02	2.04E-01
rs4526799	Brain_Putamen_basal_ganglia	12	*AVIL*	58201929	rs10876951	0.9085	57306430	T	G	0.35	1.15E-02	2.68E-02	1.94E-01
rs4526799	Brain_Putamen_basal_ganglia	12	*WIBG*	56310770	rs10747774	0.9128	57307079	T	C	0.36	1.16E-02	3.71E-02	2.00E-01
rs4526799	Brain_Putamen_basal_ganglia	12	*NABP2*	56619718	rs10747774	0.9128	57307079	T	C	0.36	1.16E-02	1.09E-02	1.71E-01
rs4526799	Brain_Putamen_basal_ganglia	12	*RP11-977G19.14*	56662806	rs10747774	0.9128	57307079	T	C	0.36	1.16E-02	1.08E-02	1.70E-01
rs4526799	Brain_Putamen_basal_ganglia	12	*RP11-977G19.11*	56701259	rs10747774	0.9128	57307079	T	C	0.36	1.16E-02	4.24E-02	2.05E-01
rs4526799	Brain_Putamen_basal_ganglia	12	*IL23A*	56733428	rs10747774	0.9128	57307079	T	C	0.36	1.16E-02	7.44E-03	1.65E-01
rs4526799	Brain_Putamen_basal_ganglia	12	*SDR9C7*	57322563	rs10747774	0.9128	57307079	T	C	0.36	1.16E-02	2.23E-02	1.85E-01
rs4526799	Brain_Putamen_basal_ganglia	12	*INHBE*	57849584	rs10747774	0.9128	57307079	T	C	0.36	1.16E-02	2.48E-02	1.88E-01
rs4526799	Brain_Putamen_basal_ganglia	12	*AVIL*	58201929	rs10747774	0.9128	57307079	T	C	0.36	1.16E-02	2.35E-02	1.87E-01
rs4526799	Brain_Putamen_basal_ganglia	12	*WIBG*	56310770	rs10783812	0.9085	57308723	C	T	0.35	1.19E-02	2.24E-02	1.92E-01
rs4526799	Brain_Putamen_basal_ganglia	12	*RPS26*	56436876	rs10783812	0.9085	57308723	C	T	0.35	1.19E-02	3.56E-02	2.05E-01
rs4526799	Brain_Putamen_basal_ganglia	12	*NABP2*	56619718	rs10783812	0.9085	57308723	C	T	0.35	1.19E-02	4.26E-02	2.11E-01
rs4526799	Brain_Putamen_basal_ganglia	12	*RP11-977G19.14*	56662806	rs10783812	0.9085	57308723	C	T	0.35	1.19E-02	2.03E-02	1.90E-01
rs4526799	Brain_Putamen_basal_ganglia	12	*IL23A*	56733428	rs10783812	0.9085	57308723	C	T	0.35	1.19E-02	3.65E-03	1.64E-01
rs4526799	Brain_Putamen_basal_ganglia	12	*NAB2*	57485933	rs10783812	0.9085	57308723	C	T	0.35	1.19E-02	4.52E-02	2.13E-01
rs4526799	Brain_Putamen_basal_ganglia	12	*INHBE*	57849584	rs10783812	0.9085	57308723	C	T	0.35	1.19E-02	4.06E-02	2.10E-01
rs4526799	Brain_Putamen_basal_ganglia	12	*DTX3*	58000996	rs10783812	0.9085	57308723	C	T	0.35	1.19E-02	3.61E-02	2.06E-01
rs4526799	Brain_Putamen_basal_ganglia	12	*AVIL*	58201929	rs10783812	0.9085	57308723	C	T	0.35	1.19E-02	3.10E-02	2.01E-01

rs4526799	Brain_Putamen_basal_ganglia	12	*WIBG*	56310770	rs11172056	0.9089	57308975	C	T	0.37	1.10E-02	3.71E-02	1.95E-01
rs4526799	Brain_Putamen_basal_ganglia	12	*NABP2*	56619718	rs11172056	0.9089	57308975	C	T	0.37	1.10E-02	1.09E-02	1.65E-01
rs4526799	Brain_Putamen_basal_ganglia	12	*RP11-977G19.14*	56662806	rs11172056	0.9089	57308975	C	T	0.37	1.10E-02	1.08E-02	1.65E-01
rs4526799	Brain_Putamen_basal_ganglia	12	*RP11-977G19.11*	56701259	rs11172056	0.9089	57308975	C	T	0.37	1.10E-02	4.24E-02	2.00E-01
rs4526799	Brain_Putamen_basal_ganglia	12	*IL23A*	56733428	rs11172056	0.9089	57308975	C	T	0.37	1.10E-02	7.44E-03	1.59E-01
rs4526799	Brain_Putamen_basal_ganglia	12	*SDR9C7*	57322563	rs11172056	0.9089	57308975	C	T	0.37	1.10E-02	2.23E-02	1.80E-01
rs4526799	Brain_Putamen_basal_ganglia	12	*INHBE*	57849584	rs11172056	0.9089	57308975	C	T	0.37	1.10E-02	2.48E-02	1.83E-01
rs4526799	Brain_Putamen_basal_ganglia	12	*AVIL*	58201929	rs11172056	0.9089	57308975	C	T	0.37	1.10E-02	2.35E-02	1.82E-01
rs4526799	Brain_Putamen_basal_ganglia	12	*NABP2*	56619718	rs7302420	0.9085	57309884	G	C	0.36	1.54E-02	1.15E-02	1.96E-01
rs4526799	Brain_Putamen_basal_ganglia	12	*RP11-977G19.14*	56662806	rs7302420	0.9085	57309884	G	C	0.36	1.54E-02	2.10E-02	2.07E-01
rs4526799	Brain_Putamen_basal_ganglia	12	*IL23A*	56733428	rs7302420	0.9085	57309884	G	C	0.36	1.54E-02	5.87E-03	1.86E-01
rs4526799	Brain_Putamen_basal_ganglia	12	*SDR9C7*	57322563	rs7302420	0.9085	57309884	G	C	0.36	1.54E-02	1.44E-02	1.99E-01
rs4526799	Brain_Putamen_basal_ganglia	12	*NAB2*	57485933	rs7302420	0.9085	57309884	G	C	0.36	1.54E-02	3.23E-02	2.18E-01
rs4526799	Brain_Putamen_basal_ganglia	12	*INHBE*	57849584	rs7302420	0.9085	57309884	G	C	0.36	1.54E-02	2.29E-02	2.09E-01
rs4526799	Brain_Putamen_basal_ganglia	12	*DTX3*	58000996	rs7302420	0.9085	57309884	G	C	0.36	1.54E-02	3.92E-02	2.24E-01
rs4526799	Brain_Putamen_basal_ganglia	12	*AVIL*	58201929	rs7302420	0.9085	57309884	G	C	0.36	1.54E-02	2.05E-02	2.07E-01
rs4526799	Brain_Putamen_basal_ganglia	12	*WIBG*	56310770	rs12228618	0.9128	57311229	T	C	0.37	1.29E-02	4.31E-02	2.09E-01
rs4526799	Brain_Putamen_basal_ganglia	12	*CDK2*	56363560	rs12228618	0.9128	57311229	T	C	0.37	1.29E-02	4.36E-02	2.09E-01
rs4526799	Brain_Putamen_basal_ganglia	12	*RPS26*	56436876	rs12228618	0.9128	57311229	T	C	0.37	1.29E-02	3.66E-02	2.03E-01
rs4526799	Brain_Putamen_basal_ganglia	12	*NABP2*	56619718	rs12228618	0.9128	57311229	T	C	0.37	1.29E-02	1.37E-02	1.78E-01
rs4526799	Brain_Putamen_basal_ganglia	12	*RP11-977G19.14*	56662806	rs12228618	0.9128	57311229	T	C	0.37	1.29E-02	2.20E-02	1.89E-01
rs4526799	Brain_Putamen_basal_ganglia	12	*RP11-977G19.11*	56701259	rs12228618	0.9128	57311229	T	C	0.37	1.29E-02	4.76E-02	2.12E-01
rs4526799	Brain_Putamen_basal_ganglia	12	*IL23A*	56733428	rs12228618	0.9128	57311229	T	C	0.37	1.29E-02	1.17E-02	1.76E-01
rs4526799	Brain_Putamen_basal_ganglia	12	*SDR9C7*	57322563	rs12228618	0.9128	57311229	T	C	0.37	1.29E-02	1.94E-02	1.86E-01
rs4526799	Brain_Putamen_basal_ganglia	12	*INHBE*	57849584	rs12228618	0.9128	57311229	T	C	0.37	1.29E-02	3.82E-02	2.04E-01
rs4526799	Brain_Putamen_basal_ganglia	12	*AVIL*	58201929	rs12228618	0.9128	57311229	T	C	0.37	1.29E-02	3.12E-02	1.98E-01
rs4526799	Brain_Putamen_basal_ganglia	12	*RPS26*	56436876	rs9739473	0.9012	57313335	A	T	0.37	2.65E-02	4.26E-02	2.73E-01
rs4526799	Brain_Putamen_basal_ganglia	12	*NABP2*	56619718	rs9739473	0.9012	57313335	A	T	0.37	2.65E-02	9.50E-03	2.44E-01
rs4526799	Brain_Putamen_basal_ganglia	12	*RP11-977G19.14*	56662806	rs9739473	0.9012	57313335	A	T	0.37	2.65E-02	2.73E-02	2.62E-01
rs4526799	Brain_Putamen_basal_ganglia	12	*RP11-977G19.11*	56701259	rs9739473	0.9012	57313335	A	T	0.37	2.65E-02	4.05E-02	2.72E-01
rs4526799	Brain_Putamen_basal_ganglia	12	*IL23A*	56733428	rs9739473	0.9012	57313335	A	T	0.37	2.65E-02	1.26E-02	2.48E-01
rs4526799	Brain_Putamen_basal_ganglia	12	*SDR9C7*	57322563	rs9739473	0.9012	57313335	A	T	0.37	2.65E-02	3.24E-02	2.66E-01
rs4526799	Brain_Putamen_basal_ganglia	12	*NAB2*	57485933	rs9739473	0.9012	57313335	A	T	0.37	2.65E-02	3.96E-02	2.71E-01
rs4526799	Brain_Putamen_basal_ganglia	12	*INHBE*	57849584	rs9739473	0.9012	57313335	A	T	0.37	2.65E-02	2.51E-02	2.60E-01
rs4526799	Brain_Putamen_basal_ganglia	12	*AVIL*	58201929	rs9739473	0.9012	57313335	A	T	0.37	2.65E-02	2.72E-02	2.62E-01
rs4526799	Brain_Spinal_cord_cervical_c-1	12	*RNF41*	56607001	rs9919772	0.8450	57260027	T	C	0.33	1.03E-02	3.70E-02	2.25E-01
rs4526799	Brain_Spinal_cord_cervical_c-1	12	*STAC3*	57641106	rs9919772	0.8450	57260027	T	C	0.33	1.03E-02	3.86E-02	2.27E-01
rs4526799	Brain_Spinal_cord_cervical_c-1	12	*CYP27B1*	58158578	rs9919772	0.8450	57260027	T	C	0.33	1.03E-02	2.70E-02	2.17E-01
rs4526799	Brain_Spinal_cord_cervical_c-1	12	*RAB5B*	56378093	rs4495925	0.8650	57268116	C	G	0.33	1.04E-02	4.10E-02	2.26E-01
rs4526799	Brain_Spinal_cord_cervical_c-1	12	*RNF41*	56607001	rs4495925	0.8650	57268116	C	G	0.33	1.04E-02	4.41E-02	2.28E-01

rs4526799	Brain_Spinal_cord_cervical_c-1	12	*CYP27B1*	58158578	rs4495925	0.8650	57268116	C	G	0.33	1.04E-02	2.55E-02	2.12E-01
rs4526799	Brain_Spinal_cord_cervical_c-1	12	*RAB5B*	56378093	rs4471472	0.8650	57268985	A	G	0.32	9.99E-03	4.29E-02	2.24E-01
rs4526799	Brain_Spinal_cord_cervical_c-1	12	*RNF41*	56607001	rs4471472	0.8650	57268985	A	G	0.32	9.99E-03	3.97E-02	2.21E-01
rs4526799	Brain_Spinal_cord_cervical_c-1	12	*CYP27B1*	58158578	rs4471472	0.8650	57268985	A	G	0.32	9.99E-03	2.05E-02	2.03E-01
rs4526799	Brain_Spinal_cord_cervical_c-1	12	*RAB5B*	56378093	rs4633499	0.8610	57269264	A	T	0.32	1.43E-02	3.97E-02	2.55E-01
rs4526799	Brain_Spinal_cord_cervical_c-1	12	*STAC3*	57641106	rs4633499	0.8610	57269264	A	T	0.32	1.43E-02	4.24E-02	2.57E-01
rs4526799	Brain_Spinal_cord_cervical_c-1	12	*CYP27B1*	58158578	rs4633499	0.8610	57269264	A	T	0.32	1.43E-02	2.86E-02	2.46E-01
rs4526799	Brain_Spinal_cord_cervical_c-1	12	*ESYT1*	56530147	rs12300079	0.9956	57273194	T	C	0.36	3.73E-03	4.85E-02	1.82E-01
rs4526799	Brain_Spinal_cord_cervical_c-1	12	*TAC3*	57413225	rs12300079	0.9956	57273194	T	C	0.36	3.73E-03	3.04E-02	1.65E-01
rs4526799	Brain_Spinal_cord_cervical_c-1	12	*ESYT1*	56530147	rs12300191	0.9956	57273289	A	G	0.36	3.59E-03	4.85E-02	1.80E-01
rs4526799	Brain_Spinal_cord_cervical_c-1	12	*TAC3*	57413225	rs12300191	0.9956	57273289	A	G	0.36	3.59E-03	3.04E-02	1.63E-01
rs4526799	Brain_Spinal_cord_cervical_c-1	12	*ESYT1*	56530147	rs4514464	0.9956	57276375	C	T	0.36	2.92E-03	4.85E-02	1.77E-01
rs4526799	Brain_Spinal_cord_cervical_c-1	12	*TAC3*	57413225	rs4514464	0.9956	57276375	C	T	0.36	2.92E-03	3.04E-02	1.60E-01
rs4526799	Brain_Spinal_cord_cervical_c-1	12	*ESYT1*	56530147	rs4417325	0.9956	57277302	G	A	0.36	3.59E-03	4.85E-02	1.80E-01
rs4526799	Brain_Spinal_cord_cervical_c-1	12	*TAC3*	57413225	rs4417325	0.9956	57277302	G	A	0.36	3.59E-03	3.04E-02	1.63E-01
rs4526799	Brain_Spinal_cord_cervical_c-1	12	*ESYT1*	56530147	rs11172030	0.9956	57278076	A	C	0.36	3.63E-03	4.85E-02	1.81E-01
rs4526799	Brain_Spinal_cord_cervical_c-1	12	*TAC3*	57413225	rs11172030	0.9956	57278076	A	C	0.36	3.63E-03	3.04E-02	1.63E-01
rs4526799	Brain_Spinal_cord_cervical_c-1	12	*ESYT1*	56530147	rs10876944	0.9956	57279372	T	A	0.36	3.63E-03	4.85E-02	1.81E-01
rs4526799	Brain_Spinal_cord_cervical_c-1	12	*TAC3*	57413225	rs10876944	0.9956	57279372	T	A	0.36	3.63E-03	3.04E-02	1.63E-01
rs4526799	Brain_Spinal_cord_cervical_c-1	12	*ESYT1*	56530147	rs4326839	0.9956	57280374	G	C	0.36	3.58E-03	4.85E-02	1.80E-01
rs4526799	Brain_Spinal_cord_cervical_c-1	12	*TAC3*	57413225	rs4326839	0.9956	57280374	G	C	0.36	3.58E-03	3.04E-02	1.63E-01
rs4526799	Brain_Spinal_cord_cervical_c-1	12	*ESYT1*	56530147	rs4526799	1.0000	57280586	T	C	0.34	7.26E-06	4.85E-02	8.46E-02
rs4526799	Brain_Spinal_cord_cervical_c-1	12	*TAC3*	57413225	rs4526799	1.0000	57280586	T	C	0.34	7.26E-06	3.04E-02	6.46E-02
rs4526799	Brain_Spinal_cord_cervical_c-1	12	*ESYT1*	56530147	rs28876529	0.9956	57285301	T	A	0.36	2.25E-03	4.85E-02	1.65E-01
rs4526799	Brain_Spinal_cord_cervical_c-1	12	*TAC3*	57413225	rs28876529	0.9956	57285301	T	A	0.36	2.25E-03	3.04E-02	1.47E-01
rs4526799	Brain_Spinal_cord_cervical_c-1	12	*ESYT1*	56530147	rs11172037	0.9956	57285427	T	A	0.36	2.25E-03	4.85E-02	1.65E-01
rs4526799	Brain_Spinal_cord_cervical_c-1	12	*TAC3*	57413225	rs11172037	0.9956	57285427	T	A	0.36	2.25E-03	3.04E-02	1.47E-01
rs4526799	Brain_Spinal_cord_cervical_c-1	12	*ESYT1*	56530147	rs12321987	0.9956	57288449	G	A	0.36	4.47E-03	4.85E-02	2.00E-01
rs4526799	Brain_Spinal_cord_cervical_c-1	12	*TAC3*	57413225	rs12321987	0.9956	57288449	G	A	0.36	4.47E-03	3.04E-02	1.84E-01
rs4526799	Brain_Spinal_cord_cervical_c-1	12	*NABP2*	56619718	rs11172043	0.9869	57293182	G	A	0.35	4.58E-03	3.76E-02	1.89E-01
rs4526799	Brain_Spinal_cord_cervical_c-1	12	*TAC3*	57413225	rs11172043	0.9869	57293182	G	A	0.35	4.58E-03	1.90E-02	1.70E-01
rs4526799	Brain_Spinal_cord_cervical_c-1	12	*NABP2*	56619718	rs12426816	0.9869	57294074	A	C	0.35	4.50E-03	3.76E-02	1.89E-01
rs4526799	Brain_Spinal_cord_cervical_c-1	12	*TAC3*	57413225	rs12426816	0.9869	57294074	A	C	0.35	4.50E-03	1.90E-02	1.70E-01
rs4526799	Brain_Spinal_cord_cervical_c-1	12	*NABP2*	56619718	rs11172047	0.9869	57298080	T	C	0.35	6.51E-03	3.76E-02	2.03E-01
rs4526799	Brain_Spinal_cord_cervical_c-1	12	*TAC3*	57413225	rs11172047	0.9869	57298080	T	C	0.35	6.51E-03	1.90E-02	1.84E-01
rs4526799	Brain_Spinal_cord_cervical_c-1	12	*NABP2*	56619718	rs2371631	0.9869	57298614	T	A	0.35	6.32E-03	3.76E-02	2.00E-01
rs4526799	Brain_Spinal_cord_cervical_c-1	12	*TAC3*	57413225	rs2371631	0.9869	57298614	T	A	0.35	6.32E-03	1.90E-02	1.81E-01
rs4526799	Brain_Spinal_cord_cervical_c-1	12	*NABP2*	56619718	rs12305763	0.9869	57299263	G	A	0.35	6.82E-03	3.76E-02	2.04E-01
rs4526799	Brain_Spinal_cord_cervical_c-1	12	*TAC3*	57413225	rs12305763	0.9869	57299263	G	A	0.35	6.82E-03	1.90E-02	1.85E-01
rs4526799	Brain_Spinal_cord_cervical_c-1	12	*RAB5B*	56378093	rs11172049	0.9128	57304203	T	C	0.35	1.15E-02	3.24E-02	1.99E-01

rs4526799	Brain_Spinal_cord_cervical_c-1	12	*TAC3*	57413225	rs11172049	0.9128	57304203	T	C	0.35	1.15E-02	3.25E-03	1.59E-01
rs4526799	Brain_Spinal_cord_cervical_c-1	12	*STAC3*	57641106	rs11172049	0.9128	57304203	T	C	0.35	1.15E-02	3.15E-02	1.99E-01
rs4526799	Brain_Spinal_cord_cervical_c-1	12	*CYP27B1*	58158578	rs11172049	0.9128	57304203	T	C	0.35	1.15E-02	3.41E-02	2.01E-01
rs4526799	Brain_Spinal_cord_cervical_c-1	12	*RAB5B*	56378093	rs1874888	0.9085	57305138	A	C	0.35	1.15E-02	3.24E-02	1.99E-01
rs4526799	Brain_Spinal_cord_cervical_c-1	12	*TAC3*	57413225	rs1874888	0.9085	57305138	A	C	0.35	1.15E-02	3.25E-03	1.59E-01
rs4526799	Brain_Spinal_cord_cervical_c-1	12	*STAC3*	57641106	rs1874888	0.9085	57305138	A	C	0.35	1.15E-02	3.15E-02	1.99E-01
rs4526799	Brain_Spinal_cord_cervical_c-1	12	*CYP27B1*	58158578	rs1874888	0.9085	57305138	A	C	0.35	1.15E-02	3.41E-02	2.01E-01
rs4526799	Brain_Spinal_cord_cervical_c-1	12	*RAB5B*	56378093	rs10506349	0.9128	57306412	T	C	0.37	1.10E-02	2.50E-02	1.83E-01
rs4526799	Brain_Spinal_cord_cervical_c-1	12	*TAC3*	57413225	rs10506349	0.9128	57306412	T	C	0.37	1.10E-02	5.05E-03	1.54E-01
rs4526799	Brain_Spinal_cord_cervical_c-1	12	*STAC3*	57641106	rs10506349	0.9128	57306412	T	C	0.37	1.10E-02	3.33E-02	1.91E-01
rs4526799	Brain_Spinal_cord_cervical_c-1	12	*RAB5B*	56378093	rs10876951	0.9085	57306430	T	G	0.35	1.15E-02	3.24E-02	1.99E-01
rs4526799	Brain_Spinal_cord_cervical_c-1	12	*TAC3*	57413225	rs10876951	0.9085	57306430	T	G	0.35	1.15E-02	3.25E-03	1.59E-01
rs4526799	Brain_Spinal_cord_cervical_c-1	12	*STAC3*	57641106	rs10876951	0.9085	57306430	T	G	0.35	1.15E-02	3.15E-02	1.99E-01
rs4526799	Brain_Spinal_cord_cervical_c-1	12	*CYP27B1*	58158578	rs10876951	0.9085	57306430	T	G	0.35	1.15E-02	3.41E-02	2.01E-01
rs4526799	Brain_Spinal_cord_cervical_c-1	12	*RAB5B*	56378093	rs10747774	0.9128	57307079	T	C	0.36	1.16E-02	2.50E-02	1.88E-01
rs4526799	Brain_Spinal_cord_cervical_c-1	12	*TAC3*	57413225	rs10747774	0.9128	57307079	T	C	0.36	1.16E-02	5.05E-03	1.60E-01
rs4526799	Brain_Spinal_cord_cervical_c-1	12	*STAC3*	57641106	rs10747774	0.9128	57307079	T	C	0.36	1.16E-02	3.33E-02	1.96E-01
rs4526799	Brain_Spinal_cord_cervical_c-1	12	*RAB5B*	56378093	rs10783812	0.9085	57308723	C	T	0.35	1.19E-02	3.34E-02	2.03E-01
rs4526799	Brain_Spinal_cord_cervical_c-1	12	*TAC3*	57413225	rs10783812	0.9085	57308723	C	T	0.35	1.19E-02	2.85E-03	1.61E-01
rs4526799	Brain_Spinal_cord_cervical_c-1	12	*STAC3*	57641106	rs10783812	0.9085	57308723	C	T	0.35	1.19E-02	2.78E-02	1.98E-01
rs4526799	Brain_Spinal_cord_cervical_c-1	12	*CYP27B1*	58158578	rs10783812	0.9085	57308723	C	T	0.35	1.19E-02	3.37E-02	2.04E-01
rs4526799	Brain_Spinal_cord_cervical_c-1	12	*RAB5B*	56378093	rs11172056	0.9089	57308975	C	T	0.37	1.10E-02	2.50E-02	1.83E-01
rs4526799	Brain_Spinal_cord_cervical_c-1	12	*TAC3*	57413225	rs11172056	0.9089	57308975	C	T	0.37	1.10E-02	5.05E-03	1.54E-01
rs4526799	Brain_Spinal_cord_cervical_c-1	12	*STAC3*	57641106	rs11172056	0.9089	57308975	C	T	0.37	1.10E-02	3.33E-02	1.92E-01
rs4526799	Brain_Spinal_cord_cervical_c-1	12	*RAB5B*	56378093	rs7302420	0.9085	57309884	G	C	0.36	1.54E-02	3.37E-02	2.19E-01
rs4526799	Brain_Spinal_cord_cervical_c-1	12	*TAC3*	57413225	rs7302420	0.9085	57309884	G	C	0.36	1.54E-02	5.12E-03	1.85E-01
rs4526799	Brain_Spinal_cord_cervical_c-1	12	*STAC3*	57641106	rs7302420	0.9085	57309884	G	C	0.36	1.54E-02	3.04E-02	2.16E-01
rs4526799	Brain_Spinal_cord_cervical_c-1	12	*CYP27B1*	58158578	rs7302420	0.9085	57309884	G	C	0.36	1.54E-02	3.19E-02	2.18E-01
rs4526799	Brain_Spinal_cord_cervical_c-1	12	*RAB5B*	56378093	rs12228618	0.9128	57311229	T	C	0.37	1.29E-02	3.39E-02	2.01E-01
rs4526799	Brain_Spinal_cord_cervical_c-1	12	*ESYT1*	56530147	rs12228618	0.9128	57311229	T	C	0.37	1.29E-02	2.76E-02	1.94E-01
rs4526799	Brain_Spinal_cord_cervical_c-1	12	*TAC3*	57413225	rs12228618	0.9128	57311229	T	C	0.37	1.29E-02	6.93E-03	1.68E-01
rs4526799	Brain_Spinal_cord_cervical_c-1	12	*STAC3*	57641106	rs12228618	0.9128	57311229	T	C	0.37	1.29E-02	2.77E-02	1.95E-01
rs4526799	Brain_Spinal_cord_cervical_c-1	12	*RAB5B*	56378093	rs9739473	0.9012	57313335	A	T	0.37	2.65E-02	3.39E-02	2.67E-01
rs4526799	Brain_Spinal_cord_cervical_c-1	12	*ESYT1*	56530147	rs9739473	0.9012	57313335	A	T	0.37	2.65E-02	2.76E-02	2.62E-01
rs4526799	Brain_Spinal_cord_cervical_c-1	12	*TAC3*	57413225	rs9739473	0.9012	57313335	A	T	0.37	2.65E-02	6.93E-03	2.41E-01
rs4526799	Brain_Spinal_cord_cervical_c-1	12	*STAC3*	57641106	rs9739473	0.9012	57313335	A	T	0.37	2.65E-02	2.77E-02	2.62E-01
rs4526799	Brain_Substantia_nigra	12	*RP11-153M3.1*	56905636	rs9919772	0.8450	57260027	T	C	0.33	1.03E-02	2.75E-02	2.17E-01
rs4526799	Brain_Substantia_nigra	12	*ZBTB39*	57396424	rs9919772	0.8450	57260027	T	C	0.33	1.03E-02	2.81E-02	2.18E-01
rs4526799	Brain_Substantia_nigra	12	*METTL21B*	58170856	rs9919772	0.8450	57260027	T	C	0.33	1.03E-02	3.96E-02	2.28E-01
rs4526799	Brain_Substantia_nigra	12	*RP11-603J24.7*	56374517	rs4495925	0.8650	57268116	C	G	0.33	1.04E-02	4.92E-02	2.32E-01

rs4526799	Brain_Substantia_nigra	12	*RP11-153M3.1*	56905636	rs4495925	0.8650	57268116	C	G	0.33	1.04E-02	4.02E-02	2.25E-01
rs4526799	Brain_Substantia_nigra	12	*ZBTB39*	57396424	rs4495925	0.8650	57268116	C	G	0.33	1.04E-02	2.39E-02	2.11E-01
rs4526799	Brain_Substantia_nigra	12	*CYP27B1*	58158578	rs4495925	0.8650	57268116	C	G	0.33	1.04E-02	4.87E-02	2.32E-01
rs4526799	Brain_Substantia_nigra	12	*METTL21B*	58170856	rs4495925	0.8650	57268116	C	G	0.33	1.04E-02	4.44E-02	2.29E-01
rs4526799	Brain_Substantia_nigra	12	*RP11-603J24.7*	56374517	rs4471472	0.8650	57268985	A	G	0.32	9.99E-03	4.94E-02	2.29E-01
rs4526799	Brain_Substantia_nigra	12	*RP11-153M3.1*	56905636	rs4471472	0.8650	57268985	A	G	0.32	9.99E-03	3.20E-02	2.15E-01
rs4526799	Brain_Substantia_nigra	12	*ZBTB39*	57396424	rs4471472	0.8650	57268985	A	G	0.32	9.99E-03	1.86E-02	2.01E-01
rs4526799	Brain_Substantia_nigra	12	*METTL21B*	58170856	rs4471472	0.8650	57268985	A	G	0.32	9.99E-03	4.14E-02	2.23E-01
rs4526799	Brain_Substantia_nigra	12	*RP11-603J24.7*	56374517	rs4633499	0.8610	57269264	A	T	0.32	1.43E-02	4.26E-02	2.57E-01
rs4526799	Brain_Substantia_nigra	12	*METTL21B*	58170856	rs4633499	0.8610	57269264	A	T	0.32	1.43E-02	4.25E-02	2.57E-01
rs4526799	Brain_Substantia_nigra	12	*RP11-603J24.7*	56374517	rs12300079	0.9956	57273194	T	C	0.36	3.73E-03	3.56E-02	1.70E-01
rs4526799	Brain_Substantia_nigra	12	*MYL6*	56554355	rs12300079	0.9956	57273194	T	C	0.36	3.73E-03	3.69E-02	1.71E-01
rs4526799	Brain_Substantia_nigra	12	*RP11-153M3.1*	56905636	rs12300079	0.9956	57273194	T	C	0.36	3.73E-03	4.31E-02	1.77E-01
rs4526799	Brain_Substantia_nigra	12	*CYP27B1*	58158578	rs12300079	0.9956	57273194	T	C	0.36	3.73E-03	4.56E-02	1.80E-01
rs4526799	Brain_Substantia_nigra	12	*RP11-603J24.7*	56374517	rs12300191	0.9956	57273289	A	G	0.36	3.59E-03	3.56E-02	1.68E-01
rs4526799	Brain_Substantia_nigra	12	*MYL6*	56554355	rs12300191	0.9956	57273289	A	G	0.36	3.59E-03	3.69E-02	1.69E-01
rs4526799	Brain_Substantia_nigra	12	*RP11-153M3.1*	56905636	rs12300191	0.9956	57273289	A	G	0.36	3.59E-03	4.31E-02	1.75E-01
rs4526799	Brain_Substantia_nigra	12	*CYP27B1*	58158578	rs12300191	0.9956	57273289	A	G	0.36	3.59E-03	4.56E-02	1.77E-01
rs4526799	Brain_Substantia_nigra	12	*RP11-603J24.7*	56374517	rs4514464	0.9956	57276375	C	T	0.36	2.92E-03	3.56E-02	1.65E-01
rs4526799	Brain_Substantia_nigra	12	*MYL6*	56554355	rs4514464	0.9956	57276375	C	T	0.36	2.92E-03	3.69E-02	1.66E-01
rs4526799	Brain_Substantia_nigra	12	*RP11-153M3.1*	56905636	rs4514464	0.9956	57276375	C	T	0.36	2.92E-03	4.31E-02	1.72E-01
rs4526799	Brain_Substantia_nigra	12	*CYP27B1*	58158578	rs4514464	0.9956	57276375	C	T	0.36	2.92E-03	4.56E-02	1.74E-01
rs4526799	Brain_Substantia_nigra	12	*RP11-603J24.7*	56374517	rs4417325	0.9956	57277302	G	A	0.36	3.59E-03	3.56E-02	1.68E-01
rs4526799	Brain_Substantia_nigra	12	*MYL6*	56554355	rs4417325	0.9956	57277302	G	A	0.36	3.59E-03	3.69E-02	1.69E-01
rs4526799	Brain_Substantia_nigra	12	*RP11-153M3.1*	56905636	rs4417325	0.9956	57277302	G	A	0.36	3.59E-03	4.31E-02	1.75E-01
rs4526799	Brain_Substantia_nigra	12	*CYP27B1*	58158578	rs4417325	0.9956	57277302	G	A	0.36	3.59E-03	4.56E-02	1.77E-01
rs4526799	Brain_Substantia_nigra	12	*RP11-603J24.7*	56374517	rs11172030	0.9956	57278076	A	C	0.36	3.63E-03	3.56E-02	1.69E-01
rs4526799	Brain_Substantia_nigra	12	*MYL6*	56554355	rs11172030	0.9956	57278076	A	C	0.36	3.63E-03	3.69E-02	1.70E-01
rs4526799	Brain_Substantia_nigra	12	*RP11-153M3.1*	56905636	rs11172030	0.9956	57278076	A	C	0.36	3.63E-03	4.31E-02	1.76E-01
rs4526799	Brain_Substantia_nigra	12	*CYP27B1*	58158578	rs11172030	0.9956	57278076	A	C	0.36	3.63E-03	4.56E-02	1.78E-01
rs4526799	Brain_Substantia_nigra	12	*RP11-603J24.7*	56374517	rs10876944	0.9956	57279372	T	A	0.36	3.63E-03	3.56E-02	1.69E-01
rs4526799	Brain_Substantia_nigra	12	*MYL6*	56554355	rs10876944	0.9956	57279372	T	A	0.36	3.63E-03	3.69E-02	1.70E-01
rs4526799	Brain_Substantia_nigra	12	*RP11-153M3.1*	56905636	rs10876944	0.9956	57279372	T	A	0.36	3.63E-03	4.31E-02	1.76E-01
rs4526799	Brain_Substantia_nigra	12	*CYP27B1*	58158578	rs10876944	0.9956	57279372	T	A	0.36	3.63E-03	4.56E-02	1.78E-01
rs4526799	Brain_Substantia_nigra	12	*RP11-603J24.7*	56374517	rs4326839	0.9956	57280374	G	C	0.36	3.58E-03	3.56E-02	1.68E-01
rs4526799	Brain_Substantia_nigra	12	*MYL6*	56554355	rs4326839	0.9956	57280374	G	C	0.36	3.58E-03	3.69E-02	1.69E-01
rs4526799	Brain_Substantia_nigra	12	*RP11-153M3.1*	56905636	rs4326839	0.9956	57280374	G	C	0.36	3.58E-03	4.31E-02	1.75E-01
rs4526799	Brain_Substantia_nigra	12	*CYP27B1*	58158578	rs4326839	0.9956	57280374	G	C	0.36	3.58E-03	4.56E-02	1.77E-01
rs4526799	Brain_Substantia_nigra	12	*RP11-603J24.7*	56374517	rs4526799	1.0000	57280586	T	C	0.34	7.26E-06	3.56E-02	7.06E-02
rs4526799	Brain_Substantia_nigra	12	*MYL6*	56554355	rs4526799	1.0000	57280586	T	C	0.34	7.26E-06	3.69E-02	7.20E-02

rs4526799	Brain_Substantia_nigra	12	*RP11-153M3.1*	56905636	rs4526799	1.0000	57280586	T	C	0.34	7.26E-06	4.31E-02	7.88E-02
rs4526799	Brain_Substantia_nigra	12	*CYP27B1*	58158578	rs4526799	1.0000	57280586	T	C	0.34	7.26E-06	4.56E-02	8.15E-02
rs4526799	Brain_Substantia_nigra	12	*RP11-603J24.7*	56374517	rs28876529	0.9956	57285301	T	A	0.36	2.25E-03	3.56E-02	1.53E-01
rs4526799	Brain_Substantia_nigra	12	*MYL6*	56554355	rs28876529	0.9956	57285301	T	A	0.36	2.25E-03	3.69E-02	1.54E-01
rs4526799	Brain_Substantia_nigra	12	*RP11-153M3.1*	56905636	rs28876529	0.9956	57285301	T	A	0.36	2.25E-03	4.31E-02	1.60E-01
rs4526799	Brain_Substantia_nigra	12	*CYP27B1*	58158578	rs28876529	0.9956	57285301	T	A	0.36	2.25E-03	4.56E-02	1.63E-01
rs4526799	Brain_Substantia_nigra	12	*RP11-603J24.7*	56374517	rs11172037	0.9956	57285427	T	A	0.36	2.25E-03	3.56E-02	1.53E-01
rs4526799	Brain_Substantia_nigra	12	*MYL6*	56554355	rs11172037	0.9956	57285427	T	A	0.36	2.25E-03	3.69E-02	1.54E-01
rs4526799	Brain_Substantia_nigra	12	*RP11-153M3.1*	56905636	rs11172037	0.9956	57285427	T	A	0.36	2.25E-03	4.31E-02	1.60E-01
rs4526799	Brain_Substantia_nigra	12	*CYP27B1*	58158578	rs11172037	0.9956	57285427	T	A	0.36	2.25E-03	4.56E-02	1.63E-01
rs4526799	Brain_Substantia_nigra	12	*RP11-603J24.7*	56374517	rs12321987	0.9956	57288449	G	A	0.36	4.47E-03	3.56E-02	1.89E-01
rs4526799	Brain_Substantia_nigra	12	*MYL6*	56554355	rs12321987	0.9956	57288449	G	A	0.36	4.47E-03	3.69E-02	1.90E-01
rs4526799	Brain_Substantia_nigra	12	*RP11-153M3.1*	56905636	rs12321987	0.9956	57288449	G	A	0.36	4.47E-03	4.31E-02	1.96E-01
rs4526799	Brain_Substantia_nigra	12	*CYP27B1*	58158578	rs12321987	0.9956	57288449	G	A	0.36	4.47E-03	4.56E-02	1.98E-01
rs4526799	Brain_Substantia_nigra	12	*RP11-603J24.7*	56374517	rs11172043	0.9869	57293182	G	A	0.35	4.58E-03	4.53E-02	1.96E-01
rs4526799	Brain_Substantia_nigra	12	*RP11-603J24.7*	56374517	rs12426816	0.9869	57294074	A	C	0.35	4.50E-03	4.53E-02	1.96E-01
rs4526799	Brain_Substantia_nigra	12	*RP11-603J24.7*	56374517	rs11172047	0.9869	57298080	T	C	0.35	6.51E-03	4.53E-02	2.09E-01
rs4526799	Brain_Substantia_nigra	12	*RP11-603J24.7*	56374517	rs2371631	0.9869	57298614	T	A	0.35	6.32E-03	4.53E-02	2.06E-01
rs4526799	Brain_Substantia_nigra	12	*RP11-603J24.7*	56374517	rs12305763	0.9869	57299263	G	A	0.35	6.82E-03	4.53E-02	2.11E-01
rs4526799	Brain_Substantia_nigra	12	*RP11-153M3.1*	56905636	rs11172049	0.9128	57304203	T	C	0.35	1.15E-02	4.76E-02	2.13E-01
rs4526799	Brain_Substantia_nigra	12	*RP11-153M3.1*	56905636	rs1874888	0.9085	57305138	A	C	0.35	1.15E-02	4.76E-02	2.13E-01
rs4526799	Brain_Substantia_nigra	12	*RP11-603J24.7*	56374517	rs10506349	0.9128	57306412	T	C	0.37	1.10E-02	1.81E-02	1.75E-01
rs4526799	Brain_Substantia_nigra	12	*HSD17B6*	57163759	rs10506349	0.9128	57306412	T	C	0.37	1.10E-02	3.83E-02	1.96E-01
rs4526799	Brain_Substantia_nigra	12	*ZBTB39*	57396424	rs10506349	0.9128	57306412	T	C	0.37	1.10E-02	4.61E-02	2.03E-01
rs4526799	Brain_Substantia_nigra	12	*NXPH4*	57615405	rs10506349	0.9128	57306412	T	C	0.37	1.10E-02	4.16E-02	1.99E-01
rs4526799	Brain_Substantia_nigra	12	*CYP27B1*	58158578	rs10506349	0.9128	57306412	T	C	0.37	1.10E-02	1.47E-02	1.71E-01
rs4526799	Brain_Substantia_nigra	12	*RP11-153M3.1*	56905636	rs10876951	0.9085	57306430	T	G	0.35	1.15E-02	4.76E-02	2.13E-01
rs4526799	Brain_Substantia_nigra	12	*RP11-603J24.7*	56374517	rs10747774	0.9128	57307079	T	C	0.36	1.16E-02	1.81E-02	1.80E-01
rs4526799	Brain_Substantia_nigra	12	*HSD17B6*	57163759	rs10747774	0.9128	57307079	T	C	0.36	1.16E-02	3.83E-02	2.01E-01
rs4526799	Brain_Substantia_nigra	12	*ZBTB39*	57396424	rs10747774	0.9128	57307079	T	C	0.36	1.16E-02	4.61E-02	2.08E-01
rs4526799	Brain_Substantia_nigra	12	*NXPH4*	57615405	rs10747774	0.9128	57307079	T	C	0.36	1.16E-02	4.16E-02	2.04E-01
rs4526799	Brain_Substantia_nigra	12	*CYP27B1*	58158578	rs10747774	0.9128	57307079	T	C	0.36	1.16E-02	1.47E-02	1.76E-01
rs4526799	Brain_Substantia_nigra	12	*RP11-153M3.1*	56905636	rs10783812	0.9085	57308723	C	T	0.35	1.19E-02	4.76E-02	2.15E-01
rs4526799	Brain_Substantia_nigra	12	*RP11-603J24.7*	56374517	rs11172056	0.9089	57308975	C	T	0.37	1.10E-02	1.81E-02	1.75E-01
rs4526799	Brain_Substantia_nigra	12	*HSD17B6*	57163759	rs11172056	0.9089	57308975	C	T	0.37	1.10E-02	3.83E-02	1.96E-01
rs4526799	Brain_Substantia_nigra	12	*ZBTB39*	57396424	rs11172056	0.9089	57308975	C	T	0.37	1.10E-02	4.61E-02	2.03E-01
rs4526799	Brain_Substantia_nigra	12	*NXPH4*	57615405	rs11172056	0.9089	57308975	C	T	0.37	1.10E-02	4.16E-02	1.99E-01
rs4526799	Brain_Substantia_nigra	12	*CYP27B1*	58158578	rs11172056	0.9089	57308975	C	T	0.37	1.10E-02	1.47E-02	1.71E-01
rs4526799	Brain_Substantia_nigra	12	*CYP27B1*	58158578	rs7302420	0.9085	57309884	G	C	0.36	1.54E-02	4.51E-02	2.28E-01
rs4526799	Brain_Substantia_nigra	12	*RP11-603J24.7*	56374517	rs12228618	0.9128	57311229	T	C	0.37	1.29E-02	1.33E-02	1.78E-01

rs4526799	Brain_Substantia_nigra	12	*NXPH4*	57615405	rs12228618	0.9128	57311229	T	C	0.37	1.29E-02	2.73E-02	1.94E-01
rs4526799	Brain_Substantia_nigra	12	*CYP27B1*	58158578	rs12228618	0.9128	57311229	T	C	0.37	1.29E-02	1.24E-02	1.77E-01
rs4526799	Brain_Substantia_nigra	12	*RP11-603J24.7*	56374517	rs9739473	0.9012	57313335	A	T	0.37	2.65E-02	1.33E-02	2.49E-01
rs4526799	Brain_Substantia_nigra	12	*NXPH4*	57615405	rs9739473	0.9012	57313335	A	T	0.37	2.65E-02	2.73E-02	2.62E-01
rs4526799	Brain_Substantia_nigra	12	*CYP27B1*	58158578	rs9739473	0.9012	57313335	A	T	0.37	2.65E-02	1.24E-02	2.48E-01
rs4526799	Whole_Blood	12	*WIBG*	56310770	rs9919772	0.8450	57260027	T	C	0.33	1.03E-02	4.91E-02	2.35E-01
rs4526799	Whole_Blood	12	*GLS2*	56873481	rs9919772	0.8450	57260027	T	C	0.33	1.03E-02	4.22E-02	2.30E-01
rs4526799	Whole_Blood	12	*NAB2*	57485933	rs9919772	0.8450	57260027	T	C	0.33	1.03E-02	7.19E-03	1.93E-01
rs4526799	Whole_Blood	12	*KIF5A*	57962098	rs9919772	0.8450	57260027	T	C	0.33	1.03E-02	1.55E-02	2.05E-01
rs4526799	Whole_Blood	12	*AGAP2*	58127694	rs9919772	0.8450	57260027	T	C	0.33	1.03E-02	1.33E-02	2.02E-01
rs4526799	Whole_Blood	12	*WIBG*	56310770	rs4495925	0.8650	57268116	C	G	0.33	1.04E-02	4.67E-02	2.30E-01
rs4526799	Whole_Blood	12	*GLS2*	56873481	rs4495925	0.8650	57268116	C	G	0.33	1.04E-02	3.20E-02	2.18E-01
rs4526799	Whole_Blood	12	*NAB2*	57485933	rs4495925	0.8650	57268116	C	G	0.33	1.04E-02	1.24E-02	1.98E-01
rs4526799	Whole_Blood	12	*KIF5A*	57962098	rs4495925	0.8650	57268116	C	G	0.33	1.04E-02	2.01E-02	2.07E-01
rs4526799	Whole_Blood	12	*AGAP2*	58127694	rs4495925	0.8650	57268116	C	G	0.33	1.04E-02	9.49E-03	1.93E-01
rs4526799	Whole_Blood	12	*WIBG*	56310770	rs4471472	0.8650	57268985	A	G	0.32	9.99E-03	4.80E-02	2.28E-01
rs4526799	Whole_Blood	12	*GLS2*	56873481	rs4471472	0.8650	57268985	A	G	0.32	9.99E-03	3.14E-02	2.14E-01
rs4526799	Whole_Blood	12	*NAB2*	57485933	rs4471472	0.8650	57268985	A	G	0.32	9.99E-03	1.33E-02	1.95E-01
rs4526799	Whole_Blood	12	*KIF5A*	57962098	rs4471472	0.8650	57268985	A	G	0.32	9.99E-03	2.14E-02	2.04E-01
rs4526799	Whole_Blood	12	*AGAP2*	58127694	rs4471472	0.8650	57268985	A	G	0.32	9.99E-03	1.03E-02	1.91E-01
rs4526799	Whole_Blood	12	*WIBG*	56310770	rs4633499	0.8610	57269264	A	T	0.32	1.43E-02	4.79E-02	2.61E-01
rs4526799	Whole_Blood	12	*GLS2*	56873481	rs4633499	0.8610	57269264	A	T	0.32	1.43E-02	2.51E-02	2.43E-01
rs4526799	Whole_Blood	12	*NAB2*	57485933	rs4633499	0.8610	57269264	A	T	0.32	1.43E-02	1.32E-02	2.31E-01
rs4526799	Whole_Blood	12	*KIF5A*	57962098	rs4633499	0.8610	57269264	A	T	0.32	1.43E-02	4.39E-02	2.58E-01
rs4526799	Whole_Blood	12	*AGAP2*	58127694	rs4633499	0.8610	57269264	A	T	0.32	1.43E-02	9.18E-03	2.26E-01
rs4526799	Whole_Blood	12	*RP11-977G19.11*	56701259	rs12300079	0.9956	57273194	T	C	0.36	3.73E-03	1.36E-02	1.44E-01
rs4526799	Whole_Blood	12	*STAT2*	56744645	rs12300079	0.9956	57273194	T	C	0.36	3.73E-03	3.35E-02	1.68E-01
rs4526799	Whole_Blood	12	*GLS2*	56873481	rs12300079	0.9956	57273194	T	C	0.36	3.73E-03	1.20E-02	1.42E-01
rs4526799	Whole_Blood	12	*NAB2*	57485933	rs12300079	0.9956	57273194	T	C	0.36	3.73E-03	7.42E-03	1.34E-01
rs4526799	Whole_Blood	12	*KIF5A*	57962098	rs12300079	0.9956	57273194	T	C	0.36	3.73E-03	5.04E-03	1.28E-01
rs4526799	Whole_Blood	12	*RP11-571M6.8*	58118261	rs12300079	0.9956	57273194	T	C	0.36	3.73E-03	4.69E-02	1.81E-01
rs4526799	Whole_Blood	12	*AGAP2*	58127694	rs12300079	0.9956	57273194	T	C	0.36	3.73E-03	1.50E-02	1.46E-01
rs4526799	Whole_Blood	12	*RP11-977G19.11*	56701259	rs12300191	0.9956	57273289	A	G	0.36	3.59E-03	1.28E-02	1.41E-01
rs4526799	Whole_Blood	12	*STAT2*	56744645	rs12300191	0.9956	57273289	A	G	0.36	3.59E-03	3.80E-02	1.70E-01
rs4526799	Whole_Blood	12	*GLS2*	56873481	rs12300191	0.9956	57273289	A	G	0.36	3.59E-03	1.11E-02	1.38E-01
rs4526799	Whole_Blood	12	*NAB2*	57485933	rs12300191	0.9956	57273289	A	G	0.36	3.59E-03	7.35E-03	1.31E-01
rs4526799	Whole_Blood	12	*KIF5A*	57962098	rs12300191	0.9956	57273289	A	G	0.36	3.59E-03	4.78E-03	1.25E-01
rs4526799	Whole_Blood	12	*AGAP2*	58127694	rs12300191	0.9956	57273289	A	G	0.36	3.59E-03	1.40E-02	1.43E-01
rs4526799	Whole_Blood	12	*RP11-977G19.11*	56701259	rs4514464	0.9956	57276375	C	T	0.36	2.92E-03	1.21E-02	1.36E-01
rs4526799	Whole_Blood	12	*STAT2*	56744645	rs4514464	0.9956	57276375	C	T	0.36	2.92E-03	3.25E-02	1.62E-01

rs4526799	Whole_Blood	12	*GLS2*	56873481	rs4514464	0.9956	57276375	C	T	0.36	2.92E-03	1.06E-02	1.34E-01
rs4526799	Whole_Blood	12	*NAB2*	57485933	rs4514464	0.9956	57276375	C	T	0.36	2.92E-03	7.08E-03	1.27E-01
rs4526799	Whole_Blood	12	*KIF5A*	57962098	rs4514464	0.9956	57276375	C	T	0.36	2.92E-03	5.09E-03	1.23E-01
rs4526799	Whole_Blood	12	*RP11-571M6.8*	58118261	rs4514464	0.9956	57276375	C	T	0.36	2.92E-03	4.56E-02	1.74E-01
rs4526799	Whole_Blood	12	*AGAP2*	58127694	rs4514464	0.9956	57276375	C	T	0.36	2.92E-03	1.48E-02	1.41E-01
rs4526799	Whole_Blood	12	*RP11-977G19.11*	56701259	rs4417325	0.9956	57277302	G	A	0.36	3.59E-03	1.21E-02	1.40E-01
rs4526799	Whole_Blood	12	*STAT2*	56744645	rs4417325	0.9956	57277302	G	A	0.36	3.59E-03	3.25E-02	1.65E-01
rs4526799	Whole_Blood	12	*GLS2*	56873481	rs4417325	0.9956	57277302	G	A	0.36	3.59E-03	1.06E-02	1.37E-01
rs4526799	Whole_Blood	12	*NAB2*	57485933	rs4417325	0.9956	57277302	G	A	0.36	3.59E-03	7.08E-03	1.31E-01
rs4526799	Whole_Blood	12	*KIF5A*	57962098	rs4417325	0.9956	57277302	G	A	0.36	3.59E-03	5.09E-03	1.26E-01
rs4526799	Whole_Blood	12	*RP11-571M6.8*	58118261	rs4417325	0.9956	57277302	G	A	0.36	3.59E-03	4.56E-02	1.78E-01
rs4526799	Whole_Blood	12	*AGAP2*	58127694	rs4417325	0.9956	57277302	G	A	0.36	3.59E-03	1.48E-02	1.44E-01
rs4526799	Whole_Blood	12	*RP11-977G19.11*	56701259	rs11172030	0.9956	57278076	A	C	0.36	3.63E-03	1.21E-02	1.40E-01
rs4526799	Whole_Blood	12	*STAT2*	56744645	rs11172030	0.9956	57278076	A	C	0.36	3.63E-03	3.25E-02	1.65E-01
rs4526799	Whole_Blood	12	*GLS2*	56873481	rs11172030	0.9956	57278076	A	C	0.36	3.63E-03	1.06E-02	1.38E-01
rs4526799	Whole_Blood	12	*NAB2*	57485933	rs11172030	0.9956	57278076	A	C	0.36	3.63E-03	7.08E-03	1.31E-01
rs4526799	Whole_Blood	12	*KIF5A*	57962098	rs11172030	0.9956	57278076	A	C	0.36	3.63E-03	5.09E-03	1.27E-01
rs4526799	Whole_Blood	12	*RP11-571M6.8*	58118261	rs11172030	0.9956	57278076	A	C	0.36	3.63E-03	4.56E-02	1.78E-01
rs4526799	Whole_Blood	12	*AGAP2*	58127694	rs11172030	0.9956	57278076	A	C	0.36	3.63E-03	1.48E-02	1.44E-01
rs4526799	Whole_Blood	12	*RP11-977G19.11*	56701259	rs10876944	0.9956	57279372	T	A	0.36	3.63E-03	1.21E-02	1.40E-01
rs4526799	Whole_Blood	12	*STAT2*	56744645	rs10876944	0.9956	57279372	T	A	0.36	3.63E-03	3.25E-02	1.65E-01
rs4526799	Whole_Blood	12	*GLS2*	56873481	rs10876944	0.9956	57279372	T	A	0.36	3.63E-03	1.06E-02	1.38E-01
rs4526799	Whole_Blood	12	*NAB2*	57485933	rs10876944	0.9956	57279372	T	A	0.36	3.63E-03	7.08E-03	1.31E-01
rs4526799	Whole_Blood	12	*KIF5A*	57962098	rs10876944	0.9956	57279372	T	A	0.36	3.63E-03	5.09E-03	1.27E-01
rs4526799	Whole_Blood	12	*RP11-571M6.8*	58118261	rs10876944	0.9956	57279372	T	A	0.36	3.63E-03	4.56E-02	1.78E-01
rs4526799	Whole_Blood	12	*AGAP2*	58127694	rs10876944	0.9956	57279372	T	A	0.36	3.63E-03	1.48E-02	1.44E-01
rs4526799	Whole_Blood	12	*RP11-977G19.11*	56701259	rs4326839	0.9956	57280374	G	C	0.36	3.58E-03	1.21E-02	1.40E-01
rs4526799	Whole_Blood	12	*STAT2*	56744645	rs4326839	0.9956	57280374	G	C	0.36	3.58E-03	3.25E-02	1.65E-01
rs4526799	Whole_Blood	12	*GLS2*	56873481	rs4326839	0.9956	57280374	G	C	0.36	3.58E-03	1.06E-02	1.37E-01
rs4526799	Whole_Blood	12	*NAB2*	57485933	rs4326839	0.9956	57280374	G	C	0.36	3.58E-03	7.08E-03	1.30E-01
rs4526799	Whole_Blood	12	*KIF5A*	57962098	rs4326839	0.9956	57280374	G	C	0.36	3.58E-03	5.09E-03	1.26E-01
rs4526799	Whole_Blood	12	*RP11-571M6.8*	58118261	rs4326839	0.9956	57280374	G	C	0.36	3.58E-03	4.56E-02	1.77E-01
rs4526799	Whole_Blood	12	*AGAP2*	58127694	rs4326839	0.9956	57280374	G	C	0.36	3.58E-03	1.48E-02	1.44E-01
rs4526799	Whole_Blood	12	*RP11-977G19.11*	56701259	rs4526799	1.0000	57280586	T	C	0.34	7.26E-06	1.21E-02	4.04E-02
rs4526799	Whole_Blood	12	*STAT2*	56744645	rs4526799	1.0000	57280586	T	C	0.34	7.26E-06	3.25E-02	6.69E-02
rs4526799	Whole_Blood	12	*GLS2*	56873481	rs4526799	1.0000	57280586	T	C	0.34	7.26E-06	1.06E-02	3.80E-02
rs4526799	Whole_Blood	12	*NAB2*	57485933	rs4526799	1.0000	57280586	T	C	0.34	7.26E-06	7.08E-03	3.19E-02
rs4526799	Whole_Blood	12	*KIF5A*	57962098	rs4526799	1.0000	57280586	T	C	0.34	7.26E-06	5.09E-03	2.79E-02
rs4526799	Whole_Blood	12	*RP11-571M6.8*	58118261	rs4526799	1.0000	57280586	T	C	0.34	7.26E-06	4.56E-02	8.15E-02
rs4526799	Whole_Blood	12	*AGAP2*	58127694	rs4526799	1.0000	57280586	T	C	0.34	7.26E-06	1.48E-02	4.45E-02

rs4526799	Whole_Blood	12	*RP11-977G19.11*	56701259	rs28876529	0.9956	57285301	T	A	0.36	2.25E-03	1.21E-02	1.24E-01
rs4526799	Whole_Blood	12	*STAT2*	56744645	rs28876529	0.9956	57285301	T	A	0.36	2.25E-03	3.25E-02	1.50E-01
rs4526799	Whole_Blood	12	*GLS2*	56873481	rs28876529	0.9956	57285301	T	A	0.36	2.25E-03	1.06E-02	1.21E-01
rs4526799	Whole_Blood	12	*NAB2*	57485933	rs28876529	0.9956	57285301	T	A	0.36	2.25E-03	7.08E-03	1.14E-01
rs4526799	Whole_Blood	12	*KIF5A*	57962098	rs28876529	0.9956	57285301	T	A	0.36	2.25E-03	5.09E-03	1.09E-01
rs4526799	Whole_Blood	12	*RP11-571M6.8*	58118261	rs28876529	0.9956	57285301	T	A	0.36	2.25E-03	4.56E-02	1.63E-01
rs4526799	Whole_Blood	12	*AGAP2*	58127694	rs28876529	0.9956	57285301	T	A	0.36	2.25E-03	1.48E-02	1.28E-01
rs4526799	Whole_Blood	12	*RP11-977G19.11*	56701259	rs11172037	0.9956	57285427	T	A	0.36	2.25E-03	1.21E-02	1.24E-01
rs4526799	Whole_Blood	12	*STAT2*	56744645	rs11172037	0.9956	57285427	T	A	0.36	2.25E-03	3.25E-02	1.50E-01
rs4526799	Whole_Blood	12	*GLS2*	56873481	rs11172037	0.9956	57285427	T	A	0.36	2.25E-03	1.06E-02	1.21E-01
rs4526799	Whole_Blood	12	*NAB2*	57485933	rs11172037	0.9956	57285427	T	A	0.36	2.25E-03	7.08E-03	1.14E-01
rs4526799	Whole_Blood	12	*KIF5A*	57962098	rs11172037	0.9956	57285427	T	A	0.36	2.25E-03	5.09E-03	1.09E-01
rs4526799	Whole_Blood	12	*RP11-571M6.8*	58118261	rs11172037	0.9956	57285427	T	A	0.36	2.25E-03	4.56E-02	1.63E-01
rs4526799	Whole_Blood	12	*AGAP2*	58127694	rs11172037	0.9956	57285427	T	A	0.36	2.25E-03	1.48E-02	1.28E-01
rs4526799	Whole_Blood	12	*RP11-977G19.11*	56701259	rs12321987	0.9956	57288449	G	A	0.36	4.47E-03	1.21E-02	1.62E-01
rs4526799	Whole_Blood	12	*STAT2*	56744645	rs12321987	0.9956	57288449	G	A	0.36	4.47E-03	3.25E-02	1.86E-01
rs4526799	Whole_Blood	12	*GLS2*	56873481	rs12321987	0.9956	57288449	G	A	0.36	4.47E-03	1.06E-02	1.60E-01
rs4526799	Whole_Blood	12	*NAB2*	57485933	rs12321987	0.9956	57288449	G	A	0.36	4.47E-03	7.08E-03	1.53E-01
rs4526799	Whole_Blood	12	*KIF5A*	57962098	rs12321987	0.9956	57288449	G	A	0.36	4.47E-03	5.09E-03	1.49E-01
rs4526799	Whole_Blood	12	*RP11-571M6.8*	58118261	rs12321987	0.9956	57288449	G	A	0.36	4.47E-03	4.56E-02	1.98E-01
rs4526799	Whole_Blood	12	*AGAP2*	58127694	rs12321987	0.9956	57288449	G	A	0.36	4.47E-03	1.48E-02	1.66E-01
rs4526799	Whole_Blood	12	*RP11-977G19.11*	56701259	rs11172043	0.9869	57293182	G	A	0.35	4.58E-03	3.06E-02	1.83E-01
rs4526799	Whole_Blood	12	*GLS2*	56873481	rs11172043	0.9869	57293182	G	A	0.35	4.58E-03	7.33E-03	1.52E-01
rs4526799	Whole_Blood	12	*NAB2*	57485933	rs11172043	0.9869	57293182	G	A	0.35	4.58E-03	2.01E-02	1.71E-01
rs4526799	Whole_Blood	12	*KIF5A*	57962098	rs11172043	0.9869	57293182	G	A	0.35	4.58E-03	2.13E-02	1.73E-01
rs4526799	Whole_Blood	12	*AGAP2*	58127694	rs11172043	0.9869	57293182	G	A	0.35	4.58E-03	2.65E-02	1.78E-01
rs4526799	Whole_Blood	12	*CDK4*	58147010	rs11172043	0.9869	57293182	G	A	0.35	4.58E-03	2.17E-02	1.73E-01
rs4526799	Whole_Blood	12	*RP11-977G19.11*	56701259	rs12426816	0.9869	57294074	A	C	0.35	4.50E-03	2.97E-02	1.81E-01
rs4526799	Whole_Blood	12	*GLS2*	56873481	rs12426816	0.9869	57294074	A	C	0.35	4.50E-03	7.30E-03	1.52E-01
rs4526799	Whole_Blood	12	*NAB2*	57485933	rs12426816	0.9869	57294074	A	C	0.35	4.50E-03	1.96E-02	1.70E-01
rs4526799	Whole_Blood	12	*KIF5A*	57962098	rs12426816	0.9869	57294074	A	C	0.35	4.50E-03	2.07E-02	1.72E-01
rs4526799	Whole_Blood	12	*AGAP2*	58127694	rs12426816	0.9869	57294074	A	C	0.35	4.50E-03	2.73E-02	1.79E-01
rs4526799	Whole_Blood	12	*CDK4*	58147010	rs12426816	0.9869	57294074	A	C	0.35	4.50E-03	2.12E-02	1.72E-01
rs4526799	Whole_Blood	12	*RP11-977G19.11*	56701259	rs11172047	0.9869	57298080	T	C	0.35	6.51E-03	3.06E-02	1.96E-01
rs4526799	Whole_Blood	12	*GLS2*	56873481	rs11172047	0.9869	57298080	T	C	0.35	6.51E-03	7.33E-03	1.67E-01
rs4526799	Whole_Blood	12	*NAB2*	57485933	rs11172047	0.9869	57298080	T	C	0.35	6.51E-03	2.01E-02	1.85E-01
rs4526799	Whole_Blood	12	*KIF5A*	57962098	rs11172047	0.9869	57298080	T	C	0.35	6.51E-03	2.13E-02	1.87E-01
rs4526799	Whole_Blood	12	*AGAP2*	58127694	rs11172047	0.9869	57298080	T	C	0.35	6.51E-03	2.65E-02	1.92E-01
rs4526799	Whole_Blood	12	*CDK4*	58147010	rs11172047	0.9869	57298080	T	C	0.35	6.51E-03	2.17E-02	1.87E-01
rs4526799	Whole_Blood	12	*RP11-977G19.11*	56701259	rs2371631	0.9869	57298614	T	A	0.35	6.32E-03	3.06E-02	1.93E-01

rs4526799	Whole_Blood	12	*GLS2*	56873481	rs2371631	0.9869	57298614	T	A	0.35	6.32E-03	7.33E-03	1.64E-01
rs4526799	Whole_Blood	12	*NAB2*	57485933	rs2371631	0.9869	57298614	T	A	0.35	6.32E-03	2.01E-02	1.82E-01
rs4526799	Whole_Blood	12	*KIF5A*	57962098	rs2371631	0.9869	57298614	T	A	0.35	6.32E-03	2.13E-02	1.84E-01
rs4526799	Whole_Blood	12	*AGAP2*	58127694	rs2371631	0.9869	57298614	T	A	0.35	6.32E-03	2.65E-02	1.89E-01
rs4526799	Whole_Blood	12	*CDK4*	58147010	rs2371631	0.9869	57298614	T	A	0.35	6.32E-03	2.17E-02	1.84E-01
rs4526799	Whole_Blood	12	*RP11-977G19.11*	56701259	rs12305763	0.9869	57299263	G	A	0.35	6.82E-03	3.29E-02	2.00E-01
rs4526799	Whole_Blood	12	*GLS2*	56873481	rs12305763	0.9869	57299263	G	A	0.35	6.82E-03	8.73E-03	1.71E-01
rs4526799	Whole_Blood	12	*NAB2*	57485933	rs12305763	0.9869	57299263	G	A	0.35	6.82E-03	6.45E-03	1.67E-01
rs4526799	Whole_Blood	12	*KIF5A*	57962098	rs12305763	0.9869	57299263	G	A	0.35	6.82E-03	1.96E-02	1.86E-01
rs4526799	Whole_Blood	12	*AGAP2*	58127694	rs12305763	0.9869	57299263	G	A	0.35	6.82E-03	2.80E-02	1.95E-01
rs4526799	Whole_Blood	12	*CDK4*	58147010	rs12305763	0.9869	57299263	G	A	0.35	6.82E-03	2.92E-02	1.96E-01
rs4526799	Whole_Blood	12	*RP11-977G19.11*	56701259	rs11172049	0.9128	57304203	T	C	0.35	1.15E-02	1.95E-02	1.86E-01
rs4526799	Whole_Blood	12	*GLS2*	56873481	rs11172049	0.9128	57304203	T	C	0.35	1.15E-02	1.24E-02	1.77E-01
rs4526799	Whole_Blood	12	*NAB2*	57485933	rs11172049	0.9128	57304203	T	C	0.35	1.15E-02	1.99E-02	1.87E-01
rs4526799	Whole_Blood	12	*AGAP2*	58127694	rs11172049	0.9128	57304203	T	C	0.35	1.15E-02	2.88E-02	1.96E-01
rs4526799	Whole_Blood	12	*RP11-977G19.11*	56701259	rs1874888	0.9085	57305138	A	C	0.35	1.15E-02	1.77E-02	1.84E-01
rs4526799	Whole_Blood	12	*GLS2*	56873481	rs1874888	0.9085	57305138	A	C	0.35	1.15E-02	1.07E-02	1.75E-01
rs4526799	Whole_Blood	12	*NAB2*	57485933	rs1874888	0.9085	57305138	A	C	0.35	1.15E-02	2.14E-02	1.88E-01
rs4526799	Whole_Blood	12	*KIF5A*	57962098	rs1874888	0.9085	57305138	A	C	0.35	1.15E-02	4.96E-02	2.14E-01
rs4526799	Whole_Blood	12	*AGAP2*	58127694	rs1874888	0.9085	57305138	A	C	0.35	1.15E-02	2.86E-02	1.96E-01
rs4526799	Whole_Blood	12	*CDK4*	58147010	rs1874888	0.9085	57305138	A	C	0.35	1.15E-02	4.55E-02	2.11E-01
rs4526799	Whole_Blood	12	*RAB5B*	56378093	rs10506349	0.9128	57306412	T	C	0.37	1.10E-02	3.75E-02	1.95E-01
rs4526799	Whole_Blood	12	*ESYT1*	56530147	rs10506349	0.9128	57306412	T	C	0.37	1.10E-02	4.96E-02	2.06E-01
rs4526799	Whole_Blood	12	*RP11-977G19.11*	56701259	rs10506349	0.9128	57306412	T	C	0.37	1.10E-02	1.98E-02	1.77E-01
rs4526799	Whole_Blood	12	*GLS2*	56873481	rs10506349	0.9128	57306412	T	C	0.37	1.10E-02	4.63E-03	1.53E-01
rs4526799	Whole_Blood	12	*NAB2*	57485933	rs10506349	0.9128	57306412	T	C	0.37	1.10E-02	8.39E-03	1.61E-01
rs4526799	Whole_Blood	12	*KIF5A*	57962098	rs10506349	0.9128	57306412	T	C	0.37	1.10E-02	1.30E-02	1.68E-01
rs4526799	Whole_Blood	12	*RP11-571M6.8*	58118261	rs10506349	0.9128	57306412	T	C	0.37	1.10E-02	1.68E-02	1.73E-01
rs4526799	Whole_Blood	12	*AGAP2*	58127694	rs10506349	0.9128	57306412	T	C	0.37	1.10E-02	8.81E-03	1.62E-01
rs4526799	Whole_Blood	12	*RP11-977G19.11*	56701259	rs10876951	0.9085	57306430	T	G	0.35	1.15E-02	1.77E-02	1.84E-01
rs4526799	Whole_Blood	12	*GLS2*	56873481	rs10876951	0.9085	57306430	T	G	0.35	1.15E-02	1.07E-02	1.75E-01
rs4526799	Whole_Blood	12	*NAB2*	57485933	rs10876951	0.9085	57306430	T	G	0.35	1.15E-02	2.14E-02	1.88E-01
rs4526799	Whole_Blood	12	*KIF5A*	57962098	rs10876951	0.9085	57306430	T	G	0.35	1.15E-02	4.96E-02	2.14E-01
rs4526799	Whole_Blood	12	*AGAP2*	58127694	rs10876951	0.9085	57306430	T	G	0.35	1.15E-02	2.86E-02	1.96E-01
rs4526799	Whole_Blood	12	*CDK4*	58147010	rs10876951	0.9085	57306430	T	G	0.35	1.15E-02	4.55E-02	2.11E-01
rs4526799	Whole_Blood	12	*RAB5B*	56378093	rs10747774	0.9128	57307079	T	C	0.36	1.16E-02	3.62E-02	1.99E-01
rs4526799	Whole_Blood	12	*RP11-977G19.11*	56701259	rs10747774	0.9128	57307079	T	C	0.36	1.16E-02	2.02E-02	1.83E-01
rs4526799	Whole_Blood	12	*GLS2*	56873481	rs10747774	0.9128	57307079	T	C	0.36	1.16E-02	4.50E-03	1.58E-01
rs4526799	Whole_Blood	12	*NAB2*	57485933	rs10747774	0.9128	57307079	T	C	0.36	1.16E-02	8.04E-03	1.66E-01
rs4526799	Whole_Blood	12	*KIF5A*	57962098	rs10747774	0.9128	57307079	T	C	0.36	1.16E-02	1.29E-02	1.74E-01

rs4526799	Whole_Blood	12	*RP11-571M6.8*	58118261	rs10747774	0.9128	57307079	T	C	0.36	1.16E-02	1.64E-02	1.78E-01
rs4526799	Whole_Blood	12	*AGAP2*	58127694	rs10747774	0.9128	57307079	T	C	0.36	1.16E-02	7.78E-03	1.65E-01
rs4526799	Whole_Blood	12	*RP11-977G19.11*	56701259	rs10783812	0.9085	57308723	C	T	0.35	1.19E-02	1.90E-02	1.89E-01
rs4526799	Whole_Blood	12	*GLS2*	56873481	rs10783812	0.9085	57308723	C	T	0.35	1.19E-02	1.52E-02	1.84E-01
rs4526799	Whole_Blood	12	*NAB2*	57485933	rs10783812	0.9085	57308723	C	T	0.35	1.19E-02	1.50E-02	1.84E-01
rs4526799	Whole_Blood	12	*AGAP2*	58127694	rs10783812	0.9085	57308723	C	T	0.35	1.19E-02	3.02E-02	2.00E-01
rs4526799	Whole_Blood	12	*CDK4*	58147010	rs10783812	0.9085	57308723	C	T	0.35	1.19E-02	3.39E-02	2.04E-01
rs4526799	Whole_Blood	12	*RAB5B*	56378093	rs11172056	0.9089	57308975	C	T	0.37	1.10E-02	3.58E-02	1.94E-01
rs4526799	Whole_Blood	12	*RP11-977G19.11*	56701259	rs11172056	0.9089	57308975	C	T	0.37	1.10E-02	2.02E-02	1.78E-01
rs4526799	Whole_Blood	12	*GLS2*	56873481	rs11172056	0.9089	57308975	C	T	0.37	1.10E-02	4.30E-03	1.52E-01
rs4526799	Whole_Blood	12	*NAB2*	57485933	rs11172056	0.9089	57308975	C	T	0.37	1.10E-02	8.65E-03	1.62E-01
rs4526799	Whole_Blood	12	*KIF5A*	57962098	rs11172056	0.9089	57308975	C	T	0.37	1.10E-02	1.29E-02	1.68E-01
rs4526799	Whole_Blood	12	*RP11-571M6.8*	58118261	rs11172056	0.9089	57308975	C	T	0.37	1.10E-02	1.64E-02	1.73E-01
rs4526799	Whole_Blood	12	*AGAP2*	58127694	rs11172056	0.9089	57308975	C	T	0.37	1.10E-02	8.73E-03	1.62E-01
rs4526799	Whole_Blood	12	*RP11-977G19.11*	56701259	rs7302420	0.9085	57309884	G	C	0.36	1.54E-02	9.42E-03	1.93E-01
rs4526799	Whole_Blood	12	*GLS2*	56873481	rs7302420	0.9085	57309884	G	C	0.36	1.54E-02	6.46E-03	1.87E-01
rs4526799	Whole_Blood	12	*NAB2*	57485933	rs7302420	0.9085	57309884	G	C	0.36	1.54E-02	7.98E-03	1.90E-01
rs4526799	Whole_Blood	12	*KIF5A*	57962098	rs7302420	0.9085	57309884	G	C	0.36	1.54E-02	3.17E-02	2.17E-01
rs4526799	Whole_Blood	12	*RP11-571M6.8*	58118261	rs7302420	0.9085	57309884	G	C	0.36	1.54E-02	3.85E-02	2.23E-01
rs4526799	Whole_Blood	12	*AGAP2*	58127694	rs7302420	0.9085	57309884	G	C	0.36	1.54E-02	8.78E-03	1.92E-01
rs4526799	Whole_Blood	12	*RAB5B*	56378093	rs12228618	0.9128	57311229	T	C	0.37	1.29E-02	3.72E-02	2.04E-01
rs4526799	Whole_Blood	12	*RP11-977G19.11*	56701259	rs12228618	0.9128	57311229	T	C	0.37	1.29E-02	1.82E-02	1.84E-01
rs4526799	Whole_Blood	12	*GLS2*	56873481	rs12228618	0.9128	57311229	T	C	0.37	1.29E-02	4.23E-03	1.62E-01
rs4526799	Whole_Blood	12	*NAB2*	57485933	rs12228618	0.9128	57311229	T	C	0.37	1.29E-02	7.58E-03	1.69E-01
rs4526799	Whole_Blood	12	*KIF5A*	57962098	rs12228618	0.9128	57311229	T	C	0.37	1.29E-02	1.39E-02	1.79E-01
rs4526799	Whole_Blood	12	*RP11-571M6.8*	58118261	rs12228618	0.9128	57311229	T	C	0.37	1.29E-02	1.38E-02	1.79E-01
rs4526799	Whole_Blood	12	*AGAP2*	58127694	rs12228618	0.9128	57311229	T	C	0.37	1.29E-02	9.11E-03	1.72E-01
rs4526799	Whole_Blood	12	*RAB5B*	56378093	rs9739473	0.9012	57313335	A	T	0.37	2.65E-02	4.71E-02	2.76E-01
rs4526799	Whole_Blood	12	*RP11-977G19.11*	56701259	rs9739473	0.9012	57313335	A	T	0.37	2.65E-02	1.48E-02	2.50E-01
rs4526799	Whole_Blood	12	*GLS2*	56873481	rs9739473	0.9012	57313335	A	T	0.37	2.65E-02	3.59E-03	2.34E-01
rs4526799	Whole_Blood	12	*NAB2*	57485933	rs9739473	0.9012	57313335	A	T	0.37	2.65E-02	5.66E-03	2.38E-01
rs4526799	Whole_Blood	12	*KIF5A*	57962098	rs9739473	0.9012	57313335	A	T	0.37	2.65E-02	2.36E-02	2.59E-01
rs4526799	Whole_Blood	12	*RP11-571M6.8*	58118261	rs9739473	0.9012	57313335	A	T	0.37	2.65E-02	1.36E-02	2.49E-01
rs4526799	Whole_Blood	12	*AGAP2*	58127694	rs9739473	0.9012	57313335	A	T	0.37	2.65E-02	5.83E-03	2.39E-01
rs17105538	Brain_Amygdala	1	*MED28P8*	82023161	rs1338149	0.8903	81303974	C	T	0.13	4.17E-05	3.49E-02	6.26E-02
rs17105538	Brain_Amygdala	1	*MED28P8*	82023161	rs28520679	0.9907	81307167	T	C	0.14	2.55E-05	2.23E-02	4.44E-02
rs17105538	Brain_Amygdala	1	*MED28P8*	82023161	rs12066728	0.9907	81307791	C	T	0.14	1.18E-05	2.23E-02	4.22E-02
rs17105538	Brain_Amygdala	1	*MED28P8*	82023161	rs74897995	0.9907	81308276	T	C	0.14	1.18E-05	1.30E-02	3.02E-02
rs17105538	Brain_Amygdala	1	*MED28P8*	82023161	rs11585618	1.0000	81313870	G	C	0.15	1.30E-05	2.23E-02	4.24E-02
rs17105538	Brain_Amygdala	1	*MED28P8*	82023161	rs17105538	1.0000	81315043	G	A	0.15	7.66E-06	2.23E-02	4.19E-02

rs17105538	Brain_Amygdala	1	*MED28P8*	82023161	rs12075367	0.9908	81316333	A	G	0.15	1.30E-05	2.23E-02	4.24E-02
rs17105538	Brain_Amygdala	1	*MED28P8*	82023161	rs7549268	1.0000	81321890	G	T	0.14	1.40E-05	2.44E-02	4.46E-02
rs17105538	Brain_Amygdala	1	*MED28P8*	82023161	rs76492418	0.9908	81323652	G	A	0.15	1.73E-05	2.23E-02	4.27E-02
rs17105538	Brain_Amygdala	1	*MED28P8*	82023161	rs75125610	0.9544	81336210	G	T	0.15	3.84E-05	3.65E-02	6.02E-02
rs17105538	Brain_Amygdala	1	*MED28P8*	82023161	rs77640848	0.9544	81336212	C	T	0.15	3.84E-05	3.65E-02	6.02E-02
rs17105538	Brain_Amygdala	1	*MED28P8*	82023161	rs17105542	0.9541	81336283	T	G	0.15	3.56E-05	3.92E-02	6.29E-02
rs17105538	Brain_Amygdala	1	*MED28P8*	82023161	rs11586177	0.9544	81336453	G	A	0.15	3.84E-05	4.90E-02	7.37E-02
rs17105538	Brain_Amygdala	1	*MED28P8*	82023161	rs11576605	0.9544	81336822	T	C	0.15	3.61E-05	4.87E-02	7.32E-02
rs17105538	Brain_Amygdala	1	*MED28P8*	82023161	rs11586583	0.9544	81336886	C	A	0.15	3.61E-05	2.72E-02	4.96E-02
rs17105538	Brain_Amygdala	1	*MED28P8*	82023161	rs17105555	0.9544	81337489	T	C	0.15	3.93E-05	4.13E-02	6.56E-02
rs17105538	Brain_Amygdala	1	*MED28P8*	82023161	rs17105556	0.9544	81337509	A	C	0.15	3.93E-05	3.57E-02	5.95E-02
rs17105538	Brain_Amygdala	1	*MED28P8*	82023161	rs78184327	0.9544	81337634	T	C	0.15	3.93E-05	3.65E-02	6.04E-02
rs17105538	Brain_Amygdala	1	*MED28P8*	82023161	rs17105560	0.9544	81337866	A	G	0.15	3.93E-05	3.65E-02	6.04E-02
rs17105538	Brain_Amygdala	1	*MED28P8*	82023161	rs17105564	0.9544	81338054	C	T	0.15	3.38E-05	3.65E-02	6.02E-02
rs17105538	Brain_Amygdala	1	*MED28P8*	82023161	rs17105567	0.9633	81338490	A	T	0.15	3.38E-05	3.65E-02	6.02E-02
rs17105538	Brain_Amygdala	1	*MED28P8*	82023161	rs17105569	0.9544	81338600	T	A	0.15	3.44E-05	3.65E-02	6.02E-02
rs17105538	Brain_Amygdala	1	*MED28P8*	82023161	rs79444150	0.9457	81339200	A	G	0.15	3.97E-05	3.65E-02	6.06E-02
rs17105538	Brain_Amygdala	1	*MED28P8*	82023161	rs17105572	0.9457	81339231	A	G	0.15	3.97E-05	3.65E-02	6.06E-02
rs17105538	Brain_Amygdala	1	*MED28P8*	82023161	rs75355701	0.9457	81339306	G	A	0.15	3.97E-05	3.65E-02	6.06E-02
rs17105538	Brain_Amygdala	1	*MED28P8*	82023161	rs12354210	0.9457	81339963	G	A	0.15	6.16E-05	3.44E-02	5.98E-02
rs17105538	Brain_Amygdala	1	*MED28P8*	82023161	rs11163145	0.9370	81340124	C	T	0.15	8.83E-05	3.65E-02	6.35E-02
rs17105538	Brain_Amygdala	1	*MED28P8*	82023161	rs17402942	0.8366	81340931	C	A	0.12	9.39E-03	4.77E-03	4.47E-02
rs17105538	Brain_Frontal_Cortex_BA9	1	*LPHN2*	82114982	rs75125610	0.9544	81336210	G	T	0.15	3.84E-05	2.85E-02	5.12E-02
rs17105538	Brain_Frontal_Cortex_BA9	1	*LPHN2*	82114982	rs77640848	0.9544	81336212	C	T	0.15	3.84E-05	2.85E-02	5.12E-02
rs17105538	Brain_Frontal_Cortex_BA9	1	*LPHN2*	82114982	rs11586177	0.9544	81336453	G	A	0.15	3.84E-05	2.85E-02	5.12E-02
rs17105538	Brain_Frontal_Cortex_BA9	1	*LPHN2*	82114982	rs11576605	0.9544	81336822	T	C	0.15	3.61E-05	2.90E-02	5.16E-02
rs17105538	Brain_Frontal_Cortex_BA9	1	*LPHN2*	82114982	rs11586583	0.9544	81336886	C	A	0.15	3.61E-05	3.17E-02	5.48E-02
rs17105538	Brain_Frontal_Cortex_BA9	1	*LPHN2*	82114982	rs17105555	0.9544	81337489	T	C	0.15	3.93E-05	4.66E-02	7.13E-02
rs17105538	Brain_Frontal_Cortex_BA9	1	*LPHN2*	82114982	rs17105556	0.9544	81337509	A	C	0.15	3.93E-05	4.35E-02	6.79E-02
rs17105538	Brain_Frontal_Cortex_BA9	1	*LPHN2*	82114982	rs78184327	0.9544	81337634	T	C	0.15	3.93E-05	3.59E-02	5.96E-02
rs17105538	Brain_Frontal_Cortex_BA9	1	*LPHN2*	82114982	rs17105560	0.9544	81337866	A	G	0.15	3.93E-05	2.87E-02	5.15E-02
rs17105538	Brain_Frontal_Cortex_BA9	1	*LPHN2*	82114982	rs17105564	0.9544	81338054	C	T	0.15	3.38E-05	3.27E-02	5.60E-02
rs17105538	Brain_Frontal_Cortex_BA9	1	*LPHN2*	82114982	rs17105567	0.9633	81338490	A	T	0.15	3.38E-05	2.85E-02	5.12E-02
rs17105538	Brain_Frontal_Cortex_BA9	1	*LPHN2*	82114982	rs17105569	0.9544	81338600	T	A	0.15	3.44E-05	2.85E-02	5.12E-02
rs17105538	Brain_Frontal_Cortex_BA9	1	*LPHN2*	82114982	rs79444150	0.9457	81339200	A	G	0.15	3.97E-05	2.85E-02	5.16E-02
rs17105538	Brain_Frontal_Cortex_BA9	1	*LPHN2*	82114982	rs17105572	0.9457	81339231	A	G	0.15	3.97E-05	3.69E-02	6.10E-02
rs17105538	Brain_Frontal_Cortex_BA9	1	*LPHN2*	82114982	rs74457604	0.9457	81339287	T	C	0.15	7.61E-05	2.86E-02	5.41E-02
rs17105538	Brain_Frontal_Cortex_BA9	1	*LPHN2*	82114982	rs75355701	0.9457	81339306	G	A	0.15	3.97E-05	2.92E-02	5.24E-02
rs17105538	Brain_Frontal_Cortex_BA9	1	*LPHN2*	82114982	rs12354210	0.9457	81339963	G	A	0.15	6.16E-05	3.53E-02	6.08E-02
rs17105538	Brain_Frontal_Cortex_BA9	1	*LPHN2*	82114982	rs11163145	0.9370	81340124	C	T	0.15	8.83E-05	2.92E-02	5.52E-02

rs17105538	Brain_Hippocampus	1	*LPHN2*	82114982	rs1338149	0.8903	81303974	C	T	0.13	4.17E-05	4.70E-02	7.57E-02
rs17105538	Brain_Hippocampus	1	*LPHN2*	82114982	rs28520679	0.9907	81307167	T	C	0.14	2.55E-05	1.48E-02	3.47E-02
rs17105538	Brain_Hippocampus	1	*LPHN2*	82114982	rs12066728	0.9907	81307791	C	T	0.14	1.18E-05	1.91E-02	3.83E-02
rs17105538	Brain_Hippocampus	1	*LPHN2*	82114982	rs74897995	0.9907	81308276	T	C	0.14	1.18E-05	2.44E-02	4.48E-02
rs17105538	Brain_Hippocampus	1	*LPHN2*	82114982	rs11585618	1.0000	81313870	G	C	0.15	1.30E-05	1.91E-02	3.84E-02
rs17105538	Brain_Hippocampus	1	*LPHN2*	82114982	rs17105538	1.0000	81315043	G	A	0.15	7.66E-06	1.48E-02	3.23E-02
rs17105538	Brain_Hippocampus	1	*LPHN2*	82114982	rs12075367	0.9908	81316333	A	G	0.15	1.30E-05	1.91E-02	3.84E-02
rs17105538	Brain_Hippocampus	1	*LPHN2*	82114982	rs7549268	1.0000	81321890	G	T	0.14	1.40E-05	1.23E-02	2.92E-02
rs17105538	Brain_Hippocampus	1	*LPHN2*	82114982	rs76492418	0.9908	81323652	G	A	0.15	1.73E-05	1.48E-02	3.31E-02
rs17105538	Brain_Hippocampus	1	*LPHN2*	82114982	rs75125610	0.9544	81336210	G	T	0.15	3.84E-05	1.27E-02	3.12E-02
rs17105538	Brain_Hippocampus	1	*LPHN2*	82114982	rs77640848	0.9544	81336212	C	T	0.15	3.84E-05	1.27E-02	3.12E-02
rs17105538	Brain_Hippocampus	1	*LPHN2*	82114982	rs17105542	0.9541	81336283	T	G	0.15	3.56E-05	1.33E-02	3.19E-02
rs17105538	Brain_Hippocampus	1	*LPHN2*	82114982	rs11586177	0.9544	81336453	G	A	0.15	3.84E-05	9.26E-03	2.61E-02
rs17105538	Brain_Hippocampus	1	*LPHN2*	82114982	rs11576605	0.9544	81336822	T	C	0.15	3.61E-05	7.32E-03	2.30E-02
rs17105538	Brain_Hippocampus	1	*LPHN2*	82114982	rs11586583	0.9544	81336886	C	A	0.15	3.61E-05	6.42E-03	2.14E-02
rs17105538	Brain_Hippocampus	1	*LPHN2*	82114982	rs17105555	0.9544	81337489	T	C	0.15	3.93E-05	1.77E-02	3.81E-02
rs17105538	Brain_Hippocampus	1	*LPHN2*	82114982	rs17105556	0.9544	81337509	A	C	0.15	3.93E-05	2.41E-02	4.61E-02
rs17105538	Brain_Hippocampus	1	*LPHN2*	82114982	rs78184327	0.9544	81337634	T	C	0.15	3.93E-05	1.90E-02	3.98E-02
rs17105538	Brain_Hippocampus	1	*LPHN2*	82114982	rs17105560	0.9544	81337866	A	G	0.15	3.93E-05	1.13E-02	2.94E-02
rs17105538	Brain_Hippocampus	1	*LPHN2*	82114982	rs17105564	0.9544	81338054	C	T	0.15	3.38E-05	1.27E-02	3.12E-02
rs17105538	Brain_Hippocampus	1	*LPHN2*	82114982	rs17105567	0.9633	81338490	A	T	0.15	3.38E-05	1.27E-02	3.11E-02
rs17105538	Brain_Hippocampus	1	*LPHN2*	82114982	rs17105569	0.9544	81338600	T	A	0.15	3.44E-05	1.27E-02	3.12E-02
rs17105538	Brain_Hippocampus	1	*LPHN2*	82114982	rs79444150	0.9457	81339200	A	G	0.15	3.97E-05	1.27E-02	3.16E-02
rs17105538	Brain_Hippocampus	1	*LPHN2*	82114982	rs74457604	0.9457	81339287	T	C	0.15	7.61E-05	1.27E-02	3.37E-02
rs17105538	Brain_Hippocampus	1	*LPHN2*	82114982	rs75355701	0.9457	81339306	G	A	0.15	3.97E-05	1.27E-02	3.16E-02
rs17105538	Brain_Hippocampus	1	*LPHN2*	82114982	rs12354210	0.9457	81339963	G	A	0.15	6.16E-05	2.18E-02	4.50E-02
rs17105538	Brain_Hippocampus	1	*LPHN2*	82114982	rs11163145	0.9370	81340124	C	T	0.15	8.83E-05	1.27E-02	3.41E-02
rs17105538	Brain_Hypothalamus	1	*MED28P8*	82023161	rs75125610	0.9544	81336210	G	T	0.15	3.84E-05	2.52E-02	4.73E-02
rs17105538	Brain_Hypothalamus	1	*MED28P8*	82023161	rs77640848	0.9544	81336212	C	T	0.15	3.84E-05	2.52E-02	4.73E-02
rs17105538	Brain_Hypothalamus	1	*MED28P8*	82023161	rs17105542	0.9541	81336283	T	G	0.15	3.56E-05	1.81E-02	3.83E-02
rs17105538	Brain_Hypothalamus	1	*MED28P8*	82023161	rs11586177	0.9544	81336453	G	A	0.15	3.84E-05	2.52E-02	4.73E-02
rs17105538	Brain_Hypothalamus	1	*MED28P8*	82023161	rs11576605	0.9544	81336822	T	C	0.15	3.61E-05	2.59E-02	4.80E-02
rs17105538	Brain_Hypothalamus	1	*MED28P8*	82023161	rs11586583	0.9544	81336886	C	A	0.15	3.61E-05	3.94E-02	6.32E-02
rs17105538	Brain_Hypothalamus	1	*MED28P8*	82023161	rs17105555	0.9544	81337489	T	C	0.15	3.93E-05	2.79E-02	5.06E-02
rs17105538	Brain_Hypothalamus	1	*MED28P8*	82023161	rs17105556	0.9544	81337509	A	C	0.15	3.93E-05	2.86E-02	5.14E-02
rs17105538	Brain_Hypothalamus	1	*MED28P8*	82023161	rs78184327	0.9544	81337634	T	C	0.15	3.93E-05	2.16E-02	4.30E-02
rs17105538	Brain_Hypothalamus	1	*MED28P8*	82023161	rs17105560	0.9544	81337866	A	G	0.15	3.93E-05	2.99E-02	5.30E-02
rs17105538	Brain_Hypothalamus	1	*MED28P8*	82023161	rs17105564	0.9544	81338054	C	T	0.15	3.38E-05	2.44E-02	4.62E-02
rs17105538	Brain_Hypothalamus	1	*MED28P8*	82023161	rs17105567	0.9633	81338490	A	T	0.15	3.38E-05	2.52E-02	4.73E-02
rs17105538	Brain_Hypothalamus	1	*MED28P8*	82023161	rs17105569	0.9544	81338600	T	A	0.15	3.44E-05	2.52E-02	4.73E-02

rs17105538	Brain_Hypothalamus	1	*MED28P8*	82023161	rs79444150	0.9457	81339200	A	G	0.15	3.97E-05	2.52E-02	4.77E-02
rs17105538	Brain_Hypothalamus	1	*MED28P8*	82023161	rs17105572	0.9457	81339231	A	G	0.15	3.97E-05	2.52E-02	4.77E-02
rs17105538	Brain_Hypothalamus	1	*MED28P8*	82023161	rs74457604	0.9457	81339287	T	C	0.15	7.61E-05	2.59E-02	5.09E-02
rs17105538	Brain_Hypothalamus	1	*MED28P8*	82023161	rs75355701	0.9457	81339306	G	A	0.15	3.97E-05	2.52E-02	4.77E-02
rs17105538	Brain_Hypothalamus	1	*MED28P8*	82023161	rs12354210	0.9457	81339963	G	A	0.15	6.16E-05	2.22E-02	4.56E-02
rs17105538	Brain_Hypothalamus	1	*MED28P8*	82023161	rs11163145	0.9370	81340124	C	T	0.15	8.83E-05	2.52E-02	5.05E-02
rs17105538	Brain_Hypothalamus	1	*MED28P8*	82023161	rs17402942	0.8366	81340931	C	A	0.12	9.39E-03	2.39E-02	7.64E-02
rs17105538	Brain_Substantia_nigra	1	*LPHN2*	82114982	rs1338149	0.8903	81303974	C	T	0.13	4.17E-05	3.35E-02	6.10E-02
rs17105538	Brain_Substantia_nigra	1	*LPHN2*	82114982	rs28520679	0.9907	81307167	T	C	0.14	2.55E-05	3.79E-02	6.24E-02
rs17105538	Brain_Substantia_nigra	1	*LPHN2*	82114982	rs12066728	0.9907	81307791	C	T	0.14	1.18E-05	3.79E-02	6.01E-02
rs17105538	Brain_Substantia_nigra	1	*LPHN2*	82114982	rs74897995	0.9907	81308276	T	C	0.14	1.18E-05	4.77E-02	7.06E-02
rs17105538	Brain_Substantia_nigra	1	*LPHN2*	82114982	rs11585618	1.0000	81313870	G	C	0.15	1.30E-05	3.79E-02	6.03E-02
rs17105538	Brain_Substantia_nigra	1	*LPHN2*	82114982	rs17105538	1.0000	81315043	G	A	0.15	7.66E-06	3.79E-02	5.98E-02
rs17105538	Brain_Substantia_nigra	1	*LPHN2*	82114982	rs12075367	0.9908	81316333	A	G	0.15	1.30E-05	3.79E-02	6.03E-02
rs17105538	Brain_Substantia_nigra	1	*LPHN2*	82114982	rs7549268	1.0000	81321890	G	T	0.14	1.40E-05	3.79E-02	6.00E-02
rs17105538	Brain_Substantia_nigra	1	*LPHN2*	82114982	rs76492418	0.9908	81323652	G	A	0.15	1.73E-05	3.79E-02	6.06E-02
rs17105538	Brain_Substantia_nigra	1	*LPHN2*	82114982	rs17105556	0.9544	81337509	A	C	0.15	3.93E-05	4.88E-02	7.36E-02
rs17105538	Whole_Blood	1	*RP5-837I24.1*	81968316	rs11586177	0.9544	81336453	G	A	0.15	3.84E-05	2.90E-02	5.18E-02
rs17105538	Whole_Blood	1	*RP5-837I24.1*	81968316	rs11576605	0.9544	81336822	T	C	0.15	3.61E-05	4.80E-02	7.25E-02
rs17105538	Whole_Blood	1	*RP5-837I24.1*	81968316	rs11586583	0.9544	81336886	C	A	0.15	3.61E-05	3.96E-02	6.35E-02
rs17105538	Whole_Blood	1	*RP5-837I24.1*	81968316	rs78184327	0.9544	81337634	T	C	0.15	3.93E-05	3.73E-02	6.12E-02
rs17105538	Whole_Blood	1	*RP5-837I24.1*	81968316	rs17105560	0.9544	81337866	A	G	0.15	3.93E-05	4.91E-02	7.39E-02
rs17105538	Whole_Blood	1	*RP5-837I24.1*	81968316	rs17105569	0.9544	81338600	T	A	0.15	3.44E-05	3.94E-02	6.34E-02
rs17105538	Whole_Blood	1	*RP5-837I24.1*	81968316	rs79444150	0.9457	81339200	A	G	0.15	3.97E-05	4.45E-02	6.93E-02
rs17105538	Whole_Blood	1	*RP5-837I24.1*	81968316	rs74457604	0.9457	81339287	T	C	0.15	7.61E-05	4.68E-02	7.42E-02
rs17105538	Whole_Blood	1	*RP5-837I24.1*	81968316	rs75355701	0.9457	81339306	G	A	0.15	3.97E-05	4.82E-02	7.33E-02
rs17105538	Whole_Blood	1	*RP5-837I24.1*	81968316	rs12354210	0.9457	81339963	G	A	0.15	6.16E-05	3.02E-02	5.50E-02
rs17105538	Whole_Blood	1	*RP5-837I24.1*	81968316	rs11163145	0.9370	81340124	C	T	0.15	8.83E-05	4.39E-02	7.16E-02
rs17105538	Whole_Blood	1	*RP5-837I24.1*	81968316	rs17402942	0.8366	81340931	C	A	0.12	9.39E-03	1.28E-02	6.05E-02
rs62121100	Brain_Amygdala	2	*RNASEH1*	3599232	rs62119802	0.9108	3065229	C	T	0.16	1.56E-05	3.31E-02	6.77E-02
rs62121100	Brain_Amygdala	2	*RNASEH1*	3599232	rs76133582	0.9189	3067000	C	T	0.16	1.56E-05	3.10E-02	6.53E-02
rs62121100	Brain_Amygdala	2	*RNASEH1*	3599232	rs62119849	0.9431	3070219	T	C	0.17	2.89E-05	1.65E-02	4.76E-02
rs62121100	Brain_Amygdala	2	*RNASEH1*	3599232	rs80199046	0.9431	3070618	C	T	0.17	2.52E-05	1.72E-02	4.85E-02
rs62121100	Brain_Amygdala	2	*RNASEH1*	3599232	rs62119850	0.9431	3070946	C	G	0.17	2.36E-05	1.65E-02	4.70E-02
rs62121100	Brain_Amygdala	2	*RNASEH1*	3599232	rs62119851	0.9431	3071496	A	G	0.17	2.36E-05	1.65E-02	4.70E-02
rs62121100	Brain_Amygdala	2	*RNASEH1*	3599232	rs62119853	0.9431	3075633	T	C	0.17	1.99E-05	1.65E-02	4.66E-02
rs62121100	Brain_Amygdala	2	*RNASEH1*	3599232	rs62119854	0.9431	3078552	C	T	0.17	2.13E-05	1.72E-02	4.80E-02
rs62121100	Brain_Amygdala	2	*RNASEH1*	3599232	rs74933641	0.9431	3079764	T	G	0.17	2.38E-05	1.72E-02	4.87E-02
rs62121100	Brain_Amygdala	2	*RNASEH1*	3599232	rs61654279	0.9431	3082049	T	C	0.17	2.53E-05	1.65E-02	4.82E-02
rs62121100	Brain_Amygdala	2	*RNASEH1*	3599232	rs62119857	0.9431	3082113	C	G	0.17	2.28E-05	1.65E-02	4.75E-02

rs62121100	Brain_Amygdala	2	*AC108488.4*	3581933	rs62119858	0.9269	3082691	A	G	0.17	4.35E-05	3.34E-02	7.29E-02
rs62121100	Brain_Amygdala	2	*RNASEH1*	3599232	rs62119858	0.9269	3082691	A	G	0.17	4.35E-05	2.49E-02	6.28E-02
rs62121100	Brain_Amygdala	2	*RNASEH1*	3599232	rs62119859	1.0000	3084061	A	G	0.18	9.91E-06	2.12E-02	4.91E-02
rs62121100	Brain_Amygdala	2	*RNASEH1*	3599232	rs75418685	1.0000	3084835	T	C	0.18	9.81E-06	2.12E-02	4.90E-02
rs62121100	Brain_Amygdala	2	*RNASEH1*	3599232	rs62121061	0.9919	3085094	C	T	0.18	1.75E-05	2.12E-02	5.20E-02
rs62121100	Brain_Amygdala	2	*RNASEH1*	3599232	rs62121062	0.9918	3087273	C	T	0.18	1.12E-05	1.47E-02	4.09E-02
rs62121100	Brain_Amygdala	2	*RNASEH1*	3599232	rs62121098	1.0000	3092676	G	C	0.18	1.12E-05	2.12E-02	4.96E-02
rs62121100	Brain_Amygdala	2	*RNASEH1*	3599232	rs62121099	1.0000	3092721	C	T	0.18	1.12E-05	2.12E-02	4.96E-02
rs62121100	Brain_Amygdala	2	*RNASEH1*	3599232	rs62121100	1.0000	3093952	G	T	0.18	8.44E-06	1.76E-02	4.34E-02
rs62121100	Brain_Anterior_cingulate_cortex_BA24	2	*RP11-1293J14.1*	3500929	rs62119802	0.9108	3065229	C	T	0.16	1.56E-05	4.87E-02	8.48E-02
rs62121100	Brain_Anterior_cingulate_cortex_BA24	2	*SNORA73*	3628241	rs62119802	0.9108	3065229	C	T	0.16	1.56E-05	1.83E-02	4.95E-02
rs62121100	Brain_Anterior_cingulate_cortex_BA24	2	*RP11-1293J14.1*	3500929	rs76133582	0.9189	3067000	C	T	0.16	1.56E-05	4.87E-02	8.48E-02
rs62121100	Brain_Anterior_cingulate_cortex_BA24	2	*SNORA73*	3628241	rs76133582	0.9189	3067000	C	T	0.16	1.56E-05	1.83E-02	4.95E-02
rs62121100	Brain_Anterior_cingulate_cortex_BA24	2	*RP11-1293J14.1*	3500929	rs62119849	0.9431	3070219	T	C	0.17	2.89E-05	4.66E-02	8.33E-02
rs62121100	Brain_Anterior_cingulate_cortex_BA24	2	*SNORA73*	3628241	rs62119849	0.9431	3070219	T	C	0.17	2.89E-05	3.15E-02	6.65E-02
rs62121100	Brain_Anterior_cingulate_cortex_BA24	2	*TRAPPC12*	3436155	rs80199046	0.9431	3070618	C	T	0.17	2.52E-05	4.75E-02	8.41E-02
rs62121100	Brain_Anterior_cingulate_cortex_BA24	2	*RP11-1293J14.1*	3500929	rs80199046	0.9431	3070618	C	T	0.17	2.52E-05	4.26E-02	7.89E-02
rs62121100	Brain_Anterior_cingulate_cortex_BA24	2	*SNORA73*	3628241	rs80199046	0.9431	3070618	C	T	0.17	2.52E-05	2.86E-02	6.29E-02
rs62121100	Brain_Anterior_cingulate_cortex_BA24	2	*RP11-1293J14.1*	3500929	rs62119850	0.9431	3070946	C	G	0.17	2.36E-05	4.66E-02	8.27E-02
rs62121100	Brain_Anterior_cingulate_cortex_BA24	2	*SNORA73*	3628241	rs62119850	0.9431	3070946	C	G	0.17	2.36E-05	3.15E-02	6.59E-02
rs62121100	Brain_Anterior_cingulate_cortex_BA24	2	*RP11-1293J14.1*	3500929	rs62119851	0.9431	3071496	A	G	0.17	2.36E-05	4.66E-02	8.27E-02
rs62121100	Brain_Anterior_cingulate_cortex_BA24	2	*SNORA73*	3628241	rs62119851	0.9431	3071496	A	G	0.17	2.36E-05	3.15E-02	6.59E-02
rs62121100	Brain_Anterior_cingulate_cortex_BA24	2	*RP11-1293J14.1*	3500929	rs62119853	0.9431	3075633	T	C	0.17	1.99E-05	4.66E-02	8.23E-02
rs62121100	Brain_Anterior_cingulate_cortex_BA24	2	*SNORA73*	3628241	rs62119853	0.9431	3075633	T	C	0.17	1.99E-05	3.15E-02	6.55E-02
rs62121100	Brain_Anterior_cingulate_cortex_BA24	2	*TRAPPC12*	3436155	rs62119854	0.9431	3078552	C	T	0.17	2.13E-05	4.75E-02	8.36E-02
rs62121100	Brain_Anterior_cingulate_cortex_BA24	2	*RP11-1293J14.1*	3500929	rs62119854	0.9431	3078552	C	T	0.17	2.13E-05	4.26E-02	7.84E-02
rs62121100	Brain_Anterior_cingulate_cortex_BA24	2	*SNORA73*	3628241	rs62119854	0.9431	3078552	C	T	0.17	2.13E-05	2.86E-02	6.25E-02
rs62121100	Brain_Anterior_cingulate_cortex_BA24	2	*TRAPPC12*	3436155	rs74933641	0.9431	3079764	T	G	0.17	2.38E-05	4.75E-02	8.43E-02
rs62121100	Brain_Anterior_cingulate_cortex_BA24	2	*RP11-1293J14.1*	3500929	rs74933641	0.9431	3079764	T	G	0.17	2.38E-05	4.26E-02	7.91E-02
rs62121100	Brain_Anterior_cingulate_cortex_BA24	2	*SNORA73*	3628241	rs74933641	0.9431	3079764	T	G	0.17	2.38E-05	2.86E-02	6.32E-02
rs62121100	Brain_Anterior_cingulate_cortex_BA24	2	*RP11-1293J14.1*	3500929	rs61654279	0.9431	3082049	T	C	0.17	2.53E-05	4.66E-02	8.40E-02
rs62121100	Brain_Anterior_cingulate_cortex_BA24	2	*SNORA73*	3628241	rs61654279	0.9431	3082049	T	C	0.17	2.53E-05	3.15E-02	6.72E-02
rs62121100	Brain_Anterior_cingulate_cortex_BA24	2	*RP11-1293J14.1*	3500929	rs62119857	0.9431	3082113	C	G	0.17	2.28E-05	4.66E-02	8.33E-02
rs62121100	Brain_Anterior_cingulate_cortex_BA24	2	*SNORA73*	3628241	rs62119857	0.9431	3082113	C	G	0.17	2.28E-05	3.15E-02	6.65E-02
rs62121100	Brain_Anterior_cingulate_cortex_BA24	2	*TRAPPC12*	3436155	rs62119859	1.0000	3084061	A	G	0.18	9.91E-06	4.89E-02	8.06E-02
rs62121100	Brain_Anterior_cingulate_cortex_BA24	2	*RP11-1293J14.1*	3500929	rs62119859	1.0000	3084061	A	G	0.18	9.91E-06	3.77E-02	6.85E-02
rs62121100	Brain_Caudate_basal_ganglia	2	*RP11-1293J14.1*	3500929	rs62119849	0.9431	3070219	T	C	0.17	2.89E-05	4.88E-02	8.56E-02
rs62121100	Brain_Caudate_basal_ganglia	2	*RP11-1293J14.1*	3500929	rs62119850	0.9431	3070946	C	G	0.17	2.36E-05	4.88E-02	8.50E-02
rs62121100	Brain_Caudate_basal_ganglia	2	*RP11-1293J14.1*	3500929	rs62119851	0.9431	3071496	A	G	0.17	2.36E-05	4.88E-02	8.50E-02
rs62121100	Brain_Caudate_basal_ganglia	2	*RP11-1293J14.1*	3500929	rs62119853	0.9431	3075633	T	C	0.17	1.99E-05	4.88E-02	8.45E-02

rs62121100	Brain_Caudate_basal_ganglia	2	*RP11-1293J14.1*	3500929	rs61654279	0.9431	3082049	T	C	0.17	2.53E-05	4.88E-02	8.62E-02
rs62121100	Brain_Caudate_basal_ganglia	2	*RP11-1293J14.1*	3500929	rs62119857	0.9431	3082113	C	G	0.17	2.28E-05	4.88E-02	8.55E-02
rs62121100	Brain_Cerebellar_Hemisphere	2	*ALLC*	3727979	rs62119802	0.9108	3065229	C	T	0.16	1.56E-05	7.43E-03	3.26E-02
rs62121100	Brain_Cerebellar_Hemisphere	2	*ALLC*	3727979	rs76133582	0.9189	3067000	C	T	0.16	1.56E-05	7.43E-03	3.26E-02
rs62121100	Brain_Cerebellar_Hemisphere	2	*ALLC*	3727979	rs62119849	0.9431	3070219	T	C	0.17	2.89E-05	2.13E-02	5.40E-02
rs62121100	Brain_Cerebellar_Hemisphere	2	*ALLC*	3727979	rs80199046	0.9431	3070618	C	T	0.17	2.52E-05	2.13E-02	5.39E-02
rs62121100	Brain_Cerebellar_Hemisphere	2	*ALLC*	3727979	rs62119850	0.9431	3070946	C	G	0.17	2.36E-05	2.13E-02	5.35E-02
rs62121100	Brain_Cerebellar_Hemisphere	2	*ALLC*	3727979	rs62119851	0.9431	3071496	A	G	0.17	2.36E-05	2.13E-02	5.35E-02
rs62121100	Brain_Cerebellar_Hemisphere	2	*ALLC*	3727979	rs62119853	0.9431	3075633	T	C	0.17	1.99E-05	2.13E-02	5.30E-02
rs62121100	Brain_Cerebellar_Hemisphere	2	*ALLC*	3727979	rs62119854	0.9431	3078552	C	T	0.17	2.13E-05	2.13E-02	5.34E-02
rs62121100	Brain_Cerebellar_Hemisphere	2	*ALLC*	3727979	rs74933641	0.9431	3079764	T	G	0.17	2.38E-05	2.13E-02	5.41E-02
rs62121100	Brain_Cerebellar_Hemisphere	2	*ALLC*	3727979	rs61654279	0.9431	3082049	T	C	0.17	2.53E-05	2.13E-02	5.47E-02
rs62121100	Brain_Cerebellar_Hemisphere	2	*ALLC*	3727979	rs62119857	0.9431	3082113	C	G	0.17	2.28E-05	2.13E-02	5.40E-02
rs62121100	Brain_Cerebellar_Hemisphere	2	*ALLC*	3727979	rs62119858	0.9269	3082691	A	G	0.17	4.35E-05	2.15E-02	5.84E-02
rs62121100	Brain_Cerebellar_Hemisphere	2	*RNASEH1*	3599232	rs62119859	1.0000	3084061	A	G	0.18	9.91E-06	4.57E-02	7.72E-02
rs62121100	Brain_Cerebellar_Hemisphere	2	*ALLC*	3727979	rs62119859	1.0000	3084061	A	G	0.18	9.91E-06	3.58E-02	6.64E-02
rs62121100	Brain_Cerebellar_Hemisphere	2	*RNASEH1*	3599232	rs75418685	1.0000	3084835	T	C	0.18	9.81E-06	4.98E-02	8.16E-02
rs62121100	Brain_Cerebellar_Hemisphere	2	*ALLC*	3727979	rs75418685	1.0000	3084835	T	C	0.18	9.81E-06	4.90E-02	8.07E-02
rs62121100	Brain_Cerebellar_Hemisphere	2	*RNASEH1*	3599232	rs62121061	0.9919	3085094	C	T	0.18	1.75E-05	4.98E-02	8.47E-02
rs62121100	Brain_Cerebellar_Hemisphere	2	*ALLC*	3727979	rs62121061	0.9919	3085094	C	T	0.18	1.75E-05	4.90E-02	8.38E-02
rs62121100	Brain_Cerebellar_Hemisphere	2	*ALLC*	3727979	rs62121062	0.9918	3087273	C	T	0.18	1.12E-05	3.80E-02	6.94E-02
rs62121100	Brain_Cerebellar_Hemisphere	2	*RNASEH1*	3599232	rs62121098	1.0000	3092676	G	C	0.18	1.12E-05	4.98E-02	8.22E-02
rs62121100	Brain_Cerebellar_Hemisphere	2	*ALLC*	3727979	rs62121098	1.0000	3092676	G	C	0.18	1.12E-05	4.90E-02	8.13E-02
rs62121100	Brain_Cerebellar_Hemisphere	2	*RNASEH1*	3599232	rs62121099	1.0000	3092721	C	T	0.18	1.12E-05	4.98E-02	8.22E-02
rs62121100	Brain_Cerebellar_Hemisphere	2	*ALLC*	3727979	rs62121099	1.0000	3092721	C	T	0.18	1.12E-05	4.90E-02	8.13E-02
rs62121100	Brain_Cerebellar_Hemisphere	2	*RNASEH1*	3599232	rs62121100	1.0000	3093952	G	T	0.18	8.44E-06	4.98E-02	8.07E-02
rs62121100	Brain_Cerebellar_Hemisphere	2	*ALLC*	3727979	rs62121100	1.0000	3093952	G	T	0.18	8.44E-06	4.90E-02	7.98E-02
rs62121100	Brain_Cerebellum	2	*RP11-1293J14.1*	3500929	rs62119802	0.9108	3065229	C	T	0.16	1.56E-05	7.13E-03	3.20E-02
rs62121100	Brain_Cerebellum	2	*RP11-1293J14.1*	3500929	rs76133582	0.9189	3067000	C	T	0.16	1.56E-05	5.62E-03	2.90E-02
rs62121100	Brain_Cerebellum	2	*RP11-1293J14.1*	3500929	rs62119849	0.9431	3070219	T	C	0.17	2.89E-05	7.13E-03	3.26E-02
rs62121100	Brain_Cerebellum	2	*RP11-1293J14.1*	3500929	rs80199046	0.9431	3070618	C	T	0.17	2.52E-05	1.85E-02	5.02E-02
rs62121100	Brain_Cerebellum	2	*RP11-1293J14.1*	3500929	rs62119850	0.9431	3070946	C	G	0.17	2.36E-05	7.13E-03	3.21E-02
rs62121100	Brain_Cerebellum	2	*RP11-1293J14.1*	3500929	rs62119851	0.9431	3071496	A	G	0.17	2.36E-05	7.13E-03	3.21E-02
rs62121100	Brain_Cerebellum	2	*RP11-1293J14.1*	3500929	rs62119853	0.9431	3075633	T	C	0.17	1.99E-05	7.13E-03	3.17E-02
rs62121100	Brain_Cerebellum	2	*RP11-1293J14.1*	3500929	rs62119854	0.9431	3078552	C	T	0.17	2.13E-05	1.54E-02	4.54E-02
rs62121100	Brain_Cerebellum	2	*RP11-1293J14.1*	3500929	rs74933641	0.9431	3079764	T	G	0.17	2.38E-05	1.54E-02	4.61E-02
rs62121100	Brain_Cerebellum	2	*RP11-1293J14.1*	3500929	rs61654279	0.9431	3082049	T	C	0.17	2.53E-05	5.11E-03	2.90E-02
rs62121100	Brain_Cerebellum	2	*RP11-1293J14.1*	3500929	rs62119857	0.9431	3082113	C	G	0.17	2.28E-05	5.11E-03	2.84E-02
rs62121100	Brain_Cerebellum	2	*RP11-1293J14.1*	3500929	rs62119858	0.9269	3082691	A	G	0.17	4.35E-05	2.51E-02	6.29E-02
rs62121100	Brain_Cerebellum	2	*RP11-1293J14.1*	3500929	rs62119859	1.0000	3084061	A	G	0.18	9.91E-06	6.24E-03	2.66E-02

rs62121100	Brain_Cerebellum	2	*AC108488.4*	3581933	rs62119859	1.0000	3084061	A	G	0.18	9.91E-06	2.63E-02	5.54E-02
rs62121100	Brain_Cerebellum	2	*RP11-1293J14.1*	3500929	rs75418685	1.0000	3084835	T	C	0.18	9.81E-06	8.83E-03	3.13E-02
rs62121100	Brain_Cerebellum	2	*RP11-1293J14.1*	3500929	rs62121061	0.9919	3085094	C	T	0.18	1.75E-05	1.43E-02	4.25E-02
rs62121100	Brain_Cerebellum	2	*RP11-1293J14.1*	3500929	rs62121062	0.9918	3087273	C	T	0.18	1.12E-05	8.83E-03	3.18E-02
rs62121100	Brain_Cerebellum	2	*RP11-1293J14.1*	3500929	rs62121098	1.0000	3092676	G	C	0.18	1.12E-05	8.83E-03	3.18E-02
rs62121100	Brain_Cerebellum	2	*RP11-1293J14.1*	3500929	rs62121099	1.0000	3092721	C	T	0.18	1.12E-05	8.83E-03	3.18E-02
rs62121100	Brain_Cerebellum	2	*RP11-1293J14.1*	3500929	rs62121100	1.0000	3093952	G	T	0.18	8.44E-06	8.66E-03	3.03E-02
rs62121100	Brain_Cortex	2	*TSSC1*	3287174	rs62119802	0.9108	3065229	C	T	0.16	1.56E-05	4.91E-02	8.53E-02
rs62121100	Brain_Cortex	2	*RPS7*	3625652	rs62119802	0.9108	3065229	C	T	0.16	1.56E-05	5.49E-03	2.88E-02
rs62121100	Brain_Cortex	2	*TSSC1*	3287174	rs76133582	0.9189	3067000	C	T	0.16	1.56E-05	3.78E-02	7.31E-02
rs62121100	Brain_Cortex	2	*RPS7*	3625652	rs76133582	0.9189	3067000	C	T	0.16	1.56E-05	6.42E-03	3.07E-02
rs62121100	Brain_Cortex	2	*TSSC1*	3287174	rs62119849	0.9431	3070219	T	C	0.17	2.89E-05	4.91E-02	8.59E-02
rs62121100	Brain_Cortex	2	*RPS7*	3625652	rs62119849	0.9431	3070219	T	C	0.17	2.89E-05	5.49E-03	2.93E-02
rs62121100	Brain_Cortex	2	*TSSC1*	3287174	rs80199046	0.9431	3070618	C	T	0.17	2.52E-05	4.66E-02	8.32E-02
rs62121100	Brain_Cortex	2	*RPS7*	3625652	rs80199046	0.9431	3070618	C	T	0.17	2.52E-05	2.26E-03	2.10E-02
rs62121100	Brain_Cortex	2	*TSSC1*	3287174	rs62119850	0.9431	3070946	C	G	0.17	2.36E-05	4.91E-02	8.53E-02
rs62121100	Brain_Cortex	2	*RPS7*	3625652	rs62119850	0.9431	3070946	C	G	0.17	2.36E-05	5.49E-03	2.88E-02
rs62121100	Brain_Cortex	2	*TSSC1*	3287174	rs62119851	0.9431	3071496	A	G	0.17	2.36E-05	4.91E-02	8.53E-02
rs62121100	Brain_Cortex	2	*RPS7*	3625652	rs62119851	0.9431	3071496	A	G	0.17	2.36E-05	5.49E-03	2.88E-02
rs62121100	Brain_Cortex	2	*TSSC1*	3287174	rs62119853	0.9431	3075633	T	C	0.17	1.99E-05	4.91E-02	8.48E-02
rs62121100	Brain_Cortex	2	*RPS7*	3625652	rs62119853	0.9431	3075633	T	C	0.17	1.99E-05	5.49E-03	2.84E-02
rs62121100	Brain_Cortex	2	*TSSC1*	3287174	rs62119854	0.9431	3078552	C	T	0.17	2.13E-05	4.66E-02	8.27E-02
rs62121100	Brain_Cortex	2	*RPS7*	3625652	rs62119854	0.9431	3078552	C	T	0.17	2.13E-05	2.26E-03	2.07E-02
rs62121100	Brain_Cortex	2	*TSSC1*	3287174	rs74933641	0.9431	3079764	T	G	0.17	2.38E-05	4.66E-02	8.34E-02
rs62121100	Brain_Cortex	2	*RPS7*	3625652	rs74933641	0.9431	3079764	T	G	0.17	2.38E-05	2.26E-03	2.12E-02
rs62121100	Brain_Cortex	2	*RPS7*	3625652	rs61654279	0.9431	3082049	T	C	0.17	2.53E-05	6.24E-03	3.14E-02
rs62121100	Brain_Cortex	2	*RPS7*	3625652	rs62119857	0.9431	3082113	C	G	0.17	2.28E-05	6.24E-03	3.08E-02
rs62121100	Brain_Cortex	2	*RPS7*	3625652	rs62119858	0.9269	3082691	A	G	0.17	4.35E-05	1.04E-02	4.22E-02
rs62121100	Brain_Cortex	2	*RPS7*	3625652	rs62119859	1.0000	3084061	A	G	0.18	9.91E-06	4.29E-03	2.26E-02
rs62121100	Brain_Cortex	2	*TSSC1*	3287174	rs75418685	1.0000	3084835	T	C	0.18	9.81E-06	4.91E-02	8.08E-02
rs62121100	Brain_Cortex	2	*RPS7*	3625652	rs75418685	1.0000	3084835	T	C	0.18	9.81E-06	5.49E-03	2.51E-02
rs62121100	Brain_Cortex	2	*TSSC1*	3287174	rs62121061	0.9919	3085094	C	T	0.18	1.75E-05	4.52E-02	7.98E-02
rs62121100	Brain_Cortex	2	*RPS7*	3625652	rs62121061	0.9919	3085094	C	T	0.18	1.75E-05	2.39E-03	2.00E-02
rs62121100	Brain_Cortex	2	*RPS7*	3625652	rs62121062	0.9918	3087273	C	T	0.18	1.12E-05	8.32E-03	3.09E-02
rs62121100	Brain_Cortex	2	*TSSC1*	3287174	rs62121098	1.0000	3092676	G	C	0.18	1.12E-05	4.66E-02	7.87E-02
rs62121100	Brain_Cortex	2	*RPS7*	3625652	rs62121098	1.0000	3092676	G	C	0.18	1.12E-05	5.90E-03	2.65E-02
rs62121100	Brain_Cortex	2	*TSSC1*	3287174	rs62121099	1.0000	3092721	C	T	0.18	1.12E-05	4.91E-02	8.14E-02
rs62121100	Brain_Cortex	2	*RPS7*	3625652	rs62121099	1.0000	3092721	C	T	0.18	1.12E-05	5.49E-03	2.56E-02
rs62121100	Brain_Cortex	2	*TSSC1*	3287174	rs62121100	1.0000	3093952	G	T	0.18	8.44E-06	3.99E-02	7.00E-02
rs62121100	Brain_Cortex	2	*RPS7*	3625652	rs62121100	1.0000	3093952	G	T	0.18	8.44E-06	7.25E-03	2.78E-02

rs62121100	Brain_Frontal_Cortex_BA9	2	*TRAPPC12*	3436155	rs62119802	0.9108	3065229	C	T	0.16	1.56E-05	1.49E-02	4.47E-02
rs62121100	Brain_Frontal_Cortex_BA9	2	*TRAPPC12*	3436155	rs76133582	0.9189	3067000	C	T	0.16	1.56E-05	1.10E-02	3.88E-02
rs62121100	Brain_Frontal_Cortex_BA9	2	*TRAPPC12*	3436155	rs62119849	0.9431	3070219	T	C	0.17	2.89E-05	3.58E-02	7.15E-02
rs62121100	Brain_Frontal_Cortex_BA9	2	*TRAPPC12*	3436155	rs80199046	0.9431	3070618	C	T	0.17	2.52E-05	4.79E-02	8.45E-02
rs62121100	Brain_Frontal_Cortex_BA9	2	*TRAPPC12*	3436155	rs62119850	0.9431	3070946	C	G	0.17	2.36E-05	3.58E-02	7.09E-02
rs62121100	Brain_Frontal_Cortex_BA9	2	*TRAPPC12*	3436155	rs62119851	0.9431	3071496	A	G	0.17	2.36E-05	3.58E-02	7.09E-02
rs62121100	Brain_Frontal_Cortex_BA9	2	*TRAPPC12*	3436155	rs62119853	0.9431	3075633	T	C	0.17	1.99E-05	3.58E-02	7.04E-02
rs62121100	Brain_Frontal_Cortex_BA9	2	*TRAPPC12*	3436155	rs62119854	0.9431	3078552	C	T	0.17	2.13E-05	4.79E-02	8.40E-02
rs62121100	Brain_Frontal_Cortex_BA9	2	*TRAPPC12*	3436155	rs74933641	0.9431	3079764	T	G	0.17	2.38E-05	4.79E-02	8.48E-02
rs62121100	Brain_Frontal_Cortex_BA9	2	*TRAPPC12*	3436155	rs61654279	0.9431	3082049	T	C	0.17	2.53E-05	3.58E-02	7.21E-02
rs62121100	Brain_Frontal_Cortex_BA9	2	*TRAPPC12*	3436155	rs62119857	0.9431	3082113	C	G	0.17	2.28E-05	3.58E-02	7.14E-02
rs62121100	Brain_Frontal_Cortex_BA9	2	*TRAPPC12*	3436155	rs62119858	0.9269	3082691	A	G	0.17	4.35E-05	2.07E-02	5.74E-02
rs62121100	Brain_Frontal_Cortex_BA9	2	*TRAPPC12*	3436155	rs75418685	1.0000	3084835	T	C	0.18	9.81E-06	3.65E-02	6.72E-02
rs62121100	Brain_Frontal_Cortex_BA9	2	*TRAPPC12*	3436155	rs62121061	0.9919	3085094	C	T	0.18	1.75E-05	3.65E-02	7.03E-02
rs62121100	Brain_Frontal_Cortex_BA9	2	*TRAPPC12*	3436155	rs62121062	0.9918	3087273	C	T	0.18	1.12E-05	3.65E-02	6.78E-02
rs62121100	Brain_Frontal_Cortex_BA9	2	*TRAPPC12*	3436155	rs62121098	1.0000	3092676	G	C	0.18	1.12E-05	3.65E-02	6.78E-02
rs62121100	Brain_Frontal_Cortex_BA9	2	*TRAPPC12*	3436155	rs62121099	1.0000	3092721	C	T	0.18	1.12E-05	3.65E-02	6.78E-02
rs62121100	Brain_Frontal_Cortex_BA9	2	*TRAPPC12*	3436155	rs62121100	1.0000	3093952	G	T	0.18	8.44E-06	2.53E-02	5.32E-02
rs62121100	Brain_Hippocampus	2	*MYT1L*	2063958	rs62119802	0.9108	3065229	C	T	0.16	1.56E-05	4.49E-02	8.08E-02
rs62121100	Brain_Hippocampus	2	*RP11-1293J14.1*	3500929	rs62119802	0.9108	3065229	C	T	0.16	1.56E-05	1.42E-02	4.37E-02
rs62121100	Brain_Hippocampus	2	*RP11-1293J14.1*	3500929	rs76133582	0.9189	3067000	C	T	0.16	1.56E-05	2.06E-02	5.25E-02
rs62121100	Brain_Hippocampus	2	*MYT1L*	2063958	rs62119849	0.9431	3070219	T	C	0.17	2.89E-05	4.49E-02	8.15E-02
rs62121100	Brain_Hippocampus	2	*RP11-1293J14.1*	3500929	rs62119849	0.9431	3070219	T	C	0.17	2.89E-05	1.42E-02	4.43E-02
rs62121100	Brain_Hippocampus	2	*RP11-1293J14.1*	3500929	rs80199046	0.9431	3070618	C	T	0.17	2.52E-05	1.67E-02	4.77E-02
rs62121100	Brain_Hippocampus	2	*RNASEH1*	3599232	rs80199046	0.9431	3070618	C	T	0.17	2.52E-05	3.84E-02	7.42E-02
rs62121100	Brain_Hippocampus	2	*AC108488.3*	3607765	rs80199046	0.9431	3070618	C	T	0.17	2.52E-05	4.10E-02	7.71E-02
rs62121100	Brain_Hippocampus	2	*MYT1L*	2063958	rs62119850	0.9431	3070946	C	G	0.17	2.36E-05	4.49E-02	8.09E-02
rs62121100	Brain_Hippocampus	2	*RP11-1293J14.1*	3500929	rs62119850	0.9431	3070946	C	G	0.17	2.36E-05	1.42E-02	4.38E-02
rs62121100	Brain_Hippocampus	2	*MYT1L*	2063958	rs62119851	0.9431	3071496	A	G	0.17	2.36E-05	4.49E-02	8.09E-02
rs62121100	Brain_Hippocampus	2	*RP11-1293J14.1*	3500929	rs62119851	0.9431	3071496	A	G	0.17	2.36E-05	1.42E-02	4.38E-02
rs62121100	Brain_Hippocampus	2	*MYT1L*	2063958	rs62119853	0.9431	3075633	T	C	0.17	1.99E-05	4.49E-02	8.04E-02
rs62121100	Brain_Hippocampus	2	*RP11-1293J14.1*	3500929	rs62119853	0.9431	3075633	T	C	0.17	1.99E-05	1.42E-02	4.33E-02
rs62121100	Brain_Hippocampus	2	*RP11-1293J14.1*	3500929	rs62119854	0.9431	3078552	C	T	0.17	2.13E-05	1.67E-02	4.72E-02
rs62121100	Brain_Hippocampus	2	*RNASEH1*	3599232	rs62119854	0.9431	3078552	C	T	0.17	2.13E-05	3.84E-02	7.37E-02
rs62121100	Brain_Hippocampus	2	*AC108488.3*	3607765	rs62119854	0.9431	3078552	C	T	0.17	2.13E-05	4.10E-02	7.66E-02
rs62121100	Brain_Hippocampus	2	*RP11-1293J14.1*	3500929	rs74933641	0.9431	3079764	T	G	0.17	2.38E-05	1.67E-02	4.79E-02
rs62121100	Brain_Hippocampus	2	*RNASEH1*	3599232	rs74933641	0.9431	3079764	T	G	0.17	2.38E-05	3.84E-02	7.44E-02
rs62121100	Brain_Hippocampus	2	*AC108488.3*	3607765	rs74933641	0.9431	3079764	T	G	0.17	2.38E-05	4.10E-02	7.73E-02
rs62121100	Brain_Hippocampus	2	*MYT1L*	2063958	rs61654279	0.9431	3082049	T	C	0.17	2.53E-05	4.49E-02	8.21E-02
rs62121100	Brain_Hippocampus	2	*RP11-1293J14.1*	3500929	rs61654279	0.9431	3082049	T	C	0.17	2.53E-05	1.42E-02	4.49E-02

rs62121100	Brain_Hippocampus	2	*MYT1L*	2063958	rs62119857	0.9431	3082113	C	G	0.17	2.28E-05	4.49E-02	8.14E-02
rs62121100	Brain_Hippocampus	2	*RP11-1293J14.1*	3500929	rs62119857	0.9431	3082113	C	G	0.17	2.28E-05	1.42E-02	4.43E-02
rs62121100	Brain_Hippocampus	2	*MYT1L*	2063958	rs62119858	0.9269	3082691	A	G	0.17	4.35E-05	4.04E-02	8.07E-02
rs62121100	Brain_Hippocampus	2	*RP11-1293J14.1*	3500929	rs62119858	0.9269	3082691	A	G	0.17	4.35E-05	3.78E-02	7.78E-02
rs62121100	Brain_Hippocampus	2	*MYT1L*	2063958	rs62119859	1.0000	3084061	A	G	0.18	9.91E-06	2.32E-02	5.16E-02
rs62121100	Brain_Hippocampus	2	*RP11-1293J14.1*	3500929	rs62119859	1.0000	3084061	A	G	0.18	9.91E-06	1.71E-02	4.37E-02
rs62121100	Brain_Hippocampus	2	*MYT1L*	2063958	rs75418685	1.0000	3084835	T	C	0.18	9.81E-06	3.72E-02	6.79E-02
rs62121100	Brain_Hippocampus	2	*RP11-1293J14.1*	3500929	rs75418685	1.0000	3084835	T	C	0.18	9.81E-06	1.76E-02	4.43E-02
rs62121100	Brain_Hippocampus	2	*MYT1L*	2063958	rs62121061	0.9919	3085094	C	T	0.18	1.75E-05	3.72E-02	7.10E-02
rs62121100	Brain_Hippocampus	2	*RP11-1293J14.1*	3500929	rs62121061	0.9919	3085094	C	T	0.18	1.75E-05	1.76E-02	4.72E-02
rs62121100	Brain_Hippocampus	2	*MYT1L*	2063958	rs62121062	0.9918	3087273	C	T	0.18	1.12E-05	4.48E-02	7.68E-02
rs62121100	Brain_Hippocampus	2	*RP11-1293J14.1*	3500929	rs62121062	0.9918	3087273	C	T	0.18	1.12E-05	1.51E-02	4.14E-02
rs62121100	Brain_Hippocampus	2	*MYT1L*	2063958	rs62121098	1.0000	3092676	G	C	0.18	1.12E-05	3.72E-02	6.85E-02
rs62121100	Brain_Hippocampus	2	*RP11-1293J14.1*	3500929	rs62121098	1.0000	3092676	G	C	0.18	1.12E-05	1.76E-02	4.49E-02
rs62121100	Brain_Hippocampus	2	*MYT1L*	2063958	rs62121099	1.0000	3092721	C	T	0.18	1.12E-05	3.72E-02	6.85E-02
rs62121100	Brain_Hippocampus	2	*RP11-1293J14.1*	3500929	rs62121099	1.0000	3092721	C	T	0.18	1.12E-05	1.76E-02	4.49E-02
rs62121100	Brain_Hippocampus	2	*MYT1L*	2063958	rs62121100	1.0000	3093952	G	T	0.18	8.44E-06	3.86E-02	6.86E-02
rs62121100	Brain_Hippocampus	2	*RP11-1293J14.1*	3500929	rs62121100	1.0000	3093952	G	T	0.18	8.44E-06	1.11E-02	3.42E-02
rs62121100	Brain_Nucleus_accumbens_basal_ganglia	2	*TSSC1-IT1*	3303674	rs62119802	0.9108	3065229	C	T	0.16	1.56E-05	1.86E-02	4.98E-02
rs62121100	Brain_Nucleus_accumbens_basal_ganglia	2	*RP11-1293J14.1*	3500929	rs62119802	0.9108	3065229	C	T	0.16	1.56E-05	3.91E-02	7.45E-02
rs62121100	Brain_Nucleus_accumbens_basal_ganglia	2	*TSSC1-IT1*	3303674	rs76133582	0.9189	3067000	C	T	0.16	1.56E-05	1.44E-02	4.40E-02
rs62121100	Brain_Nucleus_accumbens_basal_ganglia	2	*RP11-1293J14.1*	3500929	rs76133582	0.9189	3067000	C	T	0.16	1.56E-05	4.55E-02	8.15E-02
rs62121100	Brain_Nucleus_accumbens_basal_ganglia	2	*AC142528.1*	3525007	rs76133582	0.9189	3067000	C	T	0.16	1.56E-05	4.75E-02	8.36E-02
rs62121100	Brain_Nucleus_accumbens_basal_ganglia	2	*TSSC1-IT1*	3303674	rs62119849	0.9431	3070219	T	C	0.17	2.89E-05	2.43E-02	5.79E-02
rs62121100	Brain_Nucleus_accumbens_basal_ganglia	2	*AC142528.1*	3525007	rs62119849	0.9431	3070219	T	C	0.17	2.89E-05	3.10E-02	6.59E-02
rs62121100	Brain_Nucleus_accumbens_basal_ganglia	2	*TSSC1-IT1*	3303674	rs80199046	0.9431	3070618	C	T	0.17	2.52E-05	4.52E-02	8.16E-02
rs62121100	Brain_Nucleus_accumbens_basal_ganglia	2	*RP11-1293J14.1*	3500929	rs80199046	0.9431	3070618	C	T	0.17	2.52E-05	4.11E-02	7.72E-02
rs62121100	Brain_Nucleus_accumbens_basal_ganglia	2	*AC142528.1*	3525007	rs80199046	0.9431	3070618	C	T	0.17	2.52E-05	3.32E-02	6.84E-02
rs62121100	Brain_Nucleus_accumbens_basal_ganglia	2	*TSSC1-IT1*	3303674	rs62119850	0.9431	3070946	C	G	0.17	2.36E-05	2.43E-02	5.73E-02
rs62121100	Brain_Nucleus_accumbens_basal_ganglia	2	*AC142528.1*	3525007	rs62119850	0.9431	3070946	C	G	0.17	2.36E-05	3.10E-02	6.53E-02
rs62121100	Brain_Nucleus_accumbens_basal_ganglia	2	*TSSC1-IT1*	3303674	rs62119851	0.9431	3071496	A	G	0.17	2.36E-05	2.43E-02	5.73E-02
rs62121100	Brain_Nucleus_accumbens_basal_ganglia	2	*AC142528.1*	3525007	rs62119851	0.9431	3071496	A	G	0.17	2.36E-05	3.10E-02	6.53E-02
rs62121100	Brain_Nucleus_accumbens_basal_ganglia	2	*TSSC1-IT1*	3303674	rs62119853	0.9431	3075633	T	C	0.17	1.99E-05	2.43E-02	5.69E-02
rs62121100	Brain_Nucleus_accumbens_basal_ganglia	2	*AC142528.1*	3525007	rs62119853	0.9431	3075633	T	C	0.17	1.99E-05	3.10E-02	6.49E-02
rs62121100	Brain_Nucleus_accumbens_basal_ganglia	2	*TSSC1-IT1*	3303674	rs62119854	0.9431	3078552	C	T	0.17	2.13E-05	4.52E-02	8.11E-02
rs62121100	Brain_Nucleus_accumbens_basal_ganglia	2	*RP11-1293J14.1*	3500929	rs62119854	0.9431	3078552	C	T	0.17	2.13E-05	4.11E-02	7.67E-02
rs62121100	Brain_Nucleus_accumbens_basal_ganglia	2	*AC142528.1*	3525007	rs62119854	0.9431	3078552	C	T	0.17	2.13E-05	3.32E-02	6.79E-02
rs62121100	Brain_Nucleus_accumbens_basal_ganglia	2	*TSSC1-IT1*	3303674	rs74933641	0.9431	3079764	T	G	0.17	2.38E-05	4.52E-02	8.18E-02
rs62121100	Brain_Nucleus_accumbens_basal_ganglia	2	*RP11-1293J14.1*	3500929	rs74933641	0.9431	3079764	T	G	0.17	2.38E-05	4.11E-02	7.74E-02
rs62121100	Brain_Nucleus_accumbens_basal_ganglia	2	*AC142528.1*	3525007	rs74933641	0.9431	3079764	T	G	0.17	2.38E-05	3.32E-02	6.86E-02

rs62121100	Brain_Nucleus_accumbens_basal_ganglia	2	*TSSC1-IT1*	3303674	rs61654279	0.9431	3082049	T	C	0.17	2.53E-05	2.43E-02	5.85E-02
rs62121100	Brain_Nucleus_accumbens_basal_ganglia	2	*AC142528.1*	3525007	rs61654279	0.9431	3082049	T	C	0.17	2.53E-05	3.10E-02	6.66E-02
rs62121100	Brain_Nucleus_accumbens_basal_ganglia	2	*TSSC1-IT1*	3303674	rs62119857	0.9431	3082113	C	G	0.17	2.28E-05	2.43E-02	5.78E-02
rs62121100	Brain_Nucleus_accumbens_basal_ganglia	2	*AC142528.1*	3525007	rs62119857	0.9431	3082113	C	G	0.17	2.28E-05	3.10E-02	6.59E-02
rs62121100	Brain_Nucleus_accumbens_basal_ganglia	2	*TSSC1-IT1*	3303674	rs62119858	0.9269	3082691	A	G	0.17	4.35E-05	3.60E-02	7.58E-02
rs62121100	Brain_Nucleus_accumbens_basal_ganglia	2	*TSSC1-IT1*	3303674	rs62119859	1.0000	3084061	A	G	0.18	9.91E-06	2.54E-02	5.42E-02
rs62121100	Brain_Nucleus_accumbens_basal_ganglia	2	*AC142528.1*	3525007	rs62119859	1.0000	3084061	A	G	0.18	9.91E-06	1.45E-02	4.00E-02
rs62121100	Brain_Nucleus_accumbens_basal_ganglia	2	*TSSC1-IT1*	3303674	rs75418685	1.0000	3084835	T	C	0.18	9.81E-06	2.43E-02	5.29E-02
rs62121100	Brain_Nucleus_accumbens_basal_ganglia	2	*AC142528.1*	3525007	rs75418685	1.0000	3084835	T	C	0.18	9.81E-06	3.10E-02	6.09E-02
rs62121100	Brain_Nucleus_accumbens_basal_ganglia	2	*TSSC1-IT1*	3303674	rs62121061	0.9919	3085094	C	T	0.18	1.75E-05	2.43E-02	5.59E-02
rs62121100	Brain_Nucleus_accumbens_basal_ganglia	2	*AC142528.1*	3525007	rs62121061	0.9919	3085094	C	T	0.18	1.75E-05	3.10E-02	6.39E-02
rs62121100	Brain_Nucleus_accumbens_basal_ganglia	2	*TSSC1-IT1*	3303674	rs62121062	0.9918	3087273	C	T	0.18	1.12E-05	3.08E-02	6.13E-02
rs62121100	Brain_Nucleus_accumbens_basal_ganglia	2	*AC142528.1*	3525007	rs62121062	0.9918	3087273	C	T	0.18	1.12E-05	2.41E-02	5.33E-02
rs62121100	Brain_Nucleus_accumbens_basal_ganglia	2	*TSSC1-IT1*	3303674	rs62121098	1.0000	3092676	G	C	0.18	1.12E-05	2.78E-02	5.77E-02
rs62121100	Brain_Nucleus_accumbens_basal_ganglia	2	*RP11-1293J14.1*	3500929	rs62121098	1.0000	3092676	G	C	0.18	1.12E-05	4.76E-02	7.98E-02
rs62121100	Brain_Nucleus_accumbens_basal_ganglia	2	*AC142528.1*	3525007	rs62121098	1.0000	3092676	G	C	0.18	1.12E-05	2.46E-02	5.38E-02
rs62121100	Brain_Nucleus_accumbens_basal_ganglia	2	*TSSC1-IT1*	3303674	rs62121099	1.0000	3092721	C	T	0.18	1.12E-05	2.52E-02	5.46E-02
rs62121100	Brain_Nucleus_accumbens_basal_ganglia	2	*AC142528.1*	3525007	rs62121099	1.0000	3092721	C	T	0.18	1.12E-05	3.75E-02	6.89E-02
rs62121100	Brain_Nucleus_accumbens_basal_ganglia	2	*TSSC1-IT1*	3303674	rs62121100	1.0000	3093952	G	T	0.18	8.44E-06	1.98E-02	4.64E-02
rs62121100	Brain_Nucleus_accumbens_basal_ganglia	2	*RP11-1293J14.1*	3500929	rs62121100	1.0000	3093952	G	T	0.18	8.44E-06	4.22E-02	7.26E-02
rs62121100	Brain_Nucleus_accumbens_basal_ganglia	2	*AC142528.1*	3525007	rs62121100	1.0000	3093952	G	T	0.18	8.44E-06	3.56E-02	6.53E-02
rs62121100	Brain_Spinal_cord_cervical_c-1	2	*ALLC*	3727979	rs62119802	0.9108	3065229	C	T	0.16	1.56E-05	3.41E-02	6.89E-02
rs62121100	Brain_Spinal_cord_cervical_c-1	2	*ALLC*	3727979	rs76133582	0.9189	3067000	C	T	0.16	1.56E-05	3.58E-02	7.08E-02
rs62121100	Brain_Spinal_cord_cervical_c-1	2	*ALLC*	3727979	rs80199046	0.9431	3070618	C	T	0.17	2.52E-05	4.54E-02	8.19E-02
rs62121100	Brain_Spinal_cord_cervical_c-1	2	*ALLC*	3727979	rs62119854	0.9431	3078552	C	T	0.17	2.13E-05	4.54E-02	8.14E-02
rs62121100	Brain_Spinal_cord_cervical_c-1	2	*ALLC*	3727979	rs74933641	0.9431	3079764	T	G	0.17	2.38E-05	4.54E-02	8.21E-02
rs62121100	Brain_Spinal_cord_cervical_c-1	2	*ALLC*	3727979	rs62119858	0.9269	3082691	A	G	0.17	4.35E-05	4.54E-02	8.62E-02
rs62121100	Brain_Spinal_cord_cervical_c-1	2	*AC142528.1*	3525007	rs62121100	1.0000	3093952	G	T	0.18	8.44E-06	3.75E-02	6.74E-02
rs62121100	Brain_Substantia_nigra	2	*TMSB4XP2*	3665242	rs62119802	0.9108	3065229	C	T	0.16	1.56E-05	2.47E-02	5.77E-02
rs62121100	Brain_Substantia_nigra	2	*TMSB4XP2*	3665242	rs76133582	0.9189	3067000	C	T	0.16	1.56E-05	3.84E-02	7.37E-02
rs62121100	Brain_Substantia_nigra	2	*TMSB4XP2*	3665242	rs62119849	0.9431	3070219	T	C	0.17	2.89E-05	2.47E-02	5.83E-02
rs62121100	Brain_Substantia_nigra	2	*AC108488.3*	3607765	rs80199046	0.9431	3070618	C	T	0.17	2.52E-05	3.30E-02	6.81E-02
rs62121100	Brain_Substantia_nigra	2	*TMSB4XP2*	3665242	rs62119850	0.9431	3070946	C	G	0.17	2.36E-05	2.47E-02	5.78E-02
rs62121100	Brain_Substantia_nigra	2	*TMSB4XP2*	3665242	rs62119851	0.9431	3071496	A	G	0.17	2.36E-05	2.47E-02	5.78E-02
rs62121100	Brain_Substantia_nigra	2	*TMSB4XP2*	3665242	rs62119853	0.9431	3075633	T	C	0.17	1.99E-05	2.47E-02	5.73E-02
rs62121100	Brain_Substantia_nigra	2	*AC108488.3*	3607765	rs62119854	0.9431	3078552	C	T	0.17	2.13E-05	3.30E-02	6.76E-02
rs62121100	Brain_Substantia_nigra	2	*AC108488.3*	3607765	rs74933641	0.9431	3079764	T	G	0.17	2.38E-05	3.30E-02	6.83E-02
rs62121100	Brain_Substantia_nigra	2	*AC142528.1*	3525007	rs61654279	0.9431	3082049	T	C	0.17	2.53E-05	3.75E-02	7.40E-02
rs62121100	Brain_Substantia_nigra	2	*TMSB4XP2*	3665242	rs61654279	0.9431	3082049	T	C	0.17	2.53E-05	3.00E-02	6.54E-02
rs62121100	Brain_Substantia_nigra	2	*AC142528.1*	3525007	rs62119857	0.9431	3082113	C	G	0.17	2.28E-05	3.75E-02	7.33E-02

rs62121100	Brain_Substantia_nigra	2	*TMSB4XP2*	3665242	rs62119857	0.9431	3082113	C	G	0.17	2.28E-05	3.00E-02	6.47E-02
rs62121100	Brain_Substantia_nigra	2	*TMSB4XP2*	3665242	rs62119859	1.0000	3084061	A	G	0.18	9.91E-06	1.73E-02	4.39E-02
rs62121100	Brain_Substantia_nigra	2	*TMSB4XP2*	3665242	rs75418685	1.0000	3084835	T	C	0.18	9.81E-06	2.47E-02	5.34E-02
rs62121100	Brain_Substantia_nigra	2	*TMSB4XP2*	3665242	rs62121061	0.9919	3085094	C	T	0.18	1.75E-05	2.47E-02	5.64E-02
rs62121100	Brain_Substantia_nigra	2	*TMSB4XP2*	3665242	rs62121062	0.9918	3087273	C	T	0.18	1.12E-05	2.35E-02	5.26E-02
rs62121100	Brain_Substantia_nigra	2	*TMSB4XP2*	3665242	rs62121098	1.0000	3092676	G	C	0.18	1.12E-05	1.77E-02	4.50E-02
rs62121100	Brain_Substantia_nigra	2	*TMSB4XP2*	3665242	rs62121099	1.0000	3092721	C	T	0.18	1.12E-05	2.47E-02	5.40E-02
rs62121100	Brain_Substantia_nigra	2	*TMSB4XP2*	3665242	rs62121100	1.0000	3093952	G	T	0.18	8.44E-06	2.51E-02	5.30E-02
rs1809136	Brain_Amygdala	2	*NOL10*	10770496	rs1809136	1.0000	11152180	C	G	0.93	9.99E-06	1.71E-02	3.85E-02
rs1809136	Brain_Amygdala	2	*RP11-791G15.2*	10909092	rs1809136	1.0000	11152180	C	G	0.93	9.99E-06	2.43E-02	4.75E-02
rs1809136	Brain_Amygdala	2	*PDIA6*	10952755	rs1809136	1.0000	11152180	C	G	0.93	9.99E-06	4.29E-02	6.86E-02
rs1809136	Brain_Amygdala	2	*E2F6*	11595399	rs1809136	1.0000	11152180	C	G	0.93	9.99E-06	6.29E-04	8.21E-03
rs1809136	Brain_Amygdala	2	*GREB1*	11728578	rs1809136	1.0000	11152180	C	G	0.93	9.99E-06	1.31E-02	3.31E-02
rs1809136	Brain_Amygdala	2	*NOL10*	10770496	rs4669673	0.9361	11153383	A	T	0.93	4.17E-05	3.23E-03	2.05E-02
rs1809136	Brain_Amygdala	2	*RP11-791G15.2*	10909092	rs4669673	0.9361	11153383	A	T	0.93	4.17E-05	1.32E-02	3.87E-02
rs1809136	Brain_Amygdala	2	*PDIA6*	10952755	rs4669673	0.9361	11153383	A	T	0.93	4.17E-05	3.24E-02	6.31E-02
rs1809136	Brain_Amygdala	2	*E2F6*	11595399	rs4669673	0.9361	11153383	A	T	0.93	4.17E-05	1.77E-02	4.49E-02
rs1809136	Brain_Amygdala	2	*GREB1*	11728578	rs4669673	0.9361	11153383	A	T	0.93	4.17E-05	4.08E-02	7.24E-02
rs1809136	Brain_Amygdala	2	*NOL10*	10770496	rs4669674	0.9361	11153805	A	G	0.93	4.06E-05	3.23E-03	2.04E-02
rs1809136	Brain_Amygdala	2	*RP11-791G15.2*	10909092	rs4669674	0.9361	11153805	A	G	0.93	4.06E-05	1.32E-02	3.85E-02
rs1809136	Brain_Amygdala	2	*PDIA6*	10952755	rs4669674	0.9361	11153805	A	G	0.93	4.06E-05	3.24E-02	6.29E-02
rs1809136	Brain_Amygdala	2	*E2F6*	11595399	rs4669674	0.9361	11153805	A	G	0.93	4.06E-05	1.77E-02	4.48E-02
rs1809136	Brain_Amygdala	2	*GREB1*	11728578	rs4669674	0.9361	11153805	A	G	0.93	4.06E-05	4.08E-02	7.23E-02
rs1809136	Brain_Amygdala	2	*NOL10*	10770496	rs6432163	0.9361	11159080	C	A	0.93	4.88E-05	3.23E-03	2.12E-02
rs1809136	Brain_Amygdala	2	*RP11-791G15.2*	10909092	rs6432163	0.9361	11159080	C	A	0.93	4.88E-05	1.32E-02	3.95E-02
rs1809136	Brain_Amygdala	2	*PDIA6*	10952755	rs6432163	0.9361	11159080	C	A	0.93	4.88E-05	3.24E-02	6.40E-02
rs1809136	Brain_Amygdala	2	*E2F6*	11595399	rs6432163	0.9361	11159080	C	A	0.93	4.88E-05	1.77E-02	4.58E-02
rs1809136	Brain_Amygdala	2	*GREB1*	11728578	rs6432163	0.9361	11159080	C	A	0.93	4.88E-05	4.08E-02	7.34E-02
rs1809136	Brain_Anterior_cingulate_cortex_BA24	2	*KLF11*	10188761	rs4669673	0.9361	11153383	A	T	0.93	4.17E-05	4.16E-02	7.33E-02
rs1809136	Brain_Anterior_cingulate_cortex_BA24	2	*PQLC3*	11307162	rs4669673	0.9361	11153383	A	T	0.93	4.17E-05	2.78E-02	5.77E-02
rs1809136	Brain_Anterior_cingulate_cortex_BA24	2	*KLF11*	10188761	rs4669674	0.9361	11153805	A	G	0.93	4.06E-05	4.16E-02	7.31E-02
rs1809136	Brain_Anterior_cingulate_cortex_BA24	2	*PQLC3*	11307162	rs4669674	0.9361	11153805	A	G	0.93	4.06E-05	2.78E-02	5.76E-02
rs1809136	Brain_Anterior_cingulate_cortex_BA24	2	*KLF11*	10188761	rs6432163	0.9361	11159080	C	A	0.93	4.88E-05	4.16E-02	7.42E-02
rs1809136	Brain_Anterior_cingulate_cortex_BA24	2	*PQLC3*	11307162	rs6432163	0.9361	11159080	C	A	0.93	4.88E-05	2.78E-02	5.87E-02
rs1809136	Brain_Caudate_basal_ganglia	2	*RN7SL832P*	10831347	rs1809136	1.0000	11152180	C	G	0.93	9.99E-06	1.66E-02	3.78E-02
rs1809136	Brain_Caudate_basal_ganglia	2	*RRM2*	10267000	rs4669673	0.9361	11153383	A	T	0.93	4.17E-05	5.00E-02	8.23E-02
rs1809136	Brain_Caudate_basal_ganglia	2	*RN7SL832P*	10831347	rs4669673	0.9361	11153383	A	T	0.93	4.17E-05	1.74E-02	4.46E-02
rs1809136	Brain_Caudate_basal_ganglia	2	*RRM2*	10267000	rs4669674	0.9361	11153805	A	G	0.93	4.06E-05	5.00E-02	8.21E-02
rs1809136	Brain_Caudate_basal_ganglia	2	*RN7SL832P*	10831347	rs4669674	0.9361	11153805	A	G	0.93	4.06E-05	1.74E-02	4.45E-02
rs1809136	Brain_Caudate_basal_ganglia	2	*RRM2*	10267000	rs6432163	0.9361	11159080	C	A	0.93	4.88E-05	5.00E-02	8.32E-02

rs1809136	Brain_Caudate_basal_ganglia	2	*RN7SL832P*	10831347	rs6432163	0.9361	11159080	C	A	0.93	4.88E-05	1.74E-02	4.55E-02
rs1809136	Brain_Cerebellar_Hemisphere	2	*RP11-254F7.2*	10180004	rs1809136	1.0000	11152180	C	G	0.93	9.99E-06	3.36E-03	1.65E-02
rs1809136	Brain_Cerebellar_Hemisphere	2	*C2orf50*	11280047	rs1809136	1.0000	11152180	C	G	0.93	9.99E-06	1.71E-02	3.85E-02
rs1809136	Brain_Cerebellar_Hemisphere	2	*RP11-254F7.2*	10180004	rs4669673	0.9361	11153383	A	T	0.93	4.17E-05	1.71E-03	1.61E-02
rs1809136	Brain_Cerebellar_Hemisphere	2	*RP11-254F7.2*	10180004	rs4669674	0.9361	11153805	A	G	0.93	4.06E-05	1.71E-03	1.60E-02
rs1809136	Brain_Cerebellar_Hemisphere	2	*RP11-254F7.2*	10180004	rs6432163	0.9361	11159080	C	A	0.93	4.88E-05	1.71E-03	1.67E-02
rs1809136	Brain_Cerebellum	2	*ROCK2*	11404171	rs4669673	0.9361	11153383	A	T	0.93	4.17E-05	4.84E-02	8.06E-02
rs1809136	Brain_Cerebellum	2	*ROCK2*	11404171	rs4669674	0.9361	11153805	A	G	0.93	4.06E-05	4.84E-02	8.04E-02
rs1809136	Brain_Cerebellum	2	*ROCK2*	11404171	rs6432163	0.9361	11159080	C	A	0.93	4.88E-05	4.84E-02	8.15E-02
rs1809136	Brain_Cortex	2	*AC007249.3*	10594061	rs1809136	1.0000	11152180	C	G	0.93	9.99E-06	3.75E-02	6.27E-02
rs1809136	Brain_Cortex	2	*ROCK2*	11404171	rs1809136	1.0000	11152180	C	G	0.93	9.99E-06	8.31E-03	2.58E-02
rs1809136	Brain_Cortex	2	*ROCK2*	11404171	rs4669673	0.9361	11153383	A	T	0.93	4.17E-05	1.20E-02	3.68E-02
rs1809136	Brain_Cortex	2	*ROCK2*	11404171	rs4669674	0.9361	11153805	A	G	0.93	4.06E-05	1.20E-02	3.67E-02
rs1809136	Brain_Cortex	2	*ROCK2*	11404171	rs6432163	0.9361	11159080	C	A	0.93	4.88E-05	1.20E-02	3.77E-02
rs1809136	Brain_Frontal_Cortex_BA9	2	*ATP6V1C2*	10893505	rs1809136	1.0000	11152180	C	G	0.93	9.99E-06	1.64E-02	3.75E-02
rs1809136	Brain_Frontal_Cortex_BA9	2	*ODC1*	10584362	rs4669673	0.9361	11153383	A	T	0.93	4.17E-05	1.23E-02	3.72E-02
rs1809136	Brain_Frontal_Cortex_BA9	2	*ATP6V1C2*	10893505	rs4669673	0.9361	11153383	A	T	0.93	4.17E-05	4.61E-02	7.82E-02
rs1809136	Brain_Frontal_Cortex_BA9	2	*C2orf50*	11280047	rs4669673	0.9361	11153383	A	T	0.93	4.17E-05	2.39E-02	5.29E-02
rs1809136	Brain_Frontal_Cortex_BA9	2	*LPIN1*	11892628	rs4669673	0.9361	11153383	A	T	0.93	4.17E-05	4.51E-02	7.71E-02
rs1809136	Brain_Frontal_Cortex_BA9	2	*ODC1*	10584362	rs4669674	0.9361	11153805	A	G	0.93	4.06E-05	1.23E-02	3.71E-02
rs1809136	Brain_Frontal_Cortex_BA9	2	*ATP6V1C2*	10893505	rs4669674	0.9361	11153805	A	G	0.93	4.06E-05	4.61E-02	7.81E-02
rs1809136	Brain_Frontal_Cortex_BA9	2	*C2orf50*	11280047	rs4669674	0.9361	11153805	A	G	0.93	4.06E-05	2.39E-02	5.28E-02
rs1809136	Brain_Frontal_Cortex_BA9	2	*LPIN1*	11892628	rs4669674	0.9361	11153805	A	G	0.93	4.06E-05	4.51E-02	7.70E-02
rs1809136	Brain_Frontal_Cortex_BA9	2	*ODC1*	10584362	rs6432163	0.9361	11159080	C	A	0.93	4.88E-05	1.23E-02	3.81E-02
rs1809136	Brain_Frontal_Cortex_BA9	2	*ATP6V1C2*	10893505	rs6432163	0.9361	11159080	C	A	0.93	4.88E-05	4.61E-02	7.92E-02
rs1809136	Brain_Frontal_Cortex_BA9	2	*C2orf50*	11280047	rs6432163	0.9361	11159080	C	A	0.93	4.88E-05	2.39E-02	5.38E-02
rs1809136	Brain_Frontal_Cortex_BA9	2	*LPIN1*	11892628	rs6432163	0.9361	11159080	C	A	0.93	4.88E-05	4.51E-02	7.81E-02
rs1809136	Brain_Hippocampus	2	*CYS1*	10208989	rs1809136	1.0000	11152180	C	G	0.93	9.99E-06	2.82E-02	5.21E-02
rs1809136	Brain_Hippocampus	2	*ROCK2*	11404171	rs1809136	1.0000	11152180	C	G	0.93	9.99E-06	3.30E-02	5.77E-02
rs1809136	Brain_Hippocampus	2	*NTSR2*	11804297	rs1809136	1.0000	11152180	C	G	0.93	9.99E-06	2.92E-03	1.54E-02
rs1809136	Brain_Hippocampus	2	*CYS1*	10208989	rs4669673	0.9361	11153383	A	T	0.93	4.17E-05	3.33E-02	6.41E-02
rs1809136	Brain_Hippocampus	2	*AC007249.3*	10594061	rs4669673	0.9361	11153383	A	T	0.93	4.17E-05	2.85E-02	5.85E-02
rs1809136	Brain_Hippocampus	2	*NTSR2*	11804297	rs4669673	0.9361	11153383	A	T	0.93	4.17E-05	4.94E-02	8.17E-02
rs1809136	Brain_Hippocampus	2	*CYS1*	10208989	rs4669674	0.9361	11153805	A	G	0.93	4.06E-05	3.33E-02	6.39E-02
rs1809136	Brain_Hippocampus	2	*AC007249.3*	10594061	rs4669674	0.9361	11153805	A	G	0.93	4.06E-05	2.85E-02	5.84E-02
rs1809136	Brain_Hippocampus	2	*NTSR2*	11804297	rs4669674	0.9361	11153805	A	G	0.93	4.06E-05	4.94E-02	8.16E-02
rs1809136	Brain_Hippocampus	2	*CYS1*	10208989	rs6432163	0.9361	11159080	C	A	0.93	4.88E-05	3.33E-02	6.50E-02
rs1809136	Brain_Hippocampus	2	*AC007249.3*	10594061	rs6432163	0.9361	11159080	C	A	0.93	4.88E-05	2.85E-02	5.94E-02
rs1809136	Brain_Hippocampus	2	*NTSR2*	11804297	rs6432163	0.9361	11159080	C	A	0.93	4.88E-05	4.94E-02	8.26E-02
rs1809136	Brain_Hypothalamus	2	*ATP6V1C2*	10893505	rs1809136	1.0000	11152180	C	G	0.93	9.99E-06	4.93E-02	7.54E-02

rs1809136	Brain_Hypothalamus	2	*RP11-320M2.1*	10590136	rs4669673	0.9361	11153383	A	T	0.93	4.17E-05	4.36E-02	7.55E-02
rs1809136	Brain_Hypothalamus	2	*ATP6V1C2*	10893505	rs4669673	0.9361	11153383	A	T	0.93	4.17E-05	1.97E-02	4.75E-02
rs1809136	Brain_Hypothalamus	2	*RP11-320M2.1*	10590136	rs4669674	0.9361	11153805	A	G	0.93	4.06E-05	4.36E-02	7.54E-02
rs1809136	Brain_Hypothalamus	2	*ATP6V1C2*	10893505	rs4669674	0.9361	11153805	A	G	0.93	4.06E-05	1.97E-02	4.74E-02
rs1809136	Brain_Hypothalamus	2	*RP11-320M2.1*	10590136	rs6432163	0.9361	11159080	C	A	0.93	4.88E-05	4.36E-02	7.64E-02
rs1809136	Brain_Hypothalamus	2	*ATP6V1C2*	10893505	rs6432163	0.9361	11159080	C	A	0.93	4.88E-05	1.97E-02	4.84E-02
rs1809136	Brain_Nucleus_accumbens_basal_ganglia	2	*RN7SL832P*	10831347	rs4669673	0.9361	11153383	A	T	0.93	4.17E-05	4.65E-02	7.86E-02
rs1809136	Brain_Nucleus_accumbens_basal_ganglia	2	*RN7SL832P*	10831347	rs4669674	0.9361	11153805	A	G	0.93	4.06E-05	4.65E-02	7.85E-02
rs1809136	Brain_Nucleus_accumbens_basal_ganglia	2	*RN7SL832P*	10831347	rs6432163	0.9361	11159080	C	A	0.93	4.88E-05	4.65E-02	7.96E-02
rs1809136	Brain_Putamen_basal_ganglia	2	*KCNF1*	11053206	rs1809136	1.0000	11152180	C	G	0.93	9.99E-06	2.97E-02	5.39E-02
rs1809136	Brain_Putamen_basal_ganglia	2	*NTSR2*	11804297	rs1809136	1.0000	11152180	C	G	0.93	9.99E-06	1.50E-02	3.56E-02
rs1809136	Brain_Putamen_basal_ganglia	2	*NTSR2*	11804297	rs4669673	0.9361	11153383	A	T	0.93	4.17E-05	2.01E-02	4.82E-02
rs1809136	Brain_Putamen_basal_ganglia	2	*NTSR2*	11804297	rs4669674	0.9361	11153805	A	G	0.93	4.06E-05	2.01E-02	4.80E-02
rs1809136	Brain_Putamen_basal_ganglia	2	*NTSR2*	11804297	rs6432163	0.9361	11159080	C	A	0.93	4.88E-05	2.01E-02	4.91E-02
rs1809136	Brain_Spinal_cord_cervical_c-1	2	*RRM2*	10267000	rs1809136	1.0000	11152180	C	G	0.93	9.99E-06	2.32E-02	4.62E-02
rs1809136	Brain_Substantia_nigra	2	*ATP6V1C2*	10893505	rs1809136	1.0000	11152180	C	G	0.93	9.99E-06	2.13E-02	4.38E-02
rs1809136	Brain_Substantia_nigra	2	*RRM2*	10267000	rs4669673	0.9361	11153383	A	T	0.93	4.17E-05	3.32E-02	6.40E-02
rs1809136	Brain_Substantia_nigra	2	*ATP6V1C2*	10893505	rs4669673	0.9361	11153383	A	T	0.93	4.17E-05	6.35E-03	2.73E-02
rs1809136	Brain_Substantia_nigra	2	*RRM2*	10267000	rs4669674	0.9361	11153805	A	G	0.93	4.06E-05	3.32E-02	6.39E-02
rs1809136	Brain_Substantia_nigra	2	*ATP6V1C2*	10893505	rs4669674	0.9361	11153805	A	G	0.93	4.06E-05	6.35E-03	2.72E-02
rs1809136	Brain_Substantia_nigra	2	*RRM2*	10267000	rs6432163	0.9361	11159080	C	A	0.93	4.88E-05	3.32E-02	6.49E-02
rs1809136	Brain_Substantia_nigra	2	*ATP6V1C2*	10893505	rs6432163	0.9361	11159080	C	A	0.93	4.88E-05	6.35E-03	2.80E-02
rs1809136	Whole_Blood	2	*RP11-245G13.2*	11021819	rs1809136	1.0000	11152180	C	G	0.93	9.99E-06	5.78E-03	2.14E-02
rs1809136	Whole_Blood	2	*RP11-254F7.2*	10180004	rs4669673	0.9361	11153383	A	T	0.93	4.17E-05	4.17E-02	7.34E-02
rs1809136	Whole_Blood	2	*RP11-254F7.2*	10180004	rs4669674	0.9361	11153805	A	G	0.93	4.06E-05	4.17E-02	7.33E-02
rs1809136	Whole_Blood	2	*RP11-254F7.2*	10180004	rs6432163	0.9361	11159080	C	A	0.93	4.88E-05	4.17E-02	7.43E-02
